# Rotatable Small Permanent Magnet Array for Ultra-Low Field Nuclear Magnetic Resonance Instrumentation: A Concept Study

**DOI:** 10.1371/journal.pone.0157040

**Published:** 2016-06-06

**Authors:** Michael W. Vogel, Andrea Giorni, Viktor Vegh, Ruben Pellicer-Guridi, David C. Reutens

**Affiliations:** Centre for Advanced Imaging, University of Queensland, Brisbane, Queensland, Australia; National Taiwan University, TAIWAN

## Abstract

**Object:**

We studied the feasibility of generating the variable magnetic fields required for ultra-low field nuclear magnetic resonance relaxometry with dynamically adjustable permanent magnets. Our motivation was to substitute traditional electromagnets by distributed permanent magnets, increasing system portability.

**Materials and Methods:**

The finite element method (COMSOL^®^) was employed for the numerical study of a small permanent magnet array to calculate achievable magnetic field strength, homogeneity, switching time and magnetic forces. A manually operated prototype was simulated and constructed to validate the numerical approach and to verify the generated magnetic field.

**Results:**

A concentric small permanent magnet array can be used to generate strong sample pre-polarisation and variable measurement fields for ultra-low field relaxometry via simple prescribed magnet rotations. Using the array, it is possible to achieve a pre-polarisation field strength above 100 mT and variable measurement fields ranging from 20–50 μT with 200 ppm absolute field homogeneity within a field-of-view of 5 x 5 x 5 cubic centimetres.

**Conclusions:**

A dynamic small permanent magnet array can generate multiple highly homogeneous magnetic fields required in ultra-low field nuclear magnetic resonance (NMR) and magnetic resonance imaging (MRI) instruments. This design can significantly reduce the volume and energy requirements of traditional systems based on electromagnets, improving portability considerably.

## Introduction

Nuclear magnetic resonance (NMR) spectroscopy and magnetic resonance imaging (MRI) are non-invasive and non-destructive investigative tools that can provide information from the molecular to the macroscopic scale. These techniques harness the phenomenon of magnetic resonance due to the interaction, within a magnetic field, between precessing nuclear magnetic moments (nuclear spin systems) and electromagnetic radiation. NMR/MRI have a wide range of applications in materials science, structural biology, chemistry and medical imaging [1–3].

Conventional MRI instruments comprise three main components: a superconducting magnet to align the nuclear spins and generate net sample magnetisation; a transmitter/receiver coil system that radiates electromagnetic energy to the nuclear spin system and detects the NMR signal; and gradient coils that enable the encoding of spatial information allowing the generation of three dimensional images [2].

The signal-to-noise ratio (SNR) achieved in NMR/MRI is proportional to the magnitude of net sample magnetisation. Hence, the quality of NMR/MRI data is dependent on the strength and homogeneity of the main magnetic field (commonly referred to as the **B**_**0**_ field). Superconducting magnets have been utilised to increase field strength. These increase the bulk and cost of purchase, operation and maintenance of NMR/MRI instruments.

Partly in response to these drawbacks, over the last decade there has been growing interest in ultra-low magnetic field (ULF) NMR/MRI, which uses a main magnet field strength of less than 10 mT [4–14]. Potential advantages of ULF over high field NMR/MRI instruments include greater absolute magnetic field homogeneity, simple and low cost instrumentation and low power consumption [15]. ULF NMR/MRI offers the possibility of important new applications such as the ability to image in the presence of metal, for example in trauma, disaster and battlefield applications. At ULF, the Larmor frequency overlaps with a range of molecular and physiological processes such as protein folding, slow diffusion, molecular tumbling and enzyme catalysis which are difficult to observe at high field because of the large frequency mismatch [13]. This raises the possibility of new imaging paradigms sensitised to these processes. In addition, because superconducting magnets are not required, the instruments may be more portable, allowing ULF instruments to be used in remote locations [13].

Although based on the same fundamental principles of magnetic resonance as high field NMR/MRI, ULF instruments are set up differently. Prior to the measurement, sample magnetisation is generated by a pulsed magnetic field approximately three orders of magnitude higher (~0.05–0.1 T) than the Earth’s field. This technique is known as sample pre-polarisation and is a key strategy in ULF research to overcome low SNR which still severely restricts ULF-NMR/MRI applications [5, 9, 13]. Highly sensitive magnetometers are also used to increase SNR. Often, excitation pulses are not used to trigger the ULF-NMR/MRI signal. Instead, the ULF-NMR/MRI signal is generated and detected in the presence of a second magnetic field, the measurement field, that is perpendicular to the pre-polarisation field.

In most instruments, the magnetic fields in ULF-NMR/MRI instruments are generated using resistive coils, which have high power consumption and heat production [11–13, 16]. Moreover, the presence of highly conductive materials in resistive coils contributes to signal loss due to sample heating effects, residual coil noise, transients and eddy currents, and destructive interference effects [12, 13, 17]. Vesanen et al. addressed these problems by using superconducting coils [18]. Here we propose a permanent magnet solution using Halbach arrays,

Halbach arrays are a versatile arrangement of permanent magnets that can be used to generate strong, highly homogeneous magnetic fields in a field of view (FOV) that is small compared to overall array volume [19–21]. Halbach array technology has led to the development of new generations of benchtop and handheld NMR instruments, with field strength above 3T and inhomogeneity of less than 0.01% (100 ppm) [19, 22–24].

Permanent magnets do not require electric current flow to generate magnets fields. Hence, sample heating due to energy dissipation in resistive material is avoided, cooling devices obviated and power consumption significantly reduced compared to resistive coil technology. Moreover, the conductivity of the material used in magnets is much lower than materials like copper used in resistive coils. Hence, eddy current effects from rapid changes in magnetic field, which can lead to signal artefacts and noise, are reduced.

A Halbach array has recently been introduced in a prototype portable MRI scanner suitable for human brains (FOV = 16 cm) [25]. The array was used to generate a static field (**B**_**0**_ = 77 mT) within the volume which was quite inhomogeneous (1% inhomogeneity; ~10000 ppm) due to presence of fringe fields originating from both ends of the Halbach array which has a length and diameter of ~ 36 cm. The **B**_**0**_ inhomogeneity was exploited to encode spatial information and rotation of the Halbach array about the sample was required to generate a 2D image [25].

In all current designs of NMR/MRI instruments using Halbach arrays, resistive coils and excitation pulses are employed to generate signals and gradients. Our new approach is an extension of the concept of the Halbach array that obviates the need for resistive coils for low and ULF NMR/MRI by introducing a dynamic adjustable small permanent magnet array (SPMA) to generate and switch between multiple magnetic fields. This is achieved by prescribed rotations of individual magnets, allowing adjustments of magnetic field magnitudes and orientations.

**Fig 1:**
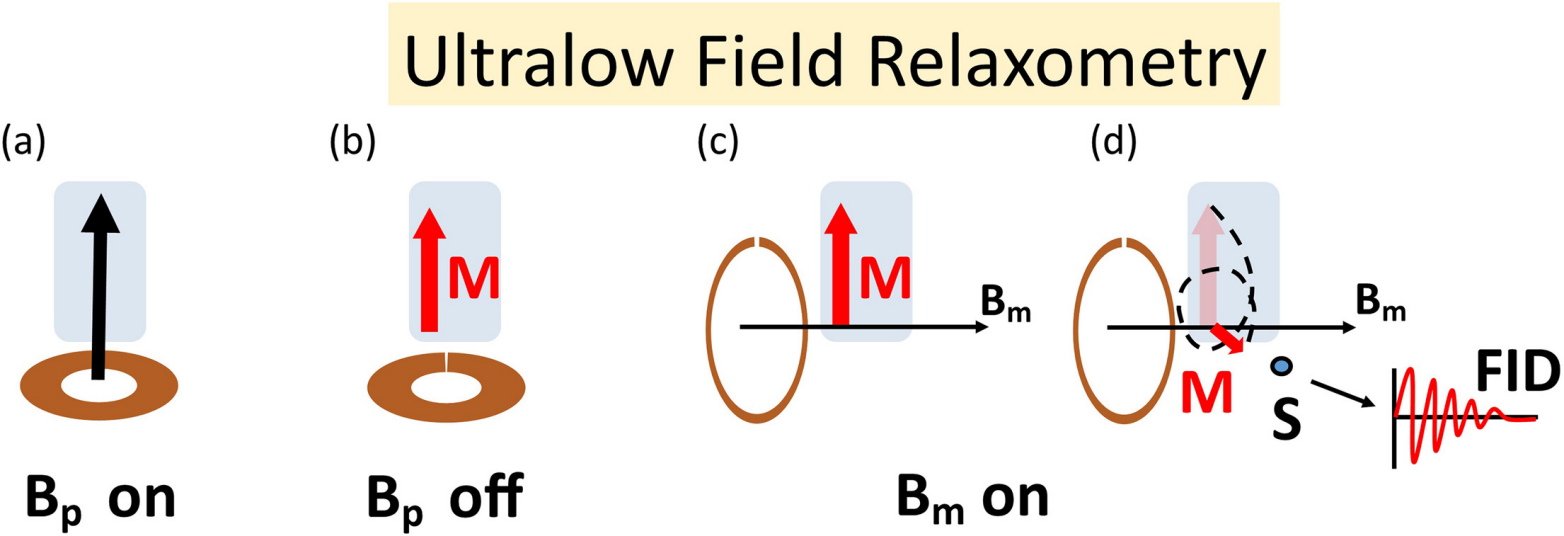
Schematic presentation of ULF relaxometry measurement. (a) Pre-polarisation coil is switched on to generate **B**_**p**_. (b) The net magnetization M reaches its maximum right before the pre-polarisation coil is switched off. (c) The measurement field **B**_**m**_, perpendicular to **B**_**p**_ is switched on. (d) The net magnetisation vector **M** precesses about **B**_**m**_ and decays, the sample demagnetises. The localised magnetic field sensor (**S**) detects the sample signal (**FID**) during demagnetisation

In this study, we examined the potential of SPMAs for ULF-NMR relaxometry. By applying the finite element method (FEM) to Maxwell’s equation, we determined the magnetic fields generated by the dynamic SPMA and analysed it in terms of achievable field strength and field homogeneity. In addition, a manually operated SPMA was simulated and built to demonstrate the ability to generate the magnetic fields required for ULF relaxometry. An ULF relaxometry instrument for measuring the longitudinal (T_1_) and transversal relaxation (T_2_) time in a sample, requires *two* perpendicular and dynamic switchable magnetic fields: the pre-polarisation field **B**_**p**_ and the measurement field **B**_**m**_. A schematic representation of the application of **B**_**p**_ and **B**_**m**_ to perform a basic ULF relaxometry measurement is shown in Fig 1: The pulsed **B**_**p**_ (Fig 1A) with magnitudes typically ranging from 30–70 milliteslas (mT) generates the net sample magnetisation **M**, according to Curie’s law (Fig 1B). The orientation and the magnitude of **B**_**m**_ defines the axis of precession of **M** and the Larmor frequency, respectively (Fig 1C). After **B**_**p**_ is switched off, the precession of **M** about **B**_**m**_ generates the sample signal known as free induction decay (FID) and is detected by the sensor (S) (Fig 1D). The duration and measurability of the FID depends on the demagnetisation characteristics of the sample and sensor location and orientation [17].

## Materials and Methods

### SPMA design

**Fig 2:**
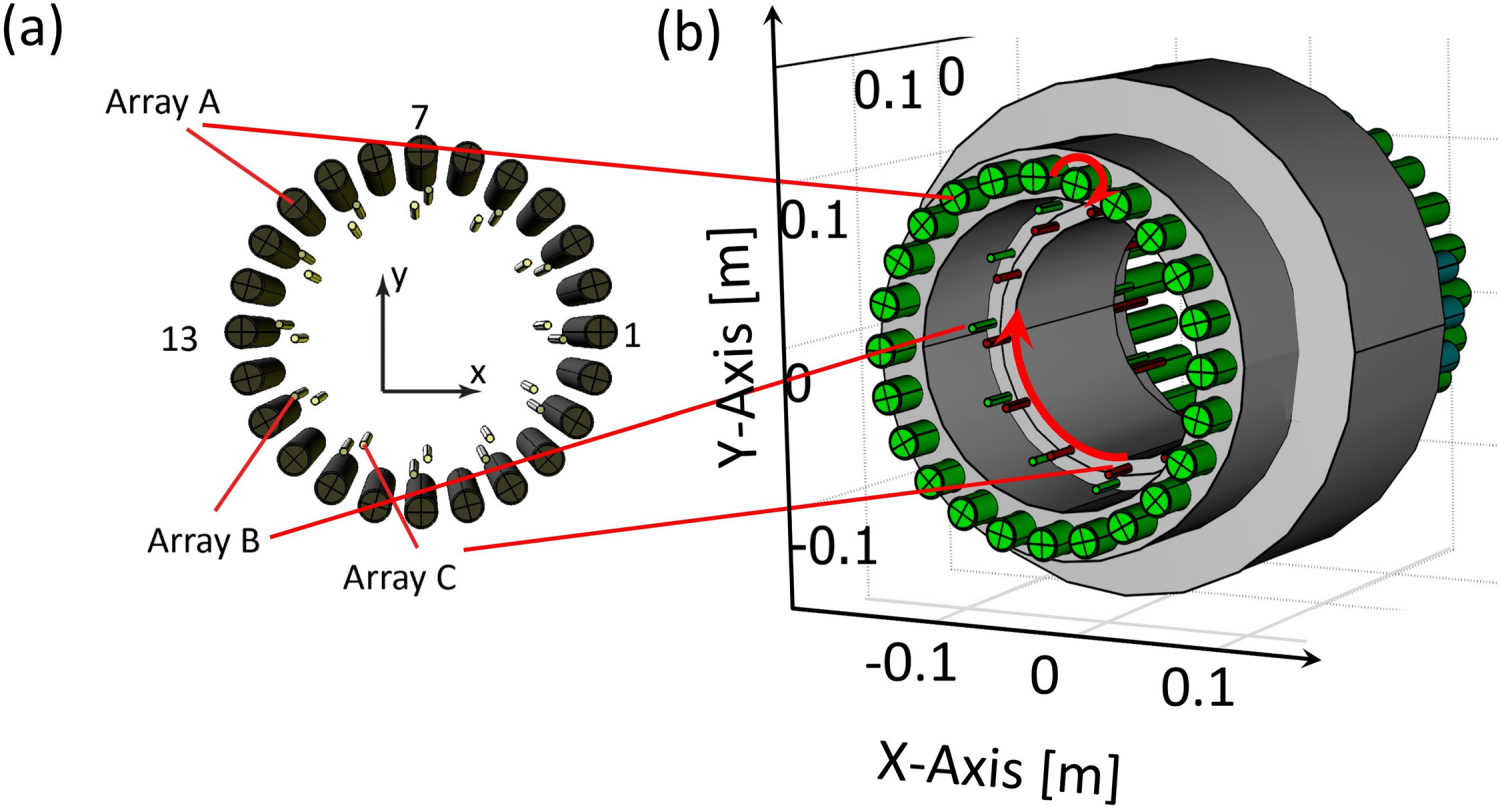
Setup of the dynamic SPMA model for ULF relaxometry. (a) The SPMA model consists of three concentric cylindrical arrays with transversely (x-y plane) magnetised rods. Array *A*, required for pre-polarisation, consists of 24 magnets; Arrays *B* and *C*, required for generating the variable measurement field, consist of 12 magnets each. The origin of the coordinate system was positioned at the centre of the arrays and the axes of the arrays were aligned along the z-axis. In the Halbach configuration, each magnet has a specific orientation different to the neighbouring magnets. To study the magnetic forces, the magnets of array *A* were numbered counter-clockwise starting at the right-hand-side. (b) Side view indicating the concentric SPMA setup and sizes. Array *A* is fixed but each magnet rotates individually along its own axis in the z-coordinate direction (small red circular arrow). Arrays *B* and *C* (with fixed magnet orientation) rotate about the z-axis, indicated by the large red circular arrow

The dynamic SPMA for ULF relaxometry is shown in Fig 2. It consists of cylindrical magnets of finite length, transversely magnetised (i.e. in the *x-y* plane) arranged in three concentric cylindrical arrays as indicated in Fig 2A. Each magnet of array *A* was assumed to be individually pivot-mounted about its own axis in the z-coordinate direction to allow the generation of different magnetic field configurations by prescribed rotations of each magnet. In contrast, the orientation of each magnet within array *B* and *C* was fixed but the two arrays were able to rotate about the z-axis (see Fig 2B).

Our SPMA design is based on strong, highly homogeneous magnetic fields generated by a Halbach dipole cylinder or array [23]. The three concentric cylindrical arrays, *A*, *B* and *C* generate the two mutually perpendicular magnetic fields required for ULF relaxometry measurements: **B**_**p**_ (array *A*) and **B**_**m**_ (array *B* and *C*). Throughout this paper it is assumed that **B**_**p**_ is oriented along the *x*-axis and **B**_**m**_ along the *y*-axis. Each cylindrical magnet within the SPMA is transversely magnetised (x-y plane) with remanent magnetisation **B**_**r**_. The following parameters were used when designing the array: field of view (FOV) within the centre of the SPMA 5 x 5 x 5 cm^3^; **B**_**p**_ magnitude >100 mT; **B**_**m**_ magnitude 20–50 μT (852–2130 Hz). The FOV was chosen to be sufficient for both ULF-NMR measurements and for a small ULF imaging device. **B**_**m**_ corresponds to the proton (^1^H) Larmor frequency *ω*_*L*_ as determined by the Larmor equation

$$\omega_{L}=2\pi f_{L}=\gamma B_{m}$$ (1)

with the units for ω_*L*_ being rad/sec and f_*L*_ being Hz. γ is the gyromagnetic ratio, which is 267.5 x 10^6^ rad/sec/T for protons.

### Magnetisation pattern

**Fig 3:**
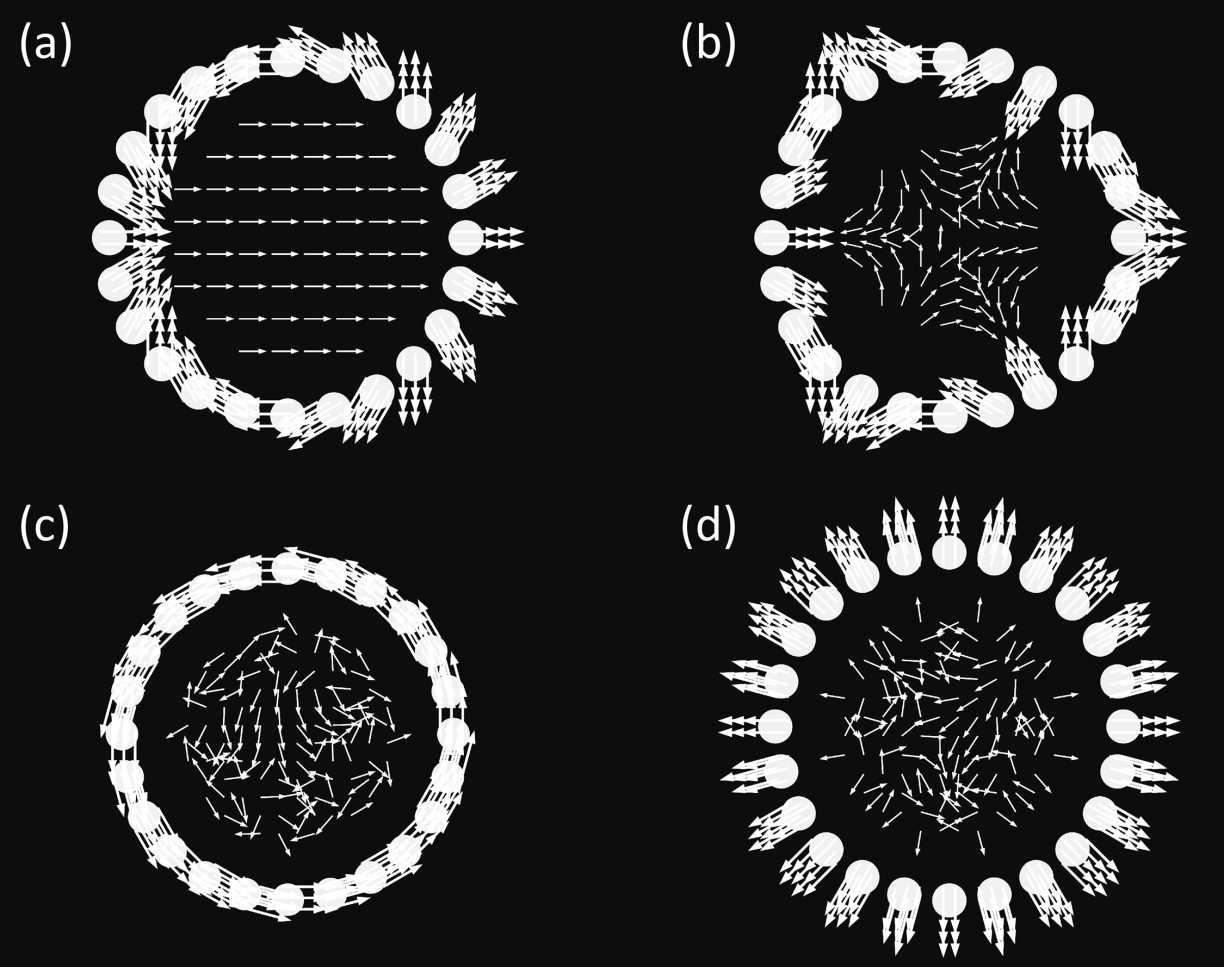
Definition and visualisation of the magnetisation patterns considered in this study. (a) *Halbach*, (b) *reverse Halbach*, (c) *tangential* and (d) *radial*. Shown as vector plots are the magnet remanent magnetisation (thick white arrows) and the normalized magnetic field distribution (thin white arrows) in the centre of the SPMA. The *Halbach pattern* leads to a strong, highly directional magnetic field, while the other patterns lead to non-directional, weak fields.

Four different magnetisation patterns were considered in this study. They were created by prescribed rotations of each cylindrical magnet in array *A* (Fig 3), with **B**_**r**_ of each magnet indicated by white arrows: *Halbach* (Fig 3A), *reverse Halbach* (Fig 3B), *tangential* (Fig 3C) and *radial* (Fig 3D). The *Halbach* pattern is known to achieve a strong homogeneous magnetic field in the centre of the array *A*, while the lowest field strength or field cancellation (indicated by irregular field distribution in the centre) is achieved with the *reverse Halbach*, *tangential* or *radial* magnetisation patterns. Different magnetisation patterns and numbers of permanent magnets in the array lead to different magnetic field distributions, field strength and homogeneity in the centre of the array. All of these variations affect suitability for ULF relaxometry measurements. A study on the impact of such effects is provided in the following sections.

### Simulation environment

The complexity of the fields associated with different conformations of the SPMA precluded an analytical approach. Hence we undertook a rigorous numerical analysis using COMSOL^®^ (version 4.3b, AC/DC module, Magnetostatic), a commercial finite element method (FEM) simulation environment with a computer-aided design interface for 3D model design. Simulations were carried out using an x64-based 16 core PC (Dell Precision T7600) with 128 GB of RAM. In the FEM simulation, the SPMA model was discretised in 3D-tetrahedral meshes using a predetermined mesh distribution and density that had been optimised using COMSOL. Mesh density was manually increased to achieve sub-millimetre spatial resolution in the centre of the array. The number of mesh points generally ranged between 40–50 million which ensured convergent and accurate results within reasonable time frames. The size of the cylindrically shaped computational window (diameter 1.2 m, length 2.1 m) was set to be sufficiently large to be able to model the SPMA (diameter 0.4 m, length 0.7 m) and to minimise numerical errors due to discontinuities. The standard COMSOL Neumann boundary condition was used to represent magnetic shielding implemented in a previously described ULF instrument developed at the Centre for Advanced Imaging [17], with thickness *d* = 12 cm and permeability μ_*r*_ = 5500. The relative permeability of the material in the magnets was set to 1.05 and for the surrounding environment (air) it was 1 (see S1 File).

### Pre-polarisation array (array *A*)

The outer Array *A* which generates **B**_**p**_, had an assumed fixed radius *R*_*A*_ = 11cm and array length *L* = 70 cm. It consisted of *n* identical cylindrical magnets each with a remanent magnetic field strength (**B**_**r**_) of 1 Tesla (T). Notably, other commercially available magnet cross-sections lead to equivalent qualitative results provided that magnet size is small compared to the distance to the centre of the array. An ideal Halbach array is characterized by a continuous change in the azimuthal direction of the magnetisation vector, which at present cannot be achieved. Hence, cylindrical Halbach arrays are discretised by identical magnets with constant **B**_**r**_ and the approximation improved by progressively increasing the number of magnets along the circumference. In this study *n* = 12, 16 and 24 magnets were considered.

**Fig 4:**
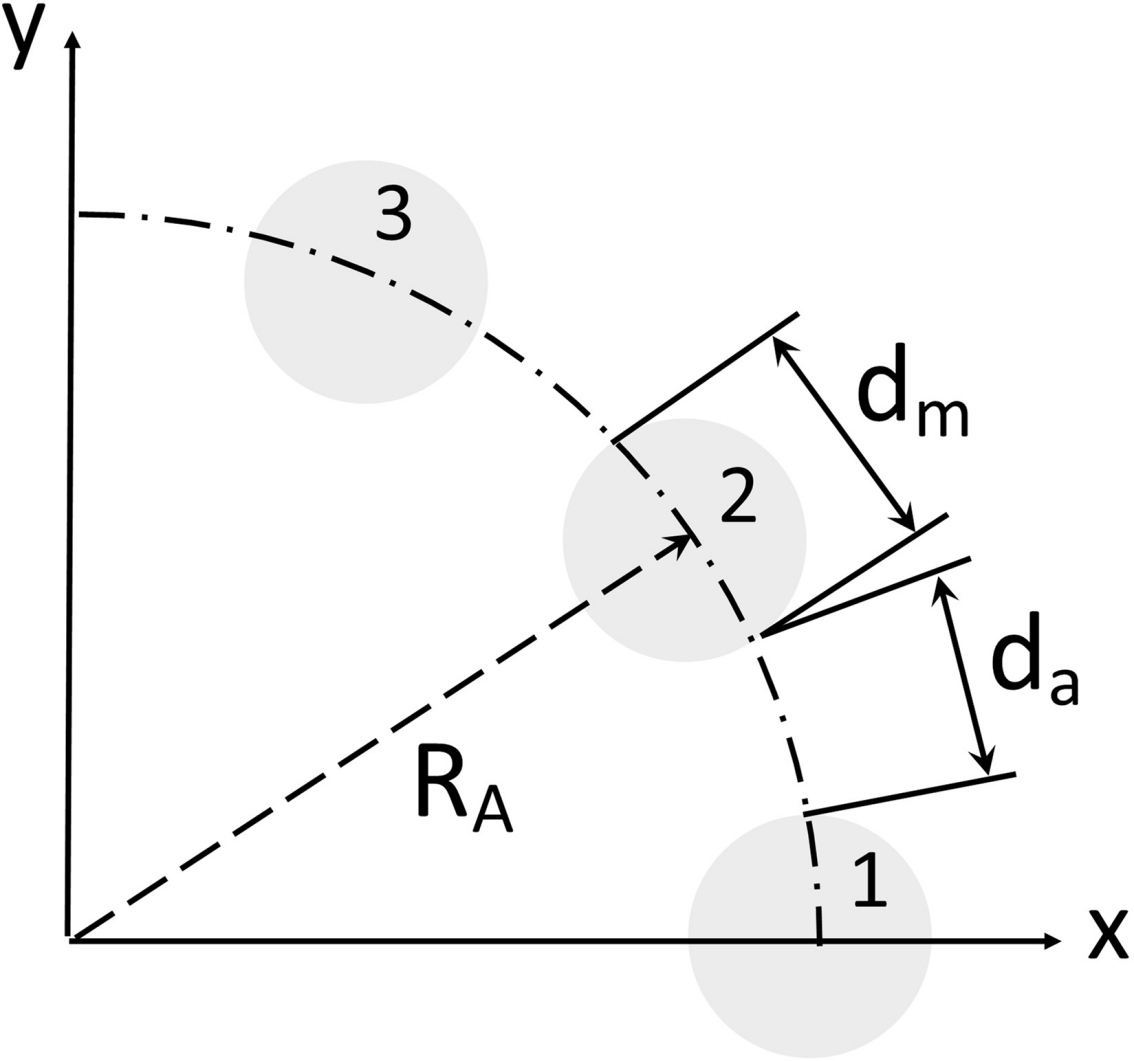
Section of an array with radius R_A_. Each cylindrical magnet, numbered in a counter-clockwise direction and with diameter d_m_, is evenly arranged along the circumference to ensure equidistant air gaps. The fill factor is defined as the ratio between d_m_ and d_a_. In this example d_m_ equals d_a_ and the fill factor is 0.5

The array radius *R*_*A*_ was fixed to ensure that the SPMA fits within the magnetic shielding device. To allow the performance of SPMAs with different numbers of magnets to be compared while keeping the overall size constant, a fill factor was introduced. This factor quantifies the ratio of magnetic material to air gap (*d*_*m*_ to *d*_*a*_) along the circumference (see Fig 4). A fill factor of 0.75, for instance, corresponds to 75% occupancy by magnets (*d*_*m*_) and 25% by air (*d*_*a*_) along the circumference. Magnet diameter *d*_*m*_ is thus dependent on the total number of magnets *n* and is approximately:

$$d_{m}=2\pi \frac{Fillfactor∙R_{A}}{n}$$ (2)

### Measurement array (arrays B and C)

**Fig 5:**
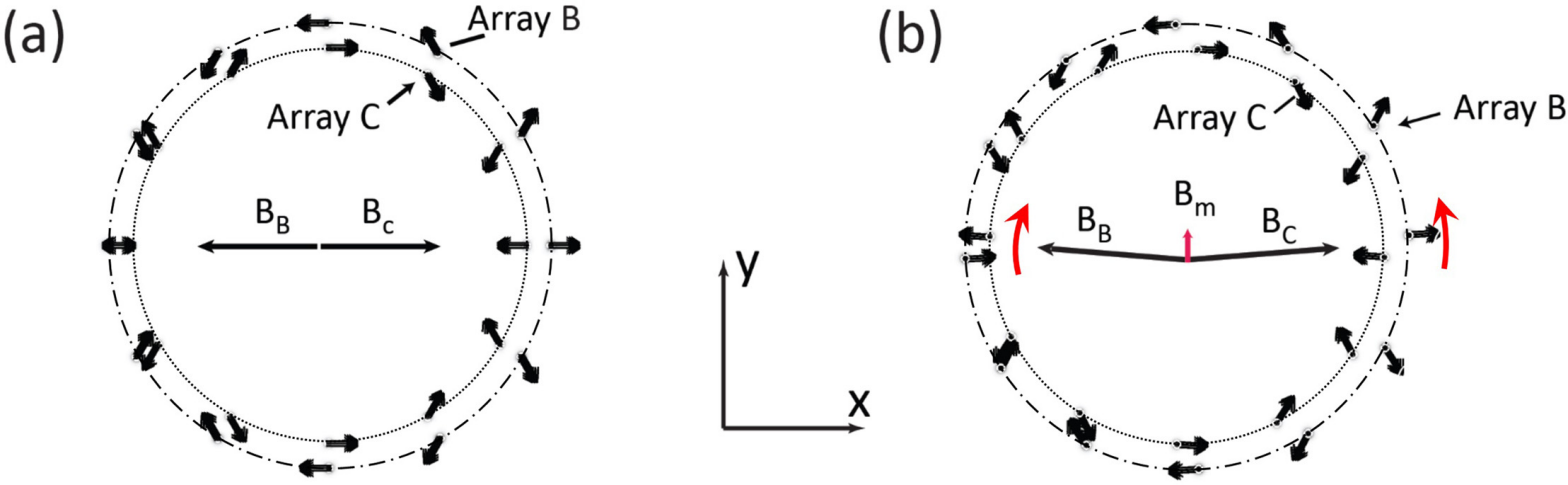
Principle of generating B_m_ simulated with COMSOL. (a) Two concentric arrays *B* and *C* (R_*B*_ = 9 cm and R_*C*_ = 8 cm), each with Halbach magnetisation pattern (see Fig 3A), generate opposing magnetic fields **B**_**B**_ and **B**_**C**_. If their magnitude is matched, the field is nearly cancelled in the centre. (b) By rotating array *B* and *C* respectively clockwise and counter-clockwise about the axis of symmetry of the SPMA (see red arrows), the x-component of the magnetic field remains cancelled but a y-component, **B**_**m**_, is generated

The measurement field **B**_**m**_ was generated by superimposing two magnetic fields generated by arrays *B* (**B**_**B**_) and *C* (**B**_**C**_) with radii R_*B*_ = 8 cm and R_*C*_ = 9 cm, respectively, each with the fixed *Halbach* pattern. As with the pre-polarisation array *A*, this arrangement ensured a highly directional and homogeneous magnetic field along the y-axis. Magnetic fields from the two arrays cancel within the FOV when (a) the magnetic field magnitudes **B**_**B**_ and **B**_**C**_ are matched and (b) their directions are opposite (Fig 5A). By rotating arrays *B* and *C* by the same angle but in opposite directions about the *z*-axis, the total magnetic field remains parallel to the y-axis and comprises the measurement field **B**_**m**_ (Fig 5B).

The most important magnetic field properties for NMR experiments are field strength, directionality and homogeneity [21]. In the case of the ULF relaxometer proposed here the qualitative targets to optimize the SNR were:

- uniform field direction and maximum magnitude of **B**_**p**_ during sample pre-polarisation (*switched on*).
- minimum magnitude for **B**_**p**_ during the measurement period.
- uniform field direction and homogeneous *variable* measurement field **B**_**m**_ (arrays *B* and *C*).

### Manual SPMA

**Fig 6:**
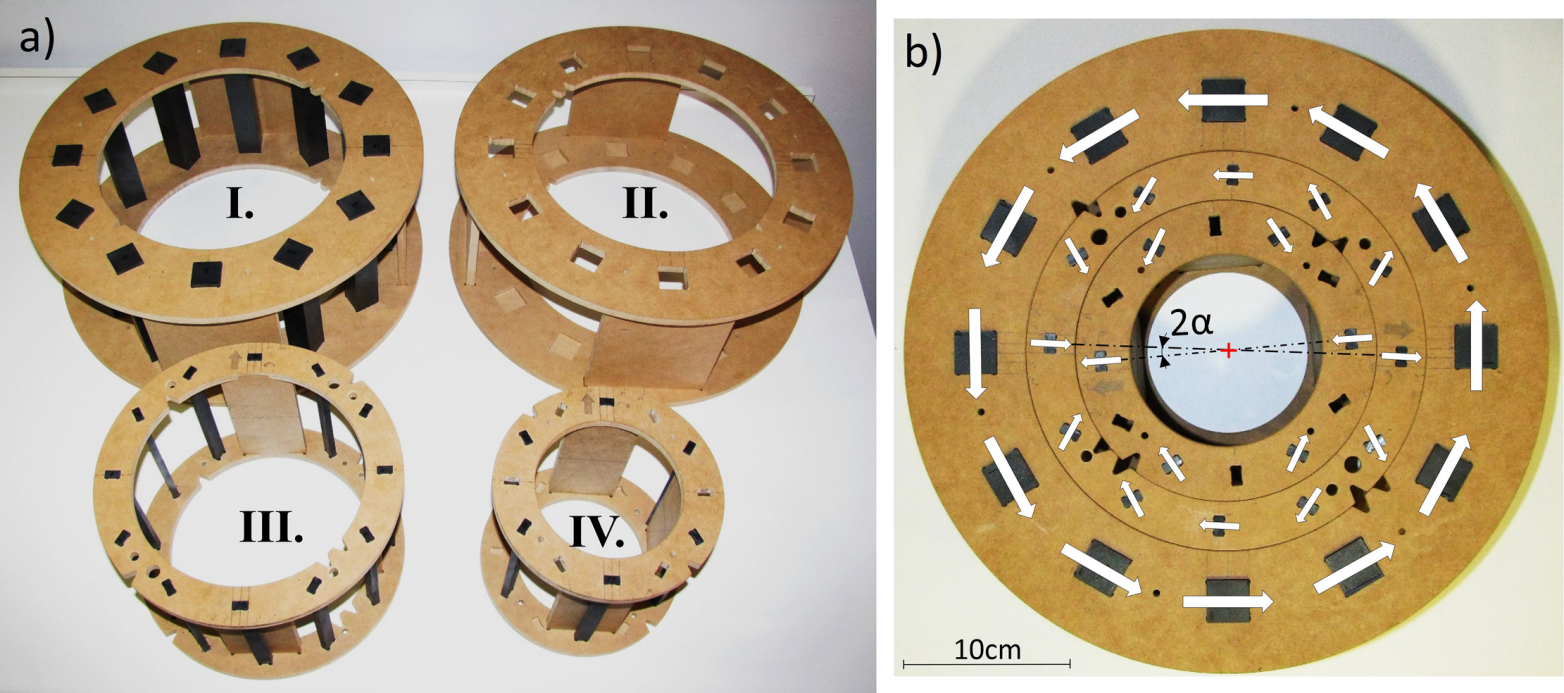
SPMA prototype. (a) Elements of the SPMA prototype shown separately: Array *D* with *Halbach* pattern (I) and *tangential* pattern (II), array *E* (III) array *F* (IV) each with *Halbach* pattern. Array *D* magnets are fitted in the MDF frame I or II to achieve **B**_**p**_ ON or **B**_**p**_ OFF configurations, respectively. (b) Arrays *E* (III) and *F* (IV) fitted inside array *D* with *tangential* pattern (II). **B**_**m**_ magnitude control is achieved by rotating arrays *E* (III) and *F* (IV) in opposite directions with prescribed angles α. The white arrows indicate the magnetisation direction of each magnet

A manually operated SPMA was built to demonstrate the generation, cancellation and regulation of **B**_**p**_ and **B**_**m**_. Like the SPMA for ULF relaxometry in the numerical study, the prototype consisted of three concentric arrays to generate the pre-polarisation field, **B**_**p**_ (array *D*), and the measurement field, **B**_**m**_ (array *E* and *F*), see Fig 6. The manually adjustable arrays were composed of ferrite permanent magnets of rectangular cross-section (ferrite grade Y30BH, B_r_ = 0.39 T, AMF Magnetics, Australia) chosen for their cost-effectiveness and availability and for simplicity of construction. Array *D* (Fig 6A, array I and II) had a radius *R*_*D*_ of 15 cm and comprises 12 magnets (15L x 2.5W x 2.54H cm) equally spaced around the circumference achieving a fill factor of 0.32. Twelve magnets (15L x 1.2W x 0.6H cm) are used in array *E* (R_E_ = 10.5 cm, Fig 6A, array III) and 6 magnets (15L x 1.2W x 0.6H cm) in array *F* (R_F_ = 7.5 cm, Fig 6A, array IV), leading to fill factors of 0.16 and 0.11, respectively. For each array, the magnets were pressure-fitted in pairs of medium density fibreboard (MDF) rings that hold the ends of the bar magnets. The moderate magnetisation of the ferrite magnets allowed safe manual rotations of the arrays. MDF rings of different diameter fitted within each other keeping the relative position of each array in place (Fig 6B). Two manually interchangeable frames for array *D* were built to hold the magnets for two different configurations: *tangential* (to generate **B**_**m**_) and *Halbach* (to generate **B**_**p**_); see Fig 6A, arrays I and II.

A Gaussmeter (F.W. Bell, Model 5080, Milwaukee, USA) mounted on a custom built 3-axis adjustable Cartesian holder was used to obtain magnetic field measurements in a grid (5 x 5 x 3 points in, *x*, *y*, *z*) covering the FOV (5 x 5 x 5 cm^3^) for 2 different angular settings (α = 0°, and 10°, see Fig 6B).

## Results

### I. SPMA for ULF relaxometry–Numerical simulation

#### Array A during pre-polarisation (‘switched on’)

**Fig 7:**
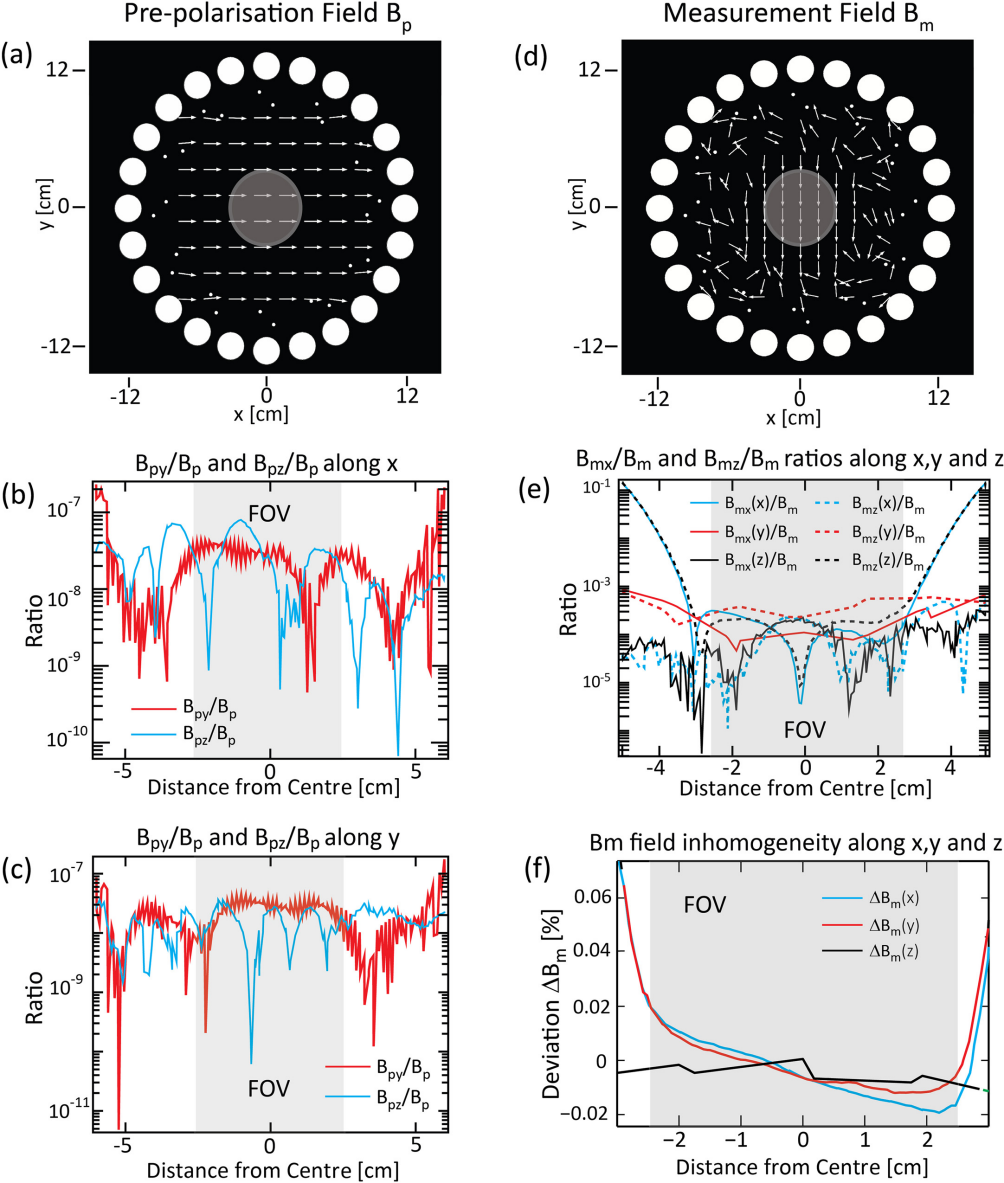
Magnitude and direction plots of magnetic flux density of a SPMA. Magnetic flux (a) during pre-polarisation (**B**_**p**_) and (d) measurement state (**B**_**m**_). Cross-sectional plots through the point of origin along the x-axis (b) and y-axis (c) of the ratio **B**_**py**_/**B**_**p**_ (red) and **B**_**pz**_/**B**_**p**_ (blue). **B**_**py**_ and **B**_**pz**_ are the y- and z-components of the pre-polarisation field **B**_**p**_ (= **B**_**px**_). **B**_**py**_ and **B**_**pz**_ are at least six orders of magnitude smaller in all directions within the FOV. Plots along the z-axis were omitted since all ratios are well below 10^−9^. (e) Cross-sections of **B**_**mx**_/**B**_**m**_ and **B**_**mz**_/**B**_**m**_ along the x-axis (blue), y-axis (red) and z-axis (black) demonstrating the x and z-component of the resultant magnetic field, generated by arrays *A*, *B* and *C*, are at least three orders of magnitude smaller than **B**_**m**_ (f). Percent deviation from measurement field magnitude of **B**_**m,**_ plotted along the x-axis (curve 1, blue), y-axis (curve 2, red) and z-axis (curve 3, black). Arrays *B* and *C* were rotated by ~4.5° to achieve a magnitude of 40 μT

The magnetic flux density distribution of Array *A* during pre-polarisation (**B**_**p**_) generated with the *Halbach* pattern (see Fig 3A), with magnet properties L = 70 cm, d_m_ = 2.16 cm, B_r_ = 1 T, is presented as *x*-*y* cross section plot at the centre of the array (z = 0) in Fig 7A. In the same figure, an arrow plot shows the local field direction of **B**_**p**_ parallel to the x-axis, and the FOV is visualised by light grey disks. In this simulation, arrays *B* and *C* are not included since **B**_**p**_ produced by array *A* is more than 1000 times larger than **B**_**m**_. The magnetic field distortion due to differences in electric permeability can be ignored because the relative permeability of the permanent magnets is very similar to that of the air (1.05 versus 1.00).

The directionality and homogeneity of **B**_**p**_ was assessed by the ratio of the minor field component (**B**_**py**_, **B**_**pz**_, as the y and z components of the **B**_**p**_ field, respectively) to the main component of **B**_**p**_ (along the *x*-axis). Fig 7B and 7C show cross-sectional plots of the magnetic flux density ratios **B**_**py**_/**B**_**p**_ (red) and **B**_**pz**_/**B**_**p**_ (blue) along *x* and *y*, demonstrating that **B**_**py**_ and **B**_**pz**_ were at least six orders of magnitude smaller than **B**_**p**_, in keeping with the high directionality of the latter along the *x*-axis. Plots along the z-axis are omitted since the ratios and the minor components are even smaller than **B**_**py**_ and **B**_**pz**_.

**Fig 8:**
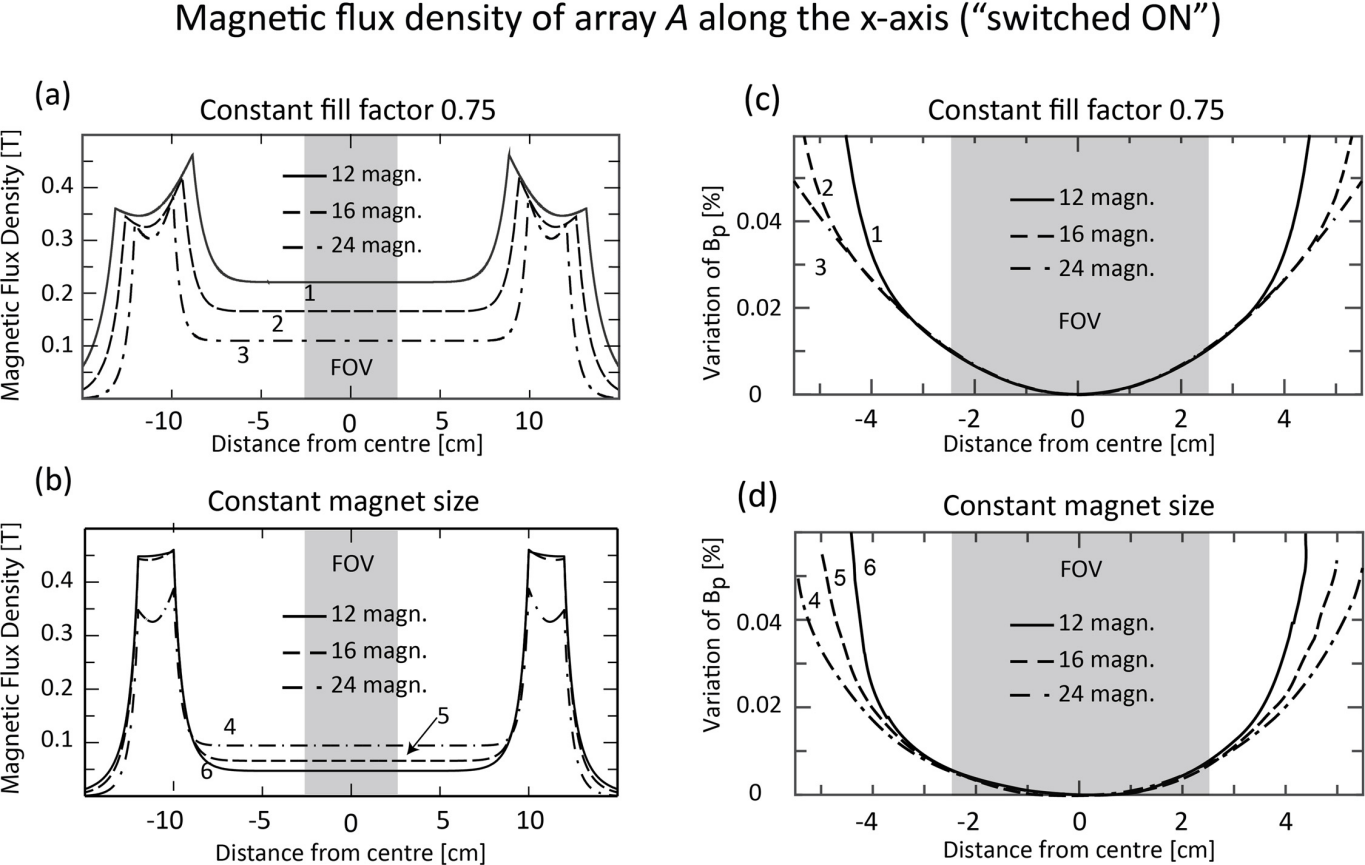
2D cross-sectional plots of pre-polarisation field B_p_ along the x-axis (switched on). For array *A* with constant fill factor, curve 1 (solid line) corresponds to 12 magnets, curve 2 (dashed line) to 16 and curve 3 (dash-dotted line) to 24 magnets. For array *A* with constant magnet dimensions (L = 70 cm, d_m_ = 2.16 cm), curve 4 (dash-dotted line) corresponds to 24 magnets, curve 5 (dashed line) to 16 magnets and curve 6 (solid line) to 12 magnets. (a) In array A with constant fill factor 0.75, the field strength within the field of view (FOV) decreases with the number of magnets, since magnet volume and surface area increase. (b) For array *A* with constant magnet size, the field strength decreases with decreasing numbers of magnets. (c) Within the FOV the field inhomogeneity decreases slightly with decreasing numbers of magnets for both constant fill factor (c) and magnet size (d). In all cases, the field inhomogeneity within the FOV was well below 0.02% (200 ppm)

**Fig 9:**
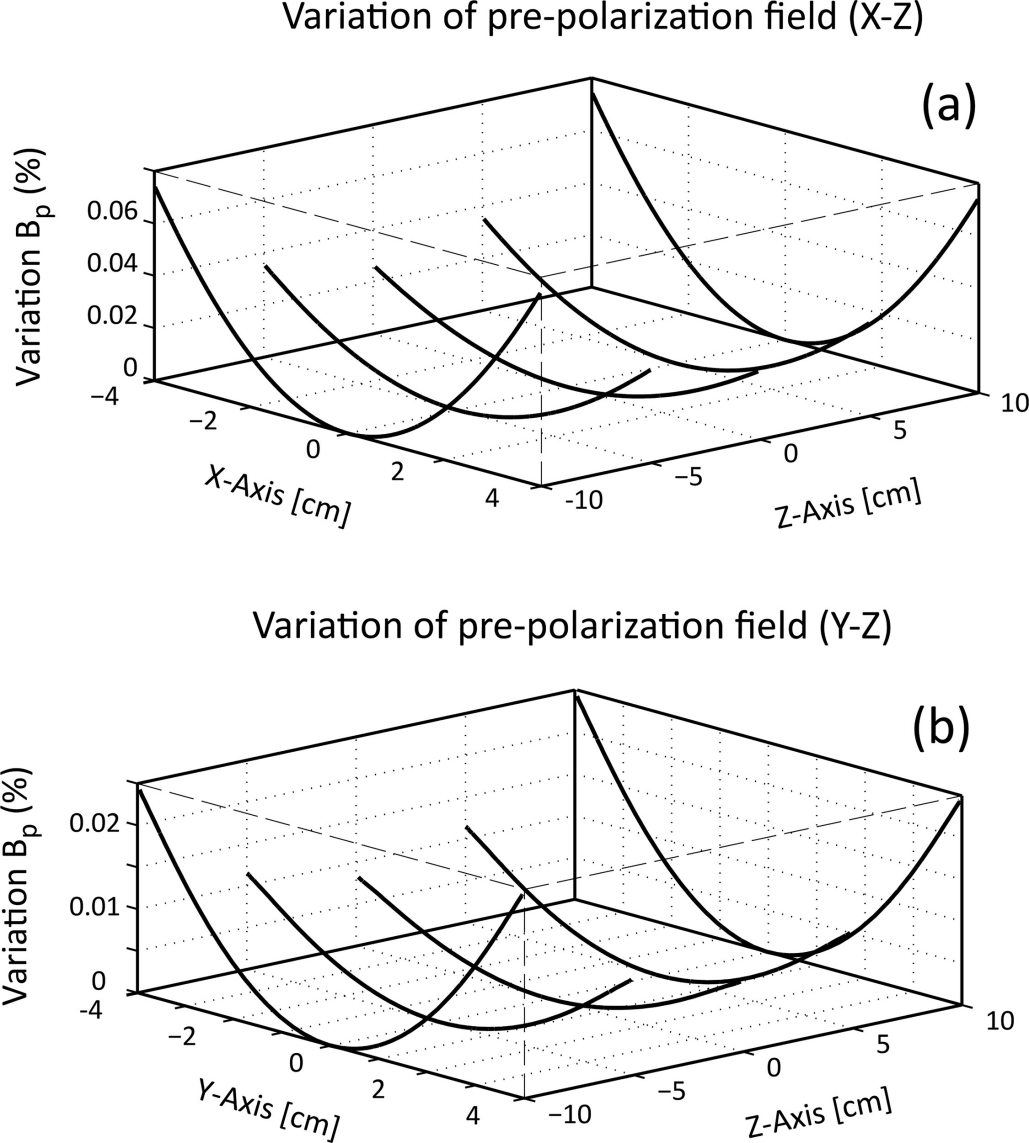
Relative magnitude variation of the pre-polarisation field, B_p_, generated by array *A* with 24 magnets. Field inhomogeneity shown as line plots in z = 2 cm steps along the x axis (a) and y axis (b). Plotted are percent magnitude deviations from the magnitude of **B**_**p**_ at the centre of the array. Within the chosen FOV of 5 x 5 x 5 cm^3^, the inhomogeneity is less than 0.02% in all cases

Twelve, 16 and 24 magnets with a fill factor of 0.75, 12 magnets with a fill factor of 0.375 and 16 magnets with a fill factor of 0.5 were analysed to study the effect of these parameters on the pre-polarisation field. The main results are summarized in Table 1 and the field magnitude along the x-axis is shown in Fig 8. The solid line plots correspond to 12 magnets, dashed line plots to 16 magnets and dash-dotted plots to 24 magnets. For a constant fill factor of 0.75, the achievable field strength increases with decreasing magnet number, as illustrated in Fig 8A, because of the greater magnet surface and volume, according to Eq 2. In contrast, by reducing the fill factor or the number of magnets, the pre-polarisation field strength decreased if magnet size was kept constant, as shown in Fig 8B. However, in all cases the magnetic field variation of **B**_**p**_ remained below 0.023% (230 ppm), as demonstrated in Fig 8C and 8D. The high field homogeneity is due to the combination of a small FOV compared to the large volume of the SPMA. Fig 9 illustrates the relative variation in magnitude of **B**_**p**_ in three dimensions for n = 24 magnets (fill factor 0.75) with respect to the magnitude at the centre plotted along the *z*-axis in 2 cm steps on the *x-z* (Fig 9A) and *y*-*z* planes (Fig 9B) demonstrating the high field homogeneity within the whole volume of the FOV.

**Table 1:** Achievable magnetic field strength at the centre of array A and field inhomogeneity within the field of view (FOV) during pre-polarisation for varying number of magnets and fill factors calculated with COMSOL.

Number of magnets	Fill factor	Centre Field strength [mT]	Field inhomogeneity within the FOV [%]
12	0.75	214.58	0.023
16	0.75	162.56	0.0175
24	0.75	109.15	0.011
16	0.5	76.29	0.008
12	0.375	54.51	0.006

#### Array A after pre-polarisation (‘switched off’)

**Fig 10:**
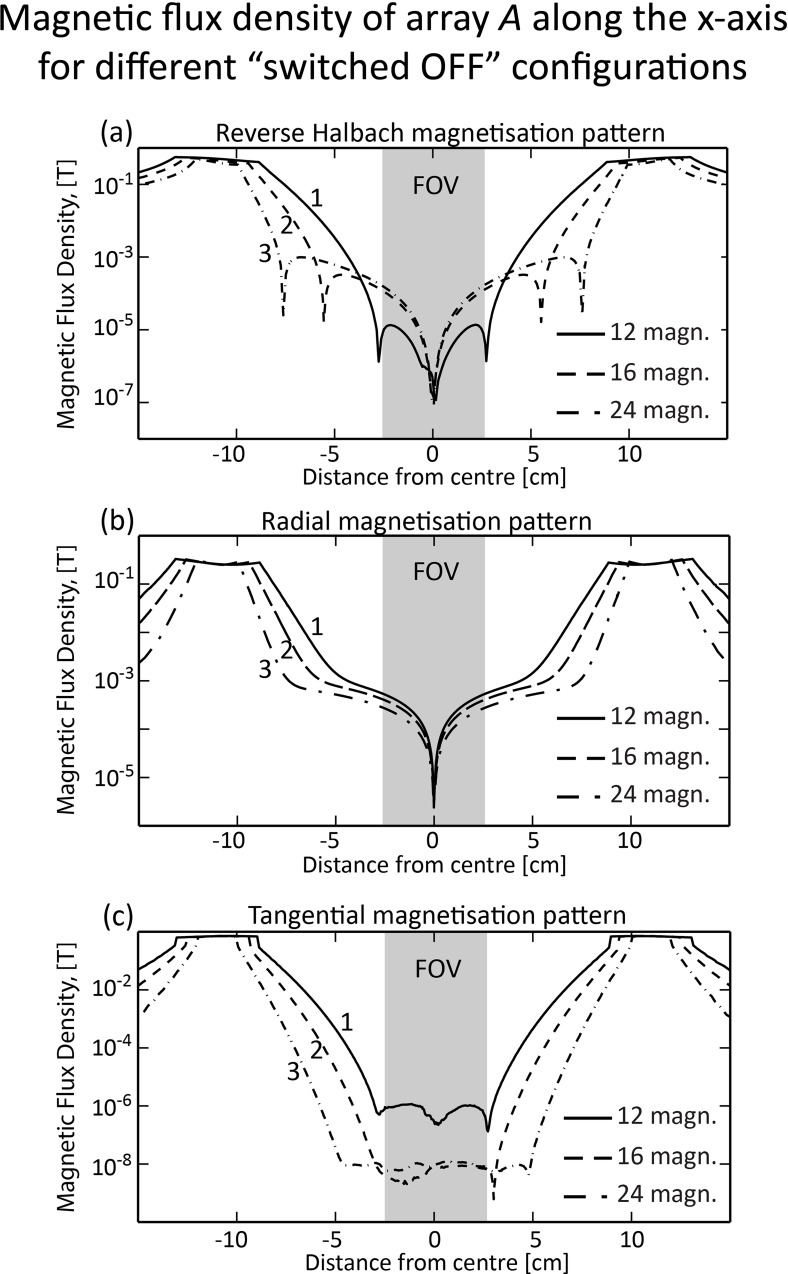
Magnetic flux density plots of array *A* along the x-axis for different magnetisation patterns, corresponding to Fig 4. In each array, 12 (solid line 1), 16 (dashed line 2) and 24 (dash-dotted line 3) magnets (L = 70 cm, d_m_ = 2.16 cm, B_r_ = 1 T, fill factor = 0.75) are considered for the (a) *reverse Halbach*, (b) *radial* and (c) *tangential* patterns. The FOV is indicated by the shaded area. Only the *tangential* pattern (c) is able to cancel the magnetic field within the FOV to magnitudes below 1 μT

During the measurement period, the magnetic field within the centre of the SPMA produced by array *A* must be minimised to enable precession and relaxation of the magnetisation vector of the sample. This is achieved by rotating each magnet of array *A* from the *Halbach* pattern (Fig 3A) to one of the three possible magnetisation patterns considered in this study, the *reverse Halbach* (Fig 3B), *tangential* (Fig 3C) or *radial* (Fig 3D) pattern. Fig 10 illustrates cross-sectional plots of the magnetic flux density generated by each pattern with the FOV indicated by the grey shaded area. Only the *tangential* pattern enabled field cancellation to magnitudes below 1 μT (fill factor = 0.75) within the FOV (Fig 10C). The residual magnetic field magnitude generated by array *A* is low enough (< 10^−8^ T for 16 or 24 magnets, see Fig 10C) not to interfere with the measurement field **B**_**m**_ (20–50 μT). Arrays *B* and *C* were excluded from the simulations in this section. The magnitude of the magnetic flux density generated by array *A* was below 1 μT in a larger area with a larger number of magnets (see Fig 10C). This is expected since the SPMA conforms better to an ideal Halbach array as the number of magnets increases. A similar effect was also observed during pre-polarisation with the *Halbach* pattern (Fig 8). From Fig 10C, it can be concluded that 16 (curve 2) or 24 cylindrical magnets (curve 3) are suitable to ensure minimum interference from array *A* during the measurement period.

The inaccuracy in the angular position of the permanent magnets of array *A* in the tangential configuration induces a field inhomogeneity of about 90μT/degree. This indicates that high precision actuators of less than 40 arcsecond tolerance would be required to keep the **B**_**p**_ field below 2μT.

#### Energy considerations for array A

Within the array, strong permanent magnets may generate considerable repulsive and attractive forces. We examined the total magnetic energy within array *A* as an index of how much energy was needed to change each permanent magnet in array *A* from pre-polarisation (‘switched on’) to measurement (‘switched off’) configurations. This parameter also relates to the mechanical stability of the array and may influence switching time. Since arrays *B* and *C* were of much lower volume and remanent magnetisation of the component magnets, their contribution was not considered.

Table 2 lists the total magnetic energy contained in array *A* for different magnetisation patterns and numbers of magnets. The total magnetic energy increased with as the number of magnets decreased due to the higher magnet volume and surface area. The highest total magnetic energy for a given number of magnets was achieved with the *radial* pattern and the lowest with the *tangential* pattern. With the *radial* pattern, each magnet experienced repelling forces only (magnetisation vector parallel) whereas only attracting forces were present with the *tangential* pattern (magnetisation vector anti-parallel). The latter is similar to two adjacent bar magnets which are allowed to rotate freely. Their opposite poles will attract each other (magnetisation vector anti-parallel) and form a stable configuration in the lowest total energy state.

**Table 2:** Total magnetic energy contained within array A for different magnetisation pattern and varying numbers of magnets.

	Total Magnetic Energy (Joule)			
Number of magnets n in Array A	Halbach	Reverse Halbach	Tangential	Radial
12	2216	2220	1270	3104
16	1683	1689	955	2377
24	1134	1139	638	1614

**Fig 11:**
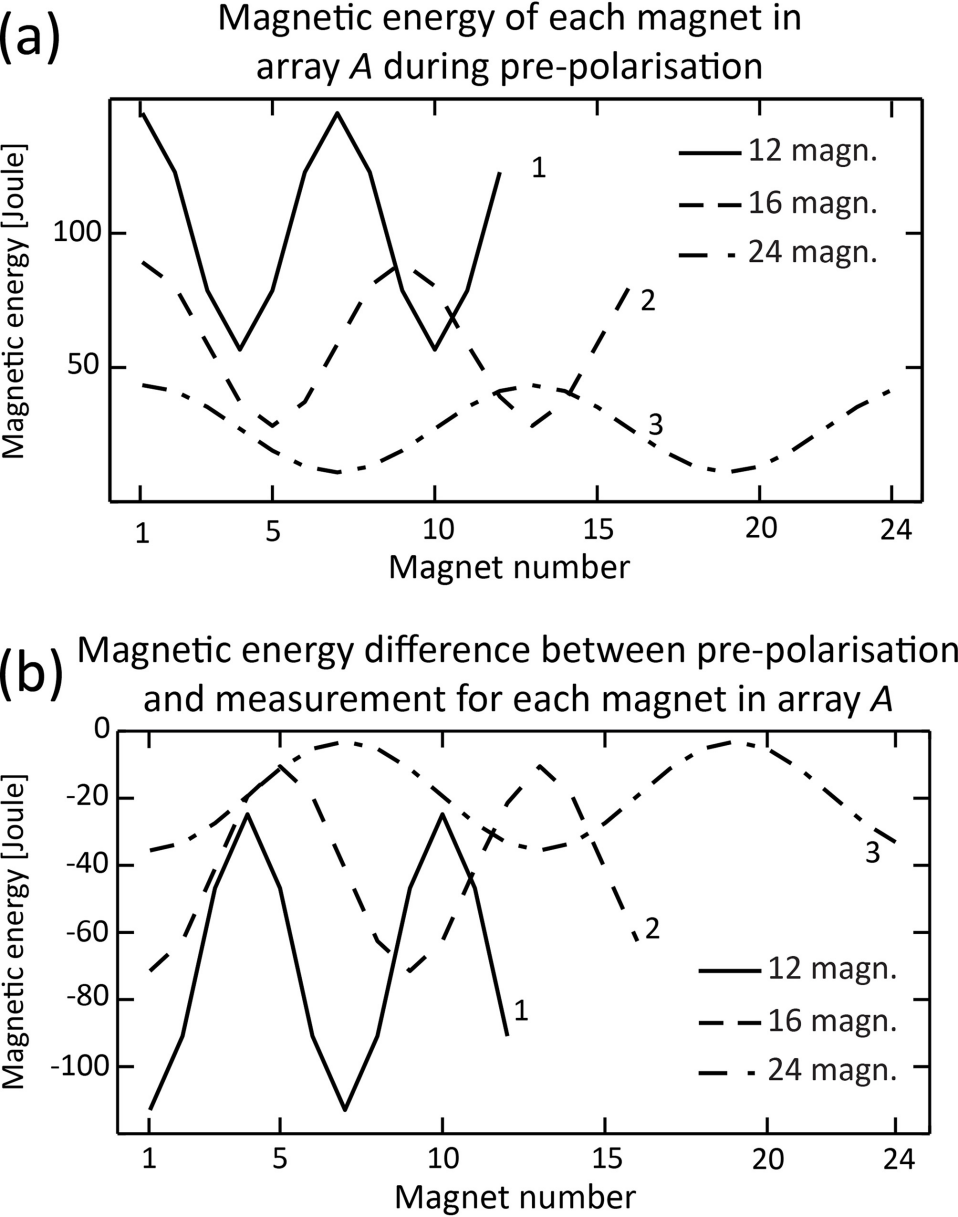
Total stored magnetic energy for each magnet in array *A* with different number of magnets. The solid line (curve 1) corresponds to 12 magnets, the dashed line (curve 2) to 16 magnets and the dash-dotted line (curve 3) to 24 magnets in array *A*. Magnet numbering follows Fig 2A. (a) Magnetic energy for each magnet in array *A* during pre-polarisation with Halbach magnetization pattern (see Fig 3A). (b) Magnetic energy difference between pre-polarisation and measurement with tangential magnetisation pattern. Negative values in Fig 11B indicate that all the magnets move to a lower magnetic energy state.

Due to the complex arrangement of magnetisation vectors in the *Halbach* pattern, each magnet has different stored total magnetic energy, as highlighted in Fig 11A for 12, 16 and 24 magnets. Consequently, the energy required to rotate each magnet varies when array *A* switches from the *Halbach* pattern (pre-polarisation field) to the *tangential* pattern (measurement state), which is illustrated in Fig 11B. As an example, the total magnetic energy difference for magnet number 7 of array *A* with 12 magnets (see Fig 2 for numbering) was -117 Joules (J) see Fig 11B, curve 1. The negative sign indicates energy release when magnet number 7 rotates from pre-polarisation to measurement states and +117 J is required to rotate it from the measurement to the pre-polarisation state. The findings favour rapid switching from the pre-polarisation to the measurement state because the magnets of array *A* tend naturally towards the tangential magnetisation pattern due to its lower total magnetic energy. Moreover, since the transition time from measurement to pre-polarisation is not a critical factor, it can be chosen to minimise mechanical vibrations, a potential source of measurement error.

The switching time from the pre-polarisation to the measurement state was estimated by assuming that the difference in magnetic energy is predominantly released as rotational energy. The rotational energy as a function of angular velocity (*ω*) is given by:

$$E_{rot}=\frac{1}{2}\omega^{2}I$$ (3)

where I = 1/2 *m_mag_r_m_*^2^ is the moment of inertia of the cylindrical magnet about the axis of rotation, and *m*_*mag*_ and *r*_*m*_ denote the mass and radius of the magnet, respectively.

The pre-polarisation field strength generated by array *A* with 24 magnets exceeded 100 mT, set as a target for our study (Fig 8A, Table 1). Hence, the study of magnetic forces in array A was performed for this configuration. The average total magnetic energy for array *A* was approximately +/- 20 J (curve 3, Fig 11B). With a magnet diameter *r*_*m*_ = 1.08 cm (refer to Eq 2, fill factor of 0.75) and using the average density of rare-earth magnets (7400 Kg/m^3^), the angular speed was estimated to be around 600 rad/sec i.e. 10 ms for one revolution (100 Hz). Therefore, in principle, rapid switching of the pre-polarisation field can be achieved using SPMAs.

The mechanical force required to rotate each magnet from the measurement state to the pre-polarisation state was calculated by relating torque (τ) to magnetic energy (E):

E=τθ (4)

with *θ* being the amount of rotation. Assuming that the force *F* was applied tangentially to the magnet (*τ* = *F∙r*_*m*_), the average mechanical force required was estimated to be ~300 N. Hence, as each magnet experiences about τ = 3.3 Nm, rotation of individual magnets could be achieved by commercially available rotary actuators [26]. Rapid switching in the presence of large forces requires careful control of magnet rotation and an engineering design that minimizes mechanical (torsional) vibrations both in individual magnets and in the entire array.

#### Arrays B and C (measurement array)

The measurement field **B**_**m**_ was generated by two concentric cylindrical arrays comprising 12 magnets, each with the *Halbach* pattern. The simulations accounted for the presence of array *A* in the tangential configuration. Fig 7D shows an arrow plot of the magnetic field in the *x*-*y* plane at *z* = 0 generated by array *A* with 24 magnets “switched off” i.e. in the *tangential* pattern (see Fig 3C) and by the *Halbach* pattern of arrays *B* and *C*. The local field direction of **B**_**m**_ in the FOV was in the y-coordinate direction. The measurement field **B**_**m**_ = 40 μT was generated by rotating arrays *B* and *C* in opposite directions, each by an angle of α = 4.5°. The directionality and homogeneity of **B**_**m**_ was evaluated by assessing the ratio of the minor field components (**B**_**mx**_, **B**_**mz**_ along *x*- and *z*) to the main component **B**_**m**_ (along the *y*-coordinate direction), plotted in Fig 7E, along the *x*- (solid line), *y*- (dash-dotted line) and *z*- (dashed line) axes. In all cases the minor components of **B**_**m**_ in the FOV were at least three orders of magnitude smaller than the main component.

Fig 7F shows detailed line plots of **B**_**m**_ along the *x*- (curve 1), *y*- (curve 2) and *z*-axes (curve 3) through the centre of the SPMA. Relative magnitude varied by less than 0.02% (200 ppm) within the FOV. The absolute magnetic field variation was around 8 nT (**B**_**m**_ = 40 μT) corresponding to a line broadening in the NMR spectrum due to this field inhomogeneity of less than 0.2 Hz, according to Eq 1.

For the chosen magnet characteristics (Ø = 0.3 cm, L = 70 cm and **B**_**r**_ = 0.2 T), rotation angles between 0 and 5° led to **B**_**m**_ values ranging between 0–50 μT. Precise rotations of arrays *B* and *C* are thus necessary to control the magnitude and direction of **B**_**m**_. A small mismatch of the rotation angles of arrays *B* and *C* could lead to a tilt from the defined axis of precession (*y*-axis) and the creation of an additional *x*-component in **B**_**m**_. This would result in a measurement field that is not perpendicular to sample magnetisation, resulting in a decrease in signal strength. Since the angles are relatively small, this effect is likely to be negligible. A misalignment of 0.5° of one array with the other arrays being correctly aligned resulted in a change in the magnitude of **B**_**m**_ from 55.4 μT to 52.6 μT corresponding to a proton Larmor frequency change of 118 Hz. Field inhomogeneity was increased from 0.02% (200 ppm) to around 0.05% (500 ppm) on average in all three directions. During an ULF experiment switching of **B**_**m**_ is not strictly necessary, since **B**_**p**_ is at least three orders of magnitude larger than **B**_**m**_. Therefore, arrays B and C can be carefully adjusted prior to experiments to minimize effects due to misalignment.

Magnetic fields of two arrays with different radii but the same number of identical magnets cannot be matched [23]. Hence, in arrays *B* and *C* field matching was achieved by reducing **B**_**r**_ from 0.2 T to 0.16 T for the magnets in array *C*. This is practical as rare earth magnets have a large range of standard remanent magnetisations, as detailed in MMPA 0100 –Standard Specifications for Permanent Magnet Materials.

### II. Manual SPMA measurement

**Fig 12:**
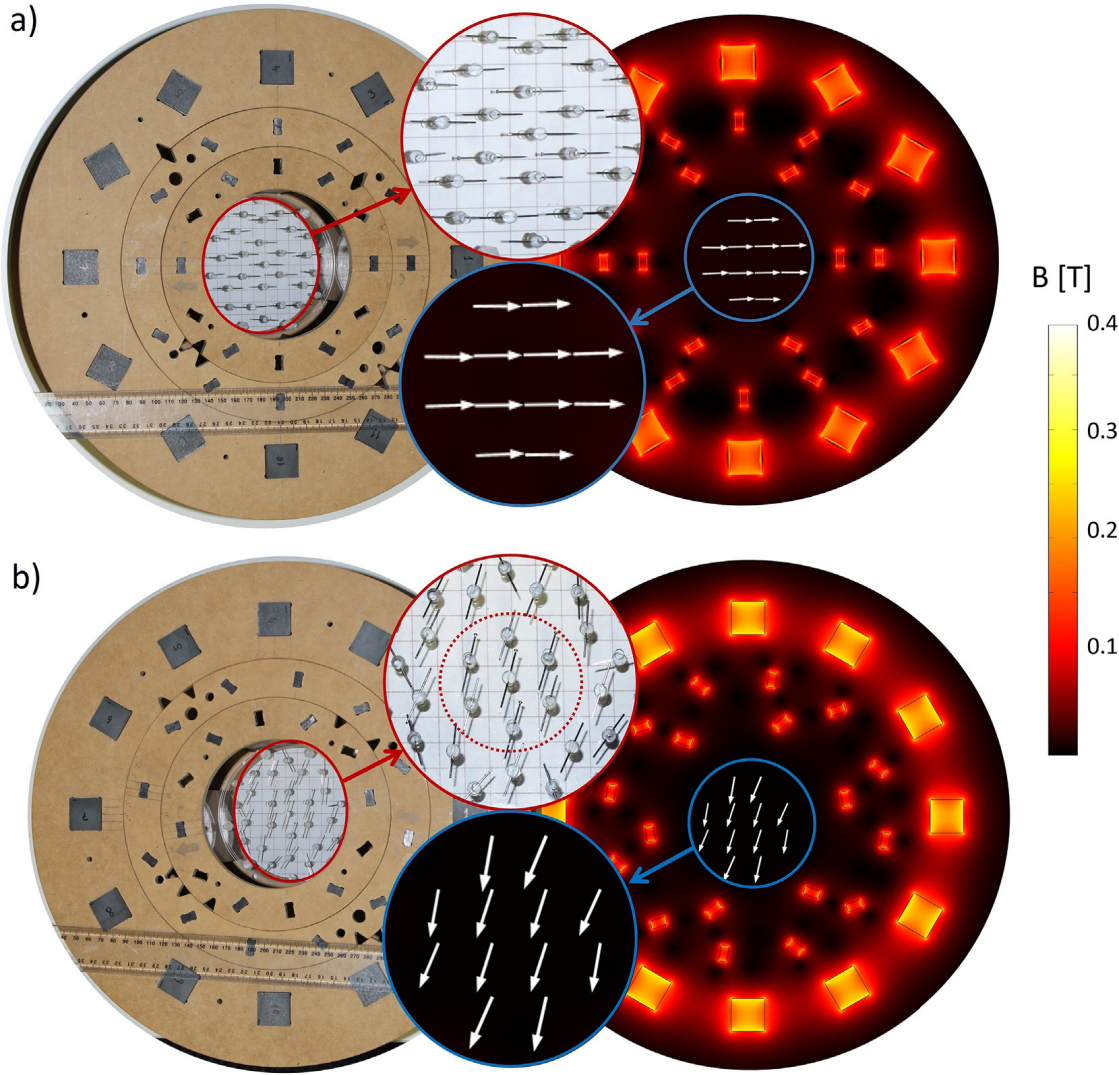
Comparison of B_p_ and B_m_ generated by a SPMA prototype (left hand side) with numerical simulation (right hand side). (a) Field direction of **B**_**p**_ indicated by array of needles (top inset, red circle) and surface plot of COMSOL (bottom inset, blue circle). (b) Field direction of **B**_**m**_ measured (top inset, red circle) and simulated (bottom inset, blue circle).

**B**_**p**_ and **B**_**m**_ in the manual SPMA prototype are presented for the pre-polarisation (Fig 12A) and the measurement state (Fig 12B). The direction of **B**_**p**_ and **B**_**m**_ within the FOV is qualitatively visualized by a custom-built array of small pivot-mounted needles. For comparison, a corresponding COMSOL model (see S2 File) was designed to simulate the magnetic fields within the FOV shown within the figure insets in blue circles. The visualised magnetic fields of the prototype are shown in the figure insets in red circles.

Table 3 summarizes the measured and calculated main field parameters for **B**_**p**_ and **B**_**m**_ generated by arrays *E* and *F* with rotation angles (α) of 0, 5 and 10°. A non-zero magnitude of **B**_**m**_ = 0.4 mT is present for α = 0° because the magnetic field generated by each array individually (**B**_**E**_ and **B**_**F**_) is not matched, hence the direction of **B**_**m**_ is not parallel to the y-axis. The dependence of **B**_**m**_ on the rotation angle *α* was correctly predicted by the simulations. However, a slight deviation was caused by the presence of a residual field generated by array *D* for the *tangential* pattern of 70 μT compared to the simulated 1 μT (Fig 6A, II). This may be due to uncompensated misalignment of individual magnets, variations in magnet dimensions or manufacturing imperfections which were not considered in the numerical model. Further optimization of the manual SPMA (for instance, with shimming [19, 25]) and detailed field homogeneity evaluation were not attempted since the primary purpose was to demonstrate the switching capabilities of **B**_**p**_ and the adjustability of **B**_**m**_. Low quality magnets in the prototype setup were also likely to have a strong effect on field homogeneity, as shown in Table 3. Measured field inhomogeneity was higher in all configurations than for simulated fields. Discrepancies in **B**_**m**_ were higher than in **B**_**p**_. Magnets producing **B**_**m**_ were closer to the FOV than magnets producing **B**_**p**_, resulting in greater sensitivity to variations the former. Additionally, the precision of the Gaussmeter (30μT + 1% of absolute scale) was lower for fields in the amplitude range of **B**_**m**_ than those in the range for **B**_**p**_.

**Table 3:** Comparison of simulated and measured magnetic fields generated by the SPMA prototype.

	Measured		Simulated	
Field	Field strength [mT]	Field inhomogeneity	Field strength [mT]	Field inhomogeneity
**B**_**p**_	13.2	1%	13.4	1%
**B**_**m**_ for α = +/- 0°	0.44	11%	0.42	3%
**B**_**m**_ for α = +/- 10°	1.30	10%	1.36	1%

**Fig 13:**
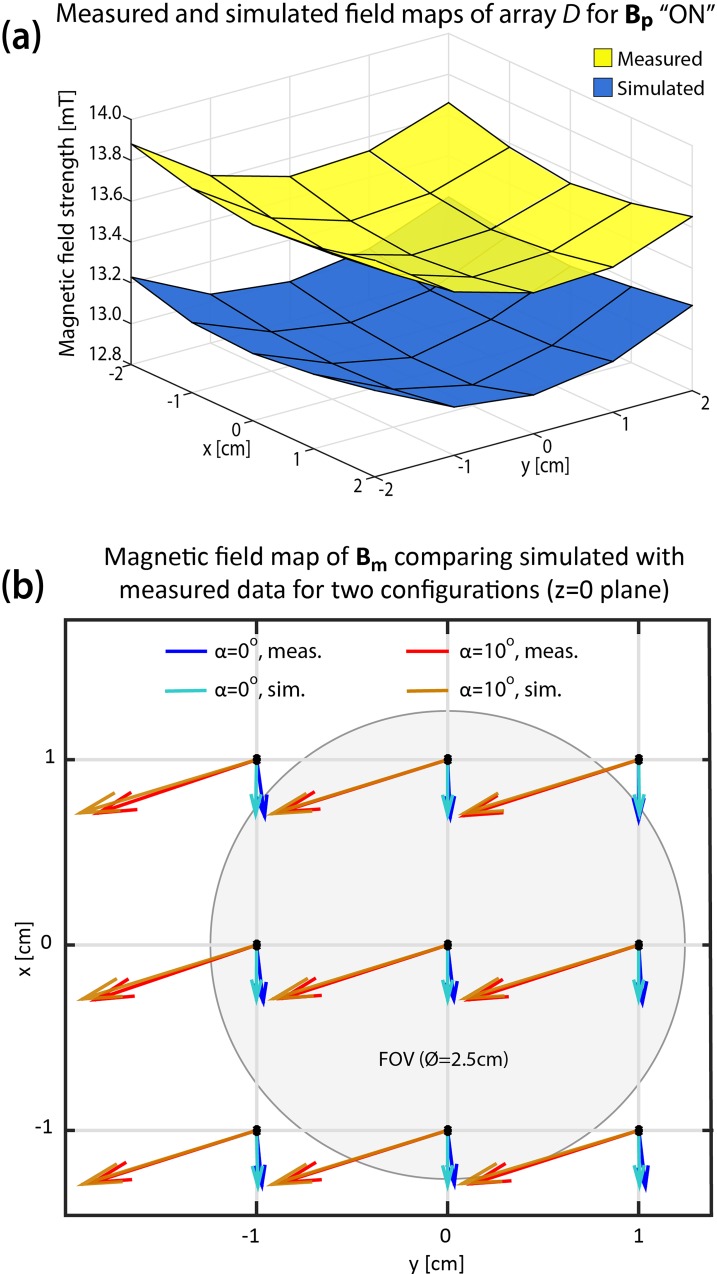
**Comparison of (a) B**_**p**_ **and (b) B**_**m**_ **generated by a SPMA prototype with results of numerical simulation.** (a) The measured magnitude of **B**_**p**_ (yellow) shows an offset of 200 μT compared to the simulated value (blue), due to a higher remanent magnetisation than specifications provided by the manufacturer. (b) Arrow plot of **B**_**m**_ for two angular positions of arrays *E* and *F*. Dashed lines represent the measured field and continuous lines represent the simulated field for α = 0^0^ and α = 10^0^. The mismatch between simulation and measurement is produced predominantly by the leftover field produced by array *D* in the tangential configuration.

The magnetic field maps of **B**_**p**_ and **B**_**m**_ generated by the manual SPMA prototype were obtained for pre-polarisation (Fig 13A) and measurement configurations (Fig 13B). Good qualitative agreement between simulation and measurement can be seen in Fig 13A. However, an offset of about 0.3 mT was present due to the higher remanent magnetisation of the magnets used compared to the manufacturer’s specifications which were used for the simulation. The measurements plotted in Fig 13B showed how **B**_**m**_ can be regulated in the setup. Here, the mismatch present between simulation and measurement is similar for both α = 0 and α = 10. This is mainly due to the leftover magnetic field produced by array *D* in the tangential configuration.

## Discussion

We introduced a small dynamic adjustable small permanent magnet array (SPMA) as a novel approach to generate multiple magnetic field configurations required for ULF NMR/MRI. As an advance on Halbach arrays, the SPMA enables magnetic fields to be generated through a combination of magnetisation patterns (see Fig 3) obtained by prescribed rotations of individual pivot-mounted permanent magnets and rotations of permanent magnets arrays. Two magnetisation patterns were implemented, the *Halbach* and *tangential* patterns, to generate and cancel the pre-polarisation field **B**_**p.**_ Two concentric arrays of permanent magnets were introduced to generate a variable measurement field **B**_**m**_ for ULF relaxometry at different frequencies. This would allow physical processes to be studied as a function of frequency.

Our simulation with 24 permanent magnets (L = 70 cm, d_m_ = 2.16 cm and B_r_ = 1 T) predicted pre-polarisation field magnitudes above 100 mT for the SPMA, higher than presently achieved in ULF instruments using resistive coil technology [13, 27, 28]. The simulation also predicted a magnetic field inhomogeneity of **B**_**p**_ less than 0.03% (300 ppm) within a field of FOV of 5 x 5 x 5 cm^3^. The SPMA field homogeneity is comparable to previously described Halbach array designs with stacked rectangular magnets for NMR [19, 29]. Higher and more homogeneous sample pre-polarisation should increase SNR [9, 10, 12].

Variable measurement fields ranging from near zero to 50 μT were generated by small rotations of two concentric cylindrical Halbach arrays *B* and *C* with the outer array *A* in the *tangential* pattern. Nominal magnitude deviations were below 0.02% or 200 ppm without shimming. This is equivalent to spectral line broadening of less than 0.2 Hz for proton Larmor frequencies at ULF. Broadening (which limits resolution) relates to absolute field inhomogeneity. Narrower spectral lines but with lower amplitude can be observed at ULF compared to high field NMR [13]. For example, 300 ppm inhomogeneity at **B**_**m**_ = 50 μT results in line width broadening equivalent to 0.015 ppm inhomogeneity at **B**_**m**_ = 1T.

Our findings highlight the importance of precise rotational adjustments both to achieve high field homogeneity and accurate control of **B**_**m**_. **B**_**m**_ does not need to be switched off during an experiment since its magnitude is at least three orders of magnitude lower than **B**_**p**_. This simplifies the design of motion controllers and facilitates adjustments to enhance field homogeneity. Switching off **B**_**p**_ rapidly is crucial and can be achieved by the use of high-quality actuators, such as the SHA25A-81 (Harmonic Drive, Massachusetts, USA).

Our model predicted that fast switching (within 6 ms) from the pre-polarisation to the measurement state would be possible given the total magnetic energy difference between the *Halbach* pattern and the *tangential* pattern. The rapid switching time is comparable to current ULF instruments using resistive coils and customized switch boxes [9, 12]. It is plausible that even faster switching can be achieved through the use of additional hydraulic or pneumatic actuator systems. However, mechanical vibration may need to be considered and was beyond the scope of this study.

Unlike resistive coils, energy is not dissipated into heat due to current flow, obviating sample heating. Furthermore, undesired signal generation due to transient currents, induced in conductors by rapid switching, is reduced because the conductivity of magnet alloys is much lower than for conductors like copper used in coils. Compared to superconducting magnets, the energy consumption of the SPMA is significantly lower because cooling is not required, further reducing cost.

A manual SPMA was built and compared with predictions from simulations. It demonstrated the ability to generate varying magnetic fields and the validity of the numerical approach. All the main field parameters were correctly described with our computational model and experimentally verified. This is in agreement with previous studies in which COMSOL was used to simulate magnetic field parameters to optimise the design of instruments based on Halbach arrays [20, 25]. Further optimization to maximize field homogeneity and to match the magnetic fields generated by array B and C was not attempted here since the primary focus was to verify the new features of the SPMA. However, shimming techniques are avenues for future research to optimise the performance of the SPMA [19, 25].

Although this study focuses on the application of the SPMA for of ULF NMR relaxometry, the flexible and modular design of the SPMA allows additional magnet arrays to be added, for instance, to generate the dynamic gradient fields required for imaging with ULF-MRI. This is achievable since the absolute field homogeneity requirements for ULF instrumentation are quite moderate. Notably, other than the switching between **B**_**p**_ and **B**_**m**_ with the SPMA, sequences for signal generation and acquisition are similar to conventional approaches with instrumentation using resistive magnets. Measurement strategies like iterative sample pre-polarisation to enhance signal-to-noise ratio through signal averaging can be applied with the SPMA similar to high field relaxometry applications.

Generating linear gradient fields with permanent magnets is more challenging at ULF due to the presence of concomitant fields [30, 31]. These are predicted by Maxwell’s equations since the field gradients are comparable in magnitude to **B**_**m**_ [13] and result in image distortion that requires correction during reconstruction. New approaches for spatial signal encoding that utilize intrinsic field inhomogeneity in combination with non-linear image reconstruction methods have been described for static Halbach arrays, allowing spatial encoding in 1D [25]. Rotation of the array is required for 2D images and the use of additional excitation hardware has been proposed for spatial encoding in the third dimension, using Bloch-Siebert Spatial Encoding (BS-SET) or TRansient Array Spatial Encoding (TRASE) [25]. We posit that 3D spatial encoding can be achieved with additional dynamically adjustable SPMAs [32]. However, in ULF-MRI slice selection remains an open question and applications needing to acquire a single slice are currently faced with having to acquire the entire 3D image. Furthermore, slice selection may require very long duration excitation pulses at ULF. The idea that the high homogeneity of **B**_**p**_ produced by the SPMA may be harnessed to create fields for slice selection requires further exploration.

## Conclusions

The proposed SPMA is substantially different from all the resistive coil-based approaches reported in the literature to date. Flexible manipulation of the magnetic field while achieving very high field homogeneities through rearrangement of a concentric SPMA was empirically demonstrated. Pre-polarisation and measurement fields relevant for ULF NMR could be generated by a combination of rotation of individual permanent magnets and rotation of Halbach arrays. Our findings may benefit future developments in ULF-NMR/MRI by eliminating the need for resistive coils, increasing the potential for compact, low energy, portable instrumentation.

## Supporting Information

S1 File SPMA Model Report. Model documentation generated by COMSOL with implemented parameters for the SPMA.
(ZIP)

S2 File Prototype Model Report. Model documentation generated by COMSOL with implemented parameters for the manual prototype.
(ZIP)
