# Supplementary material for: Rotatable Small Permanent Magnet Array for Ultra-Low Field Nuclear Magnetic Resonance Instrumentation: A Concept Study
Source: PLoS One. 2016 Jun 6;11(6):e0157040. doi: 10.1371/journal.pone.0157040 (PMC4894570; doi:10.1371/journal.pone.0157040)
Supplement: S1 File — Model documentation generated by COMSOL with implemented parameters for the SPMA. (ZIP) [file pone.0157040.s001.zip › HalBachArray24CylindricalMag.html]

xml version="1.0" encoding="utf-8"?


HalBachArray24CylindricalMag


Halbach3D 24 FF075PLosONE

|  |  |
| --- | --- |
| Date | Mar 1, 2016 12:30:31 PM |

Contents

|  |  |  |  |  |  |  |
| --- | --- | --- | --- | --- | --- | --- |
| 1. |  | Global | | | | |
| 1.1. |  |  | Definitions | | | |
| 2. |  | Component 1 | | | | |
| 2.1. |  |  | Definitions | | | |
| 2.2. |  |  | Geometry 1 | | | |
| 2.3. |  |  | Materials | | | |
| 2.4. |  |  | Magnetic Fields, No Currents | | | |
| 2.5. |  |  | Mesh 1 | | | |
| 3. |  | Component 2 | | | | |
| 3.1. |  |  | Definitions | | | |
| 3.2. |  |  | Geometry 2 | | | |
| 4. |  | Study 1 | | | | |
| 4.1. |  |  | Stationary | | | |
| 4.2. |  |  | Solver Configurations | | | |
| 5. |  | Results | | | | |
| 5.1. |  |  | Data Sets | | | |
| 5.2. |  |  | Tables | | | |
| 5.3. |  |  | Plot Groups | | | |

## 1. Global

|  |  |
| --- | --- |
| Date | Mar 1, 2016 9:51:04 AM |

Global settings

| Name | Halbach3D\_24\_FF075PLosONE.mph |
| Path | R:\projects\MVogel\_ULF\_Halbach\COMSOL Models Halbach\3D\Halbach3D\_24\_FF075PLosONE.mph |
| Program | COMSOL 5.0 (Build: 244) |
| Unit system | SI |

Used products

| COMSOL Multiphysics |
| AC/DC Module |

### 1.1. Definitions

#### 1.1.1. Parameters 1

Parameters

| Name | Expression | Value | Description |
| BpRem | 1[T] | 1.0000 T |  |
| BpState | 1 | 1.0000 | 1 = ON, -1 = OFF |
| halbach | 1 | 1.0000 | 1 = Yes 0 = NO |
| argBp | BpState\*(1 + halbach)\*pi/12 | 0.52360 |  |
| tickn | 0 | 0.0000 | 1 = Tangential, 0 = Radial |
| tick | tickn\*pi/2 | 0.0000 | pi/2 = tang, 0= radial |
| dm | 0.0216[m] | 0.021600 m |  |
| length | 0.7[m] | 0.70000 m |  |
| RA | 0.11[m] | 0.11000 m |  |
| FOV | 0.05[m] | 0.050000 m |  |

## 2. Component 1

|  |  |
| --- | --- |
| Date | Mar 1, 2016 9:51:04 AM |

Component settings

| Unit system | SI |
| Geometry shape order | automatic |

### 2.1. Definitions

#### 2.1.1. Coordinate Systems

##### Boundary System 1

|  |  |
| --- | --- |
| Coordinate system type | Boundary system |
| Tag | sys1 |

Settings

| First (t1) | Second (t2) | Third (n) |
| t1 | t2 | n |

Settings

| Name | Value |
| Create first tangent direction from | Global Cartesian |

### 2.2. Geometry 1

Geometry 1

Units

| Length unit | m |
| Angular unit | deg |

Geometry statistics

| Property | Value |
| Space dimension | 3 |
| Number of domains | 26 |
| Number of boundaries | 158 |
| Number of edges | 312 |
| Number of vertices | 206 |

#### 2.2.1. Cylinder 1 (Cyl1)

Position

| Name | Value |
| Position | {RA, 0, -length/2} |

Axis

| Name | Value |
| Axis type | z - axis |

Size and shape

| Name | Value |
| Radius | dm/2 |
| Height | length |

#### 2.2.2. Rotate 1 (Rot1)

Selections of resulting entities

| Name | Value |
| Keep input objects | On |
| Rotation | {15, 30, 45, 60, 75, 90, 105, 120, 135, 150, 165, 180, 195, 210, 225, 240, 255, 270, 285, 300, 315, 330, 345} |
| Point on axis of rotation | {0, 0, 0} |
| Axis type | z - axis |

#### 2.2.3. Cylinder 2 (Cyl2)

Position

| Name | Value |
| Position | {0, 0, -0.75} |

Axis

| Name | Value |
| Axis type | z - axis |

Size and shape

| Name | Value |
| Radius | 0.4 |
| Height | 1.5 |

#### 2.2.4. Sphere 1 (Sph1)

Position

| Name | Value |
| Position | {0, 0, 0} |

Axis

| Name | Value |
| Axis type | z - axis |

Size

| Name | Value |
| Radius | FOV/2 |

### 2.3. Materials

#### 2.3.1. Air

Air

Selection

| Geometric entity level | Domain |
| Selection | Domains 1, 13 |

Basic Settings

| Description | Value |
| Heat capacity at constant pressure | Cp(T[1/K])[J/(kg\*K)] |
| Speed of sound | cs(T[1/K])[m/s] |
| Dynamic viscosity | eta(T[1/K])[Pa\*s] |
| Ratio of specific heats | 1.4 |
| Thermal conductivity | {{k(T[1/K])[W/(m\*K)], 0, 0}, {0, k(T[1/K])[W/(m\*K)], 0}, {0, 0, k(T[1/K])[W/(m\*K)]}} |
| nu0 | nu0(T[1/K])[m^2/s] |
| Density | rho(pA[1/Pa], T[1/K])[kg/m^3] |
| Electrical conductivity | {{0[S/m], 0, 0}, {0, 0[S/m], 0}, {0, 0, 0[S/m]}} |

Functions

| Function name | Type |
| Cp | Piecewise |
| cs | Analytic |
| eta | Piecewise |
| k | Piecewise |
| nu0 | Piecewise |
| rho | Analytic |

Cp

cs

eta

k

nu0

#### 2.3.2. Soft Iron (Without Losses)

Soft Iron (without losses)

Selection

| Geometric entity level | Domain |
| Selection | Domains 2–12, 14–26 |

Material parameters

| Name | Value | Unit |
| Relative permeability | 1.05 | 1 |

Basic Settings

| Description | Value |
| Electrical conductivity | {{0.666e6[S/m], 0, 0}, {0, 0.666e6[S/m], 0}, {0, 0, 0.666e6[S/m]}} |
| Relative permittivity | {{1.05, 0, 0}, {0, 1.05, 0}, {0, 0, 1.05}} |
| Relative permeability | {{1.05, 0, 0}, {0, 1.05, 0}, {0, 0, 1.05}} |

BH curve Settings

| Description | Value |
| Magnetic flux density norm | BH(normH[m/A])[T] |
| normH | sqrt(H1^2 + H2^2 + H3^2) |

Functions

| Function name | Type |
| BH | Interpolation |

BH

HB curve Settings

| Description | Value |
| Magnetic field norm | HB(normB[1/T])[A/m] |
| normB | sqrt(B1^2 + B2^2 + B3^2) |

Functions

| Function name | Type |
| HB | Interpolation |

HB

### 2.4. Magnetic Fields, No Currents

Magnetic Fields, No Currents

Selection

| Geometric entity level | Domain |
| Selection | Domains 1–26 |

Equations

Settings

| Description | Value |
| Magnetic scalar potential | Quadratic |
| Value type when using splitting of complex variables | Complex |

Used products

| COMSOL Multiphysics |
| AC/DC Module |

Variables

| Name | Expression | Unit | Description | Selection |
| emnc.Bbx | 0 | T | Background magnetic flux density, x component | Domains 1–26 |
| emnc.Bby | 0 | T | Background magnetic flux density, y component | Domains 1–26 |
| emnc.Bbz | 0 | T | Background magnetic flux density, z component | Domains 1–26 |
| emnc.Hbx | 0 | A/m | Background magnetic field, x component | Domains 1–26 |
| emnc.Hby | 0 | A/m | Background magnetic field, y component | Domains 1–26 |
| emnc.Hbz | 0 | A/m | Background magnetic field, z component | Domains 1–26 |
| emnc.nx | nx |  | Normal vector, x component | Boundaries 5–82, 84–91, 93–158 |
| emnc.ny | ny |  | Normal vector, y component | Boundaries 5–82, 84–91, 93–158 |
| emnc.nz | nz |  | Normal vector, z component | Boundaries 5–82, 84–91, 93–158 |
| emnc.nx | dnx |  | Normal vector, x component | Boundaries 1–4, 83, 92 |
| emnc.ny | dny |  | Normal vector, y component | Boundaries 1–4, 83, 92 |
| emnc.nz | dnz |  | Normal vector, z component | Boundaries 1–4, 83, 92 |
| emnc.nmeshx | nxmesh |  | Mesh normal vector, x component | Boundaries 5–82, 84–91, 93–158 |
| emnc.nmeshy | nymesh |  | Mesh normal vector, y component | Boundaries 5–82, 84–91, 93–158 |
| emnc.nmeshz | nzmesh |  | Mesh normal vector, z component | Boundaries 5–82, 84–91, 93–158 |
| emnc.nmeshx | dnxmesh |  | Mesh normal vector, x component | Boundaries 1–4, 83, 92 |
| emnc.nmeshy | dnymesh |  | Mesh normal vector, y component | Boundaries 1–4, 83, 92 |
| emnc.nmeshz | dnzmesh |  | Mesh normal vector, z component | Boundaries 1–4, 83, 92 |
| emnc.unmeshx | unxmesh |  | Mesh normal vector, upside, x component | Boundaries 5–82, 84–91, 93–158 |
| emnc.unmeshy | unymesh |  | Mesh normal vector, upside, y component | Boundaries 5–82, 84–91, 93–158 |
| emnc.unmeshz | unzmesh |  | Mesh normal vector, upside, z component | Boundaries 5–82, 84–91, 93–158 |
| emnc.dnmeshx | dnxmesh |  | Mesh normal vector, downside, x component | Boundaries 5–82, 84–91, 93–158 |
| emnc.dnmeshy | dnymesh |  | Mesh normal vector, downside, y component | Boundaries 5–82, 84–91, 93–158 |
| emnc.dnmeshz | dnzmesh |  | Mesh normal vector, downside, z component | Boundaries 5–82, 84–91, 93–158 |
| emnc.intWm | emnc.int\_Wm(emnc.d\*emnc.dWm) | J | Total magnetic energy | Global |
| emnc.d | 1 | 1 | Contribution | Domains 1–26 |
| emnc.unTmx | 0 | Pa | Maxwell upward magnetic surface stress tensor, x component | Boundaries 1–158 |
| emnc.unTmy | 0 | Pa | Maxwell upward magnetic surface stress tensor, y component | Boundaries 1–158 |
| emnc.unTmz | 0 | Pa | Maxwell upward magnetic surface stress tensor, z component | Boundaries 1–158 |
| emnc.dnTmx | 0 | Pa | Maxwell downward magnetic surface stress tensor, x component | Boundaries 1–158 |
| emnc.dnTmy | 0 | Pa | Maxwell downward magnetic surface stress tensor, y component | Boundaries 1–158 |
| emnc.dnTmz | 0 | Pa | Maxwell downward magnetic surface stress tensor, z component | Boundaries 1–158 |

#### 2.4.1. Magnetic Flux Conservation 1

Magnetic Flux Conservation 1

Selection

| Geometric entity level | Domain |
| Selection | Domains 1, 13 |

Equations

Settings

| Description | Value |
| Constitutive relation | Remanent flux density |
| Remanent flux density, x component | 0 |
| Remanent flux density, y component | 0 |
| Remanent flux density, z component | 0 |
| Relative permeability | User defined |
| Relative permeability | {{1.1, 0, 0}, {0, 1.1, 0}, {0, 0, 1.1}} |
| Magnetic flux density norm | User defined |

##### Variables

| Name | Expression | Unit | Description | Selection |
| --- | --- | --- | --- | --- |
| emnc.unTmx | -0.5\*emnc.dnx\*(real(up(emnc.Bx))\*real(up(emnc.Hx))+real(up(emnc.By))\*real(up(emnc.Hy))+real(up(emnc.Bz))\*real(up(emnc.Hz)))+real(up(emnc.Bx))\*(real(up(emnc.Hx))\*emnc.dnx+real(up(emnc.Hy))\*emnc.dny+real(up(emnc.Hz))\*emnc.dnz) | Pa | Maxwell upward magnetic surface stress tensor, x component | Boundaries 71–74, 86–89 |
| emnc.unTmy | -0.5\*emnc.dny\*(real(up(emnc.Bx))\*real(up(emnc.Hx))+real(up(emnc.By))\*real(up(emnc.Hy))+real(up(emnc.Bz))\*real(up(emnc.Hz)))+real(up(emnc.By))\*(real(up(emnc.Hx))\*emnc.dnx+real(up(emnc.Hy))\*emnc.dny+real(up(emnc.Hz))\*emnc.dnz) | Pa | Maxwell upward magnetic surface stress tensor, y component | Boundaries 71–74, 86–89 |
| emnc.unTmz | -0.5\*emnc.dnz\*(real(up(emnc.Bx))\*real(up(emnc.Hx))+real(up(emnc.By))\*real(up(emnc.Hy))+real(up(emnc.Bz))\*real(up(emnc.Hz)))+real(up(emnc.Bz))\*(real(up(emnc.Hx))\*emnc.dnx+real(up(emnc.Hy))\*emnc.dny+real(up(emnc.Hz))\*emnc.dnz) | Pa | Maxwell upward magnetic surface stress tensor, z component | Boundaries 71–74, 86–89 |
| emnc.unTmx | -0.5\*emnc.dnx\*(real(up(emnc.Bx))\*real(up(emnc.Hx))+real(up(emnc.By))\*real(up(emnc.Hy))+real(up(emnc.Bz))\*real(up(emnc.Hz)))+real(up(emnc.Bx))\*(real(up(emnc.Hx))\*emnc.dnx+real(up(emnc.Hy))\*emnc.dny+real(up(emnc.Hz))\*emnc.dnz) | Pa | Maxwell upward magnetic surface stress tensor, x component | Boundaries 5–70, 75–82, 84–85, 90–91, 93–158 |
| emnc.unTmy | -0.5\*emnc.dny\*(real(up(emnc.Bx))\*real(up(emnc.Hx))+real(up(emnc.By))\*real(up(emnc.Hy))+real(up(emnc.Bz))\*real(up(emnc.Hz)))+real(up(emnc.By))\*(real(up(emnc.Hx))\*emnc.dnx+real(up(emnc.Hy))\*emnc.dny+real(up(emnc.Hz))\*emnc.dnz) | Pa | Maxwell upward magnetic surface stress tensor, y component | Boundaries 5–70, 75–82, 84–85, 90–91, 93–158 |
| emnc.unTmz | -0.5\*emnc.dnz\*(real(up(emnc.Bx))\*real(up(emnc.Hx))+real(up(emnc.By))\*real(up(emnc.Hy))+real(up(emnc.Bz))\*real(up(emnc.Hz)))+real(up(emnc.Bz))\*(real(up(emnc.Hx))\*emnc.dnx+real(up(emnc.Hy))\*emnc.dny+real(up(emnc.Hz))\*emnc.dnz) | Pa | Maxwell upward magnetic surface stress tensor, z component | Boundaries 5–70, 75–82, 84–85, 90–91, 93–158 |
| emnc.dnTmx | -0.5\*emnc.unx\*(real(down(emnc.Bx))\*real(down(emnc.Hx))+real(down(emnc.By))\*real(down(emnc.Hy))+real(down(emnc.Bz))\*real(down(emnc.Hz)))+real(down(emnc.Bx))\*(real(down(emnc.Hx))\*emnc.unx+real(down(emnc.Hy))\*emnc.uny+real(down(emnc.Hz))\*emnc.unz) | Pa | Maxwell downward magnetic surface stress tensor, x component | Boundaries 71–74, 86–89 |
| emnc.dnTmy | -0.5\*emnc.uny\*(real(down(emnc.Bx))\*real(down(emnc.Hx))+real(down(emnc.By))\*real(down(emnc.Hy))+real(down(emnc.Bz))\*real(down(emnc.Hz)))+real(down(emnc.By))\*(real(down(emnc.Hx))\*emnc.unx+real(down(emnc.Hy))\*emnc.uny+real(down(emnc.Hz))\*emnc.unz) | Pa | Maxwell downward magnetic surface stress tensor, y component | Boundaries 71–74, 86–89 |
| emnc.dnTmz | -0.5\*emnc.unz\*(real(down(emnc.Bx))\*real(down(emnc.Hx))+real(down(emnc.By))\*real(down(emnc.Hy))+real(down(emnc.Bz))\*real(down(emnc.Hz)))+real(down(emnc.Bz))\*(real(down(emnc.Hx))\*emnc.unx+real(down(emnc.Hy))\*emnc.uny+real(down(emnc.Hz))\*emnc.unz) | Pa | Maxwell downward magnetic surface stress tensor, z component | Boundaries 71–74, 86–89 |
| emnc.dnTmx | -0.5\*emnc.unx\*(real(down(emnc.Bx))\*real(down(emnc.Hx))+real(down(emnc.By))\*real(down(emnc.Hy))+real(down(emnc.Bz))\*real(down(emnc.Hz)))+real(down(emnc.Bx))\*(real(down(emnc.Hx))\*emnc.unx+real(down(emnc.Hy))\*emnc.uny+real(down(emnc.Hz))\*emnc.unz) | Pa | Maxwell downward magnetic surface stress tensor, x component | Boundaries 1–4, 83, 92 |
| emnc.dnTmy | -0.5\*emnc.uny\*(real(down(emnc.Bx))\*real(down(emnc.Hx))+real(down(emnc.By))\*real(down(emnc.Hy))+real(down(emnc.Bz))\*real(down(emnc.Hz)))+real(down(emnc.By))\*(real(down(emnc.Hx))\*emnc.unx+real(down(emnc.Hy))\*emnc.uny+real(down(emnc.Hz))\*emnc.unz) | Pa | Maxwell downward magnetic surface stress tensor, y component | Boundaries 1–4, 83, 92 |
| emnc.dnTmz | -0.5\*emnc.unz\*(real(down(emnc.Bx))\*real(down(emnc.Hx))+real(down(emnc.By))\*real(down(emnc.Hy))+real(down(emnc.Bz))\*real(down(emnc.Hz)))+real(down(emnc.Bz))\*(real(down(emnc.Hx))\*emnc.unx+real(down(emnc.Hy))\*emnc.uny+real(down(emnc.Hz))\*emnc.unz) | Pa | Maxwell downward magnetic surface stress tensor, z component | Boundaries 1–4, 83, 92 |
| emnc.Hx | -Vmx | A/m | Magnetic field, x component | Domains 1, 13 |
| emnc.Hy | -Vmy | A/m | Magnetic field, y component | Domains 1, 13 |
| emnc.Hz | -Vmz | A/m | Magnetic field, z component | Domains 1, 13 |
| emnc.tHx | -VmTx | A/m | Tangential magnetic field, x component | Boundaries 1–158 |
| emnc.tHy | -VmTy | A/m | Tangential magnetic field, y component | Boundaries 1–158 |
| emnc.tHz | -VmTz | A/m | Tangential magnetic field, z component | Boundaries 1–158 |
| emnc.normH | sqrt(realdot(emnc.Hx,emnc.Hx)+realdot(emnc.Hy,emnc.Hy)+realdot(emnc.Hz,emnc.Hz)) | A/m | Magnetic field norm | Domains 1, 13 |
| emnc.murxx | 1.1 | 1 | Relative permeability, xx component | Domains 1, 13 |
| emnc.muryx | 0 | 1 | Relative permeability, yx component | Domains 1, 13 |
| emnc.murzx | 0 | 1 | Relative permeability, zx component | Domains 1, 13 |
| emnc.murxy | 0 | 1 | Relative permeability, xy component | Domains 1, 13 |
| emnc.muryy | 1.1 | 1 | Relative permeability, yy component | Domains 1, 13 |
| emnc.murzy | 0 | 1 | Relative permeability, zy component | Domains 1, 13 |
| emnc.murxz | 0 | 1 | Relative permeability, xz component | Domains 1, 13 |
| emnc.muryz | 0 | 1 | Relative permeability, yz component | Domains 1, 13 |
| emnc.murzz | 1.1 | 1 | Relative permeability, zz component | Domains 1, 13 |
| emnc.Bx | mu0\_const\*emnc.Ixx\*emnc.Hx+mu0\_const\*emnc.Ixy\*emnc.Hy+mu0\_const\*emnc.Ixz\*emnc.Hz+mu0\_const\*emnc.chimxx\*emnc.Hx+mu0\_const\*emnc.chimxy\*emnc.Hy+mu0\_const\*emnc.chimxz\*emnc.Hz+emnc.Brx | T | Magnetic flux density, x component | Domains 1, 13 |
| emnc.By | mu0\_const\*emnc.Iyx\*emnc.Hx+mu0\_const\*emnc.Iyy\*emnc.Hy+mu0\_const\*emnc.Iyz\*emnc.Hz+mu0\_const\*emnc.chimyx\*emnc.Hx+mu0\_const\*emnc.chimyy\*emnc.Hy+mu0\_const\*emnc.chimyz\*emnc.Hz+emnc.Bry | T | Magnetic flux density, y component | Domains 1, 13 |
| emnc.Bz | mu0\_const\*emnc.Izx\*emnc.Hx+mu0\_const\*emnc.Izy\*emnc.Hy+mu0\_const\*emnc.Izz\*emnc.Hz+mu0\_const\*emnc.chimzx\*emnc.Hx+mu0\_const\*emnc.chimzy\*emnc.Hy+mu0\_const\*emnc.chimzz\*emnc.Hz+emnc.Brz | T | Magnetic flux density, z component | Domains 1, 13 |
| emnc.normB | sqrt(realdot(emnc.Bx,emnc.Bx)+realdot(emnc.By,emnc.By)+realdot(emnc.Bz,emnc.Bz)) | T | Magnetic flux density norm | Domains 1, 13 |
| emnc.Mx | emnc.Bx/mu0\_const-emnc.Ixx\*emnc.Hx-emnc.Ixy\*emnc.Hy-emnc.Ixz\*emnc.Hz | A/m | Magnetization, x component | Domains 1, 13 |
| emnc.My | emnc.By/mu0\_const-emnc.Iyx\*emnc.Hx-emnc.Iyy\*emnc.Hy-emnc.Iyz\*emnc.Hz | A/m | Magnetization, y component | Domains 1, 13 |
| emnc.Mz | emnc.Bz/mu0\_const-emnc.Izx\*emnc.Hx-emnc.Izy\*emnc.Hy-emnc.Izz\*emnc.Hz | A/m | Magnetization, z component | Domains 1, 13 |
| emnc.normM | sqrt(realdot(emnc.Mx,emnc.Mx)+realdot(emnc.My,emnc.My)+realdot(emnc.Mz,emnc.Mz)) | A/m | Magnetization norm | Domains 1, 13 |
| emnc.Ixx | 1 | 1 | Spatial identity matrix, xx component | Domains 1, 13 |
| emnc.Iyx | 0 | 1 | Spatial identity matrix, yx component | Domains 1, 13 |
| emnc.Izx | 0 | 1 | Spatial identity matrix, zx component | Domains 1, 13 |
| emnc.Ixy | 0 | 1 | Spatial identity matrix, xy component | Domains 1, 13 |
| emnc.Iyy | 1 | 1 | Spatial identity matrix, yy component | Domains 1, 13 |
| emnc.Izy | 0 | 1 | Spatial identity matrix, zy component | Domains 1, 13 |
| emnc.Ixz | 0 | 1 | Spatial identity matrix, xz component | Domains 1, 13 |
| emnc.Iyz | 0 | 1 | Spatial identity matrix, yz component | Domains 1, 13 |
| emnc.Izz | 1 | 1 | Spatial identity matrix, zz component | Domains 1, 13 |
| emnc.Brx | 0 | T | Remanent flux density, x component | Domains 1, 13 |
| emnc.Bry | 0 | T | Remanent flux density, y component | Domains 1, 13 |
| emnc.Brz | 0 | T | Remanent flux density, z component | Domains 1, 13 |
| emnc.normBr | sqrt(realdot(emnc.Brx,emnc.Brx)+realdot(emnc.Bry,emnc.Bry)+realdot(emnc.Brz,emnc.Brz)) | T | Remanent flux density norm | Domains 1, 13 |
| emnc.chimxx | -1+emnc.murxx | 1 | Magnetic susceptibility, xx component | Domains 1, 13 |
| emnc.chimyx | emnc.muryx | 1 | Magnetic susceptibility, yx component | Domains 1, 13 |
| emnc.chimzx | emnc.murzx | 1 | Magnetic susceptibility, zx component | Domains 1, 13 |
| emnc.chimxy | emnc.murxy | 1 | Magnetic susceptibility, xy component | Domains 1, 13 |
| emnc.chimyy | -1+emnc.muryy | 1 | Magnetic susceptibility, yy component | Domains 1, 13 |
| emnc.chimzy | emnc.murzy | 1 | Magnetic susceptibility, zy component | Domains 1, 13 |
| emnc.chimxz | emnc.murxz | 1 | Magnetic susceptibility, xz component | Domains 1, 13 |
| emnc.chimyz | emnc.muryz | 1 | Magnetic susceptibility, yz component | Domains 1, 13 |
| emnc.chimzz | -1+emnc.murzz | 1 | Magnetic susceptibility, zz component | Domains 1, 13 |
| emnc.unTx | 0 | Pa | Maxwell upward surface stress tensor, x component | Boundaries 1–4, 83, 92 |
| emnc.unTy | 0 | Pa | Maxwell upward surface stress tensor, y component | Boundaries 1–4, 83, 92 |
| emnc.unTz | 0 | Pa | Maxwell upward surface stress tensor, z component | Boundaries 1–4, 83, 92 |
| emnc.unTx | emnc.unTmx | Pa | Maxwell upward surface stress tensor, x component | Boundaries 71–74, 86–89 |
| emnc.unTy | emnc.unTmy | Pa | Maxwell upward surface stress tensor, y component | Boundaries 71–74, 86–89 |
| emnc.unTz | emnc.unTmz | Pa | Maxwell upward surface stress tensor, z component | Boundaries 71–74, 86–89 |
| emnc.unTx | emnc.unTmx | Pa | Maxwell upward surface stress tensor, x component | Boundaries 5–70, 75–82, 84–85, 90–91, 93–158 |
| emnc.unTy | emnc.unTmy | Pa | Maxwell upward surface stress tensor, y component | Boundaries 5–70, 75–82, 84–85, 90–91, 93–158 |
| emnc.unTz | emnc.unTmz | Pa | Maxwell upward surface stress tensor, z component | Boundaries 5–70, 75–82, 84–85, 90–91, 93–158 |
| emnc.dnTx | emnc.dnTmx | Pa | Maxwell downward surface stress tensor, x component | Boundaries 1–4, 83, 92 |
| emnc.dnTy | emnc.dnTmy | Pa | Maxwell downward surface stress tensor, y component | Boundaries 1–4, 83, 92 |
| emnc.dnTz | emnc.dnTmz | Pa | Maxwell downward surface stress tensor, z component | Boundaries 1–4, 83, 92 |
| emnc.dnTx | emnc.dnTmx | Pa | Maxwell downward surface stress tensor, x component | Boundaries 71–74, 86–89 |
| emnc.dnTy | emnc.dnTmy | Pa | Maxwell downward surface stress tensor, y component | Boundaries 71–74, 86–89 |
| emnc.dnTz | emnc.dnTmz | Pa | Maxwell downward surface stress tensor, z component | Boundaries 71–74, 86–89 |
| emnc.dnTx | 0 | Pa | Maxwell downward surface stress tensor, x component | Boundaries 5–70, 75–82, 84–85, 90–91, 93–158 |
| emnc.dnTy | 0 | Pa | Maxwell downward surface stress tensor, y component | Boundaries 5–70, 75–82, 84–85, 90–91, 93–158 |
| emnc.dnTz | 0 | Pa | Maxwell downward surface stress tensor, z component | Boundaries 5–70, 75–82, 84–85, 90–91, 93–158 |
| emnc.unx | unx |  | Normal vector up direction, x component | Boundaries 1–158 |
| emnc.uny | uny |  | Normal vector up direction, y component | Boundaries 1–158 |
| emnc.unz | unz |  | Normal vector up direction, z component | Boundaries 1–158 |
| emnc.dnx | dnx |  | Normal vector down direction, x component | Boundaries 1–158 |
| emnc.dny | dny |  | Normal vector down direction, y component | Boundaries 1–158 |
| emnc.dnz | dnz |  | Normal vector down direction, z component | Boundaries 1–158 |
| emnc.W | emnc.Wm | J/m^3 | Energy density | Domains 1, 13 |
| emnc.dWm | emnc.Wm | J/m^3 | Integrand for total magnetic energy | Domains 1, 13 |
| emnc.Wm | 0.5\*mu0\_const\*((emnc.murxx\*emnc.Hx+emnc.murxy\*emnc.Hy+emnc.murxz\*emnc.Hz)\*emnc.Hx+(emnc.muryx\*emnc.Hx+emnc.muryy\*emnc.Hy+emnc.muryz\*emnc.Hz)\*emnc.Hy+(emnc.murzx\*emnc.Hx+emnc.murzy\*emnc.Hy+emnc.murzz\*emnc.Hz)\*emnc.Hz) | J/m^3 | Magnetic energy density | Domains 1, 13 |

##### Shape Functions

| Name | Shape function | Unit | Description | Shape frame | Selection |
| --- | --- | --- | --- | --- | --- |
| Vm | Lagrange (Quadratic) | A | Magnetic scalar potential | Material | Domains 1, 13 |

##### Weak Expressions

| Weak expression | Integration frame | Selection |
| --- | --- | --- |
| emnc.d\*(-emnc.Bx\*test(Vmx)-emnc.By\*test(Vmy)-emnc.Bz\*test(Vmz)) | Material | Domains 1, 13 |

#### 2.4.2. Magnetic Insulation 1

Magnetic Insulation 1

Selection

| Geometric entity level | Boundary |
| Selection | No boundaries |

Equations

##### Shape Functions

| Name | Shape function | Unit | Description | Shape frame | Selection |
| --- | --- | --- | --- | --- | --- |
| Vm | Lagrange (Quadratic) | A | Magnetic scalar potential | Material | Boundaries 5–82, 84–91, 93–158 |

#### 2.4.3. Initial Values 1

Initial Values 1

Selection

| Geometric entity level | Domain |
| Selection | Domains 1–26 |

Settings

| Description | Value |
| Magnetic scalar potential | 0 |

#### 2.4.4. Magnetic Flux Conservation 2

Magnetic Flux Conservation 2

Selection

| Geometric entity level | Domain |
| Selection | Domain 26 |

Equations

Settings

| Description | Value |
| Constitutive relation | Remanent flux density |
| Remanent flux density, x component | BpRem\*cos(argBp\*(-1 + 1) + tick) |
| Remanent flux density, y component | BpRem\*sin(argBp\*(-1 + 1) + tick) |
| Remanent flux density, z component | 0 |
| Relative permeability | From material |
| Relative permeability | {{1, 0, 0}, {0, 1, 0}, {0, 0, 1}} |

Properties from material

| Property | Material | Property group |
| Relative permeability | Soft Iron (without losses) | Basic |

##### Variables

| Name | Expression | Unit | Description | Selection |
| --- | --- | --- | --- | --- |
| emnc.dnTmx | -0.5\*emnc.unx\*(real(down(emnc.Bx))\*real(down(emnc.Hx))+real(down(emnc.By))\*real(down(emnc.Hy))+real(down(emnc.Bz))\*real(down(emnc.Hz)))+real(down(emnc.Bx))\*(real(down(emnc.Hx))\*emnc.unx+real(down(emnc.Hy))\*emnc.uny+real(down(emnc.Hz))\*emnc.unz) | Pa | Maxwell downward magnetic surface stress tensor, x component | Boundaries 147–150, 157–158 |
| emnc.dnTmy | -0.5\*emnc.uny\*(real(down(emnc.Bx))\*real(down(emnc.Hx))+real(down(emnc.By))\*real(down(emnc.Hy))+real(down(emnc.Bz))\*real(down(emnc.Hz)))+real(down(emnc.By))\*(real(down(emnc.Hx))\*emnc.unx+real(down(emnc.Hy))\*emnc.uny+real(down(emnc.Hz))\*emnc.unz) | Pa | Maxwell downward magnetic surface stress tensor, y component | Boundaries 147–150, 157–158 |
| emnc.dnTmz | -0.5\*emnc.unz\*(real(down(emnc.Bx))\*real(down(emnc.Hx))+real(down(emnc.By))\*real(down(emnc.Hy))+real(down(emnc.Bz))\*real(down(emnc.Hz)))+real(down(emnc.Bz))\*(real(down(emnc.Hx))\*emnc.unx+real(down(emnc.Hy))\*emnc.uny+real(down(emnc.Hz))\*emnc.unz) | Pa | Maxwell downward magnetic surface stress tensor, z component | Boundaries 147–150, 157–158 |
| emnc.Hx | -Vmx | A/m | Magnetic field, x component | Domain 26 |
| emnc.Hy | -Vmy | A/m | Magnetic field, y component | Domain 26 |
| emnc.Hz | -Vmz | A/m | Magnetic field, z component | Domain 26 |
| emnc.tHx | -VmTx | A/m | Tangential magnetic field, x component | Boundaries 147–150, 157–158 |
| emnc.tHy | -VmTy | A/m | Tangential magnetic field, y component | Boundaries 147–150, 157–158 |
| emnc.tHz | -VmTz | A/m | Tangential magnetic field, z component | Boundaries 147–150, 157–158 |
| emnc.normH | sqrt(realdot(emnc.Hx,emnc.Hx)+realdot(emnc.Hy,emnc.Hy)+realdot(emnc.Hz,emnc.Hz)) | A/m | Magnetic field norm | Domain 26 |
| emnc.murxx | model.input.mur11 | 1 | Relative permeability, xx component | Domain 26 |
| emnc.muryx | model.input.mur21 | 1 | Relative permeability, yx component | Domain 26 |
| emnc.murzx | model.input.mur31 | 1 | Relative permeability, zx component | Domain 26 |
| emnc.murxy | model.input.mur12 | 1 | Relative permeability, xy component | Domain 26 |
| emnc.muryy | model.input.mur22 | 1 | Relative permeability, yy component | Domain 26 |
| emnc.murzy | model.input.mur32 | 1 | Relative permeability, zy component | Domain 26 |
| emnc.murxz | model.input.mur13 | 1 | Relative permeability, xz component | Domain 26 |
| emnc.muryz | model.input.mur23 | 1 | Relative permeability, yz component | Domain 26 |
| emnc.murzz | model.input.mur33 | 1 | Relative permeability, zz component | Domain 26 |
| emnc.Bx | mu0\_const\*emnc.Ixx\*emnc.Hx+mu0\_const\*emnc.Ixy\*emnc.Hy+mu0\_const\*emnc.Ixz\*emnc.Hz+mu0\_const\*emnc.chimxx\*emnc.Hx+mu0\_const\*emnc.chimxy\*emnc.Hy+mu0\_const\*emnc.chimxz\*emnc.Hz+emnc.Brx | T | Magnetic flux density, x component | Domain 26 |
| emnc.By | mu0\_const\*emnc.Iyx\*emnc.Hx+mu0\_const\*emnc.Iyy\*emnc.Hy+mu0\_const\*emnc.Iyz\*emnc.Hz+mu0\_const\*emnc.chimyx\*emnc.Hx+mu0\_const\*emnc.chimyy\*emnc.Hy+mu0\_const\*emnc.chimyz\*emnc.Hz+emnc.Bry | T | Magnetic flux density, y component | Domain 26 |
| emnc.Bz | mu0\_const\*emnc.Izx\*emnc.Hx+mu0\_const\*emnc.Izy\*emnc.Hy+mu0\_const\*emnc.Izz\*emnc.Hz+mu0\_const\*emnc.chimzx\*emnc.Hx+mu0\_const\*emnc.chimzy\*emnc.Hy+mu0\_const\*emnc.chimzz\*emnc.Hz+emnc.Brz | T | Magnetic flux density, z component | Domain 26 |
| emnc.normB | sqrt(realdot(emnc.Bx,emnc.Bx)+realdot(emnc.By,emnc.By)+realdot(emnc.Bz,emnc.Bz)) | T | Magnetic flux density norm | Domain 26 |
| emnc.Mx | emnc.Bx/mu0\_const-emnc.Ixx\*emnc.Hx-emnc.Ixy\*emnc.Hy-emnc.Ixz\*emnc.Hz | A/m | Magnetization, x component | Domain 26 |
| emnc.My | emnc.By/mu0\_const-emnc.Iyx\*emnc.Hx-emnc.Iyy\*emnc.Hy-emnc.Iyz\*emnc.Hz | A/m | Magnetization, y component | Domain 26 |
| emnc.Mz | emnc.Bz/mu0\_const-emnc.Izx\*emnc.Hx-emnc.Izy\*emnc.Hy-emnc.Izz\*emnc.Hz | A/m | Magnetization, z component | Domain 26 |
| emnc.normM | sqrt(realdot(emnc.Mx,emnc.Mx)+realdot(emnc.My,emnc.My)+realdot(emnc.Mz,emnc.Mz)) | A/m | Magnetization norm | Domain 26 |
| emnc.Ixx | 1 | 1 | Spatial identity matrix, xx component | Domain 26 |
| emnc.Iyx | 0 | 1 | Spatial identity matrix, yx component | Domain 26 |
| emnc.Izx | 0 | 1 | Spatial identity matrix, zx component | Domain 26 |
| emnc.Ixy | 0 | 1 | Spatial identity matrix, xy component | Domain 26 |
| emnc.Iyy | 1 | 1 | Spatial identity matrix, yy component | Domain 26 |
| emnc.Izy | 0 | 1 | Spatial identity matrix, zy component | Domain 26 |
| emnc.Ixz | 0 | 1 | Spatial identity matrix, xz component | Domain 26 |
| emnc.Iyz | 0 | 1 | Spatial identity matrix, yz component | Domain 26 |
| emnc.Izz | 1 | 1 | Spatial identity matrix, zz component | Domain 26 |
| emnc.Brx | BpRem\*cos(tick) | T | Remanent flux density, x component | Domain 26 |
| emnc.Bry | BpRem\*sin(tick) | T | Remanent flux density, y component | Domain 26 |
| emnc.Brz | 0 | T | Remanent flux density, z component | Domain 26 |
| emnc.normBr | sqrt(realdot(emnc.Brx,emnc.Brx)+realdot(emnc.Bry,emnc.Bry)+realdot(emnc.Brz,emnc.Brz)) | T | Remanent flux density norm | Domain 26 |
| emnc.chimxx | -1+emnc.murxx | 1 | Magnetic susceptibility, xx component | Domain 26 |
| emnc.chimyx | emnc.muryx | 1 | Magnetic susceptibility, yx component | Domain 26 |
| emnc.chimzx | emnc.murzx | 1 | Magnetic susceptibility, zx component | Domain 26 |
| emnc.chimxy | emnc.murxy | 1 | Magnetic susceptibility, xy component | Domain 26 |
| emnc.chimyy | -1+emnc.muryy | 1 | Magnetic susceptibility, yy component | Domain 26 |
| emnc.chimzy | emnc.murzy | 1 | Magnetic susceptibility, zy component | Domain 26 |
| emnc.chimxz | emnc.murxz | 1 | Magnetic susceptibility, xz component | Domain 26 |
| emnc.chimyz | emnc.muryz | 1 | Magnetic susceptibility, yz component | Domain 26 |
| emnc.chimzz | -1+emnc.murzz | 1 | Magnetic susceptibility, zz component | Domain 26 |
| emnc.unTx | 0 | Pa | Maxwell upward surface stress tensor, x component | Boundaries 147–150, 157–158 |
| emnc.unTy | 0 | Pa | Maxwell upward surface stress tensor, y component | Boundaries 147–150, 157–158 |
| emnc.unTz | 0 | Pa | Maxwell upward surface stress tensor, z component | Boundaries 147–150, 157–158 |
| emnc.dnTx | emnc.dnTmx | Pa | Maxwell downward surface stress tensor, x component | Boundaries 147–150, 157–158 |
| emnc.dnTy | emnc.dnTmy | Pa | Maxwell downward surface stress tensor, y component | Boundaries 147–150, 157–158 |
| emnc.dnTz | emnc.dnTmz | Pa | Maxwell downward surface stress tensor, z component | Boundaries 147–150, 157–158 |
| emnc.unx | unx |  | Normal vector up direction, x component | Boundaries 147–150, 157–158 |
| emnc.uny | uny |  | Normal vector up direction, y component | Boundaries 147–150, 157–158 |
| emnc.unz | unz |  | Normal vector up direction, z component | Boundaries 147–150, 157–158 |
| emnc.dnx | dnx |  | Normal vector down direction, x component | Boundaries 147–150, 157–158 |
| emnc.dny | dny |  | Normal vector down direction, y component | Boundaries 147–150, 157–158 |
| emnc.dnz | dnz |  | Normal vector down direction, z component | Boundaries 147–150, 157–158 |
| emnc.W | emnc.Wm | J/m^3 | Energy density | Domain 26 |
| emnc.dWm | emnc.Wm | J/m^3 | Integrand for total magnetic energy | Domain 26 |
| emnc.Wm | 0.5\*mu0\_const\*((emnc.murxx\*emnc.Hx+emnc.murxy\*emnc.Hy+emnc.murxz\*emnc.Hz)\*emnc.Hx+(emnc.muryx\*emnc.Hx+emnc.muryy\*emnc.Hy+emnc.muryz\*emnc.Hz)\*emnc.Hy+(emnc.murzx\*emnc.Hx+emnc.murzy\*emnc.Hy+emnc.murzz\*emnc.Hz)\*emnc.Hz) | J/m^3 | Magnetic energy density | Domain 26 |

##### Shape Functions

| Name | Shape function | Unit | Description | Shape frame | Selection |
| --- | --- | --- | --- | --- | --- |
| Vm | Lagrange (Quadratic) | A | Magnetic scalar potential | Material | Domain 26 |

##### Weak Expressions

| Weak expression | Integration frame | Selection |
| --- | --- | --- |
| emnc.d\*(-emnc.Bx\*test(Vmx)-emnc.By\*test(Vmy)-emnc.Bz\*test(Vmz)) | Material | Domain 26 |

#### 2.4.5. Magnetic Flux Conservation 3

Magnetic Flux Conservation 3

Selection

| Geometric entity level | Domain |
| Selection | Domain 25 |

Equations

Settings

| Description | Value |
| Constitutive relation | Remanent flux density |
| Remanent flux density, x component | BpRem\*cos(argBp\*(-1 + 2) + tick) |
| Remanent flux density, y component | BpRem\*sin(argBp\*(-1 + 2) + tick) |
| Remanent flux density, z component | 0 |
| Relative permeability | From material |
| Relative permeability | {{1, 0, 0}, {0, 1, 0}, {0, 0, 1}} |

Properties from material

| Property | Material | Property group |
| Relative permeability | Soft Iron (without losses) | Basic |

##### Variables

| Name | Expression | Unit | Description | Selection |
| --- | --- | --- | --- | --- |
| emnc.dnTmx | -0.5\*emnc.unx\*(real(down(emnc.Bx))\*real(down(emnc.Hx))+real(down(emnc.By))\*real(down(emnc.Hy))+real(down(emnc.Bz))\*real(down(emnc.Hz)))+real(down(emnc.Bx))\*(real(down(emnc.Hx))\*emnc.unx+real(down(emnc.Hy))\*emnc.uny+real(down(emnc.Hz))\*emnc.unz) | Pa | Maxwell downward magnetic surface stress tensor, x component | Boundaries 143–146, 154, 156 |
| emnc.dnTmy | -0.5\*emnc.uny\*(real(down(emnc.Bx))\*real(down(emnc.Hx))+real(down(emnc.By))\*real(down(emnc.Hy))+real(down(emnc.Bz))\*real(down(emnc.Hz)))+real(down(emnc.By))\*(real(down(emnc.Hx))\*emnc.unx+real(down(emnc.Hy))\*emnc.uny+real(down(emnc.Hz))\*emnc.unz) | Pa | Maxwell downward magnetic surface stress tensor, y component | Boundaries 143–146, 154, 156 |
| emnc.dnTmz | -0.5\*emnc.unz\*(real(down(emnc.Bx))\*real(down(emnc.Hx))+real(down(emnc.By))\*real(down(emnc.Hy))+real(down(emnc.Bz))\*real(down(emnc.Hz)))+real(down(emnc.Bz))\*(real(down(emnc.Hx))\*emnc.unx+real(down(emnc.Hy))\*emnc.uny+real(down(emnc.Hz))\*emnc.unz) | Pa | Maxwell downward magnetic surface stress tensor, z component | Boundaries 143–146, 154, 156 |
| emnc.Hx | -Vmx | A/m | Magnetic field, x component | Domain 25 |
| emnc.Hy | -Vmy | A/m | Magnetic field, y component | Domain 25 |
| emnc.Hz | -Vmz | A/m | Magnetic field, z component | Domain 25 |
| emnc.tHx | -VmTx | A/m | Tangential magnetic field, x component | Boundaries 143–146, 154, 156 |
| emnc.tHy | -VmTy | A/m | Tangential magnetic field, y component | Boundaries 143–146, 154, 156 |
| emnc.tHz | -VmTz | A/m | Tangential magnetic field, z component | Boundaries 143–146, 154, 156 |
| emnc.normH | sqrt(realdot(emnc.Hx,emnc.Hx)+realdot(emnc.Hy,emnc.Hy)+realdot(emnc.Hz,emnc.Hz)) | A/m | Magnetic field norm | Domain 25 |
| emnc.murxx | model.input.mur11 | 1 | Relative permeability, xx component | Domain 25 |
| emnc.muryx | model.input.mur21 | 1 | Relative permeability, yx component | Domain 25 |
| emnc.murzx | model.input.mur31 | 1 | Relative permeability, zx component | Domain 25 |
| emnc.murxy | model.input.mur12 | 1 | Relative permeability, xy component | Domain 25 |
| emnc.muryy | model.input.mur22 | 1 | Relative permeability, yy component | Domain 25 |
| emnc.murzy | model.input.mur32 | 1 | Relative permeability, zy component | Domain 25 |
| emnc.murxz | model.input.mur13 | 1 | Relative permeability, xz component | Domain 25 |
| emnc.muryz | model.input.mur23 | 1 | Relative permeability, yz component | Domain 25 |
| emnc.murzz | model.input.mur33 | 1 | Relative permeability, zz component | Domain 25 |
| emnc.Bx | mu0\_const\*emnc.Ixx\*emnc.Hx+mu0\_const\*emnc.Ixy\*emnc.Hy+mu0\_const\*emnc.Ixz\*emnc.Hz+mu0\_const\*emnc.chimxx\*emnc.Hx+mu0\_const\*emnc.chimxy\*emnc.Hy+mu0\_const\*emnc.chimxz\*emnc.Hz+emnc.Brx | T | Magnetic flux density, x component | Domain 25 |
| emnc.By | mu0\_const\*emnc.Iyx\*emnc.Hx+mu0\_const\*emnc.Iyy\*emnc.Hy+mu0\_const\*emnc.Iyz\*emnc.Hz+mu0\_const\*emnc.chimyx\*emnc.Hx+mu0\_const\*emnc.chimyy\*emnc.Hy+mu0\_const\*emnc.chimyz\*emnc.Hz+emnc.Bry | T | Magnetic flux density, y component | Domain 25 |
| emnc.Bz | mu0\_const\*emnc.Izx\*emnc.Hx+mu0\_const\*emnc.Izy\*emnc.Hy+mu0\_const\*emnc.Izz\*emnc.Hz+mu0\_const\*emnc.chimzx\*emnc.Hx+mu0\_const\*emnc.chimzy\*emnc.Hy+mu0\_const\*emnc.chimzz\*emnc.Hz+emnc.Brz | T | Magnetic flux density, z component | Domain 25 |
| emnc.normB | sqrt(realdot(emnc.Bx,emnc.Bx)+realdot(emnc.By,emnc.By)+realdot(emnc.Bz,emnc.Bz)) | T | Magnetic flux density norm | Domain 25 |
| emnc.Mx | emnc.Bx/mu0\_const-emnc.Ixx\*emnc.Hx-emnc.Ixy\*emnc.Hy-emnc.Ixz\*emnc.Hz | A/m | Magnetization, x component | Domain 25 |
| emnc.My | emnc.By/mu0\_const-emnc.Iyx\*emnc.Hx-emnc.Iyy\*emnc.Hy-emnc.Iyz\*emnc.Hz | A/m | Magnetization, y component | Domain 25 |
| emnc.Mz | emnc.Bz/mu0\_const-emnc.Izx\*emnc.Hx-emnc.Izy\*emnc.Hy-emnc.Izz\*emnc.Hz | A/m | Magnetization, z component | Domain 25 |
| emnc.normM | sqrt(realdot(emnc.Mx,emnc.Mx)+realdot(emnc.My,emnc.My)+realdot(emnc.Mz,emnc.Mz)) | A/m | Magnetization norm | Domain 25 |
| emnc.Ixx | 1 | 1 | Spatial identity matrix, xx component | Domain 25 |
| emnc.Iyx | 0 | 1 | Spatial identity matrix, yx component | Domain 25 |
| emnc.Izx | 0 | 1 | Spatial identity matrix, zx component | Domain 25 |
| emnc.Ixy | 0 | 1 | Spatial identity matrix, xy component | Domain 25 |
| emnc.Iyy | 1 | 1 | Spatial identity matrix, yy component | Domain 25 |
| emnc.Izy | 0 | 1 | Spatial identity matrix, zy component | Domain 25 |
| emnc.Ixz | 0 | 1 | Spatial identity matrix, xz component | Domain 25 |
| emnc.Iyz | 0 | 1 | Spatial identity matrix, yz component | Domain 25 |
| emnc.Izz | 1 | 1 | Spatial identity matrix, zz component | Domain 25 |
| emnc.Brx | BpRem\*cos(argBp+tick) | T | Remanent flux density, x component | Domain 25 |
| emnc.Bry | BpRem\*sin(argBp+tick) | T | Remanent flux density, y component | Domain 25 |
| emnc.Brz | 0 | T | Remanent flux density, z component | Domain 25 |
| emnc.normBr | sqrt(realdot(emnc.Brx,emnc.Brx)+realdot(emnc.Bry,emnc.Bry)+realdot(emnc.Brz,emnc.Brz)) | T | Remanent flux density norm | Domain 25 |
| emnc.chimxx | -1+emnc.murxx | 1 | Magnetic susceptibility, xx component | Domain 25 |
| emnc.chimyx | emnc.muryx | 1 | Magnetic susceptibility, yx component | Domain 25 |
| emnc.chimzx | emnc.murzx | 1 | Magnetic susceptibility, zx component | Domain 25 |
| emnc.chimxy | emnc.murxy | 1 | Magnetic susceptibility, xy component | Domain 25 |
| emnc.chimyy | -1+emnc.muryy | 1 | Magnetic susceptibility, yy component | Domain 25 |
| emnc.chimzy | emnc.murzy | 1 | Magnetic susceptibility, zy component | Domain 25 |
| emnc.chimxz | emnc.murxz | 1 | Magnetic susceptibility, xz component | Domain 25 |
| emnc.chimyz | emnc.muryz | 1 | Magnetic susceptibility, yz component | Domain 25 |
| emnc.chimzz | -1+emnc.murzz | 1 | Magnetic susceptibility, zz component | Domain 25 |
| emnc.unTx | 0 | Pa | Maxwell upward surface stress tensor, x component | Boundaries 143–146, 154, 156 |
| emnc.unTy | 0 | Pa | Maxwell upward surface stress tensor, y component | Boundaries 143–146, 154, 156 |
| emnc.unTz | 0 | Pa | Maxwell upward surface stress tensor, z component | Boundaries 143–146, 154, 156 |
| emnc.dnTx | emnc.dnTmx | Pa | Maxwell downward surface stress tensor, x component | Boundaries 143–146, 154, 156 |
| emnc.dnTy | emnc.dnTmy | Pa | Maxwell downward surface stress tensor, y component | Boundaries 143–146, 154, 156 |
| emnc.dnTz | emnc.dnTmz | Pa | Maxwell downward surface stress tensor, z component | Boundaries 143–146, 154, 156 |
| emnc.unx | unx |  | Normal vector up direction, x component | Boundaries 143–146, 154, 156 |
| emnc.uny | uny |  | Normal vector up direction, y component | Boundaries 143–146, 154, 156 |
| emnc.unz | unz |  | Normal vector up direction, z component | Boundaries 143–146, 154, 156 |
| emnc.dnx | dnx |  | Normal vector down direction, x component | Boundaries 143–146, 154, 156 |
| emnc.dny | dny |  | Normal vector down direction, y component | Boundaries 143–146, 154, 156 |
| emnc.dnz | dnz |  | Normal vector down direction, z component | Boundaries 143–146, 154, 156 |
| emnc.W | emnc.Wm | J/m^3 | Energy density | Domain 25 |
| emnc.dWm | emnc.Wm | J/m^3 | Integrand for total magnetic energy | Domain 25 |
| emnc.Wm | 0.5\*mu0\_const\*((emnc.murxx\*emnc.Hx+emnc.murxy\*emnc.Hy+emnc.murxz\*emnc.Hz)\*emnc.Hx+(emnc.muryx\*emnc.Hx+emnc.muryy\*emnc.Hy+emnc.muryz\*emnc.Hz)\*emnc.Hy+(emnc.murzx\*emnc.Hx+emnc.murzy\*emnc.Hy+emnc.murzz\*emnc.Hz)\*emnc.Hz) | J/m^3 | Magnetic energy density | Domain 25 |

##### Shape Functions

| Name | Shape function | Unit | Description | Shape frame | Selection |
| --- | --- | --- | --- | --- | --- |
| Vm | Lagrange (Quadratic) | A | Magnetic scalar potential | Material | Domain 25 |

##### Weak Expressions

| Weak expression | Integration frame | Selection |
| --- | --- | --- |
| emnc.d\*(-emnc.Bx\*test(Vmx)-emnc.By\*test(Vmy)-emnc.Bz\*test(Vmz)) | Material | Domain 25 |

#### 2.4.6. Magnetic Flux Conservation 4

Magnetic Flux Conservation 4

Selection

| Geometric entity level | Domain |
| Selection | Domain 23 |

Equations

Settings

| Description | Value |
| Constitutive relation | Remanent flux density |
| Remanent flux density, x component | BpRem\*cos(argBp\*(-1 + 3) + tick) |
| Remanent flux density, y component | BpRem\*sin(argBp\*(-1 + 3) + tick) |
| Remanent flux density, z component | 0 |
| Relative permeability | From material |
| Relative permeability | {{1, 0, 0}, {0, 1, 0}, {0, 0, 1}} |

Properties from material

| Property | Material | Property group |
| Relative permeability | Soft Iron (without losses) | Basic |

##### Variables

| Name | Expression | Unit | Description | Selection |
| --- | --- | --- | --- | --- |
| emnc.dnTmx | -0.5\*emnc.unx\*(real(down(emnc.Bx))\*real(down(emnc.Hx))+real(down(emnc.By))\*real(down(emnc.Hy))+real(down(emnc.Bz))\*real(down(emnc.Hz)))+real(down(emnc.Bx))\*(real(down(emnc.Hx))\*emnc.unx+real(down(emnc.Hy))\*emnc.uny+real(down(emnc.Hz))\*emnc.unz) | Pa | Maxwell downward magnetic surface stress tensor, x component | Boundaries 133–136, 138, 152 |
| emnc.dnTmy | -0.5\*emnc.uny\*(real(down(emnc.Bx))\*real(down(emnc.Hx))+real(down(emnc.By))\*real(down(emnc.Hy))+real(down(emnc.Bz))\*real(down(emnc.Hz)))+real(down(emnc.By))\*(real(down(emnc.Hx))\*emnc.unx+real(down(emnc.Hy))\*emnc.uny+real(down(emnc.Hz))\*emnc.unz) | Pa | Maxwell downward magnetic surface stress tensor, y component | Boundaries 133–136, 138, 152 |
| emnc.dnTmz | -0.5\*emnc.unz\*(real(down(emnc.Bx))\*real(down(emnc.Hx))+real(down(emnc.By))\*real(down(emnc.Hy))+real(down(emnc.Bz))\*real(down(emnc.Hz)))+real(down(emnc.Bz))\*(real(down(emnc.Hx))\*emnc.unx+real(down(emnc.Hy))\*emnc.uny+real(down(emnc.Hz))\*emnc.unz) | Pa | Maxwell downward magnetic surface stress tensor, z component | Boundaries 133–136, 138, 152 |
| emnc.Hx | -Vmx | A/m | Magnetic field, x component | Domain 23 |
| emnc.Hy | -Vmy | A/m | Magnetic field, y component | Domain 23 |
| emnc.Hz | -Vmz | A/m | Magnetic field, z component | Domain 23 |
| emnc.tHx | -VmTx | A/m | Tangential magnetic field, x component | Boundaries 133–136, 138, 152 |
| emnc.tHy | -VmTy | A/m | Tangential magnetic field, y component | Boundaries 133–136, 138, 152 |
| emnc.tHz | -VmTz | A/m | Tangential magnetic field, z component | Boundaries 133–136, 138, 152 |
| emnc.normH | sqrt(realdot(emnc.Hx,emnc.Hx)+realdot(emnc.Hy,emnc.Hy)+realdot(emnc.Hz,emnc.Hz)) | A/m | Magnetic field norm | Domain 23 |
| emnc.murxx | model.input.mur11 | 1 | Relative permeability, xx component | Domain 23 |
| emnc.muryx | model.input.mur21 | 1 | Relative permeability, yx component | Domain 23 |
| emnc.murzx | model.input.mur31 | 1 | Relative permeability, zx component | Domain 23 |
| emnc.murxy | model.input.mur12 | 1 | Relative permeability, xy component | Domain 23 |
| emnc.muryy | model.input.mur22 | 1 | Relative permeability, yy component | Domain 23 |
| emnc.murzy | model.input.mur32 | 1 | Relative permeability, zy component | Domain 23 |
| emnc.murxz | model.input.mur13 | 1 | Relative permeability, xz component | Domain 23 |
| emnc.muryz | model.input.mur23 | 1 | Relative permeability, yz component | Domain 23 |
| emnc.murzz | model.input.mur33 | 1 | Relative permeability, zz component | Domain 23 |
| emnc.Bx | mu0\_const\*emnc.Ixx\*emnc.Hx+mu0\_const\*emnc.Ixy\*emnc.Hy+mu0\_const\*emnc.Ixz\*emnc.Hz+mu0\_const\*emnc.chimxx\*emnc.Hx+mu0\_const\*emnc.chimxy\*emnc.Hy+mu0\_const\*emnc.chimxz\*emnc.Hz+emnc.Brx | T | Magnetic flux density, x component | Domain 23 |
| emnc.By | mu0\_const\*emnc.Iyx\*emnc.Hx+mu0\_const\*emnc.Iyy\*emnc.Hy+mu0\_const\*emnc.Iyz\*emnc.Hz+mu0\_const\*emnc.chimyx\*emnc.Hx+mu0\_const\*emnc.chimyy\*emnc.Hy+mu0\_const\*emnc.chimyz\*emnc.Hz+emnc.Bry | T | Magnetic flux density, y component | Domain 23 |
| emnc.Bz | mu0\_const\*emnc.Izx\*emnc.Hx+mu0\_const\*emnc.Izy\*emnc.Hy+mu0\_const\*emnc.Izz\*emnc.Hz+mu0\_const\*emnc.chimzx\*emnc.Hx+mu0\_const\*emnc.chimzy\*emnc.Hy+mu0\_const\*emnc.chimzz\*emnc.Hz+emnc.Brz | T | Magnetic flux density, z component | Domain 23 |
| emnc.normB | sqrt(realdot(emnc.Bx,emnc.Bx)+realdot(emnc.By,emnc.By)+realdot(emnc.Bz,emnc.Bz)) | T | Magnetic flux density norm | Domain 23 |
| emnc.Mx | emnc.Bx/mu0\_const-emnc.Ixx\*emnc.Hx-emnc.Ixy\*emnc.Hy-emnc.Ixz\*emnc.Hz | A/m | Magnetization, x component | Domain 23 |
| emnc.My | emnc.By/mu0\_const-emnc.Iyx\*emnc.Hx-emnc.Iyy\*emnc.Hy-emnc.Iyz\*emnc.Hz | A/m | Magnetization, y component | Domain 23 |
| emnc.Mz | emnc.Bz/mu0\_const-emnc.Izx\*emnc.Hx-emnc.Izy\*emnc.Hy-emnc.Izz\*emnc.Hz | A/m | Magnetization, z component | Domain 23 |
| emnc.normM | sqrt(realdot(emnc.Mx,emnc.Mx)+realdot(emnc.My,emnc.My)+realdot(emnc.Mz,emnc.Mz)) | A/m | Magnetization norm | Domain 23 |
| emnc.Ixx | 1 | 1 | Spatial identity matrix, xx component | Domain 23 |
| emnc.Iyx | 0 | 1 | Spatial identity matrix, yx component | Domain 23 |
| emnc.Izx | 0 | 1 | Spatial identity matrix, zx component | Domain 23 |
| emnc.Ixy | 0 | 1 | Spatial identity matrix, xy component | Domain 23 |
| emnc.Iyy | 1 | 1 | Spatial identity matrix, yy component | Domain 23 |
| emnc.Izy | 0 | 1 | Spatial identity matrix, zy component | Domain 23 |
| emnc.Ixz | 0 | 1 | Spatial identity matrix, xz component | Domain 23 |
| emnc.Iyz | 0 | 1 | Spatial identity matrix, yz component | Domain 23 |
| emnc.Izz | 1 | 1 | Spatial identity matrix, zz component | Domain 23 |
| emnc.Brx | BpRem\*cos(2\*argBp+tick) | T | Remanent flux density, x component | Domain 23 |
| emnc.Bry | BpRem\*sin(2\*argBp+tick) | T | Remanent flux density, y component | Domain 23 |
| emnc.Brz | 0 | T | Remanent flux density, z component | Domain 23 |
| emnc.normBr | sqrt(realdot(emnc.Brx,emnc.Brx)+realdot(emnc.Bry,emnc.Bry)+realdot(emnc.Brz,emnc.Brz)) | T | Remanent flux density norm | Domain 23 |
| emnc.chimxx | -1+emnc.murxx | 1 | Magnetic susceptibility, xx component | Domain 23 |
| emnc.chimyx | emnc.muryx | 1 | Magnetic susceptibility, yx component | Domain 23 |
| emnc.chimzx | emnc.murzx | 1 | Magnetic susceptibility, zx component | Domain 23 |
| emnc.chimxy | emnc.murxy | 1 | Magnetic susceptibility, xy component | Domain 23 |
| emnc.chimyy | -1+emnc.muryy | 1 | Magnetic susceptibility, yy component | Domain 23 |
| emnc.chimzy | emnc.murzy | 1 | Magnetic susceptibility, zy component | Domain 23 |
| emnc.chimxz | emnc.murxz | 1 | Magnetic susceptibility, xz component | Domain 23 |
| emnc.chimyz | emnc.muryz | 1 | Magnetic susceptibility, yz component | Domain 23 |
| emnc.chimzz | -1+emnc.murzz | 1 | Magnetic susceptibility, zz component | Domain 23 |
| emnc.unTx | 0 | Pa | Maxwell upward surface stress tensor, x component | Boundaries 133–136, 138, 152 |
| emnc.unTy | 0 | Pa | Maxwell upward surface stress tensor, y component | Boundaries 133–136, 138, 152 |
| emnc.unTz | 0 | Pa | Maxwell upward surface stress tensor, z component | Boundaries 133–136, 138, 152 |
| emnc.dnTx | emnc.dnTmx | Pa | Maxwell downward surface stress tensor, x component | Boundaries 133–136, 138, 152 |
| emnc.dnTy | emnc.dnTmy | Pa | Maxwell downward surface stress tensor, y component | Boundaries 133–136, 138, 152 |
| emnc.dnTz | emnc.dnTmz | Pa | Maxwell downward surface stress tensor, z component | Boundaries 133–136, 138, 152 |
| emnc.unx | unx |  | Normal vector up direction, x component | Boundaries 133–136, 138, 152 |
| emnc.uny | uny |  | Normal vector up direction, y component | Boundaries 133–136, 138, 152 |
| emnc.unz | unz |  | Normal vector up direction, z component | Boundaries 133–136, 138, 152 |
| emnc.dnx | dnx |  | Normal vector down direction, x component | Boundaries 133–136, 138, 152 |
| emnc.dny | dny |  | Normal vector down direction, y component | Boundaries 133–136, 138, 152 |
| emnc.dnz | dnz |  | Normal vector down direction, z component | Boundaries 133–136, 138, 152 |
| emnc.W | emnc.Wm | J/m^3 | Energy density | Domain 23 |
| emnc.dWm | emnc.Wm | J/m^3 | Integrand for total magnetic energy | Domain 23 |
| emnc.Wm | 0.5\*mu0\_const\*((emnc.murxx\*emnc.Hx+emnc.murxy\*emnc.Hy+emnc.murxz\*emnc.Hz)\*emnc.Hx+(emnc.muryx\*emnc.Hx+emnc.muryy\*emnc.Hy+emnc.muryz\*emnc.Hz)\*emnc.Hy+(emnc.murzx\*emnc.Hx+emnc.murzy\*emnc.Hy+emnc.murzz\*emnc.Hz)\*emnc.Hz) | J/m^3 | Magnetic energy density | Domain 23 |

##### Shape Functions

| Name | Shape function | Unit | Description | Shape frame | Selection |
| --- | --- | --- | --- | --- | --- |
| Vm | Lagrange (Quadratic) | A | Magnetic scalar potential | Material | Domain 23 |

##### Weak Expressions

| Weak expression | Integration frame | Selection |
| --- | --- | --- |
| emnc.d\*(-emnc.Bx\*test(Vmx)-emnc.By\*test(Vmy)-emnc.Bz\*test(Vmz)) | Material | Domain 23 |

#### 2.4.7. Magnetic Flux Conservation 5

Magnetic Flux Conservation 5

Selection

| Geometric entity level | Domain |
| Selection | Domain 21 |

Equations

Settings

| Description | Value |
| Constitutive relation | Remanent flux density |
| Remanent flux density, x component | BpRem\*cos(argBp\*(-1 + 4) + tick) |
| Remanent flux density, y component | BpRem\*sin(argBp\*(-1 + 4) + tick) |
| Remanent flux density, z component | 0 |
| Relative permeability | From material |
| Relative permeability | {{1, 0, 0}, {0, 1, 0}, {0, 0, 1}} |

Properties from material

| Property | Material | Property group |
| Relative permeability | Soft Iron (without losses) | Basic |

##### Variables

| Name | Expression | Unit | Description | Selection |
| --- | --- | --- | --- | --- |
| emnc.dnTmx | -0.5\*emnc.unx\*(real(down(emnc.Bx))\*real(down(emnc.Hx))+real(down(emnc.By))\*real(down(emnc.Hy))+real(down(emnc.Bz))\*real(down(emnc.Hz)))+real(down(emnc.Bx))\*(real(down(emnc.Hx))\*emnc.unx+real(down(emnc.Hy))\*emnc.uny+real(down(emnc.Hz))\*emnc.unz) | Pa | Maxwell downward magnetic surface stress tensor, x component | Boundaries 122–126, 128 |
| emnc.dnTmy | -0.5\*emnc.uny\*(real(down(emnc.Bx))\*real(down(emnc.Hx))+real(down(emnc.By))\*real(down(emnc.Hy))+real(down(emnc.Bz))\*real(down(emnc.Hz)))+real(down(emnc.By))\*(real(down(emnc.Hx))\*emnc.unx+real(down(emnc.Hy))\*emnc.uny+real(down(emnc.Hz))\*emnc.unz) | Pa | Maxwell downward magnetic surface stress tensor, y component | Boundaries 122–126, 128 |
| emnc.dnTmz | -0.5\*emnc.unz\*(real(down(emnc.Bx))\*real(down(emnc.Hx))+real(down(emnc.By))\*real(down(emnc.Hy))+real(down(emnc.Bz))\*real(down(emnc.Hz)))+real(down(emnc.Bz))\*(real(down(emnc.Hx))\*emnc.unx+real(down(emnc.Hy))\*emnc.uny+real(down(emnc.Hz))\*emnc.unz) | Pa | Maxwell downward magnetic surface stress tensor, z component | Boundaries 122–126, 128 |
| emnc.Hx | -Vmx | A/m | Magnetic field, x component | Domain 21 |
| emnc.Hy | -Vmy | A/m | Magnetic field, y component | Domain 21 |
| emnc.Hz | -Vmz | A/m | Magnetic field, z component | Domain 21 |
| emnc.tHx | -VmTx | A/m | Tangential magnetic field, x component | Boundaries 122–126, 128 |
| emnc.tHy | -VmTy | A/m | Tangential magnetic field, y component | Boundaries 122–126, 128 |
| emnc.tHz | -VmTz | A/m | Tangential magnetic field, z component | Boundaries 122–126, 128 |
| emnc.normH | sqrt(realdot(emnc.Hx,emnc.Hx)+realdot(emnc.Hy,emnc.Hy)+realdot(emnc.Hz,emnc.Hz)) | A/m | Magnetic field norm | Domain 21 |
| emnc.murxx | model.input.mur11 | 1 | Relative permeability, xx component | Domain 21 |
| emnc.muryx | model.input.mur21 | 1 | Relative permeability, yx component | Domain 21 |
| emnc.murzx | model.input.mur31 | 1 | Relative permeability, zx component | Domain 21 |
| emnc.murxy | model.input.mur12 | 1 | Relative permeability, xy component | Domain 21 |
| emnc.muryy | model.input.mur22 | 1 | Relative permeability, yy component | Domain 21 |
| emnc.murzy | model.input.mur32 | 1 | Relative permeability, zy component | Domain 21 |
| emnc.murxz | model.input.mur13 | 1 | Relative permeability, xz component | Domain 21 |
| emnc.muryz | model.input.mur23 | 1 | Relative permeability, yz component | Domain 21 |
| emnc.murzz | model.input.mur33 | 1 | Relative permeability, zz component | Domain 21 |
| emnc.Bx | mu0\_const\*emnc.Ixx\*emnc.Hx+mu0\_const\*emnc.Ixy\*emnc.Hy+mu0\_const\*emnc.Ixz\*emnc.Hz+mu0\_const\*emnc.chimxx\*emnc.Hx+mu0\_const\*emnc.chimxy\*emnc.Hy+mu0\_const\*emnc.chimxz\*emnc.Hz+emnc.Brx | T | Magnetic flux density, x component | Domain 21 |
| emnc.By | mu0\_const\*emnc.Iyx\*emnc.Hx+mu0\_const\*emnc.Iyy\*emnc.Hy+mu0\_const\*emnc.Iyz\*emnc.Hz+mu0\_const\*emnc.chimyx\*emnc.Hx+mu0\_const\*emnc.chimyy\*emnc.Hy+mu0\_const\*emnc.chimyz\*emnc.Hz+emnc.Bry | T | Magnetic flux density, y component | Domain 21 |
| emnc.Bz | mu0\_const\*emnc.Izx\*emnc.Hx+mu0\_const\*emnc.Izy\*emnc.Hy+mu0\_const\*emnc.Izz\*emnc.Hz+mu0\_const\*emnc.chimzx\*emnc.Hx+mu0\_const\*emnc.chimzy\*emnc.Hy+mu0\_const\*emnc.chimzz\*emnc.Hz+emnc.Brz | T | Magnetic flux density, z component | Domain 21 |
| emnc.normB | sqrt(realdot(emnc.Bx,emnc.Bx)+realdot(emnc.By,emnc.By)+realdot(emnc.Bz,emnc.Bz)) | T | Magnetic flux density norm | Domain 21 |
| emnc.Mx | emnc.Bx/mu0\_const-emnc.Ixx\*emnc.Hx-emnc.Ixy\*emnc.Hy-emnc.Ixz\*emnc.Hz | A/m | Magnetization, x component | Domain 21 |
| emnc.My | emnc.By/mu0\_const-emnc.Iyx\*emnc.Hx-emnc.Iyy\*emnc.Hy-emnc.Iyz\*emnc.Hz | A/m | Magnetization, y component | Domain 21 |
| emnc.Mz | emnc.Bz/mu0\_const-emnc.Izx\*emnc.Hx-emnc.Izy\*emnc.Hy-emnc.Izz\*emnc.Hz | A/m | Magnetization, z component | Domain 21 |
| emnc.normM | sqrt(realdot(emnc.Mx,emnc.Mx)+realdot(emnc.My,emnc.My)+realdot(emnc.Mz,emnc.Mz)) | A/m | Magnetization norm | Domain 21 |
| emnc.Ixx | 1 | 1 | Spatial identity matrix, xx component | Domain 21 |
| emnc.Iyx | 0 | 1 | Spatial identity matrix, yx component | Domain 21 |
| emnc.Izx | 0 | 1 | Spatial identity matrix, zx component | Domain 21 |
| emnc.Ixy | 0 | 1 | Spatial identity matrix, xy component | Domain 21 |
| emnc.Iyy | 1 | 1 | Spatial identity matrix, yy component | Domain 21 |
| emnc.Izy | 0 | 1 | Spatial identity matrix, zy component | Domain 21 |
| emnc.Ixz | 0 | 1 | Spatial identity matrix, xz component | Domain 21 |
| emnc.Iyz | 0 | 1 | Spatial identity matrix, yz component | Domain 21 |
| emnc.Izz | 1 | 1 | Spatial identity matrix, zz component | Domain 21 |
| emnc.Brx | BpRem\*cos(3\*argBp+tick) | T | Remanent flux density, x component | Domain 21 |
| emnc.Bry | BpRem\*sin(3\*argBp+tick) | T | Remanent flux density, y component | Domain 21 |
| emnc.Brz | 0 | T | Remanent flux density, z component | Domain 21 |
| emnc.normBr | sqrt(realdot(emnc.Brx,emnc.Brx)+realdot(emnc.Bry,emnc.Bry)+realdot(emnc.Brz,emnc.Brz)) | T | Remanent flux density norm | Domain 21 |
| emnc.chimxx | -1+emnc.murxx | 1 | Magnetic susceptibility, xx component | Domain 21 |
| emnc.chimyx | emnc.muryx | 1 | Magnetic susceptibility, yx component | Domain 21 |
| emnc.chimzx | emnc.murzx | 1 | Magnetic susceptibility, zx component | Domain 21 |
| emnc.chimxy | emnc.murxy | 1 | Magnetic susceptibility, xy component | Domain 21 |
| emnc.chimyy | -1+emnc.muryy | 1 | Magnetic susceptibility, yy component | Domain 21 |
| emnc.chimzy | emnc.murzy | 1 | Magnetic susceptibility, zy component | Domain 21 |
| emnc.chimxz | emnc.murxz | 1 | Magnetic susceptibility, xz component | Domain 21 |
| emnc.chimyz | emnc.muryz | 1 | Magnetic susceptibility, yz component | Domain 21 |
| emnc.chimzz | -1+emnc.murzz | 1 | Magnetic susceptibility, zz component | Domain 21 |
| emnc.unTx | 0 | Pa | Maxwell upward surface stress tensor, x component | Boundaries 122–126, 128 |
| emnc.unTy | 0 | Pa | Maxwell upward surface stress tensor, y component | Boundaries 122–126, 128 |
| emnc.unTz | 0 | Pa | Maxwell upward surface stress tensor, z component | Boundaries 122–126, 128 |
| emnc.dnTx | emnc.dnTmx | Pa | Maxwell downward surface stress tensor, x component | Boundaries 122–126, 128 |
| emnc.dnTy | emnc.dnTmy | Pa | Maxwell downward surface stress tensor, y component | Boundaries 122–126, 128 |
| emnc.dnTz | emnc.dnTmz | Pa | Maxwell downward surface stress tensor, z component | Boundaries 122–126, 128 |
| emnc.unx | unx |  | Normal vector up direction, x component | Boundaries 122–126, 128 |
| emnc.uny | uny |  | Normal vector up direction, y component | Boundaries 122–126, 128 |
| emnc.unz | unz |  | Normal vector up direction, z component | Boundaries 122–126, 128 |
| emnc.dnx | dnx |  | Normal vector down direction, x component | Boundaries 122–126, 128 |
| emnc.dny | dny |  | Normal vector down direction, y component | Boundaries 122–126, 128 |
| emnc.dnz | dnz |  | Normal vector down direction, z component | Boundaries 122–126, 128 |
| emnc.W | emnc.Wm | J/m^3 | Energy density | Domain 21 |
| emnc.dWm | emnc.Wm | J/m^3 | Integrand for total magnetic energy | Domain 21 |
| emnc.Wm | 0.5\*mu0\_const\*((emnc.murxx\*emnc.Hx+emnc.murxy\*emnc.Hy+emnc.murxz\*emnc.Hz)\*emnc.Hx+(emnc.muryx\*emnc.Hx+emnc.muryy\*emnc.Hy+emnc.muryz\*emnc.Hz)\*emnc.Hy+(emnc.murzx\*emnc.Hx+emnc.murzy\*emnc.Hy+emnc.murzz\*emnc.Hz)\*emnc.Hz) | J/m^3 | Magnetic energy density | Domain 21 |

##### Shape Functions

| Name | Shape function | Unit | Description | Shape frame | Selection |
| --- | --- | --- | --- | --- | --- |
| Vm | Lagrange (Quadratic) | A | Magnetic scalar potential | Material | Domain 21 |

##### Weak Expressions

| Weak expression | Integration frame | Selection |
| --- | --- | --- |
| emnc.d\*(-emnc.Bx\*test(Vmx)-emnc.By\*test(Vmy)-emnc.Bz\*test(Vmz)) | Material | Domain 21 |

#### 2.4.8. Magnetic Flux Conservation 6

Magnetic Flux Conservation 6

Selection

| Geometric entity level | Domain |
| Selection | Domain 19 |

Equations

Settings

| Description | Value |
| Constitutive relation | Remanent flux density |
| Remanent flux density, x component | BpRem\*cos(argBp\*(-1 + 5) + tick) |
| Remanent flux density, y component | BpRem\*sin(argBp\*(-1 + 5) + tick) |
| Remanent flux density, z component | 0 |
| Relative permeability | From material |
| Relative permeability | {{1, 0, 0}, {0, 1, 0}, {0, 0, 1}} |

Properties from material

| Property | Material | Property group |
| Relative permeability | Soft Iron (without losses) | Basic |

##### Variables

| Name | Expression | Unit | Description | Selection |
| --- | --- | --- | --- | --- |
| emnc.dnTmx | -0.5\*emnc.unx\*(real(down(emnc.Bx))\*real(down(emnc.Hx))+real(down(emnc.By))\*real(down(emnc.Hy))+real(down(emnc.Bz))\*real(down(emnc.Hz)))+real(down(emnc.Bx))\*(real(down(emnc.Hx))\*emnc.unx+real(down(emnc.Hy))\*emnc.uny+real(down(emnc.Hz))\*emnc.unz) | Pa | Maxwell downward magnetic surface stress tensor, x component | Boundaries 109–112, 114, 116 |
| emnc.dnTmy | -0.5\*emnc.uny\*(real(down(emnc.Bx))\*real(down(emnc.Hx))+real(down(emnc.By))\*real(down(emnc.Hy))+real(down(emnc.Bz))\*real(down(emnc.Hz)))+real(down(emnc.By))\*(real(down(emnc.Hx))\*emnc.unx+real(down(emnc.Hy))\*emnc.uny+real(down(emnc.Hz))\*emnc.unz) | Pa | Maxwell downward magnetic surface stress tensor, y component | Boundaries 109–112, 114, 116 |
| emnc.dnTmz | -0.5\*emnc.unz\*(real(down(emnc.Bx))\*real(down(emnc.Hx))+real(down(emnc.By))\*real(down(emnc.Hy))+real(down(emnc.Bz))\*real(down(emnc.Hz)))+real(down(emnc.Bz))\*(real(down(emnc.Hx))\*emnc.unx+real(down(emnc.Hy))\*emnc.uny+real(down(emnc.Hz))\*emnc.unz) | Pa | Maxwell downward magnetic surface stress tensor, z component | Boundaries 109–112, 114, 116 |
| emnc.Hx | -Vmx | A/m | Magnetic field, x component | Domain 19 |
| emnc.Hy | -Vmy | A/m | Magnetic field, y component | Domain 19 |
| emnc.Hz | -Vmz | A/m | Magnetic field, z component | Domain 19 |
| emnc.tHx | -VmTx | A/m | Tangential magnetic field, x component | Boundaries 109–112, 114, 116 |
| emnc.tHy | -VmTy | A/m | Tangential magnetic field, y component | Boundaries 109–112, 114, 116 |
| emnc.tHz | -VmTz | A/m | Tangential magnetic field, z component | Boundaries 109–112, 114, 116 |
| emnc.normH | sqrt(realdot(emnc.Hx,emnc.Hx)+realdot(emnc.Hy,emnc.Hy)+realdot(emnc.Hz,emnc.Hz)) | A/m | Magnetic field norm | Domain 19 |
| emnc.murxx | model.input.mur11 | 1 | Relative permeability, xx component | Domain 19 |
| emnc.muryx | model.input.mur21 | 1 | Relative permeability, yx component | Domain 19 |
| emnc.murzx | model.input.mur31 | 1 | Relative permeability, zx component | Domain 19 |
| emnc.murxy | model.input.mur12 | 1 | Relative permeability, xy component | Domain 19 |
| emnc.muryy | model.input.mur22 | 1 | Relative permeability, yy component | Domain 19 |
| emnc.murzy | model.input.mur32 | 1 | Relative permeability, zy component | Domain 19 |
| emnc.murxz | model.input.mur13 | 1 | Relative permeability, xz component | Domain 19 |
| emnc.muryz | model.input.mur23 | 1 | Relative permeability, yz component | Domain 19 |
| emnc.murzz | model.input.mur33 | 1 | Relative permeability, zz component | Domain 19 |
| emnc.Bx | mu0\_const\*emnc.Ixx\*emnc.Hx+mu0\_const\*emnc.Ixy\*emnc.Hy+mu0\_const\*emnc.Ixz\*emnc.Hz+mu0\_const\*emnc.chimxx\*emnc.Hx+mu0\_const\*emnc.chimxy\*emnc.Hy+mu0\_const\*emnc.chimxz\*emnc.Hz+emnc.Brx | T | Magnetic flux density, x component | Domain 19 |
| emnc.By | mu0\_const\*emnc.Iyx\*emnc.Hx+mu0\_const\*emnc.Iyy\*emnc.Hy+mu0\_const\*emnc.Iyz\*emnc.Hz+mu0\_const\*emnc.chimyx\*emnc.Hx+mu0\_const\*emnc.chimyy\*emnc.Hy+mu0\_const\*emnc.chimyz\*emnc.Hz+emnc.Bry | T | Magnetic flux density, y component | Domain 19 |
| emnc.Bz | mu0\_const\*emnc.Izx\*emnc.Hx+mu0\_const\*emnc.Izy\*emnc.Hy+mu0\_const\*emnc.Izz\*emnc.Hz+mu0\_const\*emnc.chimzx\*emnc.Hx+mu0\_const\*emnc.chimzy\*emnc.Hy+mu0\_const\*emnc.chimzz\*emnc.Hz+emnc.Brz | T | Magnetic flux density, z component | Domain 19 |
| emnc.normB | sqrt(realdot(emnc.Bx,emnc.Bx)+realdot(emnc.By,emnc.By)+realdot(emnc.Bz,emnc.Bz)) | T | Magnetic flux density norm | Domain 19 |
| emnc.Mx | emnc.Bx/mu0\_const-emnc.Ixx\*emnc.Hx-emnc.Ixy\*emnc.Hy-emnc.Ixz\*emnc.Hz | A/m | Magnetization, x component | Domain 19 |
| emnc.My | emnc.By/mu0\_const-emnc.Iyx\*emnc.Hx-emnc.Iyy\*emnc.Hy-emnc.Iyz\*emnc.Hz | A/m | Magnetization, y component | Domain 19 |
| emnc.Mz | emnc.Bz/mu0\_const-emnc.Izx\*emnc.Hx-emnc.Izy\*emnc.Hy-emnc.Izz\*emnc.Hz | A/m | Magnetization, z component | Domain 19 |
| emnc.normM | sqrt(realdot(emnc.Mx,emnc.Mx)+realdot(emnc.My,emnc.My)+realdot(emnc.Mz,emnc.Mz)) | A/m | Magnetization norm | Domain 19 |
| emnc.Ixx | 1 | 1 | Spatial identity matrix, xx component | Domain 19 |
| emnc.Iyx | 0 | 1 | Spatial identity matrix, yx component | Domain 19 |
| emnc.Izx | 0 | 1 | Spatial identity matrix, zx component | Domain 19 |
| emnc.Ixy | 0 | 1 | Spatial identity matrix, xy component | Domain 19 |
| emnc.Iyy | 1 | 1 | Spatial identity matrix, yy component | Domain 19 |
| emnc.Izy | 0 | 1 | Spatial identity matrix, zy component | Domain 19 |
| emnc.Ixz | 0 | 1 | Spatial identity matrix, xz component | Domain 19 |
| emnc.Iyz | 0 | 1 | Spatial identity matrix, yz component | Domain 19 |
| emnc.Izz | 1 | 1 | Spatial identity matrix, zz component | Domain 19 |
| emnc.Brx | BpRem\*cos(4\*argBp+tick) | T | Remanent flux density, x component | Domain 19 |
| emnc.Bry | BpRem\*sin(4\*argBp+tick) | T | Remanent flux density, y component | Domain 19 |
| emnc.Brz | 0 | T | Remanent flux density, z component | Domain 19 |
| emnc.normBr | sqrt(realdot(emnc.Brx,emnc.Brx)+realdot(emnc.Bry,emnc.Bry)+realdot(emnc.Brz,emnc.Brz)) | T | Remanent flux density norm | Domain 19 |
| emnc.chimxx | -1+emnc.murxx | 1 | Magnetic susceptibility, xx component | Domain 19 |
| emnc.chimyx | emnc.muryx | 1 | Magnetic susceptibility, yx component | Domain 19 |
| emnc.chimzx | emnc.murzx | 1 | Magnetic susceptibility, zx component | Domain 19 |
| emnc.chimxy | emnc.murxy | 1 | Magnetic susceptibility, xy component | Domain 19 |
| emnc.chimyy | -1+emnc.muryy | 1 | Magnetic susceptibility, yy component | Domain 19 |
| emnc.chimzy | emnc.murzy | 1 | Magnetic susceptibility, zy component | Domain 19 |
| emnc.chimxz | emnc.murxz | 1 | Magnetic susceptibility, xz component | Domain 19 |
| emnc.chimyz | emnc.muryz | 1 | Magnetic susceptibility, yz component | Domain 19 |
| emnc.chimzz | -1+emnc.murzz | 1 | Magnetic susceptibility, zz component | Domain 19 |
| emnc.unTx | 0 | Pa | Maxwell upward surface stress tensor, x component | Boundaries 109–112, 114, 116 |
| emnc.unTy | 0 | Pa | Maxwell upward surface stress tensor, y component | Boundaries 109–112, 114, 116 |
| emnc.unTz | 0 | Pa | Maxwell upward surface stress tensor, z component | Boundaries 109–112, 114, 116 |
| emnc.dnTx | emnc.dnTmx | Pa | Maxwell downward surface stress tensor, x component | Boundaries 109–112, 114, 116 |
| emnc.dnTy | emnc.dnTmy | Pa | Maxwell downward surface stress tensor, y component | Boundaries 109–112, 114, 116 |
| emnc.dnTz | emnc.dnTmz | Pa | Maxwell downward surface stress tensor, z component | Boundaries 109–112, 114, 116 |
| emnc.unx | unx |  | Normal vector up direction, x component | Boundaries 109–112, 114, 116 |
| emnc.uny | uny |  | Normal vector up direction, y component | Boundaries 109–112, 114, 116 |
| emnc.unz | unz |  | Normal vector up direction, z component | Boundaries 109–112, 114, 116 |
| emnc.dnx | dnx |  | Normal vector down direction, x component | Boundaries 109–112, 114, 116 |
| emnc.dny | dny |  | Normal vector down direction, y component | Boundaries 109–112, 114, 116 |
| emnc.dnz | dnz |  | Normal vector down direction, z component | Boundaries 109–112, 114, 116 |
| emnc.W | emnc.Wm | J/m^3 | Energy density | Domain 19 |
| emnc.dWm | emnc.Wm | J/m^3 | Integrand for total magnetic energy | Domain 19 |
| emnc.Wm | 0.5\*mu0\_const\*((emnc.murxx\*emnc.Hx+emnc.murxy\*emnc.Hy+emnc.murxz\*emnc.Hz)\*emnc.Hx+(emnc.muryx\*emnc.Hx+emnc.muryy\*emnc.Hy+emnc.muryz\*emnc.Hz)\*emnc.Hy+(emnc.murzx\*emnc.Hx+emnc.murzy\*emnc.Hy+emnc.murzz\*emnc.Hz)\*emnc.Hz) | J/m^3 | Magnetic energy density | Domain 19 |

##### Shape Functions

| Name | Shape function | Unit | Description | Shape frame | Selection |
| --- | --- | --- | --- | --- | --- |
| Vm | Lagrange (Quadratic) | A | Magnetic scalar potential | Material | Domain 19 |

##### Weak Expressions

| Weak expression | Integration frame | Selection |
| --- | --- | --- |
| emnc.d\*(-emnc.Bx\*test(Vmx)-emnc.By\*test(Vmy)-emnc.Bz\*test(Vmz)) | Material | Domain 19 |

#### 2.4.9. Magnetic Flux Conservation 7

Magnetic Flux Conservation 7

Selection

| Geometric entity level | Domain |
| Selection | Domain 17 |

Equations

Settings

| Description | Value |
| Constitutive relation | Remanent flux density |
| Remanent flux density, x component | BpRem\*cos(argBp\*(-1 + 6) + tick) |
| Remanent flux density, y component | BpRem\*sin(argBp\*(-1 + 6) + tick) |
| Remanent flux density, z component | 0 |
| Relative permeability | From material |
| Relative permeability | {{1, 0, 0}, {0, 1, 0}, {0, 0, 1}} |

Properties from material

| Property | Material | Property group |
| Relative permeability | Soft Iron (without losses) | Basic |

##### Variables

| Name | Expression | Unit | Description | Selection |
| --- | --- | --- | --- | --- |
| emnc.dnTmx | -0.5\*emnc.unx\*(real(down(emnc.Bx))\*real(down(emnc.Hx))+real(down(emnc.By))\*real(down(emnc.Hy))+real(down(emnc.Bz))\*real(down(emnc.Hz)))+real(down(emnc.Bx))\*(real(down(emnc.Hx))\*emnc.unx+real(down(emnc.Hy))\*emnc.uny+real(down(emnc.Hz))\*emnc.unz) | Pa | Maxwell downward magnetic surface stress tensor, x component | Boundaries 97–100, 102, 104 |
| emnc.dnTmy | -0.5\*emnc.uny\*(real(down(emnc.Bx))\*real(down(emnc.Hx))+real(down(emnc.By))\*real(down(emnc.Hy))+real(down(emnc.Bz))\*real(down(emnc.Hz)))+real(down(emnc.By))\*(real(down(emnc.Hx))\*emnc.unx+real(down(emnc.Hy))\*emnc.uny+real(down(emnc.Hz))\*emnc.unz) | Pa | Maxwell downward magnetic surface stress tensor, y component | Boundaries 97–100, 102, 104 |
| emnc.dnTmz | -0.5\*emnc.unz\*(real(down(emnc.Bx))\*real(down(emnc.Hx))+real(down(emnc.By))\*real(down(emnc.Hy))+real(down(emnc.Bz))\*real(down(emnc.Hz)))+real(down(emnc.Bz))\*(real(down(emnc.Hx))\*emnc.unx+real(down(emnc.Hy))\*emnc.uny+real(down(emnc.Hz))\*emnc.unz) | Pa | Maxwell downward magnetic surface stress tensor, z component | Boundaries 97–100, 102, 104 |
| emnc.Hx | -Vmx | A/m | Magnetic field, x component | Domain 17 |
| emnc.Hy | -Vmy | A/m | Magnetic field, y component | Domain 17 |
| emnc.Hz | -Vmz | A/m | Magnetic field, z component | Domain 17 |
| emnc.tHx | -VmTx | A/m | Tangential magnetic field, x component | Boundaries 97–100, 102, 104 |
| emnc.tHy | -VmTy | A/m | Tangential magnetic field, y component | Boundaries 97–100, 102, 104 |
| emnc.tHz | -VmTz | A/m | Tangential magnetic field, z component | Boundaries 97–100, 102, 104 |
| emnc.normH | sqrt(realdot(emnc.Hx,emnc.Hx)+realdot(emnc.Hy,emnc.Hy)+realdot(emnc.Hz,emnc.Hz)) | A/m | Magnetic field norm | Domain 17 |
| emnc.murxx | model.input.mur11 | 1 | Relative permeability, xx component | Domain 17 |
| emnc.muryx | model.input.mur21 | 1 | Relative permeability, yx component | Domain 17 |
| emnc.murzx | model.input.mur31 | 1 | Relative permeability, zx component | Domain 17 |
| emnc.murxy | model.input.mur12 | 1 | Relative permeability, xy component | Domain 17 |
| emnc.muryy | model.input.mur22 | 1 | Relative permeability, yy component | Domain 17 |
| emnc.murzy | model.input.mur32 | 1 | Relative permeability, zy component | Domain 17 |
| emnc.murxz | model.input.mur13 | 1 | Relative permeability, xz component | Domain 17 |
| emnc.muryz | model.input.mur23 | 1 | Relative permeability, yz component | Domain 17 |
| emnc.murzz | model.input.mur33 | 1 | Relative permeability, zz component | Domain 17 |
| emnc.Bx | mu0\_const\*emnc.Ixx\*emnc.Hx+mu0\_const\*emnc.Ixy\*emnc.Hy+mu0\_const\*emnc.Ixz\*emnc.Hz+mu0\_const\*emnc.chimxx\*emnc.Hx+mu0\_const\*emnc.chimxy\*emnc.Hy+mu0\_const\*emnc.chimxz\*emnc.Hz+emnc.Brx | T | Magnetic flux density, x component | Domain 17 |
| emnc.By | mu0\_const\*emnc.Iyx\*emnc.Hx+mu0\_const\*emnc.Iyy\*emnc.Hy+mu0\_const\*emnc.Iyz\*emnc.Hz+mu0\_const\*emnc.chimyx\*emnc.Hx+mu0\_const\*emnc.chimyy\*emnc.Hy+mu0\_const\*emnc.chimyz\*emnc.Hz+emnc.Bry | T | Magnetic flux density, y component | Domain 17 |
| emnc.Bz | mu0\_const\*emnc.Izx\*emnc.Hx+mu0\_const\*emnc.Izy\*emnc.Hy+mu0\_const\*emnc.Izz\*emnc.Hz+mu0\_const\*emnc.chimzx\*emnc.Hx+mu0\_const\*emnc.chimzy\*emnc.Hy+mu0\_const\*emnc.chimzz\*emnc.Hz+emnc.Brz | T | Magnetic flux density, z component | Domain 17 |
| emnc.normB | sqrt(realdot(emnc.Bx,emnc.Bx)+realdot(emnc.By,emnc.By)+realdot(emnc.Bz,emnc.Bz)) | T | Magnetic flux density norm | Domain 17 |
| emnc.Mx | emnc.Bx/mu0\_const-emnc.Ixx\*emnc.Hx-emnc.Ixy\*emnc.Hy-emnc.Ixz\*emnc.Hz | A/m | Magnetization, x component | Domain 17 |
| emnc.My | emnc.By/mu0\_const-emnc.Iyx\*emnc.Hx-emnc.Iyy\*emnc.Hy-emnc.Iyz\*emnc.Hz | A/m | Magnetization, y component | Domain 17 |
| emnc.Mz | emnc.Bz/mu0\_const-emnc.Izx\*emnc.Hx-emnc.Izy\*emnc.Hy-emnc.Izz\*emnc.Hz | A/m | Magnetization, z component | Domain 17 |
| emnc.normM | sqrt(realdot(emnc.Mx,emnc.Mx)+realdot(emnc.My,emnc.My)+realdot(emnc.Mz,emnc.Mz)) | A/m | Magnetization norm | Domain 17 |
| emnc.Ixx | 1 | 1 | Spatial identity matrix, xx component | Domain 17 |
| emnc.Iyx | 0 | 1 | Spatial identity matrix, yx component | Domain 17 |
| emnc.Izx | 0 | 1 | Spatial identity matrix, zx component | Domain 17 |
| emnc.Ixy | 0 | 1 | Spatial identity matrix, xy component | Domain 17 |
| emnc.Iyy | 1 | 1 | Spatial identity matrix, yy component | Domain 17 |
| emnc.Izy | 0 | 1 | Spatial identity matrix, zy component | Domain 17 |
| emnc.Ixz | 0 | 1 | Spatial identity matrix, xz component | Domain 17 |
| emnc.Iyz | 0 | 1 | Spatial identity matrix, yz component | Domain 17 |
| emnc.Izz | 1 | 1 | Spatial identity matrix, zz component | Domain 17 |
| emnc.Brx | BpRem\*cos(5\*argBp+tick) | T | Remanent flux density, x component | Domain 17 |
| emnc.Bry | BpRem\*sin(5\*argBp+tick) | T | Remanent flux density, y component | Domain 17 |
| emnc.Brz | 0 | T | Remanent flux density, z component | Domain 17 |
| emnc.normBr | sqrt(realdot(emnc.Brx,emnc.Brx)+realdot(emnc.Bry,emnc.Bry)+realdot(emnc.Brz,emnc.Brz)) | T | Remanent flux density norm | Domain 17 |
| emnc.chimxx | -1+emnc.murxx | 1 | Magnetic susceptibility, xx component | Domain 17 |
| emnc.chimyx | emnc.muryx | 1 | Magnetic susceptibility, yx component | Domain 17 |
| emnc.chimzx | emnc.murzx | 1 | Magnetic susceptibility, zx component | Domain 17 |
| emnc.chimxy | emnc.murxy | 1 | Magnetic susceptibility, xy component | Domain 17 |
| emnc.chimyy | -1+emnc.muryy | 1 | Magnetic susceptibility, yy component | Domain 17 |
| emnc.chimzy | emnc.murzy | 1 | Magnetic susceptibility, zy component | Domain 17 |
| emnc.chimxz | emnc.murxz | 1 | Magnetic susceptibility, xz component | Domain 17 |
| emnc.chimyz | emnc.muryz | 1 | Magnetic susceptibility, yz component | Domain 17 |
| emnc.chimzz | -1+emnc.murzz | 1 | Magnetic susceptibility, zz component | Domain 17 |
| emnc.unTx | 0 | Pa | Maxwell upward surface stress tensor, x component | Boundaries 97–100, 102, 104 |
| emnc.unTy | 0 | Pa | Maxwell upward surface stress tensor, y component | Boundaries 97–100, 102, 104 |
| emnc.unTz | 0 | Pa | Maxwell upward surface stress tensor, z component | Boundaries 97–100, 102, 104 |
| emnc.dnTx | emnc.dnTmx | Pa | Maxwell downward surface stress tensor, x component | Boundaries 97–100, 102, 104 |
| emnc.dnTy | emnc.dnTmy | Pa | Maxwell downward surface stress tensor, y component | Boundaries 97–100, 102, 104 |
| emnc.dnTz | emnc.dnTmz | Pa | Maxwell downward surface stress tensor, z component | Boundaries 97–100, 102, 104 |
| emnc.unx | unx |  | Normal vector up direction, x component | Boundaries 97–100, 102, 104 |
| emnc.uny | uny |  | Normal vector up direction, y component | Boundaries 97–100, 102, 104 |
| emnc.unz | unz |  | Normal vector up direction, z component | Boundaries 97–100, 102, 104 |
| emnc.dnx | dnx |  | Normal vector down direction, x component | Boundaries 97–100, 102, 104 |
| emnc.dny | dny |  | Normal vector down direction, y component | Boundaries 97–100, 102, 104 |
| emnc.dnz | dnz |  | Normal vector down direction, z component | Boundaries 97–100, 102, 104 |
| emnc.W | emnc.Wm | J/m^3 | Energy density | Domain 17 |
| emnc.dWm | emnc.Wm | J/m^3 | Integrand for total magnetic energy | Domain 17 |
| emnc.Wm | 0.5\*mu0\_const\*((emnc.murxx\*emnc.Hx+emnc.murxy\*emnc.Hy+emnc.murxz\*emnc.Hz)\*emnc.Hx+(emnc.muryx\*emnc.Hx+emnc.muryy\*emnc.Hy+emnc.muryz\*emnc.Hz)\*emnc.Hy+(emnc.murzx\*emnc.Hx+emnc.murzy\*emnc.Hy+emnc.murzz\*emnc.Hz)\*emnc.Hz) | J/m^3 | Magnetic energy density | Domain 17 |

##### Shape Functions

| Name | Shape function | Unit | Description | Shape frame | Selection |
| --- | --- | --- | --- | --- | --- |
| Vm | Lagrange (Quadratic) | A | Magnetic scalar potential | Material | Domain 17 |

##### Weak Expressions

| Weak expression | Integration frame | Selection |
| --- | --- | --- |
| emnc.d\*(-emnc.Bx\*test(Vmx)-emnc.By\*test(Vmy)-emnc.Bz\*test(Vmz)) | Material | Domain 17 |

#### 2.4.10. Magnetic Flux Conservation 8

Magnetic Flux Conservation 8

Selection

| Geometric entity level | Domain |
| Selection | Domain 15 |

Equations

Settings

| Description | Value |
| Constitutive relation | Remanent flux density |
| Remanent flux density, x component | BpRem\*cos(argBp\*(-1 + 7) + tick) |
| Remanent flux density, y component | BpRem\*sin(argBp\*(-1 + 7) + tick) |
| Remanent flux density, z component | 0 |
| Relative permeability | From material |
| Relative permeability | {{1, 0, 0}, {0, 1, 0}, {0, 0, 1}} |

Properties from material

| Property | Material | Property group |
| Relative permeability | Soft Iron (without losses) | Basic |

##### Variables

| Name | Expression | Unit | Description | Selection |
| --- | --- | --- | --- | --- |
| emnc.dnTmx | -0.5\*emnc.unx\*(real(down(emnc.Bx))\*real(down(emnc.Hx))+real(down(emnc.By))\*real(down(emnc.Hy))+real(down(emnc.Bz))\*real(down(emnc.Hz)))+real(down(emnc.Bx))\*(real(down(emnc.Hx))\*emnc.unx+real(down(emnc.Hy))\*emnc.uny+real(down(emnc.Hz))\*emnc.unz) | Pa | Maxwell downward magnetic surface stress tensor, x component | Boundaries 79–82, 90–91 |
| emnc.dnTmy | -0.5\*emnc.uny\*(real(down(emnc.Bx))\*real(down(emnc.Hx))+real(down(emnc.By))\*real(down(emnc.Hy))+real(down(emnc.Bz))\*real(down(emnc.Hz)))+real(down(emnc.By))\*(real(down(emnc.Hx))\*emnc.unx+real(down(emnc.Hy))\*emnc.uny+real(down(emnc.Hz))\*emnc.unz) | Pa | Maxwell downward magnetic surface stress tensor, y component | Boundaries 79–82, 90–91 |
| emnc.dnTmz | -0.5\*emnc.unz\*(real(down(emnc.Bx))\*real(down(emnc.Hx))+real(down(emnc.By))\*real(down(emnc.Hy))+real(down(emnc.Bz))\*real(down(emnc.Hz)))+real(down(emnc.Bz))\*(real(down(emnc.Hx))\*emnc.unx+real(down(emnc.Hy))\*emnc.uny+real(down(emnc.Hz))\*emnc.unz) | Pa | Maxwell downward magnetic surface stress tensor, z component | Boundaries 79–82, 90–91 |
| emnc.Hx | -Vmx | A/m | Magnetic field, x component | Domain 15 |
| emnc.Hy | -Vmy | A/m | Magnetic field, y component | Domain 15 |
| emnc.Hz | -Vmz | A/m | Magnetic field, z component | Domain 15 |
| emnc.tHx | -VmTx | A/m | Tangential magnetic field, x component | Boundaries 79–82, 90–91 |
| emnc.tHy | -VmTy | A/m | Tangential magnetic field, y component | Boundaries 79–82, 90–91 |
| emnc.tHz | -VmTz | A/m | Tangential magnetic field, z component | Boundaries 79–82, 90–91 |
| emnc.normH | sqrt(realdot(emnc.Hx,emnc.Hx)+realdot(emnc.Hy,emnc.Hy)+realdot(emnc.Hz,emnc.Hz)) | A/m | Magnetic field norm | Domain 15 |
| emnc.murxx | model.input.mur11 | 1 | Relative permeability, xx component | Domain 15 |
| emnc.muryx | model.input.mur21 | 1 | Relative permeability, yx component | Domain 15 |
| emnc.murzx | model.input.mur31 | 1 | Relative permeability, zx component | Domain 15 |
| emnc.murxy | model.input.mur12 | 1 | Relative permeability, xy component | Domain 15 |
| emnc.muryy | model.input.mur22 | 1 | Relative permeability, yy component | Domain 15 |
| emnc.murzy | model.input.mur32 | 1 | Relative permeability, zy component | Domain 15 |
| emnc.murxz | model.input.mur13 | 1 | Relative permeability, xz component | Domain 15 |
| emnc.muryz | model.input.mur23 | 1 | Relative permeability, yz component | Domain 15 |
| emnc.murzz | model.input.mur33 | 1 | Relative permeability, zz component | Domain 15 |
| emnc.Bx | mu0\_const\*emnc.Ixx\*emnc.Hx+mu0\_const\*emnc.Ixy\*emnc.Hy+mu0\_const\*emnc.Ixz\*emnc.Hz+mu0\_const\*emnc.chimxx\*emnc.Hx+mu0\_const\*emnc.chimxy\*emnc.Hy+mu0\_const\*emnc.chimxz\*emnc.Hz+emnc.Brx | T | Magnetic flux density, x component | Domain 15 |
| emnc.By | mu0\_const\*emnc.Iyx\*emnc.Hx+mu0\_const\*emnc.Iyy\*emnc.Hy+mu0\_const\*emnc.Iyz\*emnc.Hz+mu0\_const\*emnc.chimyx\*emnc.Hx+mu0\_const\*emnc.chimyy\*emnc.Hy+mu0\_const\*emnc.chimyz\*emnc.Hz+emnc.Bry | T | Magnetic flux density, y component | Domain 15 |
| emnc.Bz | mu0\_const\*emnc.Izx\*emnc.Hx+mu0\_const\*emnc.Izy\*emnc.Hy+mu0\_const\*emnc.Izz\*emnc.Hz+mu0\_const\*emnc.chimzx\*emnc.Hx+mu0\_const\*emnc.chimzy\*emnc.Hy+mu0\_const\*emnc.chimzz\*emnc.Hz+emnc.Brz | T | Magnetic flux density, z component | Domain 15 |
| emnc.normB | sqrt(realdot(emnc.Bx,emnc.Bx)+realdot(emnc.By,emnc.By)+realdot(emnc.Bz,emnc.Bz)) | T | Magnetic flux density norm | Domain 15 |
| emnc.Mx | emnc.Bx/mu0\_const-emnc.Ixx\*emnc.Hx-emnc.Ixy\*emnc.Hy-emnc.Ixz\*emnc.Hz | A/m | Magnetization, x component | Domain 15 |
| emnc.My | emnc.By/mu0\_const-emnc.Iyx\*emnc.Hx-emnc.Iyy\*emnc.Hy-emnc.Iyz\*emnc.Hz | A/m | Magnetization, y component | Domain 15 |
| emnc.Mz | emnc.Bz/mu0\_const-emnc.Izx\*emnc.Hx-emnc.Izy\*emnc.Hy-emnc.Izz\*emnc.Hz | A/m | Magnetization, z component | Domain 15 |
| emnc.normM | sqrt(realdot(emnc.Mx,emnc.Mx)+realdot(emnc.My,emnc.My)+realdot(emnc.Mz,emnc.Mz)) | A/m | Magnetization norm | Domain 15 |
| emnc.Ixx | 1 | 1 | Spatial identity matrix, xx component | Domain 15 |
| emnc.Iyx | 0 | 1 | Spatial identity matrix, yx component | Domain 15 |
| emnc.Izx | 0 | 1 | Spatial identity matrix, zx component | Domain 15 |
| emnc.Ixy | 0 | 1 | Spatial identity matrix, xy component | Domain 15 |
| emnc.Iyy | 1 | 1 | Spatial identity matrix, yy component | Domain 15 |
| emnc.Izy | 0 | 1 | Spatial identity matrix, zy component | Domain 15 |
| emnc.Ixz | 0 | 1 | Spatial identity matrix, xz component | Domain 15 |
| emnc.Iyz | 0 | 1 | Spatial identity matrix, yz component | Domain 15 |
| emnc.Izz | 1 | 1 | Spatial identity matrix, zz component | Domain 15 |
| emnc.Brx | BpRem\*cos(6\*argBp+tick) | T | Remanent flux density, x component | Domain 15 |
| emnc.Bry | BpRem\*sin(6\*argBp+tick) | T | Remanent flux density, y component | Domain 15 |
| emnc.Brz | 0 | T | Remanent flux density, z component | Domain 15 |
| emnc.normBr | sqrt(realdot(emnc.Brx,emnc.Brx)+realdot(emnc.Bry,emnc.Bry)+realdot(emnc.Brz,emnc.Brz)) | T | Remanent flux density norm | Domain 15 |
| emnc.chimxx | -1+emnc.murxx | 1 | Magnetic susceptibility, xx component | Domain 15 |
| emnc.chimyx | emnc.muryx | 1 | Magnetic susceptibility, yx component | Domain 15 |
| emnc.chimzx | emnc.murzx | 1 | Magnetic susceptibility, zx component | Domain 15 |
| emnc.chimxy | emnc.murxy | 1 | Magnetic susceptibility, xy component | Domain 15 |
| emnc.chimyy | -1+emnc.muryy | 1 | Magnetic susceptibility, yy component | Domain 15 |
| emnc.chimzy | emnc.murzy | 1 | Magnetic susceptibility, zy component | Domain 15 |
| emnc.chimxz | emnc.murxz | 1 | Magnetic susceptibility, xz component | Domain 15 |
| emnc.chimyz | emnc.muryz | 1 | Magnetic susceptibility, yz component | Domain 15 |
| emnc.chimzz | -1+emnc.murzz | 1 | Magnetic susceptibility, zz component | Domain 15 |
| emnc.unTx | 0 | Pa | Maxwell upward surface stress tensor, x component | Boundaries 79–82, 90–91 |
| emnc.unTy | 0 | Pa | Maxwell upward surface stress tensor, y component | Boundaries 79–82, 90–91 |
| emnc.unTz | 0 | Pa | Maxwell upward surface stress tensor, z component | Boundaries 79–82, 90–91 |
| emnc.dnTx | emnc.dnTmx | Pa | Maxwell downward surface stress tensor, x component | Boundaries 79–82, 90–91 |
| emnc.dnTy | emnc.dnTmy | Pa | Maxwell downward surface stress tensor, y component | Boundaries 79–82, 90–91 |
| emnc.dnTz | emnc.dnTmz | Pa | Maxwell downward surface stress tensor, z component | Boundaries 79–82, 90–91 |
| emnc.unx | unx |  | Normal vector up direction, x component | Boundaries 79–82, 90–91 |
| emnc.uny | uny |  | Normal vector up direction, y component | Boundaries 79–82, 90–91 |
| emnc.unz | unz |  | Normal vector up direction, z component | Boundaries 79–82, 90–91 |
| emnc.dnx | dnx |  | Normal vector down direction, x component | Boundaries 79–82, 90–91 |
| emnc.dny | dny |  | Normal vector down direction, y component | Boundaries 79–82, 90–91 |
| emnc.dnz | dnz |  | Normal vector down direction, z component | Boundaries 79–82, 90–91 |
| emnc.W | emnc.Wm | J/m^3 | Energy density | Domain 15 |
| emnc.dWm | emnc.Wm | J/m^3 | Integrand for total magnetic energy | Domain 15 |
| emnc.Wm | 0.5\*mu0\_const\*((emnc.murxx\*emnc.Hx+emnc.murxy\*emnc.Hy+emnc.murxz\*emnc.Hz)\*emnc.Hx+(emnc.muryx\*emnc.Hx+emnc.muryy\*emnc.Hy+emnc.muryz\*emnc.Hz)\*emnc.Hy+(emnc.murzx\*emnc.Hx+emnc.murzy\*emnc.Hy+emnc.murzz\*emnc.Hz)\*emnc.Hz) | J/m^3 | Magnetic energy density | Domain 15 |

##### Shape Functions

| Name | Shape function | Unit | Description | Shape frame | Selection |
| --- | --- | --- | --- | --- | --- |
| Vm | Lagrange (Quadratic) | A | Magnetic scalar potential | Material | Domain 15 |

##### Weak Expressions

| Weak expression | Integration frame | Selection |
| --- | --- | --- |
| emnc.d\*(-emnc.Bx\*test(Vmx)-emnc.By\*test(Vmy)-emnc.Bz\*test(Vmz)) | Material | Domain 15 |

#### 2.4.11. Magnetic Flux Conservation 9

Magnetic Flux Conservation 9

Selection

| Geometric entity level | Domain |
| Selection | Domain 12 |

Equations

Settings

| Description | Value |
| Constitutive relation | Remanent flux density |
| Remanent flux density, x component | BpRem\*cos(argBp\*(-1 + 8) + tick) |
| Remanent flux density, y component | BpRem\*sin(argBp\*(-1 + 8) + tick) |
| Remanent flux density, z component | 0 |
| Relative permeability | From material |
| Relative permeability | {{1, 0, 0}, {0, 1, 0}, {0, 0, 1}} |

Properties from material

| Property | Material | Property group |
| Relative permeability | Soft Iron (without losses) | Basic |

##### Variables

| Name | Expression | Unit | Description | Selection |
| --- | --- | --- | --- | --- |
| emnc.dnTmx | -0.5\*emnc.unx\*(real(down(emnc.Bx))\*real(down(emnc.Hx))+real(down(emnc.By))\*real(down(emnc.Hy))+real(down(emnc.Bz))\*real(down(emnc.Hz)))+real(down(emnc.Bx))\*(real(down(emnc.Hx))\*emnc.unx+real(down(emnc.Hy))\*emnc.uny+real(down(emnc.Hz))\*emnc.unz) | Pa | Maxwell downward magnetic surface stress tensor, x component | Boundaries 63–66, 68, 70 |
| emnc.dnTmy | -0.5\*emnc.uny\*(real(down(emnc.Bx))\*real(down(emnc.Hx))+real(down(emnc.By))\*real(down(emnc.Hy))+real(down(emnc.Bz))\*real(down(emnc.Hz)))+real(down(emnc.By))\*(real(down(emnc.Hx))\*emnc.unx+real(down(emnc.Hy))\*emnc.uny+real(down(emnc.Hz))\*emnc.unz) | Pa | Maxwell downward magnetic surface stress tensor, y component | Boundaries 63–66, 68, 70 |
| emnc.dnTmz | -0.5\*emnc.unz\*(real(down(emnc.Bx))\*real(down(emnc.Hx))+real(down(emnc.By))\*real(down(emnc.Hy))+real(down(emnc.Bz))\*real(down(emnc.Hz)))+real(down(emnc.Bz))\*(real(down(emnc.Hx))\*emnc.unx+real(down(emnc.Hy))\*emnc.uny+real(down(emnc.Hz))\*emnc.unz) | Pa | Maxwell downward magnetic surface stress tensor, z component | Boundaries 63–66, 68, 70 |
| emnc.Hx | -Vmx | A/m | Magnetic field, x component | Domain 12 |
| emnc.Hy | -Vmy | A/m | Magnetic field, y component | Domain 12 |
| emnc.Hz | -Vmz | A/m | Magnetic field, z component | Domain 12 |
| emnc.tHx | -VmTx | A/m | Tangential magnetic field, x component | Boundaries 63–66, 68, 70 |
| emnc.tHy | -VmTy | A/m | Tangential magnetic field, y component | Boundaries 63–66, 68, 70 |
| emnc.tHz | -VmTz | A/m | Tangential magnetic field, z component | Boundaries 63–66, 68, 70 |
| emnc.normH | sqrt(realdot(emnc.Hx,emnc.Hx)+realdot(emnc.Hy,emnc.Hy)+realdot(emnc.Hz,emnc.Hz)) | A/m | Magnetic field norm | Domain 12 |
| emnc.murxx | model.input.mur11 | 1 | Relative permeability, xx component | Domain 12 |
| emnc.muryx | model.input.mur21 | 1 | Relative permeability, yx component | Domain 12 |
| emnc.murzx | model.input.mur31 | 1 | Relative permeability, zx component | Domain 12 |
| emnc.murxy | model.input.mur12 | 1 | Relative permeability, xy component | Domain 12 |
| emnc.muryy | model.input.mur22 | 1 | Relative permeability, yy component | Domain 12 |
| emnc.murzy | model.input.mur32 | 1 | Relative permeability, zy component | Domain 12 |
| emnc.murxz | model.input.mur13 | 1 | Relative permeability, xz component | Domain 12 |
| emnc.muryz | model.input.mur23 | 1 | Relative permeability, yz component | Domain 12 |
| emnc.murzz | model.input.mur33 | 1 | Relative permeability, zz component | Domain 12 |
| emnc.Bx | mu0\_const\*emnc.Ixx\*emnc.Hx+mu0\_const\*emnc.Ixy\*emnc.Hy+mu0\_const\*emnc.Ixz\*emnc.Hz+mu0\_const\*emnc.chimxx\*emnc.Hx+mu0\_const\*emnc.chimxy\*emnc.Hy+mu0\_const\*emnc.chimxz\*emnc.Hz+emnc.Brx | T | Magnetic flux density, x component | Domain 12 |
| emnc.By | mu0\_const\*emnc.Iyx\*emnc.Hx+mu0\_const\*emnc.Iyy\*emnc.Hy+mu0\_const\*emnc.Iyz\*emnc.Hz+mu0\_const\*emnc.chimyx\*emnc.Hx+mu0\_const\*emnc.chimyy\*emnc.Hy+mu0\_const\*emnc.chimyz\*emnc.Hz+emnc.Bry | T | Magnetic flux density, y component | Domain 12 |
| emnc.Bz | mu0\_const\*emnc.Izx\*emnc.Hx+mu0\_const\*emnc.Izy\*emnc.Hy+mu0\_const\*emnc.Izz\*emnc.Hz+mu0\_const\*emnc.chimzx\*emnc.Hx+mu0\_const\*emnc.chimzy\*emnc.Hy+mu0\_const\*emnc.chimzz\*emnc.Hz+emnc.Brz | T | Magnetic flux density, z component | Domain 12 |
| emnc.normB | sqrt(realdot(emnc.Bx,emnc.Bx)+realdot(emnc.By,emnc.By)+realdot(emnc.Bz,emnc.Bz)) | T | Magnetic flux density norm | Domain 12 |
| emnc.Mx | emnc.Bx/mu0\_const-emnc.Ixx\*emnc.Hx-emnc.Ixy\*emnc.Hy-emnc.Ixz\*emnc.Hz | A/m | Magnetization, x component | Domain 12 |
| emnc.My | emnc.By/mu0\_const-emnc.Iyx\*emnc.Hx-emnc.Iyy\*emnc.Hy-emnc.Iyz\*emnc.Hz | A/m | Magnetization, y component | Domain 12 |
| emnc.Mz | emnc.Bz/mu0\_const-emnc.Izx\*emnc.Hx-emnc.Izy\*emnc.Hy-emnc.Izz\*emnc.Hz | A/m | Magnetization, z component | Domain 12 |
| emnc.normM | sqrt(realdot(emnc.Mx,emnc.Mx)+realdot(emnc.My,emnc.My)+realdot(emnc.Mz,emnc.Mz)) | A/m | Magnetization norm | Domain 12 |
| emnc.Ixx | 1 | 1 | Spatial identity matrix, xx component | Domain 12 |
| emnc.Iyx | 0 | 1 | Spatial identity matrix, yx component | Domain 12 |
| emnc.Izx | 0 | 1 | Spatial identity matrix, zx component | Domain 12 |
| emnc.Ixy | 0 | 1 | Spatial identity matrix, xy component | Domain 12 |
| emnc.Iyy | 1 | 1 | Spatial identity matrix, yy component | Domain 12 |
| emnc.Izy | 0 | 1 | Spatial identity matrix, zy component | Domain 12 |
| emnc.Ixz | 0 | 1 | Spatial identity matrix, xz component | Domain 12 |
| emnc.Iyz | 0 | 1 | Spatial identity matrix, yz component | Domain 12 |
| emnc.Izz | 1 | 1 | Spatial identity matrix, zz component | Domain 12 |
| emnc.Brx | BpRem\*cos(7\*argBp+tick) | T | Remanent flux density, x component | Domain 12 |
| emnc.Bry | BpRem\*sin(7\*argBp+tick) | T | Remanent flux density, y component | Domain 12 |
| emnc.Brz | 0 | T | Remanent flux density, z component | Domain 12 |
| emnc.normBr | sqrt(realdot(emnc.Brx,emnc.Brx)+realdot(emnc.Bry,emnc.Bry)+realdot(emnc.Brz,emnc.Brz)) | T | Remanent flux density norm | Domain 12 |
| emnc.chimxx | -1+emnc.murxx | 1 | Magnetic susceptibility, xx component | Domain 12 |
| emnc.chimyx | emnc.muryx | 1 | Magnetic susceptibility, yx component | Domain 12 |
| emnc.chimzx | emnc.murzx | 1 | Magnetic susceptibility, zx component | Domain 12 |
| emnc.chimxy | emnc.murxy | 1 | Magnetic susceptibility, xy component | Domain 12 |
| emnc.chimyy | -1+emnc.muryy | 1 | Magnetic susceptibility, yy component | Domain 12 |
| emnc.chimzy | emnc.murzy | 1 | Magnetic susceptibility, zy component | Domain 12 |
| emnc.chimxz | emnc.murxz | 1 | Magnetic susceptibility, xz component | Domain 12 |
| emnc.chimyz | emnc.muryz | 1 | Magnetic susceptibility, yz component | Domain 12 |
| emnc.chimzz | -1+emnc.murzz | 1 | Magnetic susceptibility, zz component | Domain 12 |
| emnc.unTx | 0 | Pa | Maxwell upward surface stress tensor, x component | Boundaries 63–66, 68, 70 |
| emnc.unTy | 0 | Pa | Maxwell upward surface stress tensor, y component | Boundaries 63–66, 68, 70 |
| emnc.unTz | 0 | Pa | Maxwell upward surface stress tensor, z component | Boundaries 63–66, 68, 70 |
| emnc.dnTx | emnc.dnTmx | Pa | Maxwell downward surface stress tensor, x component | Boundaries 63–66, 68, 70 |
| emnc.dnTy | emnc.dnTmy | Pa | Maxwell downward surface stress tensor, y component | Boundaries 63–66, 68, 70 |
| emnc.dnTz | emnc.dnTmz | Pa | Maxwell downward surface stress tensor, z component | Boundaries 63–66, 68, 70 |
| emnc.unx | unx |  | Normal vector up direction, x component | Boundaries 63–66, 68, 70 |
| emnc.uny | uny |  | Normal vector up direction, y component | Boundaries 63–66, 68, 70 |
| emnc.unz | unz |  | Normal vector up direction, z component | Boundaries 63–66, 68, 70 |
| emnc.dnx | dnx |  | Normal vector down direction, x component | Boundaries 63–66, 68, 70 |
| emnc.dny | dny |  | Normal vector down direction, y component | Boundaries 63–66, 68, 70 |
| emnc.dnz | dnz |  | Normal vector down direction, z component | Boundaries 63–66, 68, 70 |
| emnc.W | emnc.Wm | J/m^3 | Energy density | Domain 12 |
| emnc.dWm | emnc.Wm | J/m^3 | Integrand for total magnetic energy | Domain 12 |
| emnc.Wm | 0.5\*mu0\_const\*((emnc.murxx\*emnc.Hx+emnc.murxy\*emnc.Hy+emnc.murxz\*emnc.Hz)\*emnc.Hx+(emnc.muryx\*emnc.Hx+emnc.muryy\*emnc.Hy+emnc.muryz\*emnc.Hz)\*emnc.Hy+(emnc.murzx\*emnc.Hx+emnc.murzy\*emnc.Hy+emnc.murzz\*emnc.Hz)\*emnc.Hz) | J/m^3 | Magnetic energy density | Domain 12 |

##### Shape Functions

| Name | Shape function | Unit | Description | Shape frame | Selection |
| --- | --- | --- | --- | --- | --- |
| Vm | Lagrange (Quadratic) | A | Magnetic scalar potential | Material | Domain 12 |

##### Weak Expressions

| Weak expression | Integration frame | Selection |
| --- | --- | --- |
| emnc.d\*(-emnc.Bx\*test(Vmx)-emnc.By\*test(Vmy)-emnc.Bz\*test(Vmz)) | Material | Domain 12 |

#### 2.4.12. Magnetic Flux Conservation 10

Magnetic Flux Conservation 10

Selection

| Geometric entity level | Domain |
| Selection | Domain 10 |

Equations

Settings

| Description | Value |
| Constitutive relation | Remanent flux density |
| Remanent flux density, x component | BpRem\*cos(argBp\*(-1 + 9) + tick) |
| Remanent flux density, y component | BpRem\*sin(argBp\*(-1 + 9) + tick) |
| Remanent flux density, z component | 0 |
| Relative permeability | From material |
| Relative permeability | {{1, 0, 0}, {0, 1, 0}, {0, 0, 1}} |

Properties from material

| Property | Material | Property group |
| Relative permeability | Soft Iron (without losses) | Basic |

##### Variables

| Name | Expression | Unit | Description | Selection |
| --- | --- | --- | --- | --- |
| emnc.dnTmx | -0.5\*emnc.unx\*(real(down(emnc.Bx))\*real(down(emnc.Hx))+real(down(emnc.By))\*real(down(emnc.Hy))+real(down(emnc.Bz))\*real(down(emnc.Hz)))+real(down(emnc.Bx))\*(real(down(emnc.Hx))\*emnc.unx+real(down(emnc.Hy))\*emnc.uny+real(down(emnc.Hz))\*emnc.unz) | Pa | Maxwell downward magnetic surface stress tensor, x component | Boundaries 51–54, 56, 58 |
| emnc.dnTmy | -0.5\*emnc.uny\*(real(down(emnc.Bx))\*real(down(emnc.Hx))+real(down(emnc.By))\*real(down(emnc.Hy))+real(down(emnc.Bz))\*real(down(emnc.Hz)))+real(down(emnc.By))\*(real(down(emnc.Hx))\*emnc.unx+real(down(emnc.Hy))\*emnc.uny+real(down(emnc.Hz))\*emnc.unz) | Pa | Maxwell downward magnetic surface stress tensor, y component | Boundaries 51–54, 56, 58 |
| emnc.dnTmz | -0.5\*emnc.unz\*(real(down(emnc.Bx))\*real(down(emnc.Hx))+real(down(emnc.By))\*real(down(emnc.Hy))+real(down(emnc.Bz))\*real(down(emnc.Hz)))+real(down(emnc.Bz))\*(real(down(emnc.Hx))\*emnc.unx+real(down(emnc.Hy))\*emnc.uny+real(down(emnc.Hz))\*emnc.unz) | Pa | Maxwell downward magnetic surface stress tensor, z component | Boundaries 51–54, 56, 58 |
| emnc.Hx | -Vmx | A/m | Magnetic field, x component | Domain 10 |
| emnc.Hy | -Vmy | A/m | Magnetic field, y component | Domain 10 |
| emnc.Hz | -Vmz | A/m | Magnetic field, z component | Domain 10 |
| emnc.tHx | -VmTx | A/m | Tangential magnetic field, x component | Boundaries 51–54, 56, 58 |
| emnc.tHy | -VmTy | A/m | Tangential magnetic field, y component | Boundaries 51–54, 56, 58 |
| emnc.tHz | -VmTz | A/m | Tangential magnetic field, z component | Boundaries 51–54, 56, 58 |
| emnc.normH | sqrt(realdot(emnc.Hx,emnc.Hx)+realdot(emnc.Hy,emnc.Hy)+realdot(emnc.Hz,emnc.Hz)) | A/m | Magnetic field norm | Domain 10 |
| emnc.murxx | model.input.mur11 | 1 | Relative permeability, xx component | Domain 10 |
| emnc.muryx | model.input.mur21 | 1 | Relative permeability, yx component | Domain 10 |
| emnc.murzx | model.input.mur31 | 1 | Relative permeability, zx component | Domain 10 |
| emnc.murxy | model.input.mur12 | 1 | Relative permeability, xy component | Domain 10 |
| emnc.muryy | model.input.mur22 | 1 | Relative permeability, yy component | Domain 10 |
| emnc.murzy | model.input.mur32 | 1 | Relative permeability, zy component | Domain 10 |
| emnc.murxz | model.input.mur13 | 1 | Relative permeability, xz component | Domain 10 |
| emnc.muryz | model.input.mur23 | 1 | Relative permeability, yz component | Domain 10 |
| emnc.murzz | model.input.mur33 | 1 | Relative permeability, zz component | Domain 10 |
| emnc.Bx | mu0\_const\*emnc.Ixx\*emnc.Hx+mu0\_const\*emnc.Ixy\*emnc.Hy+mu0\_const\*emnc.Ixz\*emnc.Hz+mu0\_const\*emnc.chimxx\*emnc.Hx+mu0\_const\*emnc.chimxy\*emnc.Hy+mu0\_const\*emnc.chimxz\*emnc.Hz+emnc.Brx | T | Magnetic flux density, x component | Domain 10 |
| emnc.By | mu0\_const\*emnc.Iyx\*emnc.Hx+mu0\_const\*emnc.Iyy\*emnc.Hy+mu0\_const\*emnc.Iyz\*emnc.Hz+mu0\_const\*emnc.chimyx\*emnc.Hx+mu0\_const\*emnc.chimyy\*emnc.Hy+mu0\_const\*emnc.chimyz\*emnc.Hz+emnc.Bry | T | Magnetic flux density, y component | Domain 10 |
| emnc.Bz | mu0\_const\*emnc.Izx\*emnc.Hx+mu0\_const\*emnc.Izy\*emnc.Hy+mu0\_const\*emnc.Izz\*emnc.Hz+mu0\_const\*emnc.chimzx\*emnc.Hx+mu0\_const\*emnc.chimzy\*emnc.Hy+mu0\_const\*emnc.chimzz\*emnc.Hz+emnc.Brz | T | Magnetic flux density, z component | Domain 10 |
| emnc.normB | sqrt(realdot(emnc.Bx,emnc.Bx)+realdot(emnc.By,emnc.By)+realdot(emnc.Bz,emnc.Bz)) | T | Magnetic flux density norm | Domain 10 |
| emnc.Mx | emnc.Bx/mu0\_const-emnc.Ixx\*emnc.Hx-emnc.Ixy\*emnc.Hy-emnc.Ixz\*emnc.Hz | A/m | Magnetization, x component | Domain 10 |
| emnc.My | emnc.By/mu0\_const-emnc.Iyx\*emnc.Hx-emnc.Iyy\*emnc.Hy-emnc.Iyz\*emnc.Hz | A/m | Magnetization, y component | Domain 10 |
| emnc.Mz | emnc.Bz/mu0\_const-emnc.Izx\*emnc.Hx-emnc.Izy\*emnc.Hy-emnc.Izz\*emnc.Hz | A/m | Magnetization, z component | Domain 10 |
| emnc.normM | sqrt(realdot(emnc.Mx,emnc.Mx)+realdot(emnc.My,emnc.My)+realdot(emnc.Mz,emnc.Mz)) | A/m | Magnetization norm | Domain 10 |
| emnc.Ixx | 1 | 1 | Spatial identity matrix, xx component | Domain 10 |
| emnc.Iyx | 0 | 1 | Spatial identity matrix, yx component | Domain 10 |
| emnc.Izx | 0 | 1 | Spatial identity matrix, zx component | Domain 10 |
| emnc.Ixy | 0 | 1 | Spatial identity matrix, xy component | Domain 10 |
| emnc.Iyy | 1 | 1 | Spatial identity matrix, yy component | Domain 10 |
| emnc.Izy | 0 | 1 | Spatial identity matrix, zy component | Domain 10 |
| emnc.Ixz | 0 | 1 | Spatial identity matrix, xz component | Domain 10 |
| emnc.Iyz | 0 | 1 | Spatial identity matrix, yz component | Domain 10 |
| emnc.Izz | 1 | 1 | Spatial identity matrix, zz component | Domain 10 |
| emnc.Brx | BpRem\*cos(8\*argBp+tick) | T | Remanent flux density, x component | Domain 10 |
| emnc.Bry | BpRem\*sin(8\*argBp+tick) | T | Remanent flux density, y component | Domain 10 |
| emnc.Brz | 0 | T | Remanent flux density, z component | Domain 10 |
| emnc.normBr | sqrt(realdot(emnc.Brx,emnc.Brx)+realdot(emnc.Bry,emnc.Bry)+realdot(emnc.Brz,emnc.Brz)) | T | Remanent flux density norm | Domain 10 |
| emnc.chimxx | -1+emnc.murxx | 1 | Magnetic susceptibility, xx component | Domain 10 |
| emnc.chimyx | emnc.muryx | 1 | Magnetic susceptibility, yx component | Domain 10 |
| emnc.chimzx | emnc.murzx | 1 | Magnetic susceptibility, zx component | Domain 10 |
| emnc.chimxy | emnc.murxy | 1 | Magnetic susceptibility, xy component | Domain 10 |
| emnc.chimyy | -1+emnc.muryy | 1 | Magnetic susceptibility, yy component | Domain 10 |
| emnc.chimzy | emnc.murzy | 1 | Magnetic susceptibility, zy component | Domain 10 |
| emnc.chimxz | emnc.murxz | 1 | Magnetic susceptibility, xz component | Domain 10 |
| emnc.chimyz | emnc.muryz | 1 | Magnetic susceptibility, yz component | Domain 10 |
| emnc.chimzz | -1+emnc.murzz | 1 | Magnetic susceptibility, zz component | Domain 10 |
| emnc.unTx | 0 | Pa | Maxwell upward surface stress tensor, x component | Boundaries 51–54, 56, 58 |
| emnc.unTy | 0 | Pa | Maxwell upward surface stress tensor, y component | Boundaries 51–54, 56, 58 |
| emnc.unTz | 0 | Pa | Maxwell upward surface stress tensor, z component | Boundaries 51–54, 56, 58 |
| emnc.dnTx | emnc.dnTmx | Pa | Maxwell downward surface stress tensor, x component | Boundaries 51–54, 56, 58 |
| emnc.dnTy | emnc.dnTmy | Pa | Maxwell downward surface stress tensor, y component | Boundaries 51–54, 56, 58 |
| emnc.dnTz | emnc.dnTmz | Pa | Maxwell downward surface stress tensor, z component | Boundaries 51–54, 56, 58 |
| emnc.unx | unx |  | Normal vector up direction, x component | Boundaries 51–54, 56, 58 |
| emnc.uny | uny |  | Normal vector up direction, y component | Boundaries 51–54, 56, 58 |
| emnc.unz | unz |  | Normal vector up direction, z component | Boundaries 51–54, 56, 58 |
| emnc.dnx | dnx |  | Normal vector down direction, x component | Boundaries 51–54, 56, 58 |
| emnc.dny | dny |  | Normal vector down direction, y component | Boundaries 51–54, 56, 58 |
| emnc.dnz | dnz |  | Normal vector down direction, z component | Boundaries 51–54, 56, 58 |
| emnc.W | emnc.Wm | J/m^3 | Energy density | Domain 10 |
| emnc.dWm | emnc.Wm | J/m^3 | Integrand for total magnetic energy | Domain 10 |
| emnc.Wm | 0.5\*mu0\_const\*((emnc.murxx\*emnc.Hx+emnc.murxy\*emnc.Hy+emnc.murxz\*emnc.Hz)\*emnc.Hx+(emnc.muryx\*emnc.Hx+emnc.muryy\*emnc.Hy+emnc.muryz\*emnc.Hz)\*emnc.Hy+(emnc.murzx\*emnc.Hx+emnc.murzy\*emnc.Hy+emnc.murzz\*emnc.Hz)\*emnc.Hz) | J/m^3 | Magnetic energy density | Domain 10 |

##### Shape Functions

| Name | Shape function | Unit | Description | Shape frame | Selection |
| --- | --- | --- | --- | --- | --- |
| Vm | Lagrange (Quadratic) | A | Magnetic scalar potential | Material | Domain 10 |

##### Weak Expressions

| Weak expression | Integration frame | Selection |
| --- | --- | --- |
| emnc.d\*(-emnc.Bx\*test(Vmx)-emnc.By\*test(Vmy)-emnc.Bz\*test(Vmz)) | Material | Domain 10 |

#### 2.4.13. Magnetic Flux Conservation 11

Magnetic Flux Conservation 11

Selection

| Geometric entity level | Domain |
| Selection | Domain 8 |

Equations

Settings

| Description | Value |
| Constitutive relation | Remanent flux density |
| Remanent flux density, x component | BpRem\*cos(argBp\*(-1 + 10) + tick) |
| Remanent flux density, y component | BpRem\*sin(argBp\*(-1 + 10) + tick) |
| Remanent flux density, z component | 0 |
| Relative permeability | From material |
| Relative permeability | {{1, 0, 0}, {0, 1, 0}, {0, 0, 1}} |

Properties from material

| Property | Material | Property group |
| Relative permeability | Soft Iron (without losses) | Basic |

##### Variables

| Name | Expression | Unit | Description | Selection |
| --- | --- | --- | --- | --- |
| emnc.dnTmx | -0.5\*emnc.unx\*(real(down(emnc.Bx))\*real(down(emnc.Hx))+real(down(emnc.By))\*real(down(emnc.Hy))+real(down(emnc.Bz))\*real(down(emnc.Hz)))+real(down(emnc.Bx))\*(real(down(emnc.Hx))\*emnc.unx+real(down(emnc.Hy))\*emnc.uny+real(down(emnc.Hz))\*emnc.unz) | Pa | Maxwell downward magnetic surface stress tensor, x component | Boundaries 40–44, 46 |
| emnc.dnTmy | -0.5\*emnc.uny\*(real(down(emnc.Bx))\*real(down(emnc.Hx))+real(down(emnc.By))\*real(down(emnc.Hy))+real(down(emnc.Bz))\*real(down(emnc.Hz)))+real(down(emnc.By))\*(real(down(emnc.Hx))\*emnc.unx+real(down(emnc.Hy))\*emnc.uny+real(down(emnc.Hz))\*emnc.unz) | Pa | Maxwell downward magnetic surface stress tensor, y component | Boundaries 40–44, 46 |
| emnc.dnTmz | -0.5\*emnc.unz\*(real(down(emnc.Bx))\*real(down(emnc.Hx))+real(down(emnc.By))\*real(down(emnc.Hy))+real(down(emnc.Bz))\*real(down(emnc.Hz)))+real(down(emnc.Bz))\*(real(down(emnc.Hx))\*emnc.unx+real(down(emnc.Hy))\*emnc.uny+real(down(emnc.Hz))\*emnc.unz) | Pa | Maxwell downward magnetic surface stress tensor, z component | Boundaries 40–44, 46 |
| emnc.Hx | -Vmx | A/m | Magnetic field, x component | Domain 8 |
| emnc.Hy | -Vmy | A/m | Magnetic field, y component | Domain 8 |
| emnc.Hz | -Vmz | A/m | Magnetic field, z component | Domain 8 |
| emnc.tHx | -VmTx | A/m | Tangential magnetic field, x component | Boundaries 40–44, 46 |
| emnc.tHy | -VmTy | A/m | Tangential magnetic field, y component | Boundaries 40–44, 46 |
| emnc.tHz | -VmTz | A/m | Tangential magnetic field, z component | Boundaries 40–44, 46 |
| emnc.normH | sqrt(realdot(emnc.Hx,emnc.Hx)+realdot(emnc.Hy,emnc.Hy)+realdot(emnc.Hz,emnc.Hz)) | A/m | Magnetic field norm | Domain 8 |
| emnc.murxx | model.input.mur11 | 1 | Relative permeability, xx component | Domain 8 |
| emnc.muryx | model.input.mur21 | 1 | Relative permeability, yx component | Domain 8 |
| emnc.murzx | model.input.mur31 | 1 | Relative permeability, zx component | Domain 8 |
| emnc.murxy | model.input.mur12 | 1 | Relative permeability, xy component | Domain 8 |
| emnc.muryy | model.input.mur22 | 1 | Relative permeability, yy component | Domain 8 |
| emnc.murzy | model.input.mur32 | 1 | Relative permeability, zy component | Domain 8 |
| emnc.murxz | model.input.mur13 | 1 | Relative permeability, xz component | Domain 8 |
| emnc.muryz | model.input.mur23 | 1 | Relative permeability, yz component | Domain 8 |
| emnc.murzz | model.input.mur33 | 1 | Relative permeability, zz component | Domain 8 |
| emnc.Bx | mu0\_const\*emnc.Ixx\*emnc.Hx+mu0\_const\*emnc.Ixy\*emnc.Hy+mu0\_const\*emnc.Ixz\*emnc.Hz+mu0\_const\*emnc.chimxx\*emnc.Hx+mu0\_const\*emnc.chimxy\*emnc.Hy+mu0\_const\*emnc.chimxz\*emnc.Hz+emnc.Brx | T | Magnetic flux density, x component | Domain 8 |
| emnc.By | mu0\_const\*emnc.Iyx\*emnc.Hx+mu0\_const\*emnc.Iyy\*emnc.Hy+mu0\_const\*emnc.Iyz\*emnc.Hz+mu0\_const\*emnc.chimyx\*emnc.Hx+mu0\_const\*emnc.chimyy\*emnc.Hy+mu0\_const\*emnc.chimyz\*emnc.Hz+emnc.Bry | T | Magnetic flux density, y component | Domain 8 |
| emnc.Bz | mu0\_const\*emnc.Izx\*emnc.Hx+mu0\_const\*emnc.Izy\*emnc.Hy+mu0\_const\*emnc.Izz\*emnc.Hz+mu0\_const\*emnc.chimzx\*emnc.Hx+mu0\_const\*emnc.chimzy\*emnc.Hy+mu0\_const\*emnc.chimzz\*emnc.Hz+emnc.Brz | T | Magnetic flux density, z component | Domain 8 |
| emnc.normB | sqrt(realdot(emnc.Bx,emnc.Bx)+realdot(emnc.By,emnc.By)+realdot(emnc.Bz,emnc.Bz)) | T | Magnetic flux density norm | Domain 8 |
| emnc.Mx | emnc.Bx/mu0\_const-emnc.Ixx\*emnc.Hx-emnc.Ixy\*emnc.Hy-emnc.Ixz\*emnc.Hz | A/m | Magnetization, x component | Domain 8 |
| emnc.My | emnc.By/mu0\_const-emnc.Iyx\*emnc.Hx-emnc.Iyy\*emnc.Hy-emnc.Iyz\*emnc.Hz | A/m | Magnetization, y component | Domain 8 |
| emnc.Mz | emnc.Bz/mu0\_const-emnc.Izx\*emnc.Hx-emnc.Izy\*emnc.Hy-emnc.Izz\*emnc.Hz | A/m | Magnetization, z component | Domain 8 |
| emnc.normM | sqrt(realdot(emnc.Mx,emnc.Mx)+realdot(emnc.My,emnc.My)+realdot(emnc.Mz,emnc.Mz)) | A/m | Magnetization norm | Domain 8 |
| emnc.Ixx | 1 | 1 | Spatial identity matrix, xx component | Domain 8 |
| emnc.Iyx | 0 | 1 | Spatial identity matrix, yx component | Domain 8 |
| emnc.Izx | 0 | 1 | Spatial identity matrix, zx component | Domain 8 |
| emnc.Ixy | 0 | 1 | Spatial identity matrix, xy component | Domain 8 |
| emnc.Iyy | 1 | 1 | Spatial identity matrix, yy component | Domain 8 |
| emnc.Izy | 0 | 1 | Spatial identity matrix, zy component | Domain 8 |
| emnc.Ixz | 0 | 1 | Spatial identity matrix, xz component | Domain 8 |
| emnc.Iyz | 0 | 1 | Spatial identity matrix, yz component | Domain 8 |
| emnc.Izz | 1 | 1 | Spatial identity matrix, zz component | Domain 8 |
| emnc.Brx | BpRem\*cos(9\*argBp+tick) | T | Remanent flux density, x component | Domain 8 |
| emnc.Bry | BpRem\*sin(9\*argBp+tick) | T | Remanent flux density, y component | Domain 8 |
| emnc.Brz | 0 | T | Remanent flux density, z component | Domain 8 |
| emnc.normBr | sqrt(realdot(emnc.Brx,emnc.Brx)+realdot(emnc.Bry,emnc.Bry)+realdot(emnc.Brz,emnc.Brz)) | T | Remanent flux density norm | Domain 8 |
| emnc.chimxx | -1+emnc.murxx | 1 | Magnetic susceptibility, xx component | Domain 8 |
| emnc.chimyx | emnc.muryx | 1 | Magnetic susceptibility, yx component | Domain 8 |
| emnc.chimzx | emnc.murzx | 1 | Magnetic susceptibility, zx component | Domain 8 |
| emnc.chimxy | emnc.murxy | 1 | Magnetic susceptibility, xy component | Domain 8 |
| emnc.chimyy | -1+emnc.muryy | 1 | Magnetic susceptibility, yy component | Domain 8 |
| emnc.chimzy | emnc.murzy | 1 | Magnetic susceptibility, zy component | Domain 8 |
| emnc.chimxz | emnc.murxz | 1 | Magnetic susceptibility, xz component | Domain 8 |
| emnc.chimyz | emnc.muryz | 1 | Magnetic susceptibility, yz component | Domain 8 |
| emnc.chimzz | -1+emnc.murzz | 1 | Magnetic susceptibility, zz component | Domain 8 |
| emnc.unTx | 0 | Pa | Maxwell upward surface stress tensor, x component | Boundaries 40–44, 46 |
| emnc.unTy | 0 | Pa | Maxwell upward surface stress tensor, y component | Boundaries 40–44, 46 |
| emnc.unTz | 0 | Pa | Maxwell upward surface stress tensor, z component | Boundaries 40–44, 46 |
| emnc.dnTx | emnc.dnTmx | Pa | Maxwell downward surface stress tensor, x component | Boundaries 40–44, 46 |
| emnc.dnTy | emnc.dnTmy | Pa | Maxwell downward surface stress tensor, y component | Boundaries 40–44, 46 |
| emnc.dnTz | emnc.dnTmz | Pa | Maxwell downward surface stress tensor, z component | Boundaries 40–44, 46 |
| emnc.unx | unx |  | Normal vector up direction, x component | Boundaries 40–44, 46 |
| emnc.uny | uny |  | Normal vector up direction, y component | Boundaries 40–44, 46 |
| emnc.unz | unz |  | Normal vector up direction, z component | Boundaries 40–44, 46 |
| emnc.dnx | dnx |  | Normal vector down direction, x component | Boundaries 40–44, 46 |
| emnc.dny | dny |  | Normal vector down direction, y component | Boundaries 40–44, 46 |
| emnc.dnz | dnz |  | Normal vector down direction, z component | Boundaries 40–44, 46 |
| emnc.W | emnc.Wm | J/m^3 | Energy density | Domain 8 |
| emnc.dWm | emnc.Wm | J/m^3 | Integrand for total magnetic energy | Domain 8 |
| emnc.Wm | 0.5\*mu0\_const\*((emnc.murxx\*emnc.Hx+emnc.murxy\*emnc.Hy+emnc.murxz\*emnc.Hz)\*emnc.Hx+(emnc.muryx\*emnc.Hx+emnc.muryy\*emnc.Hy+emnc.muryz\*emnc.Hz)\*emnc.Hy+(emnc.murzx\*emnc.Hx+emnc.murzy\*emnc.Hy+emnc.murzz\*emnc.Hz)\*emnc.Hz) | J/m^3 | Magnetic energy density | Domain 8 |

##### Shape Functions

| Name | Shape function | Unit | Description | Shape frame | Selection |
| --- | --- | --- | --- | --- | --- |
| Vm | Lagrange (Quadratic) | A | Magnetic scalar potential | Material | Domain 8 |

##### Weak Expressions

| Weak expression | Integration frame | Selection |
| --- | --- | --- |
| emnc.d\*(-emnc.Bx\*test(Vmx)-emnc.By\*test(Vmy)-emnc.Bz\*test(Vmz)) | Material | Domain 8 |

#### 2.4.14. Magnetic Flux Conservation 12

Magnetic Flux Conservation 12

Selection

| Geometric entity level | Domain |
| Selection | Domain 6 |

Equations

Settings

| Description | Value |
| Constitutive relation | Remanent flux density |
| Remanent flux density, x component | BpRem\*cos(argBp\*(-1 + 11) + tick) |
| Remanent flux density, y component | BpRem\*sin(argBp\*(-1 + 11) + tick) |
| Remanent flux density, z component | 0 |
| Relative permeability | From material |
| Relative permeability | {{1, 0, 0}, {0, 1, 0}, {0, 0, 1}} |

Properties from material

| Property | Material | Property group |
| Relative permeability | Soft Iron (without losses) | Basic |

##### Variables

| Name | Expression | Unit | Description | Selection |
| --- | --- | --- | --- | --- |
| emnc.dnTmx | -0.5\*emnc.unx\*(real(down(emnc.Bx))\*real(down(emnc.Hx))+real(down(emnc.By))\*real(down(emnc.Hy))+real(down(emnc.Bz))\*real(down(emnc.Hz)))+real(down(emnc.Bx))\*(real(down(emnc.Hx))\*emnc.unx+real(down(emnc.Hy))\*emnc.uny+real(down(emnc.Hz))\*emnc.unz) | Pa | Maxwell downward magnetic surface stress tensor, x component | Boundaries 25–28, 32, 34 |
| emnc.dnTmy | -0.5\*emnc.uny\*(real(down(emnc.Bx))\*real(down(emnc.Hx))+real(down(emnc.By))\*real(down(emnc.Hy))+real(down(emnc.Bz))\*real(down(emnc.Hz)))+real(down(emnc.By))\*(real(down(emnc.Hx))\*emnc.unx+real(down(emnc.Hy))\*emnc.uny+real(down(emnc.Hz))\*emnc.unz) | Pa | Maxwell downward magnetic surface stress tensor, y component | Boundaries 25–28, 32, 34 |
| emnc.dnTmz | -0.5\*emnc.unz\*(real(down(emnc.Bx))\*real(down(emnc.Hx))+real(down(emnc.By))\*real(down(emnc.Hy))+real(down(emnc.Bz))\*real(down(emnc.Hz)))+real(down(emnc.Bz))\*(real(down(emnc.Hx))\*emnc.unx+real(down(emnc.Hy))\*emnc.uny+real(down(emnc.Hz))\*emnc.unz) | Pa | Maxwell downward magnetic surface stress tensor, z component | Boundaries 25–28, 32, 34 |
| emnc.Hx | -Vmx | A/m | Magnetic field, x component | Domain 6 |
| emnc.Hy | -Vmy | A/m | Magnetic field, y component | Domain 6 |
| emnc.Hz | -Vmz | A/m | Magnetic field, z component | Domain 6 |
| emnc.tHx | -VmTx | A/m | Tangential magnetic field, x component | Boundaries 25–28, 32, 34 |
| emnc.tHy | -VmTy | A/m | Tangential magnetic field, y component | Boundaries 25–28, 32, 34 |
| emnc.tHz | -VmTz | A/m | Tangential magnetic field, z component | Boundaries 25–28, 32, 34 |
| emnc.normH | sqrt(realdot(emnc.Hx,emnc.Hx)+realdot(emnc.Hy,emnc.Hy)+realdot(emnc.Hz,emnc.Hz)) | A/m | Magnetic field norm | Domain 6 |
| emnc.murxx | model.input.mur11 | 1 | Relative permeability, xx component | Domain 6 |
| emnc.muryx | model.input.mur21 | 1 | Relative permeability, yx component | Domain 6 |
| emnc.murzx | model.input.mur31 | 1 | Relative permeability, zx component | Domain 6 |
| emnc.murxy | model.input.mur12 | 1 | Relative permeability, xy component | Domain 6 |
| emnc.muryy | model.input.mur22 | 1 | Relative permeability, yy component | Domain 6 |
| emnc.murzy | model.input.mur32 | 1 | Relative permeability, zy component | Domain 6 |
| emnc.murxz | model.input.mur13 | 1 | Relative permeability, xz component | Domain 6 |
| emnc.muryz | model.input.mur23 | 1 | Relative permeability, yz component | Domain 6 |
| emnc.murzz | model.input.mur33 | 1 | Relative permeability, zz component | Domain 6 |
| emnc.Bx | mu0\_const\*emnc.Ixx\*emnc.Hx+mu0\_const\*emnc.Ixy\*emnc.Hy+mu0\_const\*emnc.Ixz\*emnc.Hz+mu0\_const\*emnc.chimxx\*emnc.Hx+mu0\_const\*emnc.chimxy\*emnc.Hy+mu0\_const\*emnc.chimxz\*emnc.Hz+emnc.Brx | T | Magnetic flux density, x component | Domain 6 |
| emnc.By | mu0\_const\*emnc.Iyx\*emnc.Hx+mu0\_const\*emnc.Iyy\*emnc.Hy+mu0\_const\*emnc.Iyz\*emnc.Hz+mu0\_const\*emnc.chimyx\*emnc.Hx+mu0\_const\*emnc.chimyy\*emnc.Hy+mu0\_const\*emnc.chimyz\*emnc.Hz+emnc.Bry | T | Magnetic flux density, y component | Domain 6 |
| emnc.Bz | mu0\_const\*emnc.Izx\*emnc.Hx+mu0\_const\*emnc.Izy\*emnc.Hy+mu0\_const\*emnc.Izz\*emnc.Hz+mu0\_const\*emnc.chimzx\*emnc.Hx+mu0\_const\*emnc.chimzy\*emnc.Hy+mu0\_const\*emnc.chimzz\*emnc.Hz+emnc.Brz | T | Magnetic flux density, z component | Domain 6 |
| emnc.normB | sqrt(realdot(emnc.Bx,emnc.Bx)+realdot(emnc.By,emnc.By)+realdot(emnc.Bz,emnc.Bz)) | T | Magnetic flux density norm | Domain 6 |
| emnc.Mx | emnc.Bx/mu0\_const-emnc.Ixx\*emnc.Hx-emnc.Ixy\*emnc.Hy-emnc.Ixz\*emnc.Hz | A/m | Magnetization, x component | Domain 6 |
| emnc.My | emnc.By/mu0\_const-emnc.Iyx\*emnc.Hx-emnc.Iyy\*emnc.Hy-emnc.Iyz\*emnc.Hz | A/m | Magnetization, y component | Domain 6 |
| emnc.Mz | emnc.Bz/mu0\_const-emnc.Izx\*emnc.Hx-emnc.Izy\*emnc.Hy-emnc.Izz\*emnc.Hz | A/m | Magnetization, z component | Domain 6 |
| emnc.normM | sqrt(realdot(emnc.Mx,emnc.Mx)+realdot(emnc.My,emnc.My)+realdot(emnc.Mz,emnc.Mz)) | A/m | Magnetization norm | Domain 6 |
| emnc.Ixx | 1 | 1 | Spatial identity matrix, xx component | Domain 6 |
| emnc.Iyx | 0 | 1 | Spatial identity matrix, yx component | Domain 6 |
| emnc.Izx | 0 | 1 | Spatial identity matrix, zx component | Domain 6 |
| emnc.Ixy | 0 | 1 | Spatial identity matrix, xy component | Domain 6 |
| emnc.Iyy | 1 | 1 | Spatial identity matrix, yy component | Domain 6 |
| emnc.Izy | 0 | 1 | Spatial identity matrix, zy component | Domain 6 |
| emnc.Ixz | 0 | 1 | Spatial identity matrix, xz component | Domain 6 |
| emnc.Iyz | 0 | 1 | Spatial identity matrix, yz component | Domain 6 |
| emnc.Izz | 1 | 1 | Spatial identity matrix, zz component | Domain 6 |
| emnc.Brx | BpRem\*cos(10\*argBp+tick) | T | Remanent flux density, x component | Domain 6 |
| emnc.Bry | BpRem\*sin(10\*argBp+tick) | T | Remanent flux density, y component | Domain 6 |
| emnc.Brz | 0 | T | Remanent flux density, z component | Domain 6 |
| emnc.normBr | sqrt(realdot(emnc.Brx,emnc.Brx)+realdot(emnc.Bry,emnc.Bry)+realdot(emnc.Brz,emnc.Brz)) | T | Remanent flux density norm | Domain 6 |
| emnc.chimxx | -1+emnc.murxx | 1 | Magnetic susceptibility, xx component | Domain 6 |
| emnc.chimyx | emnc.muryx | 1 | Magnetic susceptibility, yx component | Domain 6 |
| emnc.chimzx | emnc.murzx | 1 | Magnetic susceptibility, zx component | Domain 6 |
| emnc.chimxy | emnc.murxy | 1 | Magnetic susceptibility, xy component | Domain 6 |
| emnc.chimyy | -1+emnc.muryy | 1 | Magnetic susceptibility, yy component | Domain 6 |
| emnc.chimzy | emnc.murzy | 1 | Magnetic susceptibility, zy component | Domain 6 |
| emnc.chimxz | emnc.murxz | 1 | Magnetic susceptibility, xz component | Domain 6 |
| emnc.chimyz | emnc.muryz | 1 | Magnetic susceptibility, yz component | Domain 6 |
| emnc.chimzz | -1+emnc.murzz | 1 | Magnetic susceptibility, zz component | Domain 6 |
| emnc.unTx | 0 | Pa | Maxwell upward surface stress tensor, x component | Boundaries 25–28, 32, 34 |
| emnc.unTy | 0 | Pa | Maxwell upward surface stress tensor, y component | Boundaries 25–28, 32, 34 |
| emnc.unTz | 0 | Pa | Maxwell upward surface stress tensor, z component | Boundaries 25–28, 32, 34 |
| emnc.dnTx | emnc.dnTmx | Pa | Maxwell downward surface stress tensor, x component | Boundaries 25–28, 32, 34 |
| emnc.dnTy | emnc.dnTmy | Pa | Maxwell downward surface stress tensor, y component | Boundaries 25–28, 32, 34 |
| emnc.dnTz | emnc.dnTmz | Pa | Maxwell downward surface stress tensor, z component | Boundaries 25–28, 32, 34 |
| emnc.unx | unx |  | Normal vector up direction, x component | Boundaries 25–28, 32, 34 |
| emnc.uny | uny |  | Normal vector up direction, y component | Boundaries 25–28, 32, 34 |
| emnc.unz | unz |  | Normal vector up direction, z component | Boundaries 25–28, 32, 34 |
| emnc.dnx | dnx |  | Normal vector down direction, x component | Boundaries 25–28, 32, 34 |
| emnc.dny | dny |  | Normal vector down direction, y component | Boundaries 25–28, 32, 34 |
| emnc.dnz | dnz |  | Normal vector down direction, z component | Boundaries 25–28, 32, 34 |
| emnc.W | emnc.Wm | J/m^3 | Energy density | Domain 6 |
| emnc.dWm | emnc.Wm | J/m^3 | Integrand for total magnetic energy | Domain 6 |
| emnc.Wm | 0.5\*mu0\_const\*((emnc.murxx\*emnc.Hx+emnc.murxy\*emnc.Hy+emnc.murxz\*emnc.Hz)\*emnc.Hx+(emnc.muryx\*emnc.Hx+emnc.muryy\*emnc.Hy+emnc.muryz\*emnc.Hz)\*emnc.Hy+(emnc.murzx\*emnc.Hx+emnc.murzy\*emnc.Hy+emnc.murzz\*emnc.Hz)\*emnc.Hz) | J/m^3 | Magnetic energy density | Domain 6 |

##### Shape Functions

| Name | Shape function | Unit | Description | Shape frame | Selection |
| --- | --- | --- | --- | --- | --- |
| Vm | Lagrange (Quadratic) | A | Magnetic scalar potential | Material | Domain 6 |

##### Weak Expressions

| Weak expression | Integration frame | Selection |
| --- | --- | --- |
| emnc.d\*(-emnc.Bx\*test(Vmx)-emnc.By\*test(Vmy)-emnc.Bz\*test(Vmz)) | Material | Domain 6 |

#### 2.4.15. Magnetic Flux Conservation 13

Magnetic Flux Conservation 13

Selection

| Geometric entity level | Domain |
| Selection | Domain 4 |

Equations

Settings

| Description | Value |
| Constitutive relation | Remanent flux density |
| Remanent flux density, x component | BpRem\*cos(argBp\*(-1 + 12) + tick) |
| Remanent flux density, y component | BpRem\*sin(argBp\*(-1 + 12) + tick) |
| Remanent flux density, z component | 0 |
| Relative permeability | From material |
| Relative permeability | {{1, 0, 0}, {0, 1, 0}, {0, 0, 1}} |

Properties from material

| Property | Material | Property group |
| Relative permeability | Soft Iron (without losses) | Basic |

##### Variables

| Name | Expression | Unit | Description | Selection |
| --- | --- | --- | --- | --- |
| emnc.dnTmx | -0.5\*emnc.unx\*(real(down(emnc.Bx))\*real(down(emnc.Hx))+real(down(emnc.By))\*real(down(emnc.Hy))+real(down(emnc.Bz))\*real(down(emnc.Hz)))+real(down(emnc.Bx))\*(real(down(emnc.Hx))\*emnc.unx+real(down(emnc.Hy))\*emnc.uny+real(down(emnc.Hz))\*emnc.unz) | Pa | Maxwell downward magnetic surface stress tensor, x component | Boundaries 13–16, 20, 30 |
| emnc.dnTmy | -0.5\*emnc.uny\*(real(down(emnc.Bx))\*real(down(emnc.Hx))+real(down(emnc.By))\*real(down(emnc.Hy))+real(down(emnc.Bz))\*real(down(emnc.Hz)))+real(down(emnc.By))\*(real(down(emnc.Hx))\*emnc.unx+real(down(emnc.Hy))\*emnc.uny+real(down(emnc.Hz))\*emnc.unz) | Pa | Maxwell downward magnetic surface stress tensor, y component | Boundaries 13–16, 20, 30 |
| emnc.dnTmz | -0.5\*emnc.unz\*(real(down(emnc.Bx))\*real(down(emnc.Hx))+real(down(emnc.By))\*real(down(emnc.Hy))+real(down(emnc.Bz))\*real(down(emnc.Hz)))+real(down(emnc.Bz))\*(real(down(emnc.Hx))\*emnc.unx+real(down(emnc.Hy))\*emnc.uny+real(down(emnc.Hz))\*emnc.unz) | Pa | Maxwell downward magnetic surface stress tensor, z component | Boundaries 13–16, 20, 30 |
| emnc.Hx | -Vmx | A/m | Magnetic field, x component | Domain 4 |
| emnc.Hy | -Vmy | A/m | Magnetic field, y component | Domain 4 |
| emnc.Hz | -Vmz | A/m | Magnetic field, z component | Domain 4 |
| emnc.tHx | -VmTx | A/m | Tangential magnetic field, x component | Boundaries 13–16, 20, 30 |
| emnc.tHy | -VmTy | A/m | Tangential magnetic field, y component | Boundaries 13–16, 20, 30 |
| emnc.tHz | -VmTz | A/m | Tangential magnetic field, z component | Boundaries 13–16, 20, 30 |
| emnc.normH | sqrt(realdot(emnc.Hx,emnc.Hx)+realdot(emnc.Hy,emnc.Hy)+realdot(emnc.Hz,emnc.Hz)) | A/m | Magnetic field norm | Domain 4 |
| emnc.murxx | model.input.mur11 | 1 | Relative permeability, xx component | Domain 4 |
| emnc.muryx | model.input.mur21 | 1 | Relative permeability, yx component | Domain 4 |
| emnc.murzx | model.input.mur31 | 1 | Relative permeability, zx component | Domain 4 |
| emnc.murxy | model.input.mur12 | 1 | Relative permeability, xy component | Domain 4 |
| emnc.muryy | model.input.mur22 | 1 | Relative permeability, yy component | Domain 4 |
| emnc.murzy | model.input.mur32 | 1 | Relative permeability, zy component | Domain 4 |
| emnc.murxz | model.input.mur13 | 1 | Relative permeability, xz component | Domain 4 |
| emnc.muryz | model.input.mur23 | 1 | Relative permeability, yz component | Domain 4 |
| emnc.murzz | model.input.mur33 | 1 | Relative permeability, zz component | Domain 4 |
| emnc.Bx | mu0\_const\*emnc.Ixx\*emnc.Hx+mu0\_const\*emnc.Ixy\*emnc.Hy+mu0\_const\*emnc.Ixz\*emnc.Hz+mu0\_const\*emnc.chimxx\*emnc.Hx+mu0\_const\*emnc.chimxy\*emnc.Hy+mu0\_const\*emnc.chimxz\*emnc.Hz+emnc.Brx | T | Magnetic flux density, x component | Domain 4 |
| emnc.By | mu0\_const\*emnc.Iyx\*emnc.Hx+mu0\_const\*emnc.Iyy\*emnc.Hy+mu0\_const\*emnc.Iyz\*emnc.Hz+mu0\_const\*emnc.chimyx\*emnc.Hx+mu0\_const\*emnc.chimyy\*emnc.Hy+mu0\_const\*emnc.chimyz\*emnc.Hz+emnc.Bry | T | Magnetic flux density, y component | Domain 4 |
| emnc.Bz | mu0\_const\*emnc.Izx\*emnc.Hx+mu0\_const\*emnc.Izy\*emnc.Hy+mu0\_const\*emnc.Izz\*emnc.Hz+mu0\_const\*emnc.chimzx\*emnc.Hx+mu0\_const\*emnc.chimzy\*emnc.Hy+mu0\_const\*emnc.chimzz\*emnc.Hz+emnc.Brz | T | Magnetic flux density, z component | Domain 4 |
| emnc.normB | sqrt(realdot(emnc.Bx,emnc.Bx)+realdot(emnc.By,emnc.By)+realdot(emnc.Bz,emnc.Bz)) | T | Magnetic flux density norm | Domain 4 |
| emnc.Mx | emnc.Bx/mu0\_const-emnc.Ixx\*emnc.Hx-emnc.Ixy\*emnc.Hy-emnc.Ixz\*emnc.Hz | A/m | Magnetization, x component | Domain 4 |
| emnc.My | emnc.By/mu0\_const-emnc.Iyx\*emnc.Hx-emnc.Iyy\*emnc.Hy-emnc.Iyz\*emnc.Hz | A/m | Magnetization, y component | Domain 4 |
| emnc.Mz | emnc.Bz/mu0\_const-emnc.Izx\*emnc.Hx-emnc.Izy\*emnc.Hy-emnc.Izz\*emnc.Hz | A/m | Magnetization, z component | Domain 4 |
| emnc.normM | sqrt(realdot(emnc.Mx,emnc.Mx)+realdot(emnc.My,emnc.My)+realdot(emnc.Mz,emnc.Mz)) | A/m | Magnetization norm | Domain 4 |
| emnc.Ixx | 1 | 1 | Spatial identity matrix, xx component | Domain 4 |
| emnc.Iyx | 0 | 1 | Spatial identity matrix, yx component | Domain 4 |
| emnc.Izx | 0 | 1 | Spatial identity matrix, zx component | Domain 4 |
| emnc.Ixy | 0 | 1 | Spatial identity matrix, xy component | Domain 4 |
| emnc.Iyy | 1 | 1 | Spatial identity matrix, yy component | Domain 4 |
| emnc.Izy | 0 | 1 | Spatial identity matrix, zy component | Domain 4 |
| emnc.Ixz | 0 | 1 | Spatial identity matrix, xz component | Domain 4 |
| emnc.Iyz | 0 | 1 | Spatial identity matrix, yz component | Domain 4 |
| emnc.Izz | 1 | 1 | Spatial identity matrix, zz component | Domain 4 |
| emnc.Brx | BpRem\*cos(11\*argBp+tick) | T | Remanent flux density, x component | Domain 4 |
| emnc.Bry | BpRem\*sin(11\*argBp+tick) | T | Remanent flux density, y component | Domain 4 |
| emnc.Brz | 0 | T | Remanent flux density, z component | Domain 4 |
| emnc.normBr | sqrt(realdot(emnc.Brx,emnc.Brx)+realdot(emnc.Bry,emnc.Bry)+realdot(emnc.Brz,emnc.Brz)) | T | Remanent flux density norm | Domain 4 |
| emnc.chimxx | -1+emnc.murxx | 1 | Magnetic susceptibility, xx component | Domain 4 |
| emnc.chimyx | emnc.muryx | 1 | Magnetic susceptibility, yx component | Domain 4 |
| emnc.chimzx | emnc.murzx | 1 | Magnetic susceptibility, zx component | Domain 4 |
| emnc.chimxy | emnc.murxy | 1 | Magnetic susceptibility, xy component | Domain 4 |
| emnc.chimyy | -1+emnc.muryy | 1 | Magnetic susceptibility, yy component | Domain 4 |
| emnc.chimzy | emnc.murzy | 1 | Magnetic susceptibility, zy component | Domain 4 |
| emnc.chimxz | emnc.murxz | 1 | Magnetic susceptibility, xz component | Domain 4 |
| emnc.chimyz | emnc.muryz | 1 | Magnetic susceptibility, yz component | Domain 4 |
| emnc.chimzz | -1+emnc.murzz | 1 | Magnetic susceptibility, zz component | Domain 4 |
| emnc.unTx | 0 | Pa | Maxwell upward surface stress tensor, x component | Boundaries 13–16, 20, 30 |
| emnc.unTy | 0 | Pa | Maxwell upward surface stress tensor, y component | Boundaries 13–16, 20, 30 |
| emnc.unTz | 0 | Pa | Maxwell upward surface stress tensor, z component | Boundaries 13–16, 20, 30 |
| emnc.dnTx | emnc.dnTmx | Pa | Maxwell downward surface stress tensor, x component | Boundaries 13–16, 20, 30 |
| emnc.dnTy | emnc.dnTmy | Pa | Maxwell downward surface stress tensor, y component | Boundaries 13–16, 20, 30 |
| emnc.dnTz | emnc.dnTmz | Pa | Maxwell downward surface stress tensor, z component | Boundaries 13–16, 20, 30 |
| emnc.unx | unx |  | Normal vector up direction, x component | Boundaries 13–16, 20, 30 |
| emnc.uny | uny |  | Normal vector up direction, y component | Boundaries 13–16, 20, 30 |
| emnc.unz | unz |  | Normal vector up direction, z component | Boundaries 13–16, 20, 30 |
| emnc.dnx | dnx |  | Normal vector down direction, x component | Boundaries 13–16, 20, 30 |
| emnc.dny | dny |  | Normal vector down direction, y component | Boundaries 13–16, 20, 30 |
| emnc.dnz | dnz |  | Normal vector down direction, z component | Boundaries 13–16, 20, 30 |
| emnc.W | emnc.Wm | J/m^3 | Energy density | Domain 4 |
| emnc.dWm | emnc.Wm | J/m^3 | Integrand for total magnetic energy | Domain 4 |
| emnc.Wm | 0.5\*mu0\_const\*((emnc.murxx\*emnc.Hx+emnc.murxy\*emnc.Hy+emnc.murxz\*emnc.Hz)\*emnc.Hx+(emnc.muryx\*emnc.Hx+emnc.muryy\*emnc.Hy+emnc.muryz\*emnc.Hz)\*emnc.Hy+(emnc.murzx\*emnc.Hx+emnc.murzy\*emnc.Hy+emnc.murzz\*emnc.Hz)\*emnc.Hz) | J/m^3 | Magnetic energy density | Domain 4 |

##### Shape Functions

| Name | Shape function | Unit | Description | Shape frame | Selection |
| --- | --- | --- | --- | --- | --- |
| Vm | Lagrange (Quadratic) | A | Magnetic scalar potential | Material | Domain 4 |

##### Weak Expressions

| Weak expression | Integration frame | Selection |
| --- | --- | --- |
| emnc.d\*(-emnc.Bx\*test(Vmx)-emnc.By\*test(Vmy)-emnc.Bz\*test(Vmz)) | Material | Domain 4 |

#### 2.4.16. Magnetic Flux Conservation 14

Magnetic Flux Conservation 14

Selection

| Geometric entity level | Domain |
| Selection | Domain 2 |

Equations

Settings

| Description | Value |
| Constitutive relation | Remanent flux density |
| Remanent flux density, x component | BpRem\*cos(argBp\*(-1 + 13) + tick) |
| Remanent flux density, y component | BpRem\*sin(argBp\*(-1 + 13) + tick) |
| Remanent flux density, z component | 0 |
| Relative permeability | From material |
| Relative permeability | {{1, 0, 0}, {0, 1, 0}, {0, 0, 1}} |

Properties from material

| Property | Material | Property group |
| Relative permeability | Soft Iron (without losses) | Basic |

##### Variables

| Name | Expression | Unit | Description | Selection |
| --- | --- | --- | --- | --- |
| emnc.dnTmx | -0.5\*emnc.unx\*(real(down(emnc.Bx))\*real(down(emnc.Hx))+real(down(emnc.By))\*real(down(emnc.Hy))+real(down(emnc.Bz))\*real(down(emnc.Hz)))+real(down(emnc.Bx))\*(real(down(emnc.Hx))\*emnc.unx+real(down(emnc.Hy))\*emnc.uny+real(down(emnc.Hz))\*emnc.unz) | Pa | Maxwell downward magnetic surface stress tensor, x component | Boundaries 5–8, 17–18 |
| emnc.dnTmy | -0.5\*emnc.uny\*(real(down(emnc.Bx))\*real(down(emnc.Hx))+real(down(emnc.By))\*real(down(emnc.Hy))+real(down(emnc.Bz))\*real(down(emnc.Hz)))+real(down(emnc.By))\*(real(down(emnc.Hx))\*emnc.unx+real(down(emnc.Hy))\*emnc.uny+real(down(emnc.Hz))\*emnc.unz) | Pa | Maxwell downward magnetic surface stress tensor, y component | Boundaries 5–8, 17–18 |
| emnc.dnTmz | -0.5\*emnc.unz\*(real(down(emnc.Bx))\*real(down(emnc.Hx))+real(down(emnc.By))\*real(down(emnc.Hy))+real(down(emnc.Bz))\*real(down(emnc.Hz)))+real(down(emnc.Bz))\*(real(down(emnc.Hx))\*emnc.unx+real(down(emnc.Hy))\*emnc.uny+real(down(emnc.Hz))\*emnc.unz) | Pa | Maxwell downward magnetic surface stress tensor, z component | Boundaries 5–8, 17–18 |
| emnc.Hx | -Vmx | A/m | Magnetic field, x component | Domain 2 |
| emnc.Hy | -Vmy | A/m | Magnetic field, y component | Domain 2 |
| emnc.Hz | -Vmz | A/m | Magnetic field, z component | Domain 2 |
| emnc.tHx | -VmTx | A/m | Tangential magnetic field, x component | Boundaries 5–8, 17–18 |
| emnc.tHy | -VmTy | A/m | Tangential magnetic field, y component | Boundaries 5–8, 17–18 |
| emnc.tHz | -VmTz | A/m | Tangential magnetic field, z component | Boundaries 5–8, 17–18 |
| emnc.normH | sqrt(realdot(emnc.Hx,emnc.Hx)+realdot(emnc.Hy,emnc.Hy)+realdot(emnc.Hz,emnc.Hz)) | A/m | Magnetic field norm | Domain 2 |
| emnc.murxx | model.input.mur11 | 1 | Relative permeability, xx component | Domain 2 |
| emnc.muryx | model.input.mur21 | 1 | Relative permeability, yx component | Domain 2 |
| emnc.murzx | model.input.mur31 | 1 | Relative permeability, zx component | Domain 2 |
| emnc.murxy | model.input.mur12 | 1 | Relative permeability, xy component | Domain 2 |
| emnc.muryy | model.input.mur22 | 1 | Relative permeability, yy component | Domain 2 |
| emnc.murzy | model.input.mur32 | 1 | Relative permeability, zy component | Domain 2 |
| emnc.murxz | model.input.mur13 | 1 | Relative permeability, xz component | Domain 2 |
| emnc.muryz | model.input.mur23 | 1 | Relative permeability, yz component | Domain 2 |
| emnc.murzz | model.input.mur33 | 1 | Relative permeability, zz component | Domain 2 |
| emnc.Bx | mu0\_const\*emnc.Ixx\*emnc.Hx+mu0\_const\*emnc.Ixy\*emnc.Hy+mu0\_const\*emnc.Ixz\*emnc.Hz+mu0\_const\*emnc.chimxx\*emnc.Hx+mu0\_const\*emnc.chimxy\*emnc.Hy+mu0\_const\*emnc.chimxz\*emnc.Hz+emnc.Brx | T | Magnetic flux density, x component | Domain 2 |
| emnc.By | mu0\_const\*emnc.Iyx\*emnc.Hx+mu0\_const\*emnc.Iyy\*emnc.Hy+mu0\_const\*emnc.Iyz\*emnc.Hz+mu0\_const\*emnc.chimyx\*emnc.Hx+mu0\_const\*emnc.chimyy\*emnc.Hy+mu0\_const\*emnc.chimyz\*emnc.Hz+emnc.Bry | T | Magnetic flux density, y component | Domain 2 |
| emnc.Bz | mu0\_const\*emnc.Izx\*emnc.Hx+mu0\_const\*emnc.Izy\*emnc.Hy+mu0\_const\*emnc.Izz\*emnc.Hz+mu0\_const\*emnc.chimzx\*emnc.Hx+mu0\_const\*emnc.chimzy\*emnc.Hy+mu0\_const\*emnc.chimzz\*emnc.Hz+emnc.Brz | T | Magnetic flux density, z component | Domain 2 |
| emnc.normB | sqrt(realdot(emnc.Bx,emnc.Bx)+realdot(emnc.By,emnc.By)+realdot(emnc.Bz,emnc.Bz)) | T | Magnetic flux density norm | Domain 2 |
| emnc.Mx | emnc.Bx/mu0\_const-emnc.Ixx\*emnc.Hx-emnc.Ixy\*emnc.Hy-emnc.Ixz\*emnc.Hz | A/m | Magnetization, x component | Domain 2 |
| emnc.My | emnc.By/mu0\_const-emnc.Iyx\*emnc.Hx-emnc.Iyy\*emnc.Hy-emnc.Iyz\*emnc.Hz | A/m | Magnetization, y component | Domain 2 |
| emnc.Mz | emnc.Bz/mu0\_const-emnc.Izx\*emnc.Hx-emnc.Izy\*emnc.Hy-emnc.Izz\*emnc.Hz | A/m | Magnetization, z component | Domain 2 |
| emnc.normM | sqrt(realdot(emnc.Mx,emnc.Mx)+realdot(emnc.My,emnc.My)+realdot(emnc.Mz,emnc.Mz)) | A/m | Magnetization norm | Domain 2 |
| emnc.Ixx | 1 | 1 | Spatial identity matrix, xx component | Domain 2 |
| emnc.Iyx | 0 | 1 | Spatial identity matrix, yx component | Domain 2 |
| emnc.Izx | 0 | 1 | Spatial identity matrix, zx component | Domain 2 |
| emnc.Ixy | 0 | 1 | Spatial identity matrix, xy component | Domain 2 |
| emnc.Iyy | 1 | 1 | Spatial identity matrix, yy component | Domain 2 |
| emnc.Izy | 0 | 1 | Spatial identity matrix, zy component | Domain 2 |
| emnc.Ixz | 0 | 1 | Spatial identity matrix, xz component | Domain 2 |
| emnc.Iyz | 0 | 1 | Spatial identity matrix, yz component | Domain 2 |
| emnc.Izz | 1 | 1 | Spatial identity matrix, zz component | Domain 2 |
| emnc.Brx | BpRem\*cos(12\*argBp+tick) | T | Remanent flux density, x component | Domain 2 |
| emnc.Bry | BpRem\*sin(12\*argBp+tick) | T | Remanent flux density, y component | Domain 2 |
| emnc.Brz | 0 | T | Remanent flux density, z component | Domain 2 |
| emnc.normBr | sqrt(realdot(emnc.Brx,emnc.Brx)+realdot(emnc.Bry,emnc.Bry)+realdot(emnc.Brz,emnc.Brz)) | T | Remanent flux density norm | Domain 2 |
| emnc.chimxx | -1+emnc.murxx | 1 | Magnetic susceptibility, xx component | Domain 2 |
| emnc.chimyx | emnc.muryx | 1 | Magnetic susceptibility, yx component | Domain 2 |
| emnc.chimzx | emnc.murzx | 1 | Magnetic susceptibility, zx component | Domain 2 |
| emnc.chimxy | emnc.murxy | 1 | Magnetic susceptibility, xy component | Domain 2 |
| emnc.chimyy | -1+emnc.muryy | 1 | Magnetic susceptibility, yy component | Domain 2 |
| emnc.chimzy | emnc.murzy | 1 | Magnetic susceptibility, zy component | Domain 2 |
| emnc.chimxz | emnc.murxz | 1 | Magnetic susceptibility, xz component | Domain 2 |
| emnc.chimyz | emnc.muryz | 1 | Magnetic susceptibility, yz component | Domain 2 |
| emnc.chimzz | -1+emnc.murzz | 1 | Magnetic susceptibility, zz component | Domain 2 |
| emnc.unTx | 0 | Pa | Maxwell upward surface stress tensor, x component | Boundaries 5–8, 17–18 |
| emnc.unTy | 0 | Pa | Maxwell upward surface stress tensor, y component | Boundaries 5–8, 17–18 |
| emnc.unTz | 0 | Pa | Maxwell upward surface stress tensor, z component | Boundaries 5–8, 17–18 |
| emnc.dnTx | emnc.dnTmx | Pa | Maxwell downward surface stress tensor, x component | Boundaries 5–8, 17–18 |
| emnc.dnTy | emnc.dnTmy | Pa | Maxwell downward surface stress tensor, y component | Boundaries 5–8, 17–18 |
| emnc.dnTz | emnc.dnTmz | Pa | Maxwell downward surface stress tensor, z component | Boundaries 5–8, 17–18 |
| emnc.unx | unx |  | Normal vector up direction, x component | Boundaries 5–8, 17–18 |
| emnc.uny | uny |  | Normal vector up direction, y component | Boundaries 5–8, 17–18 |
| emnc.unz | unz |  | Normal vector up direction, z component | Boundaries 5–8, 17–18 |
| emnc.dnx | dnx |  | Normal vector down direction, x component | Boundaries 5–8, 17–18 |
| emnc.dny | dny |  | Normal vector down direction, y component | Boundaries 5–8, 17–18 |
| emnc.dnz | dnz |  | Normal vector down direction, z component | Boundaries 5–8, 17–18 |
| emnc.W | emnc.Wm | J/m^3 | Energy density | Domain 2 |
| emnc.dWm | emnc.Wm | J/m^3 | Integrand for total magnetic energy | Domain 2 |
| emnc.Wm | 0.5\*mu0\_const\*((emnc.murxx\*emnc.Hx+emnc.murxy\*emnc.Hy+emnc.murxz\*emnc.Hz)\*emnc.Hx+(emnc.muryx\*emnc.Hx+emnc.muryy\*emnc.Hy+emnc.muryz\*emnc.Hz)\*emnc.Hy+(emnc.murzx\*emnc.Hx+emnc.murzy\*emnc.Hy+emnc.murzz\*emnc.Hz)\*emnc.Hz) | J/m^3 | Magnetic energy density | Domain 2 |

##### Shape Functions

| Name | Shape function | Unit | Description | Shape frame | Selection |
| --- | --- | --- | --- | --- | --- |
| Vm | Lagrange (Quadratic) | A | Magnetic scalar potential | Material | Domain 2 |

##### Weak Expressions

| Weak expression | Integration frame | Selection |
| --- | --- | --- |
| emnc.d\*(-emnc.Bx\*test(Vmx)-emnc.By\*test(Vmy)-emnc.Bz\*test(Vmz)) | Material | Domain 2 |

#### 2.4.17. Magnetic Flux Conservation 15

Magnetic Flux Conservation 15

Selection

| Geometric entity level | Domain |
| Selection | Domain 3 |

Equations

Settings

| Description | Value |
| Constitutive relation | Remanent flux density |
| Remanent flux density, x component | BpRem\*cos(argBp\*(-1 + 14) + tick) |
| Remanent flux density, y component | BpRem\*sin(argBp\*(-1 + 14) + tick) |
| Remanent flux density, z component | 0 |
| Relative permeability | From material |
| Relative permeability | {{1, 0, 0}, {0, 1, 0}, {0, 0, 1}} |

Properties from material

| Property | Material | Property group |
| Relative permeability | Soft Iron (without losses) | Basic |

##### Variables

| Name | Expression | Unit | Description | Selection |
| --- | --- | --- | --- | --- |
| emnc.dnTmx | -0.5\*emnc.unx\*(real(down(emnc.Bx))\*real(down(emnc.Hx))+real(down(emnc.By))\*real(down(emnc.Hy))+real(down(emnc.Bz))\*real(down(emnc.Hz)))+real(down(emnc.Bx))\*(real(down(emnc.Hx))\*emnc.unx+real(down(emnc.Hy))\*emnc.uny+real(down(emnc.Hz))\*emnc.unz) | Pa | Maxwell downward magnetic surface stress tensor, x component | Boundaries 9–12, 19, 29 |
| emnc.dnTmy | -0.5\*emnc.uny\*(real(down(emnc.Bx))\*real(down(emnc.Hx))+real(down(emnc.By))\*real(down(emnc.Hy))+real(down(emnc.Bz))\*real(down(emnc.Hz)))+real(down(emnc.By))\*(real(down(emnc.Hx))\*emnc.unx+real(down(emnc.Hy))\*emnc.uny+real(down(emnc.Hz))\*emnc.unz) | Pa | Maxwell downward magnetic surface stress tensor, y component | Boundaries 9–12, 19, 29 |
| emnc.dnTmz | -0.5\*emnc.unz\*(real(down(emnc.Bx))\*real(down(emnc.Hx))+real(down(emnc.By))\*real(down(emnc.Hy))+real(down(emnc.Bz))\*real(down(emnc.Hz)))+real(down(emnc.Bz))\*(real(down(emnc.Hx))\*emnc.unx+real(down(emnc.Hy))\*emnc.uny+real(down(emnc.Hz))\*emnc.unz) | Pa | Maxwell downward magnetic surface stress tensor, z component | Boundaries 9–12, 19, 29 |
| emnc.Hx | -Vmx | A/m | Magnetic field, x component | Domain 3 |
| emnc.Hy | -Vmy | A/m | Magnetic field, y component | Domain 3 |
| emnc.Hz | -Vmz | A/m | Magnetic field, z component | Domain 3 |
| emnc.tHx | -VmTx | A/m | Tangential magnetic field, x component | Boundaries 9–12, 19, 29 |
| emnc.tHy | -VmTy | A/m | Tangential magnetic field, y component | Boundaries 9–12, 19, 29 |
| emnc.tHz | -VmTz | A/m | Tangential magnetic field, z component | Boundaries 9–12, 19, 29 |
| emnc.normH | sqrt(realdot(emnc.Hx,emnc.Hx)+realdot(emnc.Hy,emnc.Hy)+realdot(emnc.Hz,emnc.Hz)) | A/m | Magnetic field norm | Domain 3 |
| emnc.murxx | model.input.mur11 | 1 | Relative permeability, xx component | Domain 3 |
| emnc.muryx | model.input.mur21 | 1 | Relative permeability, yx component | Domain 3 |
| emnc.murzx | model.input.mur31 | 1 | Relative permeability, zx component | Domain 3 |
| emnc.murxy | model.input.mur12 | 1 | Relative permeability, xy component | Domain 3 |
| emnc.muryy | model.input.mur22 | 1 | Relative permeability, yy component | Domain 3 |
| emnc.murzy | model.input.mur32 | 1 | Relative permeability, zy component | Domain 3 |
| emnc.murxz | model.input.mur13 | 1 | Relative permeability, xz component | Domain 3 |
| emnc.muryz | model.input.mur23 | 1 | Relative permeability, yz component | Domain 3 |
| emnc.murzz | model.input.mur33 | 1 | Relative permeability, zz component | Domain 3 |
| emnc.Bx | mu0\_const\*emnc.Ixx\*emnc.Hx+mu0\_const\*emnc.Ixy\*emnc.Hy+mu0\_const\*emnc.Ixz\*emnc.Hz+mu0\_const\*emnc.chimxx\*emnc.Hx+mu0\_const\*emnc.chimxy\*emnc.Hy+mu0\_const\*emnc.chimxz\*emnc.Hz+emnc.Brx | T | Magnetic flux density, x component | Domain 3 |
| emnc.By | mu0\_const\*emnc.Iyx\*emnc.Hx+mu0\_const\*emnc.Iyy\*emnc.Hy+mu0\_const\*emnc.Iyz\*emnc.Hz+mu0\_const\*emnc.chimyx\*emnc.Hx+mu0\_const\*emnc.chimyy\*emnc.Hy+mu0\_const\*emnc.chimyz\*emnc.Hz+emnc.Bry | T | Magnetic flux density, y component | Domain 3 |
| emnc.Bz | mu0\_const\*emnc.Izx\*emnc.Hx+mu0\_const\*emnc.Izy\*emnc.Hy+mu0\_const\*emnc.Izz\*emnc.Hz+mu0\_const\*emnc.chimzx\*emnc.Hx+mu0\_const\*emnc.chimzy\*emnc.Hy+mu0\_const\*emnc.chimzz\*emnc.Hz+emnc.Brz | T | Magnetic flux density, z component | Domain 3 |
| emnc.normB | sqrt(realdot(emnc.Bx,emnc.Bx)+realdot(emnc.By,emnc.By)+realdot(emnc.Bz,emnc.Bz)) | T | Magnetic flux density norm | Domain 3 |
| emnc.Mx | emnc.Bx/mu0\_const-emnc.Ixx\*emnc.Hx-emnc.Ixy\*emnc.Hy-emnc.Ixz\*emnc.Hz | A/m | Magnetization, x component | Domain 3 |
| emnc.My | emnc.By/mu0\_const-emnc.Iyx\*emnc.Hx-emnc.Iyy\*emnc.Hy-emnc.Iyz\*emnc.Hz | A/m | Magnetization, y component | Domain 3 |
| emnc.Mz | emnc.Bz/mu0\_const-emnc.Izx\*emnc.Hx-emnc.Izy\*emnc.Hy-emnc.Izz\*emnc.Hz | A/m | Magnetization, z component | Domain 3 |
| emnc.normM | sqrt(realdot(emnc.Mx,emnc.Mx)+realdot(emnc.My,emnc.My)+realdot(emnc.Mz,emnc.Mz)) | A/m | Magnetization norm | Domain 3 |
| emnc.Ixx | 1 | 1 | Spatial identity matrix, xx component | Domain 3 |
| emnc.Iyx | 0 | 1 | Spatial identity matrix, yx component | Domain 3 |
| emnc.Izx | 0 | 1 | Spatial identity matrix, zx component | Domain 3 |
| emnc.Ixy | 0 | 1 | Spatial identity matrix, xy component | Domain 3 |
| emnc.Iyy | 1 | 1 | Spatial identity matrix, yy component | Domain 3 |
| emnc.Izy | 0 | 1 | Spatial identity matrix, zy component | Domain 3 |
| emnc.Ixz | 0 | 1 | Spatial identity matrix, xz component | Domain 3 |
| emnc.Iyz | 0 | 1 | Spatial identity matrix, yz component | Domain 3 |
| emnc.Izz | 1 | 1 | Spatial identity matrix, zz component | Domain 3 |
| emnc.Brx | BpRem\*cos(13\*argBp+tick) | T | Remanent flux density, x component | Domain 3 |
| emnc.Bry | BpRem\*sin(13\*argBp+tick) | T | Remanent flux density, y component | Domain 3 |
| emnc.Brz | 0 | T | Remanent flux density, z component | Domain 3 |
| emnc.normBr | sqrt(realdot(emnc.Brx,emnc.Brx)+realdot(emnc.Bry,emnc.Bry)+realdot(emnc.Brz,emnc.Brz)) | T | Remanent flux density norm | Domain 3 |
| emnc.chimxx | -1+emnc.murxx | 1 | Magnetic susceptibility, xx component | Domain 3 |
| emnc.chimyx | emnc.muryx | 1 | Magnetic susceptibility, yx component | Domain 3 |
| emnc.chimzx | emnc.murzx | 1 | Magnetic susceptibility, zx component | Domain 3 |
| emnc.chimxy | emnc.murxy | 1 | Magnetic susceptibility, xy component | Domain 3 |
| emnc.chimyy | -1+emnc.muryy | 1 | Magnetic susceptibility, yy component | Domain 3 |
| emnc.chimzy | emnc.murzy | 1 | Magnetic susceptibility, zy component | Domain 3 |
| emnc.chimxz | emnc.murxz | 1 | Magnetic susceptibility, xz component | Domain 3 |
| emnc.chimyz | emnc.muryz | 1 | Magnetic susceptibility, yz component | Domain 3 |
| emnc.chimzz | -1+emnc.murzz | 1 | Magnetic susceptibility, zz component | Domain 3 |
| emnc.unTx | 0 | Pa | Maxwell upward surface stress tensor, x component | Boundaries 9–12, 19, 29 |
| emnc.unTy | 0 | Pa | Maxwell upward surface stress tensor, y component | Boundaries 9–12, 19, 29 |
| emnc.unTz | 0 | Pa | Maxwell upward surface stress tensor, z component | Boundaries 9–12, 19, 29 |
| emnc.dnTx | emnc.dnTmx | Pa | Maxwell downward surface stress tensor, x component | Boundaries 9–12, 19, 29 |
| emnc.dnTy | emnc.dnTmy | Pa | Maxwell downward surface stress tensor, y component | Boundaries 9–12, 19, 29 |
| emnc.dnTz | emnc.dnTmz | Pa | Maxwell downward surface stress tensor, z component | Boundaries 9–12, 19, 29 |
| emnc.unx | unx |  | Normal vector up direction, x component | Boundaries 9–12, 19, 29 |
| emnc.uny | uny |  | Normal vector up direction, y component | Boundaries 9–12, 19, 29 |
| emnc.unz | unz |  | Normal vector up direction, z component | Boundaries 9–12, 19, 29 |
| emnc.dnx | dnx |  | Normal vector down direction, x component | Boundaries 9–12, 19, 29 |
| emnc.dny | dny |  | Normal vector down direction, y component | Boundaries 9–12, 19, 29 |
| emnc.dnz | dnz |  | Normal vector down direction, z component | Boundaries 9–12, 19, 29 |
| emnc.W | emnc.Wm | J/m^3 | Energy density | Domain 3 |
| emnc.dWm | emnc.Wm | J/m^3 | Integrand for total magnetic energy | Domain 3 |
| emnc.Wm | 0.5\*mu0\_const\*((emnc.murxx\*emnc.Hx+emnc.murxy\*emnc.Hy+emnc.murxz\*emnc.Hz)\*emnc.Hx+(emnc.muryx\*emnc.Hx+emnc.muryy\*emnc.Hy+emnc.muryz\*emnc.Hz)\*emnc.Hy+(emnc.murzx\*emnc.Hx+emnc.murzy\*emnc.Hy+emnc.murzz\*emnc.Hz)\*emnc.Hz) | J/m^3 | Magnetic energy density | Domain 3 |

##### Shape Functions

| Name | Shape function | Unit | Description | Shape frame | Selection |
| --- | --- | --- | --- | --- | --- |
| Vm | Lagrange (Quadratic) | A | Magnetic scalar potential | Material | Domain 3 |

##### Weak Expressions

| Weak expression | Integration frame | Selection |
| --- | --- | --- |
| emnc.d\*(-emnc.Bx\*test(Vmx)-emnc.By\*test(Vmy)-emnc.Bz\*test(Vmz)) | Material | Domain 3 |

#### 2.4.18. Magnetic Flux Conservation 16

Magnetic Flux Conservation 16

Selection

| Geometric entity level | Domain |
| Selection | Domain 5 |

Equations

Settings

| Description | Value |
| Constitutive relation | Remanent flux density |
| Remanent flux density, x component | BpRem\*cos(argBp\*(-1 + 15) + tick) |
| Remanent flux density, y component | BpRem\*sin(argBp\*(-1 + 15) + tick) |
| Remanent flux density, z component | 0 |
| Relative permeability | From material |
| Relative permeability | {{1, 0, 0}, {0, 1, 0}, {0, 0, 1}} |

Properties from material

| Property | Material | Property group |
| Relative permeability | Soft Iron (without losses) | Basic |

##### Variables

| Name | Expression | Unit | Description | Selection |
| --- | --- | --- | --- | --- |
| emnc.dnTmx | -0.5\*emnc.unx\*(real(down(emnc.Bx))\*real(down(emnc.Hx))+real(down(emnc.By))\*real(down(emnc.Hy))+real(down(emnc.Bz))\*real(down(emnc.Hz)))+real(down(emnc.Bx))\*(real(down(emnc.Hx))\*emnc.unx+real(down(emnc.Hy))\*emnc.uny+real(down(emnc.Hz))\*emnc.unz) | Pa | Maxwell downward magnetic surface stress tensor, x component | Boundaries 21–24, 31, 33 |
| emnc.dnTmy | -0.5\*emnc.uny\*(real(down(emnc.Bx))\*real(down(emnc.Hx))+real(down(emnc.By))\*real(down(emnc.Hy))+real(down(emnc.Bz))\*real(down(emnc.Hz)))+real(down(emnc.By))\*(real(down(emnc.Hx))\*emnc.unx+real(down(emnc.Hy))\*emnc.uny+real(down(emnc.Hz))\*emnc.unz) | Pa | Maxwell downward magnetic surface stress tensor, y component | Boundaries 21–24, 31, 33 |
| emnc.dnTmz | -0.5\*emnc.unz\*(real(down(emnc.Bx))\*real(down(emnc.Hx))+real(down(emnc.By))\*real(down(emnc.Hy))+real(down(emnc.Bz))\*real(down(emnc.Hz)))+real(down(emnc.Bz))\*(real(down(emnc.Hx))\*emnc.unx+real(down(emnc.Hy))\*emnc.uny+real(down(emnc.Hz))\*emnc.unz) | Pa | Maxwell downward magnetic surface stress tensor, z component | Boundaries 21–24, 31, 33 |
| emnc.Hx | -Vmx | A/m | Magnetic field, x component | Domain 5 |
| emnc.Hy | -Vmy | A/m | Magnetic field, y component | Domain 5 |
| emnc.Hz | -Vmz | A/m | Magnetic field, z component | Domain 5 |
| emnc.tHx | -VmTx | A/m | Tangential magnetic field, x component | Boundaries 21–24, 31, 33 |
| emnc.tHy | -VmTy | A/m | Tangential magnetic field, y component | Boundaries 21–24, 31, 33 |
| emnc.tHz | -VmTz | A/m | Tangential magnetic field, z component | Boundaries 21–24, 31, 33 |
| emnc.normH | sqrt(realdot(emnc.Hx,emnc.Hx)+realdot(emnc.Hy,emnc.Hy)+realdot(emnc.Hz,emnc.Hz)) | A/m | Magnetic field norm | Domain 5 |
| emnc.murxx | model.input.mur11 | 1 | Relative permeability, xx component | Domain 5 |
| emnc.muryx | model.input.mur21 | 1 | Relative permeability, yx component | Domain 5 |
| emnc.murzx | model.input.mur31 | 1 | Relative permeability, zx component | Domain 5 |
| emnc.murxy | model.input.mur12 | 1 | Relative permeability, xy component | Domain 5 |
| emnc.muryy | model.input.mur22 | 1 | Relative permeability, yy component | Domain 5 |
| emnc.murzy | model.input.mur32 | 1 | Relative permeability, zy component | Domain 5 |
| emnc.murxz | model.input.mur13 | 1 | Relative permeability, xz component | Domain 5 |
| emnc.muryz | model.input.mur23 | 1 | Relative permeability, yz component | Domain 5 |
| emnc.murzz | model.input.mur33 | 1 | Relative permeability, zz component | Domain 5 |
| emnc.Bx | mu0\_const\*emnc.Ixx\*emnc.Hx+mu0\_const\*emnc.Ixy\*emnc.Hy+mu0\_const\*emnc.Ixz\*emnc.Hz+mu0\_const\*emnc.chimxx\*emnc.Hx+mu0\_const\*emnc.chimxy\*emnc.Hy+mu0\_const\*emnc.chimxz\*emnc.Hz+emnc.Brx | T | Magnetic flux density, x component | Domain 5 |
| emnc.By | mu0\_const\*emnc.Iyx\*emnc.Hx+mu0\_const\*emnc.Iyy\*emnc.Hy+mu0\_const\*emnc.Iyz\*emnc.Hz+mu0\_const\*emnc.chimyx\*emnc.Hx+mu0\_const\*emnc.chimyy\*emnc.Hy+mu0\_const\*emnc.chimyz\*emnc.Hz+emnc.Bry | T | Magnetic flux density, y component | Domain 5 |
| emnc.Bz | mu0\_const\*emnc.Izx\*emnc.Hx+mu0\_const\*emnc.Izy\*emnc.Hy+mu0\_const\*emnc.Izz\*emnc.Hz+mu0\_const\*emnc.chimzx\*emnc.Hx+mu0\_const\*emnc.chimzy\*emnc.Hy+mu0\_const\*emnc.chimzz\*emnc.Hz+emnc.Brz | T | Magnetic flux density, z component | Domain 5 |
| emnc.normB | sqrt(realdot(emnc.Bx,emnc.Bx)+realdot(emnc.By,emnc.By)+realdot(emnc.Bz,emnc.Bz)) | T | Magnetic flux density norm | Domain 5 |
| emnc.Mx | emnc.Bx/mu0\_const-emnc.Ixx\*emnc.Hx-emnc.Ixy\*emnc.Hy-emnc.Ixz\*emnc.Hz | A/m | Magnetization, x component | Domain 5 |
| emnc.My | emnc.By/mu0\_const-emnc.Iyx\*emnc.Hx-emnc.Iyy\*emnc.Hy-emnc.Iyz\*emnc.Hz | A/m | Magnetization, y component | Domain 5 |
| emnc.Mz | emnc.Bz/mu0\_const-emnc.Izx\*emnc.Hx-emnc.Izy\*emnc.Hy-emnc.Izz\*emnc.Hz | A/m | Magnetization, z component | Domain 5 |
| emnc.normM | sqrt(realdot(emnc.Mx,emnc.Mx)+realdot(emnc.My,emnc.My)+realdot(emnc.Mz,emnc.Mz)) | A/m | Magnetization norm | Domain 5 |
| emnc.Ixx | 1 | 1 | Spatial identity matrix, xx component | Domain 5 |
| emnc.Iyx | 0 | 1 | Spatial identity matrix, yx component | Domain 5 |
| emnc.Izx | 0 | 1 | Spatial identity matrix, zx component | Domain 5 |
| emnc.Ixy | 0 | 1 | Spatial identity matrix, xy component | Domain 5 |
| emnc.Iyy | 1 | 1 | Spatial identity matrix, yy component | Domain 5 |
| emnc.Izy | 0 | 1 | Spatial identity matrix, zy component | Domain 5 |
| emnc.Ixz | 0 | 1 | Spatial identity matrix, xz component | Domain 5 |
| emnc.Iyz | 0 | 1 | Spatial identity matrix, yz component | Domain 5 |
| emnc.Izz | 1 | 1 | Spatial identity matrix, zz component | Domain 5 |
| emnc.Brx | BpRem\*cos(14\*argBp+tick) | T | Remanent flux density, x component | Domain 5 |
| emnc.Bry | BpRem\*sin(14\*argBp+tick) | T | Remanent flux density, y component | Domain 5 |
| emnc.Brz | 0 | T | Remanent flux density, z component | Domain 5 |
| emnc.normBr | sqrt(realdot(emnc.Brx,emnc.Brx)+realdot(emnc.Bry,emnc.Bry)+realdot(emnc.Brz,emnc.Brz)) | T | Remanent flux density norm | Domain 5 |
| emnc.chimxx | -1+emnc.murxx | 1 | Magnetic susceptibility, xx component | Domain 5 |
| emnc.chimyx | emnc.muryx | 1 | Magnetic susceptibility, yx component | Domain 5 |
| emnc.chimzx | emnc.murzx | 1 | Magnetic susceptibility, zx component | Domain 5 |
| emnc.chimxy | emnc.murxy | 1 | Magnetic susceptibility, xy component | Domain 5 |
| emnc.chimyy | -1+emnc.muryy | 1 | Magnetic susceptibility, yy component | Domain 5 |
| emnc.chimzy | emnc.murzy | 1 | Magnetic susceptibility, zy component | Domain 5 |
| emnc.chimxz | emnc.murxz | 1 | Magnetic susceptibility, xz component | Domain 5 |
| emnc.chimyz | emnc.muryz | 1 | Magnetic susceptibility, yz component | Domain 5 |
| emnc.chimzz | -1+emnc.murzz | 1 | Magnetic susceptibility, zz component | Domain 5 |
| emnc.unTx | 0 | Pa | Maxwell upward surface stress tensor, x component | Boundaries 21–24, 31, 33 |
| emnc.unTy | 0 | Pa | Maxwell upward surface stress tensor, y component | Boundaries 21–24, 31, 33 |
| emnc.unTz | 0 | Pa | Maxwell upward surface stress tensor, z component | Boundaries 21–24, 31, 33 |
| emnc.dnTx | emnc.dnTmx | Pa | Maxwell downward surface stress tensor, x component | Boundaries 21–24, 31, 33 |
| emnc.dnTy | emnc.dnTmy | Pa | Maxwell downward surface stress tensor, y component | Boundaries 21–24, 31, 33 |
| emnc.dnTz | emnc.dnTmz | Pa | Maxwell downward surface stress tensor, z component | Boundaries 21–24, 31, 33 |
| emnc.unx | unx |  | Normal vector up direction, x component | Boundaries 21–24, 31, 33 |
| emnc.uny | uny |  | Normal vector up direction, y component | Boundaries 21–24, 31, 33 |
| emnc.unz | unz |  | Normal vector up direction, z component | Boundaries 21–24, 31, 33 |
| emnc.dnx | dnx |  | Normal vector down direction, x component | Boundaries 21–24, 31, 33 |
| emnc.dny | dny |  | Normal vector down direction, y component | Boundaries 21–24, 31, 33 |
| emnc.dnz | dnz |  | Normal vector down direction, z component | Boundaries 21–24, 31, 33 |
| emnc.W | emnc.Wm | J/m^3 | Energy density | Domain 5 |
| emnc.dWm | emnc.Wm | J/m^3 | Integrand for total magnetic energy | Domain 5 |
| emnc.Wm | 0.5\*mu0\_const\*((emnc.murxx\*emnc.Hx+emnc.murxy\*emnc.Hy+emnc.murxz\*emnc.Hz)\*emnc.Hx+(emnc.muryx\*emnc.Hx+emnc.muryy\*emnc.Hy+emnc.muryz\*emnc.Hz)\*emnc.Hy+(emnc.murzx\*emnc.Hx+emnc.murzy\*emnc.Hy+emnc.murzz\*emnc.Hz)\*emnc.Hz) | J/m^3 | Magnetic energy density | Domain 5 |

##### Shape Functions

| Name | Shape function | Unit | Description | Shape frame | Selection |
| --- | --- | --- | --- | --- | --- |
| Vm | Lagrange (Quadratic) | A | Magnetic scalar potential | Material | Domain 5 |

##### Weak Expressions

| Weak expression | Integration frame | Selection |
| --- | --- | --- |
| emnc.d\*(-emnc.Bx\*test(Vmx)-emnc.By\*test(Vmy)-emnc.Bz\*test(Vmz)) | Material | Domain 5 |

#### 2.4.19. Magnetic Flux Conservation 17

Magnetic Flux Conservation 17

Selection

| Geometric entity level | Domain |
| Selection | Domain 7 |

Equations

Settings

| Description | Value |
| Constitutive relation | Remanent flux density |
| Remanent flux density, x component | BpRem\*cos(argBp\*(-1 + 16) + tick) |
| Remanent flux density, y component | BpRem\*sin(argBp\*(-1 + 16) + tick) |
| Remanent flux density, z component | 0 |
| Relative permeability | From material |
| Relative permeability | {{1, 0, 0}, {0, 1, 0}, {0, 0, 1}} |

Properties from material

| Property | Material | Property group |
| Relative permeability | Soft Iron (without losses) | Basic |

##### Variables

| Name | Expression | Unit | Description | Selection |
| --- | --- | --- | --- | --- |
| emnc.dnTmx | -0.5\*emnc.unx\*(real(down(emnc.Bx))\*real(down(emnc.Hx))+real(down(emnc.By))\*real(down(emnc.Hy))+real(down(emnc.Bz))\*real(down(emnc.Hz)))+real(down(emnc.Bx))\*(real(down(emnc.Hx))\*emnc.unx+real(down(emnc.Hy))\*emnc.uny+real(down(emnc.Hz))\*emnc.unz) | Pa | Maxwell downward magnetic surface stress tensor, x component | Boundaries 35–39, 45 |
| emnc.dnTmy | -0.5\*emnc.uny\*(real(down(emnc.Bx))\*real(down(emnc.Hx))+real(down(emnc.By))\*real(down(emnc.Hy))+real(down(emnc.Bz))\*real(down(emnc.Hz)))+real(down(emnc.By))\*(real(down(emnc.Hx))\*emnc.unx+real(down(emnc.Hy))\*emnc.uny+real(down(emnc.Hz))\*emnc.unz) | Pa | Maxwell downward magnetic surface stress tensor, y component | Boundaries 35–39, 45 |
| emnc.dnTmz | -0.5\*emnc.unz\*(real(down(emnc.Bx))\*real(down(emnc.Hx))+real(down(emnc.By))\*real(down(emnc.Hy))+real(down(emnc.Bz))\*real(down(emnc.Hz)))+real(down(emnc.Bz))\*(real(down(emnc.Hx))\*emnc.unx+real(down(emnc.Hy))\*emnc.uny+real(down(emnc.Hz))\*emnc.unz) | Pa | Maxwell downward magnetic surface stress tensor, z component | Boundaries 35–39, 45 |
| emnc.Hx | -Vmx | A/m | Magnetic field, x component | Domain 7 |
| emnc.Hy | -Vmy | A/m | Magnetic field, y component | Domain 7 |
| emnc.Hz | -Vmz | A/m | Magnetic field, z component | Domain 7 |
| emnc.tHx | -VmTx | A/m | Tangential magnetic field, x component | Boundaries 35–39, 45 |
| emnc.tHy | -VmTy | A/m | Tangential magnetic field, y component | Boundaries 35–39, 45 |
| emnc.tHz | -VmTz | A/m | Tangential magnetic field, z component | Boundaries 35–39, 45 |
| emnc.normH | sqrt(realdot(emnc.Hx,emnc.Hx)+realdot(emnc.Hy,emnc.Hy)+realdot(emnc.Hz,emnc.Hz)) | A/m | Magnetic field norm | Domain 7 |
| emnc.murxx | model.input.mur11 | 1 | Relative permeability, xx component | Domain 7 |
| emnc.muryx | model.input.mur21 | 1 | Relative permeability, yx component | Domain 7 |
| emnc.murzx | model.input.mur31 | 1 | Relative permeability, zx component | Domain 7 |
| emnc.murxy | model.input.mur12 | 1 | Relative permeability, xy component | Domain 7 |
| emnc.muryy | model.input.mur22 | 1 | Relative permeability, yy component | Domain 7 |
| emnc.murzy | model.input.mur32 | 1 | Relative permeability, zy component | Domain 7 |
| emnc.murxz | model.input.mur13 | 1 | Relative permeability, xz component | Domain 7 |
| emnc.muryz | model.input.mur23 | 1 | Relative permeability, yz component | Domain 7 |
| emnc.murzz | model.input.mur33 | 1 | Relative permeability, zz component | Domain 7 |
| emnc.Bx | mu0\_const\*emnc.Ixx\*emnc.Hx+mu0\_const\*emnc.Ixy\*emnc.Hy+mu0\_const\*emnc.Ixz\*emnc.Hz+mu0\_const\*emnc.chimxx\*emnc.Hx+mu0\_const\*emnc.chimxy\*emnc.Hy+mu0\_const\*emnc.chimxz\*emnc.Hz+emnc.Brx | T | Magnetic flux density, x component | Domain 7 |
| emnc.By | mu0\_const\*emnc.Iyx\*emnc.Hx+mu0\_const\*emnc.Iyy\*emnc.Hy+mu0\_const\*emnc.Iyz\*emnc.Hz+mu0\_const\*emnc.chimyx\*emnc.Hx+mu0\_const\*emnc.chimyy\*emnc.Hy+mu0\_const\*emnc.chimyz\*emnc.Hz+emnc.Bry | T | Magnetic flux density, y component | Domain 7 |
| emnc.Bz | mu0\_const\*emnc.Izx\*emnc.Hx+mu0\_const\*emnc.Izy\*emnc.Hy+mu0\_const\*emnc.Izz\*emnc.Hz+mu0\_const\*emnc.chimzx\*emnc.Hx+mu0\_const\*emnc.chimzy\*emnc.Hy+mu0\_const\*emnc.chimzz\*emnc.Hz+emnc.Brz | T | Magnetic flux density, z component | Domain 7 |
| emnc.normB | sqrt(realdot(emnc.Bx,emnc.Bx)+realdot(emnc.By,emnc.By)+realdot(emnc.Bz,emnc.Bz)) | T | Magnetic flux density norm | Domain 7 |
| emnc.Mx | emnc.Bx/mu0\_const-emnc.Ixx\*emnc.Hx-emnc.Ixy\*emnc.Hy-emnc.Ixz\*emnc.Hz | A/m | Magnetization, x component | Domain 7 |
| emnc.My | emnc.By/mu0\_const-emnc.Iyx\*emnc.Hx-emnc.Iyy\*emnc.Hy-emnc.Iyz\*emnc.Hz | A/m | Magnetization, y component | Domain 7 |
| emnc.Mz | emnc.Bz/mu0\_const-emnc.Izx\*emnc.Hx-emnc.Izy\*emnc.Hy-emnc.Izz\*emnc.Hz | A/m | Magnetization, z component | Domain 7 |
| emnc.normM | sqrt(realdot(emnc.Mx,emnc.Mx)+realdot(emnc.My,emnc.My)+realdot(emnc.Mz,emnc.Mz)) | A/m | Magnetization norm | Domain 7 |
| emnc.Ixx | 1 | 1 | Spatial identity matrix, xx component | Domain 7 |
| emnc.Iyx | 0 | 1 | Spatial identity matrix, yx component | Domain 7 |
| emnc.Izx | 0 | 1 | Spatial identity matrix, zx component | Domain 7 |
| emnc.Ixy | 0 | 1 | Spatial identity matrix, xy component | Domain 7 |
| emnc.Iyy | 1 | 1 | Spatial identity matrix, yy component | Domain 7 |
| emnc.Izy | 0 | 1 | Spatial identity matrix, zy component | Domain 7 |
| emnc.Ixz | 0 | 1 | Spatial identity matrix, xz component | Domain 7 |
| emnc.Iyz | 0 | 1 | Spatial identity matrix, yz component | Domain 7 |
| emnc.Izz | 1 | 1 | Spatial identity matrix, zz component | Domain 7 |
| emnc.Brx | BpRem\*cos(15\*argBp+tick) | T | Remanent flux density, x component | Domain 7 |
| emnc.Bry | BpRem\*sin(15\*argBp+tick) | T | Remanent flux density, y component | Domain 7 |
| emnc.Brz | 0 | T | Remanent flux density, z component | Domain 7 |
| emnc.normBr | sqrt(realdot(emnc.Brx,emnc.Brx)+realdot(emnc.Bry,emnc.Bry)+realdot(emnc.Brz,emnc.Brz)) | T | Remanent flux density norm | Domain 7 |
| emnc.chimxx | -1+emnc.murxx | 1 | Magnetic susceptibility, xx component | Domain 7 |
| emnc.chimyx | emnc.muryx | 1 | Magnetic susceptibility, yx component | Domain 7 |
| emnc.chimzx | emnc.murzx | 1 | Magnetic susceptibility, zx component | Domain 7 |
| emnc.chimxy | emnc.murxy | 1 | Magnetic susceptibility, xy component | Domain 7 |
| emnc.chimyy | -1+emnc.muryy | 1 | Magnetic susceptibility, yy component | Domain 7 |
| emnc.chimzy | emnc.murzy | 1 | Magnetic susceptibility, zy component | Domain 7 |
| emnc.chimxz | emnc.murxz | 1 | Magnetic susceptibility, xz component | Domain 7 |
| emnc.chimyz | emnc.muryz | 1 | Magnetic susceptibility, yz component | Domain 7 |
| emnc.chimzz | -1+emnc.murzz | 1 | Magnetic susceptibility, zz component | Domain 7 |
| emnc.unTx | 0 | Pa | Maxwell upward surface stress tensor, x component | Boundaries 35–39, 45 |
| emnc.unTy | 0 | Pa | Maxwell upward surface stress tensor, y component | Boundaries 35–39, 45 |
| emnc.unTz | 0 | Pa | Maxwell upward surface stress tensor, z component | Boundaries 35–39, 45 |
| emnc.dnTx | emnc.dnTmx | Pa | Maxwell downward surface stress tensor, x component | Boundaries 35–39, 45 |
| emnc.dnTy | emnc.dnTmy | Pa | Maxwell downward surface stress tensor, y component | Boundaries 35–39, 45 |
| emnc.dnTz | emnc.dnTmz | Pa | Maxwell downward surface stress tensor, z component | Boundaries 35–39, 45 |
| emnc.unx | unx |  | Normal vector up direction, x component | Boundaries 35–39, 45 |
| emnc.uny | uny |  | Normal vector up direction, y component | Boundaries 35–39, 45 |
| emnc.unz | unz |  | Normal vector up direction, z component | Boundaries 35–39, 45 |
| emnc.dnx | dnx |  | Normal vector down direction, x component | Boundaries 35–39, 45 |
| emnc.dny | dny |  | Normal vector down direction, y component | Boundaries 35–39, 45 |
| emnc.dnz | dnz |  | Normal vector down direction, z component | Boundaries 35–39, 45 |
| emnc.W | emnc.Wm | J/m^3 | Energy density | Domain 7 |
| emnc.dWm | emnc.Wm | J/m^3 | Integrand for total magnetic energy | Domain 7 |
| emnc.Wm | 0.5\*mu0\_const\*((emnc.murxx\*emnc.Hx+emnc.murxy\*emnc.Hy+emnc.murxz\*emnc.Hz)\*emnc.Hx+(emnc.muryx\*emnc.Hx+emnc.muryy\*emnc.Hy+emnc.muryz\*emnc.Hz)\*emnc.Hy+(emnc.murzx\*emnc.Hx+emnc.murzy\*emnc.Hy+emnc.murzz\*emnc.Hz)\*emnc.Hz) | J/m^3 | Magnetic energy density | Domain 7 |

##### Shape Functions

| Name | Shape function | Unit | Description | Shape frame | Selection |
| --- | --- | --- | --- | --- | --- |
| Vm | Lagrange (Quadratic) | A | Magnetic scalar potential | Material | Domain 7 |

##### Weak Expressions

| Weak expression | Integration frame | Selection |
| --- | --- | --- |
| emnc.d\*(-emnc.Bx\*test(Vmx)-emnc.By\*test(Vmy)-emnc.Bz\*test(Vmz)) | Material | Domain 7 |

#### 2.4.20. Magnetic Flux Conservation 18

Magnetic Flux Conservation 18

Selection

| Geometric entity level | Domain |
| Selection | Domain 9 |

Equations

Settings

| Description | Value |
| Constitutive relation | Remanent flux density |
| Remanent flux density, x component | BpRem\*cos(argBp\*(-1 + 17) + tick) |
| Remanent flux density, y component | BpRem\*sin(argBp\*(-1 + 17) + tick) |
| Remanent flux density, z component | 0 |
| Relative permeability | From material |
| Relative permeability | {{1, 0, 0}, {0, 1, 0}, {0, 0, 1}} |

Properties from material

| Property | Material | Property group |
| Relative permeability | Soft Iron (without losses) | Basic |

##### Variables

| Name | Expression | Unit | Description | Selection |
| --- | --- | --- | --- | --- |
| emnc.dnTmx | -0.5\*emnc.unx\*(real(down(emnc.Bx))\*real(down(emnc.Hx))+real(down(emnc.By))\*real(down(emnc.Hy))+real(down(emnc.Bz))\*real(down(emnc.Hz)))+real(down(emnc.Bx))\*(real(down(emnc.Hx))\*emnc.unx+real(down(emnc.Hy))\*emnc.uny+real(down(emnc.Hz))\*emnc.unz) | Pa | Maxwell downward magnetic surface stress tensor, x component | Boundaries 47–50, 55, 57 |
| emnc.dnTmy | -0.5\*emnc.uny\*(real(down(emnc.Bx))\*real(down(emnc.Hx))+real(down(emnc.By))\*real(down(emnc.Hy))+real(down(emnc.Bz))\*real(down(emnc.Hz)))+real(down(emnc.By))\*(real(down(emnc.Hx))\*emnc.unx+real(down(emnc.Hy))\*emnc.uny+real(down(emnc.Hz))\*emnc.unz) | Pa | Maxwell downward magnetic surface stress tensor, y component | Boundaries 47–50, 55, 57 |
| emnc.dnTmz | -0.5\*emnc.unz\*(real(down(emnc.Bx))\*real(down(emnc.Hx))+real(down(emnc.By))\*real(down(emnc.Hy))+real(down(emnc.Bz))\*real(down(emnc.Hz)))+real(down(emnc.Bz))\*(real(down(emnc.Hx))\*emnc.unx+real(down(emnc.Hy))\*emnc.uny+real(down(emnc.Hz))\*emnc.unz) | Pa | Maxwell downward magnetic surface stress tensor, z component | Boundaries 47–50, 55, 57 |
| emnc.Hx | -Vmx | A/m | Magnetic field, x component | Domain 9 |
| emnc.Hy | -Vmy | A/m | Magnetic field, y component | Domain 9 |
| emnc.Hz | -Vmz | A/m | Magnetic field, z component | Domain 9 |
| emnc.tHx | -VmTx | A/m | Tangential magnetic field, x component | Boundaries 47–50, 55, 57 |
| emnc.tHy | -VmTy | A/m | Tangential magnetic field, y component | Boundaries 47–50, 55, 57 |
| emnc.tHz | -VmTz | A/m | Tangential magnetic field, z component | Boundaries 47–50, 55, 57 |
| emnc.normH | sqrt(realdot(emnc.Hx,emnc.Hx)+realdot(emnc.Hy,emnc.Hy)+realdot(emnc.Hz,emnc.Hz)) | A/m | Magnetic field norm | Domain 9 |
| emnc.murxx | model.input.mur11 | 1 | Relative permeability, xx component | Domain 9 |
| emnc.muryx | model.input.mur21 | 1 | Relative permeability, yx component | Domain 9 |
| emnc.murzx | model.input.mur31 | 1 | Relative permeability, zx component | Domain 9 |
| emnc.murxy | model.input.mur12 | 1 | Relative permeability, xy component | Domain 9 |
| emnc.muryy | model.input.mur22 | 1 | Relative permeability, yy component | Domain 9 |
| emnc.murzy | model.input.mur32 | 1 | Relative permeability, zy component | Domain 9 |
| emnc.murxz | model.input.mur13 | 1 | Relative permeability, xz component | Domain 9 |
| emnc.muryz | model.input.mur23 | 1 | Relative permeability, yz component | Domain 9 |
| emnc.murzz | model.input.mur33 | 1 | Relative permeability, zz component | Domain 9 |
| emnc.Bx | mu0\_const\*emnc.Ixx\*emnc.Hx+mu0\_const\*emnc.Ixy\*emnc.Hy+mu0\_const\*emnc.Ixz\*emnc.Hz+mu0\_const\*emnc.chimxx\*emnc.Hx+mu0\_const\*emnc.chimxy\*emnc.Hy+mu0\_const\*emnc.chimxz\*emnc.Hz+emnc.Brx | T | Magnetic flux density, x component | Domain 9 |
| emnc.By | mu0\_const\*emnc.Iyx\*emnc.Hx+mu0\_const\*emnc.Iyy\*emnc.Hy+mu0\_const\*emnc.Iyz\*emnc.Hz+mu0\_const\*emnc.chimyx\*emnc.Hx+mu0\_const\*emnc.chimyy\*emnc.Hy+mu0\_const\*emnc.chimyz\*emnc.Hz+emnc.Bry | T | Magnetic flux density, y component | Domain 9 |
| emnc.Bz | mu0\_const\*emnc.Izx\*emnc.Hx+mu0\_const\*emnc.Izy\*emnc.Hy+mu0\_const\*emnc.Izz\*emnc.Hz+mu0\_const\*emnc.chimzx\*emnc.Hx+mu0\_const\*emnc.chimzy\*emnc.Hy+mu0\_const\*emnc.chimzz\*emnc.Hz+emnc.Brz | T | Magnetic flux density, z component | Domain 9 |
| emnc.normB | sqrt(realdot(emnc.Bx,emnc.Bx)+realdot(emnc.By,emnc.By)+realdot(emnc.Bz,emnc.Bz)) | T | Magnetic flux density norm | Domain 9 |
| emnc.Mx | emnc.Bx/mu0\_const-emnc.Ixx\*emnc.Hx-emnc.Ixy\*emnc.Hy-emnc.Ixz\*emnc.Hz | A/m | Magnetization, x component | Domain 9 |
| emnc.My | emnc.By/mu0\_const-emnc.Iyx\*emnc.Hx-emnc.Iyy\*emnc.Hy-emnc.Iyz\*emnc.Hz | A/m | Magnetization, y component | Domain 9 |
| emnc.Mz | emnc.Bz/mu0\_const-emnc.Izx\*emnc.Hx-emnc.Izy\*emnc.Hy-emnc.Izz\*emnc.Hz | A/m | Magnetization, z component | Domain 9 |
| emnc.normM | sqrt(realdot(emnc.Mx,emnc.Mx)+realdot(emnc.My,emnc.My)+realdot(emnc.Mz,emnc.Mz)) | A/m | Magnetization norm | Domain 9 |
| emnc.Ixx | 1 | 1 | Spatial identity matrix, xx component | Domain 9 |
| emnc.Iyx | 0 | 1 | Spatial identity matrix, yx component | Domain 9 |
| emnc.Izx | 0 | 1 | Spatial identity matrix, zx component | Domain 9 |
| emnc.Ixy | 0 | 1 | Spatial identity matrix, xy component | Domain 9 |
| emnc.Iyy | 1 | 1 | Spatial identity matrix, yy component | Domain 9 |
| emnc.Izy | 0 | 1 | Spatial identity matrix, zy component | Domain 9 |
| emnc.Ixz | 0 | 1 | Spatial identity matrix, xz component | Domain 9 |
| emnc.Iyz | 0 | 1 | Spatial identity matrix, yz component | Domain 9 |
| emnc.Izz | 1 | 1 | Spatial identity matrix, zz component | Domain 9 |
| emnc.Brx | BpRem\*cos(16\*argBp+tick) | T | Remanent flux density, x component | Domain 9 |
| emnc.Bry | BpRem\*sin(16\*argBp+tick) | T | Remanent flux density, y component | Domain 9 |
| emnc.Brz | 0 | T | Remanent flux density, z component | Domain 9 |
| emnc.normBr | sqrt(realdot(emnc.Brx,emnc.Brx)+realdot(emnc.Bry,emnc.Bry)+realdot(emnc.Brz,emnc.Brz)) | T | Remanent flux density norm | Domain 9 |
| emnc.chimxx | -1+emnc.murxx | 1 | Magnetic susceptibility, xx component | Domain 9 |
| emnc.chimyx | emnc.muryx | 1 | Magnetic susceptibility, yx component | Domain 9 |
| emnc.chimzx | emnc.murzx | 1 | Magnetic susceptibility, zx component | Domain 9 |
| emnc.chimxy | emnc.murxy | 1 | Magnetic susceptibility, xy component | Domain 9 |
| emnc.chimyy | -1+emnc.muryy | 1 | Magnetic susceptibility, yy component | Domain 9 |
| emnc.chimzy | emnc.murzy | 1 | Magnetic susceptibility, zy component | Domain 9 |
| emnc.chimxz | emnc.murxz | 1 | Magnetic susceptibility, xz component | Domain 9 |
| emnc.chimyz | emnc.muryz | 1 | Magnetic susceptibility, yz component | Domain 9 |
| emnc.chimzz | -1+emnc.murzz | 1 | Magnetic susceptibility, zz component | Domain 9 |
| emnc.unTx | 0 | Pa | Maxwell upward surface stress tensor, x component | Boundaries 47–50, 55, 57 |
| emnc.unTy | 0 | Pa | Maxwell upward surface stress tensor, y component | Boundaries 47–50, 55, 57 |
| emnc.unTz | 0 | Pa | Maxwell upward surface stress tensor, z component | Boundaries 47–50, 55, 57 |
| emnc.dnTx | emnc.dnTmx | Pa | Maxwell downward surface stress tensor, x component | Boundaries 47–50, 55, 57 |
| emnc.dnTy | emnc.dnTmy | Pa | Maxwell downward surface stress tensor, y component | Boundaries 47–50, 55, 57 |
| emnc.dnTz | emnc.dnTmz | Pa | Maxwell downward surface stress tensor, z component | Boundaries 47–50, 55, 57 |
| emnc.unx | unx |  | Normal vector up direction, x component | Boundaries 47–50, 55, 57 |
| emnc.uny | uny |  | Normal vector up direction, y component | Boundaries 47–50, 55, 57 |
| emnc.unz | unz |  | Normal vector up direction, z component | Boundaries 47–50, 55, 57 |
| emnc.dnx | dnx |  | Normal vector down direction, x component | Boundaries 47–50, 55, 57 |
| emnc.dny | dny |  | Normal vector down direction, y component | Boundaries 47–50, 55, 57 |
| emnc.dnz | dnz |  | Normal vector down direction, z component | Boundaries 47–50, 55, 57 |
| emnc.W | emnc.Wm | J/m^3 | Energy density | Domain 9 |
| emnc.dWm | emnc.Wm | J/m^3 | Integrand for total magnetic energy | Domain 9 |
| emnc.Wm | 0.5\*mu0\_const\*((emnc.murxx\*emnc.Hx+emnc.murxy\*emnc.Hy+emnc.murxz\*emnc.Hz)\*emnc.Hx+(emnc.muryx\*emnc.Hx+emnc.muryy\*emnc.Hy+emnc.muryz\*emnc.Hz)\*emnc.Hy+(emnc.murzx\*emnc.Hx+emnc.murzy\*emnc.Hy+emnc.murzz\*emnc.Hz)\*emnc.Hz) | J/m^3 | Magnetic energy density | Domain 9 |

##### Shape Functions

| Name | Shape function | Unit | Description | Shape frame | Selection |
| --- | --- | --- | --- | --- | --- |
| Vm | Lagrange (Quadratic) | A | Magnetic scalar potential | Material | Domain 9 |

##### Weak Expressions

| Weak expression | Integration frame | Selection |
| --- | --- | --- |
| emnc.d\*(-emnc.Bx\*test(Vmx)-emnc.By\*test(Vmy)-emnc.Bz\*test(Vmz)) | Material | Domain 9 |

#### 2.4.21. Magnetic Flux Conservation 19

Magnetic Flux Conservation 19

Selection

| Geometric entity level | Domain |
| Selection | Domain 11 |

Equations

Settings

| Description | Value |
| Constitutive relation | Remanent flux density |
| Remanent flux density, x component | BpRem\*cos(argBp\*(-1 + 18) + tick) |
| Remanent flux density, y component | BpRem\*sin(argBp\*(-1 + 18) + tick) |
| Remanent flux density, z component | 0 |
| Relative permeability | From material |
| Relative permeability | {{1, 0, 0}, {0, 1, 0}, {0, 0, 1}} |

Properties from material

| Property | Material | Property group |
| Relative permeability | Soft Iron (without losses) | Basic |

##### Variables

| Name | Expression | Unit | Description | Selection |
| --- | --- | --- | --- | --- |
| emnc.dnTmx | -0.5\*emnc.unx\*(real(down(emnc.Bx))\*real(down(emnc.Hx))+real(down(emnc.By))\*real(down(emnc.Hy))+real(down(emnc.Bz))\*real(down(emnc.Hz)))+real(down(emnc.Bx))\*(real(down(emnc.Hx))\*emnc.unx+real(down(emnc.Hy))\*emnc.uny+real(down(emnc.Hz))\*emnc.unz) | Pa | Maxwell downward magnetic surface stress tensor, x component | Boundaries 59–62, 67, 69 |
| emnc.dnTmy | -0.5\*emnc.uny\*(real(down(emnc.Bx))\*real(down(emnc.Hx))+real(down(emnc.By))\*real(down(emnc.Hy))+real(down(emnc.Bz))\*real(down(emnc.Hz)))+real(down(emnc.By))\*(real(down(emnc.Hx))\*emnc.unx+real(down(emnc.Hy))\*emnc.uny+real(down(emnc.Hz))\*emnc.unz) | Pa | Maxwell downward magnetic surface stress tensor, y component | Boundaries 59–62, 67, 69 |
| emnc.dnTmz | -0.5\*emnc.unz\*(real(down(emnc.Bx))\*real(down(emnc.Hx))+real(down(emnc.By))\*real(down(emnc.Hy))+real(down(emnc.Bz))\*real(down(emnc.Hz)))+real(down(emnc.Bz))\*(real(down(emnc.Hx))\*emnc.unx+real(down(emnc.Hy))\*emnc.uny+real(down(emnc.Hz))\*emnc.unz) | Pa | Maxwell downward magnetic surface stress tensor, z component | Boundaries 59–62, 67, 69 |
| emnc.Hx | -Vmx | A/m | Magnetic field, x component | Domain 11 |
| emnc.Hy | -Vmy | A/m | Magnetic field, y component | Domain 11 |
| emnc.Hz | -Vmz | A/m | Magnetic field, z component | Domain 11 |
| emnc.tHx | -VmTx | A/m | Tangential magnetic field, x component | Boundaries 59–62, 67, 69 |
| emnc.tHy | -VmTy | A/m | Tangential magnetic field, y component | Boundaries 59–62, 67, 69 |
| emnc.tHz | -VmTz | A/m | Tangential magnetic field, z component | Boundaries 59–62, 67, 69 |
| emnc.normH | sqrt(realdot(emnc.Hx,emnc.Hx)+realdot(emnc.Hy,emnc.Hy)+realdot(emnc.Hz,emnc.Hz)) | A/m | Magnetic field norm | Domain 11 |
| emnc.murxx | model.input.mur11 | 1 | Relative permeability, xx component | Domain 11 |
| emnc.muryx | model.input.mur21 | 1 | Relative permeability, yx component | Domain 11 |
| emnc.murzx | model.input.mur31 | 1 | Relative permeability, zx component | Domain 11 |
| emnc.murxy | model.input.mur12 | 1 | Relative permeability, xy component | Domain 11 |
| emnc.muryy | model.input.mur22 | 1 | Relative permeability, yy component | Domain 11 |
| emnc.murzy | model.input.mur32 | 1 | Relative permeability, zy component | Domain 11 |
| emnc.murxz | model.input.mur13 | 1 | Relative permeability, xz component | Domain 11 |
| emnc.muryz | model.input.mur23 | 1 | Relative permeability, yz component | Domain 11 |
| emnc.murzz | model.input.mur33 | 1 | Relative permeability, zz component | Domain 11 |
| emnc.Bx | mu0\_const\*emnc.Ixx\*emnc.Hx+mu0\_const\*emnc.Ixy\*emnc.Hy+mu0\_const\*emnc.Ixz\*emnc.Hz+mu0\_const\*emnc.chimxx\*emnc.Hx+mu0\_const\*emnc.chimxy\*emnc.Hy+mu0\_const\*emnc.chimxz\*emnc.Hz+emnc.Brx | T | Magnetic flux density, x component | Domain 11 |
| emnc.By | mu0\_const\*emnc.Iyx\*emnc.Hx+mu0\_const\*emnc.Iyy\*emnc.Hy+mu0\_const\*emnc.Iyz\*emnc.Hz+mu0\_const\*emnc.chimyx\*emnc.Hx+mu0\_const\*emnc.chimyy\*emnc.Hy+mu0\_const\*emnc.chimyz\*emnc.Hz+emnc.Bry | T | Magnetic flux density, y component | Domain 11 |
| emnc.Bz | mu0\_const\*emnc.Izx\*emnc.Hx+mu0\_const\*emnc.Izy\*emnc.Hy+mu0\_const\*emnc.Izz\*emnc.Hz+mu0\_const\*emnc.chimzx\*emnc.Hx+mu0\_const\*emnc.chimzy\*emnc.Hy+mu0\_const\*emnc.chimzz\*emnc.Hz+emnc.Brz | T | Magnetic flux density, z component | Domain 11 |
| emnc.normB | sqrt(realdot(emnc.Bx,emnc.Bx)+realdot(emnc.By,emnc.By)+realdot(emnc.Bz,emnc.Bz)) | T | Magnetic flux density norm | Domain 11 |
| emnc.Mx | emnc.Bx/mu0\_const-emnc.Ixx\*emnc.Hx-emnc.Ixy\*emnc.Hy-emnc.Ixz\*emnc.Hz | A/m | Magnetization, x component | Domain 11 |
| emnc.My | emnc.By/mu0\_const-emnc.Iyx\*emnc.Hx-emnc.Iyy\*emnc.Hy-emnc.Iyz\*emnc.Hz | A/m | Magnetization, y component | Domain 11 |
| emnc.Mz | emnc.Bz/mu0\_const-emnc.Izx\*emnc.Hx-emnc.Izy\*emnc.Hy-emnc.Izz\*emnc.Hz | A/m | Magnetization, z component | Domain 11 |
| emnc.normM | sqrt(realdot(emnc.Mx,emnc.Mx)+realdot(emnc.My,emnc.My)+realdot(emnc.Mz,emnc.Mz)) | A/m | Magnetization norm | Domain 11 |
| emnc.Ixx | 1 | 1 | Spatial identity matrix, xx component | Domain 11 |
| emnc.Iyx | 0 | 1 | Spatial identity matrix, yx component | Domain 11 |
| emnc.Izx | 0 | 1 | Spatial identity matrix, zx component | Domain 11 |
| emnc.Ixy | 0 | 1 | Spatial identity matrix, xy component | Domain 11 |
| emnc.Iyy | 1 | 1 | Spatial identity matrix, yy component | Domain 11 |
| emnc.Izy | 0 | 1 | Spatial identity matrix, zy component | Domain 11 |
| emnc.Ixz | 0 | 1 | Spatial identity matrix, xz component | Domain 11 |
| emnc.Iyz | 0 | 1 | Spatial identity matrix, yz component | Domain 11 |
| emnc.Izz | 1 | 1 | Spatial identity matrix, zz component | Domain 11 |
| emnc.Brx | BpRem\*cos(17\*argBp+tick) | T | Remanent flux density, x component | Domain 11 |
| emnc.Bry | BpRem\*sin(17\*argBp+tick) | T | Remanent flux density, y component | Domain 11 |
| emnc.Brz | 0 | T | Remanent flux density, z component | Domain 11 |
| emnc.normBr | sqrt(realdot(emnc.Brx,emnc.Brx)+realdot(emnc.Bry,emnc.Bry)+realdot(emnc.Brz,emnc.Brz)) | T | Remanent flux density norm | Domain 11 |
| emnc.chimxx | -1+emnc.murxx | 1 | Magnetic susceptibility, xx component | Domain 11 |
| emnc.chimyx | emnc.muryx | 1 | Magnetic susceptibility, yx component | Domain 11 |
| emnc.chimzx | emnc.murzx | 1 | Magnetic susceptibility, zx component | Domain 11 |
| emnc.chimxy | emnc.murxy | 1 | Magnetic susceptibility, xy component | Domain 11 |
| emnc.chimyy | -1+emnc.muryy | 1 | Magnetic susceptibility, yy component | Domain 11 |
| emnc.chimzy | emnc.murzy | 1 | Magnetic susceptibility, zy component | Domain 11 |
| emnc.chimxz | emnc.murxz | 1 | Magnetic susceptibility, xz component | Domain 11 |
| emnc.chimyz | emnc.muryz | 1 | Magnetic susceptibility, yz component | Domain 11 |
| emnc.chimzz | -1+emnc.murzz | 1 | Magnetic susceptibility, zz component | Domain 11 |
| emnc.unTx | 0 | Pa | Maxwell upward surface stress tensor, x component | Boundaries 59–62, 67, 69 |
| emnc.unTy | 0 | Pa | Maxwell upward surface stress tensor, y component | Boundaries 59–62, 67, 69 |
| emnc.unTz | 0 | Pa | Maxwell upward surface stress tensor, z component | Boundaries 59–62, 67, 69 |
| emnc.dnTx | emnc.dnTmx | Pa | Maxwell downward surface stress tensor, x component | Boundaries 59–62, 67, 69 |
| emnc.dnTy | emnc.dnTmy | Pa | Maxwell downward surface stress tensor, y component | Boundaries 59–62, 67, 69 |
| emnc.dnTz | emnc.dnTmz | Pa | Maxwell downward surface stress tensor, z component | Boundaries 59–62, 67, 69 |
| emnc.unx | unx |  | Normal vector up direction, x component | Boundaries 59–62, 67, 69 |
| emnc.uny | uny |  | Normal vector up direction, y component | Boundaries 59–62, 67, 69 |
| emnc.unz | unz |  | Normal vector up direction, z component | Boundaries 59–62, 67, 69 |
| emnc.dnx | dnx |  | Normal vector down direction, x component | Boundaries 59–62, 67, 69 |
| emnc.dny | dny |  | Normal vector down direction, y component | Boundaries 59–62, 67, 69 |
| emnc.dnz | dnz |  | Normal vector down direction, z component | Boundaries 59–62, 67, 69 |
| emnc.W | emnc.Wm | J/m^3 | Energy density | Domain 11 |
| emnc.dWm | emnc.Wm | J/m^3 | Integrand for total magnetic energy | Domain 11 |
| emnc.Wm | 0.5\*mu0\_const\*((emnc.murxx\*emnc.Hx+emnc.murxy\*emnc.Hy+emnc.murxz\*emnc.Hz)\*emnc.Hx+(emnc.muryx\*emnc.Hx+emnc.muryy\*emnc.Hy+emnc.muryz\*emnc.Hz)\*emnc.Hy+(emnc.murzx\*emnc.Hx+emnc.murzy\*emnc.Hy+emnc.murzz\*emnc.Hz)\*emnc.Hz) | J/m^3 | Magnetic energy density | Domain 11 |

##### Shape Functions

| Name | Shape function | Unit | Description | Shape frame | Selection |
| --- | --- | --- | --- | --- | --- |
| Vm | Lagrange (Quadratic) | A | Magnetic scalar potential | Material | Domain 11 |

##### Weak Expressions

| Weak expression | Integration frame | Selection |
| --- | --- | --- |
| emnc.d\*(-emnc.Bx\*test(Vmx)-emnc.By\*test(Vmy)-emnc.Bz\*test(Vmz)) | Material | Domain 11 |

#### 2.4.22. Magnetic Flux Conservation 20

Magnetic Flux Conservation 20

Selection

| Geometric entity level | Domain |
| Selection | Domain 14 |

Equations

Settings

| Description | Value |
| Constitutive relation | Remanent flux density |
| Remanent flux density, x component | BpRem\*cos(argBp\*(-1 + 19) + tick) |
| Remanent flux density, y component | BpRem\*sin(argBp\*(-1 + 19) + tick) |
| Remanent flux density, z component | 0 |
| Relative permeability | From material |
| Relative permeability | {{1, 0, 0}, {0, 1, 0}, {0, 0, 1}} |

Properties from material

| Property | Material | Property group |
| Relative permeability | Soft Iron (without losses) | Basic |

##### Variables

| Name | Expression | Unit | Description | Selection |
| --- | --- | --- | --- | --- |
| emnc.dnTmx | -0.5\*emnc.unx\*(real(down(emnc.Bx))\*real(down(emnc.Hx))+real(down(emnc.By))\*real(down(emnc.Hy))+real(down(emnc.Bz))\*real(down(emnc.Hz)))+real(down(emnc.Bx))\*(real(down(emnc.Hx))\*emnc.unx+real(down(emnc.Hy))\*emnc.uny+real(down(emnc.Hz))\*emnc.unz) | Pa | Maxwell downward magnetic surface stress tensor, x component | Boundaries 75–78, 84–85 |
| emnc.dnTmy | -0.5\*emnc.uny\*(real(down(emnc.Bx))\*real(down(emnc.Hx))+real(down(emnc.By))\*real(down(emnc.Hy))+real(down(emnc.Bz))\*real(down(emnc.Hz)))+real(down(emnc.By))\*(real(down(emnc.Hx))\*emnc.unx+real(down(emnc.Hy))\*emnc.uny+real(down(emnc.Hz))\*emnc.unz) | Pa | Maxwell downward magnetic surface stress tensor, y component | Boundaries 75–78, 84–85 |
| emnc.dnTmz | -0.5\*emnc.unz\*(real(down(emnc.Bx))\*real(down(emnc.Hx))+real(down(emnc.By))\*real(down(emnc.Hy))+real(down(emnc.Bz))\*real(down(emnc.Hz)))+real(down(emnc.Bz))\*(real(down(emnc.Hx))\*emnc.unx+real(down(emnc.Hy))\*emnc.uny+real(down(emnc.Hz))\*emnc.unz) | Pa | Maxwell downward magnetic surface stress tensor, z component | Boundaries 75–78, 84–85 |
| emnc.Hx | -Vmx | A/m | Magnetic field, x component | Domain 14 |
| emnc.Hy | -Vmy | A/m | Magnetic field, y component | Domain 14 |
| emnc.Hz | -Vmz | A/m | Magnetic field, z component | Domain 14 |
| emnc.tHx | -VmTx | A/m | Tangential magnetic field, x component | Boundaries 75–78, 84–85 |
| emnc.tHy | -VmTy | A/m | Tangential magnetic field, y component | Boundaries 75–78, 84–85 |
| emnc.tHz | -VmTz | A/m | Tangential magnetic field, z component | Boundaries 75–78, 84–85 |
| emnc.normH | sqrt(realdot(emnc.Hx,emnc.Hx)+realdot(emnc.Hy,emnc.Hy)+realdot(emnc.Hz,emnc.Hz)) | A/m | Magnetic field norm | Domain 14 |
| emnc.murxx | model.input.mur11 | 1 | Relative permeability, xx component | Domain 14 |
| emnc.muryx | model.input.mur21 | 1 | Relative permeability, yx component | Domain 14 |
| emnc.murzx | model.input.mur31 | 1 | Relative permeability, zx component | Domain 14 |
| emnc.murxy | model.input.mur12 | 1 | Relative permeability, xy component | Domain 14 |
| emnc.muryy | model.input.mur22 | 1 | Relative permeability, yy component | Domain 14 |
| emnc.murzy | model.input.mur32 | 1 | Relative permeability, zy component | Domain 14 |
| emnc.murxz | model.input.mur13 | 1 | Relative permeability, xz component | Domain 14 |
| emnc.muryz | model.input.mur23 | 1 | Relative permeability, yz component | Domain 14 |
| emnc.murzz | model.input.mur33 | 1 | Relative permeability, zz component | Domain 14 |
| emnc.Bx | mu0\_const\*emnc.Ixx\*emnc.Hx+mu0\_const\*emnc.Ixy\*emnc.Hy+mu0\_const\*emnc.Ixz\*emnc.Hz+mu0\_const\*emnc.chimxx\*emnc.Hx+mu0\_const\*emnc.chimxy\*emnc.Hy+mu0\_const\*emnc.chimxz\*emnc.Hz+emnc.Brx | T | Magnetic flux density, x component | Domain 14 |
| emnc.By | mu0\_const\*emnc.Iyx\*emnc.Hx+mu0\_const\*emnc.Iyy\*emnc.Hy+mu0\_const\*emnc.Iyz\*emnc.Hz+mu0\_const\*emnc.chimyx\*emnc.Hx+mu0\_const\*emnc.chimyy\*emnc.Hy+mu0\_const\*emnc.chimyz\*emnc.Hz+emnc.Bry | T | Magnetic flux density, y component | Domain 14 |
| emnc.Bz | mu0\_const\*emnc.Izx\*emnc.Hx+mu0\_const\*emnc.Izy\*emnc.Hy+mu0\_const\*emnc.Izz\*emnc.Hz+mu0\_const\*emnc.chimzx\*emnc.Hx+mu0\_const\*emnc.chimzy\*emnc.Hy+mu0\_const\*emnc.chimzz\*emnc.Hz+emnc.Brz | T | Magnetic flux density, z component | Domain 14 |
| emnc.normB | sqrt(realdot(emnc.Bx,emnc.Bx)+realdot(emnc.By,emnc.By)+realdot(emnc.Bz,emnc.Bz)) | T | Magnetic flux density norm | Domain 14 |
| emnc.Mx | emnc.Bx/mu0\_const-emnc.Ixx\*emnc.Hx-emnc.Ixy\*emnc.Hy-emnc.Ixz\*emnc.Hz | A/m | Magnetization, x component | Domain 14 |
| emnc.My | emnc.By/mu0\_const-emnc.Iyx\*emnc.Hx-emnc.Iyy\*emnc.Hy-emnc.Iyz\*emnc.Hz | A/m | Magnetization, y component | Domain 14 |
| emnc.Mz | emnc.Bz/mu0\_const-emnc.Izx\*emnc.Hx-emnc.Izy\*emnc.Hy-emnc.Izz\*emnc.Hz | A/m | Magnetization, z component | Domain 14 |
| emnc.normM | sqrt(realdot(emnc.Mx,emnc.Mx)+realdot(emnc.My,emnc.My)+realdot(emnc.Mz,emnc.Mz)) | A/m | Magnetization norm | Domain 14 |
| emnc.Ixx | 1 | 1 | Spatial identity matrix, xx component | Domain 14 |
| emnc.Iyx | 0 | 1 | Spatial identity matrix, yx component | Domain 14 |
| emnc.Izx | 0 | 1 | Spatial identity matrix, zx component | Domain 14 |
| emnc.Ixy | 0 | 1 | Spatial identity matrix, xy component | Domain 14 |
| emnc.Iyy | 1 | 1 | Spatial identity matrix, yy component | Domain 14 |
| emnc.Izy | 0 | 1 | Spatial identity matrix, zy component | Domain 14 |
| emnc.Ixz | 0 | 1 | Spatial identity matrix, xz component | Domain 14 |
| emnc.Iyz | 0 | 1 | Spatial identity matrix, yz component | Domain 14 |
| emnc.Izz | 1 | 1 | Spatial identity matrix, zz component | Domain 14 |
| emnc.Brx | BpRem\*cos(18\*argBp+tick) | T | Remanent flux density, x component | Domain 14 |
| emnc.Bry | BpRem\*sin(18\*argBp+tick) | T | Remanent flux density, y component | Domain 14 |
| emnc.Brz | 0 | T | Remanent flux density, z component | Domain 14 |
| emnc.normBr | sqrt(realdot(emnc.Brx,emnc.Brx)+realdot(emnc.Bry,emnc.Bry)+realdot(emnc.Brz,emnc.Brz)) | T | Remanent flux density norm | Domain 14 |
| emnc.chimxx | -1+emnc.murxx | 1 | Magnetic susceptibility, xx component | Domain 14 |
| emnc.chimyx | emnc.muryx | 1 | Magnetic susceptibility, yx component | Domain 14 |
| emnc.chimzx | emnc.murzx | 1 | Magnetic susceptibility, zx component | Domain 14 |
| emnc.chimxy | emnc.murxy | 1 | Magnetic susceptibility, xy component | Domain 14 |
| emnc.chimyy | -1+emnc.muryy | 1 | Magnetic susceptibility, yy component | Domain 14 |
| emnc.chimzy | emnc.murzy | 1 | Magnetic susceptibility, zy component | Domain 14 |
| emnc.chimxz | emnc.murxz | 1 | Magnetic susceptibility, xz component | Domain 14 |
| emnc.chimyz | emnc.muryz | 1 | Magnetic susceptibility, yz component | Domain 14 |
| emnc.chimzz | -1+emnc.murzz | 1 | Magnetic susceptibility, zz component | Domain 14 |
| emnc.unTx | 0 | Pa | Maxwell upward surface stress tensor, x component | Boundaries 75–78, 84–85 |
| emnc.unTy | 0 | Pa | Maxwell upward surface stress tensor, y component | Boundaries 75–78, 84–85 |
| emnc.unTz | 0 | Pa | Maxwell upward surface stress tensor, z component | Boundaries 75–78, 84–85 |
| emnc.dnTx | emnc.dnTmx | Pa | Maxwell downward surface stress tensor, x component | Boundaries 75–78, 84–85 |
| emnc.dnTy | emnc.dnTmy | Pa | Maxwell downward surface stress tensor, y component | Boundaries 75–78, 84–85 |
| emnc.dnTz | emnc.dnTmz | Pa | Maxwell downward surface stress tensor, z component | Boundaries 75–78, 84–85 |
| emnc.unx | unx |  | Normal vector up direction, x component | Boundaries 75–78, 84–85 |
| emnc.uny | uny |  | Normal vector up direction, y component | Boundaries 75–78, 84–85 |
| emnc.unz | unz |  | Normal vector up direction, z component | Boundaries 75–78, 84–85 |
| emnc.dnx | dnx |  | Normal vector down direction, x component | Boundaries 75–78, 84–85 |
| emnc.dny | dny |  | Normal vector down direction, y component | Boundaries 75–78, 84–85 |
| emnc.dnz | dnz |  | Normal vector down direction, z component | Boundaries 75–78, 84–85 |
| emnc.W | emnc.Wm | J/m^3 | Energy density | Domain 14 |
| emnc.dWm | emnc.Wm | J/m^3 | Integrand for total magnetic energy | Domain 14 |
| emnc.Wm | 0.5\*mu0\_const\*((emnc.murxx\*emnc.Hx+emnc.murxy\*emnc.Hy+emnc.murxz\*emnc.Hz)\*emnc.Hx+(emnc.muryx\*emnc.Hx+emnc.muryy\*emnc.Hy+emnc.muryz\*emnc.Hz)\*emnc.Hy+(emnc.murzx\*emnc.Hx+emnc.murzy\*emnc.Hy+emnc.murzz\*emnc.Hz)\*emnc.Hz) | J/m^3 | Magnetic energy density | Domain 14 |

##### Shape Functions

| Name | Shape function | Unit | Description | Shape frame | Selection |
| --- | --- | --- | --- | --- | --- |
| Vm | Lagrange (Quadratic) | A | Magnetic scalar potential | Material | Domain 14 |

##### Weak Expressions

| Weak expression | Integration frame | Selection |
| --- | --- | --- |
| emnc.d\*(-emnc.Bx\*test(Vmx)-emnc.By\*test(Vmy)-emnc.Bz\*test(Vmz)) | Material | Domain 14 |

#### 2.4.23. Magnetic Flux Conservation 21

Magnetic Flux Conservation 21

Selection

| Geometric entity level | Domain |
| Selection | Domain 16 |

Equations

Settings

| Description | Value |
| Constitutive relation | Remanent flux density |
| Remanent flux density, x component | BpRem\*cos(argBp\*(-1 + 20) + tick) |
| Remanent flux density, y component | BpRem\*sin(argBp\*(-1 + 20) + tick) |
| Remanent flux density, z component | 0 |
| Relative permeability | From material |
| Relative permeability | {{1, 0, 0}, {0, 1, 0}, {0, 0, 1}} |

Properties from material

| Property | Material | Property group |
| Relative permeability | Soft Iron (without losses) | Basic |

##### Variables

| Name | Expression | Unit | Description | Selection |
| --- | --- | --- | --- | --- |
| emnc.dnTmx | -0.5\*emnc.unx\*(real(down(emnc.Bx))\*real(down(emnc.Hx))+real(down(emnc.By))\*real(down(emnc.Hy))+real(down(emnc.Bz))\*real(down(emnc.Hz)))+real(down(emnc.Bx))\*(real(down(emnc.Hx))\*emnc.unx+real(down(emnc.Hy))\*emnc.uny+real(down(emnc.Hz))\*emnc.unz) | Pa | Maxwell downward magnetic surface stress tensor, x component | Boundaries 93–96, 101, 103 |
| emnc.dnTmy | -0.5\*emnc.uny\*(real(down(emnc.Bx))\*real(down(emnc.Hx))+real(down(emnc.By))\*real(down(emnc.Hy))+real(down(emnc.Bz))\*real(down(emnc.Hz)))+real(down(emnc.By))\*(real(down(emnc.Hx))\*emnc.unx+real(down(emnc.Hy))\*emnc.uny+real(down(emnc.Hz))\*emnc.unz) | Pa | Maxwell downward magnetic surface stress tensor, y component | Boundaries 93–96, 101, 103 |
| emnc.dnTmz | -0.5\*emnc.unz\*(real(down(emnc.Bx))\*real(down(emnc.Hx))+real(down(emnc.By))\*real(down(emnc.Hy))+real(down(emnc.Bz))\*real(down(emnc.Hz)))+real(down(emnc.Bz))\*(real(down(emnc.Hx))\*emnc.unx+real(down(emnc.Hy))\*emnc.uny+real(down(emnc.Hz))\*emnc.unz) | Pa | Maxwell downward magnetic surface stress tensor, z component | Boundaries 93–96, 101, 103 |
| emnc.Hx | -Vmx | A/m | Magnetic field, x component | Domain 16 |
| emnc.Hy | -Vmy | A/m | Magnetic field, y component | Domain 16 |
| emnc.Hz | -Vmz | A/m | Magnetic field, z component | Domain 16 |
| emnc.tHx | -VmTx | A/m | Tangential magnetic field, x component | Boundaries 93–96, 101, 103 |
| emnc.tHy | -VmTy | A/m | Tangential magnetic field, y component | Boundaries 93–96, 101, 103 |
| emnc.tHz | -VmTz | A/m | Tangential magnetic field, z component | Boundaries 93–96, 101, 103 |
| emnc.normH | sqrt(realdot(emnc.Hx,emnc.Hx)+realdot(emnc.Hy,emnc.Hy)+realdot(emnc.Hz,emnc.Hz)) | A/m | Magnetic field norm | Domain 16 |
| emnc.murxx | model.input.mur11 | 1 | Relative permeability, xx component | Domain 16 |
| emnc.muryx | model.input.mur21 | 1 | Relative permeability, yx component | Domain 16 |
| emnc.murzx | model.input.mur31 | 1 | Relative permeability, zx component | Domain 16 |
| emnc.murxy | model.input.mur12 | 1 | Relative permeability, xy component | Domain 16 |
| emnc.muryy | model.input.mur22 | 1 | Relative permeability, yy component | Domain 16 |
| emnc.murzy | model.input.mur32 | 1 | Relative permeability, zy component | Domain 16 |
| emnc.murxz | model.input.mur13 | 1 | Relative permeability, xz component | Domain 16 |
| emnc.muryz | model.input.mur23 | 1 | Relative permeability, yz component | Domain 16 |
| emnc.murzz | model.input.mur33 | 1 | Relative permeability, zz component | Domain 16 |
| emnc.Bx | mu0\_const\*emnc.Ixx\*emnc.Hx+mu0\_const\*emnc.Ixy\*emnc.Hy+mu0\_const\*emnc.Ixz\*emnc.Hz+mu0\_const\*emnc.chimxx\*emnc.Hx+mu0\_const\*emnc.chimxy\*emnc.Hy+mu0\_const\*emnc.chimxz\*emnc.Hz+emnc.Brx | T | Magnetic flux density, x component | Domain 16 |
| emnc.By | mu0\_const\*emnc.Iyx\*emnc.Hx+mu0\_const\*emnc.Iyy\*emnc.Hy+mu0\_const\*emnc.Iyz\*emnc.Hz+mu0\_const\*emnc.chimyx\*emnc.Hx+mu0\_const\*emnc.chimyy\*emnc.Hy+mu0\_const\*emnc.chimyz\*emnc.Hz+emnc.Bry | T | Magnetic flux density, y component | Domain 16 |
| emnc.Bz | mu0\_const\*emnc.Izx\*emnc.Hx+mu0\_const\*emnc.Izy\*emnc.Hy+mu0\_const\*emnc.Izz\*emnc.Hz+mu0\_const\*emnc.chimzx\*emnc.Hx+mu0\_const\*emnc.chimzy\*emnc.Hy+mu0\_const\*emnc.chimzz\*emnc.Hz+emnc.Brz | T | Magnetic flux density, z component | Domain 16 |
| emnc.normB | sqrt(realdot(emnc.Bx,emnc.Bx)+realdot(emnc.By,emnc.By)+realdot(emnc.Bz,emnc.Bz)) | T | Magnetic flux density norm | Domain 16 |
| emnc.Mx | emnc.Bx/mu0\_const-emnc.Ixx\*emnc.Hx-emnc.Ixy\*emnc.Hy-emnc.Ixz\*emnc.Hz | A/m | Magnetization, x component | Domain 16 |
| emnc.My | emnc.By/mu0\_const-emnc.Iyx\*emnc.Hx-emnc.Iyy\*emnc.Hy-emnc.Iyz\*emnc.Hz | A/m | Magnetization, y component | Domain 16 |
| emnc.Mz | emnc.Bz/mu0\_const-emnc.Izx\*emnc.Hx-emnc.Izy\*emnc.Hy-emnc.Izz\*emnc.Hz | A/m | Magnetization, z component | Domain 16 |
| emnc.normM | sqrt(realdot(emnc.Mx,emnc.Mx)+realdot(emnc.My,emnc.My)+realdot(emnc.Mz,emnc.Mz)) | A/m | Magnetization norm | Domain 16 |
| emnc.Ixx | 1 | 1 | Spatial identity matrix, xx component | Domain 16 |
| emnc.Iyx | 0 | 1 | Spatial identity matrix, yx component | Domain 16 |
| emnc.Izx | 0 | 1 | Spatial identity matrix, zx component | Domain 16 |
| emnc.Ixy | 0 | 1 | Spatial identity matrix, xy component | Domain 16 |
| emnc.Iyy | 1 | 1 | Spatial identity matrix, yy component | Domain 16 |
| emnc.Izy | 0 | 1 | Spatial identity matrix, zy component | Domain 16 |
| emnc.Ixz | 0 | 1 | Spatial identity matrix, xz component | Domain 16 |
| emnc.Iyz | 0 | 1 | Spatial identity matrix, yz component | Domain 16 |
| emnc.Izz | 1 | 1 | Spatial identity matrix, zz component | Domain 16 |
| emnc.Brx | BpRem\*cos(19\*argBp+tick) | T | Remanent flux density, x component | Domain 16 |
| emnc.Bry | BpRem\*sin(19\*argBp+tick) | T | Remanent flux density, y component | Domain 16 |
| emnc.Brz | 0 | T | Remanent flux density, z component | Domain 16 |
| emnc.normBr | sqrt(realdot(emnc.Brx,emnc.Brx)+realdot(emnc.Bry,emnc.Bry)+realdot(emnc.Brz,emnc.Brz)) | T | Remanent flux density norm | Domain 16 |
| emnc.chimxx | -1+emnc.murxx | 1 | Magnetic susceptibility, xx component | Domain 16 |
| emnc.chimyx | emnc.muryx | 1 | Magnetic susceptibility, yx component | Domain 16 |
| emnc.chimzx | emnc.murzx | 1 | Magnetic susceptibility, zx component | Domain 16 |
| emnc.chimxy | emnc.murxy | 1 | Magnetic susceptibility, xy component | Domain 16 |
| emnc.chimyy | -1+emnc.muryy | 1 | Magnetic susceptibility, yy component | Domain 16 |
| emnc.chimzy | emnc.murzy | 1 | Magnetic susceptibility, zy component | Domain 16 |
| emnc.chimxz | emnc.murxz | 1 | Magnetic susceptibility, xz component | Domain 16 |
| emnc.chimyz | emnc.muryz | 1 | Magnetic susceptibility, yz component | Domain 16 |
| emnc.chimzz | -1+emnc.murzz | 1 | Magnetic susceptibility, zz component | Domain 16 |
| emnc.unTx | 0 | Pa | Maxwell upward surface stress tensor, x component | Boundaries 93–96, 101, 103 |
| emnc.unTy | 0 | Pa | Maxwell upward surface stress tensor, y component | Boundaries 93–96, 101, 103 |
| emnc.unTz | 0 | Pa | Maxwell upward surface stress tensor, z component | Boundaries 93–96, 101, 103 |
| emnc.dnTx | emnc.dnTmx | Pa | Maxwell downward surface stress tensor, x component | Boundaries 93–96, 101, 103 |
| emnc.dnTy | emnc.dnTmy | Pa | Maxwell downward surface stress tensor, y component | Boundaries 93–96, 101, 103 |
| emnc.dnTz | emnc.dnTmz | Pa | Maxwell downward surface stress tensor, z component | Boundaries 93–96, 101, 103 |
| emnc.unx | unx |  | Normal vector up direction, x component | Boundaries 93–96, 101, 103 |
| emnc.uny | uny |  | Normal vector up direction, y component | Boundaries 93–96, 101, 103 |
| emnc.unz | unz |  | Normal vector up direction, z component | Boundaries 93–96, 101, 103 |
| emnc.dnx | dnx |  | Normal vector down direction, x component | Boundaries 93–96, 101, 103 |
| emnc.dny | dny |  | Normal vector down direction, y component | Boundaries 93–96, 101, 103 |
| emnc.dnz | dnz |  | Normal vector down direction, z component | Boundaries 93–96, 101, 103 |
| emnc.W | emnc.Wm | J/m^3 | Energy density | Domain 16 |
| emnc.dWm | emnc.Wm | J/m^3 | Integrand for total magnetic energy | Domain 16 |
| emnc.Wm | 0.5\*mu0\_const\*((emnc.murxx\*emnc.Hx+emnc.murxy\*emnc.Hy+emnc.murxz\*emnc.Hz)\*emnc.Hx+(emnc.muryx\*emnc.Hx+emnc.muryy\*emnc.Hy+emnc.muryz\*emnc.Hz)\*emnc.Hy+(emnc.murzx\*emnc.Hx+emnc.murzy\*emnc.Hy+emnc.murzz\*emnc.Hz)\*emnc.Hz) | J/m^3 | Magnetic energy density | Domain 16 |

##### Shape Functions

| Name | Shape function | Unit | Description | Shape frame | Selection |
| --- | --- | --- | --- | --- | --- |
| Vm | Lagrange (Quadratic) | A | Magnetic scalar potential | Material | Domain 16 |

##### Weak Expressions

| Weak expression | Integration frame | Selection |
| --- | --- | --- |
| emnc.d\*(-emnc.Bx\*test(Vmx)-emnc.By\*test(Vmy)-emnc.Bz\*test(Vmz)) | Material | Domain 16 |

#### 2.4.24. Magnetic Flux Conservation 22

Magnetic Flux Conservation 22

Selection

| Geometric entity level | Domain |
| Selection | Domain 18 |

Equations

Settings

| Description | Value |
| Constitutive relation | Remanent flux density |
| Remanent flux density, x component | BpRem\*cos(argBp\*(-1 + 21) + tick) |
| Remanent flux density, y component | BpRem\*sin(argBp\*(-1 + 21) + tick) |
| Remanent flux density, z component | 0 |
| Relative permeability | From material |
| Relative permeability | {{1, 0, 0}, {0, 1, 0}, {0, 0, 1}} |

Properties from material

| Property | Material | Property group |
| Relative permeability | Soft Iron (without losses) | Basic |

##### Variables

| Name | Expression | Unit | Description | Selection |
| --- | --- | --- | --- | --- |
| emnc.dnTmx | -0.5\*emnc.unx\*(real(down(emnc.Bx))\*real(down(emnc.Hx))+real(down(emnc.By))\*real(down(emnc.Hy))+real(down(emnc.Bz))\*real(down(emnc.Hz)))+real(down(emnc.Bx))\*(real(down(emnc.Hx))\*emnc.unx+real(down(emnc.Hy))\*emnc.uny+real(down(emnc.Hz))\*emnc.unz) | Pa | Maxwell downward magnetic surface stress tensor, x component | Boundaries 105–108, 113, 115 |
| emnc.dnTmy | -0.5\*emnc.uny\*(real(down(emnc.Bx))\*real(down(emnc.Hx))+real(down(emnc.By))\*real(down(emnc.Hy))+real(down(emnc.Bz))\*real(down(emnc.Hz)))+real(down(emnc.By))\*(real(down(emnc.Hx))\*emnc.unx+real(down(emnc.Hy))\*emnc.uny+real(down(emnc.Hz))\*emnc.unz) | Pa | Maxwell downward magnetic surface stress tensor, y component | Boundaries 105–108, 113, 115 |
| emnc.dnTmz | -0.5\*emnc.unz\*(real(down(emnc.Bx))\*real(down(emnc.Hx))+real(down(emnc.By))\*real(down(emnc.Hy))+real(down(emnc.Bz))\*real(down(emnc.Hz)))+real(down(emnc.Bz))\*(real(down(emnc.Hx))\*emnc.unx+real(down(emnc.Hy))\*emnc.uny+real(down(emnc.Hz))\*emnc.unz) | Pa | Maxwell downward magnetic surface stress tensor, z component | Boundaries 105–108, 113, 115 |
| emnc.Hx | -Vmx | A/m | Magnetic field, x component | Domain 18 |
| emnc.Hy | -Vmy | A/m | Magnetic field, y component | Domain 18 |
| emnc.Hz | -Vmz | A/m | Magnetic field, z component | Domain 18 |
| emnc.tHx | -VmTx | A/m | Tangential magnetic field, x component | Boundaries 105–108, 113, 115 |
| emnc.tHy | -VmTy | A/m | Tangential magnetic field, y component | Boundaries 105–108, 113, 115 |
| emnc.tHz | -VmTz | A/m | Tangential magnetic field, z component | Boundaries 105–108, 113, 115 |
| emnc.normH | sqrt(realdot(emnc.Hx,emnc.Hx)+realdot(emnc.Hy,emnc.Hy)+realdot(emnc.Hz,emnc.Hz)) | A/m | Magnetic field norm | Domain 18 |
| emnc.murxx | model.input.mur11 | 1 | Relative permeability, xx component | Domain 18 |
| emnc.muryx | model.input.mur21 | 1 | Relative permeability, yx component | Domain 18 |
| emnc.murzx | model.input.mur31 | 1 | Relative permeability, zx component | Domain 18 |
| emnc.murxy | model.input.mur12 | 1 | Relative permeability, xy component | Domain 18 |
| emnc.muryy | model.input.mur22 | 1 | Relative permeability, yy component | Domain 18 |
| emnc.murzy | model.input.mur32 | 1 | Relative permeability, zy component | Domain 18 |
| emnc.murxz | model.input.mur13 | 1 | Relative permeability, xz component | Domain 18 |
| emnc.muryz | model.input.mur23 | 1 | Relative permeability, yz component | Domain 18 |
| emnc.murzz | model.input.mur33 | 1 | Relative permeability, zz component | Domain 18 |
| emnc.Bx | mu0\_const\*emnc.Ixx\*emnc.Hx+mu0\_const\*emnc.Ixy\*emnc.Hy+mu0\_const\*emnc.Ixz\*emnc.Hz+mu0\_const\*emnc.chimxx\*emnc.Hx+mu0\_const\*emnc.chimxy\*emnc.Hy+mu0\_const\*emnc.chimxz\*emnc.Hz+emnc.Brx | T | Magnetic flux density, x component | Domain 18 |
| emnc.By | mu0\_const\*emnc.Iyx\*emnc.Hx+mu0\_const\*emnc.Iyy\*emnc.Hy+mu0\_const\*emnc.Iyz\*emnc.Hz+mu0\_const\*emnc.chimyx\*emnc.Hx+mu0\_const\*emnc.chimyy\*emnc.Hy+mu0\_const\*emnc.chimyz\*emnc.Hz+emnc.Bry | T | Magnetic flux density, y component | Domain 18 |
| emnc.Bz | mu0\_const\*emnc.Izx\*emnc.Hx+mu0\_const\*emnc.Izy\*emnc.Hy+mu0\_const\*emnc.Izz\*emnc.Hz+mu0\_const\*emnc.chimzx\*emnc.Hx+mu0\_const\*emnc.chimzy\*emnc.Hy+mu0\_const\*emnc.chimzz\*emnc.Hz+emnc.Brz | T | Magnetic flux density, z component | Domain 18 |
| emnc.normB | sqrt(realdot(emnc.Bx,emnc.Bx)+realdot(emnc.By,emnc.By)+realdot(emnc.Bz,emnc.Bz)) | T | Magnetic flux density norm | Domain 18 |
| emnc.Mx | emnc.Bx/mu0\_const-emnc.Ixx\*emnc.Hx-emnc.Ixy\*emnc.Hy-emnc.Ixz\*emnc.Hz | A/m | Magnetization, x component | Domain 18 |
| emnc.My | emnc.By/mu0\_const-emnc.Iyx\*emnc.Hx-emnc.Iyy\*emnc.Hy-emnc.Iyz\*emnc.Hz | A/m | Magnetization, y component | Domain 18 |
| emnc.Mz | emnc.Bz/mu0\_const-emnc.Izx\*emnc.Hx-emnc.Izy\*emnc.Hy-emnc.Izz\*emnc.Hz | A/m | Magnetization, z component | Domain 18 |
| emnc.normM | sqrt(realdot(emnc.Mx,emnc.Mx)+realdot(emnc.My,emnc.My)+realdot(emnc.Mz,emnc.Mz)) | A/m | Magnetization norm | Domain 18 |
| emnc.Ixx | 1 | 1 | Spatial identity matrix, xx component | Domain 18 |
| emnc.Iyx | 0 | 1 | Spatial identity matrix, yx component | Domain 18 |
| emnc.Izx | 0 | 1 | Spatial identity matrix, zx component | Domain 18 |
| emnc.Ixy | 0 | 1 | Spatial identity matrix, xy component | Domain 18 |
| emnc.Iyy | 1 | 1 | Spatial identity matrix, yy component | Domain 18 |
| emnc.Izy | 0 | 1 | Spatial identity matrix, zy component | Domain 18 |
| emnc.Ixz | 0 | 1 | Spatial identity matrix, xz component | Domain 18 |
| emnc.Iyz | 0 | 1 | Spatial identity matrix, yz component | Domain 18 |
| emnc.Izz | 1 | 1 | Spatial identity matrix, zz component | Domain 18 |
| emnc.Brx | BpRem\*cos(20\*argBp+tick) | T | Remanent flux density, x component | Domain 18 |
| emnc.Bry | BpRem\*sin(20\*argBp+tick) | T | Remanent flux density, y component | Domain 18 |
| emnc.Brz | 0 | T | Remanent flux density, z component | Domain 18 |
| emnc.normBr | sqrt(realdot(emnc.Brx,emnc.Brx)+realdot(emnc.Bry,emnc.Bry)+realdot(emnc.Brz,emnc.Brz)) | T | Remanent flux density norm | Domain 18 |
| emnc.chimxx | -1+emnc.murxx | 1 | Magnetic susceptibility, xx component | Domain 18 |
| emnc.chimyx | emnc.muryx | 1 | Magnetic susceptibility, yx component | Domain 18 |
| emnc.chimzx | emnc.murzx | 1 | Magnetic susceptibility, zx component | Domain 18 |
| emnc.chimxy | emnc.murxy | 1 | Magnetic susceptibility, xy component | Domain 18 |
| emnc.chimyy | -1+emnc.muryy | 1 | Magnetic susceptibility, yy component | Domain 18 |
| emnc.chimzy | emnc.murzy | 1 | Magnetic susceptibility, zy component | Domain 18 |
| emnc.chimxz | emnc.murxz | 1 | Magnetic susceptibility, xz component | Domain 18 |
| emnc.chimyz | emnc.muryz | 1 | Magnetic susceptibility, yz component | Domain 18 |
| emnc.chimzz | -1+emnc.murzz | 1 | Magnetic susceptibility, zz component | Domain 18 |
| emnc.unTx | 0 | Pa | Maxwell upward surface stress tensor, x component | Boundaries 105–108, 113, 115 |
| emnc.unTy | 0 | Pa | Maxwell upward surface stress tensor, y component | Boundaries 105–108, 113, 115 |
| emnc.unTz | 0 | Pa | Maxwell upward surface stress tensor, z component | Boundaries 105–108, 113, 115 |
| emnc.dnTx | emnc.dnTmx | Pa | Maxwell downward surface stress tensor, x component | Boundaries 105–108, 113, 115 |
| emnc.dnTy | emnc.dnTmy | Pa | Maxwell downward surface stress tensor, y component | Boundaries 105–108, 113, 115 |
| emnc.dnTz | emnc.dnTmz | Pa | Maxwell downward surface stress tensor, z component | Boundaries 105–108, 113, 115 |
| emnc.unx | unx |  | Normal vector up direction, x component | Boundaries 105–108, 113, 115 |
| emnc.uny | uny |  | Normal vector up direction, y component | Boundaries 105–108, 113, 115 |
| emnc.unz | unz |  | Normal vector up direction, z component | Boundaries 105–108, 113, 115 |
| emnc.dnx | dnx |  | Normal vector down direction, x component | Boundaries 105–108, 113, 115 |
| emnc.dny | dny |  | Normal vector down direction, y component | Boundaries 105–108, 113, 115 |
| emnc.dnz | dnz |  | Normal vector down direction, z component | Boundaries 105–108, 113, 115 |
| emnc.W | emnc.Wm | J/m^3 | Energy density | Domain 18 |
| emnc.dWm | emnc.Wm | J/m^3 | Integrand for total magnetic energy | Domain 18 |
| emnc.Wm | 0.5\*mu0\_const\*((emnc.murxx\*emnc.Hx+emnc.murxy\*emnc.Hy+emnc.murxz\*emnc.Hz)\*emnc.Hx+(emnc.muryx\*emnc.Hx+emnc.muryy\*emnc.Hy+emnc.muryz\*emnc.Hz)\*emnc.Hy+(emnc.murzx\*emnc.Hx+emnc.murzy\*emnc.Hy+emnc.murzz\*emnc.Hz)\*emnc.Hz) | J/m^3 | Magnetic energy density | Domain 18 |

##### Shape Functions

| Name | Shape function | Unit | Description | Shape frame | Selection |
| --- | --- | --- | --- | --- | --- |
| Vm | Lagrange (Quadratic) | A | Magnetic scalar potential | Material | Domain 18 |

##### Weak Expressions

| Weak expression | Integration frame | Selection |
| --- | --- | --- |
| emnc.d\*(-emnc.Bx\*test(Vmx)-emnc.By\*test(Vmy)-emnc.Bz\*test(Vmz)) | Material | Domain 18 |

#### 2.4.25. Magnetic Flux Conservation 23

Magnetic Flux Conservation 23

Selection

| Geometric entity level | Domain |
| Selection | Domain 20 |

Equations

Settings

| Description | Value |
| Constitutive relation | Remanent flux density |
| Remanent flux density, x component | BpRem\*cos(argBp\*(-1 + 22) + tick) |
| Remanent flux density, y component | BpRem\*sin(argBp\*(-1 + 22) + tick) |
| Remanent flux density, z component | 0 |
| Relative permeability | From material |
| Relative permeability | {{1, 0, 0}, {0, 1, 0}, {0, 0, 1}} |

Properties from material

| Property | Material | Property group |
| Relative permeability | Soft Iron (without losses) | Basic |

##### Variables

| Name | Expression | Unit | Description | Selection |
| --- | --- | --- | --- | --- |
| emnc.dnTmx | -0.5\*emnc.unx\*(real(down(emnc.Bx))\*real(down(emnc.Hx))+real(down(emnc.By))\*real(down(emnc.Hy))+real(down(emnc.Bz))\*real(down(emnc.Hz)))+real(down(emnc.Bx))\*(real(down(emnc.Hx))\*emnc.unx+real(down(emnc.Hy))\*emnc.uny+real(down(emnc.Hz))\*emnc.unz) | Pa | Maxwell downward magnetic surface stress tensor, x component | Boundaries 117–121, 127 |
| emnc.dnTmy | -0.5\*emnc.uny\*(real(down(emnc.Bx))\*real(down(emnc.Hx))+real(down(emnc.By))\*real(down(emnc.Hy))+real(down(emnc.Bz))\*real(down(emnc.Hz)))+real(down(emnc.By))\*(real(down(emnc.Hx))\*emnc.unx+real(down(emnc.Hy))\*emnc.uny+real(down(emnc.Hz))\*emnc.unz) | Pa | Maxwell downward magnetic surface stress tensor, y component | Boundaries 117–121, 127 |
| emnc.dnTmz | -0.5\*emnc.unz\*(real(down(emnc.Bx))\*real(down(emnc.Hx))+real(down(emnc.By))\*real(down(emnc.Hy))+real(down(emnc.Bz))\*real(down(emnc.Hz)))+real(down(emnc.Bz))\*(real(down(emnc.Hx))\*emnc.unx+real(down(emnc.Hy))\*emnc.uny+real(down(emnc.Hz))\*emnc.unz) | Pa | Maxwell downward magnetic surface stress tensor, z component | Boundaries 117–121, 127 |
| emnc.Hx | -Vmx | A/m | Magnetic field, x component | Domain 20 |
| emnc.Hy | -Vmy | A/m | Magnetic field, y component | Domain 20 |
| emnc.Hz | -Vmz | A/m | Magnetic field, z component | Domain 20 |
| emnc.tHx | -VmTx | A/m | Tangential magnetic field, x component | Boundaries 117–121, 127 |
| emnc.tHy | -VmTy | A/m | Tangential magnetic field, y component | Boundaries 117–121, 127 |
| emnc.tHz | -VmTz | A/m | Tangential magnetic field, z component | Boundaries 117–121, 127 |
| emnc.normH | sqrt(realdot(emnc.Hx,emnc.Hx)+realdot(emnc.Hy,emnc.Hy)+realdot(emnc.Hz,emnc.Hz)) | A/m | Magnetic field norm | Domain 20 |
| emnc.murxx | model.input.mur11 | 1 | Relative permeability, xx component | Domain 20 |
| emnc.muryx | model.input.mur21 | 1 | Relative permeability, yx component | Domain 20 |
| emnc.murzx | model.input.mur31 | 1 | Relative permeability, zx component | Domain 20 |
| emnc.murxy | model.input.mur12 | 1 | Relative permeability, xy component | Domain 20 |
| emnc.muryy | model.input.mur22 | 1 | Relative permeability, yy component | Domain 20 |
| emnc.murzy | model.input.mur32 | 1 | Relative permeability, zy component | Domain 20 |
| emnc.murxz | model.input.mur13 | 1 | Relative permeability, xz component | Domain 20 |
| emnc.muryz | model.input.mur23 | 1 | Relative permeability, yz component | Domain 20 |
| emnc.murzz | model.input.mur33 | 1 | Relative permeability, zz component | Domain 20 |
| emnc.Bx | mu0\_const\*emnc.Ixx\*emnc.Hx+mu0\_const\*emnc.Ixy\*emnc.Hy+mu0\_const\*emnc.Ixz\*emnc.Hz+mu0\_const\*emnc.chimxx\*emnc.Hx+mu0\_const\*emnc.chimxy\*emnc.Hy+mu0\_const\*emnc.chimxz\*emnc.Hz+emnc.Brx | T | Magnetic flux density, x component | Domain 20 |
| emnc.By | mu0\_const\*emnc.Iyx\*emnc.Hx+mu0\_const\*emnc.Iyy\*emnc.Hy+mu0\_const\*emnc.Iyz\*emnc.Hz+mu0\_const\*emnc.chimyx\*emnc.Hx+mu0\_const\*emnc.chimyy\*emnc.Hy+mu0\_const\*emnc.chimyz\*emnc.Hz+emnc.Bry | T | Magnetic flux density, y component | Domain 20 |
| emnc.Bz | mu0\_const\*emnc.Izx\*emnc.Hx+mu0\_const\*emnc.Izy\*emnc.Hy+mu0\_const\*emnc.Izz\*emnc.Hz+mu0\_const\*emnc.chimzx\*emnc.Hx+mu0\_const\*emnc.chimzy\*emnc.Hy+mu0\_const\*emnc.chimzz\*emnc.Hz+emnc.Brz | T | Magnetic flux density, z component | Domain 20 |
| emnc.normB | sqrt(realdot(emnc.Bx,emnc.Bx)+realdot(emnc.By,emnc.By)+realdot(emnc.Bz,emnc.Bz)) | T | Magnetic flux density norm | Domain 20 |
| emnc.Mx | emnc.Bx/mu0\_const-emnc.Ixx\*emnc.Hx-emnc.Ixy\*emnc.Hy-emnc.Ixz\*emnc.Hz | A/m | Magnetization, x component | Domain 20 |
| emnc.My | emnc.By/mu0\_const-emnc.Iyx\*emnc.Hx-emnc.Iyy\*emnc.Hy-emnc.Iyz\*emnc.Hz | A/m | Magnetization, y component | Domain 20 |
| emnc.Mz | emnc.Bz/mu0\_const-emnc.Izx\*emnc.Hx-emnc.Izy\*emnc.Hy-emnc.Izz\*emnc.Hz | A/m | Magnetization, z component | Domain 20 |
| emnc.normM | sqrt(realdot(emnc.Mx,emnc.Mx)+realdot(emnc.My,emnc.My)+realdot(emnc.Mz,emnc.Mz)) | A/m | Magnetization norm | Domain 20 |
| emnc.Ixx | 1 | 1 | Spatial identity matrix, xx component | Domain 20 |
| emnc.Iyx | 0 | 1 | Spatial identity matrix, yx component | Domain 20 |
| emnc.Izx | 0 | 1 | Spatial identity matrix, zx component | Domain 20 |
| emnc.Ixy | 0 | 1 | Spatial identity matrix, xy component | Domain 20 |
| emnc.Iyy | 1 | 1 | Spatial identity matrix, yy component | Domain 20 |
| emnc.Izy | 0 | 1 | Spatial identity matrix, zy component | Domain 20 |
| emnc.Ixz | 0 | 1 | Spatial identity matrix, xz component | Domain 20 |
| emnc.Iyz | 0 | 1 | Spatial identity matrix, yz component | Domain 20 |
| emnc.Izz | 1 | 1 | Spatial identity matrix, zz component | Domain 20 |
| emnc.Brx | BpRem\*cos(21\*argBp+tick) | T | Remanent flux density, x component | Domain 20 |
| emnc.Bry | BpRem\*sin(21\*argBp+tick) | T | Remanent flux density, y component | Domain 20 |
| emnc.Brz | 0 | T | Remanent flux density, z component | Domain 20 |
| emnc.normBr | sqrt(realdot(emnc.Brx,emnc.Brx)+realdot(emnc.Bry,emnc.Bry)+realdot(emnc.Brz,emnc.Brz)) | T | Remanent flux density norm | Domain 20 |
| emnc.chimxx | -1+emnc.murxx | 1 | Magnetic susceptibility, xx component | Domain 20 |
| emnc.chimyx | emnc.muryx | 1 | Magnetic susceptibility, yx component | Domain 20 |
| emnc.chimzx | emnc.murzx | 1 | Magnetic susceptibility, zx component | Domain 20 |
| emnc.chimxy | emnc.murxy | 1 | Magnetic susceptibility, xy component | Domain 20 |
| emnc.chimyy | -1+emnc.muryy | 1 | Magnetic susceptibility, yy component | Domain 20 |
| emnc.chimzy | emnc.murzy | 1 | Magnetic susceptibility, zy component | Domain 20 |
| emnc.chimxz | emnc.murxz | 1 | Magnetic susceptibility, xz component | Domain 20 |
| emnc.chimyz | emnc.muryz | 1 | Magnetic susceptibility, yz component | Domain 20 |
| emnc.chimzz | -1+emnc.murzz | 1 | Magnetic susceptibility, zz component | Domain 20 |
| emnc.unTx | 0 | Pa | Maxwell upward surface stress tensor, x component | Boundaries 117–121, 127 |
| emnc.unTy | 0 | Pa | Maxwell upward surface stress tensor, y component | Boundaries 117–121, 127 |
| emnc.unTz | 0 | Pa | Maxwell upward surface stress tensor, z component | Boundaries 117–121, 127 |
| emnc.dnTx | emnc.dnTmx | Pa | Maxwell downward surface stress tensor, x component | Boundaries 117–121, 127 |
| emnc.dnTy | emnc.dnTmy | Pa | Maxwell downward surface stress tensor, y component | Boundaries 117–121, 127 |
| emnc.dnTz | emnc.dnTmz | Pa | Maxwell downward surface stress tensor, z component | Boundaries 117–121, 127 |
| emnc.unx | unx |  | Normal vector up direction, x component | Boundaries 117–121, 127 |
| emnc.uny | uny |  | Normal vector up direction, y component | Boundaries 117–121, 127 |
| emnc.unz | unz |  | Normal vector up direction, z component | Boundaries 117–121, 127 |
| emnc.dnx | dnx |  | Normal vector down direction, x component | Boundaries 117–121, 127 |
| emnc.dny | dny |  | Normal vector down direction, y component | Boundaries 117–121, 127 |
| emnc.dnz | dnz |  | Normal vector down direction, z component | Boundaries 117–121, 127 |
| emnc.W | emnc.Wm | J/m^3 | Energy density | Domain 20 |
| emnc.dWm | emnc.Wm | J/m^3 | Integrand for total magnetic energy | Domain 20 |
| emnc.Wm | 0.5\*mu0\_const\*((emnc.murxx\*emnc.Hx+emnc.murxy\*emnc.Hy+emnc.murxz\*emnc.Hz)\*emnc.Hx+(emnc.muryx\*emnc.Hx+emnc.muryy\*emnc.Hy+emnc.muryz\*emnc.Hz)\*emnc.Hy+(emnc.murzx\*emnc.Hx+emnc.murzy\*emnc.Hy+emnc.murzz\*emnc.Hz)\*emnc.Hz) | J/m^3 | Magnetic energy density | Domain 20 |

##### Shape Functions

| Name | Shape function | Unit | Description | Shape frame | Selection |
| --- | --- | --- | --- | --- | --- |
| Vm | Lagrange (Quadratic) | A | Magnetic scalar potential | Material | Domain 20 |

##### Weak Expressions

| Weak expression | Integration frame | Selection |
| --- | --- | --- |
| emnc.d\*(-emnc.Bx\*test(Vmx)-emnc.By\*test(Vmy)-emnc.Bz\*test(Vmz)) | Material | Domain 20 |

#### 2.4.26. Magnetic Flux Conservation 24

Magnetic Flux Conservation 24

Selection

| Geometric entity level | Domain |
| Selection | Domain 22 |

Equations

Settings

| Description | Value |
| Constitutive relation | Remanent flux density |
| Remanent flux density, x component | BpRem\*cos(argBp\*(-1 + 23) + tick) |
| Remanent flux density, y component | BpRem\*sin(argBp\*(-1 + 23) + tick) |
| Remanent flux density, z component | 0 |
| Relative permeability | From material |
| Relative permeability | {{1, 0, 0}, {0, 1, 0}, {0, 0, 1}} |

Properties from material

| Property | Material | Property group |
| Relative permeability | Soft Iron (without losses) | Basic |

##### Variables

| Name | Expression | Unit | Description | Selection |
| --- | --- | --- | --- | --- |
| emnc.dnTmx | -0.5\*emnc.unx\*(real(down(emnc.Bx))\*real(down(emnc.Hx))+real(down(emnc.By))\*real(down(emnc.Hy))+real(down(emnc.Bz))\*real(down(emnc.Hz)))+real(down(emnc.Bx))\*(real(down(emnc.Hx))\*emnc.unx+real(down(emnc.Hy))\*emnc.uny+real(down(emnc.Hz))\*emnc.unz) | Pa | Maxwell downward magnetic surface stress tensor, x component | Boundaries 129–132, 137, 151 |
| emnc.dnTmy | -0.5\*emnc.uny\*(real(down(emnc.Bx))\*real(down(emnc.Hx))+real(down(emnc.By))\*real(down(emnc.Hy))+real(down(emnc.Bz))\*real(down(emnc.Hz)))+real(down(emnc.By))\*(real(down(emnc.Hx))\*emnc.unx+real(down(emnc.Hy))\*emnc.uny+real(down(emnc.Hz))\*emnc.unz) | Pa | Maxwell downward magnetic surface stress tensor, y component | Boundaries 129–132, 137, 151 |
| emnc.dnTmz | -0.5\*emnc.unz\*(real(down(emnc.Bx))\*real(down(emnc.Hx))+real(down(emnc.By))\*real(down(emnc.Hy))+real(down(emnc.Bz))\*real(down(emnc.Hz)))+real(down(emnc.Bz))\*(real(down(emnc.Hx))\*emnc.unx+real(down(emnc.Hy))\*emnc.uny+real(down(emnc.Hz))\*emnc.unz) | Pa | Maxwell downward magnetic surface stress tensor, z component | Boundaries 129–132, 137, 151 |
| emnc.Hx | -Vmx | A/m | Magnetic field, x component | Domain 22 |
| emnc.Hy | -Vmy | A/m | Magnetic field, y component | Domain 22 |
| emnc.Hz | -Vmz | A/m | Magnetic field, z component | Domain 22 |
| emnc.tHx | -VmTx | A/m | Tangential magnetic field, x component | Boundaries 129–132, 137, 151 |
| emnc.tHy | -VmTy | A/m | Tangential magnetic field, y component | Boundaries 129–132, 137, 151 |
| emnc.tHz | -VmTz | A/m | Tangential magnetic field, z component | Boundaries 129–132, 137, 151 |
| emnc.normH | sqrt(realdot(emnc.Hx,emnc.Hx)+realdot(emnc.Hy,emnc.Hy)+realdot(emnc.Hz,emnc.Hz)) | A/m | Magnetic field norm | Domain 22 |
| emnc.murxx | model.input.mur11 | 1 | Relative permeability, xx component | Domain 22 |
| emnc.muryx | model.input.mur21 | 1 | Relative permeability, yx component | Domain 22 |
| emnc.murzx | model.input.mur31 | 1 | Relative permeability, zx component | Domain 22 |
| emnc.murxy | model.input.mur12 | 1 | Relative permeability, xy component | Domain 22 |
| emnc.muryy | model.input.mur22 | 1 | Relative permeability, yy component | Domain 22 |
| emnc.murzy | model.input.mur32 | 1 | Relative permeability, zy component | Domain 22 |
| emnc.murxz | model.input.mur13 | 1 | Relative permeability, xz component | Domain 22 |
| emnc.muryz | model.input.mur23 | 1 | Relative permeability, yz component | Domain 22 |
| emnc.murzz | model.input.mur33 | 1 | Relative permeability, zz component | Domain 22 |
| emnc.Bx | mu0\_const\*emnc.Ixx\*emnc.Hx+mu0\_const\*emnc.Ixy\*emnc.Hy+mu0\_const\*emnc.Ixz\*emnc.Hz+mu0\_const\*emnc.chimxx\*emnc.Hx+mu0\_const\*emnc.chimxy\*emnc.Hy+mu0\_const\*emnc.chimxz\*emnc.Hz+emnc.Brx | T | Magnetic flux density, x component | Domain 22 |
| emnc.By | mu0\_const\*emnc.Iyx\*emnc.Hx+mu0\_const\*emnc.Iyy\*emnc.Hy+mu0\_const\*emnc.Iyz\*emnc.Hz+mu0\_const\*emnc.chimyx\*emnc.Hx+mu0\_const\*emnc.chimyy\*emnc.Hy+mu0\_const\*emnc.chimyz\*emnc.Hz+emnc.Bry | T | Magnetic flux density, y component | Domain 22 |
| emnc.Bz | mu0\_const\*emnc.Izx\*emnc.Hx+mu0\_const\*emnc.Izy\*emnc.Hy+mu0\_const\*emnc.Izz\*emnc.Hz+mu0\_const\*emnc.chimzx\*emnc.Hx+mu0\_const\*emnc.chimzy\*emnc.Hy+mu0\_const\*emnc.chimzz\*emnc.Hz+emnc.Brz | T | Magnetic flux density, z component | Domain 22 |
| emnc.normB | sqrt(realdot(emnc.Bx,emnc.Bx)+realdot(emnc.By,emnc.By)+realdot(emnc.Bz,emnc.Bz)) | T | Magnetic flux density norm | Domain 22 |
| emnc.Mx | emnc.Bx/mu0\_const-emnc.Ixx\*emnc.Hx-emnc.Ixy\*emnc.Hy-emnc.Ixz\*emnc.Hz | A/m | Magnetization, x component | Domain 22 |
| emnc.My | emnc.By/mu0\_const-emnc.Iyx\*emnc.Hx-emnc.Iyy\*emnc.Hy-emnc.Iyz\*emnc.Hz | A/m | Magnetization, y component | Domain 22 |
| emnc.Mz | emnc.Bz/mu0\_const-emnc.Izx\*emnc.Hx-emnc.Izy\*emnc.Hy-emnc.Izz\*emnc.Hz | A/m | Magnetization, z component | Domain 22 |
| emnc.normM | sqrt(realdot(emnc.Mx,emnc.Mx)+realdot(emnc.My,emnc.My)+realdot(emnc.Mz,emnc.Mz)) | A/m | Magnetization norm | Domain 22 |
| emnc.Ixx | 1 | 1 | Spatial identity matrix, xx component | Domain 22 |
| emnc.Iyx | 0 | 1 | Spatial identity matrix, yx component | Domain 22 |
| emnc.Izx | 0 | 1 | Spatial identity matrix, zx component | Domain 22 |
| emnc.Ixy | 0 | 1 | Spatial identity matrix, xy component | Domain 22 |
| emnc.Iyy | 1 | 1 | Spatial identity matrix, yy component | Domain 22 |
| emnc.Izy | 0 | 1 | Spatial identity matrix, zy component | Domain 22 |
| emnc.Ixz | 0 | 1 | Spatial identity matrix, xz component | Domain 22 |
| emnc.Iyz | 0 | 1 | Spatial identity matrix, yz component | Domain 22 |
| emnc.Izz | 1 | 1 | Spatial identity matrix, zz component | Domain 22 |
| emnc.Brx | BpRem\*cos(22\*argBp+tick) | T | Remanent flux density, x component | Domain 22 |
| emnc.Bry | BpRem\*sin(22\*argBp+tick) | T | Remanent flux density, y component | Domain 22 |
| emnc.Brz | 0 | T | Remanent flux density, z component | Domain 22 |
| emnc.normBr | sqrt(realdot(emnc.Brx,emnc.Brx)+realdot(emnc.Bry,emnc.Bry)+realdot(emnc.Brz,emnc.Brz)) | T | Remanent flux density norm | Domain 22 |
| emnc.chimxx | -1+emnc.murxx | 1 | Magnetic susceptibility, xx component | Domain 22 |
| emnc.chimyx | emnc.muryx | 1 | Magnetic susceptibility, yx component | Domain 22 |
| emnc.chimzx | emnc.murzx | 1 | Magnetic susceptibility, zx component | Domain 22 |
| emnc.chimxy | emnc.murxy | 1 | Magnetic susceptibility, xy component | Domain 22 |
| emnc.chimyy | -1+emnc.muryy | 1 | Magnetic susceptibility, yy component | Domain 22 |
| emnc.chimzy | emnc.murzy | 1 | Magnetic susceptibility, zy component | Domain 22 |
| emnc.chimxz | emnc.murxz | 1 | Magnetic susceptibility, xz component | Domain 22 |
| emnc.chimyz | emnc.muryz | 1 | Magnetic susceptibility, yz component | Domain 22 |
| emnc.chimzz | -1+emnc.murzz | 1 | Magnetic susceptibility, zz component | Domain 22 |
| emnc.unTx | 0 | Pa | Maxwell upward surface stress tensor, x component | Boundaries 129–132, 137, 151 |
| emnc.unTy | 0 | Pa | Maxwell upward surface stress tensor, y component | Boundaries 129–132, 137, 151 |
| emnc.unTz | 0 | Pa | Maxwell upward surface stress tensor, z component | Boundaries 129–132, 137, 151 |
| emnc.dnTx | emnc.dnTmx | Pa | Maxwell downward surface stress tensor, x component | Boundaries 129–132, 137, 151 |
| emnc.dnTy | emnc.dnTmy | Pa | Maxwell downward surface stress tensor, y component | Boundaries 129–132, 137, 151 |
| emnc.dnTz | emnc.dnTmz | Pa | Maxwell downward surface stress tensor, z component | Boundaries 129–132, 137, 151 |
| emnc.unx | unx |  | Normal vector up direction, x component | Boundaries 129–132, 137, 151 |
| emnc.uny | uny |  | Normal vector up direction, y component | Boundaries 129–132, 137, 151 |
| emnc.unz | unz |  | Normal vector up direction, z component | Boundaries 129–132, 137, 151 |
| emnc.dnx | dnx |  | Normal vector down direction, x component | Boundaries 129–132, 137, 151 |
| emnc.dny | dny |  | Normal vector down direction, y component | Boundaries 129–132, 137, 151 |
| emnc.dnz | dnz |  | Normal vector down direction, z component | Boundaries 129–132, 137, 151 |
| emnc.W | emnc.Wm | J/m^3 | Energy density | Domain 22 |
| emnc.dWm | emnc.Wm | J/m^3 | Integrand for total magnetic energy | Domain 22 |
| emnc.Wm | 0.5\*mu0\_const\*((emnc.murxx\*emnc.Hx+emnc.murxy\*emnc.Hy+emnc.murxz\*emnc.Hz)\*emnc.Hx+(emnc.muryx\*emnc.Hx+emnc.muryy\*emnc.Hy+emnc.muryz\*emnc.Hz)\*emnc.Hy+(emnc.murzx\*emnc.Hx+emnc.murzy\*emnc.Hy+emnc.murzz\*emnc.Hz)\*emnc.Hz) | J/m^3 | Magnetic energy density | Domain 22 |

##### Shape Functions

| Name | Shape function | Unit | Description | Shape frame | Selection |
| --- | --- | --- | --- | --- | --- |
| Vm | Lagrange (Quadratic) | A | Magnetic scalar potential | Material | Domain 22 |

##### Weak Expressions

| Weak expression | Integration frame | Selection |
| --- | --- | --- |
| emnc.d\*(-emnc.Bx\*test(Vmx)-emnc.By\*test(Vmy)-emnc.Bz\*test(Vmz)) | Material | Domain 22 |

#### 2.4.27. Magnetic Flux Conservation 25

Magnetic Flux Conservation 25

Selection

| Geometric entity level | Domain |
| Selection | Domain 24 |

Equations

Settings

| Description | Value |
| Constitutive relation | Remanent flux density |
| Remanent flux density, x component | BpRem\*cos(argBp\*(-1 + 24) + tick) |
| Remanent flux density, y component | BpRem\*sin(argBp\*(-1 + 24) + tick) |
| Remanent flux density, z component | 0 |
| Relative permeability | From material |
| Relative permeability | {{1, 0, 0}, {0, 1, 0}, {0, 0, 1}} |

Properties from material

| Property | Material | Property group |
| Relative permeability | Soft Iron (without losses) | Basic |

##### Variables

| Name | Expression | Unit | Description | Selection |
| --- | --- | --- | --- | --- |
| emnc.dnTmx | -0.5\*emnc.unx\*(real(down(emnc.Bx))\*real(down(emnc.Hx))+real(down(emnc.By))\*real(down(emnc.Hy))+real(down(emnc.Bz))\*real(down(emnc.Hz)))+real(down(emnc.Bx))\*(real(down(emnc.Hx))\*emnc.unx+real(down(emnc.Hy))\*emnc.uny+real(down(emnc.Hz))\*emnc.unz) | Pa | Maxwell downward magnetic surface stress tensor, x component | Boundaries 139–142, 153, 155 |
| emnc.dnTmy | -0.5\*emnc.uny\*(real(down(emnc.Bx))\*real(down(emnc.Hx))+real(down(emnc.By))\*real(down(emnc.Hy))+real(down(emnc.Bz))\*real(down(emnc.Hz)))+real(down(emnc.By))\*(real(down(emnc.Hx))\*emnc.unx+real(down(emnc.Hy))\*emnc.uny+real(down(emnc.Hz))\*emnc.unz) | Pa | Maxwell downward magnetic surface stress tensor, y component | Boundaries 139–142, 153, 155 |
| emnc.dnTmz | -0.5\*emnc.unz\*(real(down(emnc.Bx))\*real(down(emnc.Hx))+real(down(emnc.By))\*real(down(emnc.Hy))+real(down(emnc.Bz))\*real(down(emnc.Hz)))+real(down(emnc.Bz))\*(real(down(emnc.Hx))\*emnc.unx+real(down(emnc.Hy))\*emnc.uny+real(down(emnc.Hz))\*emnc.unz) | Pa | Maxwell downward magnetic surface stress tensor, z component | Boundaries 139–142, 153, 155 |
| emnc.Hx | -Vmx | A/m | Magnetic field, x component | Domain 24 |
| emnc.Hy | -Vmy | A/m | Magnetic field, y component | Domain 24 |
| emnc.Hz | -Vmz | A/m | Magnetic field, z component | Domain 24 |
| emnc.tHx | -VmTx | A/m | Tangential magnetic field, x component | Boundaries 139–142, 153, 155 |
| emnc.tHy | -VmTy | A/m | Tangential magnetic field, y component | Boundaries 139–142, 153, 155 |
| emnc.tHz | -VmTz | A/m | Tangential magnetic field, z component | Boundaries 139–142, 153, 155 |
| emnc.normH | sqrt(realdot(emnc.Hx,emnc.Hx)+realdot(emnc.Hy,emnc.Hy)+realdot(emnc.Hz,emnc.Hz)) | A/m | Magnetic field norm | Domain 24 |
| emnc.murxx | model.input.mur11 | 1 | Relative permeability, xx component | Domain 24 |
| emnc.muryx | model.input.mur21 | 1 | Relative permeability, yx component | Domain 24 |
| emnc.murzx | model.input.mur31 | 1 | Relative permeability, zx component | Domain 24 |
| emnc.murxy | model.input.mur12 | 1 | Relative permeability, xy component | Domain 24 |
| emnc.muryy | model.input.mur22 | 1 | Relative permeability, yy component | Domain 24 |
| emnc.murzy | model.input.mur32 | 1 | Relative permeability, zy component | Domain 24 |
| emnc.murxz | model.input.mur13 | 1 | Relative permeability, xz component | Domain 24 |
| emnc.muryz | model.input.mur23 | 1 | Relative permeability, yz component | Domain 24 |
| emnc.murzz | model.input.mur33 | 1 | Relative permeability, zz component | Domain 24 |
| emnc.Bx | mu0\_const\*emnc.Ixx\*emnc.Hx+mu0\_const\*emnc.Ixy\*emnc.Hy+mu0\_const\*emnc.Ixz\*emnc.Hz+mu0\_const\*emnc.chimxx\*emnc.Hx+mu0\_const\*emnc.chimxy\*emnc.Hy+mu0\_const\*emnc.chimxz\*emnc.Hz+emnc.Brx | T | Magnetic flux density, x component | Domain 24 |
| emnc.By | mu0\_const\*emnc.Iyx\*emnc.Hx+mu0\_const\*emnc.Iyy\*emnc.Hy+mu0\_const\*emnc.Iyz\*emnc.Hz+mu0\_const\*emnc.chimyx\*emnc.Hx+mu0\_const\*emnc.chimyy\*emnc.Hy+mu0\_const\*emnc.chimyz\*emnc.Hz+emnc.Bry | T | Magnetic flux density, y component | Domain 24 |
| emnc.Bz | mu0\_const\*emnc.Izx\*emnc.Hx+mu0\_const\*emnc.Izy\*emnc.Hy+mu0\_const\*emnc.Izz\*emnc.Hz+mu0\_const\*emnc.chimzx\*emnc.Hx+mu0\_const\*emnc.chimzy\*emnc.Hy+mu0\_const\*emnc.chimzz\*emnc.Hz+emnc.Brz | T | Magnetic flux density, z component | Domain 24 |
| emnc.normB | sqrt(realdot(emnc.Bx,emnc.Bx)+realdot(emnc.By,emnc.By)+realdot(emnc.Bz,emnc.Bz)) | T | Magnetic flux density norm | Domain 24 |
| emnc.Mx | emnc.Bx/mu0\_const-emnc.Ixx\*emnc.Hx-emnc.Ixy\*emnc.Hy-emnc.Ixz\*emnc.Hz | A/m | Magnetization, x component | Domain 24 |
| emnc.My | emnc.By/mu0\_const-emnc.Iyx\*emnc.Hx-emnc.Iyy\*emnc.Hy-emnc.Iyz\*emnc.Hz | A/m | Magnetization, y component | Domain 24 |
| emnc.Mz | emnc.Bz/mu0\_const-emnc.Izx\*emnc.Hx-emnc.Izy\*emnc.Hy-emnc.Izz\*emnc.Hz | A/m | Magnetization, z component | Domain 24 |
| emnc.normM | sqrt(realdot(emnc.Mx,emnc.Mx)+realdot(emnc.My,emnc.My)+realdot(emnc.Mz,emnc.Mz)) | A/m | Magnetization norm | Domain 24 |
| emnc.Ixx | 1 | 1 | Spatial identity matrix, xx component | Domain 24 |
| emnc.Iyx | 0 | 1 | Spatial identity matrix, yx component | Domain 24 |
| emnc.Izx | 0 | 1 | Spatial identity matrix, zx component | Domain 24 |
| emnc.Ixy | 0 | 1 | Spatial identity matrix, xy component | Domain 24 |
| emnc.Iyy | 1 | 1 | Spatial identity matrix, yy component | Domain 24 |
| emnc.Izy | 0 | 1 | Spatial identity matrix, zy component | Domain 24 |
| emnc.Ixz | 0 | 1 | Spatial identity matrix, xz component | Domain 24 |
| emnc.Iyz | 0 | 1 | Spatial identity matrix, yz component | Domain 24 |
| emnc.Izz | 1 | 1 | Spatial identity matrix, zz component | Domain 24 |
| emnc.Brx | BpRem\*cos(23\*argBp+tick) | T | Remanent flux density, x component | Domain 24 |
| emnc.Bry | BpRem\*sin(23\*argBp+tick) | T | Remanent flux density, y component | Domain 24 |
| emnc.Brz | 0 | T | Remanent flux density, z component | Domain 24 |
| emnc.normBr | sqrt(realdot(emnc.Brx,emnc.Brx)+realdot(emnc.Bry,emnc.Bry)+realdot(emnc.Brz,emnc.Brz)) | T | Remanent flux density norm | Domain 24 |
| emnc.chimxx | -1+emnc.murxx | 1 | Magnetic susceptibility, xx component | Domain 24 |
| emnc.chimyx | emnc.muryx | 1 | Magnetic susceptibility, yx component | Domain 24 |
| emnc.chimzx | emnc.murzx | 1 | Magnetic susceptibility, zx component | Domain 24 |
| emnc.chimxy | emnc.murxy | 1 | Magnetic susceptibility, xy component | Domain 24 |
| emnc.chimyy | -1+emnc.muryy | 1 | Magnetic susceptibility, yy component | Domain 24 |
| emnc.chimzy | emnc.murzy | 1 | Magnetic susceptibility, zy component | Domain 24 |
| emnc.chimxz | emnc.murxz | 1 | Magnetic susceptibility, xz component | Domain 24 |
| emnc.chimyz | emnc.muryz | 1 | Magnetic susceptibility, yz component | Domain 24 |
| emnc.chimzz | -1+emnc.murzz | 1 | Magnetic susceptibility, zz component | Domain 24 |
| emnc.unTx | 0 | Pa | Maxwell upward surface stress tensor, x component | Boundaries 139–142, 153, 155 |
| emnc.unTy | 0 | Pa | Maxwell upward surface stress tensor, y component | Boundaries 139–142, 153, 155 |
| emnc.unTz | 0 | Pa | Maxwell upward surface stress tensor, z component | Boundaries 139–142, 153, 155 |
| emnc.dnTx | emnc.dnTmx | Pa | Maxwell downward surface stress tensor, x component | Boundaries 139–142, 153, 155 |
| emnc.dnTy | emnc.dnTmy | Pa | Maxwell downward surface stress tensor, y component | Boundaries 139–142, 153, 155 |
| emnc.dnTz | emnc.dnTmz | Pa | Maxwell downward surface stress tensor, z component | Boundaries 139–142, 153, 155 |
| emnc.unx | unx |  | Normal vector up direction, x component | Boundaries 139–142, 153, 155 |
| emnc.uny | uny |  | Normal vector up direction, y component | Boundaries 139–142, 153, 155 |
| emnc.unz | unz |  | Normal vector up direction, z component | Boundaries 139–142, 153, 155 |
| emnc.dnx | dnx |  | Normal vector down direction, x component | Boundaries 139–142, 153, 155 |
| emnc.dny | dny |  | Normal vector down direction, y component | Boundaries 139–142, 153, 155 |
| emnc.dnz | dnz |  | Normal vector down direction, z component | Boundaries 139–142, 153, 155 |
| emnc.W | emnc.Wm | J/m^3 | Energy density | Domain 24 |
| emnc.dWm | emnc.Wm | J/m^3 | Integrand for total magnetic energy | Domain 24 |
| emnc.Wm | 0.5\*mu0\_const\*((emnc.murxx\*emnc.Hx+emnc.murxy\*emnc.Hy+emnc.murxz\*emnc.Hz)\*emnc.Hx+(emnc.muryx\*emnc.Hx+emnc.muryy\*emnc.Hy+emnc.muryz\*emnc.Hz)\*emnc.Hy+(emnc.murzx\*emnc.Hx+emnc.murzy\*emnc.Hy+emnc.murzz\*emnc.Hz)\*emnc.Hz) | J/m^3 | Magnetic energy density | Domain 24 |

##### Shape Functions

| Name | Shape function | Unit | Description | Shape frame | Selection |
| --- | --- | --- | --- | --- | --- |
| Vm | Lagrange (Quadratic) | A | Magnetic scalar potential | Material | Domain 24 |

##### Weak Expressions

| Weak expression | Integration frame | Selection |
| --- | --- | --- |
| emnc.d\*(-emnc.Bx\*test(Vmx)-emnc.By\*test(Vmy)-emnc.Bz\*test(Vmz)) | Material | Domain 24 |

#### 2.4.28. Magnetic Shielding 1

Magnetic Shielding 1

Selection

| Geometric entity level | Boundary |
| Selection | Boundaries 1–4, 83, 92 |

Equations

Settings

| Description | Value |
| Surface thickness | 0.12 |
| Relative permeability | User defined |
| Relative permeability | {{5500, 0, 0}, {0, 5500, 0}, {0, 0, 5500}} |

##### Variables

| Name | Expression | Unit | Description | Selection |
| --- | --- | --- | --- | --- |
| emnc.murxx | 5500 | 1 | Relative permeability, xx component | Boundaries 1–4, 83, 92 |
| emnc.muryx | 0 | 1 | Relative permeability, yx component | Boundaries 1–4, 83, 92 |
| emnc.murzx | 0 | 1 | Relative permeability, zx component | Boundaries 1–4, 83, 92 |
| emnc.murxy | 0 | 1 | Relative permeability, xy component | Boundaries 1–4, 83, 92 |
| emnc.muryy | 5500 | 1 | Relative permeability, yy component | Boundaries 1–4, 83, 92 |
| emnc.murzy | 0 | 1 | Relative permeability, zy component | Boundaries 1–4, 83, 92 |
| emnc.murxz | 0 | 1 | Relative permeability, xz component | Boundaries 1–4, 83, 92 |
| emnc.muryz | 0 | 1 | Relative permeability, yz component | Boundaries 1–4, 83, 92 |
| emnc.murzz | 5500 | 1 | Relative permeability, zz component | Boundaries 1–4, 83, 92 |
| emnc.Ixx | 1 | 1 | Spatial identity matrix, xx component | Boundaries 1–4, 83, 92 |
| emnc.Iyx | 0 | 1 | Spatial identity matrix, yx component | Boundaries 1–4, 83, 92 |
| emnc.Izx | 0 | 1 | Spatial identity matrix, zx component | Boundaries 1–4, 83, 92 |
| emnc.Ixy | 0 | 1 | Spatial identity matrix, xy component | Boundaries 1–4, 83, 92 |
| emnc.Iyy | 1 | 1 | Spatial identity matrix, yy component | Boundaries 1–4, 83, 92 |
| emnc.Izy | 0 | 1 | Spatial identity matrix, zy component | Boundaries 1–4, 83, 92 |
| emnc.Ixz | 0 | 1 | Spatial identity matrix, xz component | Boundaries 1–4, 83, 92 |
| emnc.Iyz | 0 | 1 | Spatial identity matrix, yz component | Boundaries 1–4, 83, 92 |
| emnc.Izz | 1 | 1 | Spatial identity matrix, zz component | Boundaries 1–4, 83, 92 |
| emnc.chimxx | -1+emnc.murxx | 1 | Magnetic susceptibility, xx component | Boundaries 1–4, 83, 92 |
| emnc.chimyx | emnc.muryx | 1 | Magnetic susceptibility, yx component | Boundaries 1–4, 83, 92 |
| emnc.chimzx | emnc.murzx | 1 | Magnetic susceptibility, zx component | Boundaries 1–4, 83, 92 |
| emnc.chimxy | emnc.murxy | 1 | Magnetic susceptibility, xy component | Boundaries 1–4, 83, 92 |
| emnc.chimyy | -1+emnc.muryy | 1 | Magnetic susceptibility, yy component | Boundaries 1–4, 83, 92 |
| emnc.chimzy | emnc.murzy | 1 | Magnetic susceptibility, zy component | Boundaries 1–4, 83, 92 |
| emnc.chimxz | emnc.murxz | 1 | Magnetic susceptibility, xz component | Boundaries 1–4, 83, 92 |
| emnc.chimyz | emnc.muryz | 1 | Magnetic susceptibility, yz component | Boundaries 1–4, 83, 92 |
| emnc.chimzz | -1+emnc.murzz | 1 | Magnetic susceptibility, zz component | Boundaries 1–4, 83, 92 |
| emnc.ds | 0.12 | m | Surface thickness | Boundaries 1–4, 83, 92 |
| emnc.tBx | mu0\_const\*(emnc.Ixx\*emnc.tHx+emnc.Ixy\*emnc.tHy+emnc.Ixz\*emnc.tHz+emnc.chimxx\*emnc.tHx+emnc.chimxy\*emnc.tHy+emnc.chimxz\*emnc.tHz) | T | Tangential magnetic flux density, x component | Boundaries 1–4, 83, 92 |
| emnc.tBy | mu0\_const\*(emnc.Iyx\*emnc.tHx+emnc.Iyy\*emnc.tHy+emnc.Iyz\*emnc.tHz+emnc.chimyx\*emnc.tHx+emnc.chimyy\*emnc.tHy+emnc.chimyz\*emnc.tHz) | T | Tangential magnetic flux density, y component | Boundaries 1–4, 83, 92 |
| emnc.tBz | mu0\_const\*(emnc.Izx\*emnc.tHx+emnc.Izy\*emnc.tHy+emnc.Izz\*emnc.tHz+emnc.chimzx\*emnc.tHx+emnc.chimzy\*emnc.tHy+emnc.chimzz\*emnc.tHz) | T | Tangential magnetic flux density, z component | Boundaries 1–4, 83, 92 |
| emnc.normtB | sqrt(realdot(emnc.tBx,emnc.tBx)+realdot(emnc.tBy,emnc.tBy)+realdot(emnc.tBz,emnc.tBz)) | T | Tangential magnetic flux density norm | Boundaries 1–4, 83, 92 |

##### Weak Expressions

| Weak expression | Integration frame | Selection |
| --- | --- | --- |
| (-emnc.tBx\*test(VmTx)-emnc.tBy\*test(VmTy)-emnc.tBz\*test(VmTz))\*emnc.d\*emnc.ds | Material | Boundaries 1–4, 83, 92 |

### 2.5. Mesh 1

Mesh statistics

| Property | Value |
| Minimum element quality | 0.06942 |
| Average element quality | 0.739 |
| Tetrahedral elements | 3644457 |
| Triangular elements | 260242 |
| Edge elements | 22220 |
| Vertex elements | 206 |

Mesh 1

#### 2.5.1. Size (Size)

Settings

| Name | Value |
| Maximum element size | 0.0825 |
| Minimum element size | 0.006 |
| Curvature factor | 0.4 |
| Resolution of narrow regions | 0.7 |
| Maximum element growth rate | 1.4 |
| Predefined size | Finer |

#### 2.5.2. Free Tetrahedral 1 (Ftet1)

Selection

| Geometric entity level | Domain |
| Selection | Domain 13 |

Free Tetrahedral 1

##### Size 1 (Size1)

Selection

| Geometric entity level | Domain |
| Selection | Domain 13 |

Size 1

Settings

| Name | Value |
| Maximum element size | 0.03 |
| Minimum element size | 3.0E-4 |
| Curvature factor | 0.2 |
| Maximum element growth rate | 1.3 |
| Predefined size | Extremely fine |

#### 2.5.3. Free Tetrahedral 2 (Ftet2)

Selection

| Geometric entity level | Remaining |

##### Size 1 (Size1)

Selection

| Geometric entity level | Domain |
| Selection | Domains 2–12, 14–26 |

Size 1

Settings

| Name | Value |
| Maximum element size | 0.0525 |
| Minimum element size | 0.00225 |
| Curvature factor | 0.3 |
| Resolution of narrow regions | 0.85 |
| Maximum element growth rate | 1.35 |
| Predefined size | Extra fine |

#### 2.5.4. Free Tetrahedral 3 (Ftet3)

Selection

| Geometric entity level | Remaining |

##### Size 1 (Size1)

Selection

| Geometric entity level | Domain |
| Selection | Geometry geom1 |

Size 1

Settings

| Name | Value |
| Maximum element size | 0.0525 |
| Minimum element size | 0.00225 |
| Curvature factor | 0.3 |
| Resolution of narrow regions | 0.85 |
| Maximum element growth rate | 1.35 |
| Predefined size | Extra fine |

## 3. Component 2

|  |  |
| --- | --- |
| Date | Mar 1, 2016 9:51:04 AM |

Component settings

| Unit system | SI |
| Geometry shape order | automatic |

### 3.1. Definitions

#### 3.1.1. Coordinate Systems

##### Boundary System 2

|  |  |
| --- | --- |
| Coordinate system type | Boundary system |
| Tag | sys2 |

Settings

| First (t1) | Second (n) | Third (to) |
| t1 | n | to |

Settings

| Name | Value |
| Create first tangent direction from | Global Cartesian |

### 3.2. Geometry 2

Geometry 2

Units

| Length unit | m |
| Angular unit | deg |

Geometry statistics

| Property | Value |
| Space dimension | 2 |
| Number of domains | 1 |
| Number of boundaries | 6 |
| Number of vertices | 6 |

#### 3.2.1. Composite Object 2 (CO2)

Selections of resulting entities

| Name | Value |
| Filename | $FILENAME$\_geom2\_CO2.mphbin |

## 4. Study 1

### 4.1. Stationary

Study settings

| Property | Value |
| Include geometric nonlinearity | Off |

Physics and variables selection

| Physics interface | Discretization |
| Magnetic Fields, No Currents (emnc) | physics |

Mesh selection

| Geometry | Mesh |
| Geometry 1 (geom1) | mesh1 |
| Geometry 2 (geom2) | nomesh |

### 4.2. Solver Configurations

#### 4.2.1. Solution 1

##### Compile Equations: Stationary (St1)

Study and step

| Name | Value |
| Use study | Study 1 |
| Use study step | Stationary |

##### Dependent Variables 1 (V1)

General

| Name | Value |
| Defined by study step | Stationary |

Initial values of variables solved for

| Name | Value |
| Solution | COMSOL 3.5a Solution |

Values of variables not solved for

| Name | Value |
| Solution | COMSOL 3.5a Solution |

###### Magnetic Scalar Potential (Mod1.Vm) (Mod1\_Vm)

General

| Name | Value |
| Field components | mod1.Vm |

##### Stationary Solver 1 (S1)

General

| Name | Value |
| Defined by study step | Stationary |
| Relative tolerance | 1.0E-6 |

Log

Stationary Solver 1 in Solution 1 started at 1-Mar-2016 11:43:26.

Linear solver

Number of degrees of freedom solved for: 4869978.

Symmetric matrices found.

Scales for dependent variables:

Magnetic scalar potential (mod1.Vm): 1

Orthonormal null-space function used.

Iter      SolEst     Damping    Stepsize #Res #Jac #Sol LinIt   LinErr   LinRes

1        0.94   1.0000000        0.94    1    1    1    37 4.7e-007 3.6e-009

Stationary Solver 1 in Solution 1: Solution time: 156 s (2 minutes, 36 seconds)

Physical memory: 10.15 GB

Virtual memory: 10.35 GB

###### Fully Coupled 1 (Fc1)

General

| Name | Value |
| Linear solver | Iterative 1 |

Method and termination

| Name | Value |
| Initial damping factor | 1.0 |
| Minimum damping factor | 1.0e-4 |
| Restriction for step-size update | 10.0 |
| Termination criterion | Solution |

###### Iterative 1 (I1)

General

| Name | Value |
| Solver | Conjugate gradients |

###### Multigrid 1 (Mg1)

General

| Name | Value |
| Solver | Algebraic multigrid |

###### Presmoother (Pr)

###### SOR 1 (So1)

General

| Name | Value |
| Relaxation factor | 1.0 |

###### Postsmoother (Po)

###### SOR 1 (So1)

General

| Name | Value |
| Relaxation factor | 1.0 |

###### Coarse Solver (Cs)

###### Direct 1 (D1)

Error

| Name | Value |
| Check error estimate | No |

#### 4.2.2. COMSOL 3.5a Solution

## 5. Results

### 5.1. Data Sets

#### 5.1.1. Study 1/Solution 1

Solution

| Name | Value |
| Solution | Solution 1 |
| Component | Save Point Geometry 1 |

Data set: Study 1/Solution 1

#### 5.1.2. Cut Plane 1

Data

| Name | Value |
| Data set | Study 1/Solution 1 |

Plane data

| Name | Value |
| Plane type | Quick |
| Plane | xy - planes |
| z-coordinate | 0 |

Advanced

| Name | Value |
| Space variables | {cpl1x, cpl1y} |

Data set: Cut Plane 1

#### 5.1.3. Cut Line 3D 1

Data

| Name | Value |
| Data set | Study 1/Solution 1 |

Line data

| Name | Value |
| Line entry method | Two points |
| Points | {{-0.1, 0, 0}, {0.1, 0, 0}} |

Advanced

| Name | Value |
| Space variable | cln1x |

Data set: Cut Line 3D 1

### 5.2. Tables

#### 5.2.1. Pg3/Mmv1

Max/min volume

pg3/mmv1

| x | y | z | Magnetic flux density norm (T) |
| 0.0000 | 0.0000 | 0.025000 | 0.11812 |
| -0.025000 | 0.0000 | 0.0000 | 0.11815 |

### 5.3. Plot Groups

#### 5.3.1. 2D Plot Group 1

Surface: Magnetic flux density norm (T) Arrow Line: Remanent flux density

#### 5.3.2. 1D Plot Group 2

Line Graph: (emnc.normB) (T)

#### 5.3.3. 3D Plot Group 3

Isosurface: (emnc.normB) (T) Max/Min Volume: Magnetic flux density norm (T)
