# Supplementary figures and images for: Rotatable Small Permanent Magnet Array for Ultra-Low Field Nuclear Magnetic Resonance Instrumentation: A Concept Study (part 1 of 2)
Source: PLoS One. 2016 Jun 6;11(6):e0157040. doi: 10.1371/journal.pone.0157040 (PMC4894570; doi:10.1371/journal.pone.0157040)

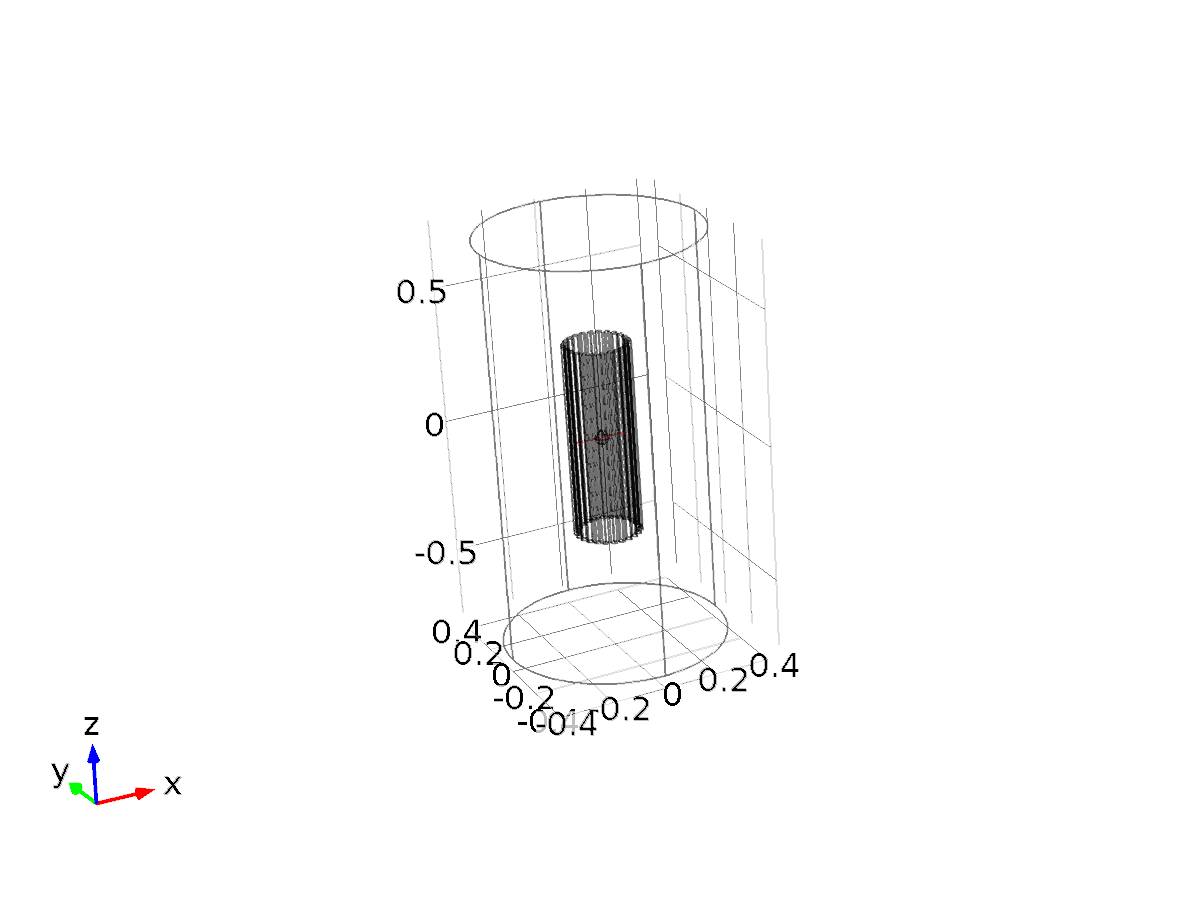

Supplement: S1 File — Model documentation generated by COMSOL with implemented parameters for the SPMA. (ZIP) [file pone.0157040.s001.zip › HalBachArray24CylindricalMag_files/dataset_cln1.png]

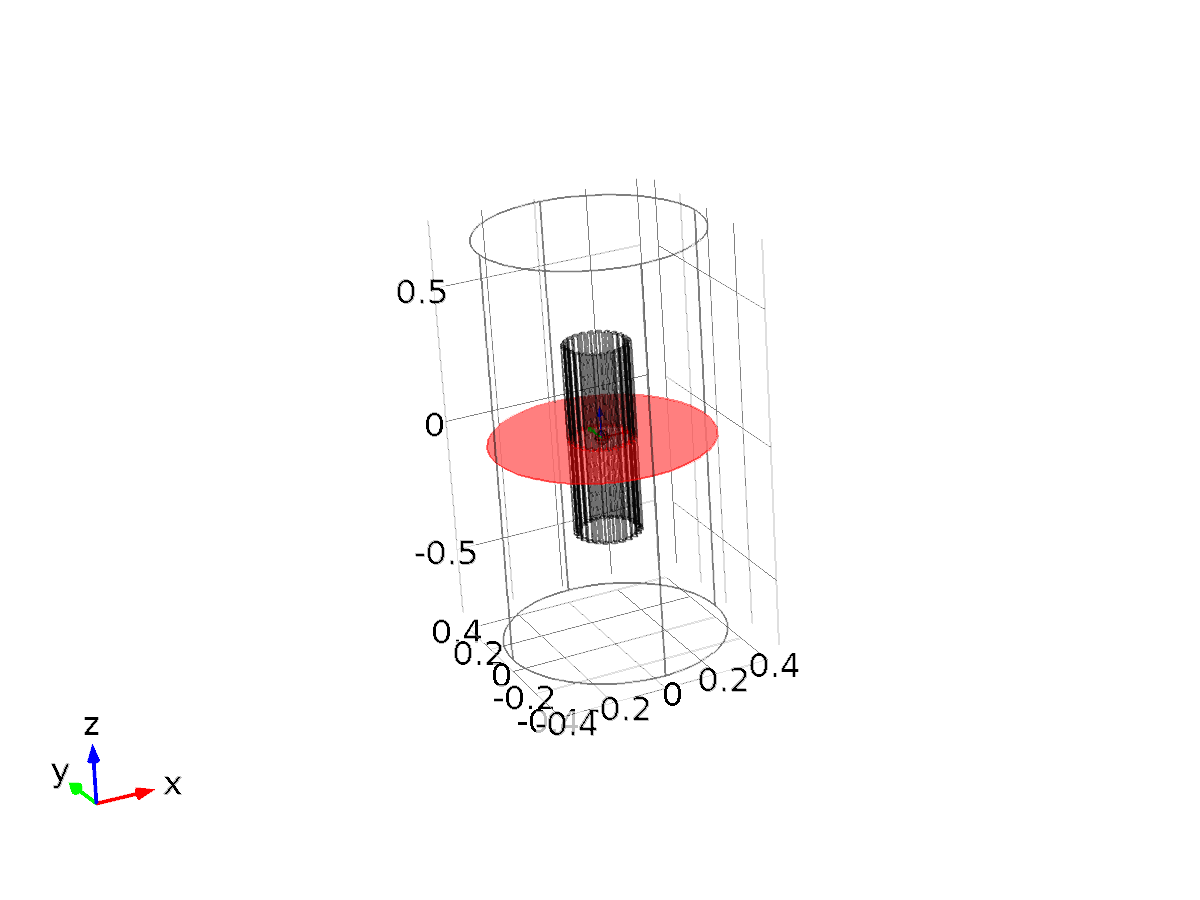

Supplement: S1 File — Model documentation generated by COMSOL with implemented parameters for the SPMA. (ZIP) [file pone.0157040.s001.zip › HalBachArray24CylindricalMag_files/dataset_cpl1.png]

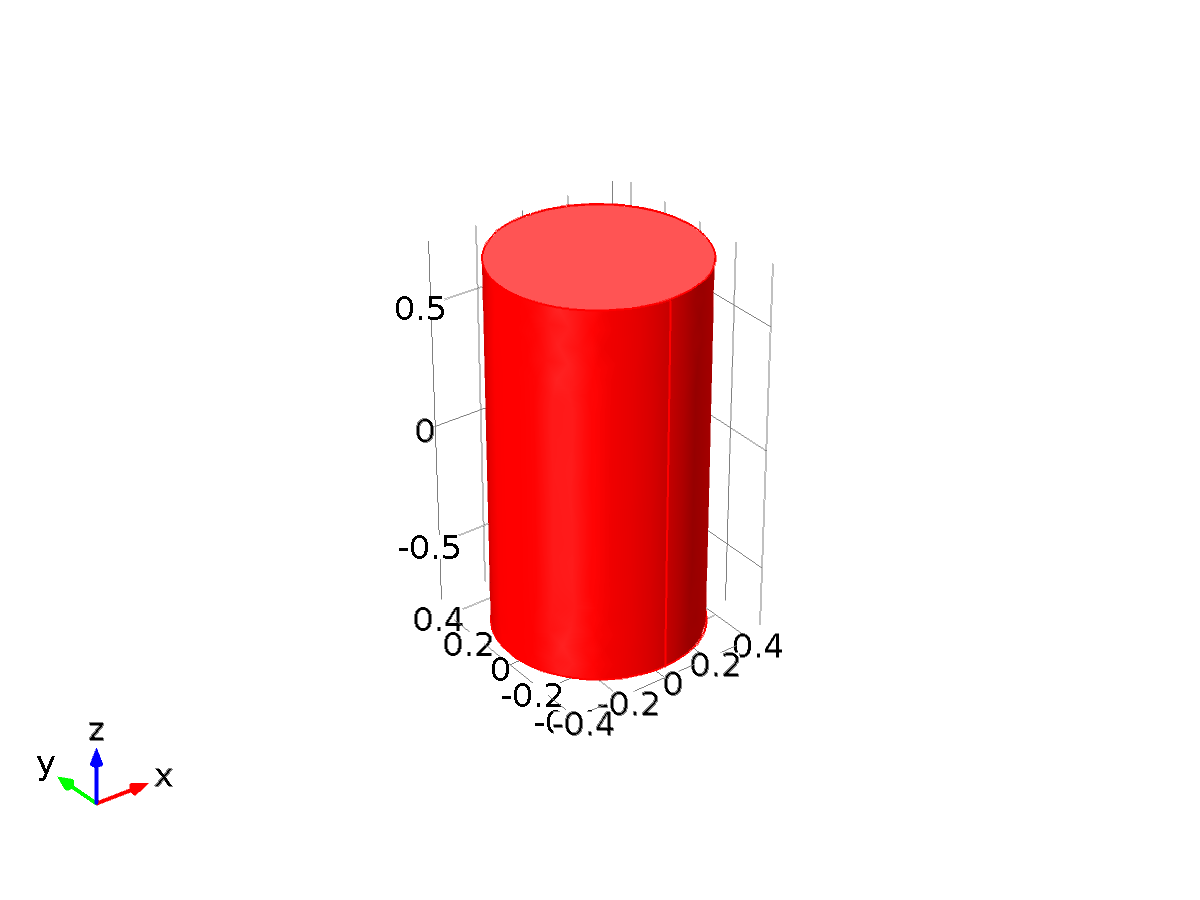

Supplement: S1 File — Model documentation generated by COMSOL with implemented parameters for the SPMA. (ZIP) [file pone.0157040.s001.zip › HalBachArray24CylindricalMag_files/dataset_dset1.png]

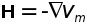

Supplement: S1 File — Model documentation generated by COMSOL with implemented parameters for the SPMA. (ZIP) [file pone.0157040.s001.zip › HalBachArray24CylindricalMag_files/equ_emnc_1.png]

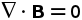

Supplement: S1 File — Model documentation generated by COMSOL with implemented parameters for the SPMA. (ZIP) [file pone.0157040.s001.zip › HalBachArray24CylindricalMag_files/equ_emnc_2.png]

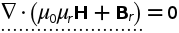

Supplement: S1 File — Model documentation generated by COMSOL with implemented parameters for the SPMA. (ZIP) [file pone.0157040.s001.zip › HalBachArray24CylindricalMag_files/equ_emnc_mfc10_2.png]

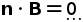

Supplement: S1 File — Model documentation generated by COMSOL with implemented parameters for the SPMA. (ZIP) [file pone.0157040.s001.zip › HalBachArray24CylindricalMag_files/equ_emnc_mi1.png]

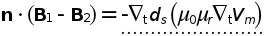

Supplement: S1 File — Model documentation generated by COMSOL with implemented parameters for the SPMA. (ZIP) [file pone.0157040.s001.zip › HalBachArray24CylindricalMag_files/equ_emnc_ms1.png]

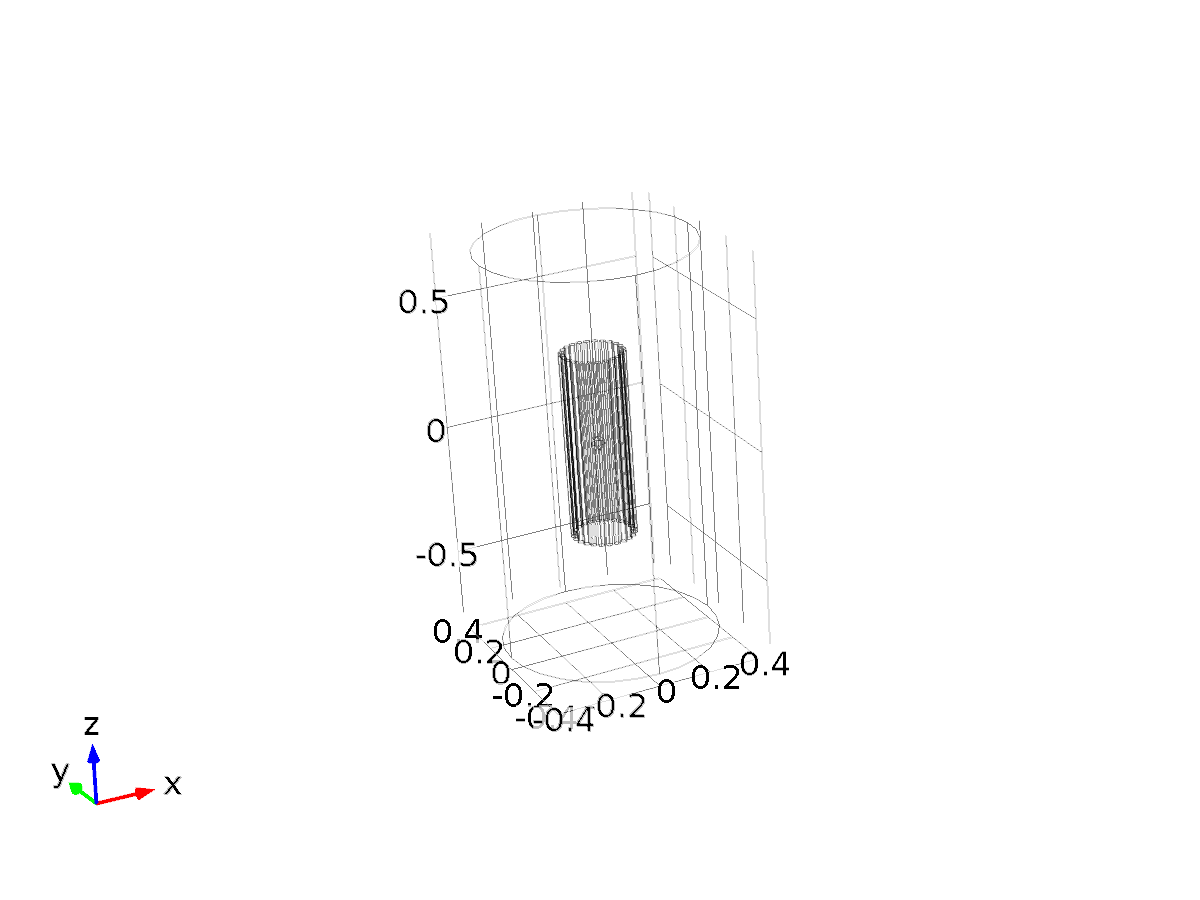

Supplement: S1 File — Model documentation generated by COMSOL with implemented parameters for the SPMA. (ZIP) [file pone.0157040.s001.zip › HalBachArray24CylindricalMag_files/geom_geom1.png]

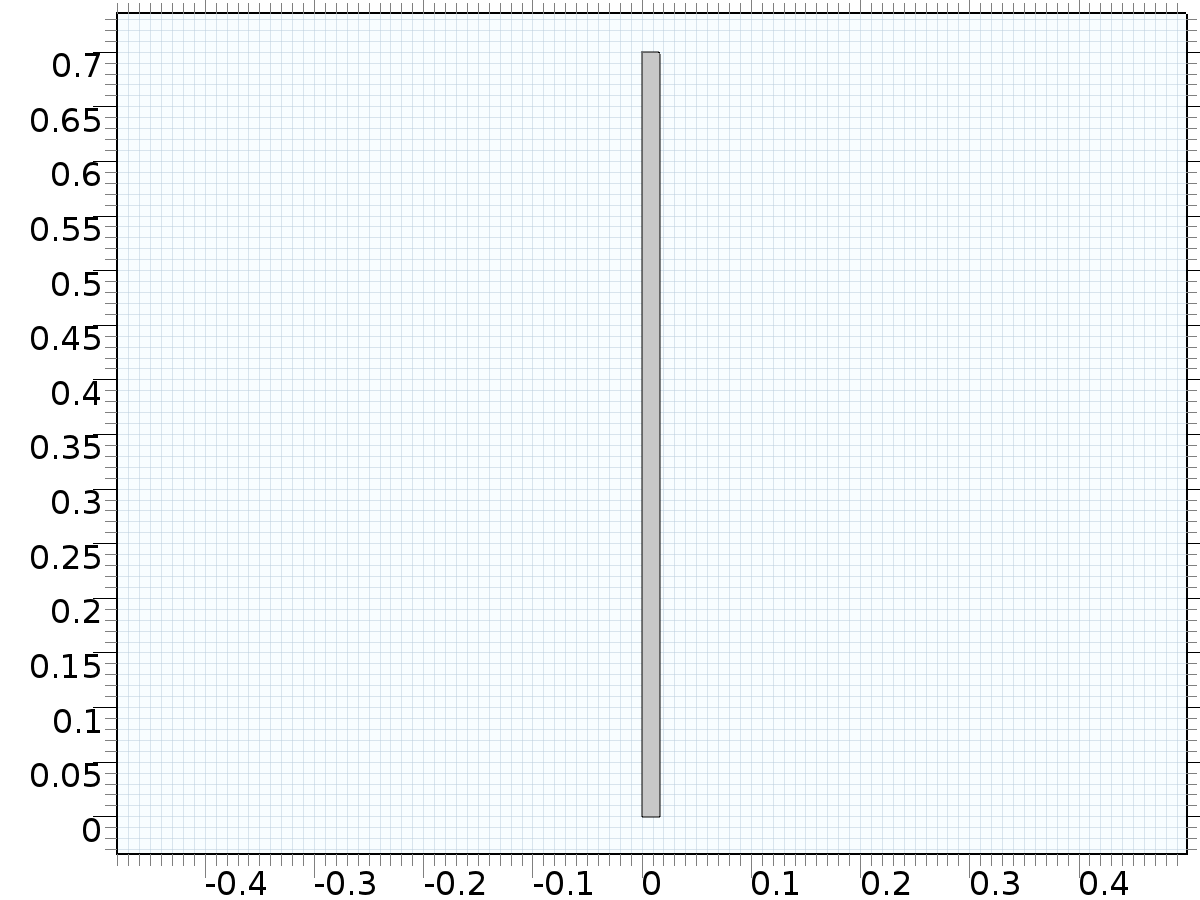

Supplement: S1 File — Model documentation generated by COMSOL with implemented parameters for the SPMA. (ZIP) [file pone.0157040.s001.zip › HalBachArray24CylindricalMag_files/geom_geom2.png]

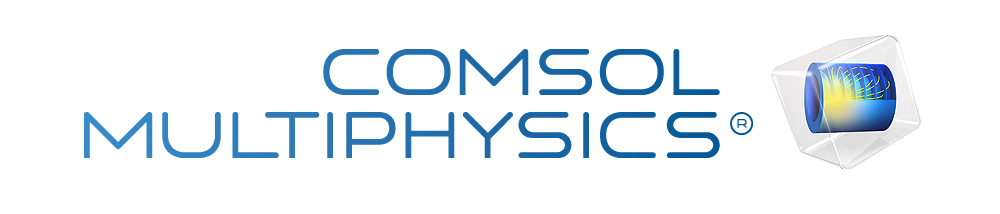

Supplement: S1 File — Model documentation generated by COMSOL with implemented parameters for the SPMA. (ZIP) [file pone.0157040.s001.zip › HalBachArray24CylindricalMag_files/logo.png]

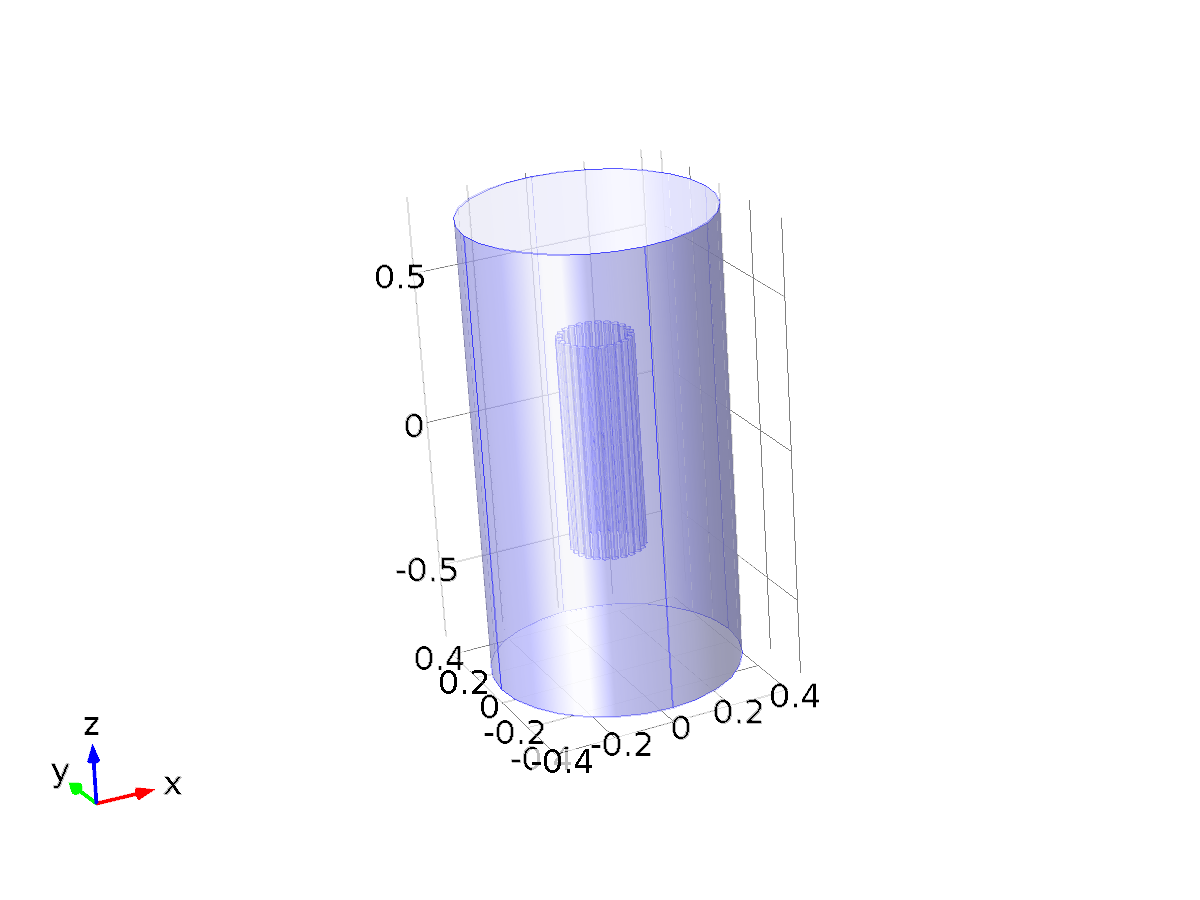

Supplement: S1 File — Model documentation generated by COMSOL with implemented parameters for the SPMA. (ZIP) [file pone.0157040.s001.zip › HalBachArray24CylindricalMag_files/material_mat1.png]

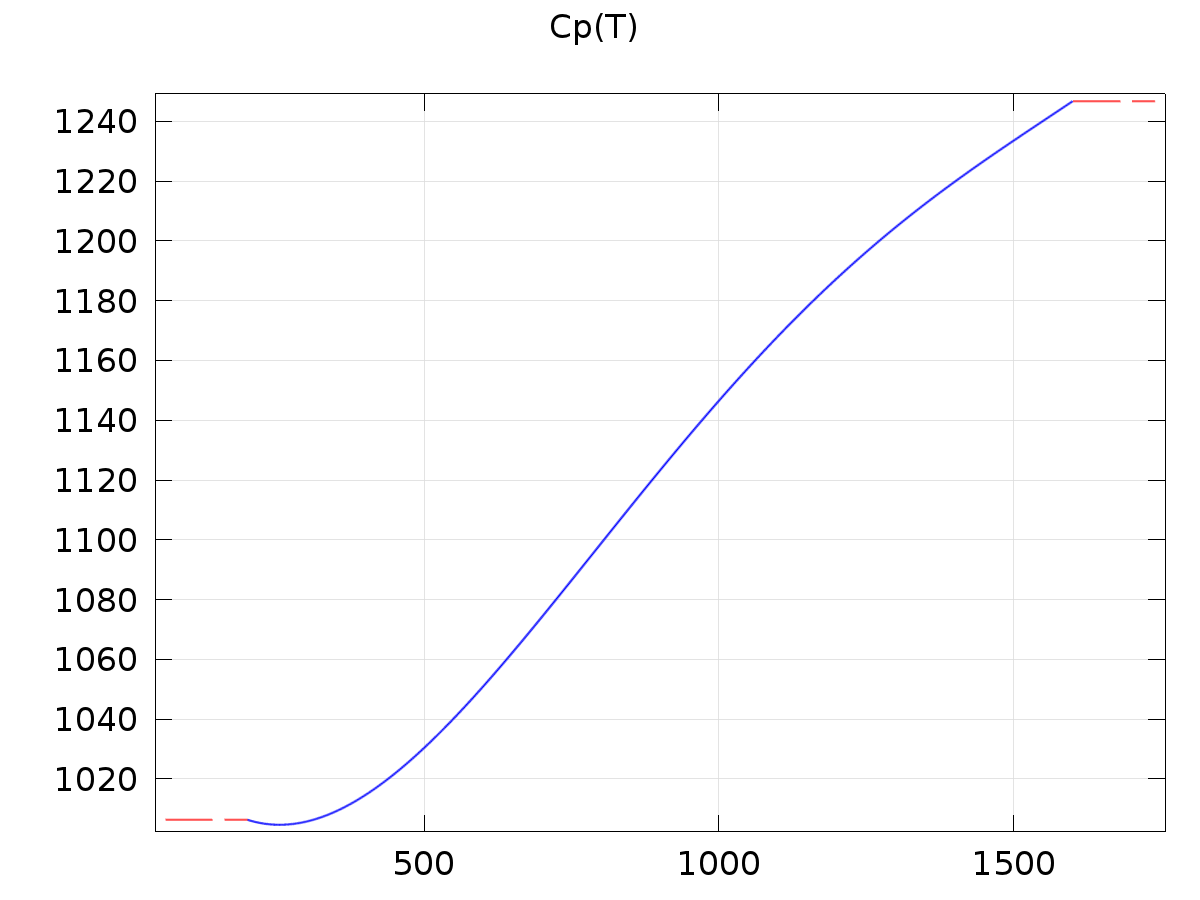

Supplement: S1 File — Model documentation generated by COMSOL with implemented parameters for the SPMA. (ZIP) [file pone.0157040.s001.zip › HalBachArray24CylindricalMag_files/material_mat1_def_Cp.png]

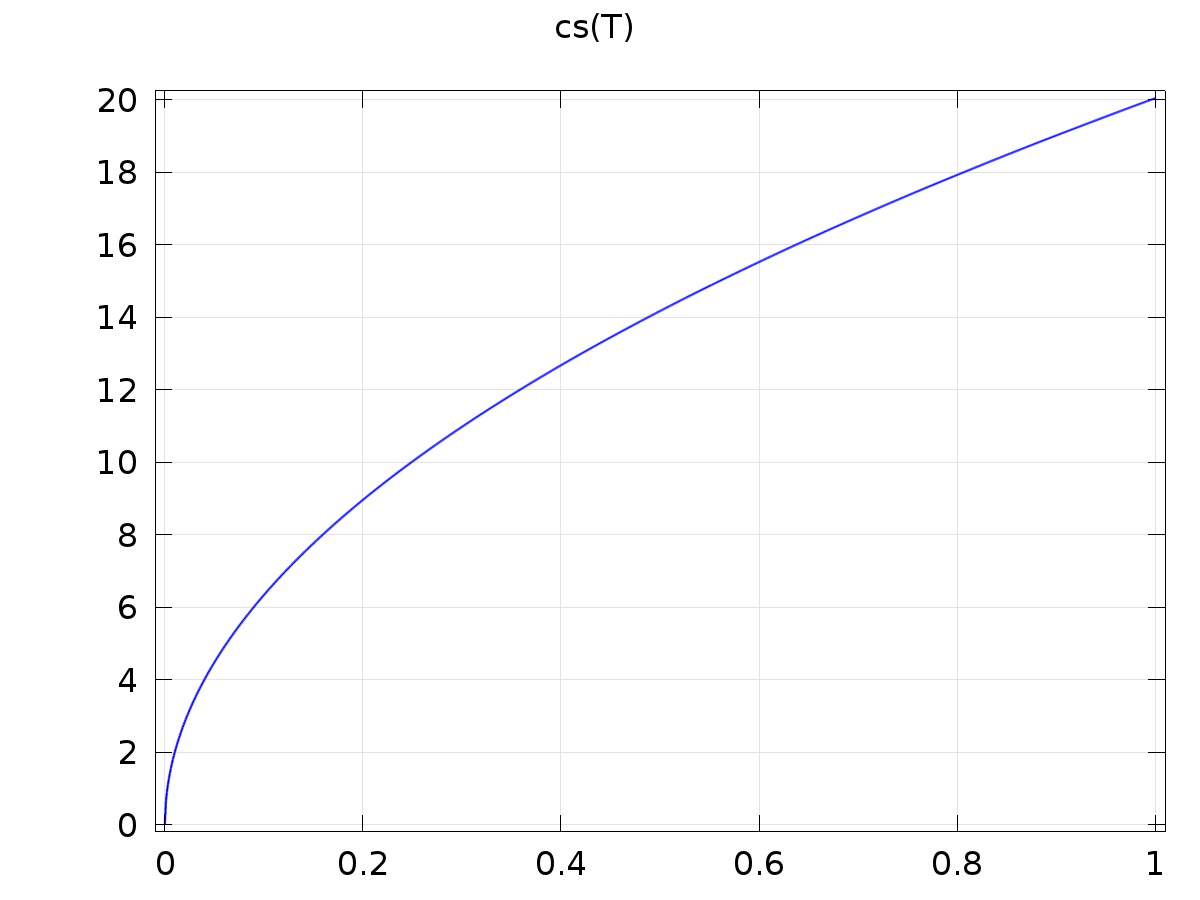

Supplement: S1 File — Model documentation generated by COMSOL with implemented parameters for the SPMA. (ZIP) [file pone.0157040.s001.zip › HalBachArray24CylindricalMag_files/material_mat1_def_cs.png]

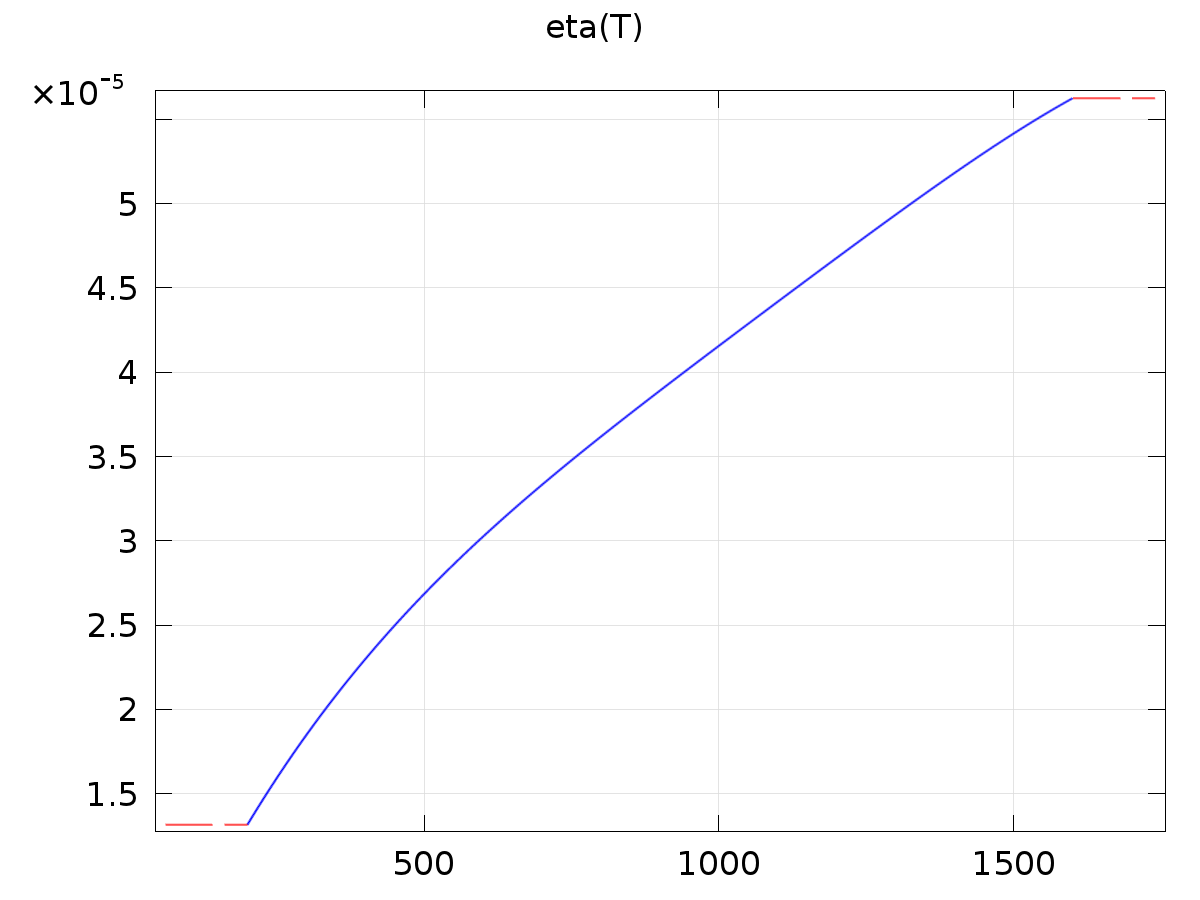

Supplement: S1 File — Model documentation generated by COMSOL with implemented parameters for the SPMA. (ZIP) [file pone.0157040.s001.zip › HalBachArray24CylindricalMag_files/material_mat1_def_eta.png]

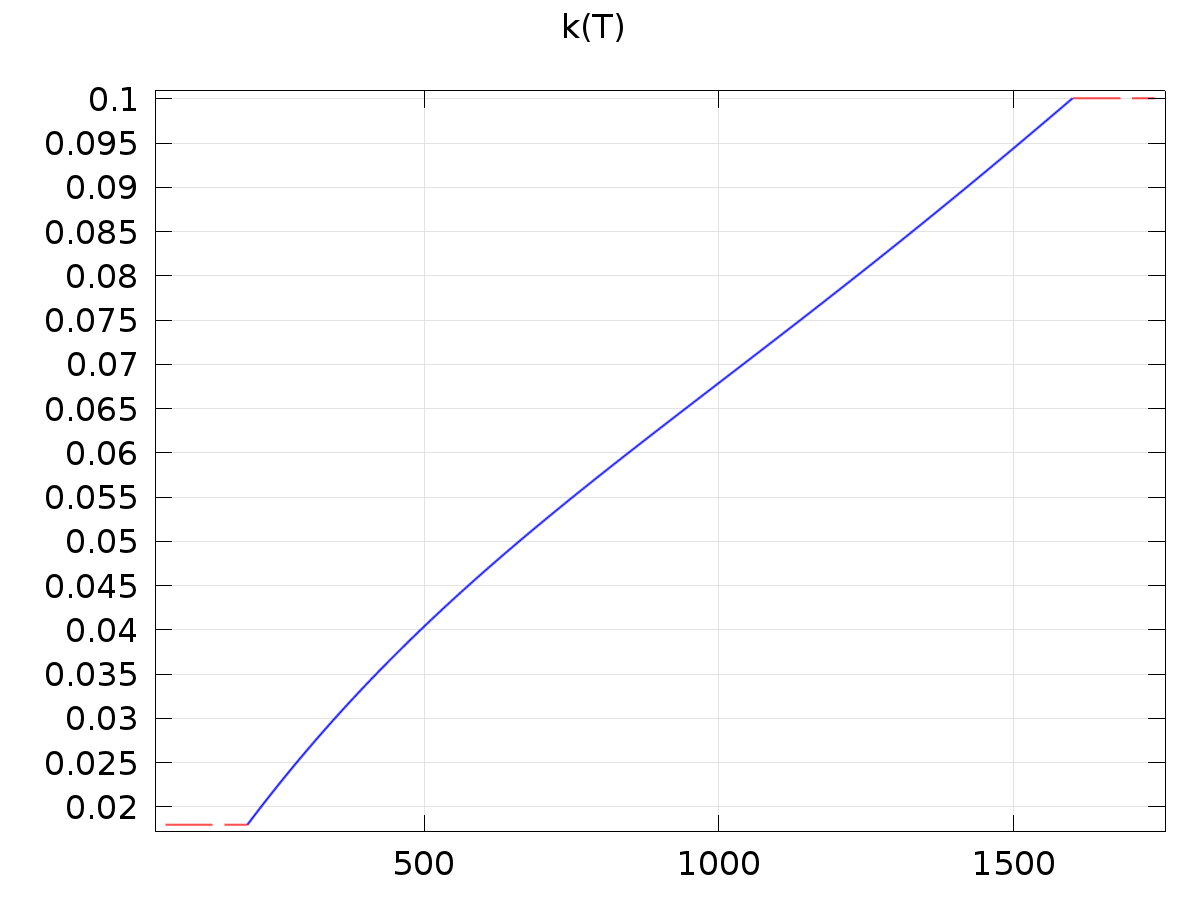

Supplement: S1 File — Model documentation generated by COMSOL with implemented parameters for the SPMA. (ZIP) [file pone.0157040.s001.zip › HalBachArray24CylindricalMag_files/material_mat1_def_k.png]

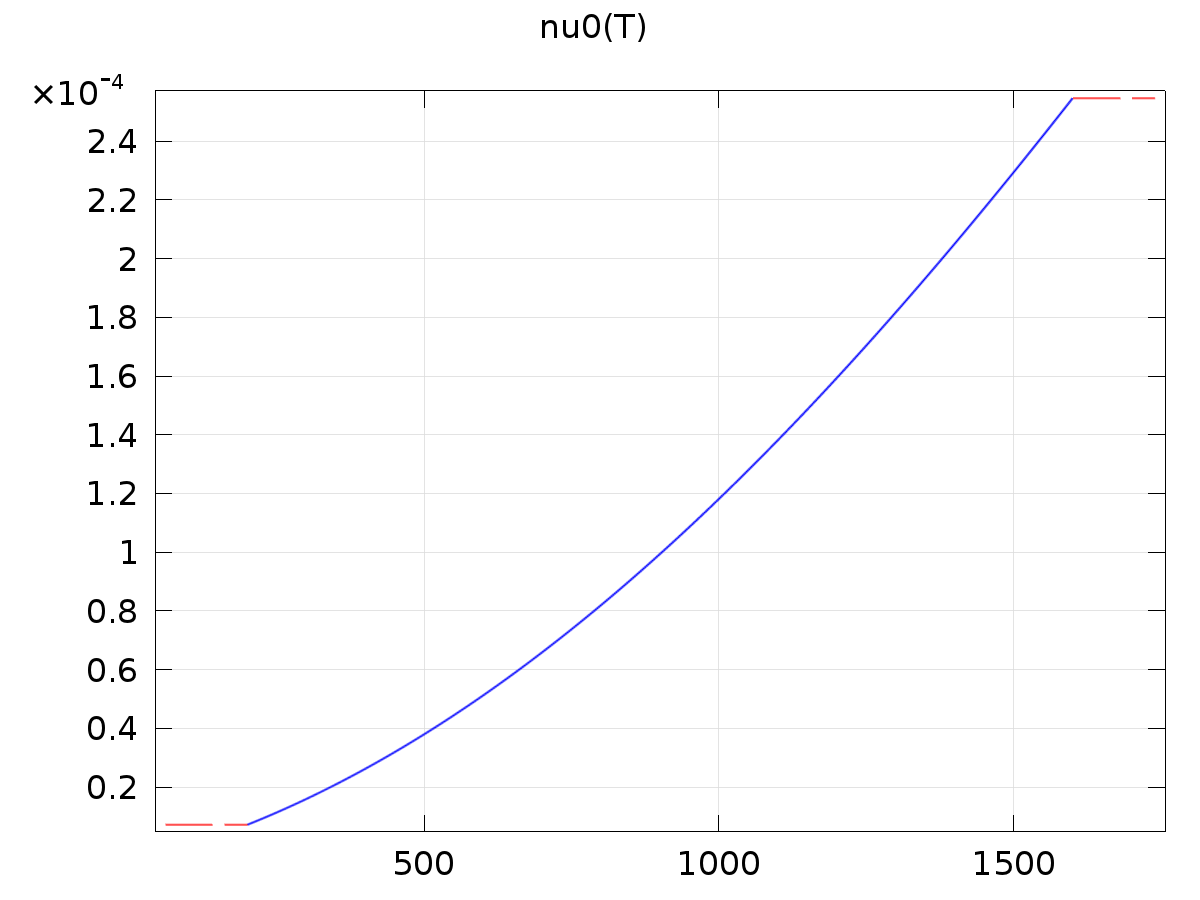

Supplement: S1 File — Model documentation generated by COMSOL with implemented parameters for the SPMA. (ZIP) [file pone.0157040.s001.zip › HalBachArray24CylindricalMag_files/material_mat1_def_nu0.png]

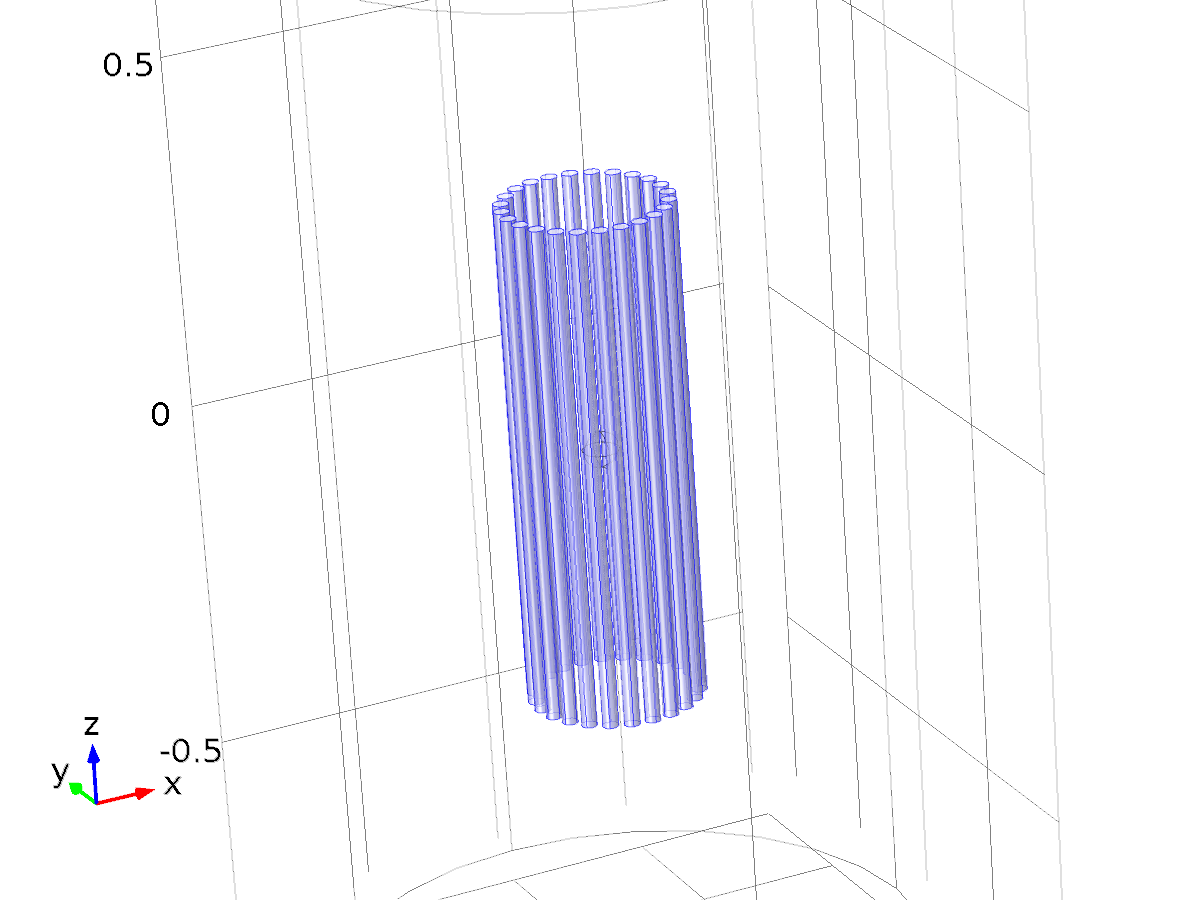

Supplement: S1 File — Model documentation generated by COMSOL with implemented parameters for the SPMA. (ZIP) [file pone.0157040.s001.zip › HalBachArray24CylindricalMag_files/material_mat2.png]

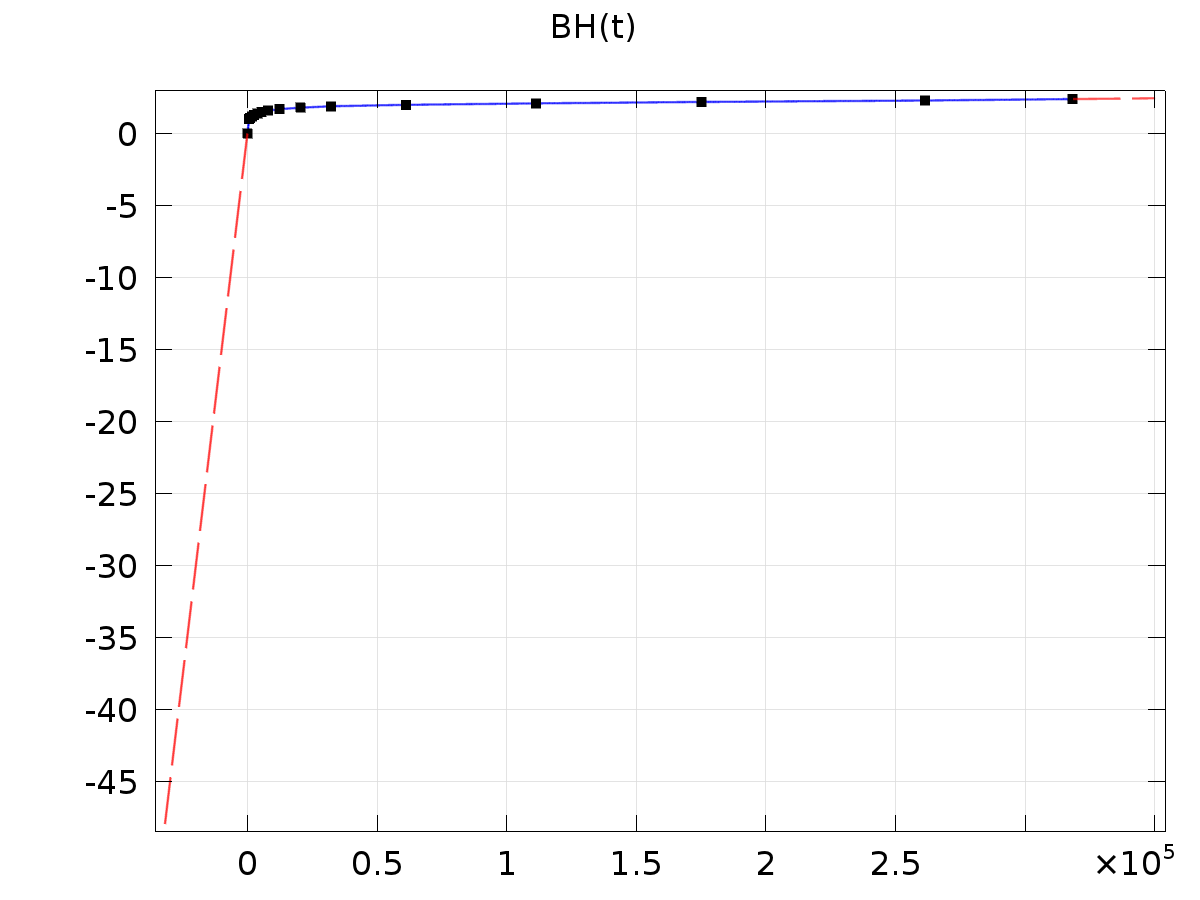

Supplement: S1 File — Model documentation generated by COMSOL with implemented parameters for the SPMA. (ZIP) [file pone.0157040.s001.zip › HalBachArray24CylindricalMag_files/material_mat2_BHCurve_BH.png]

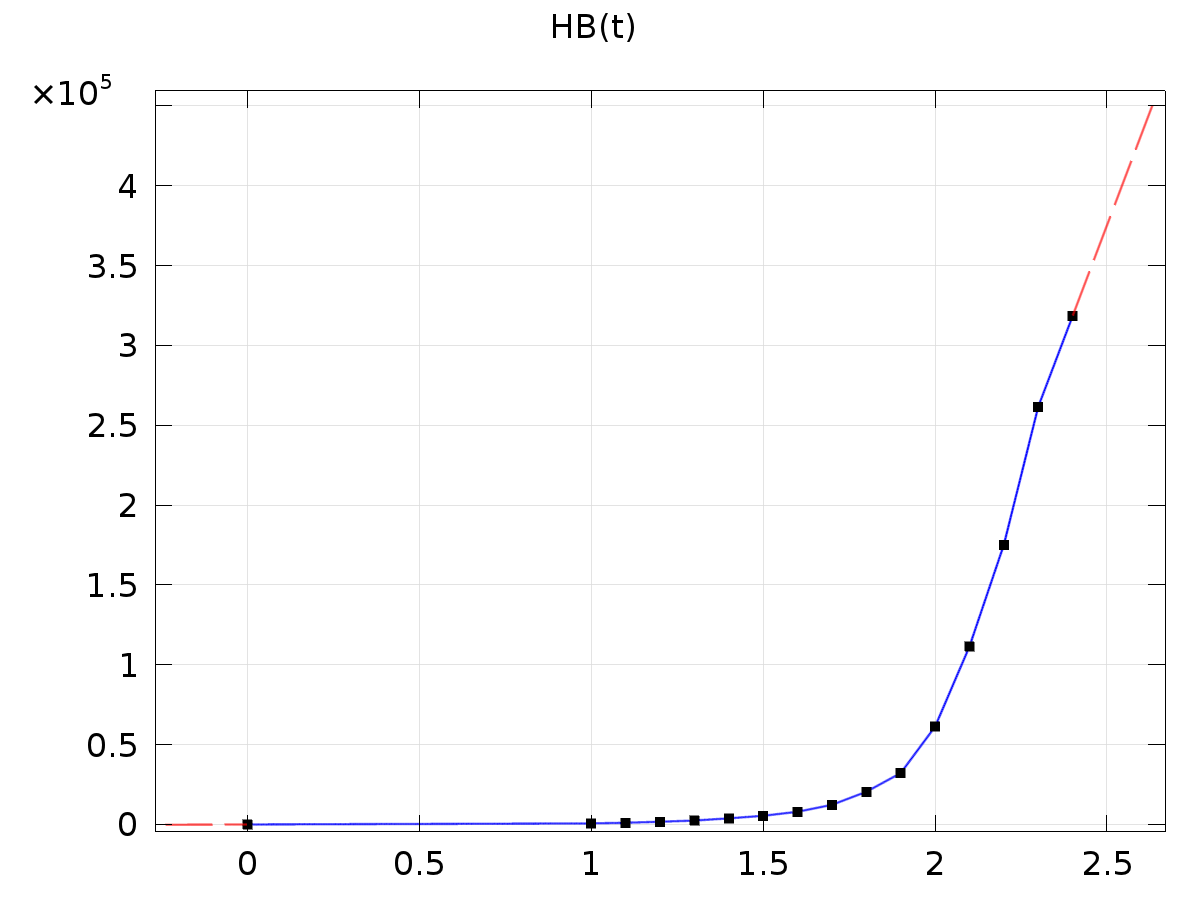

Supplement: S1 File — Model documentation generated by COMSOL with implemented parameters for the SPMA. (ZIP) [file pone.0157040.s001.zip › HalBachArray24CylindricalMag_files/material_mat2_HBCurve_HB.png]

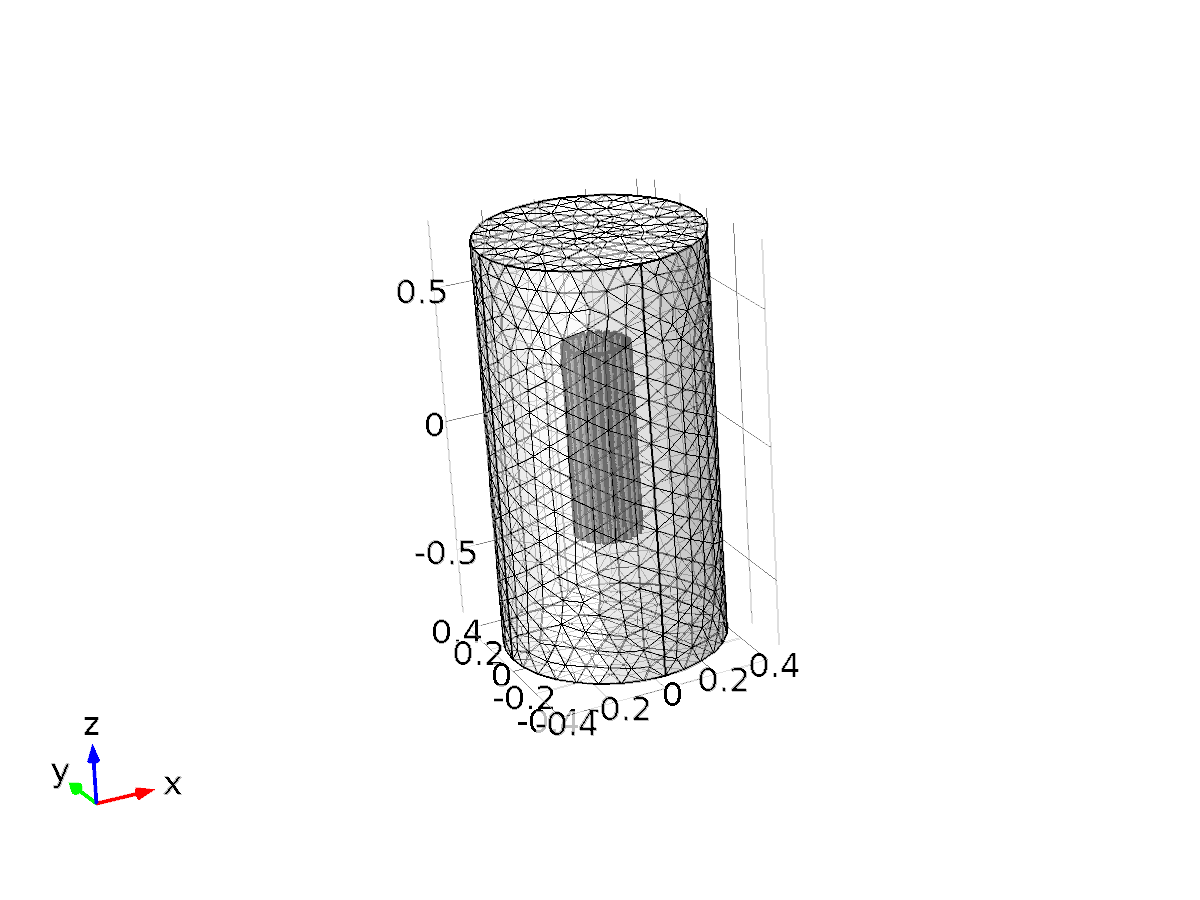

Supplement: S1 File — Model documentation generated by COMSOL with implemented parameters for the SPMA. (ZIP) [file pone.0157040.s001.zip › HalBachArray24CylindricalMag_files/mesh_mesh1.png]

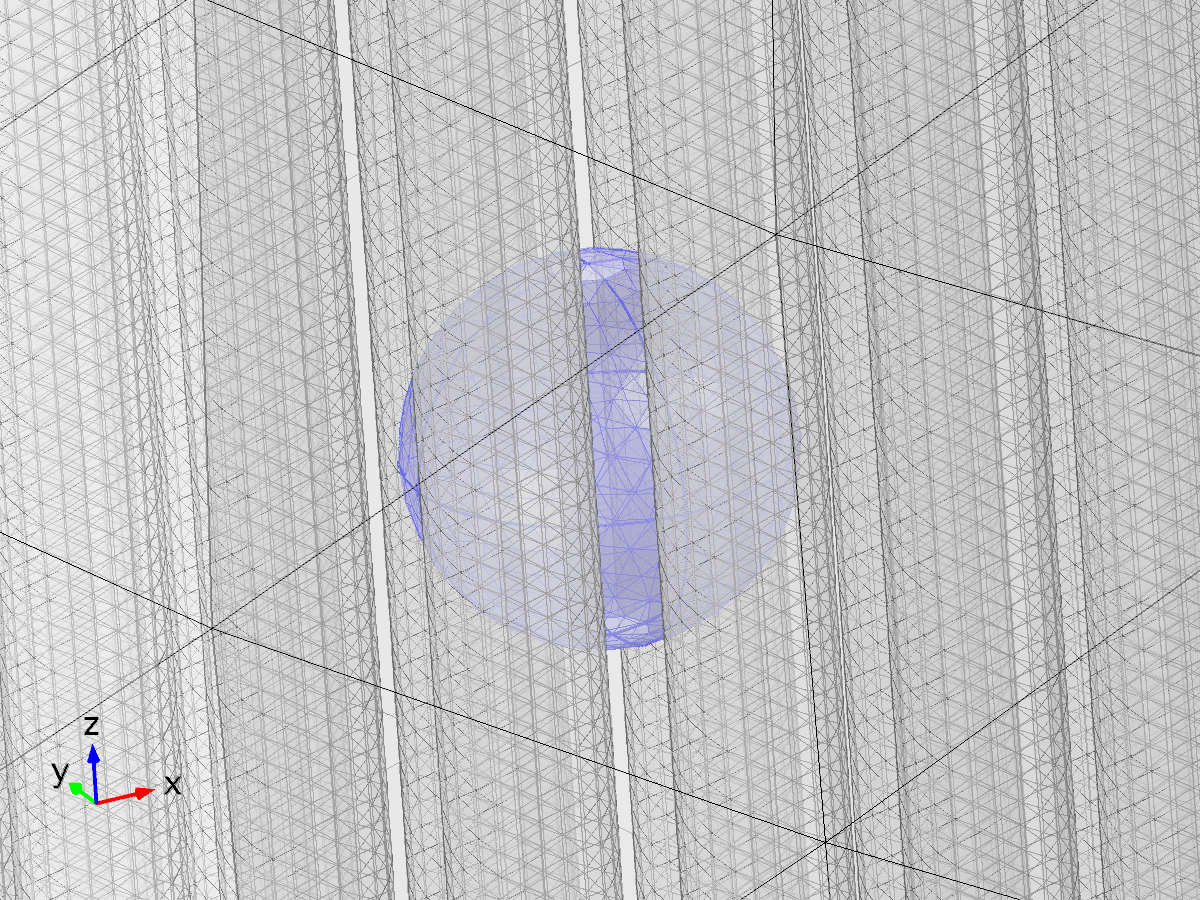

Supplement: S1 File — Model documentation generated by COMSOL with implemented parameters for the SPMA. (ZIP) [file pone.0157040.s001.zip › HalBachArray24CylindricalMag_files/mesh_mesh1_ftet1.png]

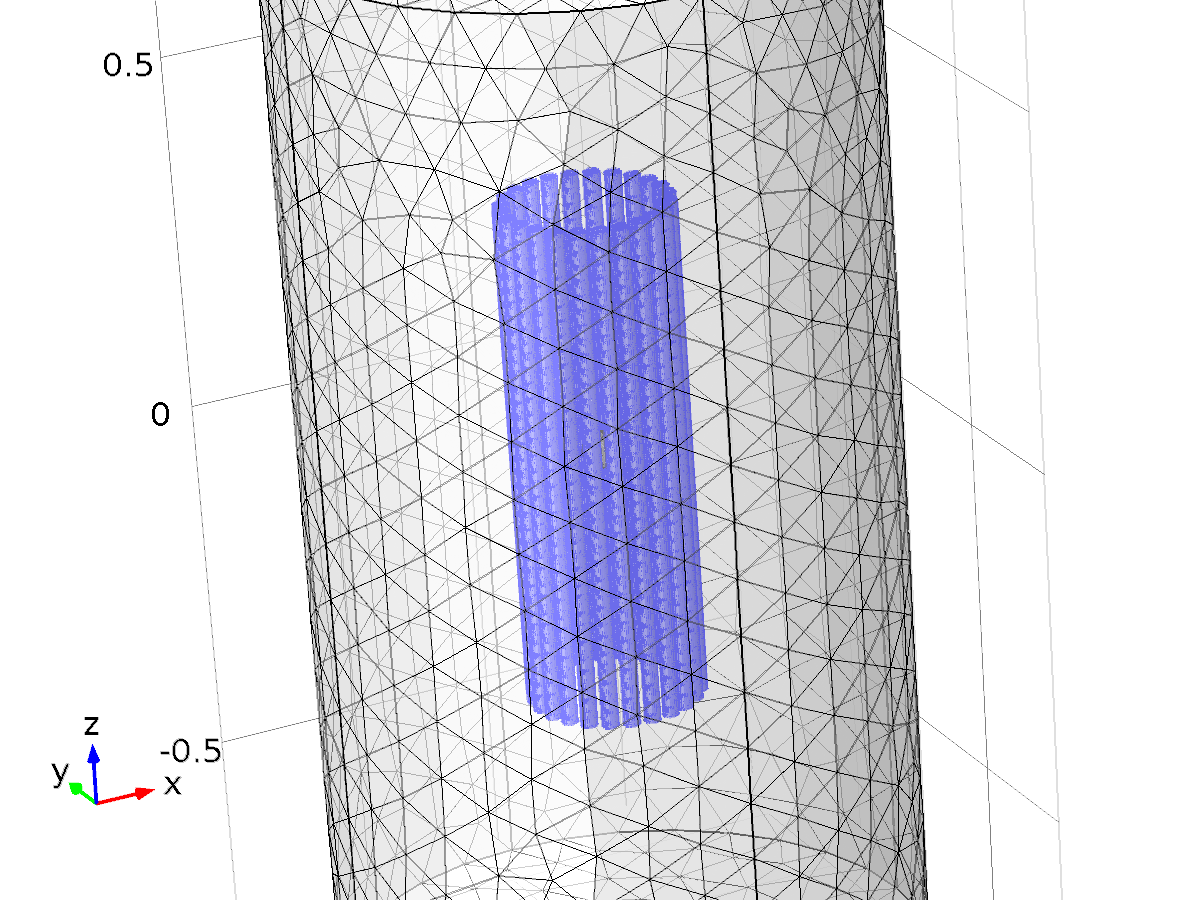

Supplement: S1 File — Model documentation generated by COMSOL with implemented parameters for the SPMA. (ZIP) [file pone.0157040.s001.zip › HalBachArray24CylindricalMag_files/mesh_mesh1_ftet2_size1.png]

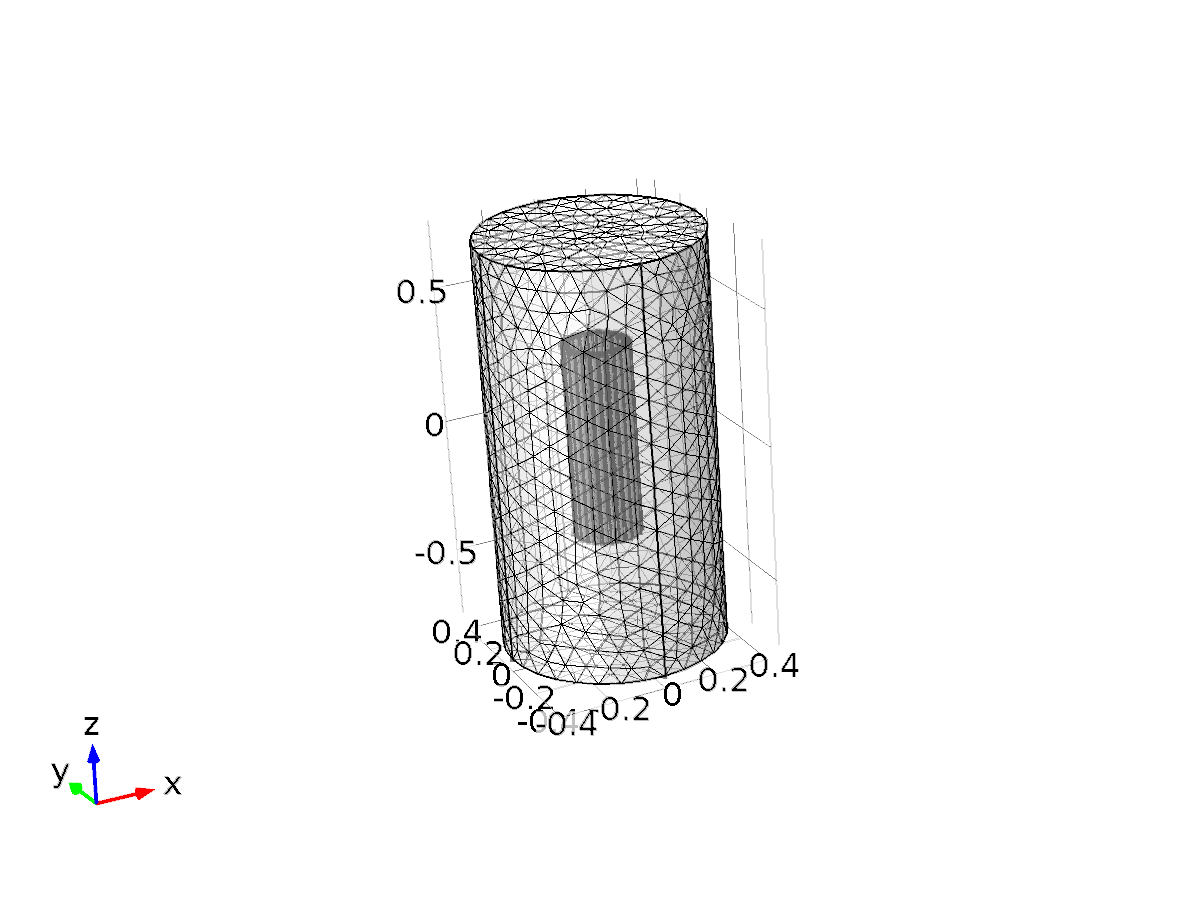

Supplement: S1 File — Model documentation generated by COMSOL with implemented parameters for the SPMA. (ZIP) [file pone.0157040.s001.zip › HalBachArray24CylindricalMag_files/mesh_mesh1_ftet3_size1.png]

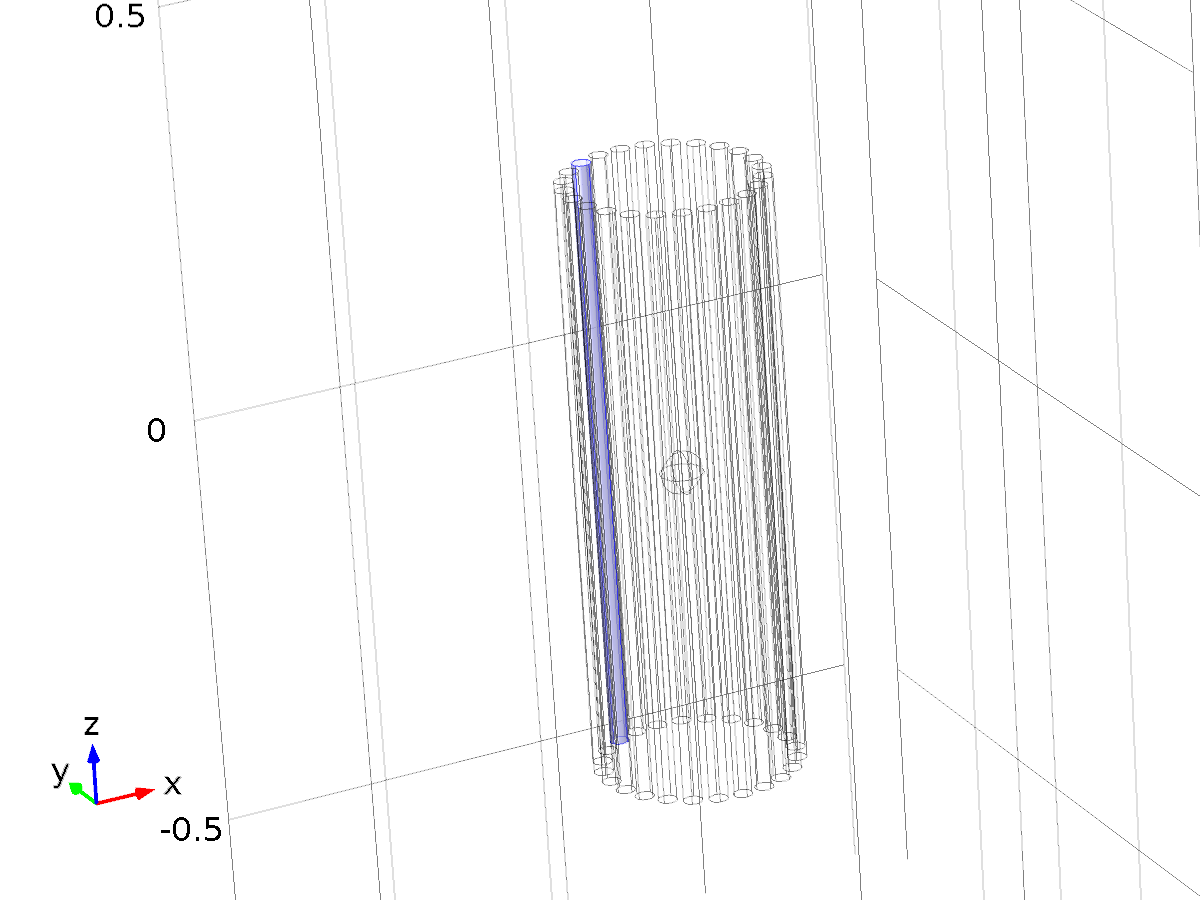

Supplement: S1 File — Model documentation generated by COMSOL with implemented parameters for the SPMA. (ZIP) [file pone.0157040.s001.zip › HalBachArray24CylindricalMag_files/physics_emnc_mfc10.png]

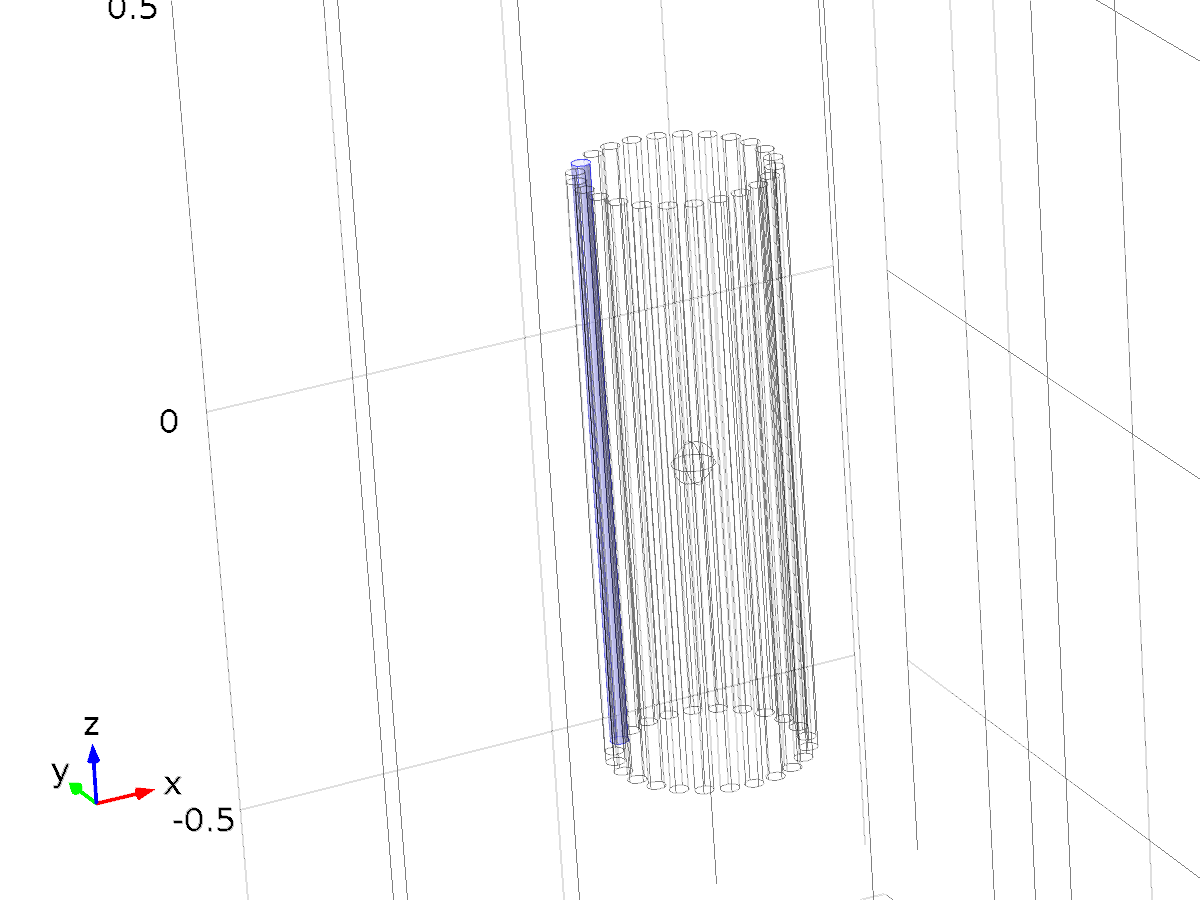

Supplement: S1 File — Model documentation generated by COMSOL with implemented parameters for the SPMA. (ZIP) [file pone.0157040.s001.zip › HalBachArray24CylindricalMag_files/physics_emnc_mfc11.png]

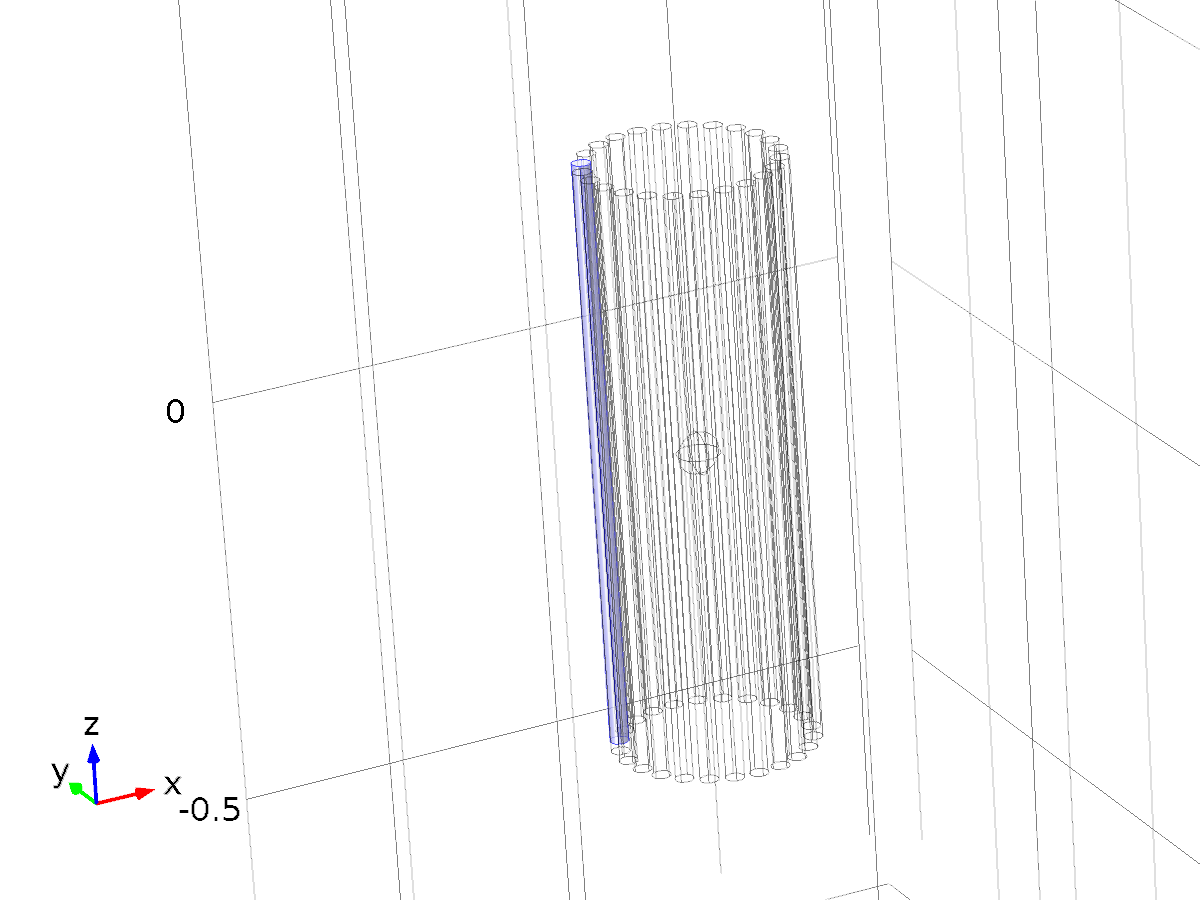

Supplement: S1 File — Model documentation generated by COMSOL with implemented parameters for the SPMA. (ZIP) [file pone.0157040.s001.zip › HalBachArray24CylindricalMag_files/physics_emnc_mfc12.png]

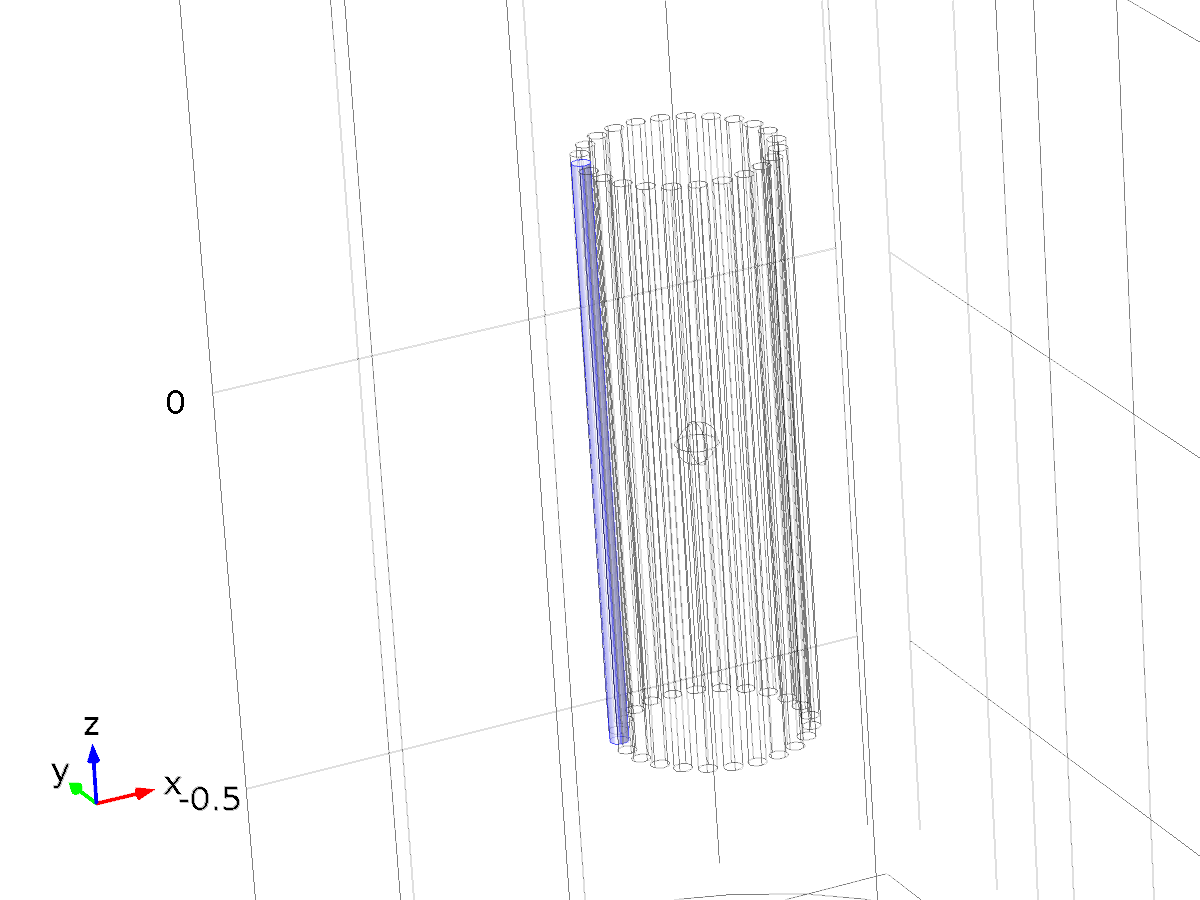

Supplement: S1 File — Model documentation generated by COMSOL with implemented parameters for the SPMA. (ZIP) [file pone.0157040.s001.zip › HalBachArray24CylindricalMag_files/physics_emnc_mfc13.png]

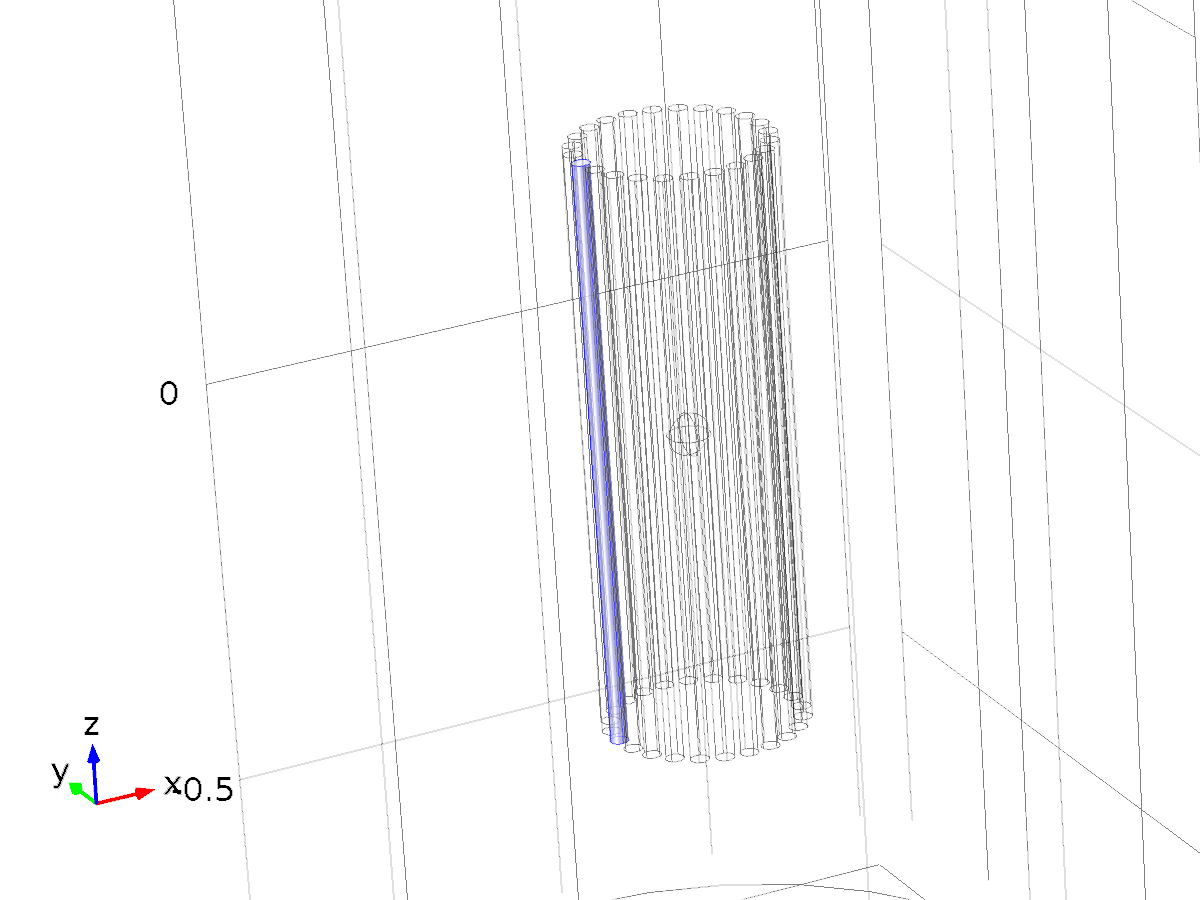

Supplement: S1 File — Model documentation generated by COMSOL with implemented parameters for the SPMA. (ZIP) [file pone.0157040.s001.zip › HalBachArray24CylindricalMag_files/physics_emnc_mfc14.png]

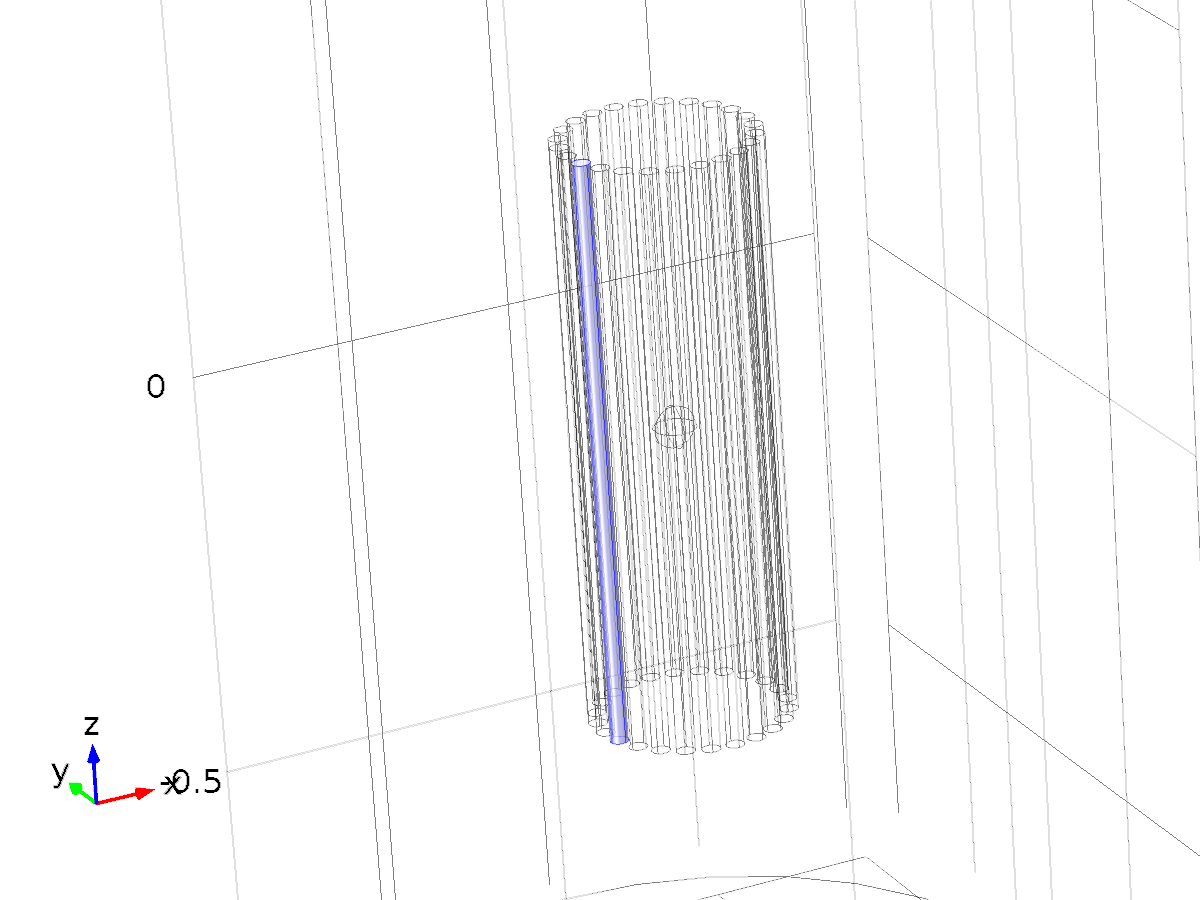

Supplement: S1 File — Model documentation generated by COMSOL with implemented parameters for the SPMA. (ZIP) [file pone.0157040.s001.zip › HalBachArray24CylindricalMag_files/physics_emnc_mfc15.png]

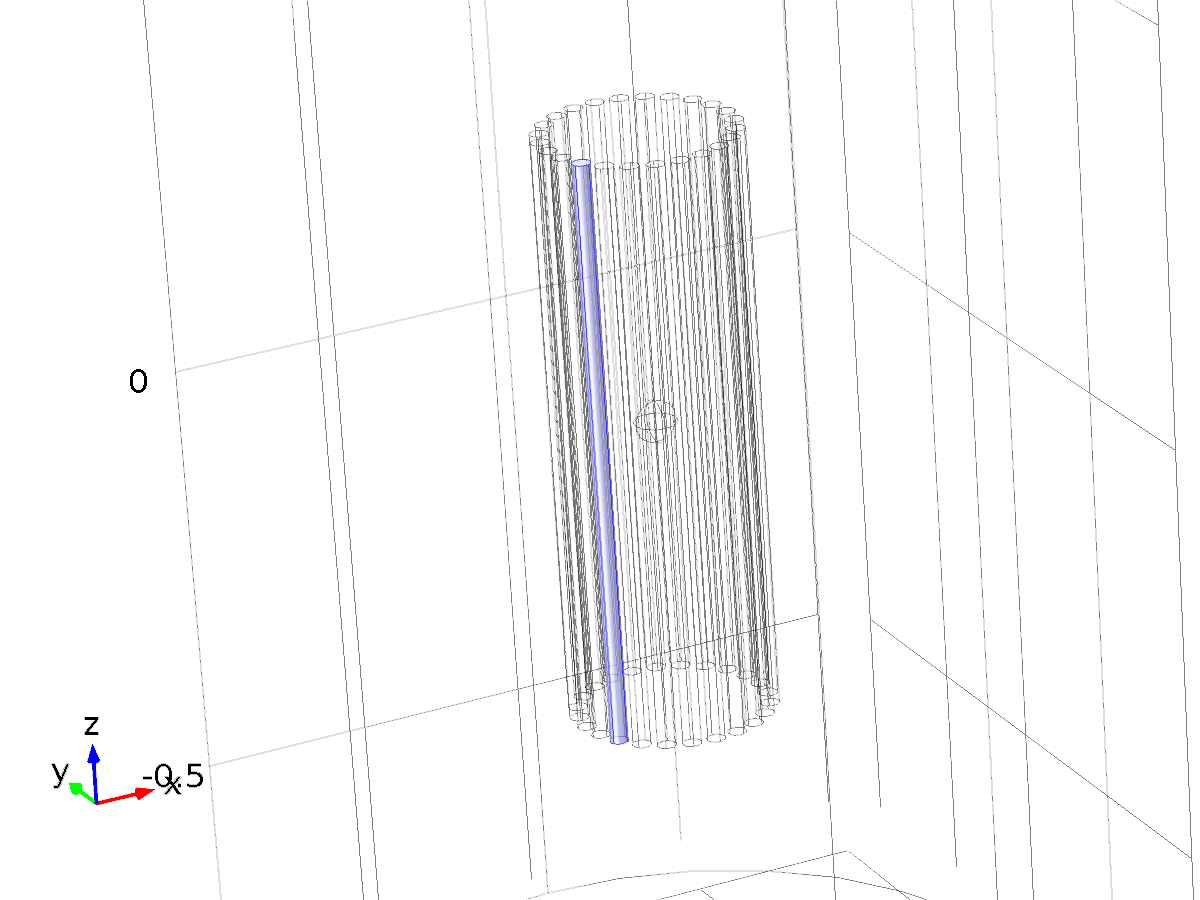

Supplement: S1 File — Model documentation generated by COMSOL with implemented parameters for the SPMA. (ZIP) [file pone.0157040.s001.zip › HalBachArray24CylindricalMag_files/physics_emnc_mfc16.png]

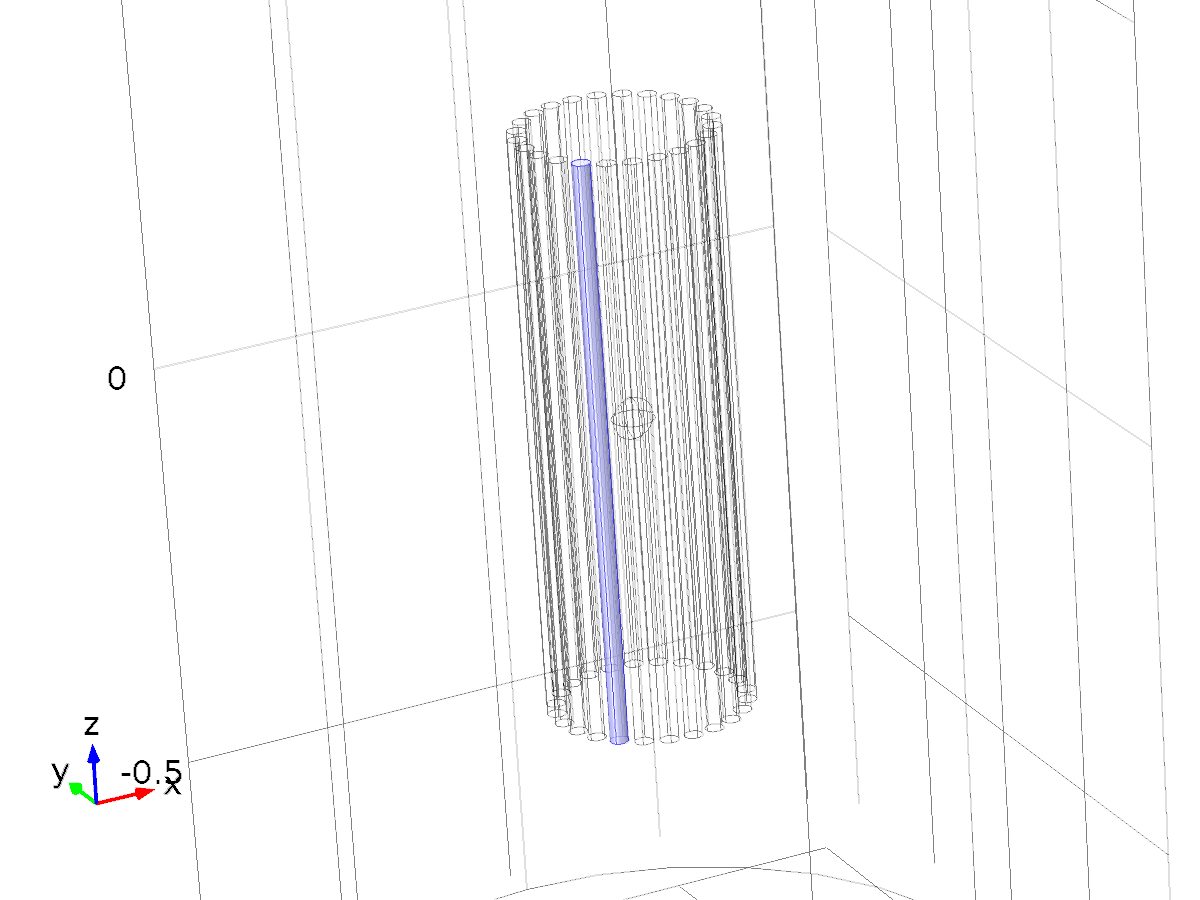

Supplement: S1 File — Model documentation generated by COMSOL with implemented parameters for the SPMA. (ZIP) [file pone.0157040.s001.zip › HalBachArray24CylindricalMag_files/physics_emnc_mfc17.png]

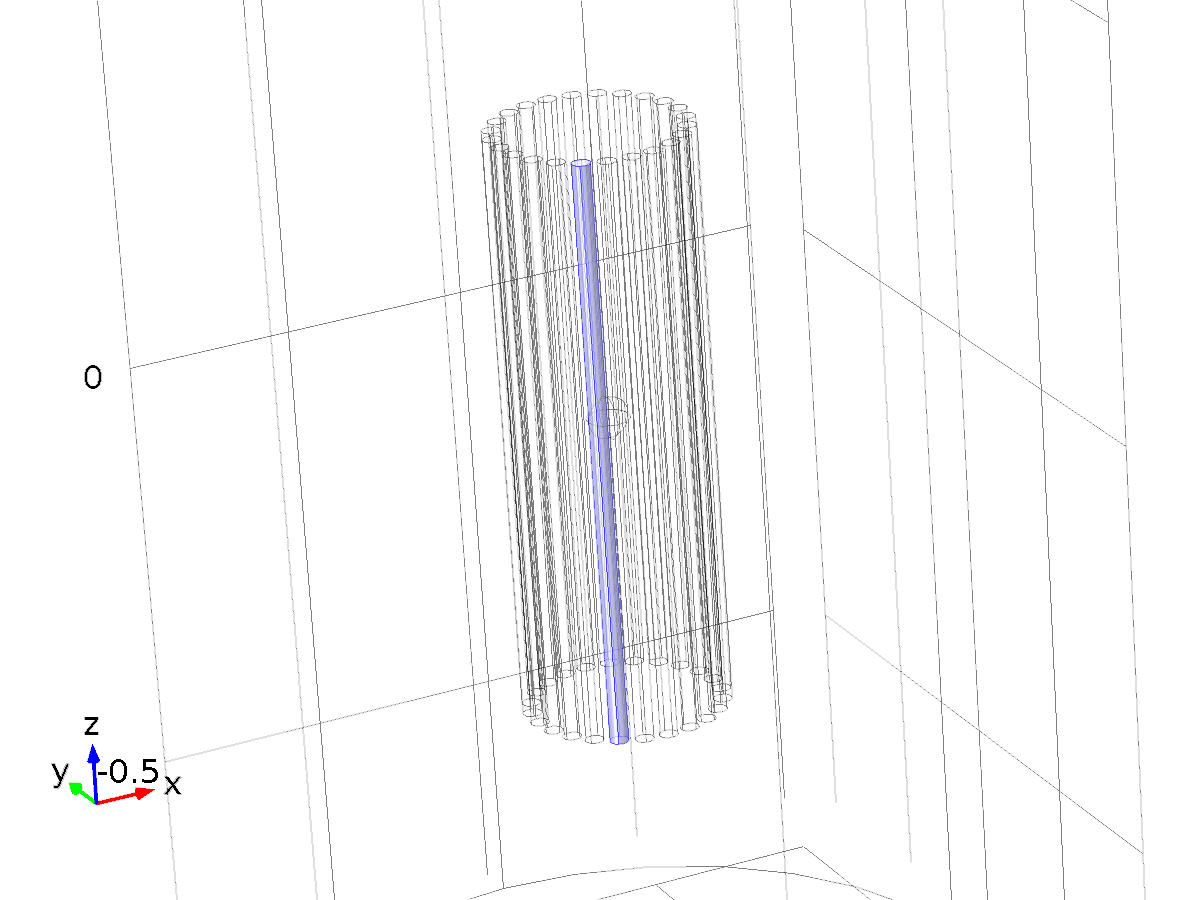

Supplement: S1 File — Model documentation generated by COMSOL with implemented parameters for the SPMA. (ZIP) [file pone.0157040.s001.zip › HalBachArray24CylindricalMag_files/physics_emnc_mfc18.png]

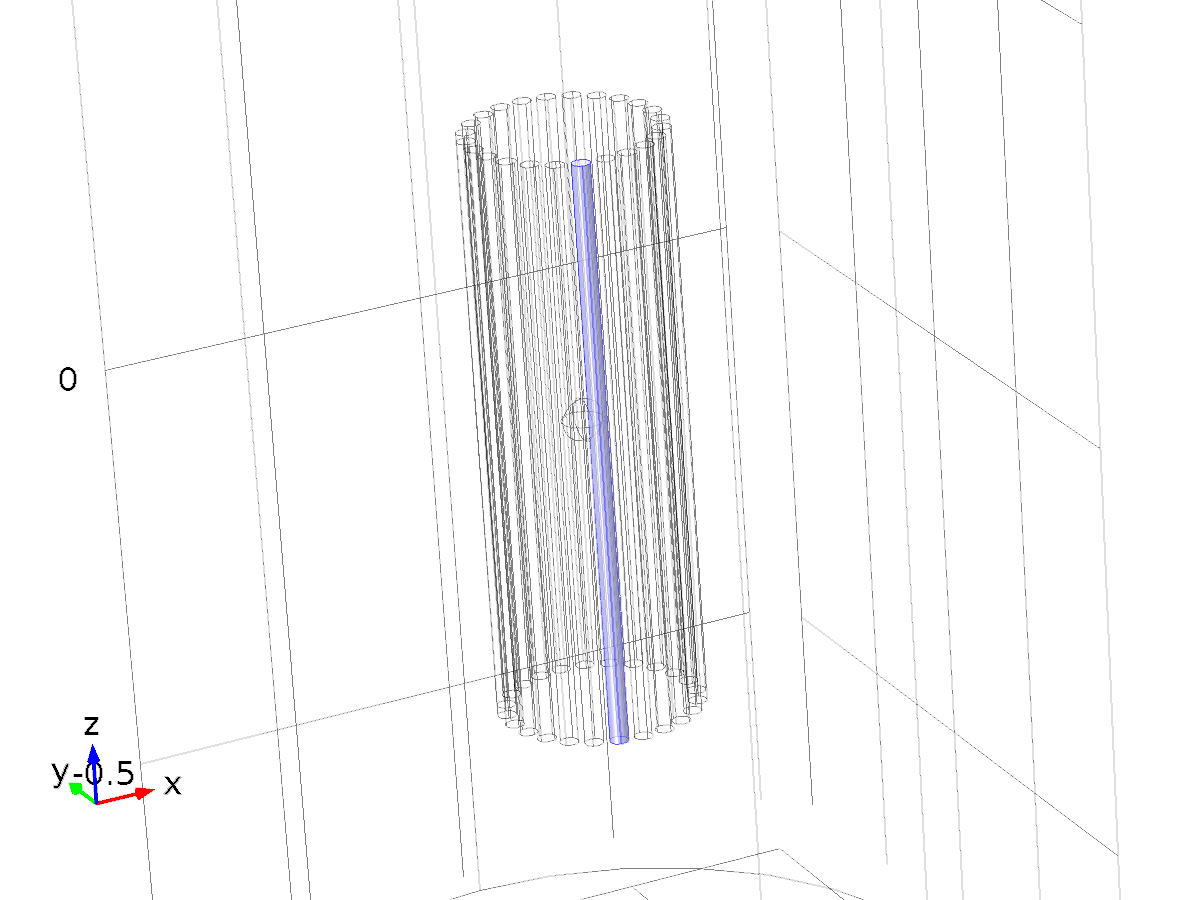

Supplement: S1 File — Model documentation generated by COMSOL with implemented parameters for the SPMA. (ZIP) [file pone.0157040.s001.zip › HalBachArray24CylindricalMag_files/physics_emnc_mfc19.png]

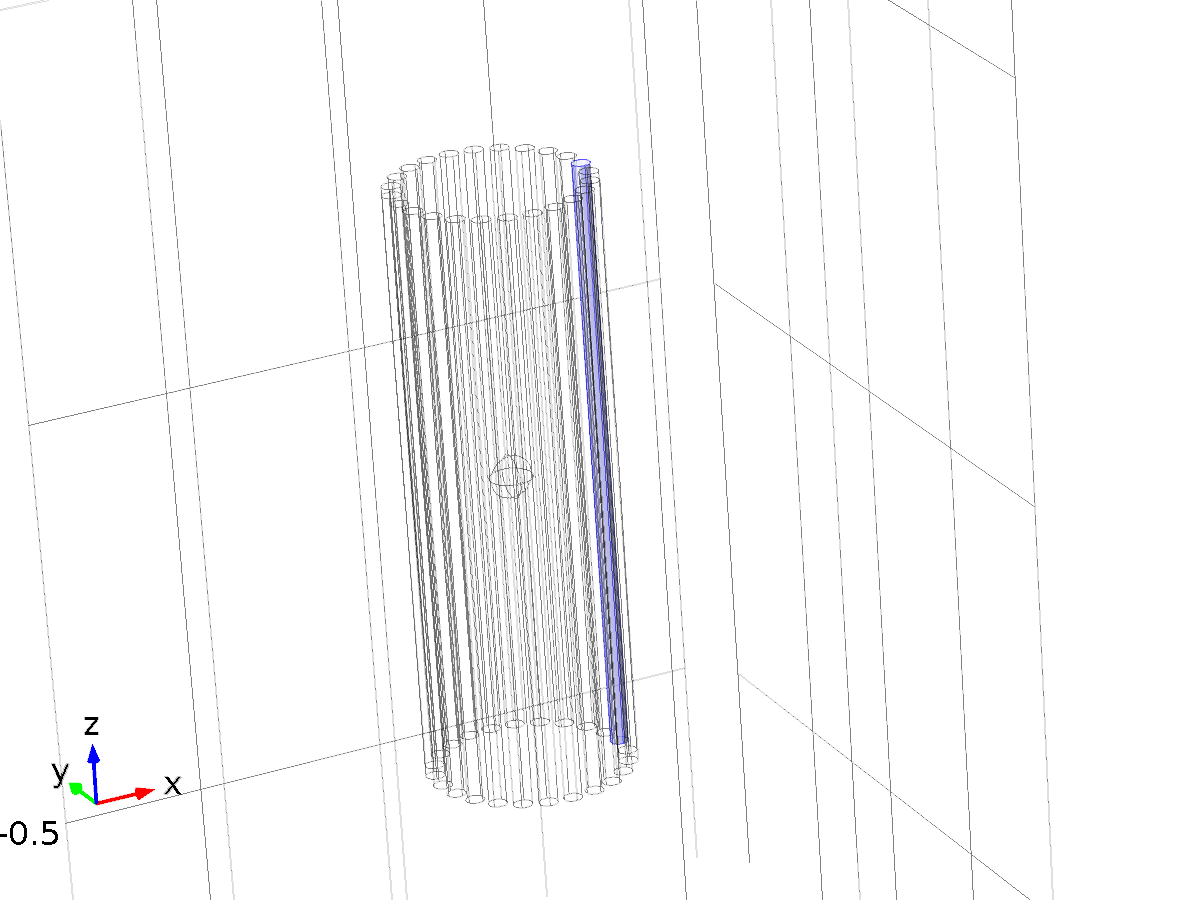

Supplement: S1 File — Model documentation generated by COMSOL with implemented parameters for the SPMA. (ZIP) [file pone.0157040.s001.zip › HalBachArray24CylindricalMag_files/physics_emnc_mfc2.png]

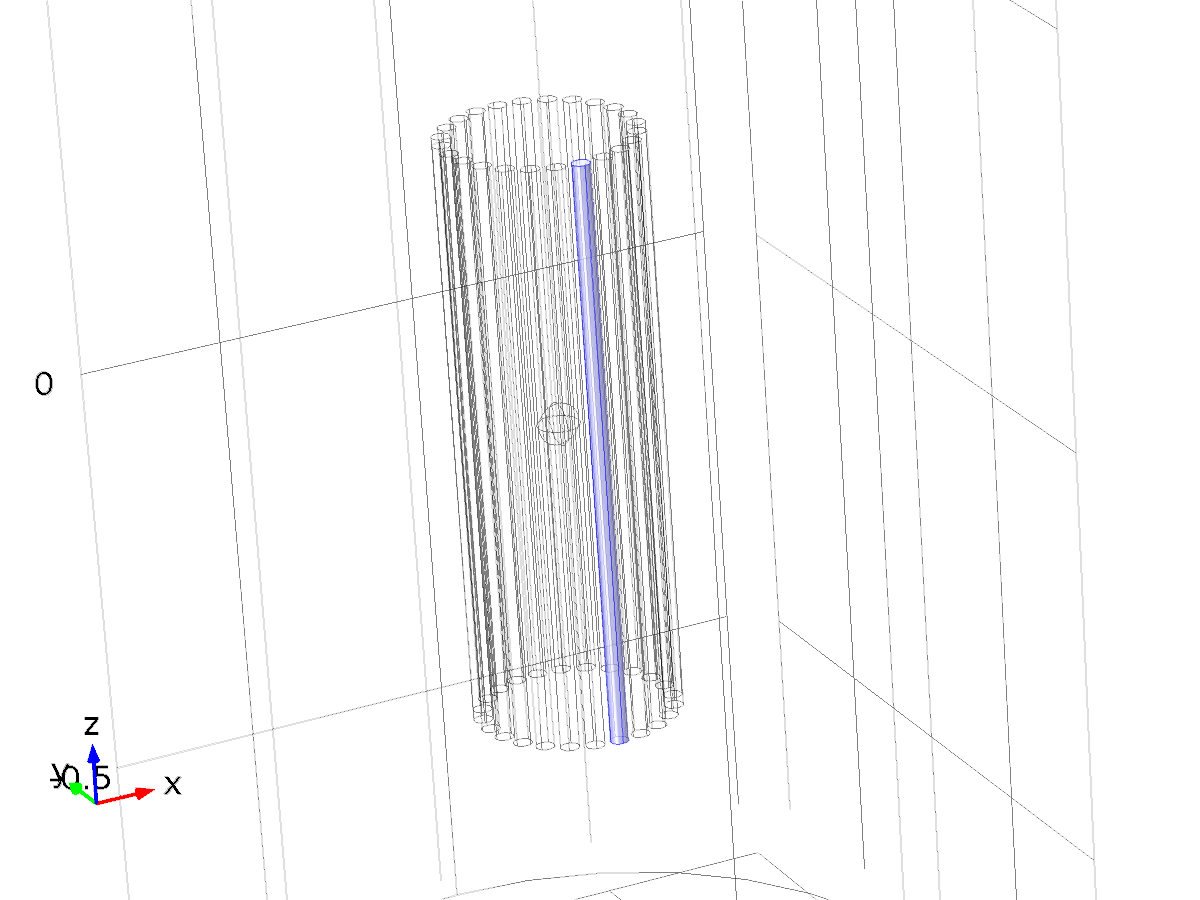

Supplement: S1 File — Model documentation generated by COMSOL with implemented parameters for the SPMA. (ZIP) [file pone.0157040.s001.zip › HalBachArray24CylindricalMag_files/physics_emnc_mfc20.png]

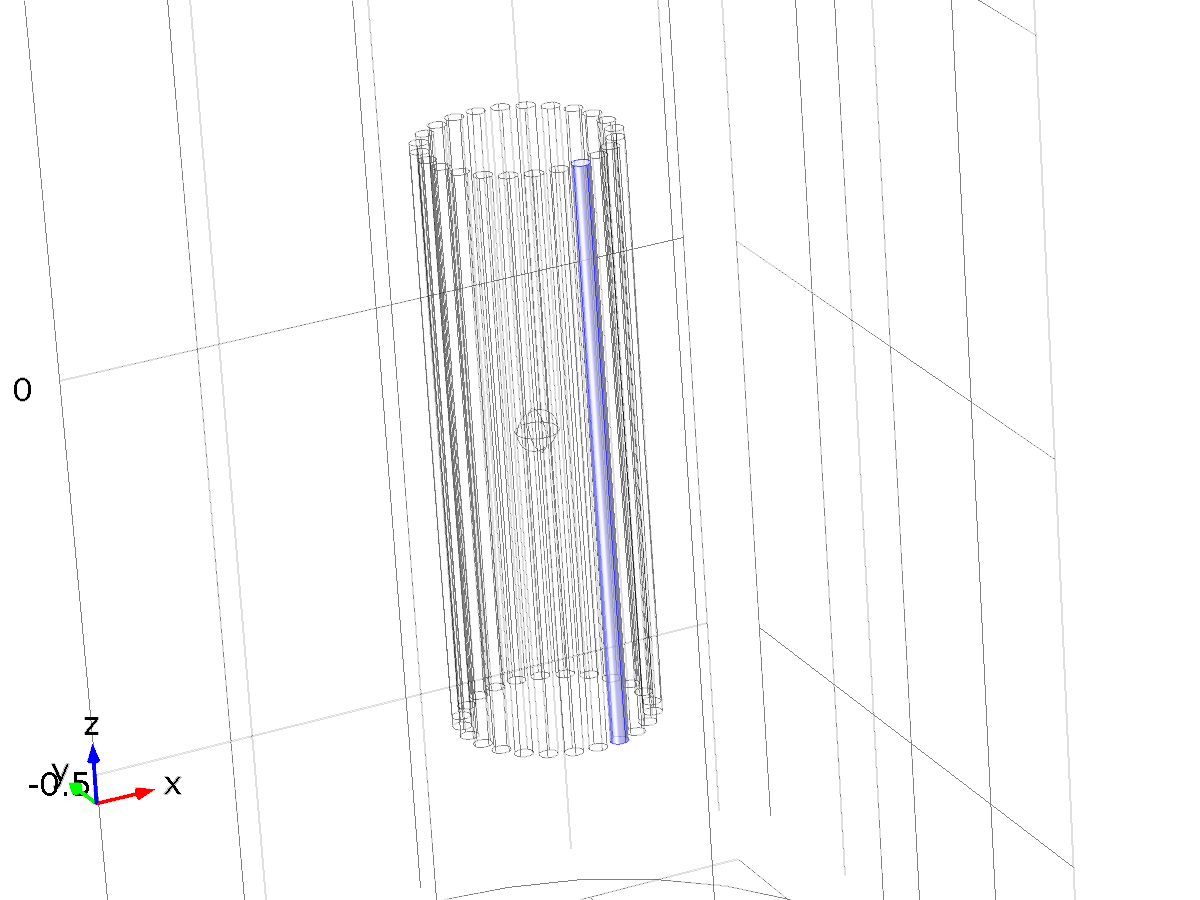

Supplement: S1 File — Model documentation generated by COMSOL with implemented parameters for the SPMA. (ZIP) [file pone.0157040.s001.zip › HalBachArray24CylindricalMag_files/physics_emnc_mfc21.png]

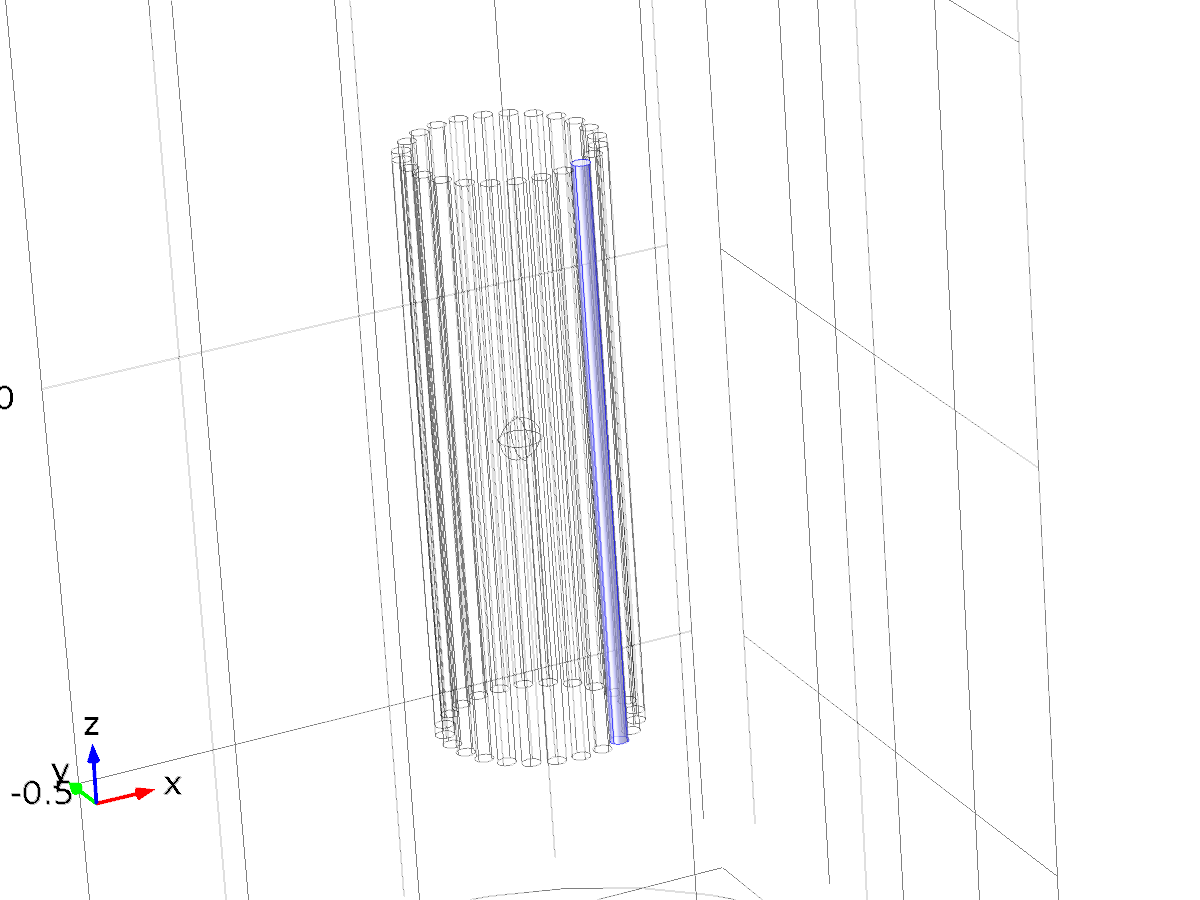

Supplement: S1 File — Model documentation generated by COMSOL with implemented parameters for the SPMA. (ZIP) [file pone.0157040.s001.zip › HalBachArray24CylindricalMag_files/physics_emnc_mfc22.png]

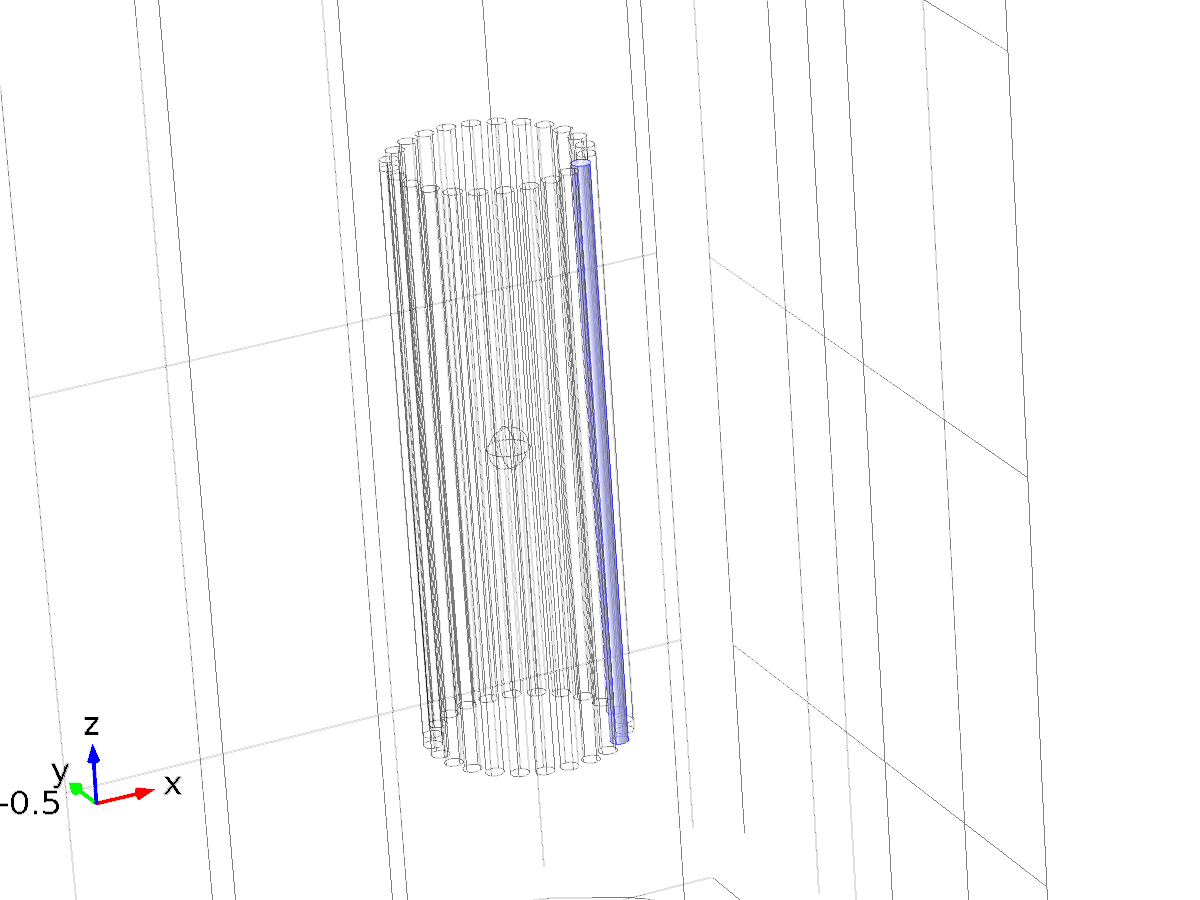

Supplement: S1 File — Model documentation generated by COMSOL with implemented parameters for the SPMA. (ZIP) [file pone.0157040.s001.zip › HalBachArray24CylindricalMag_files/physics_emnc_mfc23.png]

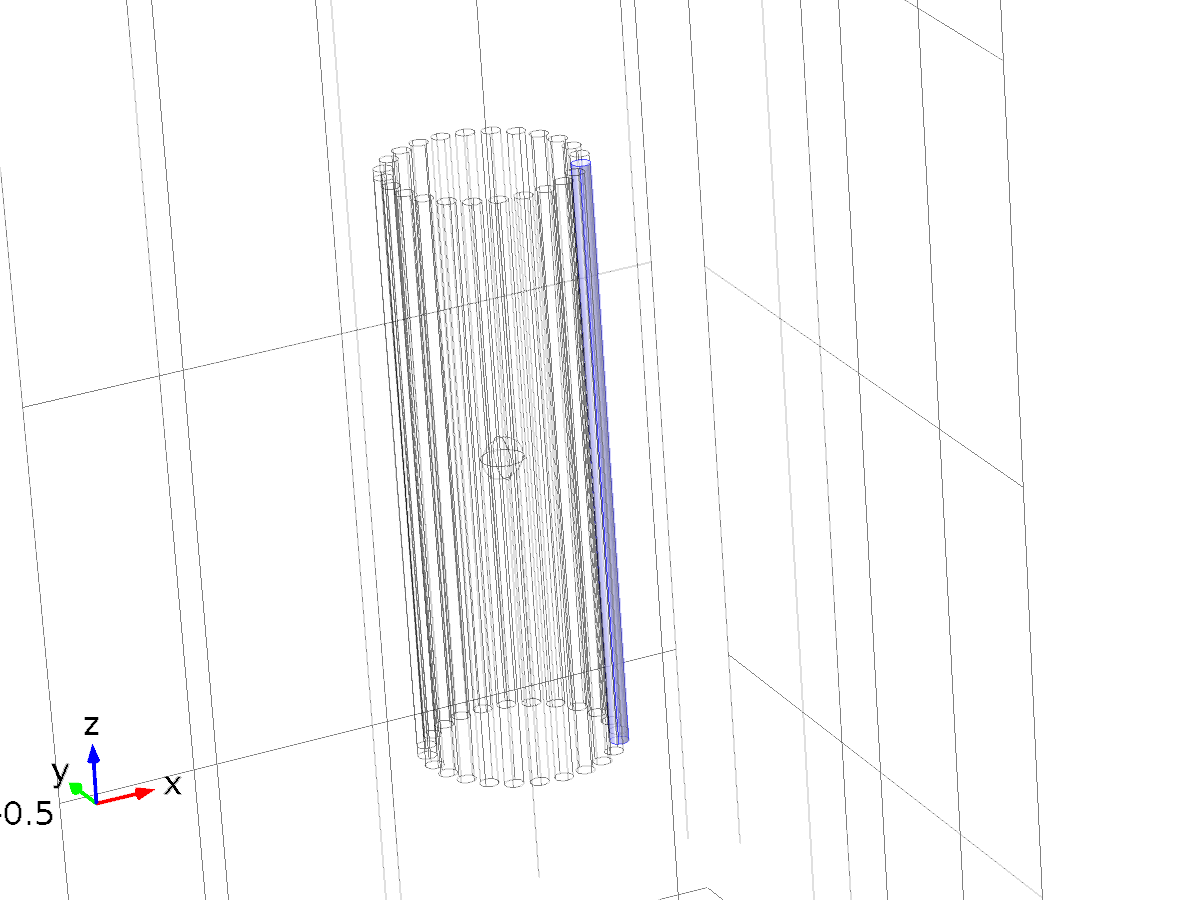

Supplement: S1 File — Model documentation generated by COMSOL with implemented parameters for the SPMA. (ZIP) [file pone.0157040.s001.zip › HalBachArray24CylindricalMag_files/physics_emnc_mfc24.png]

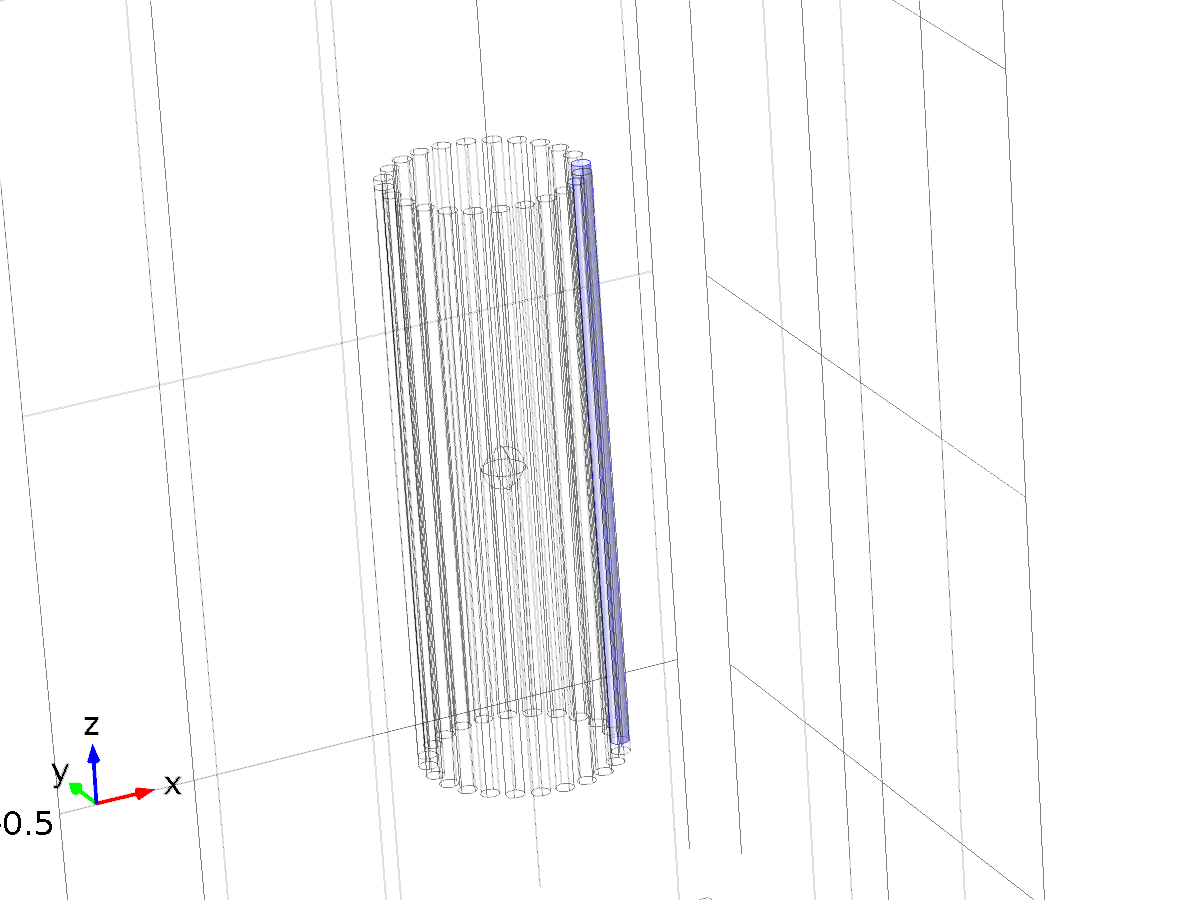

Supplement: S1 File — Model documentation generated by COMSOL with implemented parameters for the SPMA. (ZIP) [file pone.0157040.s001.zip › HalBachArray24CylindricalMag_files/physics_emnc_mfc25.png]

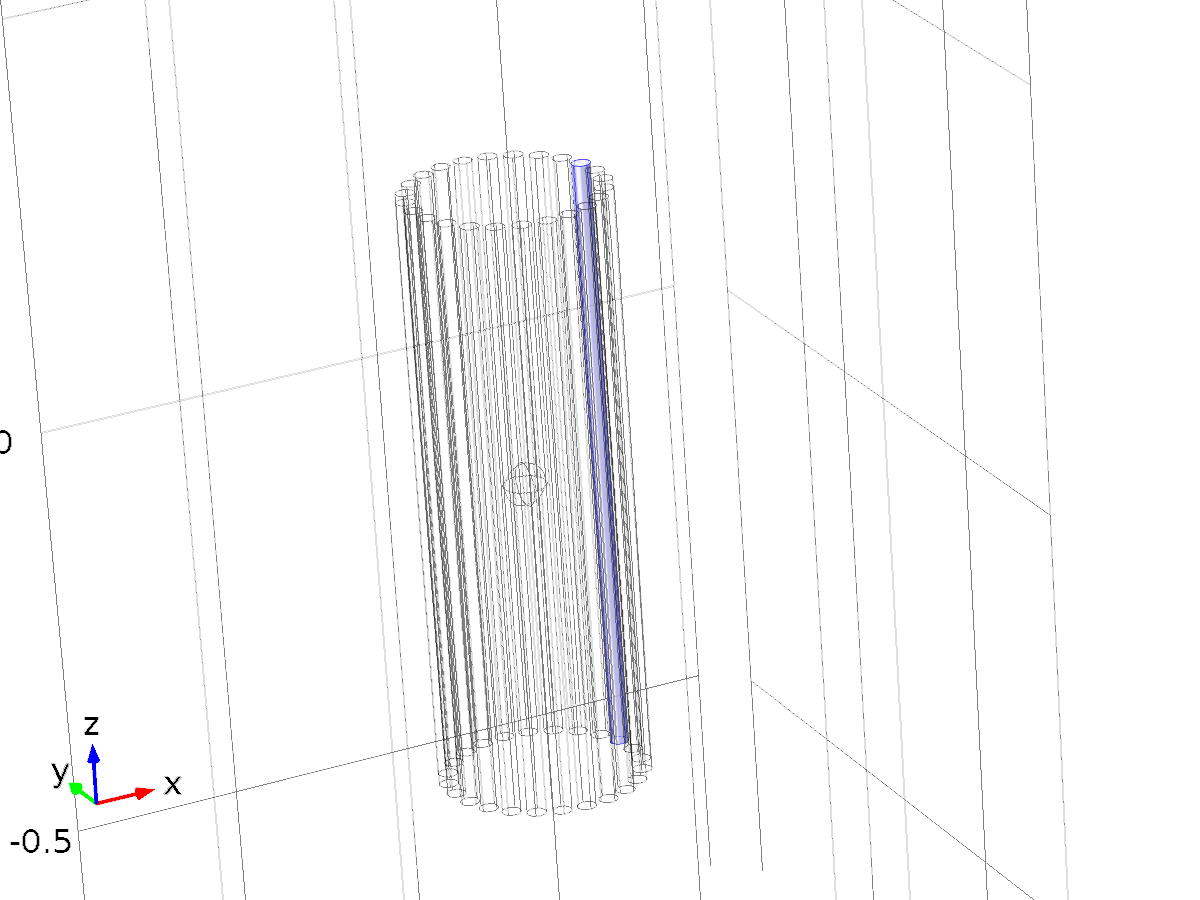

Supplement: S1 File — Model documentation generated by COMSOL with implemented parameters for the SPMA. (ZIP) [file pone.0157040.s001.zip › HalBachArray24CylindricalMag_files/physics_emnc_mfc3.png]

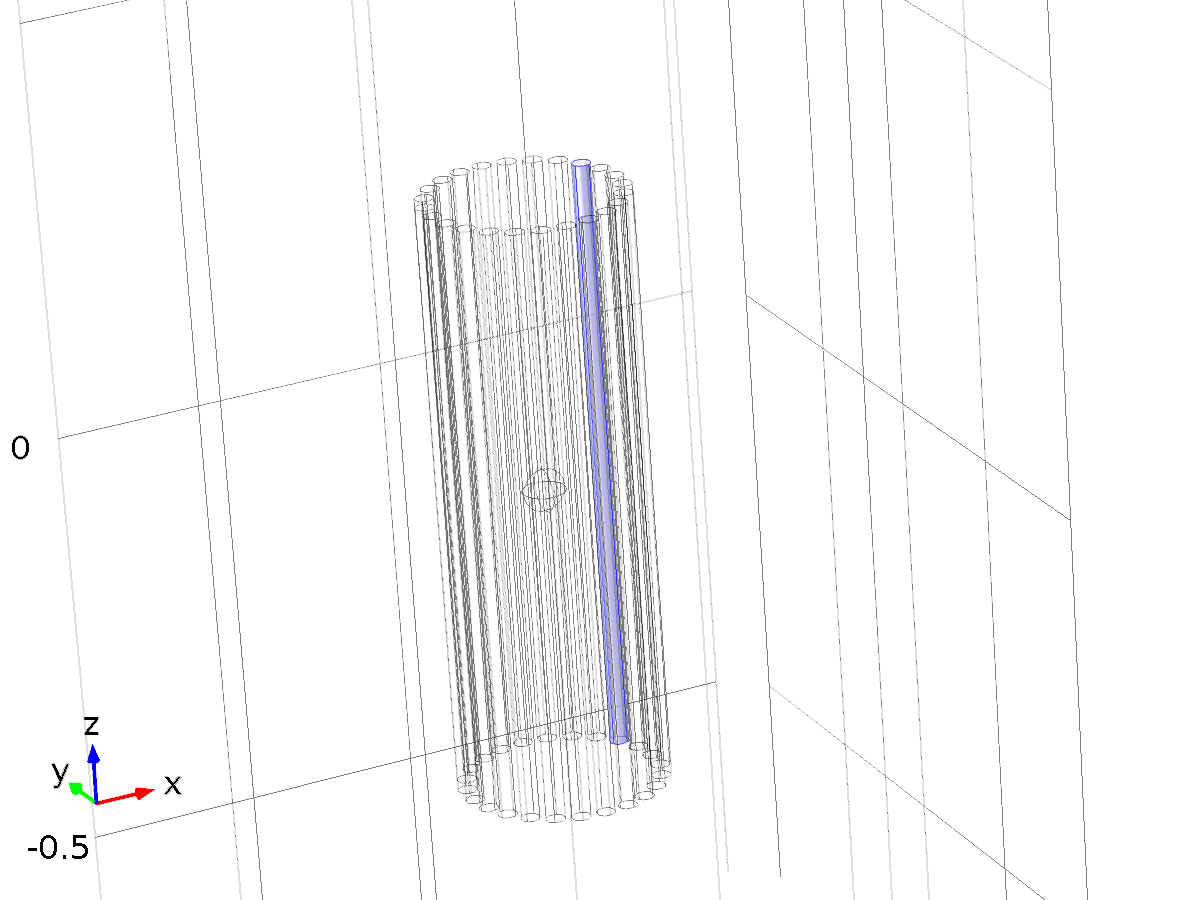

Supplement: S1 File — Model documentation generated by COMSOL with implemented parameters for the SPMA. (ZIP) [file pone.0157040.s001.zip › HalBachArray24CylindricalMag_files/physics_emnc_mfc4.png]

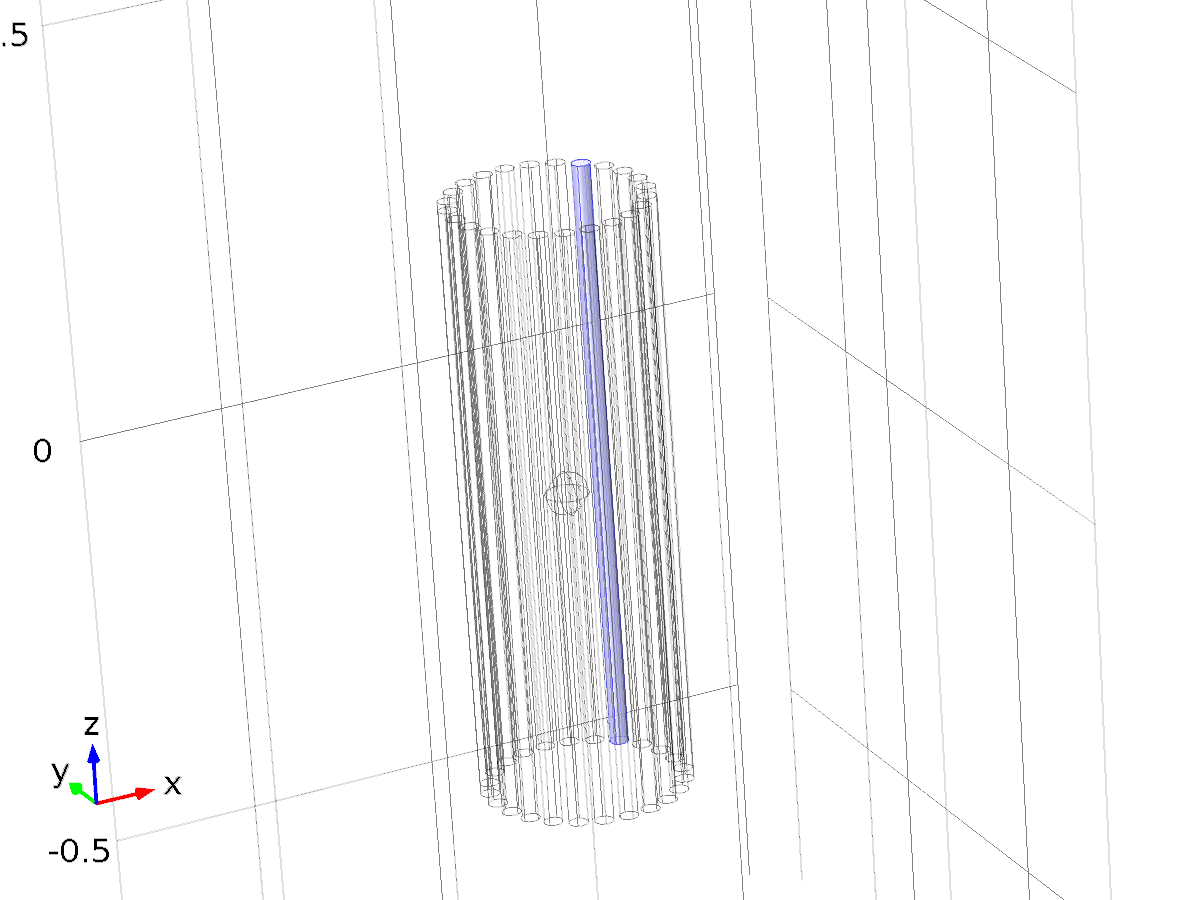

Supplement: S1 File — Model documentation generated by COMSOL with implemented parameters for the SPMA. (ZIP) [file pone.0157040.s001.zip › HalBachArray24CylindricalMag_files/physics_emnc_mfc5.png]

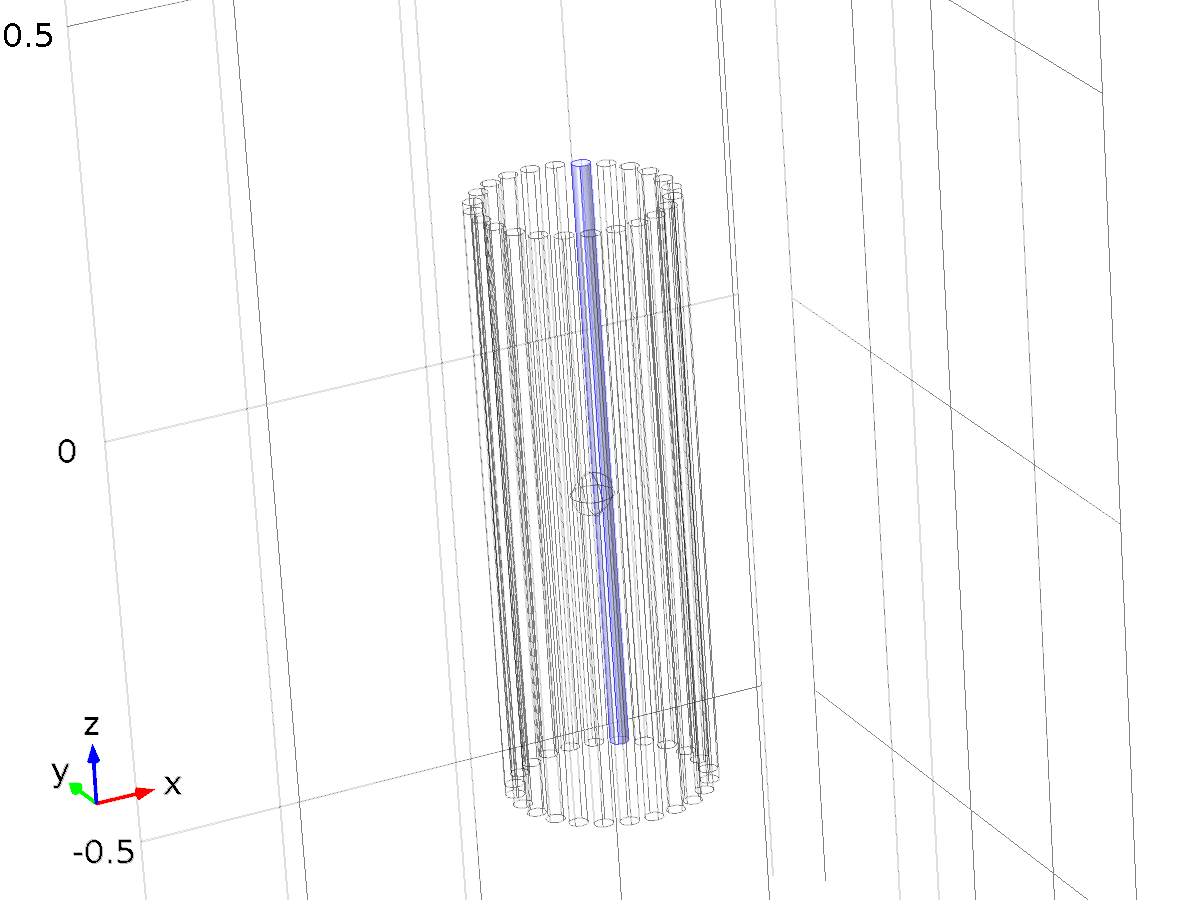

Supplement: S1 File — Model documentation generated by COMSOL with implemented parameters for the SPMA. (ZIP) [file pone.0157040.s001.zip › HalBachArray24CylindricalMag_files/physics_emnc_mfc6.png]

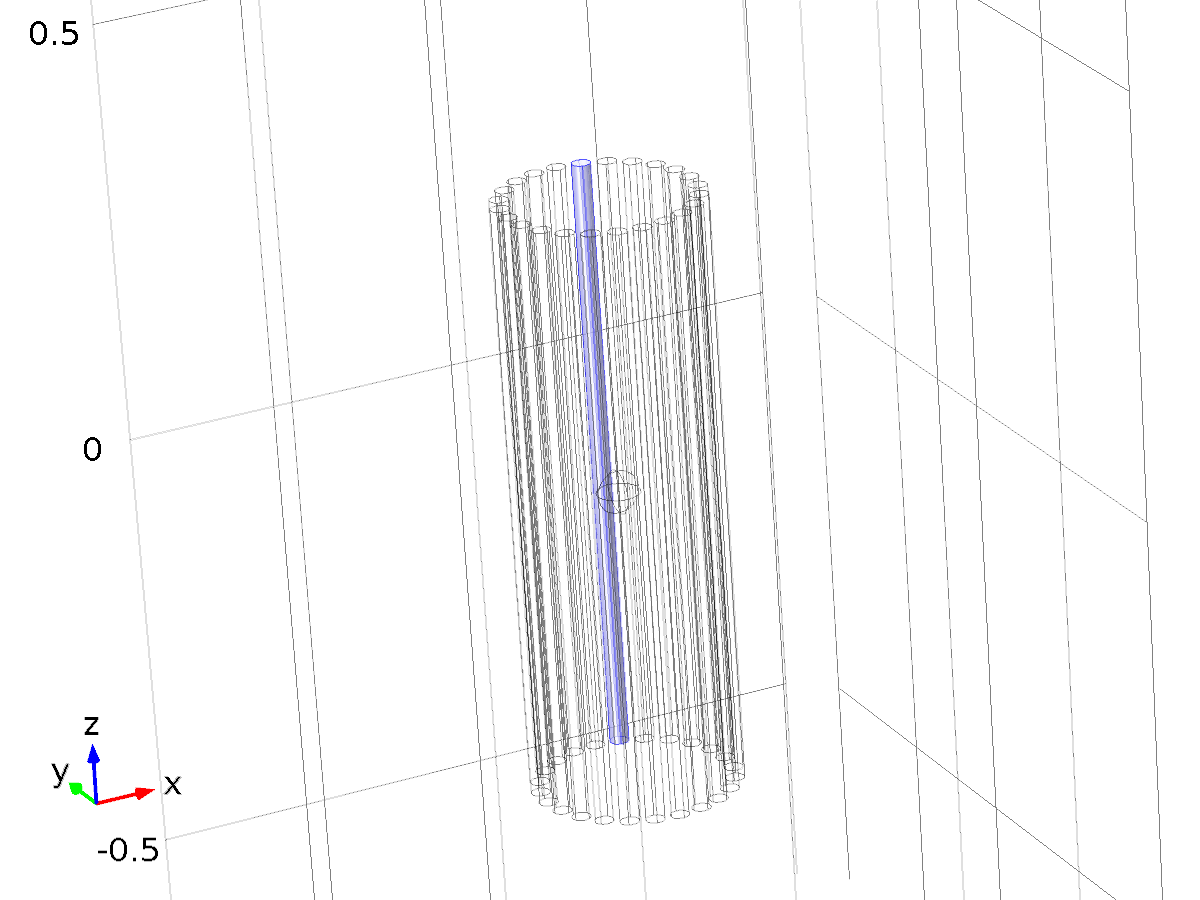

Supplement: S1 File — Model documentation generated by COMSOL with implemented parameters for the SPMA. (ZIP) [file pone.0157040.s001.zip › HalBachArray24CylindricalMag_files/physics_emnc_mfc7.png]

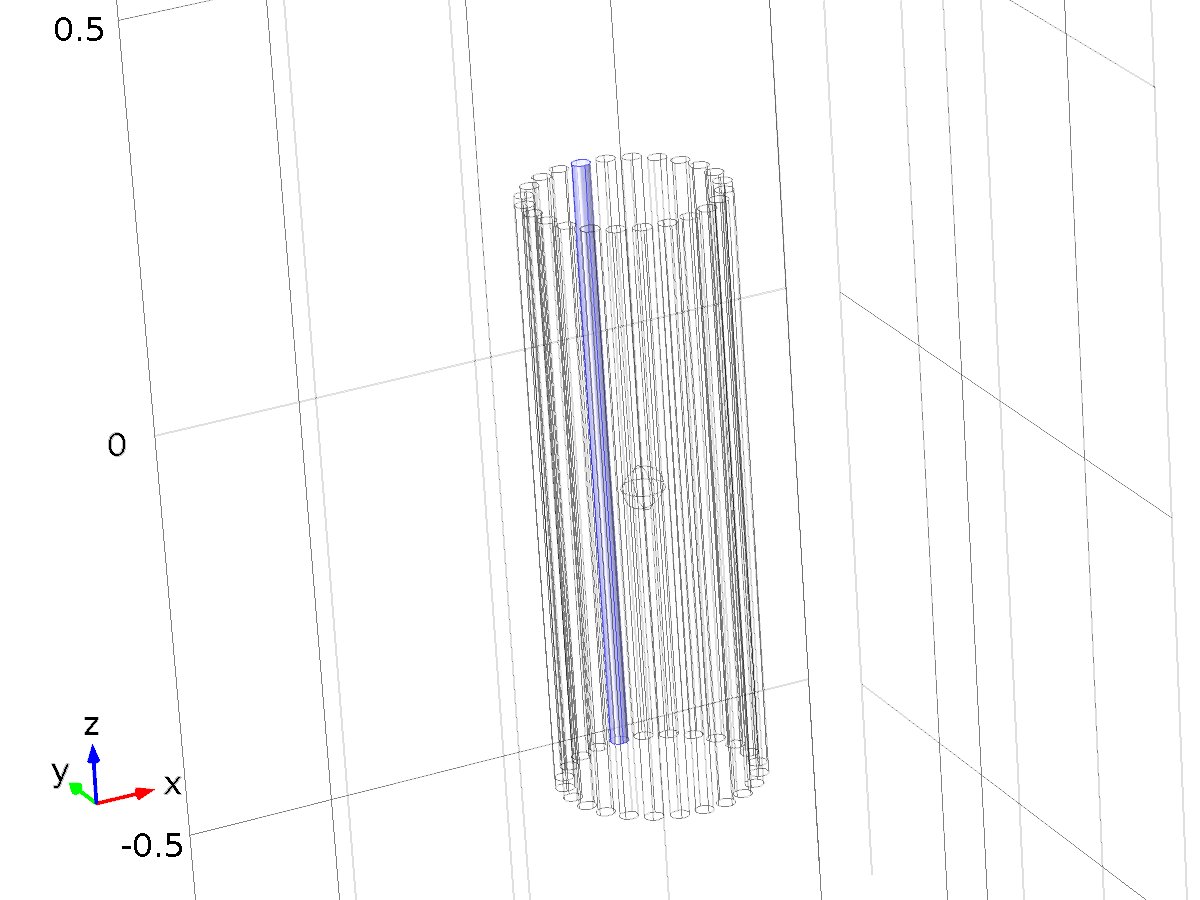

Supplement: S1 File — Model documentation generated by COMSOL with implemented parameters for the SPMA. (ZIP) [file pone.0157040.s001.zip › HalBachArray24CylindricalMag_files/physics_emnc_mfc8.png]

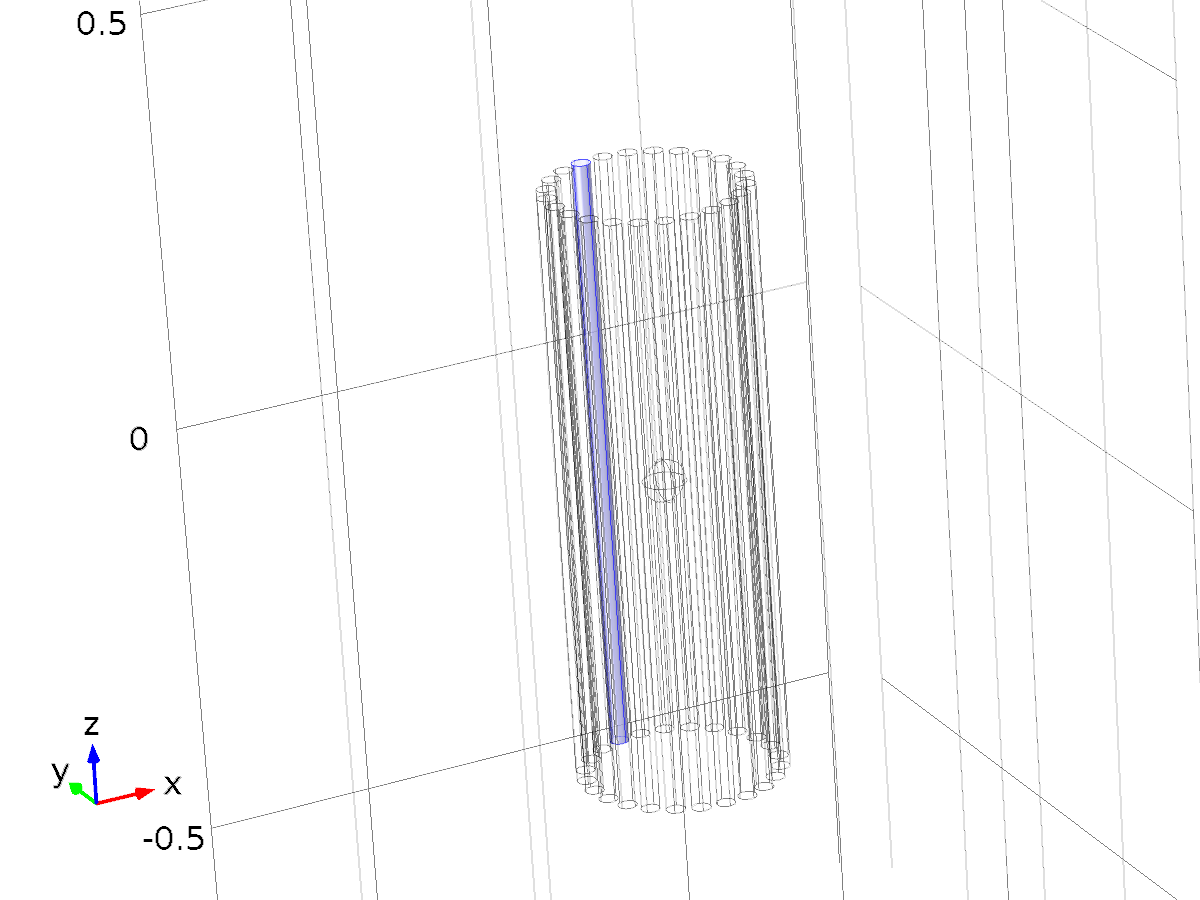

Supplement: S1 File — Model documentation generated by COMSOL with implemented parameters for the SPMA. (ZIP) [file pone.0157040.s001.zip › HalBachArray24CylindricalMag_files/physics_emnc_mfc9.png]

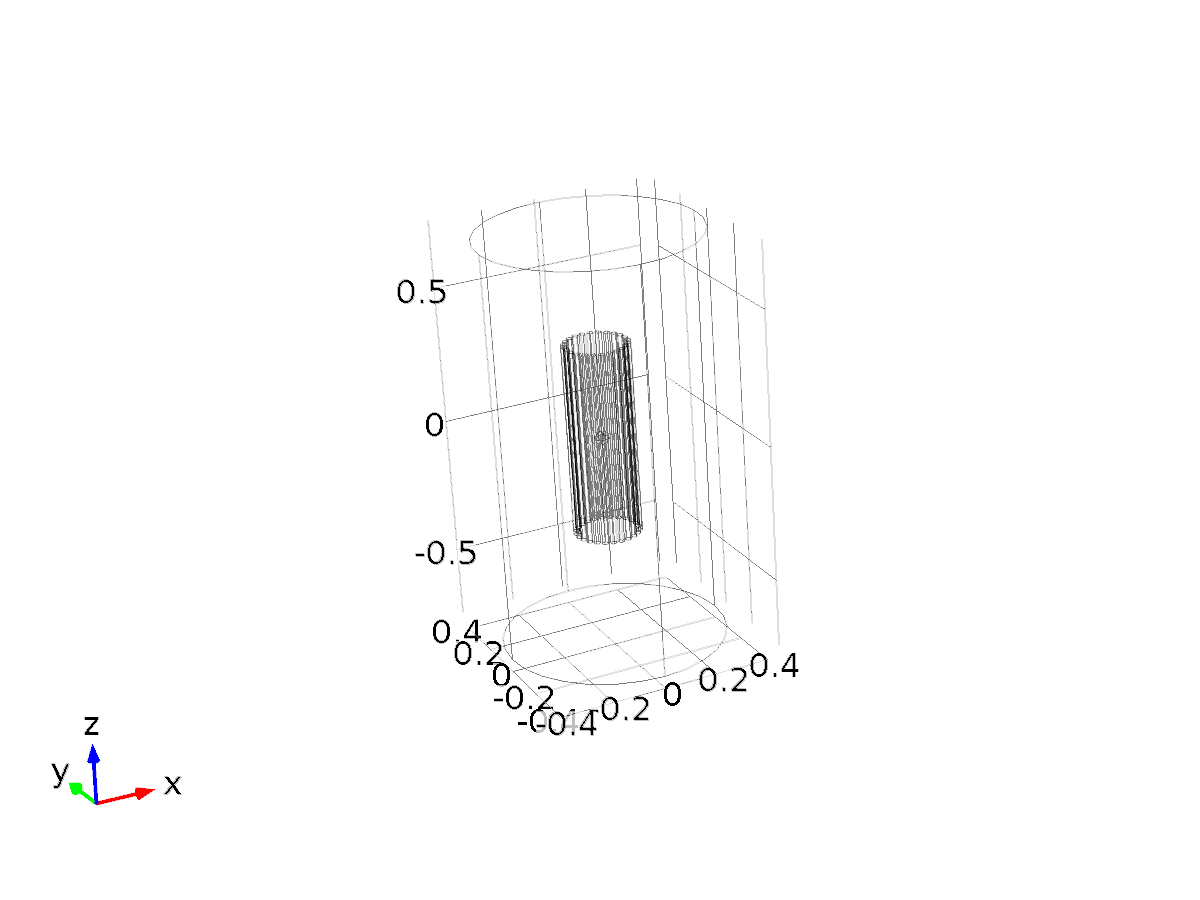

Supplement: S1 File — Model documentation generated by COMSOL with implemented parameters for the SPMA. (ZIP) [file pone.0157040.s001.zip › HalBachArray24CylindricalMag_files/physics_emnc_mi1.png]

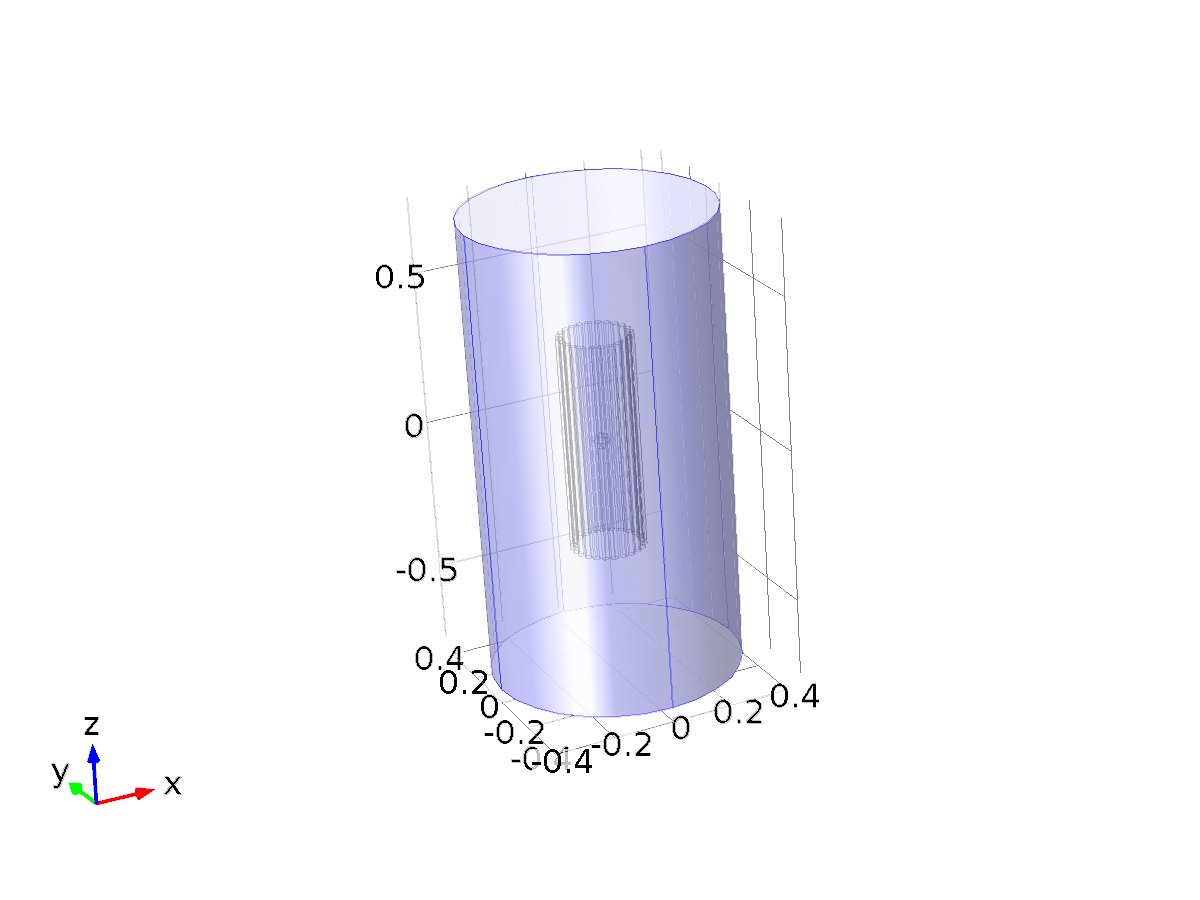

Supplement: S1 File — Model documentation generated by COMSOL with implemented parameters for the SPMA. (ZIP) [file pone.0157040.s001.zip › HalBachArray24CylindricalMag_files/physics_emnc_ms1.png]

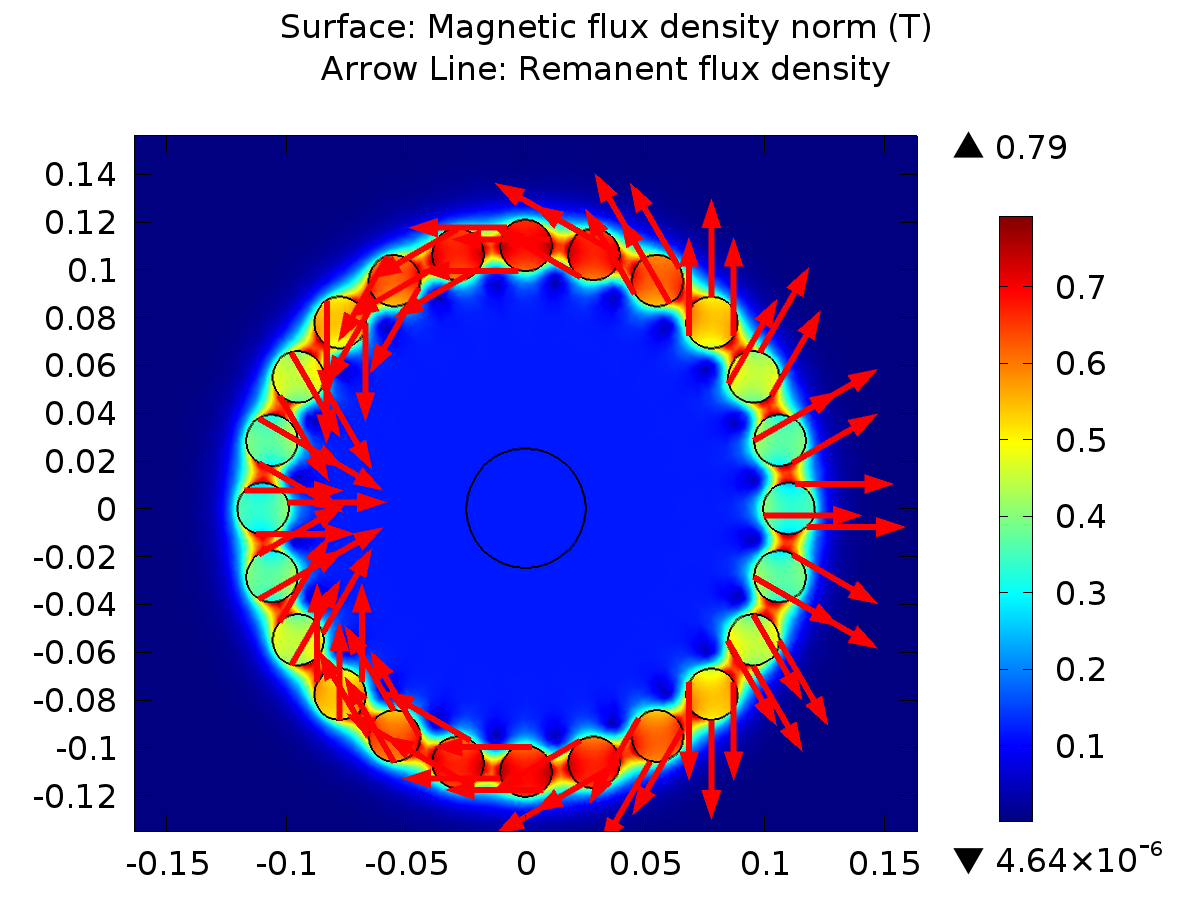

Supplement: S1 File — Model documentation generated by COMSOL with implemented parameters for the SPMA. (ZIP) [file pone.0157040.s001.zip › HalBachArray24CylindricalMag_files/plotgroup_pg1.png]

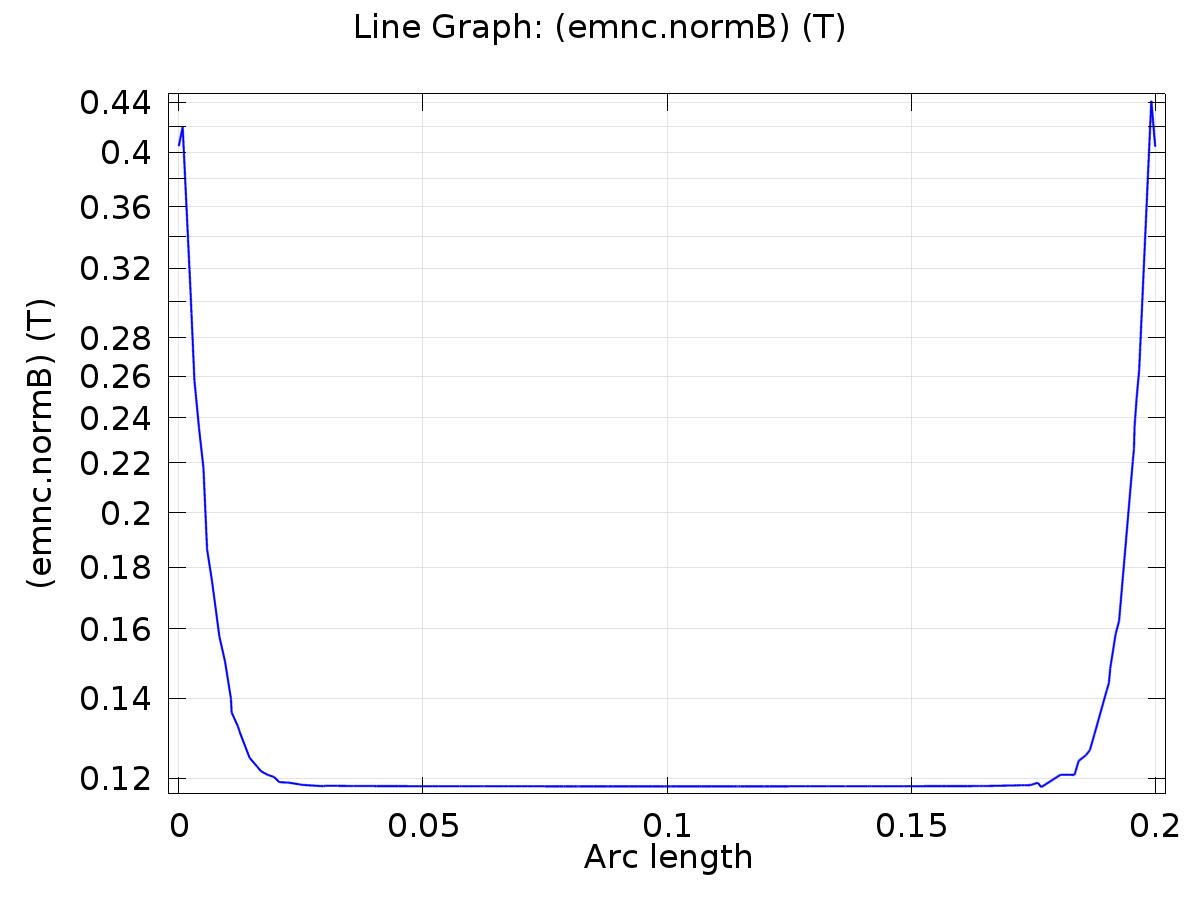

Supplement: S1 File — Model documentation generated by COMSOL with implemented parameters for the SPMA. (ZIP) [file pone.0157040.s001.zip › HalBachArray24CylindricalMag_files/plotgroup_pg2.png]

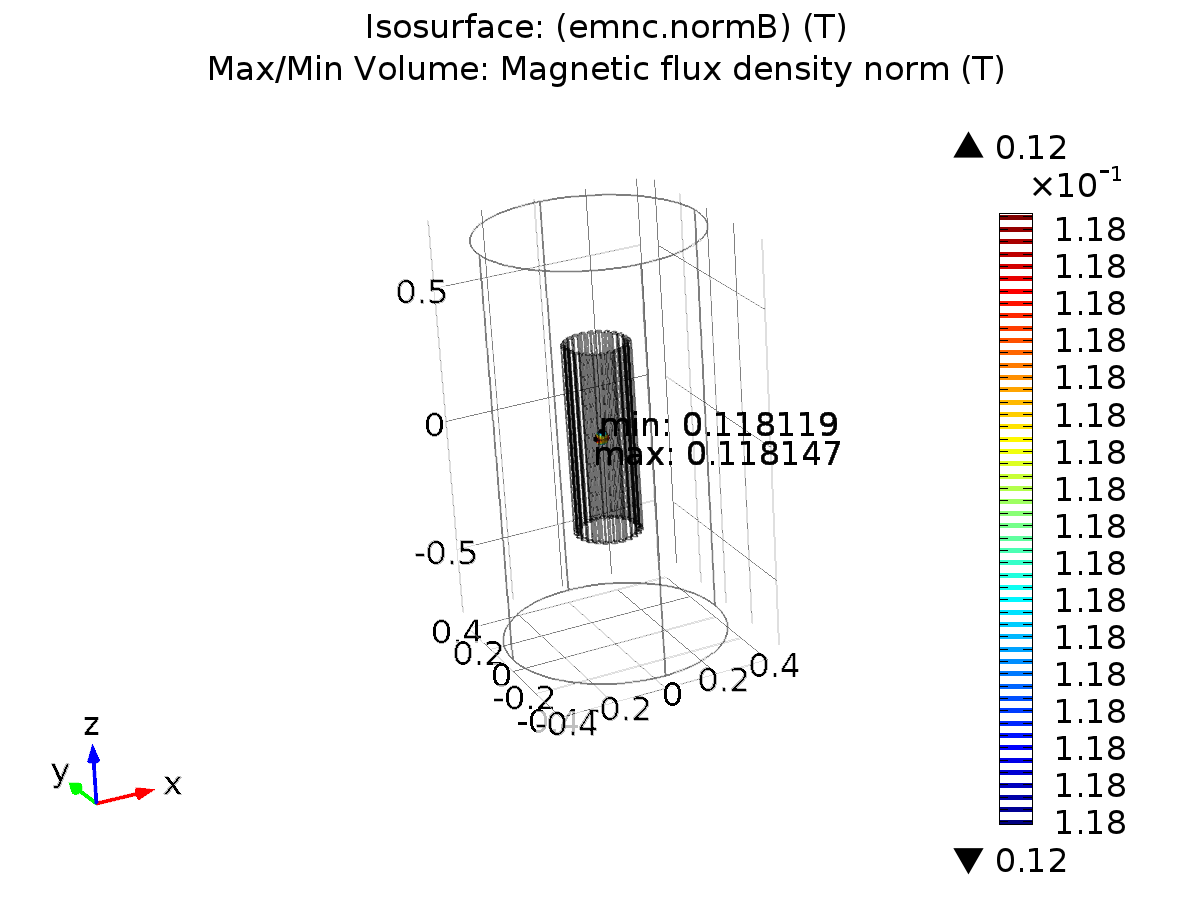

Supplement: S1 File — Model documentation generated by COMSOL with implemented parameters for the SPMA. (ZIP) [file pone.0157040.s001.zip › HalBachArray24CylindricalMag_files/plotgroup_pg3.png]

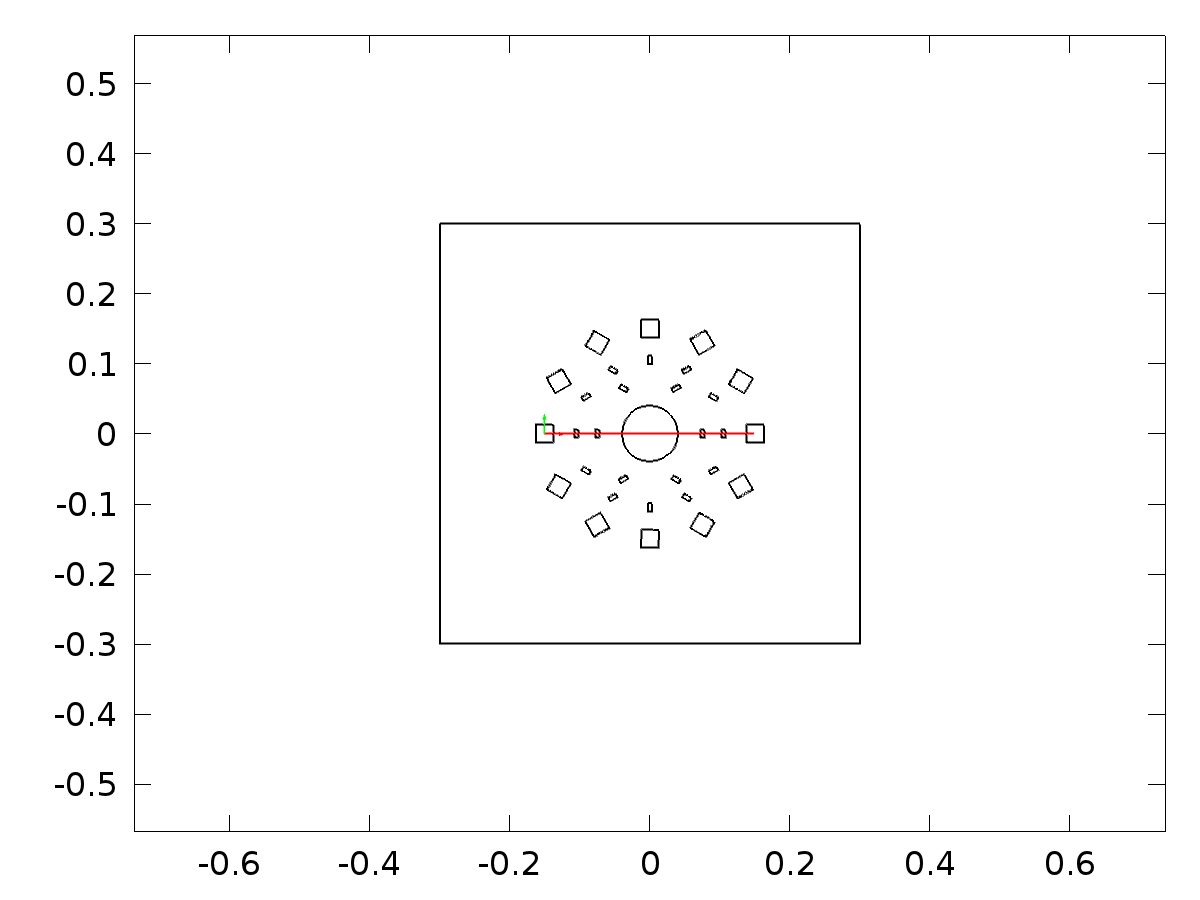

Supplement: S2 File — Model documentation generated by COMSOL with implemented parameters for the manual prototype. (ZIP) [file pone.0157040.s002.zip › SPMA_Rectangle_files/dataset_cln1.png]

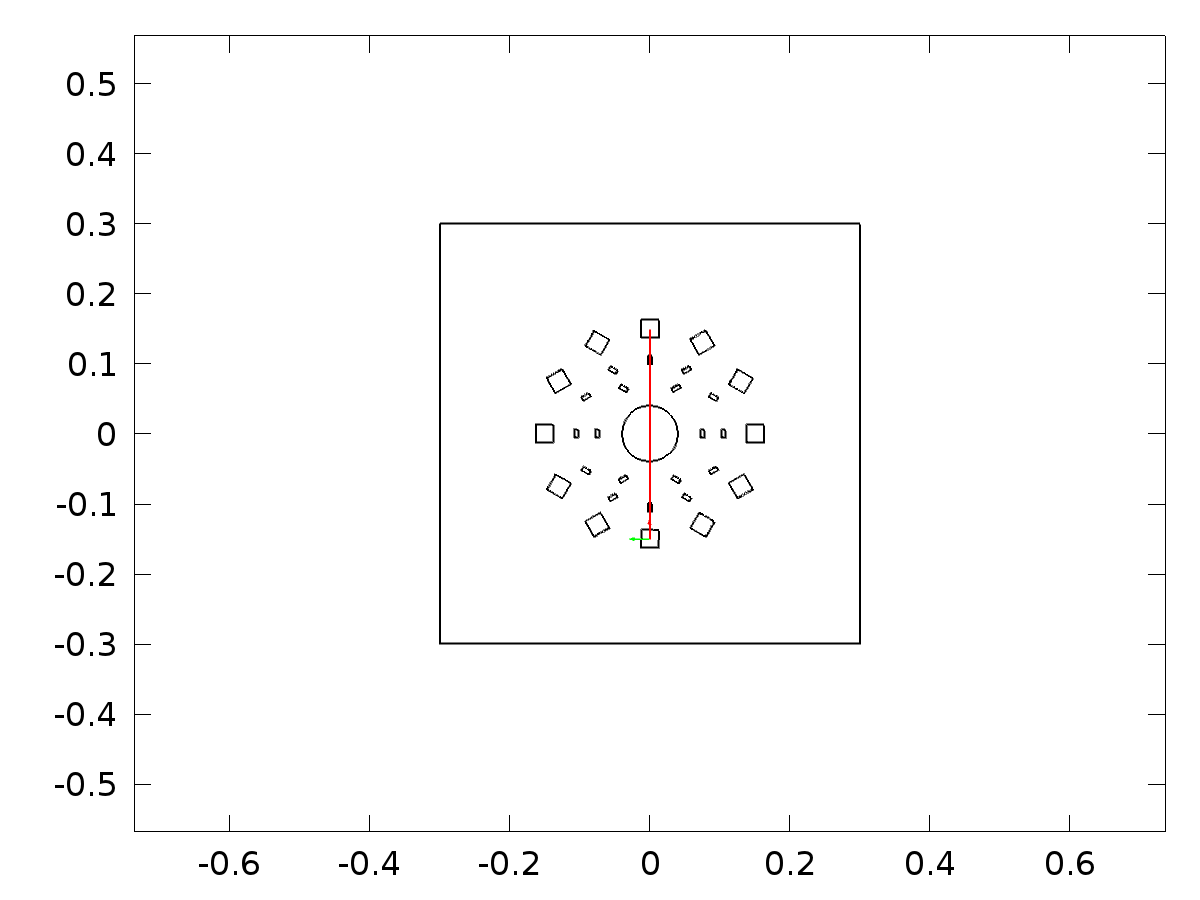

Supplement: S2 File — Model documentation generated by COMSOL with implemented parameters for the manual prototype. (ZIP) [file pone.0157040.s002.zip › SPMA_Rectangle_files/dataset_cln2.png]

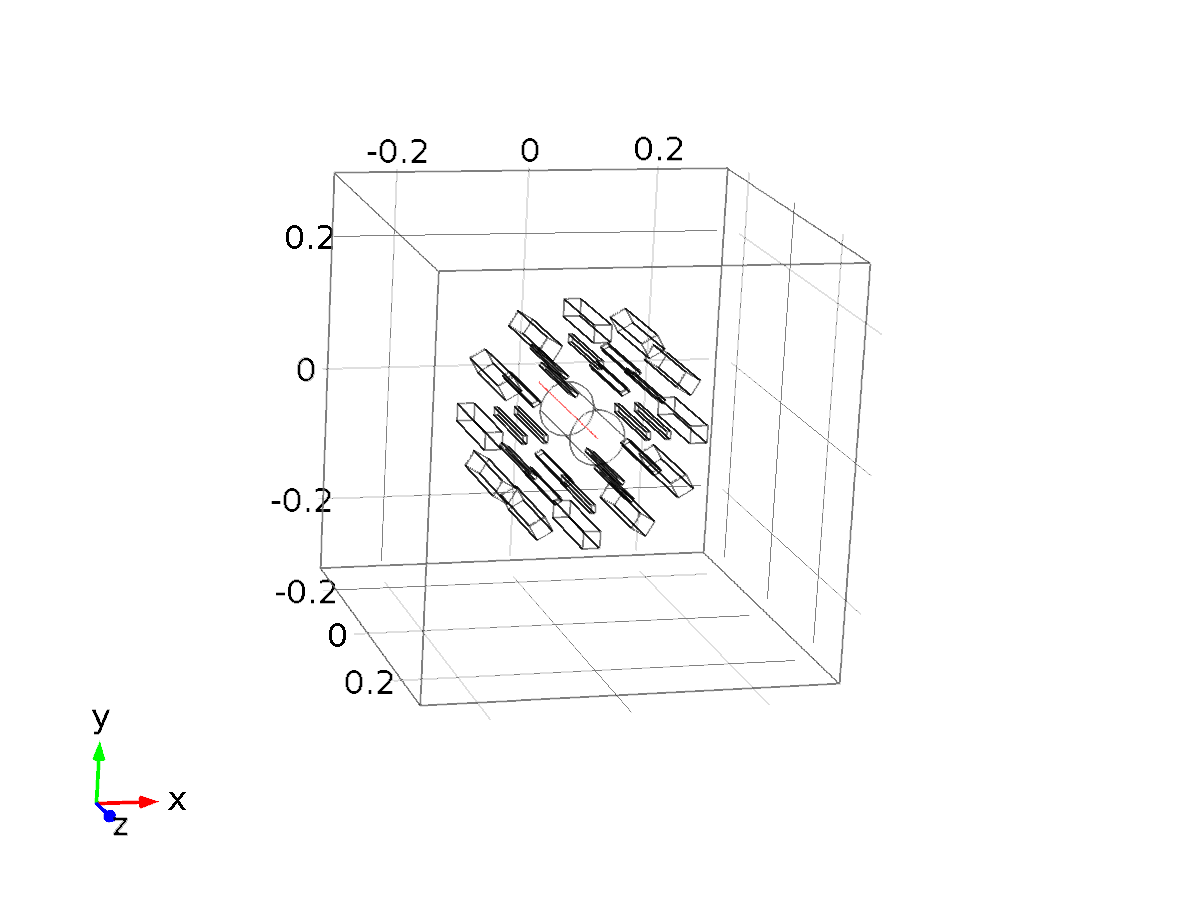

Supplement: S2 File — Model documentation generated by COMSOL with implemented parameters for the manual prototype. (ZIP) [file pone.0157040.s002.zip › SPMA_Rectangle_files/dataset_cln3.png]

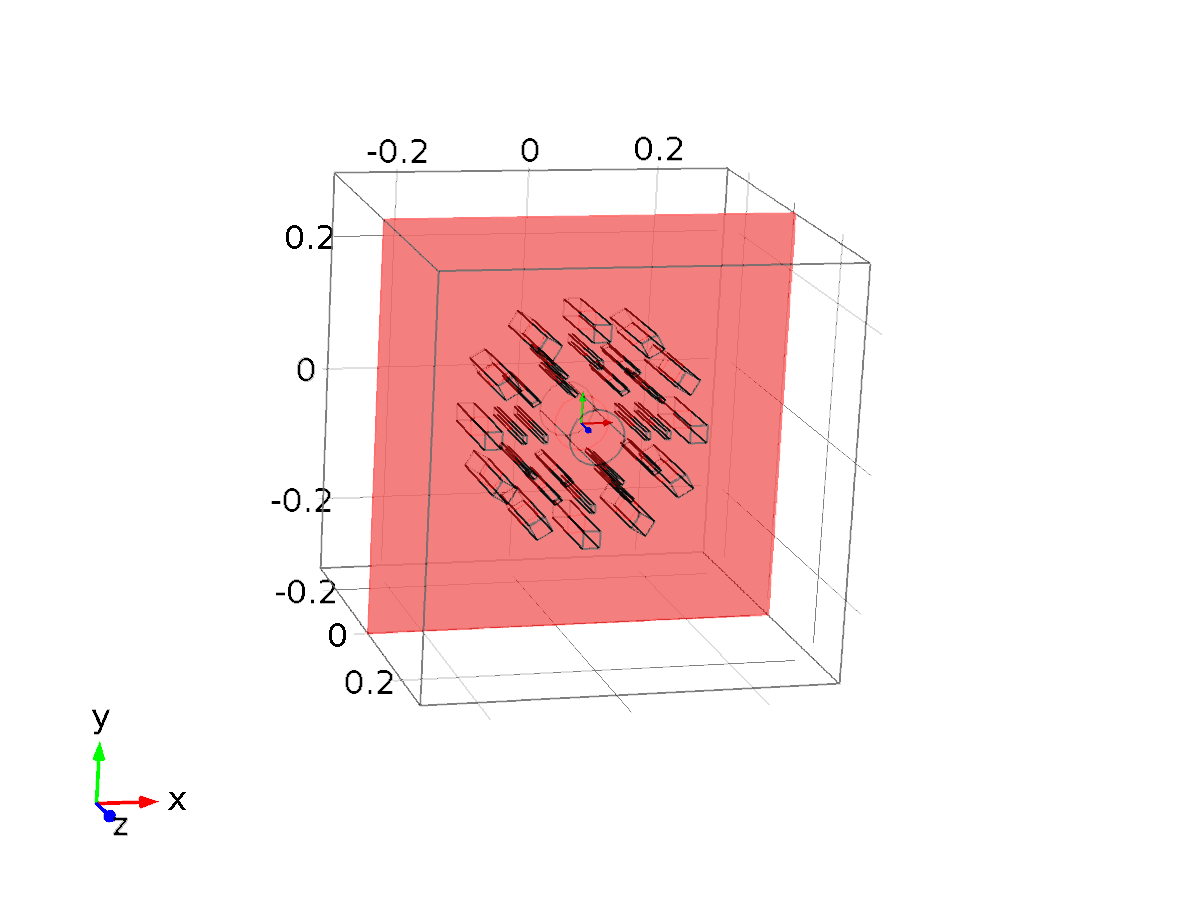

Supplement: S2 File — Model documentation generated by COMSOL with implemented parameters for the manual prototype. (ZIP) [file pone.0157040.s002.zip › SPMA_Rectangle_files/dataset_cpl1.png]

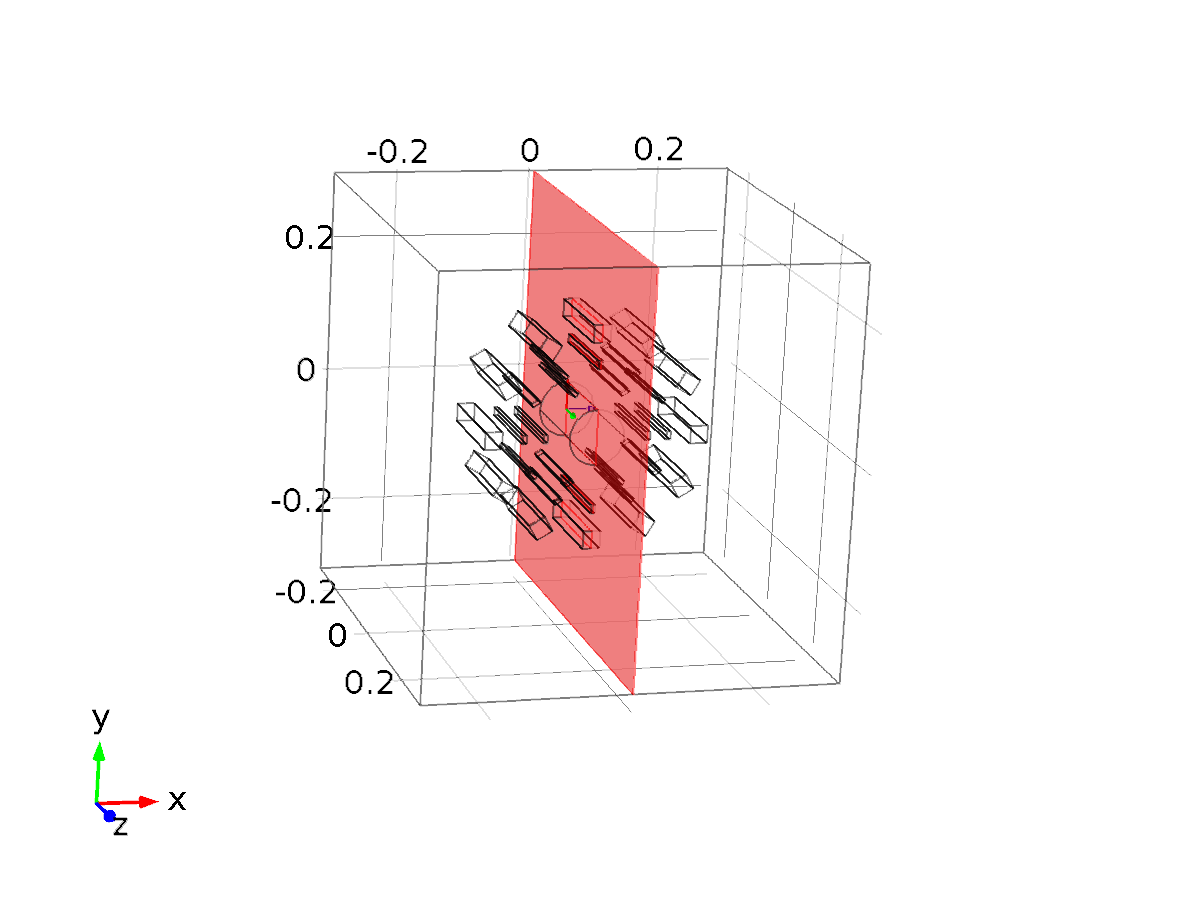

Supplement: S2 File — Model documentation generated by COMSOL with implemented parameters for the manual prototype. (ZIP) [file pone.0157040.s002.zip › SPMA_Rectangle_files/dataset_cpl2.png]

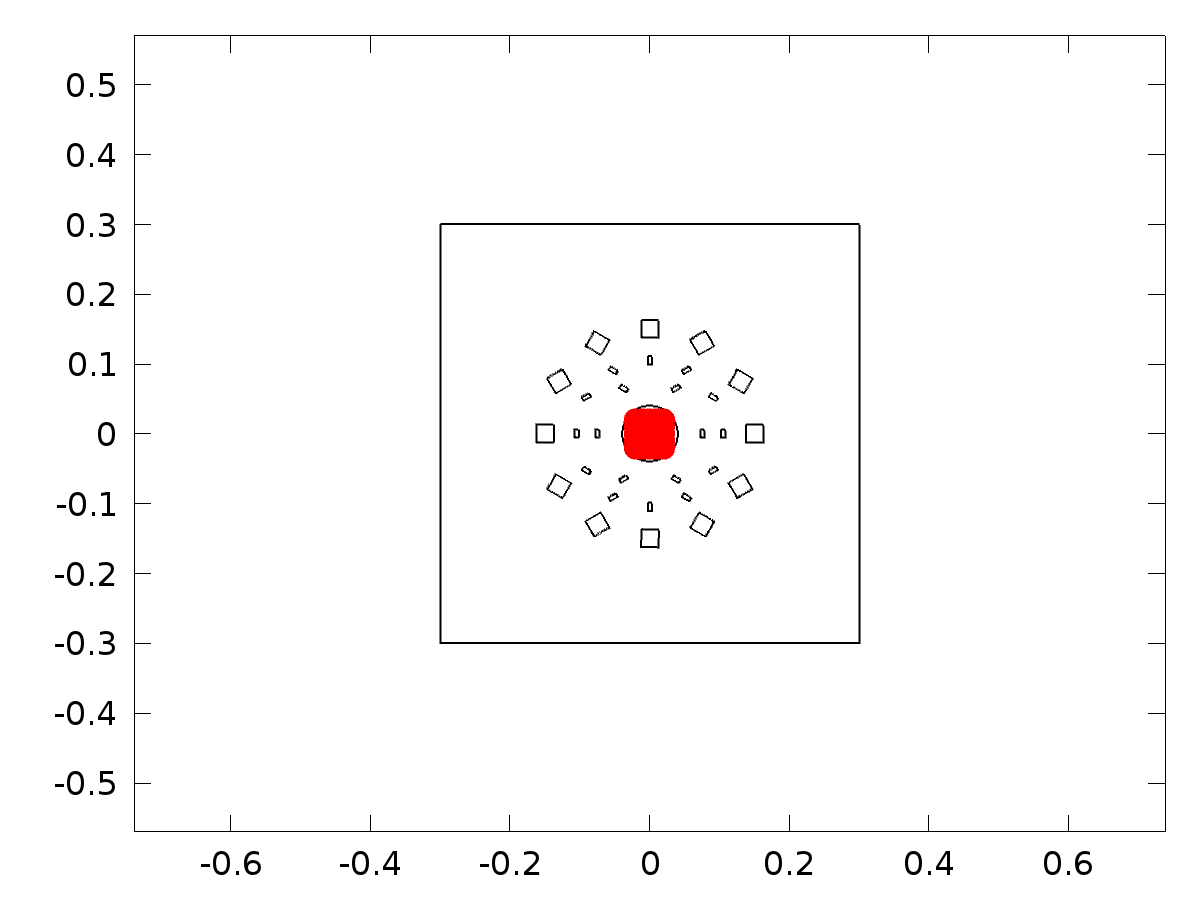

Supplement: S2 File — Model documentation generated by COMSOL with implemented parameters for the manual prototype. (ZIP) [file pone.0157040.s002.zip › SPMA_Rectangle_files/dataset_cpt1.png]

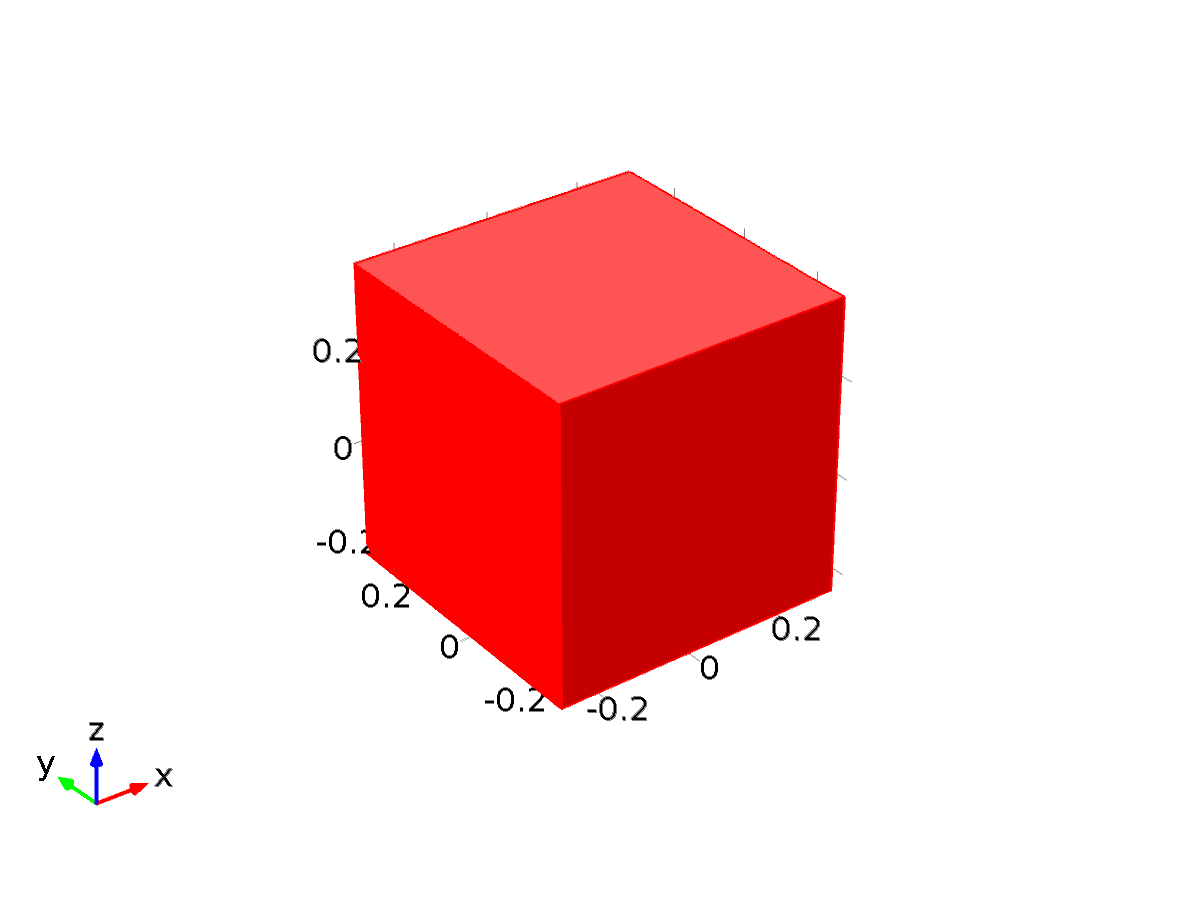

Supplement: S2 File — Model documentation generated by COMSOL with implemented parameters for the manual prototype. (ZIP) [file pone.0157040.s002.zip › SPMA_Rectangle_files/dataset_dset1.png]

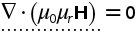

Supplement: S2 File — Model documentation generated by COMSOL with implemented parameters for the manual prototype. (ZIP) [file pone.0157040.s002.zip › SPMA_Rectangle_files/equ_mfnc_mfc1_2.png]

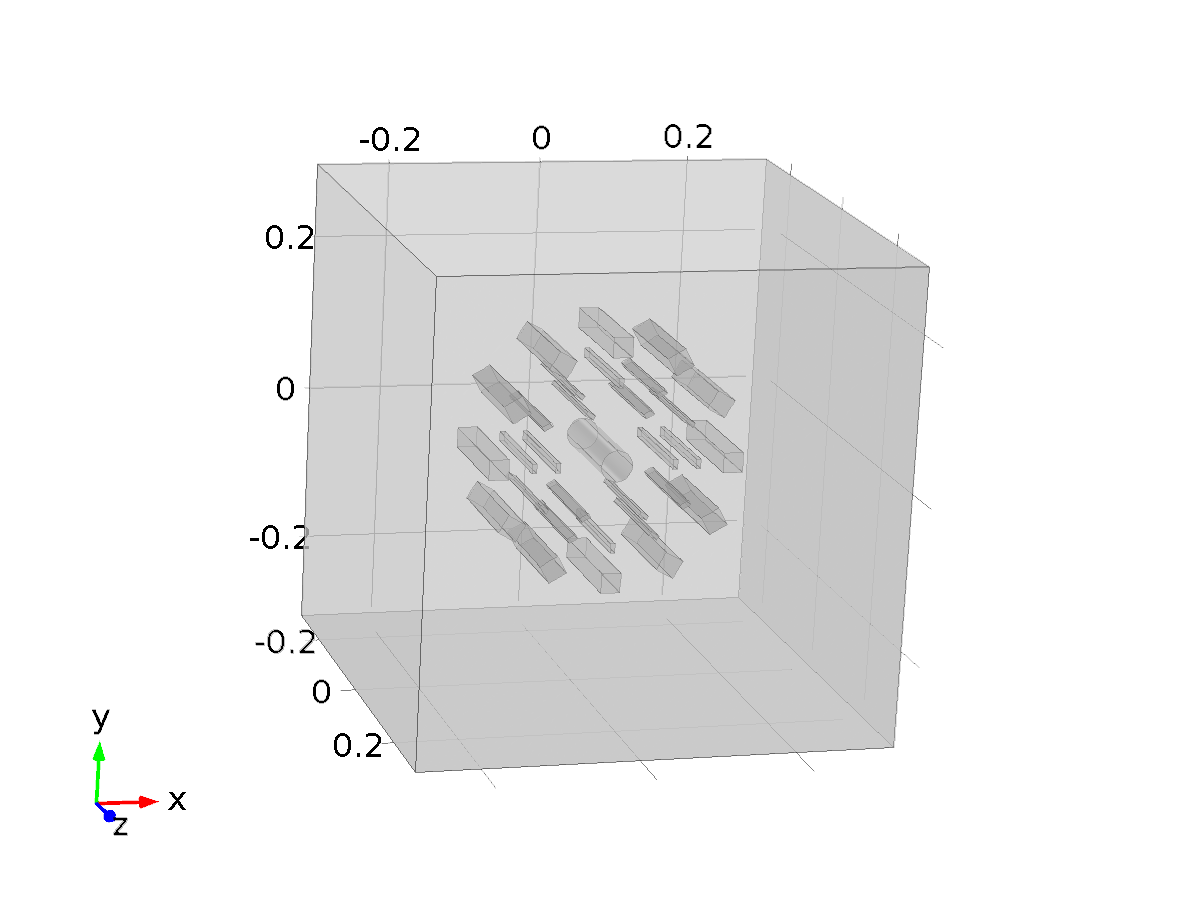

Supplement: S2 File — Model documentation generated by COMSOL with implemented parameters for the manual prototype. (ZIP) [file pone.0157040.s002.zip › SPMA_Rectangle_files/geom_geom1.png]

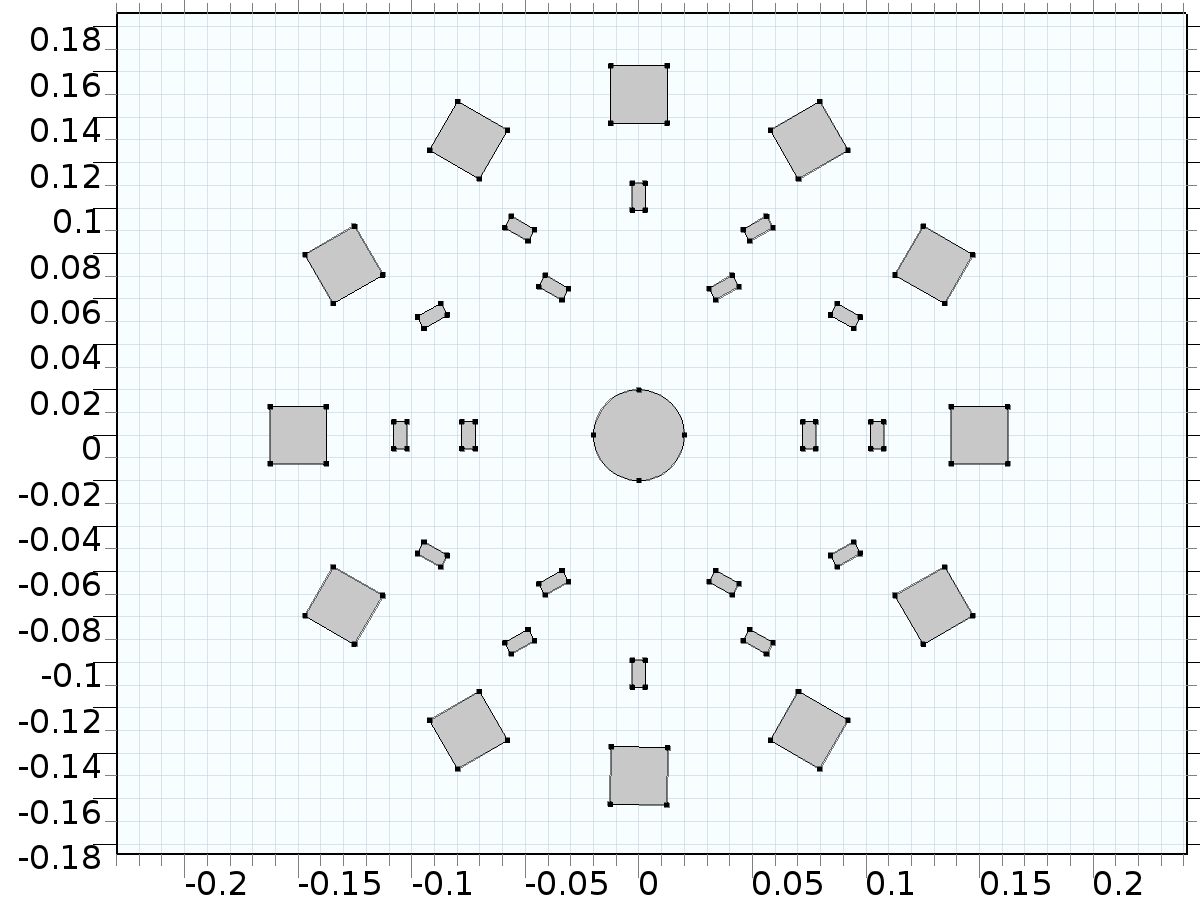

Supplement: S2 File — Model documentation generated by COMSOL with implemented parameters for the manual prototype. (ZIP) [file pone.0157040.s002.zip › SPMA_Rectangle_files/geom_geom1_wp1.png]

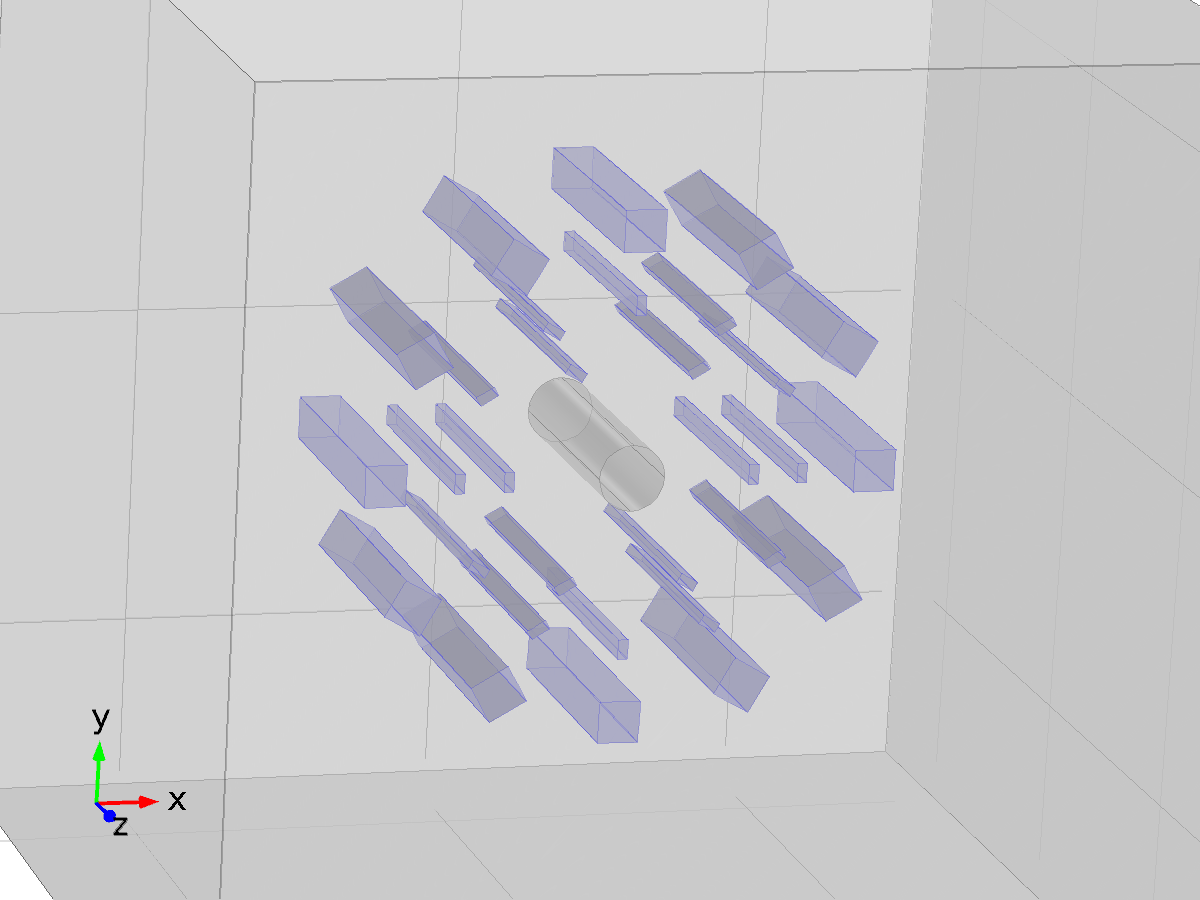

Supplement: S2 File — Model documentation generated by COMSOL with implemented parameters for the manual prototype. (ZIP) [file pone.0157040.s002.zip › SPMA_Rectangle_files/material_mat1.png]

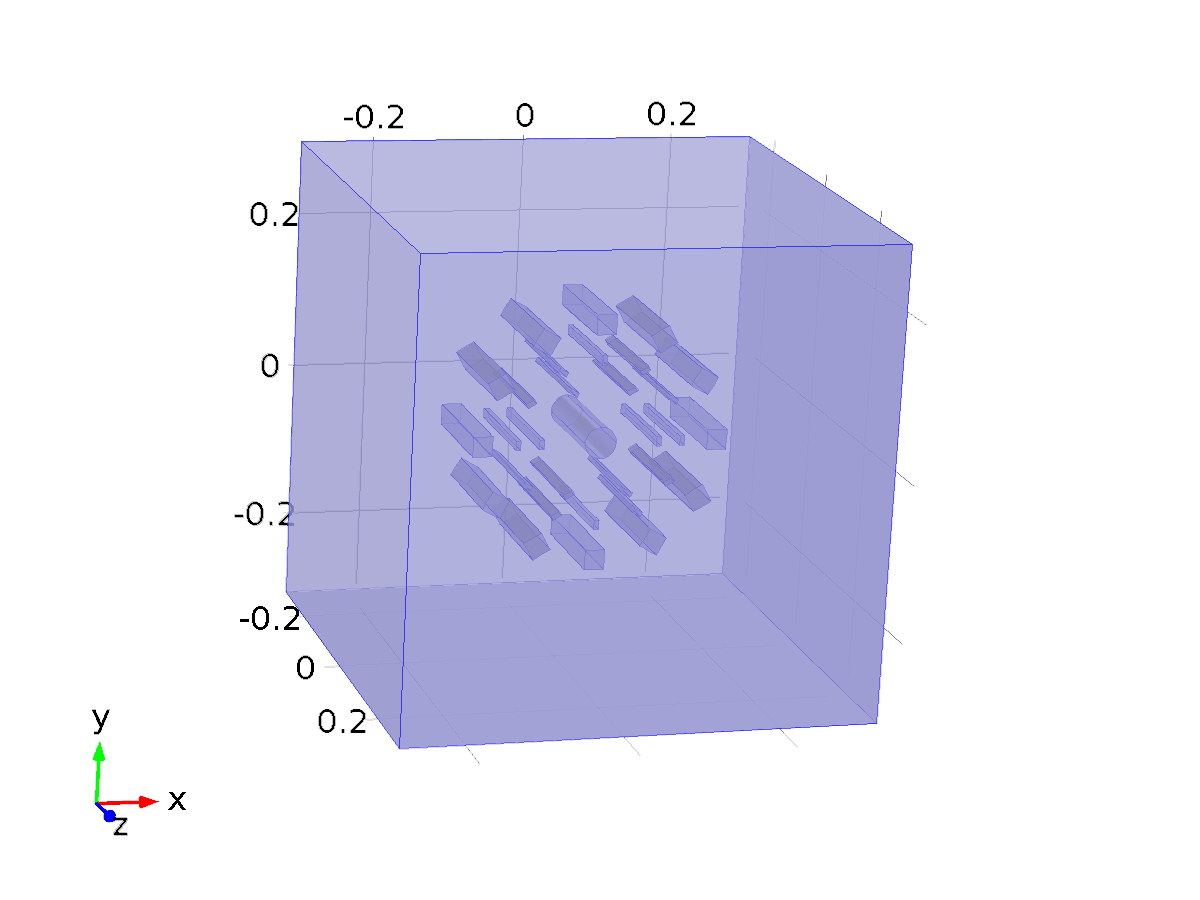

Supplement: S2 File — Model documentation generated by COMSOL with implemented parameters for the manual prototype. (ZIP) [file pone.0157040.s002.zip › SPMA_Rectangle_files/material_mat2.png]

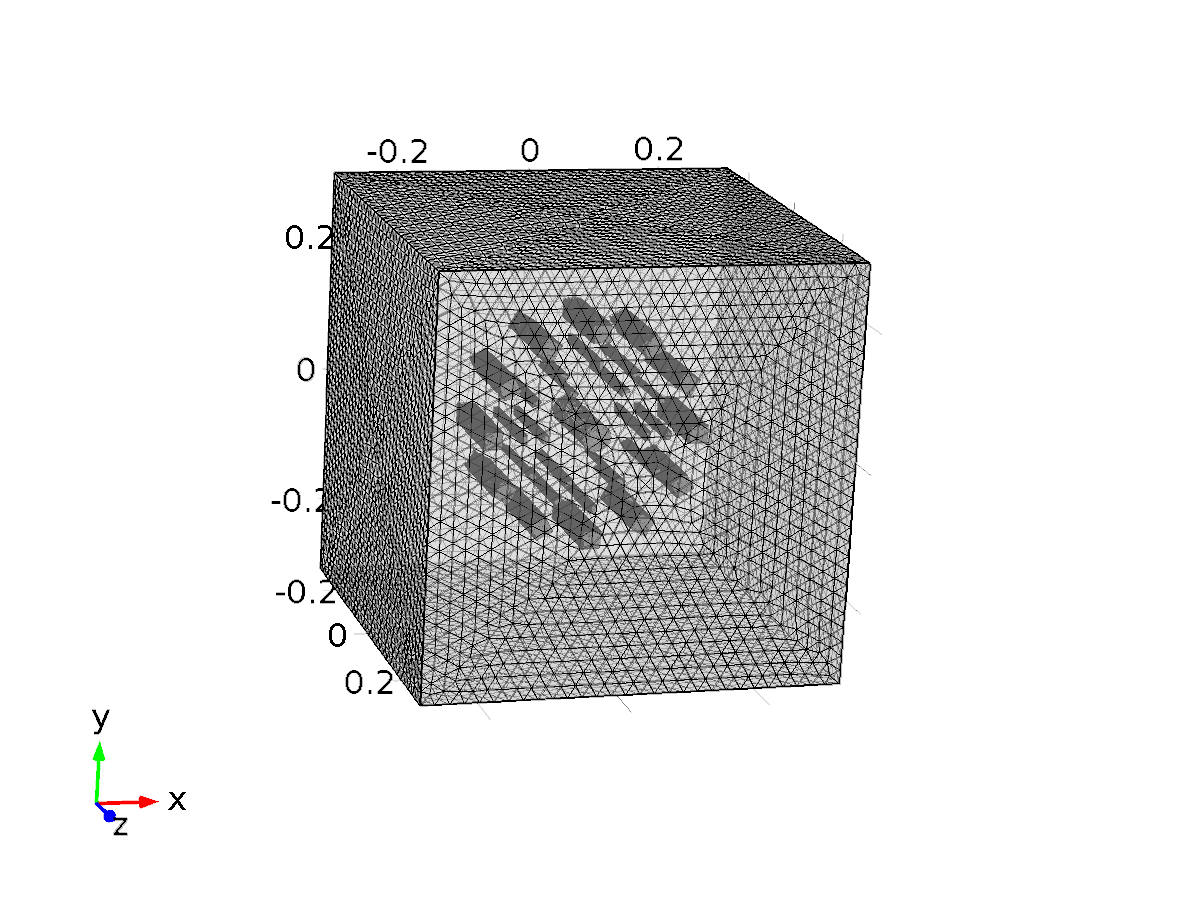

Supplement: S2 File — Model documentation generated by COMSOL with implemented parameters for the manual prototype. (ZIP) [file pone.0157040.s002.zip › SPMA_Rectangle_files/mesh_mesh1.png]

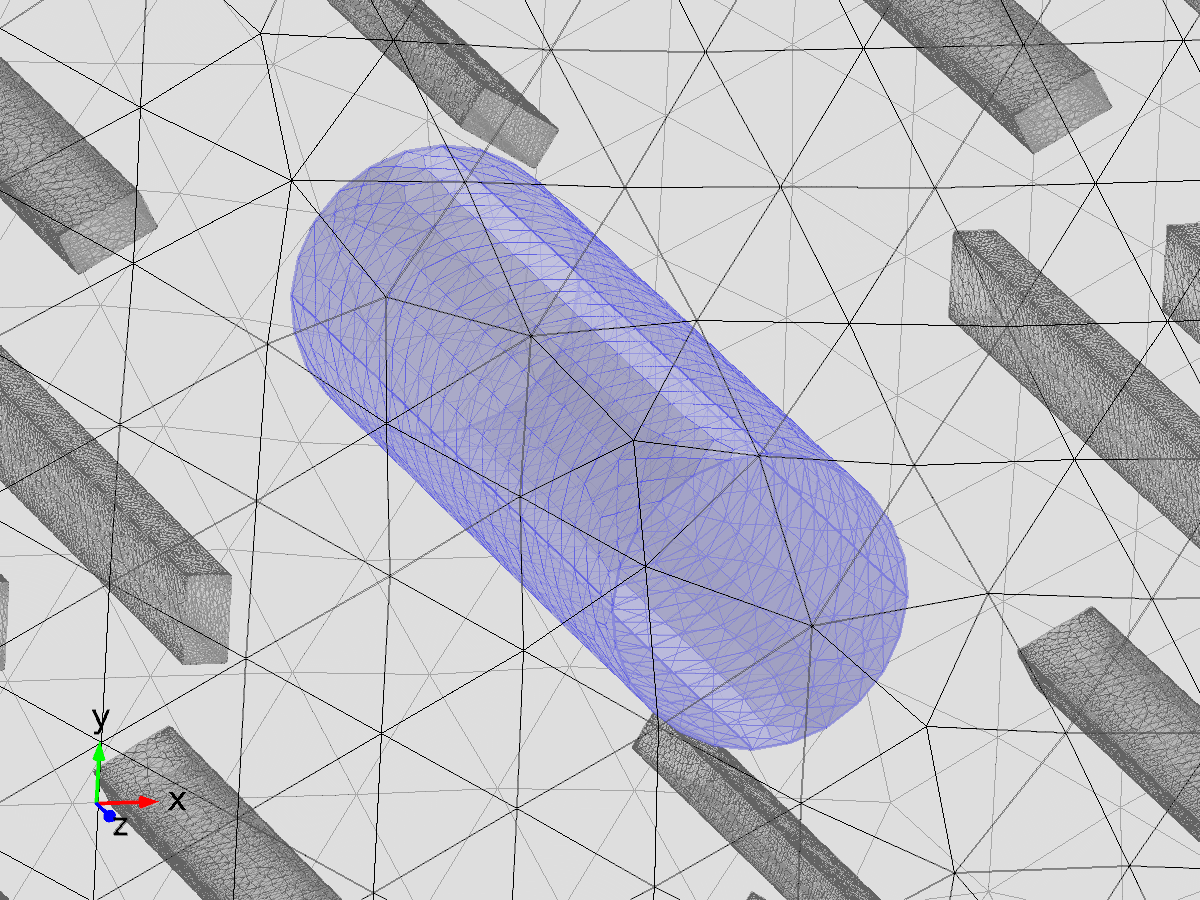

Supplement: S2 File — Model documentation generated by COMSOL with implemented parameters for the manual prototype. (ZIP) [file pone.0157040.s002.zip › SPMA_Rectangle_files/mesh_mesh1_ftet2_size1.png]

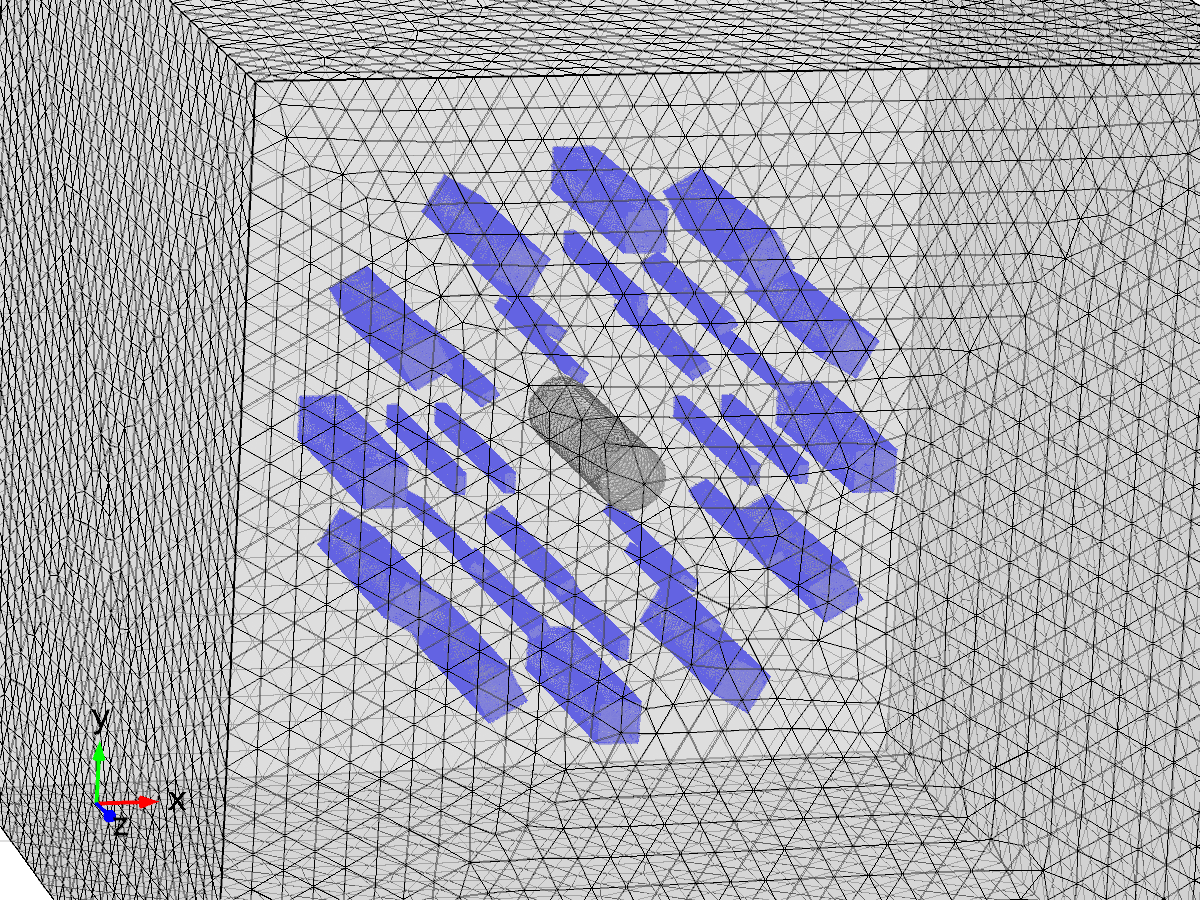

Supplement: S2 File — Model documentation generated by COMSOL with implemented parameters for the manual prototype. (ZIP) [file pone.0157040.s002.zip › SPMA_Rectangle_files/mesh_mesh1_ftet3_size1.png]

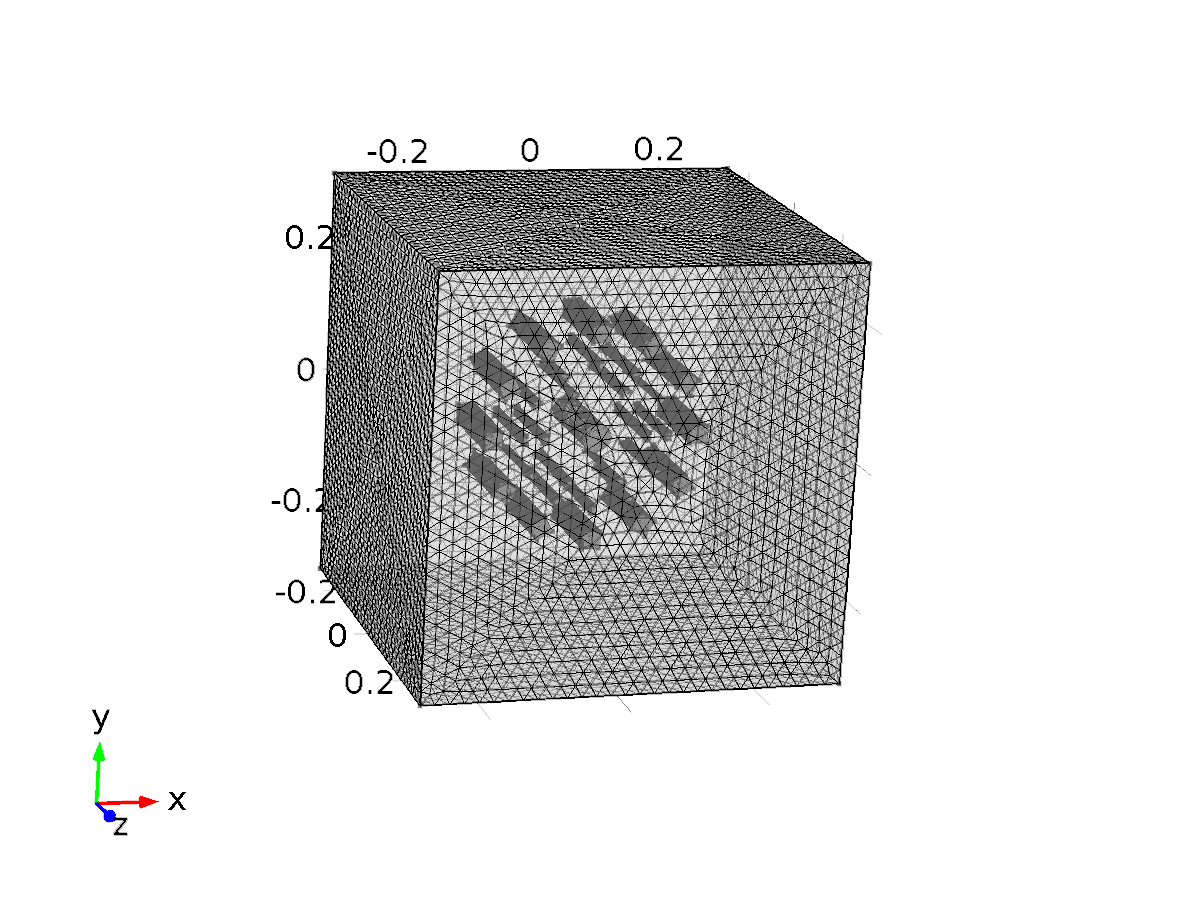

Supplement: S2 File — Model documentation generated by COMSOL with implemented parameters for the manual prototype. (ZIP) [file pone.0157040.s002.zip › SPMA_Rectangle_files/mesh_mesh1_ftet4_size1.png]

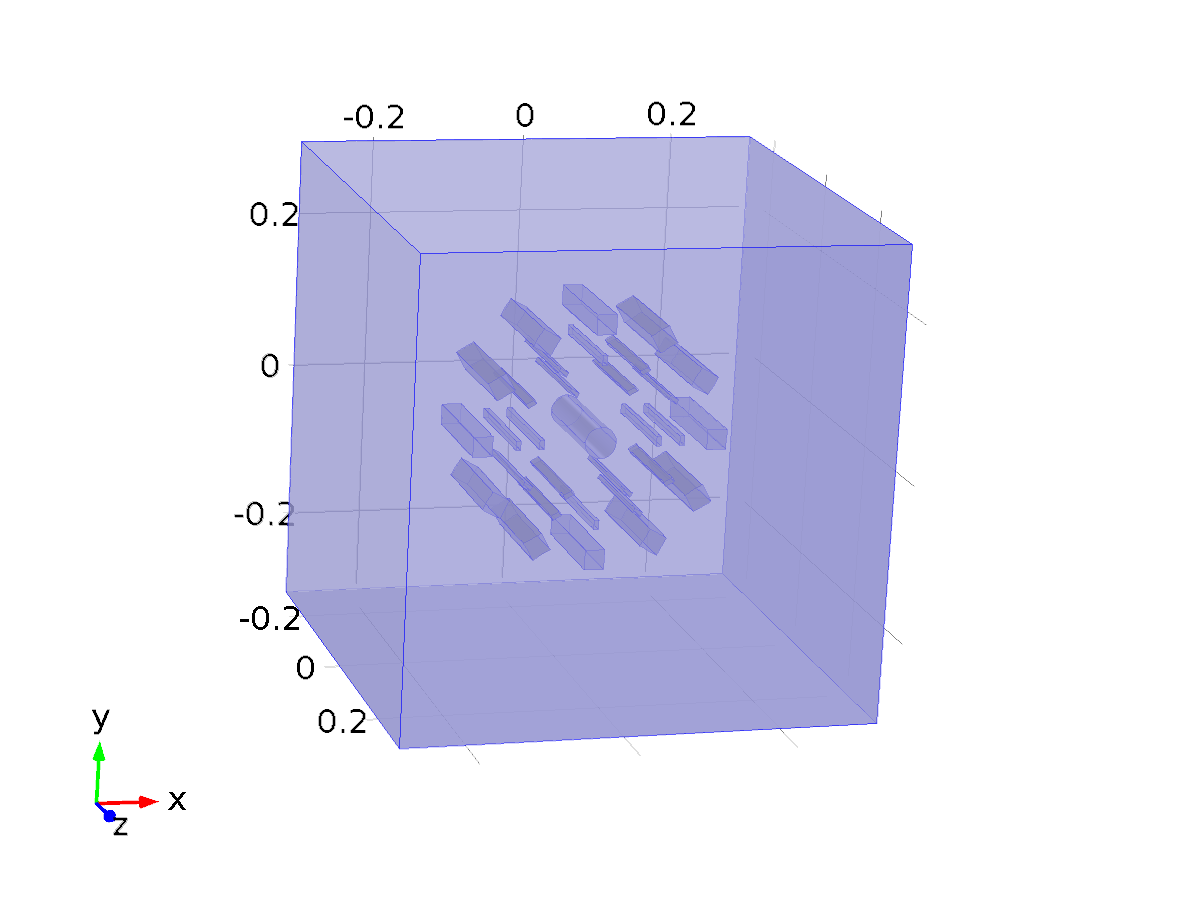

Supplement: S2 File — Model documentation generated by COMSOL with implemented parameters for the manual prototype. (ZIP) [file pone.0157040.s002.zip › SPMA_Rectangle_files/physics_mfnc_init1.png]

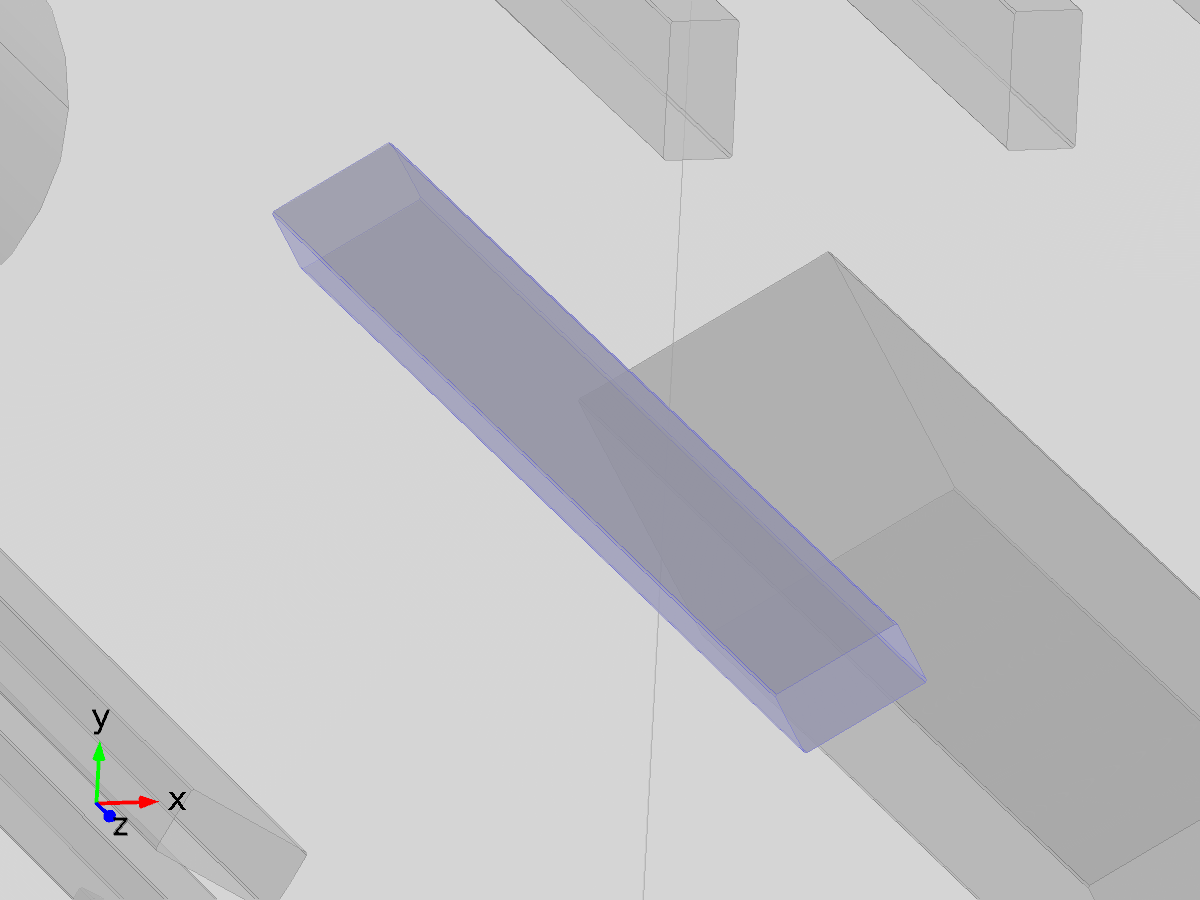

Supplement: S2 File — Model documentation generated by COMSOL with implemented parameters for the manual prototype. (ZIP) [file pone.0157040.s002.zip › SPMA_Rectangle_files/physics_mfnc_mfc12.png]

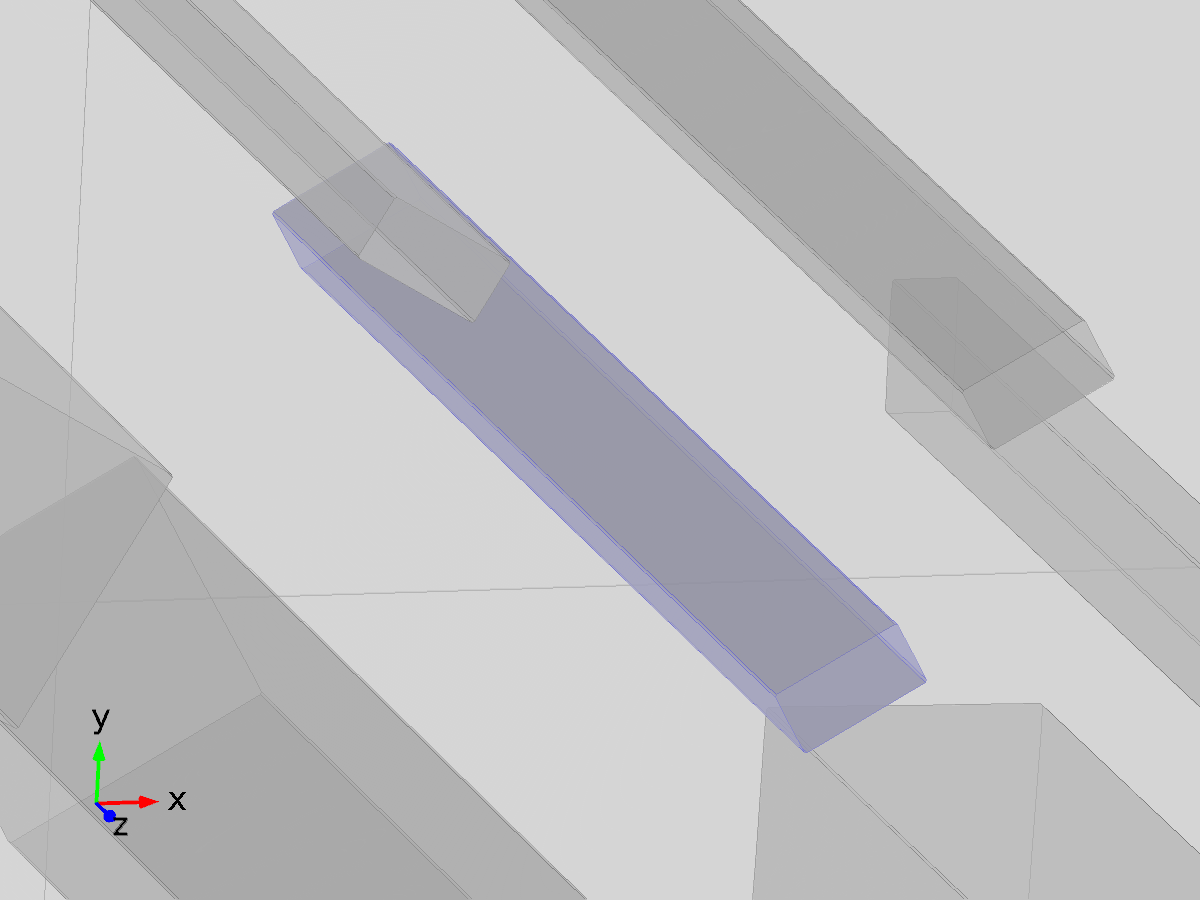

Supplement: S2 File — Model documentation generated by COMSOL with implemented parameters for the manual prototype. (ZIP) [file pone.0157040.s002.zip › SPMA_Rectangle_files/physics_mfnc_mfc13.png]

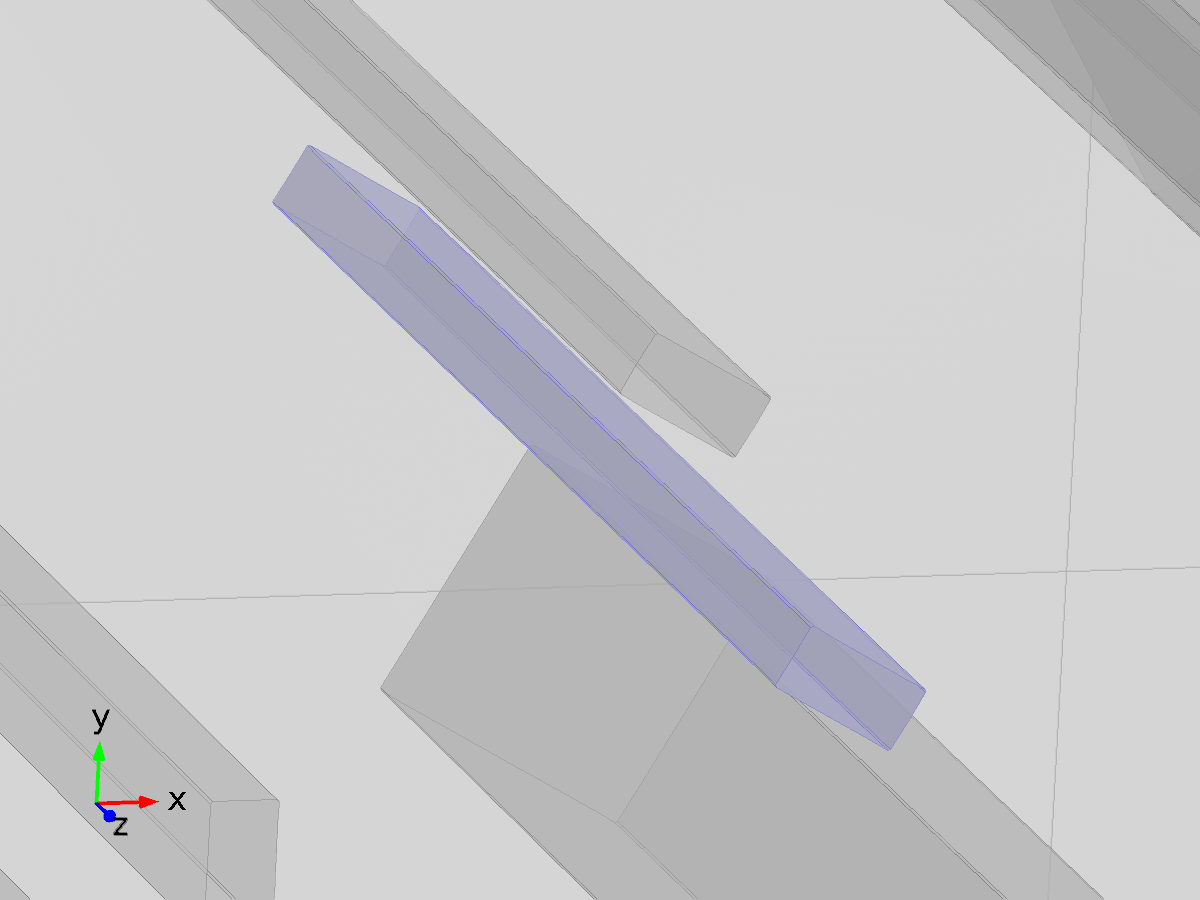

Supplement: S2 File — Model documentation generated by COMSOL with implemented parameters for the manual prototype. (ZIP) [file pone.0157040.s002.zip › SPMA_Rectangle_files/physics_mfnc_mfc14.png]

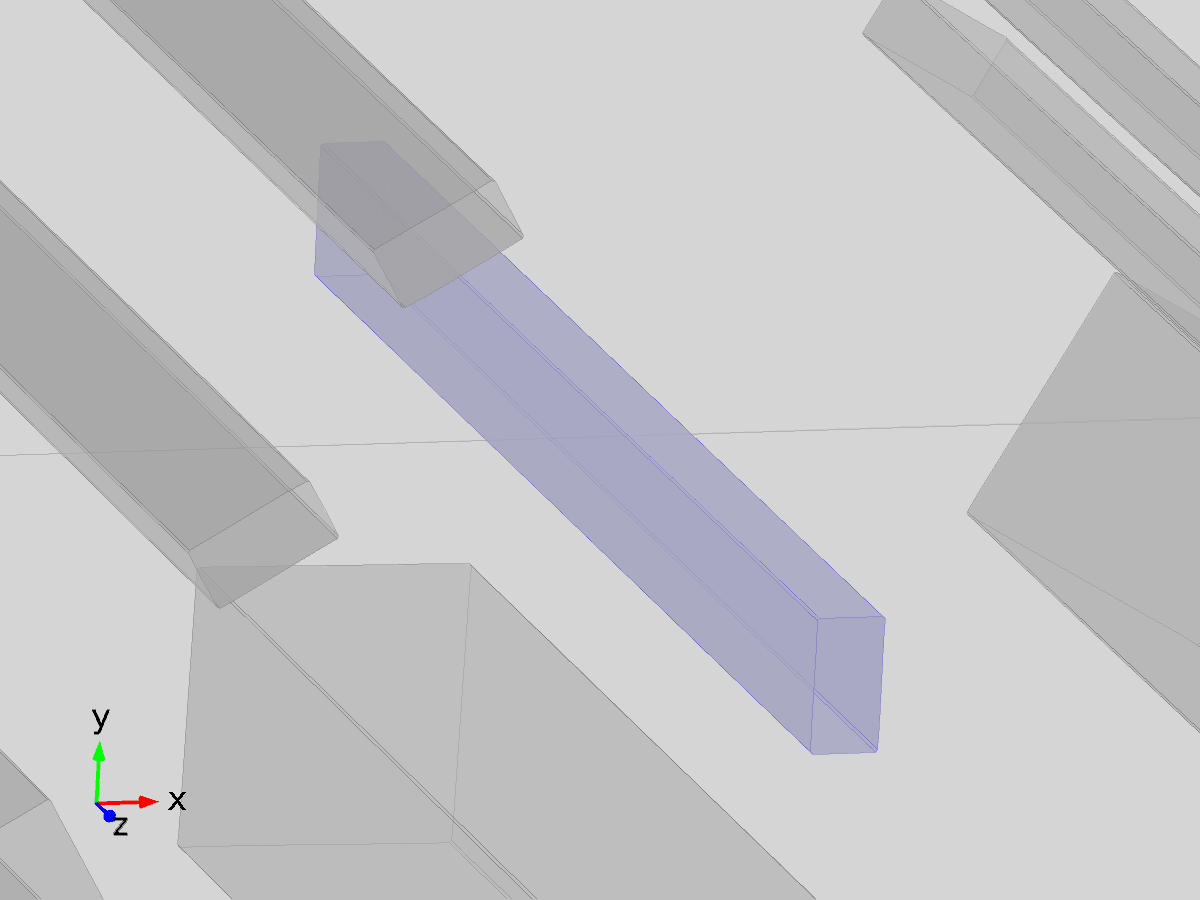

Supplement: S2 File — Model documentation generated by COMSOL with implemented parameters for the manual prototype. (ZIP) [file pone.0157040.s002.zip › SPMA_Rectangle_files/physics_mfnc_mfc15.png]

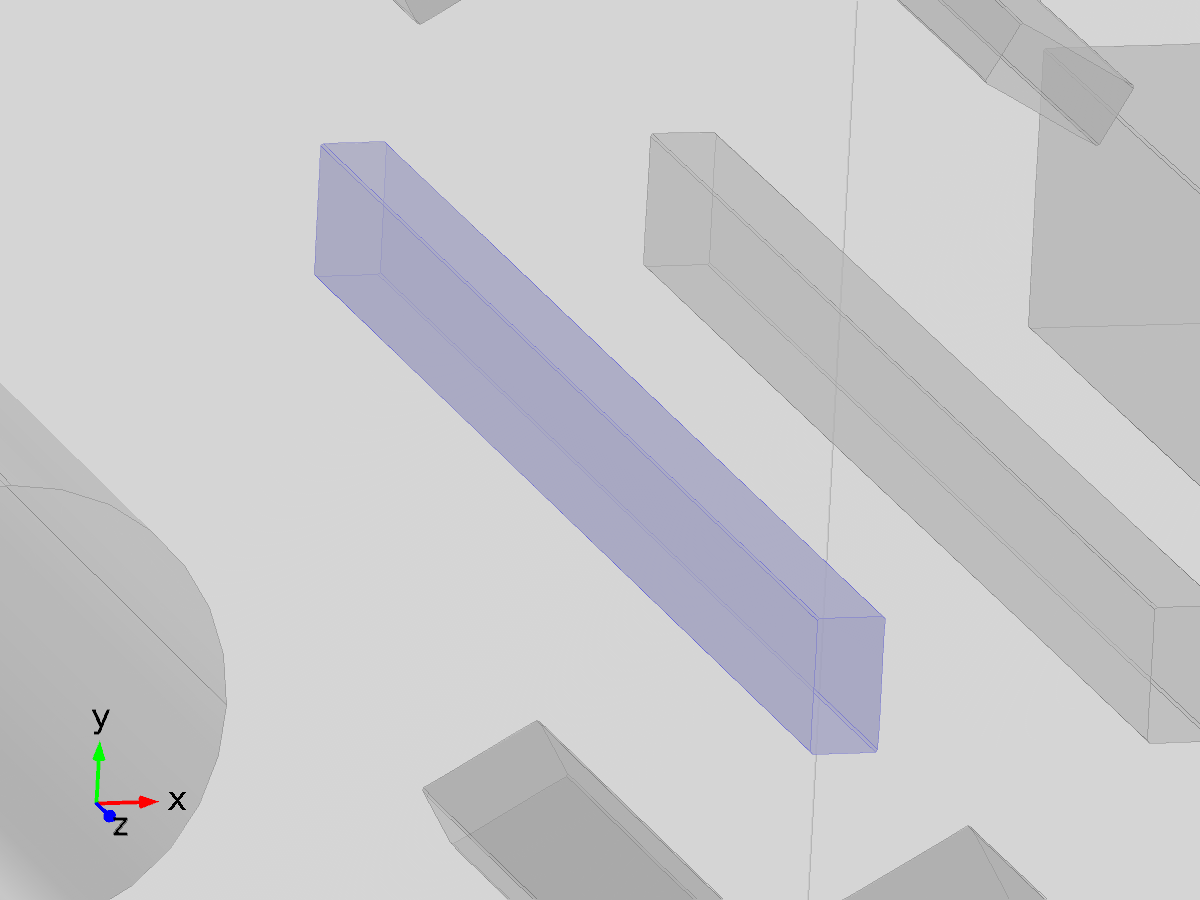

Supplement: S2 File — Model documentation generated by COMSOL with implemented parameters for the manual prototype. (ZIP) [file pone.0157040.s002.zip › SPMA_Rectangle_files/physics_mfnc_mfc16.png]

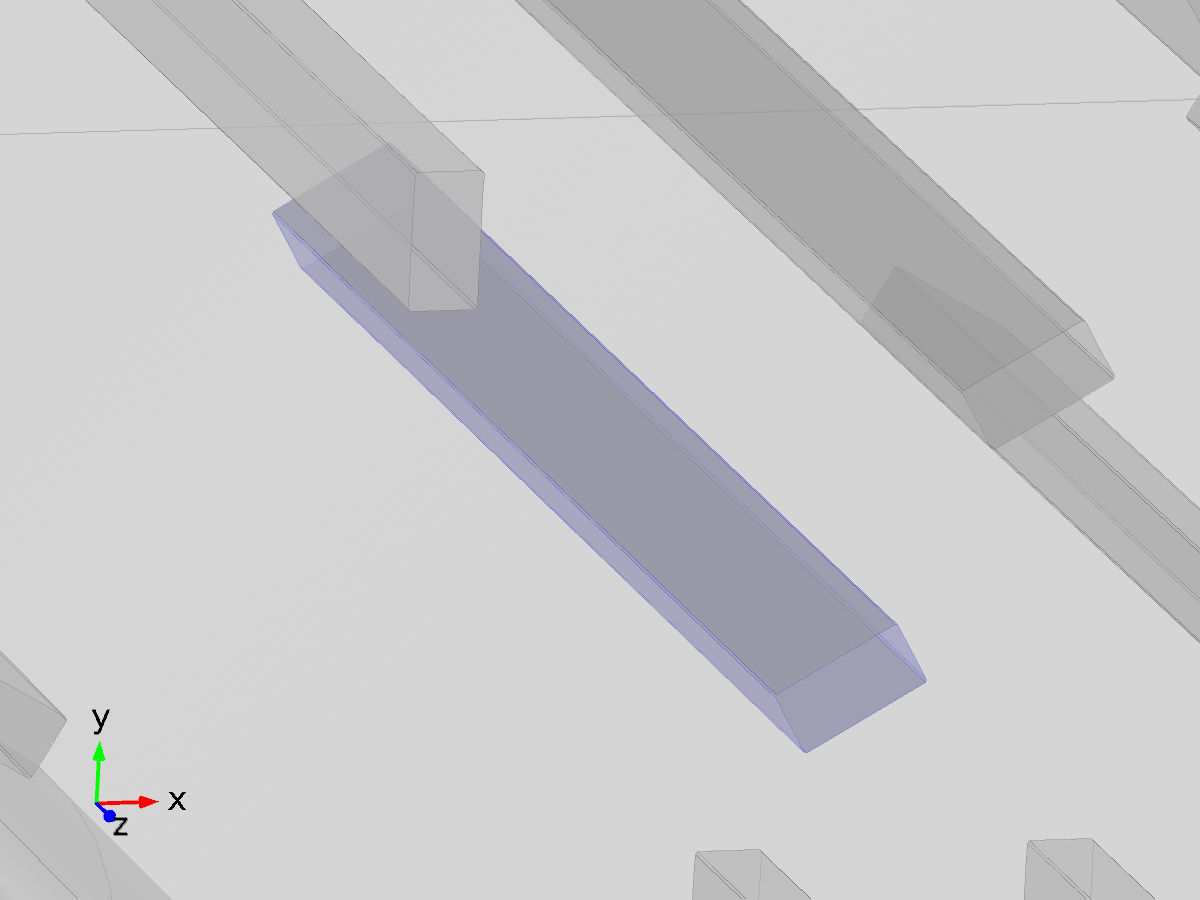

Supplement: S2 File — Model documentation generated by COMSOL with implemented parameters for the manual prototype. (ZIP) [file pone.0157040.s002.zip › SPMA_Rectangle_files/physics_mfnc_mfc18.png]

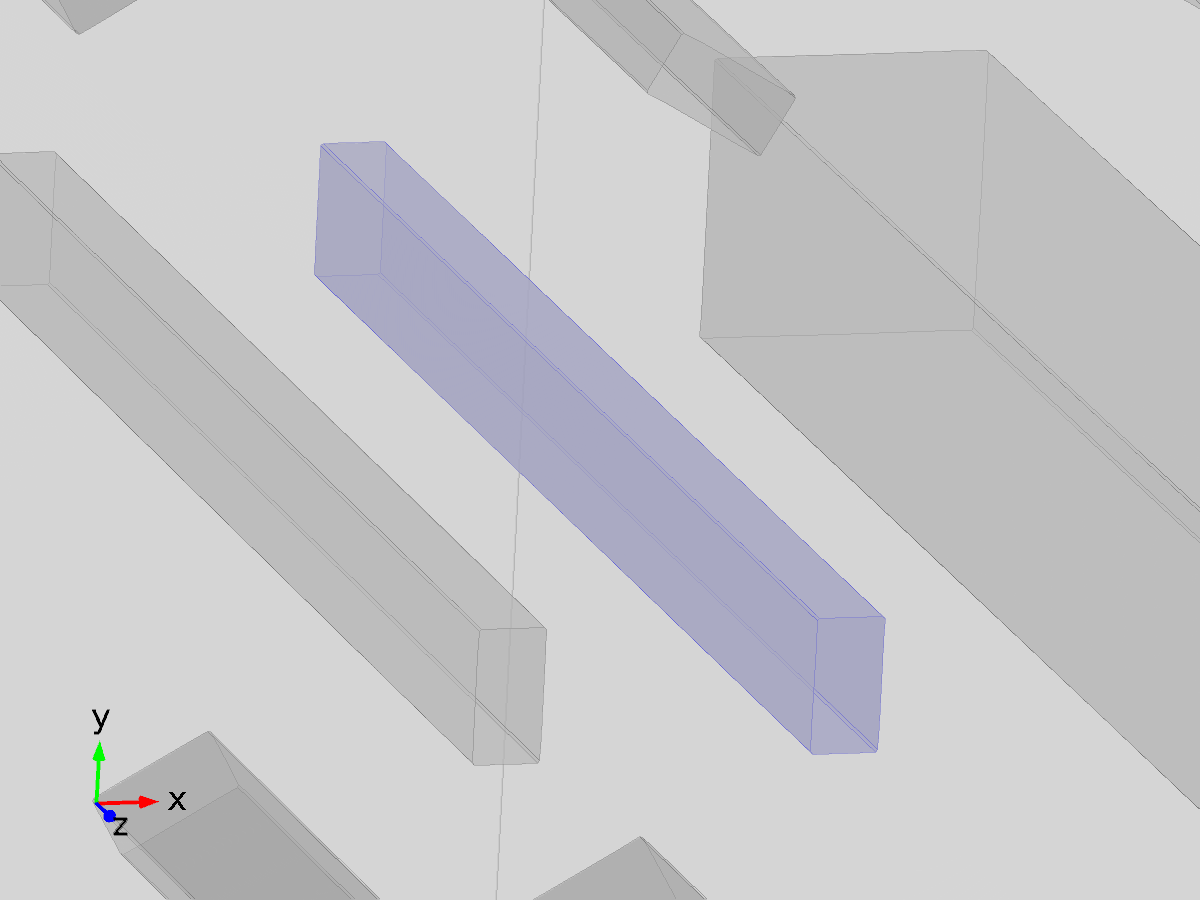

Supplement: S2 File — Model documentation generated by COMSOL with implemented parameters for the manual prototype. (ZIP) [file pone.0157040.s002.zip › SPMA_Rectangle_files/physics_mfnc_mfc2.png]

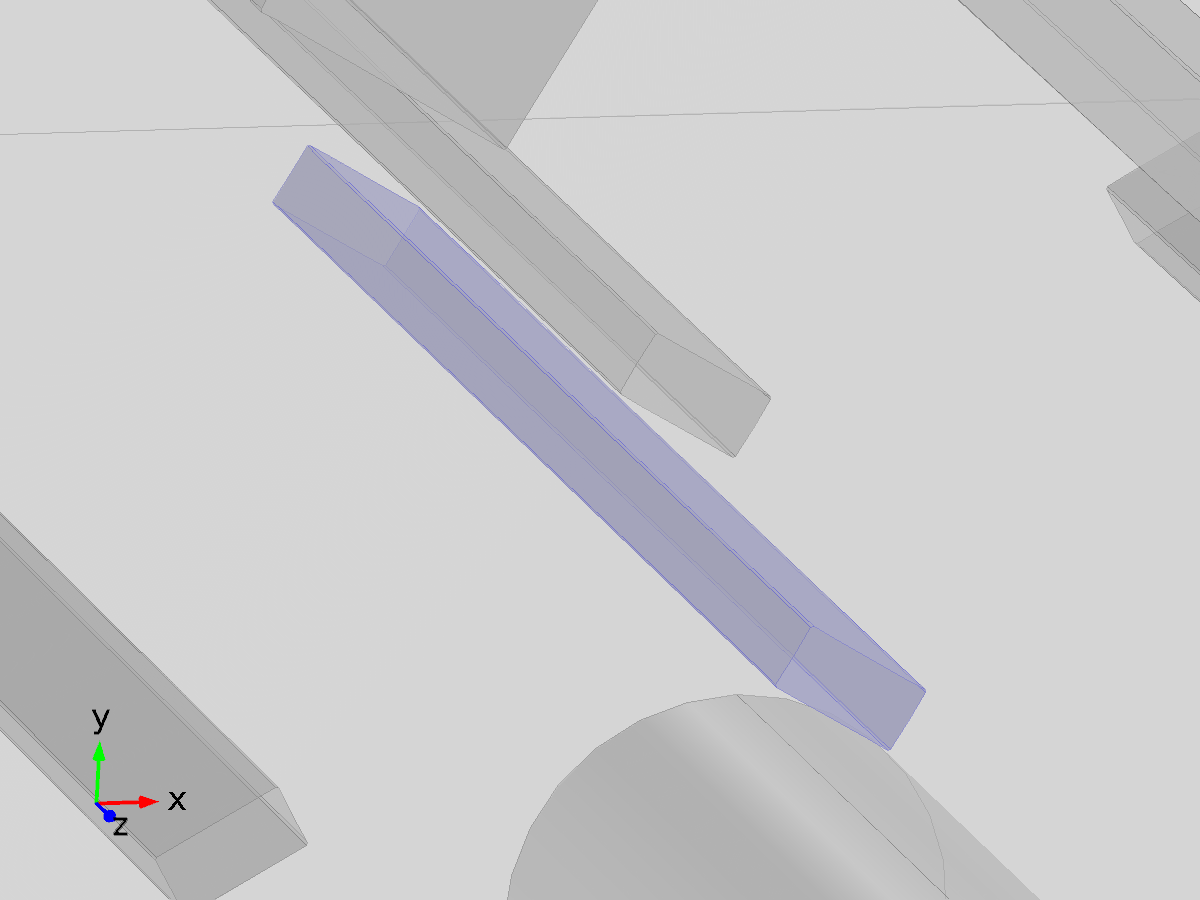

Supplement: S2 File — Model documentation generated by COMSOL with implemented parameters for the manual prototype. (ZIP) [file pone.0157040.s002.zip › SPMA_Rectangle_files/physics_mfnc_mfc20.png]

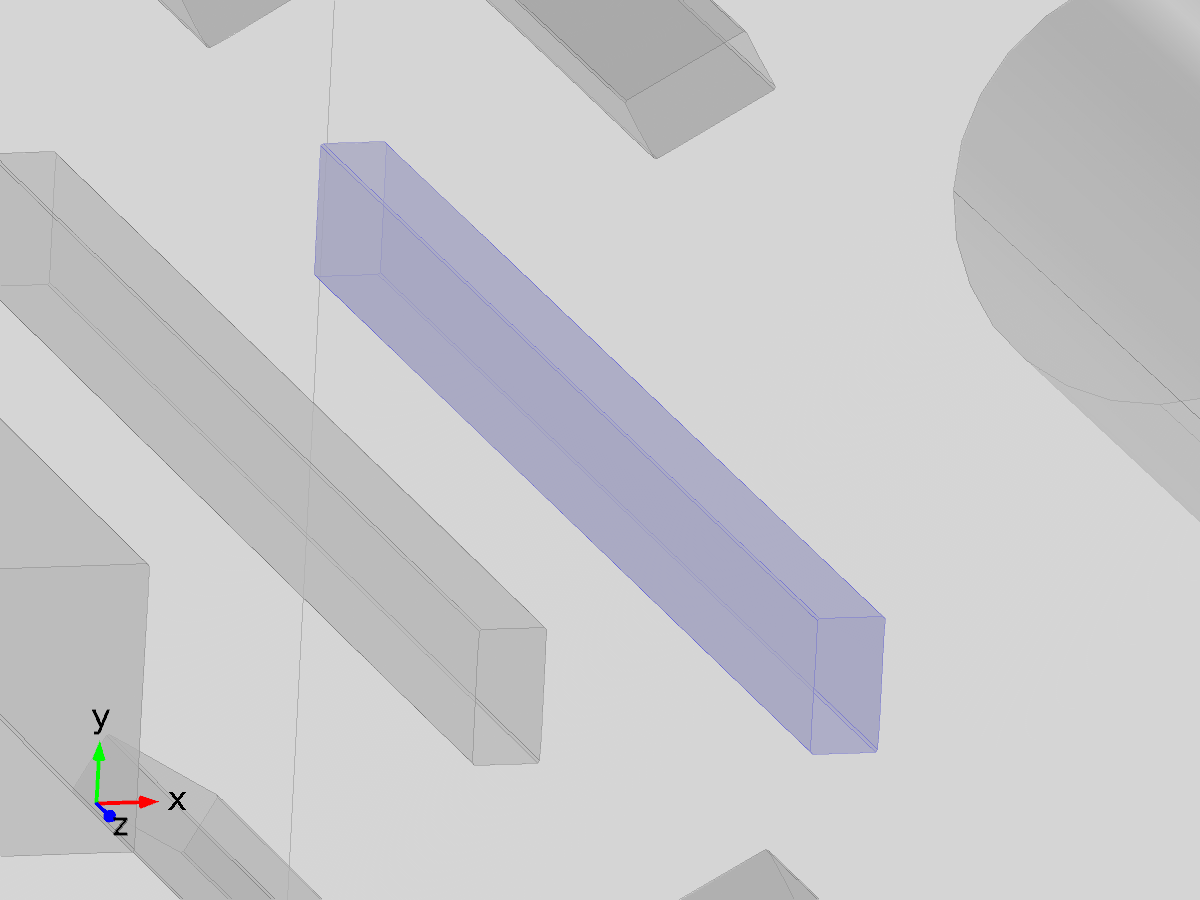

Supplement: S2 File — Model documentation generated by COMSOL with implemented parameters for the manual prototype. (ZIP) [file pone.0157040.s002.zip › SPMA_Rectangle_files/physics_mfnc_mfc22.png]

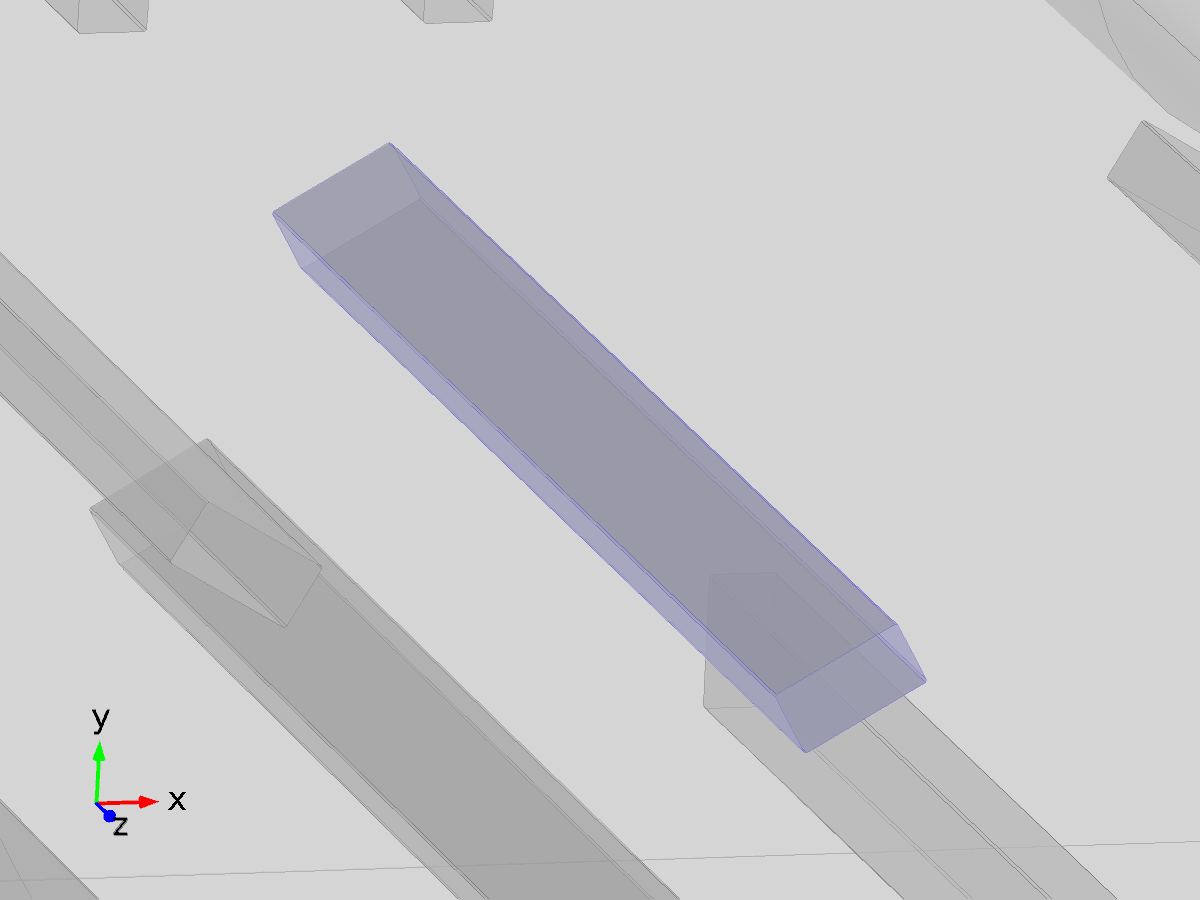

Supplement: S2 File — Model documentation generated by COMSOL with implemented parameters for the manual prototype. (ZIP) [file pone.0157040.s002.zip › SPMA_Rectangle_files/physics_mfnc_mfc24.png]

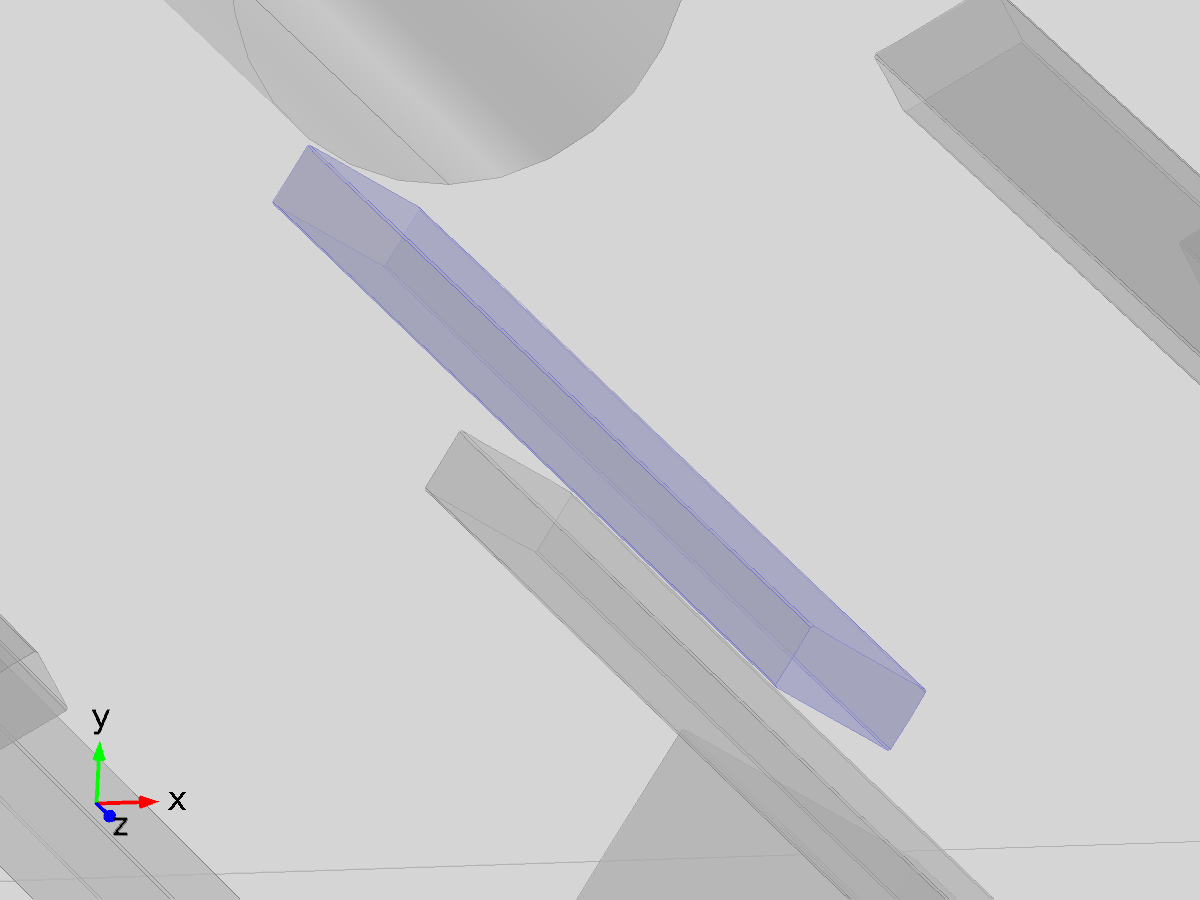

Supplement: S2 File — Model documentation generated by COMSOL with implemented parameters for the manual prototype. (ZIP) [file pone.0157040.s002.zip › SPMA_Rectangle_files/physics_mfnc_mfc26.png]

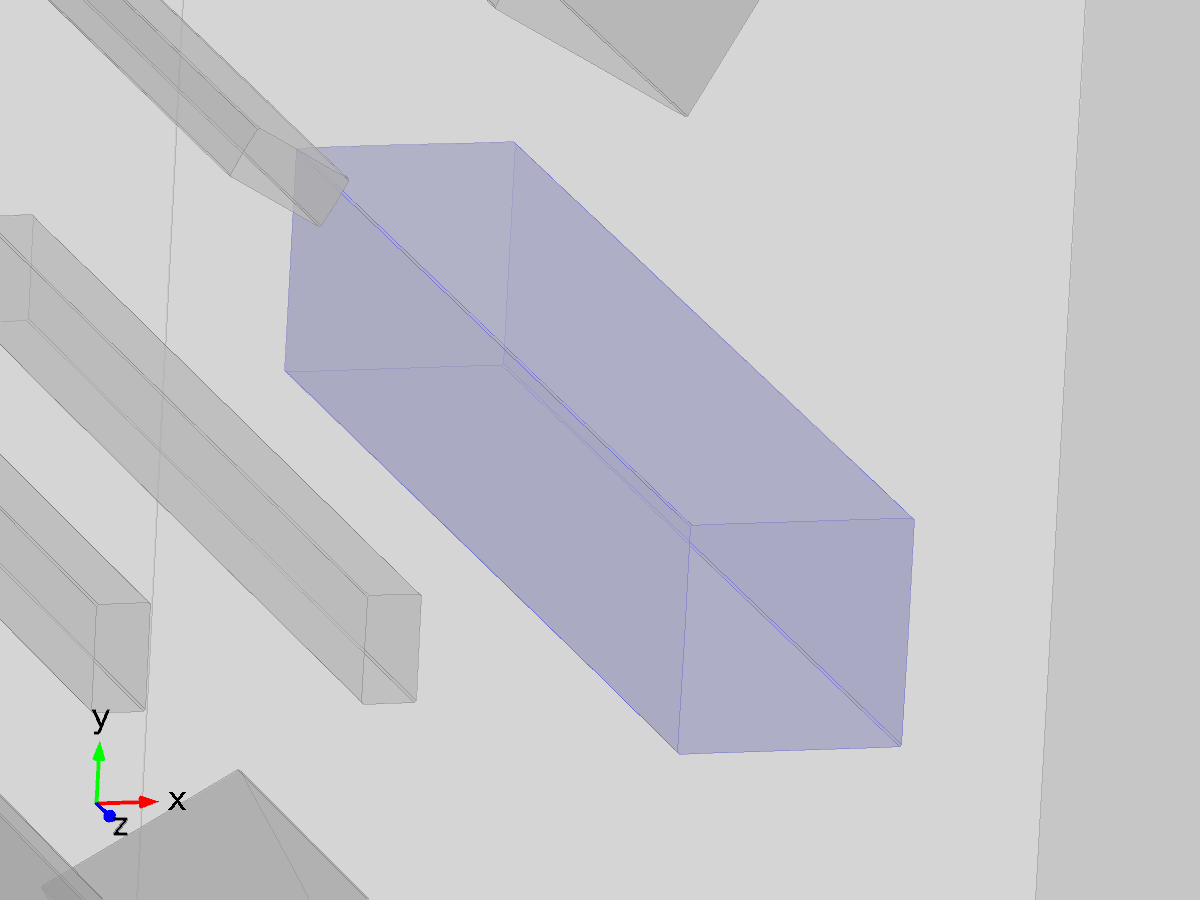

Supplement: S2 File — Model documentation generated by COMSOL with implemented parameters for the manual prototype. (ZIP) [file pone.0157040.s002.zip › SPMA_Rectangle_files/physics_mfnc_mfc28.png]

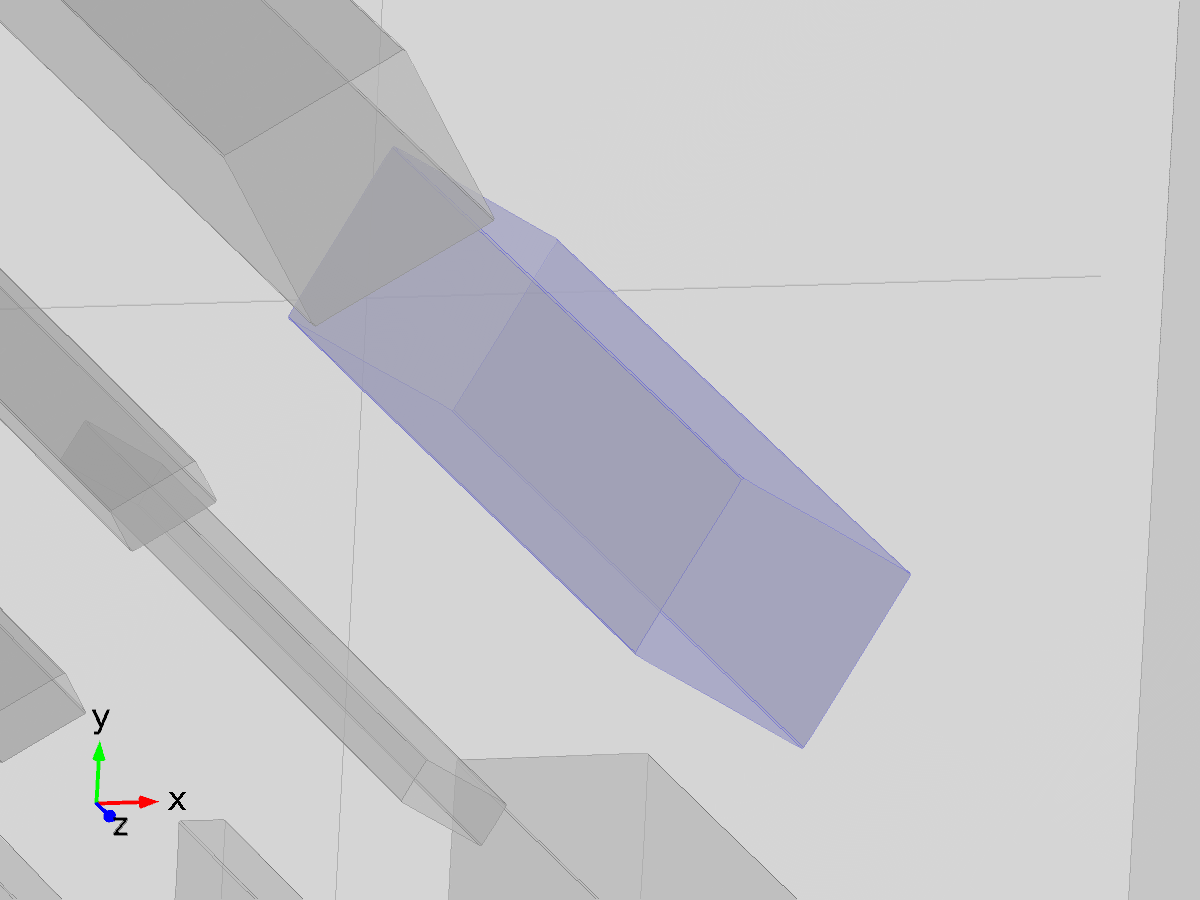

Supplement: S2 File — Model documentation generated by COMSOL with implemented parameters for the manual prototype. (ZIP) [file pone.0157040.s002.zip › SPMA_Rectangle_files/physics_mfnc_mfc29.png]

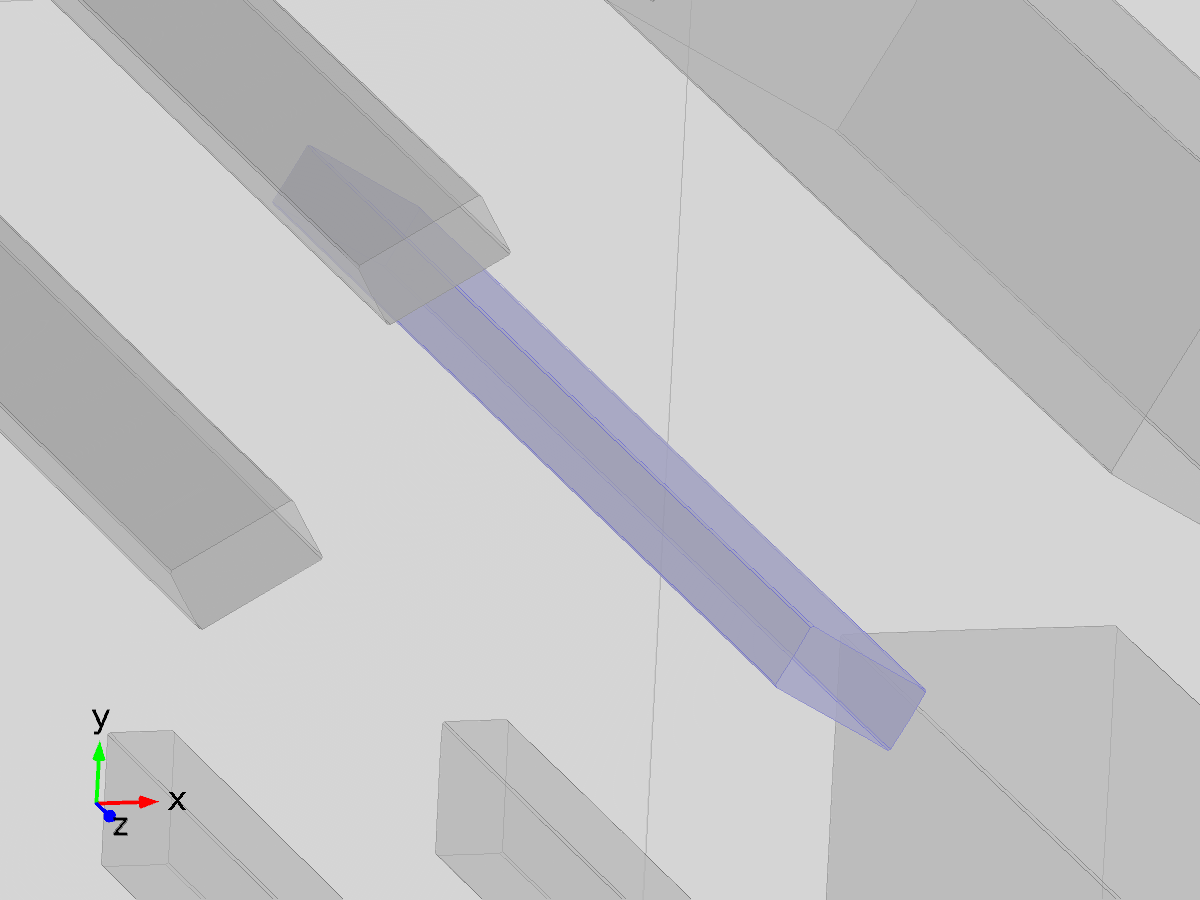

Supplement: S2 File — Model documentation generated by COMSOL with implemented parameters for the manual prototype. (ZIP) [file pone.0157040.s002.zip › SPMA_Rectangle_files/physics_mfnc_mfc3.png]

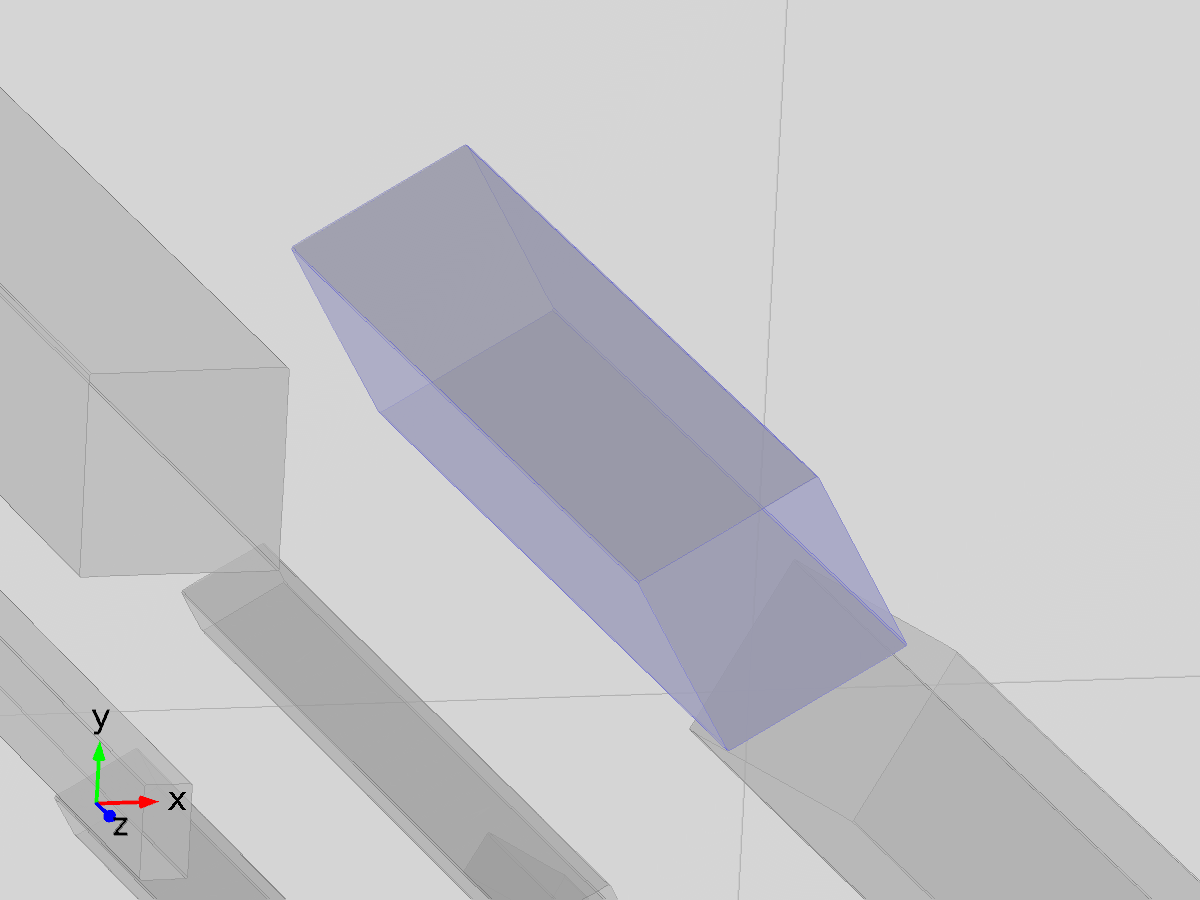

Supplement: S2 File — Model documentation generated by COMSOL with implemented parameters for the manual prototype. (ZIP) [file pone.0157040.s002.zip › SPMA_Rectangle_files/physics_mfnc_mfc30.png]

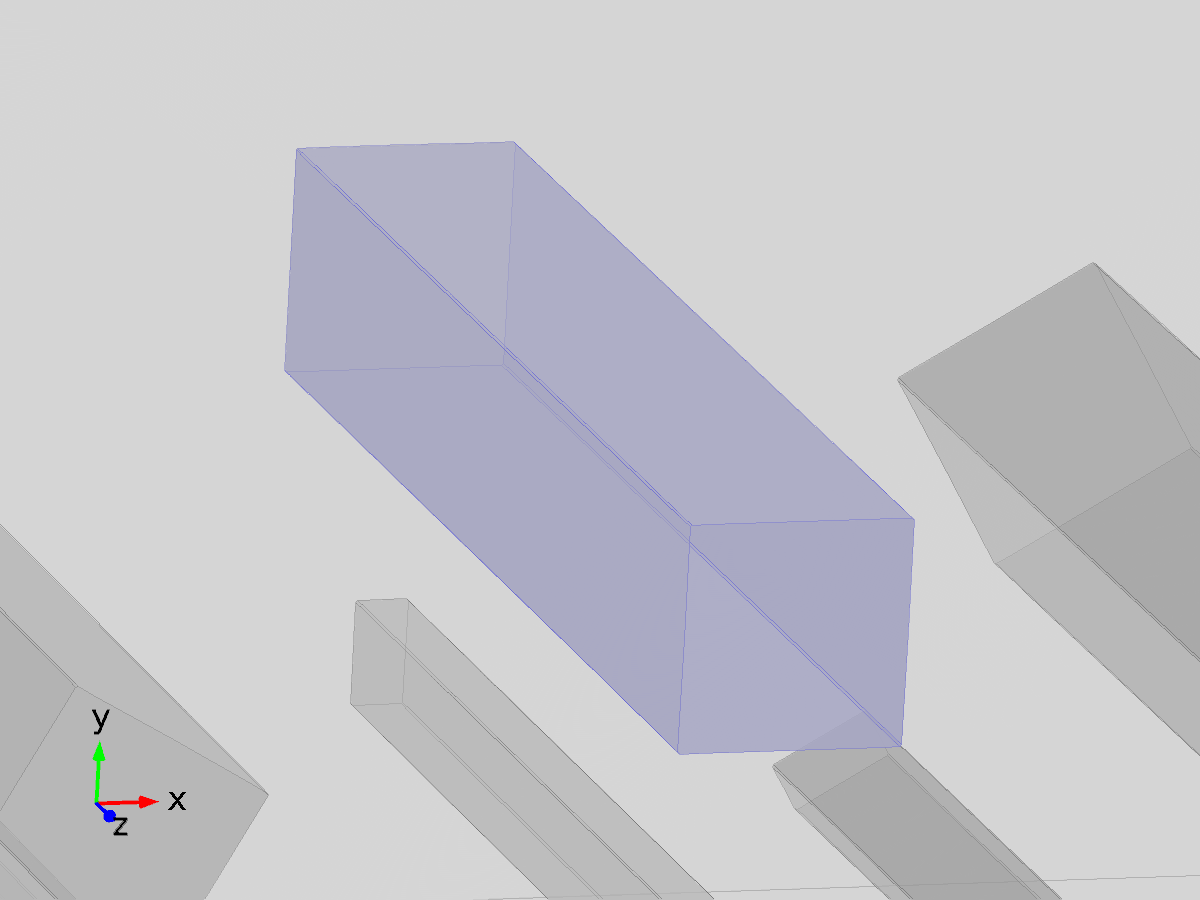

Supplement: S2 File — Model documentation generated by COMSOL with implemented parameters for the manual prototype. (ZIP) [file pone.0157040.s002.zip › SPMA_Rectangle_files/physics_mfnc_mfc31.png]

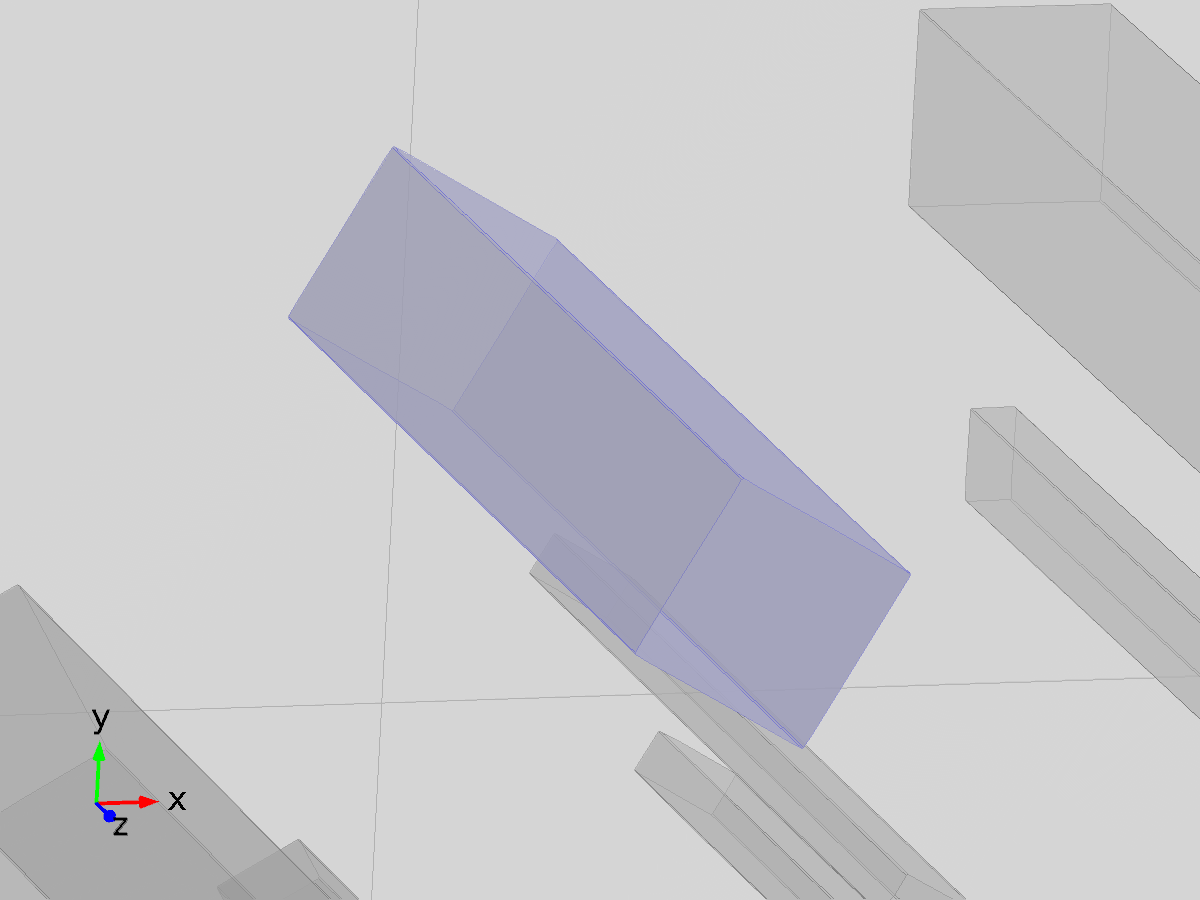

Supplement: S2 File — Model documentation generated by COMSOL with implemented parameters for the manual prototype. (ZIP) [file pone.0157040.s002.zip › SPMA_Rectangle_files/physics_mfnc_mfc32.png]

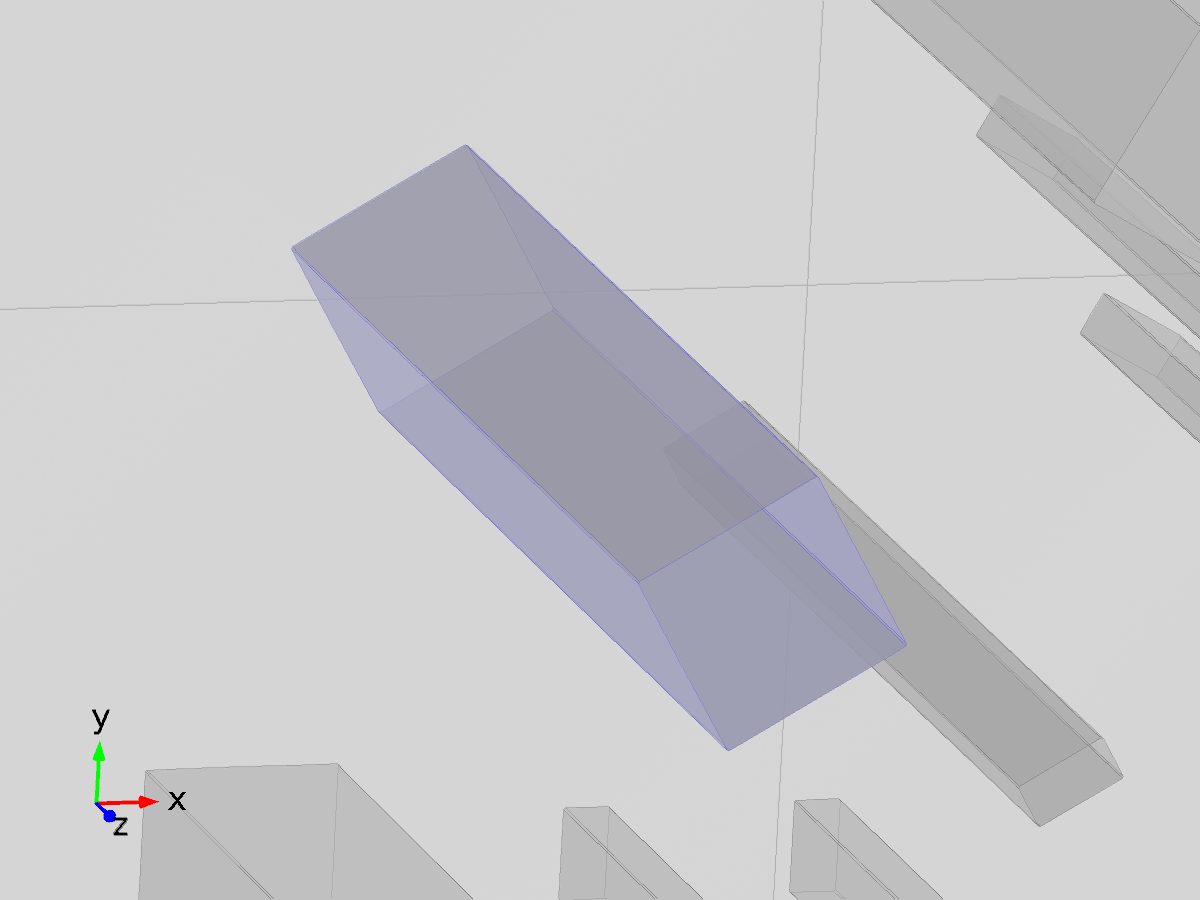

Supplement: S2 File — Model documentation generated by COMSOL with implemented parameters for the manual prototype. (ZIP) [file pone.0157040.s002.zip › SPMA_Rectangle_files/physics_mfnc_mfc33.png]

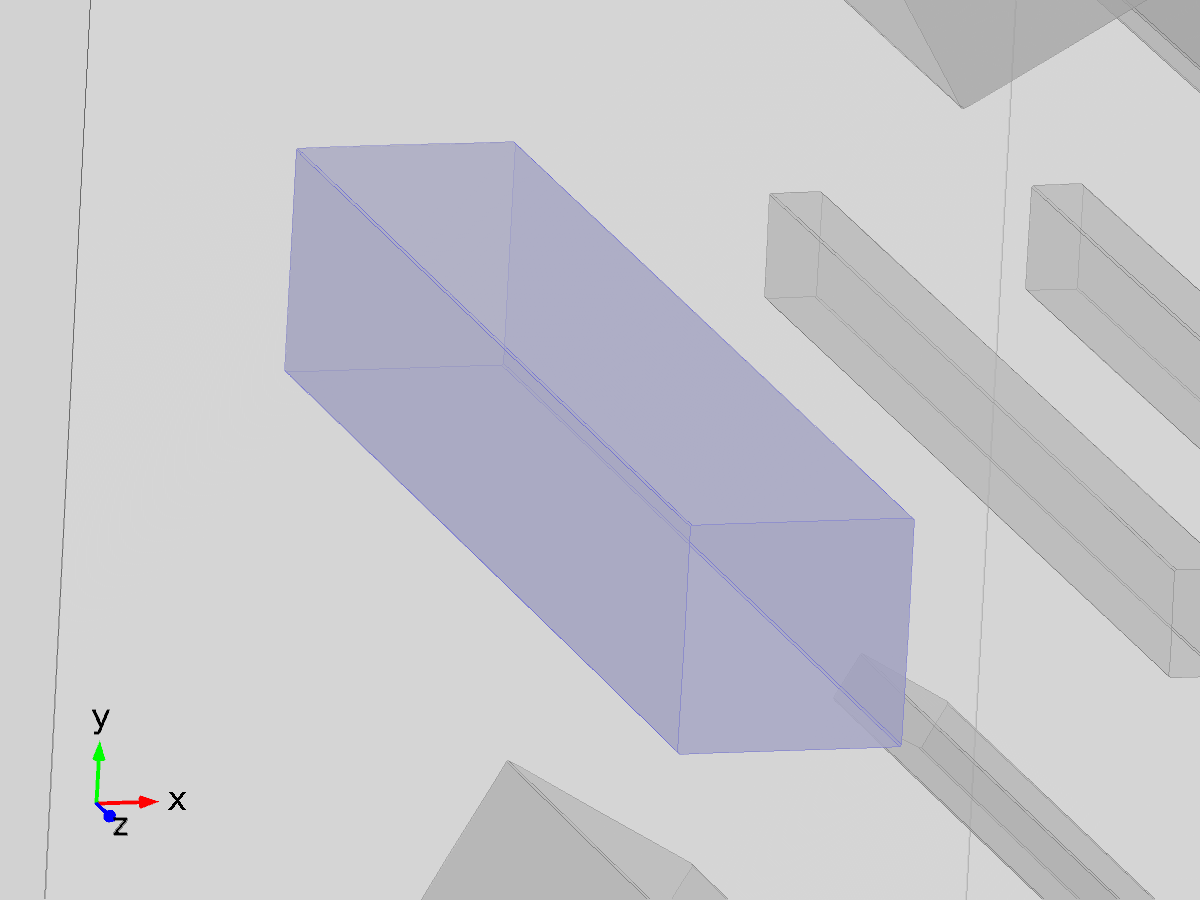

Supplement: S2 File — Model documentation generated by COMSOL with implemented parameters for the manual prototype. (ZIP) [file pone.0157040.s002.zip › SPMA_Rectangle_files/physics_mfnc_mfc34.png]

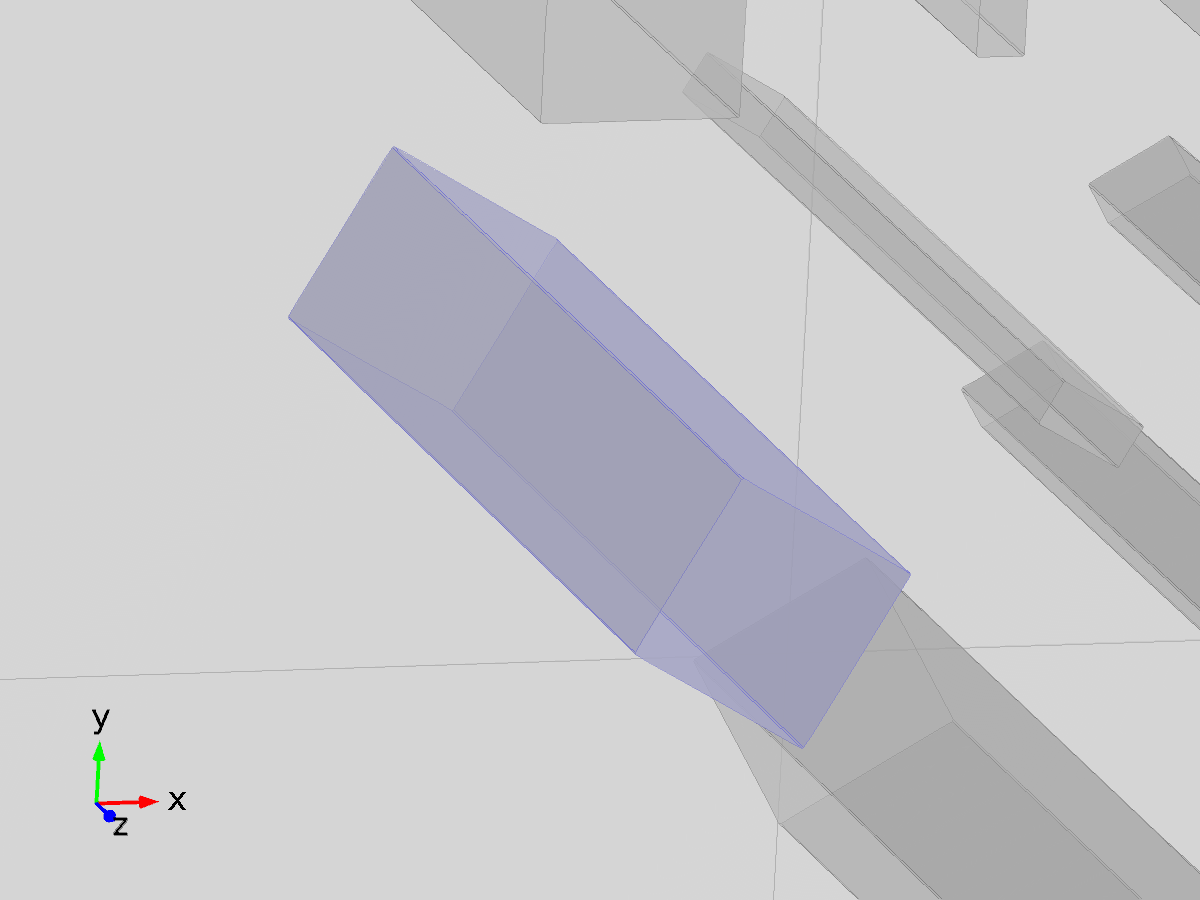

Supplement: S2 File — Model documentation generated by COMSOL with implemented parameters for the manual prototype. (ZIP) [file pone.0157040.s002.zip › SPMA_Rectangle_files/physics_mfnc_mfc35.png]

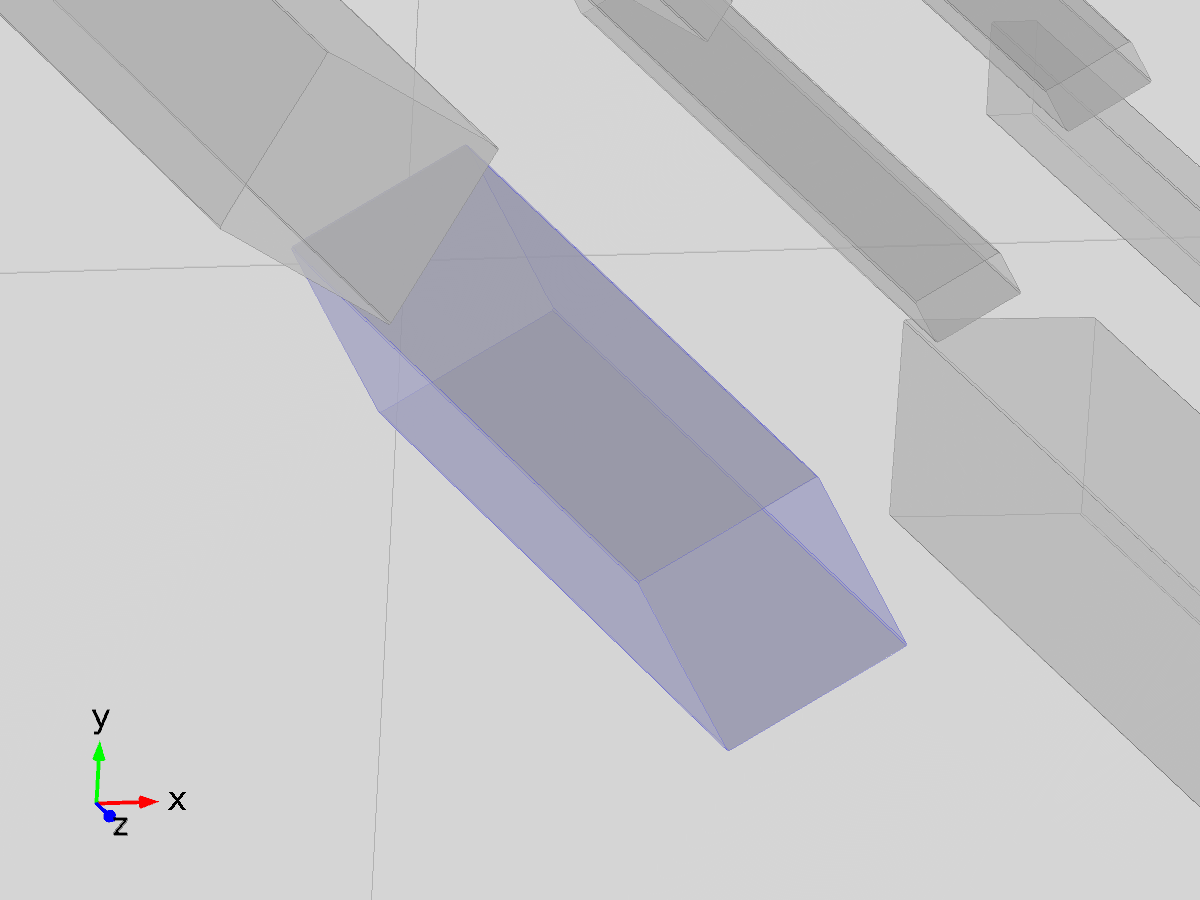

Supplement: S2 File — Model documentation generated by COMSOL with implemented parameters for the manual prototype. (ZIP) [file pone.0157040.s002.zip › SPMA_Rectangle_files/physics_mfnc_mfc36.png]

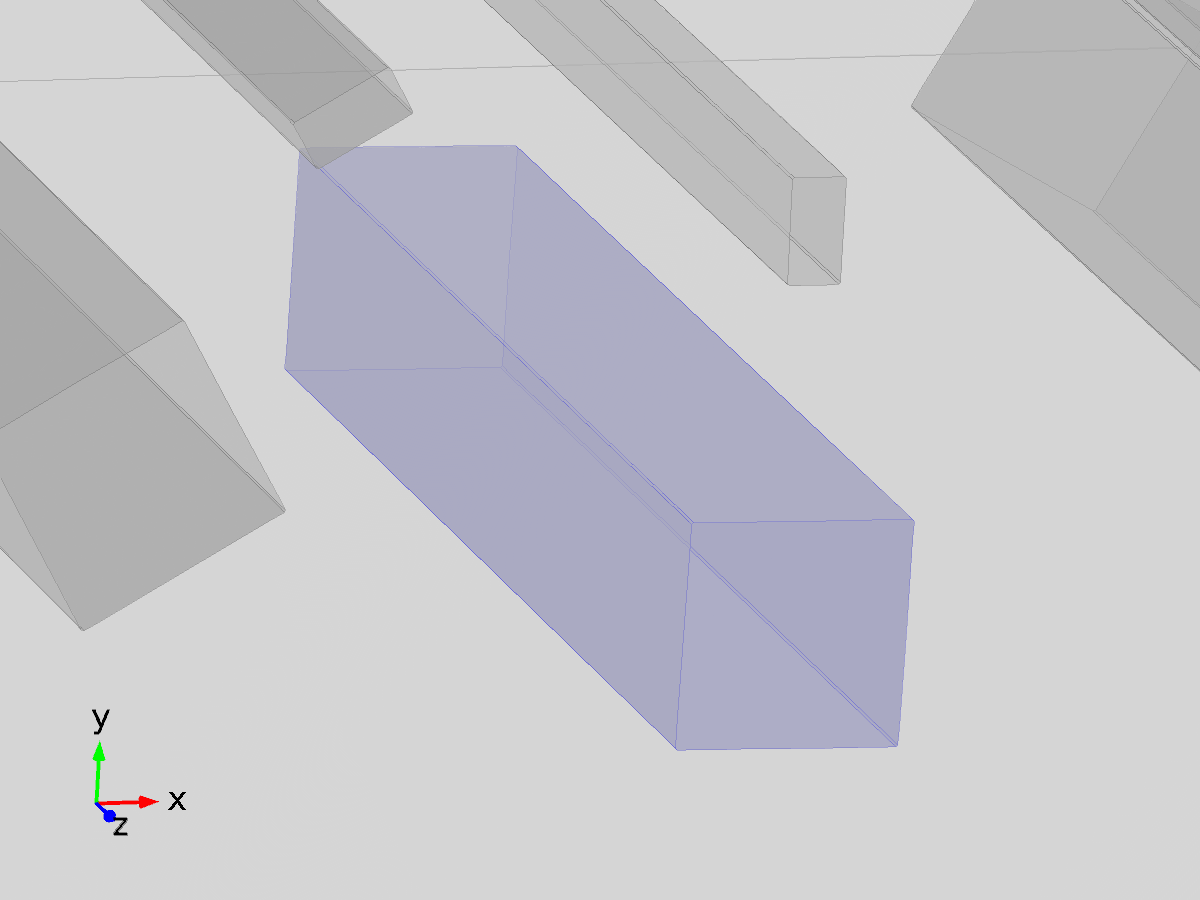

Supplement: S2 File — Model documentation generated by COMSOL with implemented parameters for the manual prototype. (ZIP) [file pone.0157040.s002.zip › SPMA_Rectangle_files/physics_mfnc_mfc37.png]

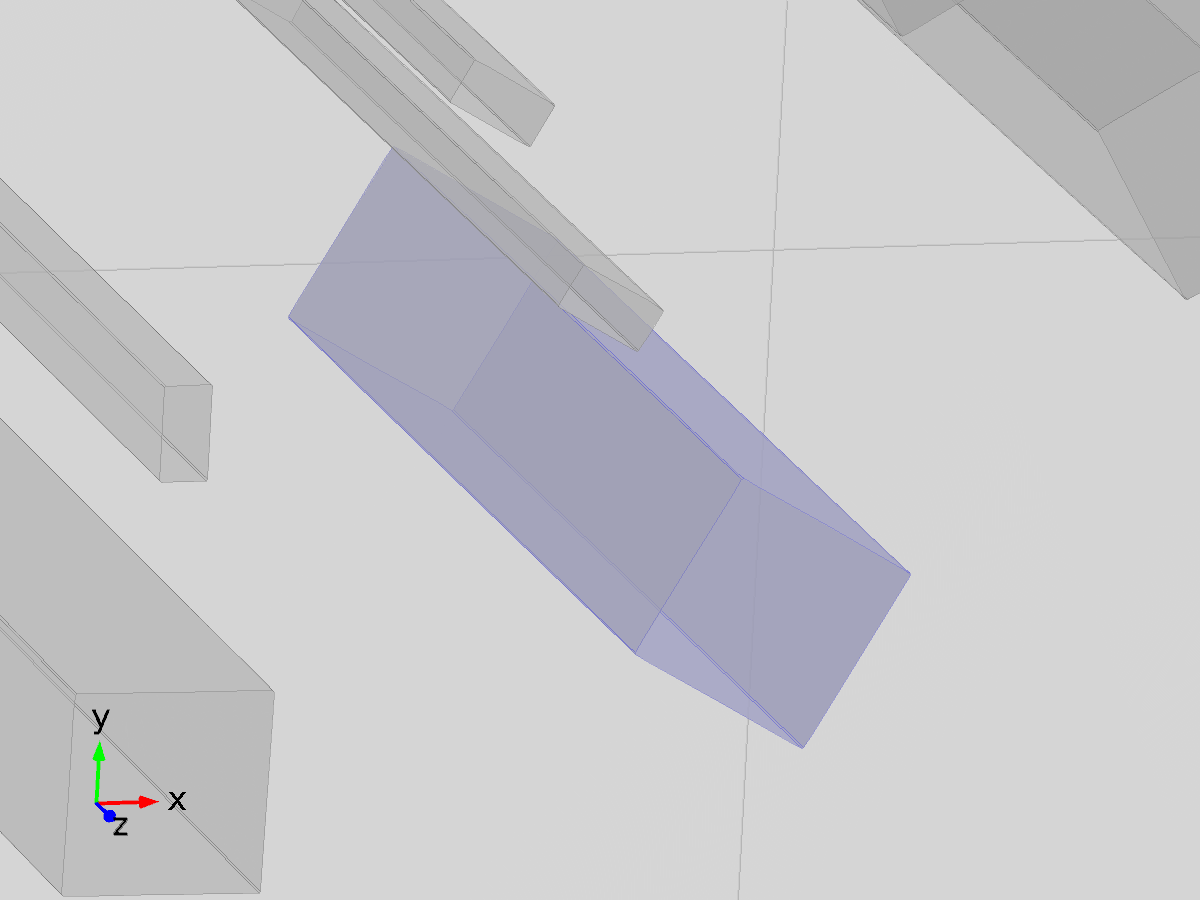

Supplement: S2 File — Model documentation generated by COMSOL with implemented parameters for the manual prototype. (ZIP) [file pone.0157040.s002.zip › SPMA_Rectangle_files/physics_mfnc_mfc38.png]

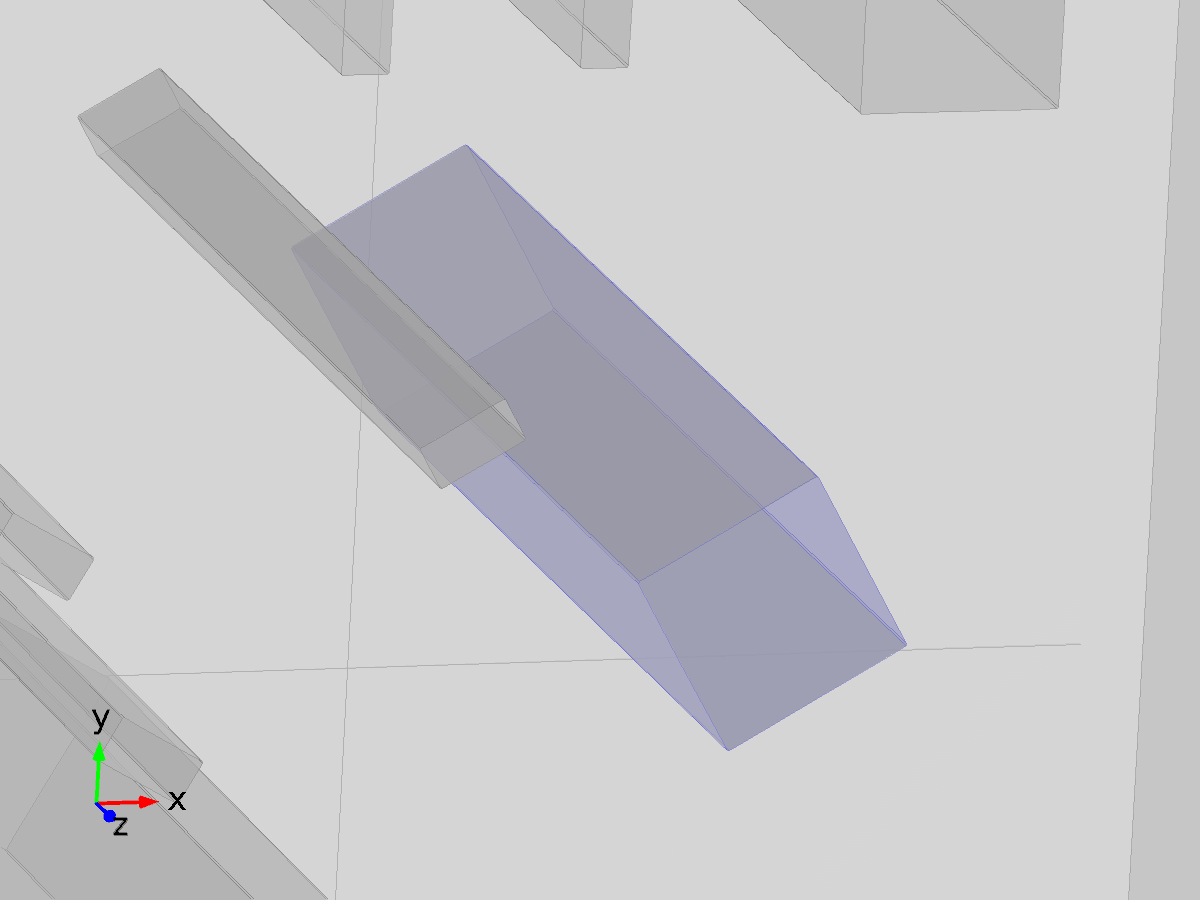

Supplement: S2 File — Model documentation generated by COMSOL with implemented parameters for the manual prototype. (ZIP) [file pone.0157040.s002.zip › SPMA_Rectangle_files/physics_mfnc_mfc39.png]

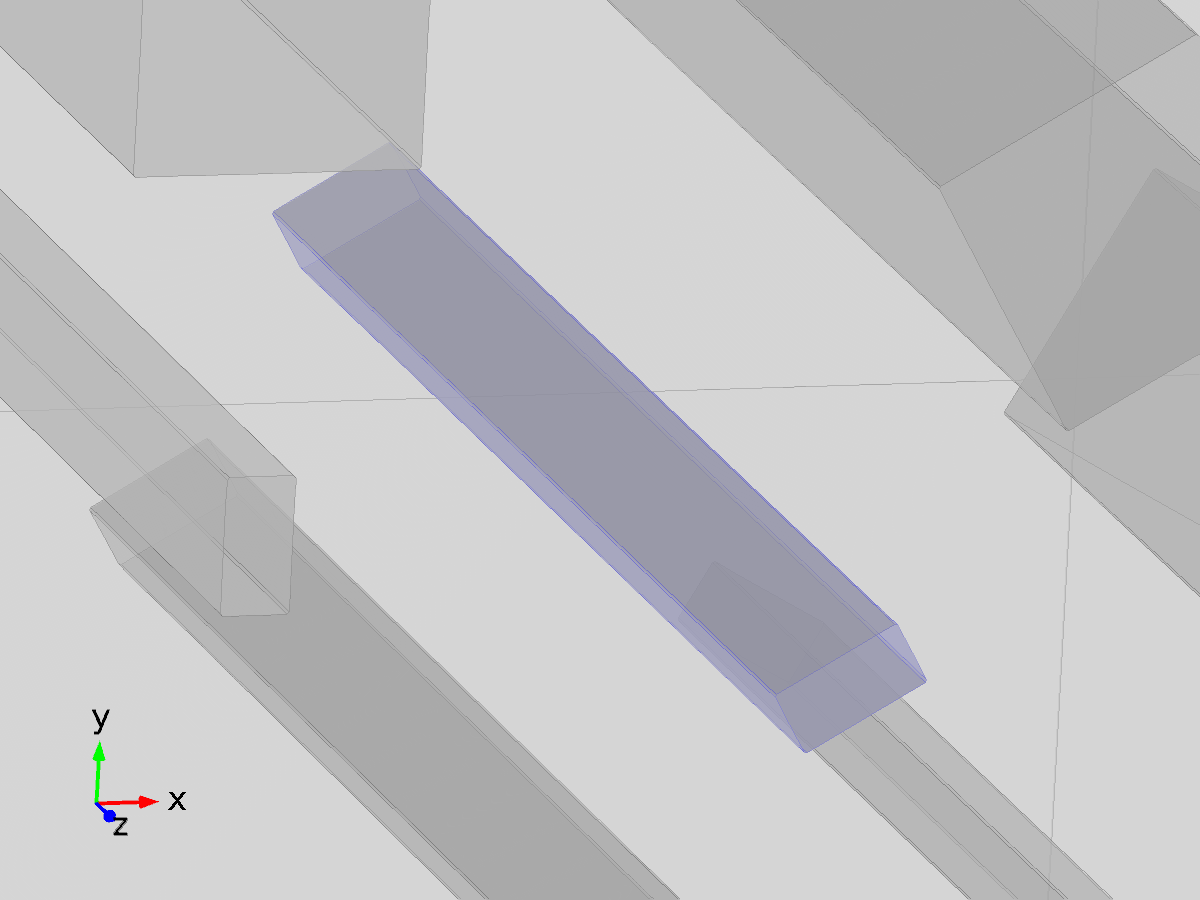

Supplement: S2 File — Model documentation generated by COMSOL with implemented parameters for the manual prototype. (ZIP) [file pone.0157040.s002.zip › SPMA_Rectangle_files/physics_mfnc_mfc4.png]

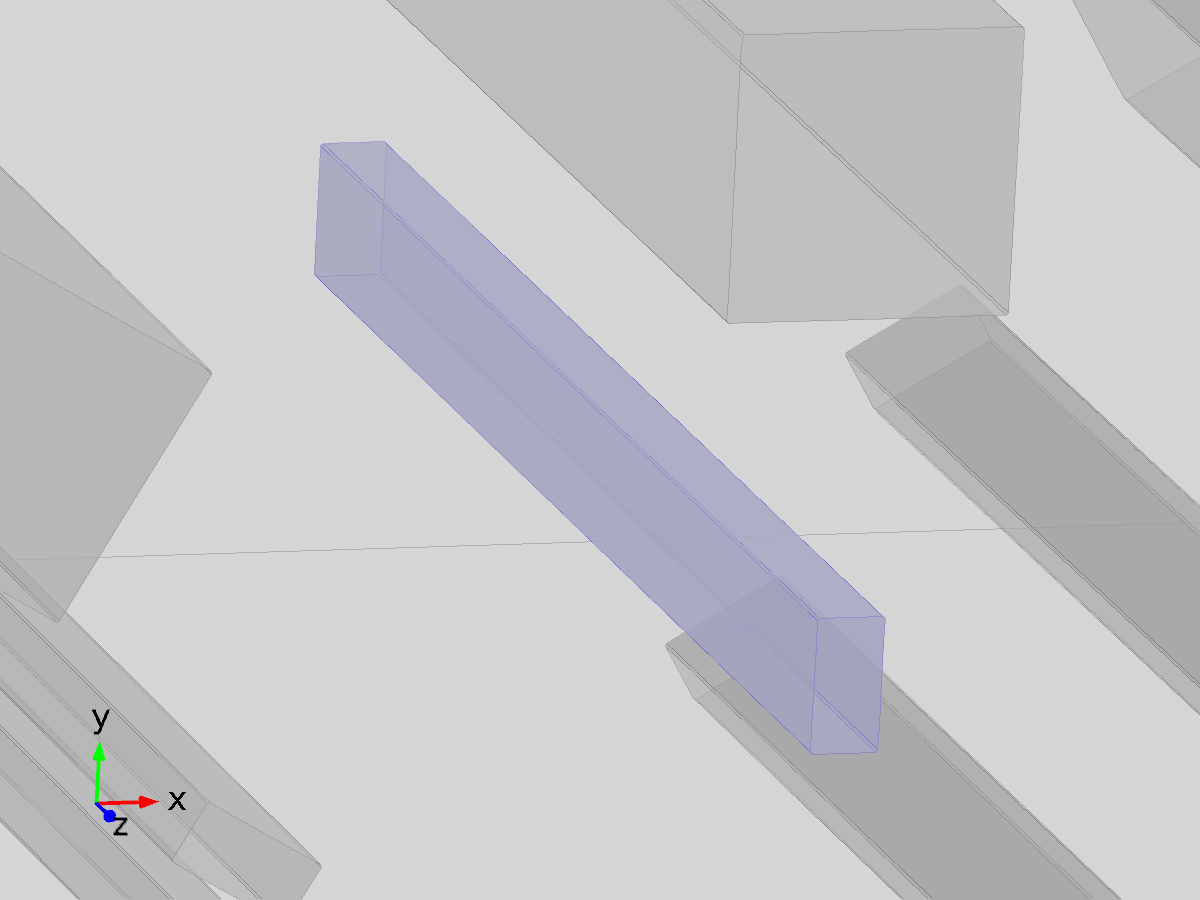

Supplement: S2 File — Model documentation generated by COMSOL with implemented parameters for the manual prototype. (ZIP) [file pone.0157040.s002.zip › SPMA_Rectangle_files/physics_mfnc_mfc5.png]

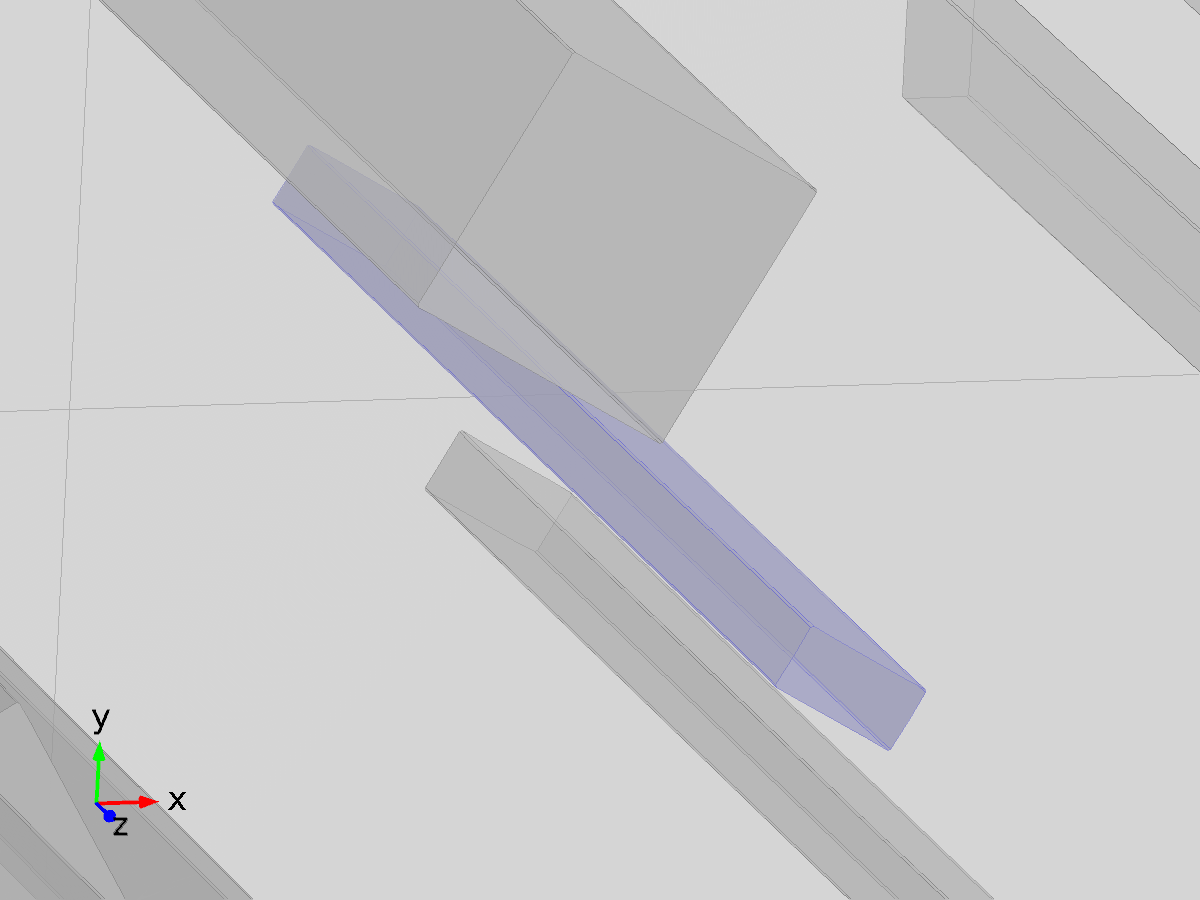

Supplement: S2 File — Model documentation generated by COMSOL with implemented parameters for the manual prototype. (ZIP) [file pone.0157040.s002.zip › SPMA_Rectangle_files/physics_mfnc_mfc6.png]

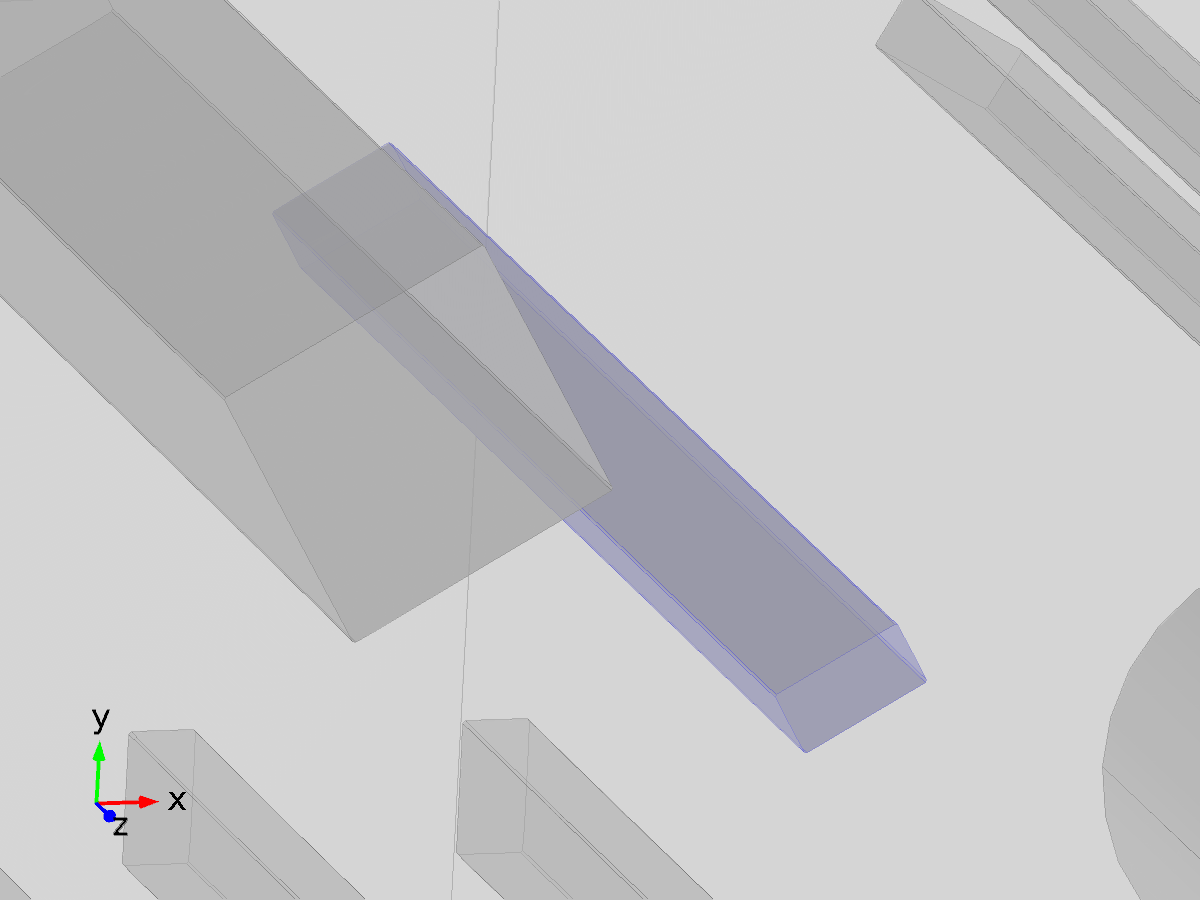

Supplement: S2 File — Model documentation generated by COMSOL with implemented parameters for the manual prototype. (ZIP) [file pone.0157040.s002.zip › SPMA_Rectangle_files/physics_mfnc_mfc7.png]

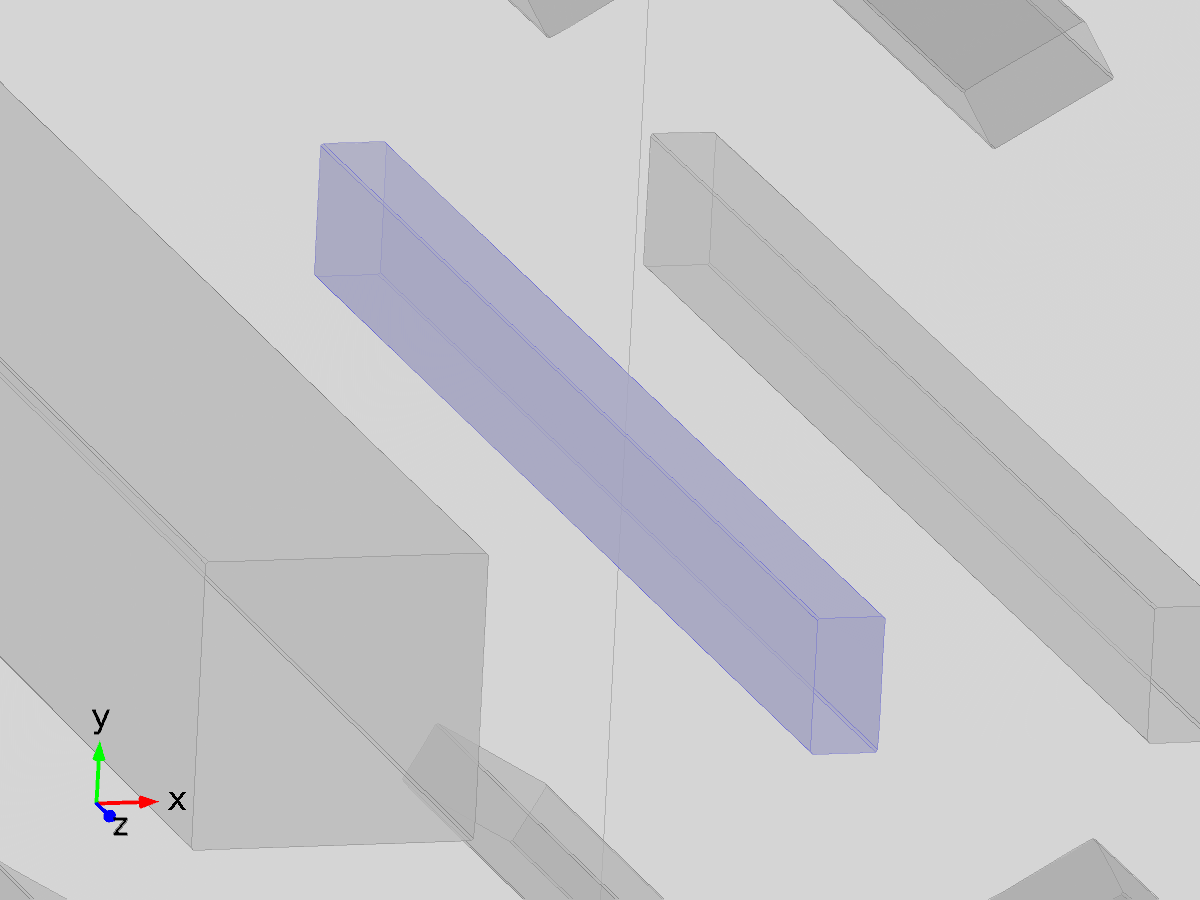

Supplement: S2 File — Model documentation generated by COMSOL with implemented parameters for the manual prototype. (ZIP) [file pone.0157040.s002.zip › SPMA_Rectangle_files/physics_mfnc_mfc8.png]

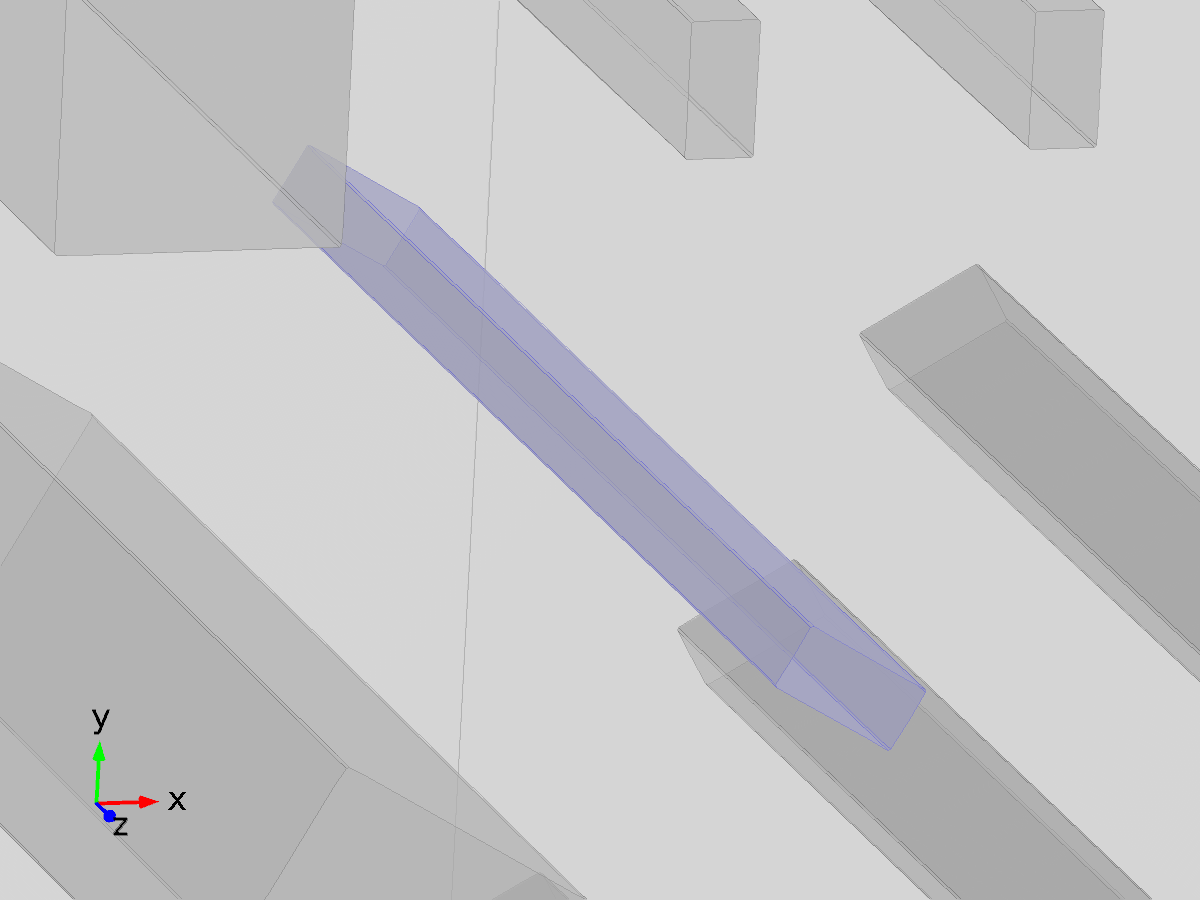

Supplement: S2 File — Model documentation generated by COMSOL with implemented parameters for the manual prototype. (ZIP) [file pone.0157040.s002.zip › SPMA_Rectangle_files/physics_mfnc_mfc9.png]
