# Supplementary material for: Rotatable Small Permanent Magnet Array for Ultra-Low Field Nuclear Magnetic Resonance Instrumentation: A Concept Study
Source: PLoS One. 2016 Jun 6;11(6):e0157040. doi: 10.1371/journal.pone.0157040 (PMC4894570; doi:10.1371/journal.pone.0157040)
Supplement: S2 File — Model documentation generated by COMSOL with implemented parameters for the manual prototype. (ZIP) [file pone.0157040.s002.zip › SPMA_Rectangle.html]

xml version="1.0" encoding="utf-8"?


SPMA\_Rectangle


HalbachRectangular De

|  |  |
| --- | --- |
| Date | Mar 3, 2016 1:02:40 PM |

Contents

|  |  |  |  |  |  |  |
| --- | --- | --- | --- | --- | --- | --- |
| 1. |  | Global | | | | |
| 1.1. |  |  | Definitions | | | |
| 2. |  | Component 1 | | | | |
| 2.1. |  |  | Definitions | | | |
| 2.2. |  |  | Geometry 1 | | | |
| 2.3. |  |  | Materials | | | |
| 2.4. |  |  | Magnetic Fields, No Currents | | | |
| 2.5. |  |  | Mesh 1 | | | |
| 3. |  | Study 1 | | | | |
| 3.1. |  |  | Stationary | | | |
| 3.2. |  |  | Solver Configurations | | | |
| 4. |  | Results | | | | |
| 4.1. |  |  | Data Sets | | | |
| 4.2. |  |  | Tables | | | |
| 4.3. |  |  | Plot Groups | | | |

## 1. Global

|  |  |
| --- | --- |
| Date | Dec 2, 2014 3:24:49 PM |

Global settings

| Name | HalbachRectangular\_de.mph |
| Path | C:\2\_UQ folder\1\_Project\Journal Paper 2 Halbach Array\SPMA Rectangular Magnets\HalbachRectangular\_de.mph |
| Program | COMSOL 5.0 (Build: 244) |
| Unit system | SI |

Used products

| COMSOL Multiphysics |
| AC/DC Module |

### 1.1. Definitions

#### 1.1.1. Parameters 1

Parameters

| Name | Expression | Value | Description |
| RC | 0.075[m] | 0.075000 m |  |
| RB | 0.105[m] | 0.10500 m |  |
| RA | 0.15[m] | 0.15000 m |  |
| BC | 0.39[T] | 0.39000 T |  |
| BB | -0.39[T] | -0.39000 T |  |
| corr\_c | 0 | 0.0000 |  |
| corr\_b | 0 | 0.0000 |  |
| BA | 0.39[T] | 0.39000 T |  |

## 2. Component 1

|  |  |
| --- | --- |
| Date | Dec 2, 2014 3:24:49 PM |

Component settings

| Unit system | SI |
| Geometry shape order | automatic |

### 2.1. Definitions

#### 2.1.1. Coordinate Systems

##### Boundary System 1

|  |  |
| --- | --- |
| Coordinate system type | Boundary system |
| Tag | sys1 |

Settings

| First (t1) | Second (t2) | Third (n) |
| t1 | t2 | n |

Settings

| Name | Value |
| Create first tangent direction from | Global Cartesian |

### 2.2. Geometry 1

Geometry 1

Units

| Length unit | m |
| Angular unit | deg |

Geometry statistics

| Property | Value |
| Space dimension | 3 |
| Number of domains | 32 |
| Number of boundaries | 312 |
| Number of edges | 744 |
| Number of vertices | 496 |

#### 2.2.1. Work Plane 1 (Wp1)

Unite objects

| Name | Value |
| Unite objects | On |

##### Plane Geometry (Wp1)

Plane Geometry

###### ArrayCMagnet1 (R1)

Position

| Name | Value |
| Position | {RC, 0} |
| Base | Center |

Rotation angle

| Name | Value |
| Rotation | 90 |

Size

| Name | Value |
| Width | 0.012 |
| Height | 0.006 |

###### ArrayCMagnet3 (R3)

Position

| Name | Value |
| Position | {RC\*cos(2\*pi\*60/360), RC\*sin(2\*pi\*60/360)} |
| Base | Center |

Rotation angle

| Name | Value |
| Rotation | 210 |

Size

| Name | Value |
| Width | 0.012 |
| Height | 0.006 |

###### ArrayCMagnet5 (R5)

Position

| Name | Value |
| Position | {RC\*cos(2\*pi\*120/360), RC\*sin(2\*pi\*120/360)} |
| Base | Center |

Rotation angle

| Name | Value |
| Rotation | 330 |

Size

| Name | Value |
| Width | 0.012 |
| Height | 0.006 |

###### ArrayCMagnet7 (R7)

Position

| Name | Value |
| Position | {RC\*cos(2\*pi\*180/360), RC\*sin(2\*pi\*180/360)} |
| Base | Center |

Rotation angle

| Name | Value |
| Rotation | 450 |

Size

| Name | Value |
| Width | 0.012 |
| Height | 0.006 |

###### ArrayCMagnet9 (R9)

Position

| Name | Value |
| Position | {RC\*cos(2\*pi\*240/360), RC\*sin(2\*pi\*240/360)} |
| Base | Center |

Rotation angle

| Name | Value |
| Rotation | 570 |

Size

| Name | Value |
| Width | 0.012 |
| Height | 0.006 |

###### ArrayCMagnet11 (R11)

Position

| Name | Value |
| Position | {RC\*cos(2\*pi\*300/360), RC\*sin(2\*pi\*300/360)} |
| Base | Center |

Rotation angle

| Name | Value |
| Rotation | 690 |

Size

| Name | Value |
| Width | 0.012 |
| Height | 0.006 |

###### Rotate 1 (Rot1)

Settings

| Name | Value |
| Rotation | 0 |
| Point on axis of rotation | {0, 0} |

###### ArrayBMagnet1 (R13)

Position

| Name | Value |
| Position | {RB, 0} |
| Base | Center |

Rotation angle

| Name | Value |
| Rotation | 90 |

Size

| Name | Value |
| Width | 0.012 |
| Height | 0.006 |

###### ArrayBMagnet2 (R14)

Position

| Name | Value |
| Position | {RB\*cos(2\*pi\*30/360), RB\*sin(2\*pi\*30/360)} |
| Base | Center |

Rotation angle

| Name | Value |
| Rotation | 150 |

Size

| Name | Value |
| Width | 0.012 |
| Height | 0.006 |

###### ArrayBMagnet3 (R15)

Position

| Name | Value |
| Position | {RB\*cos(2\*pi\*60/360), RB\*sin(2\*pi\*60/360)} |
| Base | Center |

Rotation angle

| Name | Value |
| Rotation | 210 |

Size

| Name | Value |
| Width | 0.012 |
| Height | 0.006 |

###### ArrayBMagnet4 (R16)

Position

| Name | Value |
| Position | {RB\*cos(2\*pi\*90/360), RB\*sin(2\*pi\*90/360)} |
| Base | Center |

Rotation angle

| Name | Value |
| Rotation | 270 |

Size

| Name | Value |
| Width | 0.012 |
| Height | 0.006 |

###### ArrayBMagnet5 (R17)

Position

| Name | Value |
| Position | {RB\*cos(2\*pi\*120/360), RB\*sin(2\*pi\*120/360)} |
| Base | Center |

Rotation angle

| Name | Value |
| Rotation | 330 |

Size

| Name | Value |
| Width | 0.012 |
| Height | 0.006 |

###### ArrayBMagnet6 (R18)

Position

| Name | Value |
| Position | {RB\*cos(2\*pi\*150/360), RB\*sin(2\*pi\*150/360)} |
| Base | Center |

Rotation angle

| Name | Value |
| Rotation | 390 |

Size

| Name | Value |
| Width | 0.012 |
| Height | 0.006 |

###### ArrayBMagnet7 (R19)

Position

| Name | Value |
| Position | {RB\*cos(2\*pi\*180/360), RB\*sin(2\*pi\*180/360)} |
| Base | Center |

Rotation angle

| Name | Value |
| Rotation | 450 |

Size

| Name | Value |
| Width | 0.012 |
| Height | 0.006 |

###### ArrayBMagnet8 (R20)

Position

| Name | Value |
| Position | {RB\*cos(2\*pi\*210/360), RB\*sin(2\*pi\*210/360)} |
| Base | Center |

Rotation angle

| Name | Value |
| Rotation | 510 |

Size

| Name | Value |
| Width | 0.012 |
| Height | 0.006 |

###### ArrayBMagnet9 (R21)

Position

| Name | Value |
| Position | {RB\*cos(2\*pi\*240/360), RB\*sin(2\*pi\*240/360)} |
| Base | Center |

Rotation angle

| Name | Value |
| Rotation | 570 |

Size

| Name | Value |
| Width | 0.012 |
| Height | 0.006 |

###### ArrayBMagnet10 (R22)

Position

| Name | Value |
| Position | {RB\*cos(2\*pi\*270/360), RB\*sin(2\*pi\*270/360)} |
| Base | Center |

Rotation angle

| Name | Value |
| Rotation | 630 |

Size

| Name | Value |
| Width | 0.012 |
| Height | 0.006 |

###### ArrayBMagnet11 (R23)

Position

| Name | Value |
| Position | {RB\*cos(2\*pi\*300/360), RB\*sin(2\*pi\*300/360)} |
| Base | Center |

Rotation angle

| Name | Value |
| Rotation | 690 |

Size

| Name | Value |
| Width | 0.012 |
| Height | 0.006 |

###### ArrayBMagnet12 (R24)

Position

| Name | Value |
| Position | {RB\*cos(2\*pi\*330/360), RB\*sin(2\*pi\*330/360)} |
| Base | Center |

Rotation angle

| Name | Value |
| Rotation | 750 |

Size

| Name | Value |
| Width | 0.012 |
| Height | 0.006 |

###### Rotate 2 (Rot2)

Settings

| Name | Value |
| Rotation | 0 |
| Point on axis of rotation | {0, 0} |

###### Fillet 1 (Fil1)

Settings

| Name | Value |
| Radius | 0.0002 |

###### ArrayAMagnet1 (R25)

Position

| Name | Value |
| Position | {RA, 0} |
| Base | Center |

Rotation angle

| Name | Value |
| Rotation | 90 |

Size

| Name | Value |
| Width | 0.0254 |
| Height | 0.025 |

###### ArrayAMagnet2 (R26)

Position

| Name | Value |
| Position | {RA\*cos(2\*pi\*30/360), RA\*sin(2\*pi\*30/360)} |
| Base | Center |

Rotation angle

| Name | Value |
| Rotation | 150 |

Size

| Name | Value |
| Width | 0.0254 |
| Height | 0.025 |

###### ArrayAMagnet3 (R27)

Position

| Name | Value |
| Position | {RA\*cos(2\*pi\*60/360), RA\*sin(2\*pi\*60/360)} |
| Base | Center |

Rotation angle

| Name | Value |
| Rotation | 210 |

Size

| Name | Value |
| Width | 0.0254 |
| Height | 0.025 |

###### ArrayAMagnet4 (R28)

Position

| Name | Value |
| Position | {RA\*cos(2\*pi\*90/360), RA\*sin(2\*pi\*90/360)} |
| Base | Center |

Rotation angle

| Name | Value |
| Rotation | 270 |

Size

| Name | Value |
| Width | 0.0254 |
| Height | 0.025 |

###### ArrayAMagnet5 (R29)

Position

| Name | Value |
| Position | {RA\*cos(2\*pi\*120/360), RA\*sin(2\*pi\*120/360)} |
| Base | Center |

Rotation angle

| Name | Value |
| Rotation | 330 |

Size

| Name | Value |
| Width | 0.0254 |
| Height | 0.025 |

###### ArrayAMagnet6 (R30)

Position

| Name | Value |
| Position | {RA\*cos(2\*pi\*150/360), RA\*sin(2\*pi\*150/360)} |
| Base | Center |

Rotation angle

| Name | Value |
| Rotation | 390 |

Size

| Name | Value |
| Width | 0.0254 |
| Height | 0.025 |

###### ArrayAMagnet7 (R31)

Position

| Name | Value |
| Position | {RA\*cos(2\*pi\*180/360), RA\*sin(2\*pi\*180/360)} |
| Base | Center |

Rotation angle

| Name | Value |
| Rotation | 450 |

Size

| Name | Value |
| Width | 0.0254 |
| Height | 0.025 |

###### ArrayAMagnet8 (R32)

Position

| Name | Value |
| Position | {RA\*cos(2\*pi\*210/360), RA\*sin(2\*pi\*210/360)} |
| Base | Center |

Rotation angle

| Name | Value |
| Rotation | 510 |

Size

| Name | Value |
| Width | 0.0254 |
| Height | 0.025 |

###### ArrayAMagnet9 (R33)

Position

| Name | Value |
| Position | {RA\*cos(2\*pi\*240/360), RA\*sin(2\*pi\*240/360)} |
| Base | Center |

Rotation angle

| Name | Value |
| Rotation | 570 |

Size

| Name | Value |
| Width | 0.0254 |
| Height | 0.025 |

###### ArrayAMagnet10 (R34)

Position

| Name | Value |
| Position | {RA\*cos(2\*pi\*270/360), RA\*sin(2\*pi\*270/360)} |
| Base | Center |

Rotation angle

| Name | Value |
| Rotation | 629 |

Size

| Name | Value |
| Width | 0.0254 |
| Height | 0.025 |

###### ArrayAMagnet11 (R35)

Position

| Name | Value |
| Position | {RA\*cos(2\*pi\*300/360), RA\*sin(2\*pi\*300/360)} |
| Base | Center |

Rotation angle

| Name | Value |
| Rotation | 690 |

Size

| Name | Value |
| Width | 0.0254 |
| Height | 0.025 |

###### ArrayAMagnet12 (R36)

Position

| Name | Value |
| Position | {RA\*cos(2\*pi\*330/360), RA\*sin(2\*pi\*330/360)} |
| Base | Center |

Rotation angle

| Name | Value |
| Rotation | 750 |

Size

| Name | Value |
| Width | 0.0254 |
| Height | 0.025 |

###### Fillet 2 (Fil2)

Settings

| Name | Value |
| Radius | 0.0002 |

###### Circle 1 (C1)

Position

| Name | Value |
| Position | {0, 0} |

Size and shape

| Name | Value |
| Radius | 0.02 |

#### 2.2.2. Extrude 1 (Ext1)

Settings

| Name | Value |
| Work plane | Work Plane 1 |

Distances from plane

| Name | Value |
| Distances | 0.15 |

Scales

| Scales xw | Scales yw |
| 1 | 1 |

Displacements

| Displacements xw (m) | Displacements yw (m) |
| 0 | 0 |

Twist angles

| Name | Value |
| Twist\_angles | 0 |

#### 2.2.3. Block 1 (Blk1)

Position

| Name | Value |
| Position | {0, 0, 0.075} |
| Base | Center |

Axis

| Name | Value |
| Axis type | z - axis |

Size and shape

| Name | Value |
| Width | 0.6 |
| Depth | 0.6 |
| Height | 0.6 |

### 2.3. Materials

#### 2.3.1. Soft Iron (Without Losses)

Soft Iron (without losses)

Selection

| Geometric entity level | Domain |
| Selection | Domains 2–14, 16–32 |

Material parameters

| Name | Value | Unit |
| Relative permeability | 1.05 | 1 |

Basic Settings

| Description | Value |
| Electrical conductivity | {{0.666e6[S/m], 0, 0}, {0, 0.666e6[S/m], 0}, {0, 0, 0.666e6[S/m]}} |
| Relative permittivity | {{1.05, 0, 0}, {0, 1.05, 0}, {0, 0, 1.05}} |
| Relative permeability | {{1.05, 0, 0}, {0, 1.05, 0}, {0, 0, 1.05}} |

BH curve Settings

| Description | Value |
| Magnetic flux density norm | BH(normH[m/A])[T] |
| normH | sqrt(H1^2 + H2^2 + H3^2) |

Functions

| Function name | Type |
| BH | Interpolation |

BH

HB curve Settings

| Description | Value |
| Magnetic field norm | HB(normB[1/T])[A/m] |
| normB | sqrt(B1^2 + B2^2 + B3^2) |

Functions

| Function name | Type |
| HB | Interpolation |

HB

#### 2.3.2. Air

Air

Selection

| Geometric entity level | Domain |
| Selection | Domains 1, 15 |

Material parameters

| Name | Value | Unit |
| Relative permeability | 1 | 1 |

Basic Settings

| Description | Value |
| Relative permeability | {{1, 0, 0}, {0, 1, 0}, {0, 0, 1}} |
| Relative permittivity | {{1, 0, 0}, {0, 1, 0}, {0, 0, 1}} |
| Dynamic viscosity | eta(T[1/K])[Pa\*s] |
| Ratio of specific heats | 1.4 |
| Electrical conductivity | {{0[S/m], 0, 0}, {0, 0[S/m], 0}, {0, 0, 0[S/m]}} |
| Heat capacity at constant pressure | Cp(T[1/K])[J/(kg\*K)] |
| Density | rho(pA[1/Pa], T[1/K])[kg/m^3] |
| Thermal conductivity | {{k(T[1/K])[W/(m\*K)], 0, 0}, {0, k(T[1/K])[W/(m\*K)], 0}, {0, 0, k(T[1/K])[W/(m\*K)]}} |
| Speed of sound | cs(T[1/K])[m/s] |

Functions

| Function name | Type |
| eta | Piecewise |
| Cp | Piecewise |
| rho | Analytic |
| k | Piecewise |
| cs | Analytic |

eta

Cp

k

cs

Refractive index Settings

| Description | Value |
| Refractive index | {{1, 0, 0}, {0, 1, 0}, {0, 0, 1}} |
| Refractive index, imaginary part | {{0, 0, 0}, {0, 0, 0}, {0, 0, 0}} |

### 2.4. Magnetic Fields, No Currents

Magnetic Fields, No Currents

Selection

| Geometric entity level | Domain |
| Selection | Domains 1–32 |

Equations

Settings

| Description | Value |
| Magnetic scalar potential | Quadratic |
| Value type when using splitting of complex variables | Complex |

Used products

| COMSOL Multiphysics |
| AC/DC Module |

Variables

| Name | Expression | Unit | Description | Selection |
| mfnc.Bbx | 0 | T | Background magnetic flux density, x component | Domains 1–32 |
| mfnc.Bby | 0 | T | Background magnetic flux density, y component | Domains 1–32 |
| mfnc.Bbz | 0 | T | Background magnetic flux density, z component | Domains 1–32 |
| mfnc.Hbx | 0 | A/m | Background magnetic field, x component | Domains 1–32 |
| mfnc.Hby | 0 | A/m | Background magnetic field, y component | Domains 1–32 |
| mfnc.Hbz | 0 | A/m | Background magnetic field, z component | Domains 1–32 |
| mfnc.nx | nx |  | Normal vector, x component | Boundaries 6–311 |
| mfnc.ny | ny |  | Normal vector, y component | Boundaries 6–311 |
| mfnc.nz | nz |  | Normal vector, z component | Boundaries 6–311 |
| mfnc.nx | dnx |  | Normal vector, x component | Boundaries 1–5, 312 |
| mfnc.ny | dny |  | Normal vector, y component | Boundaries 1–5, 312 |
| mfnc.nz | dnz |  | Normal vector, z component | Boundaries 1–5, 312 |
| mfnc.nmeshx | nxmesh |  | Mesh normal vector, x component | Boundaries 6–311 |
| mfnc.nmeshy | nymesh |  | Mesh normal vector, y component | Boundaries 6–311 |
| mfnc.nmeshz | nzmesh |  | Mesh normal vector, z component | Boundaries 6–311 |
| mfnc.nmeshx | dnxmesh |  | Mesh normal vector, x component | Boundaries 1–5, 312 |
| mfnc.nmeshy | dnymesh |  | Mesh normal vector, y component | Boundaries 1–5, 312 |
| mfnc.nmeshz | dnzmesh |  | Mesh normal vector, z component | Boundaries 1–5, 312 |
| mfnc.unmeshx | unxmesh |  | Mesh normal vector, upside, x component | Boundaries 6–311 |
| mfnc.unmeshy | unymesh |  | Mesh normal vector, upside, y component | Boundaries 6–311 |
| mfnc.unmeshz | unzmesh |  | Mesh normal vector, upside, z component | Boundaries 6–311 |
| mfnc.dnmeshx | dnxmesh |  | Mesh normal vector, downside, x component | Boundaries 6–311 |
| mfnc.dnmeshy | dnymesh |  | Mesh normal vector, downside, y component | Boundaries 6–311 |
| mfnc.dnmeshz | dnzmesh |  | Mesh normal vector, downside, z component | Boundaries 6–311 |
| mfnc.intWm | mfnc.int\_Wm(mfnc.d\*mfnc.dWm) | J | Total magnetic energy | Global |
| mfnc.d | 1 | 1 | Contribution | Domains 1–32 |
| mfnc.unTmx | 0 | Pa | Maxwell upward magnetic surface stress tensor, x component | Boundaries 1–312 |
| mfnc.unTmy | 0 | Pa | Maxwell upward magnetic surface stress tensor, y component | Boundaries 1–312 |
| mfnc.unTmz | 0 | Pa | Maxwell upward magnetic surface stress tensor, z component | Boundaries 1–312 |
| mfnc.dnTmx | 0 | Pa | Maxwell downward magnetic surface stress tensor, x component | Boundaries 1–312 |
| mfnc.dnTmy | 0 | Pa | Maxwell downward magnetic surface stress tensor, y component | Boundaries 1–312 |
| mfnc.dnTmz | 0 | Pa | Maxwell downward magnetic surface stress tensor, z component | Boundaries 1–312 |

#### 2.4.1. Magnetic Flux Conservation 1

Magnetic Flux Conservation 1

Selection

| Geometric entity level | Domain |
| Selection | Domains 1, 15 |

Equations

Settings

| Description | Value |
| Constitutive relation | Relative permeability |
| Relative permeability | From material |
| Relative permeability | {{1, 0, 0}, {0, 1, 0}, {0, 0, 1}} |

Properties from material

| Property | Material | Property group |
| Relative permeability | Air | Basic |

##### Variables

| Name | Expression | Unit | Description | Selection |
| --- | --- | --- | --- | --- |
| mfnc.unTmx | -0.5\*mfnc.dnx\*(real(up(mfnc.Bx))\*real(up(mfnc.Hx))+real(up(mfnc.By))\*real(up(mfnc.Hy))+real(up(mfnc.Bz))\*real(up(mfnc.Hz)))+real(up(mfnc.Bx))\*(real(up(mfnc.Hx))\*mfnc.dnx+real(up(mfnc.Hy))\*mfnc.dny+real(up(mfnc.Hz))\*mfnc.dnz) | Pa | Maxwell upward magnetic surface stress tensor, x component | Boundaries 136–139, 168–169 |
| mfnc.unTmy | -0.5\*mfnc.dny\*(real(up(mfnc.Bx))\*real(up(mfnc.Hx))+real(up(mfnc.By))\*real(up(mfnc.Hy))+real(up(mfnc.Bz))\*real(up(mfnc.Hz)))+real(up(mfnc.By))\*(real(up(mfnc.Hx))\*mfnc.dnx+real(up(mfnc.Hy))\*mfnc.dny+real(up(mfnc.Hz))\*mfnc.dnz) | Pa | Maxwell upward magnetic surface stress tensor, y component | Boundaries 136–139, 168–169 |
| mfnc.unTmz | -0.5\*mfnc.dnz\*(real(up(mfnc.Bx))\*real(up(mfnc.Hx))+real(up(mfnc.By))\*real(up(mfnc.Hy))+real(up(mfnc.Bz))\*real(up(mfnc.Hz)))+real(up(mfnc.Bz))\*(real(up(mfnc.Hx))\*mfnc.dnx+real(up(mfnc.Hy))\*mfnc.dny+real(up(mfnc.Hz))\*mfnc.dnz) | Pa | Maxwell upward magnetic surface stress tensor, z component | Boundaries 136–139, 168–169 |
| mfnc.unTmx | -0.5\*mfnc.dnx\*(real(up(mfnc.Bx))\*real(up(mfnc.Hx))+real(up(mfnc.By))\*real(up(mfnc.Hy))+real(up(mfnc.Bz))\*real(up(mfnc.Hz)))+real(up(mfnc.Bx))\*(real(up(mfnc.Hx))\*mfnc.dnx+real(up(mfnc.Hy))\*mfnc.dny+real(up(mfnc.Hz))\*mfnc.dnz) | Pa | Maxwell upward magnetic surface stress tensor, x component | Boundaries 6–135, 140–167, 170–311 |
| mfnc.unTmy | -0.5\*mfnc.dny\*(real(up(mfnc.Bx))\*real(up(mfnc.Hx))+real(up(mfnc.By))\*real(up(mfnc.Hy))+real(up(mfnc.Bz))\*real(up(mfnc.Hz)))+real(up(mfnc.By))\*(real(up(mfnc.Hx))\*mfnc.dnx+real(up(mfnc.Hy))\*mfnc.dny+real(up(mfnc.Hz))\*mfnc.dnz) | Pa | Maxwell upward magnetic surface stress tensor, y component | Boundaries 6–135, 140–167, 170–311 |
| mfnc.unTmz | -0.5\*mfnc.dnz\*(real(up(mfnc.Bx))\*real(up(mfnc.Hx))+real(up(mfnc.By))\*real(up(mfnc.Hy))+real(up(mfnc.Bz))\*real(up(mfnc.Hz)))+real(up(mfnc.Bz))\*(real(up(mfnc.Hx))\*mfnc.dnx+real(up(mfnc.Hy))\*mfnc.dny+real(up(mfnc.Hz))\*mfnc.dnz) | Pa | Maxwell upward magnetic surface stress tensor, z component | Boundaries 6–135, 140–167, 170–311 |
| mfnc.dnTmx | -0.5\*mfnc.unx\*(real(down(mfnc.Bx))\*real(down(mfnc.Hx))+real(down(mfnc.By))\*real(down(mfnc.Hy))+real(down(mfnc.Bz))\*real(down(mfnc.Hz)))+real(down(mfnc.Bx))\*(real(down(mfnc.Hx))\*mfnc.unx+real(down(mfnc.Hy))\*mfnc.uny+real(down(mfnc.Hz))\*mfnc.unz) | Pa | Maxwell downward magnetic surface stress tensor, x component | Boundaries 136–139, 168–169 |
| mfnc.dnTmy | -0.5\*mfnc.uny\*(real(down(mfnc.Bx))\*real(down(mfnc.Hx))+real(down(mfnc.By))\*real(down(mfnc.Hy))+real(down(mfnc.Bz))\*real(down(mfnc.Hz)))+real(down(mfnc.By))\*(real(down(mfnc.Hx))\*mfnc.unx+real(down(mfnc.Hy))\*mfnc.uny+real(down(mfnc.Hz))\*mfnc.unz) | Pa | Maxwell downward magnetic surface stress tensor, y component | Boundaries 136–139, 168–169 |
| mfnc.dnTmz | -0.5\*mfnc.unz\*(real(down(mfnc.Bx))\*real(down(mfnc.Hx))+real(down(mfnc.By))\*real(down(mfnc.Hy))+real(down(mfnc.Bz))\*real(down(mfnc.Hz)))+real(down(mfnc.Bz))\*(real(down(mfnc.Hx))\*mfnc.unx+real(down(mfnc.Hy))\*mfnc.uny+real(down(mfnc.Hz))\*mfnc.unz) | Pa | Maxwell downward magnetic surface stress tensor, z component | Boundaries 136–139, 168–169 |
| mfnc.dnTmx | -0.5\*mfnc.unx\*(real(down(mfnc.Bx))\*real(down(mfnc.Hx))+real(down(mfnc.By))\*real(down(mfnc.Hy))+real(down(mfnc.Bz))\*real(down(mfnc.Hz)))+real(down(mfnc.Bx))\*(real(down(mfnc.Hx))\*mfnc.unx+real(down(mfnc.Hy))\*mfnc.uny+real(down(mfnc.Hz))\*mfnc.unz) | Pa | Maxwell downward magnetic surface stress tensor, x component | Boundaries 1–5, 312 |
| mfnc.dnTmy | -0.5\*mfnc.uny\*(real(down(mfnc.Bx))\*real(down(mfnc.Hx))+real(down(mfnc.By))\*real(down(mfnc.Hy))+real(down(mfnc.Bz))\*real(down(mfnc.Hz)))+real(down(mfnc.By))\*(real(down(mfnc.Hx))\*mfnc.unx+real(down(mfnc.Hy))\*mfnc.uny+real(down(mfnc.Hz))\*mfnc.unz) | Pa | Maxwell downward magnetic surface stress tensor, y component | Boundaries 1–5, 312 |
| mfnc.dnTmz | -0.5\*mfnc.unz\*(real(down(mfnc.Bx))\*real(down(mfnc.Hx))+real(down(mfnc.By))\*real(down(mfnc.Hy))+real(down(mfnc.Bz))\*real(down(mfnc.Hz)))+real(down(mfnc.Bz))\*(real(down(mfnc.Hx))\*mfnc.unx+real(down(mfnc.Hy))\*mfnc.uny+real(down(mfnc.Hz))\*mfnc.unz) | Pa | Maxwell downward magnetic surface stress tensor, z component | Boundaries 1–5, 312 |
| mfnc.Hx | -Vmx | A/m | Magnetic field, x component | Domains 1, 15 |
| mfnc.Hy | -Vmy | A/m | Magnetic field, y component | Domains 1, 15 |
| mfnc.Hz | -Vmz | A/m | Magnetic field, z component | Domains 1, 15 |
| mfnc.tHx | -VmTx | A/m | Tangential magnetic field, x component | Boundaries 1–312 |
| mfnc.tHy | -VmTy | A/m | Tangential magnetic field, y component | Boundaries 1–312 |
| mfnc.tHz | -VmTz | A/m | Tangential magnetic field, z component | Boundaries 1–312 |
| mfnc.normH | sqrt(realdot(mfnc.Hx,mfnc.Hx)+realdot(mfnc.Hy,mfnc.Hy)+realdot(mfnc.Hz,mfnc.Hz)) | A/m | Magnetic field norm | Domains 1, 15 |
| mfnc.murxx | model.input.mur11 | 1 | Relative permeability, xx component | Domains 1, 15 |
| mfnc.muryx | model.input.mur21 | 1 | Relative permeability, yx component | Domains 1, 15 |
| mfnc.murzx | model.input.mur31 | 1 | Relative permeability, zx component | Domains 1, 15 |
| mfnc.murxy | model.input.mur12 | 1 | Relative permeability, xy component | Domains 1, 15 |
| mfnc.muryy | model.input.mur22 | 1 | Relative permeability, yy component | Domains 1, 15 |
| mfnc.murzy | model.input.mur32 | 1 | Relative permeability, zy component | Domains 1, 15 |
| mfnc.murxz | model.input.mur13 | 1 | Relative permeability, xz component | Domains 1, 15 |
| mfnc.muryz | model.input.mur23 | 1 | Relative permeability, yz component | Domains 1, 15 |
| mfnc.murzz | model.input.mur33 | 1 | Relative permeability, zz component | Domains 1, 15 |
| mfnc.Bx | mu0\_const\*(mfnc.Ixx\*mfnc.Hx+mfnc.Ixy\*mfnc.Hy+mfnc.Ixz\*mfnc.Hz+mfnc.Mx) | T | Magnetic flux density, x component | Domains 1, 15 |
| mfnc.By | mu0\_const\*(mfnc.Iyx\*mfnc.Hx+mfnc.Iyy\*mfnc.Hy+mfnc.Iyz\*mfnc.Hz+mfnc.My) | T | Magnetic flux density, y component | Domains 1, 15 |
| mfnc.Bz | mu0\_const\*(mfnc.Izx\*mfnc.Hx+mfnc.Izy\*mfnc.Hy+mfnc.Izz\*mfnc.Hz+mfnc.Mz) | T | Magnetic flux density, z component | Domains 1, 15 |
| mfnc.normB | sqrt(realdot(mfnc.Bx,mfnc.Bx)+realdot(mfnc.By,mfnc.By)+realdot(mfnc.Bz,mfnc.Bz)) | T | Magnetic flux density norm | Domains 1, 15 |
| mfnc.Mx | mfnc.chimxx\*mfnc.Hx+mfnc.chimxy\*mfnc.Hy+mfnc.chimxz\*mfnc.Hz | A/m | Magnetization, x component | Domains 1, 15 |
| mfnc.My | mfnc.chimyx\*mfnc.Hx+mfnc.chimyy\*mfnc.Hy+mfnc.chimyz\*mfnc.Hz | A/m | Magnetization, y component | Domains 1, 15 |
| mfnc.Mz | mfnc.chimzx\*mfnc.Hx+mfnc.chimzy\*mfnc.Hy+mfnc.chimzz\*mfnc.Hz | A/m | Magnetization, z component | Domains 1, 15 |
| mfnc.normM | sqrt(realdot(mfnc.Mx,mfnc.Mx)+realdot(mfnc.My,mfnc.My)+realdot(mfnc.Mz,mfnc.Mz)) | A/m | Magnetization norm | Domains 1, 15 |
| mfnc.Ixx | 1 | 1 | Spatial identity matrix, xx component | Domains 1, 15 |
| mfnc.Iyx | 0 | 1 | Spatial identity matrix, yx component | Domains 1, 15 |
| mfnc.Izx | 0 | 1 | Spatial identity matrix, zx component | Domains 1, 15 |
| mfnc.Ixy | 0 | 1 | Spatial identity matrix, xy component | Domains 1, 15 |
| mfnc.Iyy | 1 | 1 | Spatial identity matrix, yy component | Domains 1, 15 |
| mfnc.Izy | 0 | 1 | Spatial identity matrix, zy component | Domains 1, 15 |
| mfnc.Ixz | 0 | 1 | Spatial identity matrix, xz component | Domains 1, 15 |
| mfnc.Iyz | 0 | 1 | Spatial identity matrix, yz component | Domains 1, 15 |
| mfnc.Izz | 1 | 1 | Spatial identity matrix, zz component | Domains 1, 15 |
| mfnc.chimxx | -1+mfnc.murxx | 1 | Magnetic susceptibility, xx component | Domains 1, 15 |
| mfnc.chimyx | mfnc.muryx | 1 | Magnetic susceptibility, yx component | Domains 1, 15 |
| mfnc.chimzx | mfnc.murzx | 1 | Magnetic susceptibility, zx component | Domains 1, 15 |
| mfnc.chimxy | mfnc.murxy | 1 | Magnetic susceptibility, xy component | Domains 1, 15 |
| mfnc.chimyy | -1+mfnc.muryy | 1 | Magnetic susceptibility, yy component | Domains 1, 15 |
| mfnc.chimzy | mfnc.murzy | 1 | Magnetic susceptibility, zy component | Domains 1, 15 |
| mfnc.chimxz | mfnc.murxz | 1 | Magnetic susceptibility, xz component | Domains 1, 15 |
| mfnc.chimyz | mfnc.muryz | 1 | Magnetic susceptibility, yz component | Domains 1, 15 |
| mfnc.chimzz | -1+mfnc.murzz | 1 | Magnetic susceptibility, zz component | Domains 1, 15 |
| mfnc.Brx | 0 | T | Remanent flux density, x component | Domains 1, 15 |
| mfnc.Bry | 0 | T | Remanent flux density, y component | Domains 1, 15 |
| mfnc.Brz | 0 | T | Remanent flux density, z component | Domains 1, 15 |
| mfnc.normBr | sqrt(realdot(mfnc.Brx,mfnc.Brx)+realdot(mfnc.Bry,mfnc.Bry)+realdot(mfnc.Brz,mfnc.Brz)) | T | Remanent flux density norm | Domains 1, 15 |
| mfnc.unTx | 0 | Pa | Maxwell upward surface stress tensor, x component | Boundaries 1–5, 312 |
| mfnc.unTy | 0 | Pa | Maxwell upward surface stress tensor, y component | Boundaries 1–5, 312 |
| mfnc.unTz | 0 | Pa | Maxwell upward surface stress tensor, z component | Boundaries 1–5, 312 |
| mfnc.unTx | mfnc.unTmx | Pa | Maxwell upward surface stress tensor, x component | Boundaries 136–139, 168–169 |
| mfnc.unTy | mfnc.unTmy | Pa | Maxwell upward surface stress tensor, y component | Boundaries 136–139, 168–169 |
| mfnc.unTz | mfnc.unTmz | Pa | Maxwell upward surface stress tensor, z component | Boundaries 136–139, 168–169 |
| mfnc.unTx | mfnc.unTmx | Pa | Maxwell upward surface stress tensor, x component | Boundaries 6–135, 140–167, 170–311 |
| mfnc.unTy | mfnc.unTmy | Pa | Maxwell upward surface stress tensor, y component | Boundaries 6–135, 140–167, 170–311 |
| mfnc.unTz | mfnc.unTmz | Pa | Maxwell upward surface stress tensor, z component | Boundaries 6–135, 140–167, 170–311 |
| mfnc.dnTx | mfnc.dnTmx | Pa | Maxwell downward surface stress tensor, x component | Boundaries 1–5, 312 |
| mfnc.dnTy | mfnc.dnTmy | Pa | Maxwell downward surface stress tensor, y component | Boundaries 1–5, 312 |
| mfnc.dnTz | mfnc.dnTmz | Pa | Maxwell downward surface stress tensor, z component | Boundaries 1–5, 312 |
| mfnc.dnTx | mfnc.dnTmx | Pa | Maxwell downward surface stress tensor, x component | Boundaries 136–139, 168–169 |
| mfnc.dnTy | mfnc.dnTmy | Pa | Maxwell downward surface stress tensor, y component | Boundaries 136–139, 168–169 |
| mfnc.dnTz | mfnc.dnTmz | Pa | Maxwell downward surface stress tensor, z component | Boundaries 136–139, 168–169 |
| mfnc.dnTx | 0 | Pa | Maxwell downward surface stress tensor, x component | Boundaries 6–135, 140–167, 170–311 |
| mfnc.dnTy | 0 | Pa | Maxwell downward surface stress tensor, y component | Boundaries 6–135, 140–167, 170–311 |
| mfnc.dnTz | 0 | Pa | Maxwell downward surface stress tensor, z component | Boundaries 6–135, 140–167, 170–311 |
| mfnc.unx | unx |  | Normal vector up direction, x component | Boundaries 1–312 |
| mfnc.uny | uny |  | Normal vector up direction, y component | Boundaries 1–312 |
| mfnc.unz | unz |  | Normal vector up direction, z component | Boundaries 1–312 |
| mfnc.dnx | dnx |  | Normal vector down direction, x component | Boundaries 1–312 |
| mfnc.dny | dny |  | Normal vector down direction, y component | Boundaries 1–312 |
| mfnc.dnz | dnz |  | Normal vector down direction, z component | Boundaries 1–312 |
| mfnc.W | mfnc.Wm | J/m^3 | Energy density | Domains 1, 15 |
| mfnc.dWm | mfnc.Wm | J/m^3 | Integrand for total magnetic energy | Domains 1, 15 |
| mfnc.Wm | 0.5\*mu0\_const\*((mfnc.murxx\*mfnc.Hx+mfnc.murxy\*mfnc.Hy+mfnc.murxz\*mfnc.Hz)\*mfnc.Hx+(mfnc.muryx\*mfnc.Hx+mfnc.muryy\*mfnc.Hy+mfnc.muryz\*mfnc.Hz)\*mfnc.Hy+(mfnc.murzx\*mfnc.Hx+mfnc.murzy\*mfnc.Hy+mfnc.murzz\*mfnc.Hz)\*mfnc.Hz) | J/m^3 | Magnetic energy density | Domains 1, 15 |
| mfnc.mfc1.minput\_temperature | model.input.minput\_temperature | K | Temperature | Domains 1, 15 |
| mfnc.mfc1.minput\_pressure | model.input.minput\_pressure | Pa | Absolute pressure | Domains 1, 15 |

##### Shape Functions

| Name | Shape function | Unit | Description | Shape frame | Selection |
| --- | --- | --- | --- | --- | --- |
| Vm | Lagrange (Quadratic) | A | Magnetic scalar potential | Material | Domains 1, 15 |

##### Weak Expressions

| Weak expression | Integration frame | Selection |
| --- | --- | --- |
| mfnc.d\*(-mfnc.Bx\*test(Vmx)-mfnc.By\*test(Vmy)-mfnc.Bz\*test(Vmz)) | Material | Domains 1, 15 |

#### 2.4.2. Magnetic Insulation 1

Magnetic Insulation 1

Selection

| Geometric entity level | Boundary |
| Selection | Boundaries 1–5, 312 |

Equations

##### Shape Functions

| Name | Shape function | Unit | Description | Shape frame | Selection |
| --- | --- | --- | --- | --- | --- |
| Vm | Lagrange (Quadratic) | A | Magnetic scalar potential | Material | Boundaries 6–311 |

#### 2.4.3. Initial Values 1

Initial Values 1

Selection

| Geometric entity level | Domain |
| Selection | Domains 1–32 |

Settings

| Description | Value |
| Magnetic scalar potential | 0 |

#### 2.4.4. ArrayBMagnet1

ArrayBMagnet1

Selection

| Geometric entity level | Domain |
| Selection | Domain 29 |

Equations

Settings

| Description | Value |
| Constitutive relation | Remanent flux density |
| Remanent flux density, x component | BB\*cos(2\*pi\*(0\*60 + corr\_b)/360) |
| Remanent flux density, y component | BB\*sin(2\*pi\*(0\*60 + corr\_b)/360) |
| Remanent flux density, z component | 0 |
| Relative permeability | From material |
| Relative permeability | {{1, 0, 0}, {0, 1, 0}, {0, 0, 1}} |

Properties from material

| Property | Material | Property group |
| Relative permeability | Soft Iron (without losses) | Basic |

##### Variables

| Name | Expression | Unit | Description | Selection |
| --- | --- | --- | --- | --- |
| mfnc.dnTmx | -0.5\*mfnc.unx\*(real(down(mfnc.Bx))\*real(down(mfnc.Hx))+real(down(mfnc.By))\*real(down(mfnc.Hy))+real(down(mfnc.Bz))\*real(down(mfnc.Hz)))+real(down(mfnc.Bx))\*(real(down(mfnc.Hx))\*mfnc.unx+real(down(mfnc.Hy))\*mfnc.uny+real(down(mfnc.Hz))\*mfnc.unz) | Pa | Maxwell downward magnetic surface stress tensor, x component | Boundaries 272–281 |
| mfnc.dnTmy | -0.5\*mfnc.uny\*(real(down(mfnc.Bx))\*real(down(mfnc.Hx))+real(down(mfnc.By))\*real(down(mfnc.Hy))+real(down(mfnc.Bz))\*real(down(mfnc.Hz)))+real(down(mfnc.By))\*(real(down(mfnc.Hx))\*mfnc.unx+real(down(mfnc.Hy))\*mfnc.uny+real(down(mfnc.Hz))\*mfnc.unz) | Pa | Maxwell downward magnetic surface stress tensor, y component | Boundaries 272–281 |
| mfnc.dnTmz | -0.5\*mfnc.unz\*(real(down(mfnc.Bx))\*real(down(mfnc.Hx))+real(down(mfnc.By))\*real(down(mfnc.Hy))+real(down(mfnc.Bz))\*real(down(mfnc.Hz)))+real(down(mfnc.Bz))\*(real(down(mfnc.Hx))\*mfnc.unx+real(down(mfnc.Hy))\*mfnc.uny+real(down(mfnc.Hz))\*mfnc.unz) | Pa | Maxwell downward magnetic surface stress tensor, z component | Boundaries 272–281 |
| mfnc.Hx | -Vmx | A/m | Magnetic field, x component | Domain 29 |
| mfnc.Hy | -Vmy | A/m | Magnetic field, y component | Domain 29 |
| mfnc.Hz | -Vmz | A/m | Magnetic field, z component | Domain 29 |
| mfnc.tHx | -VmTx | A/m | Tangential magnetic field, x component | Boundaries 272–281 |
| mfnc.tHy | -VmTy | A/m | Tangential magnetic field, y component | Boundaries 272–281 |
| mfnc.tHz | -VmTz | A/m | Tangential magnetic field, z component | Boundaries 272–281 |
| mfnc.normH | sqrt(realdot(mfnc.Hx,mfnc.Hx)+realdot(mfnc.Hy,mfnc.Hy)+realdot(mfnc.Hz,mfnc.Hz)) | A/m | Magnetic field norm | Domain 29 |
| mfnc.murxx | model.input.mur11 | 1 | Relative permeability, xx component | Domain 29 |
| mfnc.muryx | model.input.mur21 | 1 | Relative permeability, yx component | Domain 29 |
| mfnc.murzx | model.input.mur31 | 1 | Relative permeability, zx component | Domain 29 |
| mfnc.murxy | model.input.mur12 | 1 | Relative permeability, xy component | Domain 29 |
| mfnc.muryy | model.input.mur22 | 1 | Relative permeability, yy component | Domain 29 |
| mfnc.murzy | model.input.mur32 | 1 | Relative permeability, zy component | Domain 29 |
| mfnc.murxz | model.input.mur13 | 1 | Relative permeability, xz component | Domain 29 |
| mfnc.muryz | model.input.mur23 | 1 | Relative permeability, yz component | Domain 29 |
| mfnc.murzz | model.input.mur33 | 1 | Relative permeability, zz component | Domain 29 |
| mfnc.Bx | mu0\_const\*mfnc.Ixx\*mfnc.Hx+mu0\_const\*mfnc.Ixy\*mfnc.Hy+mu0\_const\*mfnc.Ixz\*mfnc.Hz+mu0\_const\*mfnc.chimxx\*mfnc.Hx+mu0\_const\*mfnc.chimxy\*mfnc.Hy+mu0\_const\*mfnc.chimxz\*mfnc.Hz+mfnc.Brx | T | Magnetic flux density, x component | Domain 29 |
| mfnc.By | mu0\_const\*mfnc.Iyx\*mfnc.Hx+mu0\_const\*mfnc.Iyy\*mfnc.Hy+mu0\_const\*mfnc.Iyz\*mfnc.Hz+mu0\_const\*mfnc.chimyx\*mfnc.Hx+mu0\_const\*mfnc.chimyy\*mfnc.Hy+mu0\_const\*mfnc.chimyz\*mfnc.Hz+mfnc.Bry | T | Magnetic flux density, y component | Domain 29 |
| mfnc.Bz | mu0\_const\*mfnc.Izx\*mfnc.Hx+mu0\_const\*mfnc.Izy\*mfnc.Hy+mu0\_const\*mfnc.Izz\*mfnc.Hz+mu0\_const\*mfnc.chimzx\*mfnc.Hx+mu0\_const\*mfnc.chimzy\*mfnc.Hy+mu0\_const\*mfnc.chimzz\*mfnc.Hz+mfnc.Brz | T | Magnetic flux density, z component | Domain 29 |
| mfnc.normB | sqrt(realdot(mfnc.Bx,mfnc.Bx)+realdot(mfnc.By,mfnc.By)+realdot(mfnc.Bz,mfnc.Bz)) | T | Magnetic flux density norm | Domain 29 |
| mfnc.Mx | mfnc.Bx/mu0\_const-mfnc.Ixx\*mfnc.Hx-mfnc.Ixy\*mfnc.Hy-mfnc.Ixz\*mfnc.Hz | A/m | Magnetization, x component | Domain 29 |
| mfnc.My | mfnc.By/mu0\_const-mfnc.Iyx\*mfnc.Hx-mfnc.Iyy\*mfnc.Hy-mfnc.Iyz\*mfnc.Hz | A/m | Magnetization, y component | Domain 29 |
| mfnc.Mz | mfnc.Bz/mu0\_const-mfnc.Izx\*mfnc.Hx-mfnc.Izy\*mfnc.Hy-mfnc.Izz\*mfnc.Hz | A/m | Magnetization, z component | Domain 29 |
| mfnc.normM | sqrt(realdot(mfnc.Mx,mfnc.Mx)+realdot(mfnc.My,mfnc.My)+realdot(mfnc.Mz,mfnc.Mz)) | A/m | Magnetization norm | Domain 29 |
| mfnc.Ixx | 1 | 1 | Spatial identity matrix, xx component | Domain 29 |
| mfnc.Iyx | 0 | 1 | Spatial identity matrix, yx component | Domain 29 |
| mfnc.Izx | 0 | 1 | Spatial identity matrix, zx component | Domain 29 |
| mfnc.Ixy | 0 | 1 | Spatial identity matrix, xy component | Domain 29 |
| mfnc.Iyy | 1 | 1 | Spatial identity matrix, yy component | Domain 29 |
| mfnc.Izy | 0 | 1 | Spatial identity matrix, zy component | Domain 29 |
| mfnc.Ixz | 0 | 1 | Spatial identity matrix, xz component | Domain 29 |
| mfnc.Iyz | 0 | 1 | Spatial identity matrix, yz component | Domain 29 |
| mfnc.Izz | 1 | 1 | Spatial identity matrix, zz component | Domain 29 |
| mfnc.chimxx | -1+mfnc.murxx | 1 | Magnetic susceptibility, xx component | Domain 29 |
| mfnc.chimyx | mfnc.muryx | 1 | Magnetic susceptibility, yx component | Domain 29 |
| mfnc.chimzx | mfnc.murzx | 1 | Magnetic susceptibility, zx component | Domain 29 |
| mfnc.chimxy | mfnc.murxy | 1 | Magnetic susceptibility, xy component | Domain 29 |
| mfnc.chimyy | -1+mfnc.muryy | 1 | Magnetic susceptibility, yy component | Domain 29 |
| mfnc.chimzy | mfnc.murzy | 1 | Magnetic susceptibility, zy component | Domain 29 |
| mfnc.chimxz | mfnc.murxz | 1 | Magnetic susceptibility, xz component | Domain 29 |
| mfnc.chimyz | mfnc.muryz | 1 | Magnetic susceptibility, yz component | Domain 29 |
| mfnc.chimzz | -1+mfnc.murzz | 1 | Magnetic susceptibility, zz component | Domain 29 |
| mfnc.Brx | BB\*cos(pi\*corr\_b/180) | T | Remanent flux density, x component | Domain 29 |
| mfnc.Bry | BB\*sin(pi\*corr\_b/180) | T | Remanent flux density, y component | Domain 29 |
| mfnc.Brz | 0 | T | Remanent flux density, z component | Domain 29 |
| mfnc.normBr | sqrt(realdot(mfnc.Brx,mfnc.Brx)+realdot(mfnc.Bry,mfnc.Bry)+realdot(mfnc.Brz,mfnc.Brz)) | T | Remanent flux density norm | Domain 29 |
| mfnc.unTx | 0 | Pa | Maxwell upward surface stress tensor, x component | Boundaries 272–281 |
| mfnc.unTy | 0 | Pa | Maxwell upward surface stress tensor, y component | Boundaries 272–281 |
| mfnc.unTz | 0 | Pa | Maxwell upward surface stress tensor, z component | Boundaries 272–281 |
| mfnc.dnTx | mfnc.dnTmx | Pa | Maxwell downward surface stress tensor, x component | Boundaries 272–281 |
| mfnc.dnTy | mfnc.dnTmy | Pa | Maxwell downward surface stress tensor, y component | Boundaries 272–281 |
| mfnc.dnTz | mfnc.dnTmz | Pa | Maxwell downward surface stress tensor, z component | Boundaries 272–281 |
| mfnc.unx | unx |  | Normal vector up direction, x component | Boundaries 272–281 |
| mfnc.uny | uny |  | Normal vector up direction, y component | Boundaries 272–281 |
| mfnc.unz | unz |  | Normal vector up direction, z component | Boundaries 272–281 |
| mfnc.dnx | dnx |  | Normal vector down direction, x component | Boundaries 272–281 |
| mfnc.dny | dny |  | Normal vector down direction, y component | Boundaries 272–281 |
| mfnc.dnz | dnz |  | Normal vector down direction, z component | Boundaries 272–281 |
| mfnc.W | mfnc.Wm | J/m^3 | Energy density | Domain 29 |
| mfnc.dWm | mfnc.Wm | J/m^3 | Integrand for total magnetic energy | Domain 29 |
| mfnc.Wm | 0.5\*mu0\_const\*((mfnc.murxx\*mfnc.Hx+mfnc.murxy\*mfnc.Hy+mfnc.murxz\*mfnc.Hz)\*mfnc.Hx+(mfnc.muryx\*mfnc.Hx+mfnc.muryy\*mfnc.Hy+mfnc.muryz\*mfnc.Hz)\*mfnc.Hy+(mfnc.murzx\*mfnc.Hx+mfnc.murzy\*mfnc.Hy+mfnc.murzz\*mfnc.Hz)\*mfnc.Hz) | J/m^3 | Magnetic energy density | Domain 29 |

##### Shape Functions

| Name | Shape function | Unit | Description | Shape frame | Selection |
| --- | --- | --- | --- | --- | --- |
| Vm | Lagrange (Quadratic) | A | Magnetic scalar potential | Material | Domain 29 |

##### Weak Expressions

| Weak expression | Integration frame | Selection |
| --- | --- | --- |
| mfnc.d\*(-mfnc.Bx\*test(Vmx)-mfnc.By\*test(Vmy)-mfnc.Bz\*test(Vmz)) | Material | Domain 29 |

#### 2.4.5. ArrayBMagnet2

ArrayBMagnet2

Selection

| Geometric entity level | Domain |
| Selection | Domain 28 |

Equations

Settings

| Description | Value |
| Constitutive relation | Remanent flux density |
| Remanent flux density, x component | BB\*cos(2\*pi/360\*(1\*60 + corr\_b)) |
| Remanent flux density, y component | BB\*sin(2\*pi/360\*(1\*60 + corr\_b)) |
| Remanent flux density, z component | 0 |
| Relative permeability | From material |
| Relative permeability | {{1, 0, 0}, {0, 1, 0}, {0, 0, 1}} |

Properties from material

| Property | Material | Property group |
| Relative permeability | Soft Iron (without losses) | Basic |

##### Variables

| Name | Expression | Unit | Description | Selection |
| --- | --- | --- | --- | --- |
| mfnc.dnTmx | -0.5\*mfnc.unx\*(real(down(mfnc.Bx))\*real(down(mfnc.Hx))+real(down(mfnc.By))\*real(down(mfnc.Hy))+real(down(mfnc.Bz))\*real(down(mfnc.Hz)))+real(down(mfnc.Bx))\*(real(down(mfnc.Hx))\*mfnc.unx+real(down(mfnc.Hy))\*mfnc.uny+real(down(mfnc.Hz))\*mfnc.unz) | Pa | Maxwell downward magnetic surface stress tensor, x component | Boundaries 254–257, 259, 261, 263, 267, 269, 271 |
| mfnc.dnTmy | -0.5\*mfnc.uny\*(real(down(mfnc.Bx))\*real(down(mfnc.Hx))+real(down(mfnc.By))\*real(down(mfnc.Hy))+real(down(mfnc.Bz))\*real(down(mfnc.Hz)))+real(down(mfnc.By))\*(real(down(mfnc.Hx))\*mfnc.unx+real(down(mfnc.Hy))\*mfnc.uny+real(down(mfnc.Hz))\*mfnc.unz) | Pa | Maxwell downward magnetic surface stress tensor, y component | Boundaries 254–257, 259, 261, 263, 267, 269, 271 |
| mfnc.dnTmz | -0.5\*mfnc.unz\*(real(down(mfnc.Bx))\*real(down(mfnc.Hx))+real(down(mfnc.By))\*real(down(mfnc.Hy))+real(down(mfnc.Bz))\*real(down(mfnc.Hz)))+real(down(mfnc.Bz))\*(real(down(mfnc.Hx))\*mfnc.unx+real(down(mfnc.Hy))\*mfnc.uny+real(down(mfnc.Hz))\*mfnc.unz) | Pa | Maxwell downward magnetic surface stress tensor, z component | Boundaries 254–257, 259, 261, 263, 267, 269, 271 |
| mfnc.Hx | -Vmx | A/m | Magnetic field, x component | Domain 28 |
| mfnc.Hy | -Vmy | A/m | Magnetic field, y component | Domain 28 |
| mfnc.Hz | -Vmz | A/m | Magnetic field, z component | Domain 28 |
| mfnc.tHx | -VmTx | A/m | Tangential magnetic field, x component | Boundaries 254–257, 259, 261, 263, 267, 269, 271 |
| mfnc.tHy | -VmTy | A/m | Tangential magnetic field, y component | Boundaries 254–257, 259, 261, 263, 267, 269, 271 |
| mfnc.tHz | -VmTz | A/m | Tangential magnetic field, z component | Boundaries 254–257, 259, 261, 263, 267, 269, 271 |
| mfnc.normH | sqrt(realdot(mfnc.Hx,mfnc.Hx)+realdot(mfnc.Hy,mfnc.Hy)+realdot(mfnc.Hz,mfnc.Hz)) | A/m | Magnetic field norm | Domain 28 |
| mfnc.murxx | model.input.mur11 | 1 | Relative permeability, xx component | Domain 28 |
| mfnc.muryx | model.input.mur21 | 1 | Relative permeability, yx component | Domain 28 |
| mfnc.murzx | model.input.mur31 | 1 | Relative permeability, zx component | Domain 28 |
| mfnc.murxy | model.input.mur12 | 1 | Relative permeability, xy component | Domain 28 |
| mfnc.muryy | model.input.mur22 | 1 | Relative permeability, yy component | Domain 28 |
| mfnc.murzy | model.input.mur32 | 1 | Relative permeability, zy component | Domain 28 |
| mfnc.murxz | model.input.mur13 | 1 | Relative permeability, xz component | Domain 28 |
| mfnc.muryz | model.input.mur23 | 1 | Relative permeability, yz component | Domain 28 |
| mfnc.murzz | model.input.mur33 | 1 | Relative permeability, zz component | Domain 28 |
| mfnc.Bx | mu0\_const\*mfnc.Ixx\*mfnc.Hx+mu0\_const\*mfnc.Ixy\*mfnc.Hy+mu0\_const\*mfnc.Ixz\*mfnc.Hz+mu0\_const\*mfnc.chimxx\*mfnc.Hx+mu0\_const\*mfnc.chimxy\*mfnc.Hy+mu0\_const\*mfnc.chimxz\*mfnc.Hz+mfnc.Brx | T | Magnetic flux density, x component | Domain 28 |
| mfnc.By | mu0\_const\*mfnc.Iyx\*mfnc.Hx+mu0\_const\*mfnc.Iyy\*mfnc.Hy+mu0\_const\*mfnc.Iyz\*mfnc.Hz+mu0\_const\*mfnc.chimyx\*mfnc.Hx+mu0\_const\*mfnc.chimyy\*mfnc.Hy+mu0\_const\*mfnc.chimyz\*mfnc.Hz+mfnc.Bry | T | Magnetic flux density, y component | Domain 28 |
| mfnc.Bz | mu0\_const\*mfnc.Izx\*mfnc.Hx+mu0\_const\*mfnc.Izy\*mfnc.Hy+mu0\_const\*mfnc.Izz\*mfnc.Hz+mu0\_const\*mfnc.chimzx\*mfnc.Hx+mu0\_const\*mfnc.chimzy\*mfnc.Hy+mu0\_const\*mfnc.chimzz\*mfnc.Hz+mfnc.Brz | T | Magnetic flux density, z component | Domain 28 |
| mfnc.normB | sqrt(realdot(mfnc.Bx,mfnc.Bx)+realdot(mfnc.By,mfnc.By)+realdot(mfnc.Bz,mfnc.Bz)) | T | Magnetic flux density norm | Domain 28 |
| mfnc.Mx | mfnc.Bx/mu0\_const-mfnc.Ixx\*mfnc.Hx-mfnc.Ixy\*mfnc.Hy-mfnc.Ixz\*mfnc.Hz | A/m | Magnetization, x component | Domain 28 |
| mfnc.My | mfnc.By/mu0\_const-mfnc.Iyx\*mfnc.Hx-mfnc.Iyy\*mfnc.Hy-mfnc.Iyz\*mfnc.Hz | A/m | Magnetization, y component | Domain 28 |
| mfnc.Mz | mfnc.Bz/mu0\_const-mfnc.Izx\*mfnc.Hx-mfnc.Izy\*mfnc.Hy-mfnc.Izz\*mfnc.Hz | A/m | Magnetization, z component | Domain 28 |
| mfnc.normM | sqrt(realdot(mfnc.Mx,mfnc.Mx)+realdot(mfnc.My,mfnc.My)+realdot(mfnc.Mz,mfnc.Mz)) | A/m | Magnetization norm | Domain 28 |
| mfnc.Ixx | 1 | 1 | Spatial identity matrix, xx component | Domain 28 |
| mfnc.Iyx | 0 | 1 | Spatial identity matrix, yx component | Domain 28 |
| mfnc.Izx | 0 | 1 | Spatial identity matrix, zx component | Domain 28 |
| mfnc.Ixy | 0 | 1 | Spatial identity matrix, xy component | Domain 28 |
| mfnc.Iyy | 1 | 1 | Spatial identity matrix, yy component | Domain 28 |
| mfnc.Izy | 0 | 1 | Spatial identity matrix, zy component | Domain 28 |
| mfnc.Ixz | 0 | 1 | Spatial identity matrix, xz component | Domain 28 |
| mfnc.Iyz | 0 | 1 | Spatial identity matrix, yz component | Domain 28 |
| mfnc.Izz | 1 | 1 | Spatial identity matrix, zz component | Domain 28 |
| mfnc.chimxx | -1+mfnc.murxx | 1 | Magnetic susceptibility, xx component | Domain 28 |
| mfnc.chimyx | mfnc.muryx | 1 | Magnetic susceptibility, yx component | Domain 28 |
| mfnc.chimzx | mfnc.murzx | 1 | Magnetic susceptibility, zx component | Domain 28 |
| mfnc.chimxy | mfnc.murxy | 1 | Magnetic susceptibility, xy component | Domain 28 |
| mfnc.chimyy | -1+mfnc.muryy | 1 | Magnetic susceptibility, yy component | Domain 28 |
| mfnc.chimzy | mfnc.murzy | 1 | Magnetic susceptibility, zy component | Domain 28 |
| mfnc.chimxz | mfnc.murxz | 1 | Magnetic susceptibility, xz component | Domain 28 |
| mfnc.chimyz | mfnc.muryz | 1 | Magnetic susceptibility, yz component | Domain 28 |
| mfnc.chimzz | -1+mfnc.murzz | 1 | Magnetic susceptibility, zz component | Domain 28 |
| mfnc.Brx | BB\*cos(pi\*(60+corr\_b)/180) | T | Remanent flux density, x component | Domain 28 |
| mfnc.Bry | BB\*sin(pi\*(60+corr\_b)/180) | T | Remanent flux density, y component | Domain 28 |
| mfnc.Brz | 0 | T | Remanent flux density, z component | Domain 28 |
| mfnc.normBr | sqrt(realdot(mfnc.Brx,mfnc.Brx)+realdot(mfnc.Bry,mfnc.Bry)+realdot(mfnc.Brz,mfnc.Brz)) | T | Remanent flux density norm | Domain 28 |
| mfnc.unTx | 0 | Pa | Maxwell upward surface stress tensor, x component | Boundaries 254–257, 259, 261, 263, 267, 269, 271 |
| mfnc.unTy | 0 | Pa | Maxwell upward surface stress tensor, y component | Boundaries 254–257, 259, 261, 263, 267, 269, 271 |
| mfnc.unTz | 0 | Pa | Maxwell upward surface stress tensor, z component | Boundaries 254–257, 259, 261, 263, 267, 269, 271 |
| mfnc.dnTx | mfnc.dnTmx | Pa | Maxwell downward surface stress tensor, x component | Boundaries 254–257, 259, 261, 263, 267, 269, 271 |
| mfnc.dnTy | mfnc.dnTmy | Pa | Maxwell downward surface stress tensor, y component | Boundaries 254–257, 259, 261, 263, 267, 269, 271 |
| mfnc.dnTz | mfnc.dnTmz | Pa | Maxwell downward surface stress tensor, z component | Boundaries 254–257, 259, 261, 263, 267, 269, 271 |
| mfnc.unx | unx |  | Normal vector up direction, x component | Boundaries 254–257, 259, 261, 263, 267, 269, 271 |
| mfnc.uny | uny |  | Normal vector up direction, y component | Boundaries 254–257, 259, 261, 263, 267, 269, 271 |
| mfnc.unz | unz |  | Normal vector up direction, z component | Boundaries 254–257, 259, 261, 263, 267, 269, 271 |
| mfnc.dnx | dnx |  | Normal vector down direction, x component | Boundaries 254–257, 259, 261, 263, 267, 269, 271 |
| mfnc.dny | dny |  | Normal vector down direction, y component | Boundaries 254–257, 259, 261, 263, 267, 269, 271 |
| mfnc.dnz | dnz |  | Normal vector down direction, z component | Boundaries 254–257, 259, 261, 263, 267, 269, 271 |
| mfnc.W | mfnc.Wm | J/m^3 | Energy density | Domain 28 |
| mfnc.dWm | mfnc.Wm | J/m^3 | Integrand for total magnetic energy | Domain 28 |
| mfnc.Wm | 0.5\*mu0\_const\*((mfnc.murxx\*mfnc.Hx+mfnc.murxy\*mfnc.Hy+mfnc.murxz\*mfnc.Hz)\*mfnc.Hx+(mfnc.muryx\*mfnc.Hx+mfnc.muryy\*mfnc.Hy+mfnc.muryz\*mfnc.Hz)\*mfnc.Hy+(mfnc.murzx\*mfnc.Hx+mfnc.murzy\*mfnc.Hy+mfnc.murzz\*mfnc.Hz)\*mfnc.Hz) | J/m^3 | Magnetic energy density | Domain 28 |

##### Shape Functions

| Name | Shape function | Unit | Description | Shape frame | Selection |
| --- | --- | --- | --- | --- | --- |
| Vm | Lagrange (Quadratic) | A | Magnetic scalar potential | Material | Domain 28 |

##### Weak Expressions

| Weak expression | Integration frame | Selection |
| --- | --- | --- |
| mfnc.d\*(-mfnc.Bx\*test(Vmx)-mfnc.By\*test(Vmy)-mfnc.Bz\*test(Vmz)) | Material | Domain 28 |

#### 2.4.6. ArrayBMagnet3

ArrayBMagnet3

Selection

| Geometric entity level | Domain |
| Selection | Domain 23 |

Equations

Settings

| Description | Value |
| Constitutive relation | Remanent flux density |
| Remanent flux density, x component | BB\*cos(2\*pi/360\*(2\*60 + corr\_b)) |
| Remanent flux density, y component | BB\*sin(2\*pi/360\*(2\*60 + corr\_b)) |
| Remanent flux density, z component | 0 |
| Relative permeability | From material |
| Relative permeability | {{1, 0, 0}, {0, 1, 0}, {0, 0, 1}} |

Properties from material

| Property | Material | Property group |
| Relative permeability | Soft Iron (without losses) | Basic |

##### Variables

| Name | Expression | Unit | Description | Selection |
| --- | --- | --- | --- | --- |
| mfnc.dnTmx | -0.5\*mfnc.unx\*(real(down(mfnc.Bx))\*real(down(mfnc.Hx))+real(down(mfnc.By))\*real(down(mfnc.Hy))+real(down(mfnc.Bz))\*real(down(mfnc.Hz)))+real(down(mfnc.Bx))\*(real(down(mfnc.Hx))\*mfnc.unx+real(down(mfnc.Hy))\*mfnc.uny+real(down(mfnc.Hz))\*mfnc.unz) | Pa | Maxwell downward magnetic surface stress tensor, x component | Boundaries 206–209, 211, 213, 215, 217, 219, 231 |
| mfnc.dnTmy | -0.5\*mfnc.uny\*(real(down(mfnc.Bx))\*real(down(mfnc.Hx))+real(down(mfnc.By))\*real(down(mfnc.Hy))+real(down(mfnc.Bz))\*real(down(mfnc.Hz)))+real(down(mfnc.By))\*(real(down(mfnc.Hx))\*mfnc.unx+real(down(mfnc.Hy))\*mfnc.uny+real(down(mfnc.Hz))\*mfnc.unz) | Pa | Maxwell downward magnetic surface stress tensor, y component | Boundaries 206–209, 211, 213, 215, 217, 219, 231 |
| mfnc.dnTmz | -0.5\*mfnc.unz\*(real(down(mfnc.Bx))\*real(down(mfnc.Hx))+real(down(mfnc.By))\*real(down(mfnc.Hy))+real(down(mfnc.Bz))\*real(down(mfnc.Hz)))+real(down(mfnc.Bz))\*(real(down(mfnc.Hx))\*mfnc.unx+real(down(mfnc.Hy))\*mfnc.uny+real(down(mfnc.Hz))\*mfnc.unz) | Pa | Maxwell downward magnetic surface stress tensor, z component | Boundaries 206–209, 211, 213, 215, 217, 219, 231 |
| mfnc.Hx | -Vmx | A/m | Magnetic field, x component | Domain 23 |
| mfnc.Hy | -Vmy | A/m | Magnetic field, y component | Domain 23 |
| mfnc.Hz | -Vmz | A/m | Magnetic field, z component | Domain 23 |
| mfnc.tHx | -VmTx | A/m | Tangential magnetic field, x component | Boundaries 206–209, 211, 213, 215, 217, 219, 231 |
| mfnc.tHy | -VmTy | A/m | Tangential magnetic field, y component | Boundaries 206–209, 211, 213, 215, 217, 219, 231 |
| mfnc.tHz | -VmTz | A/m | Tangential magnetic field, z component | Boundaries 206–209, 211, 213, 215, 217, 219, 231 |
| mfnc.normH | sqrt(realdot(mfnc.Hx,mfnc.Hx)+realdot(mfnc.Hy,mfnc.Hy)+realdot(mfnc.Hz,mfnc.Hz)) | A/m | Magnetic field norm | Domain 23 |
| mfnc.murxx | model.input.mur11 | 1 | Relative permeability, xx component | Domain 23 |
| mfnc.muryx | model.input.mur21 | 1 | Relative permeability, yx component | Domain 23 |
| mfnc.murzx | model.input.mur31 | 1 | Relative permeability, zx component | Domain 23 |
| mfnc.murxy | model.input.mur12 | 1 | Relative permeability, xy component | Domain 23 |
| mfnc.muryy | model.input.mur22 | 1 | Relative permeability, yy component | Domain 23 |
| mfnc.murzy | model.input.mur32 | 1 | Relative permeability, zy component | Domain 23 |
| mfnc.murxz | model.input.mur13 | 1 | Relative permeability, xz component | Domain 23 |
| mfnc.muryz | model.input.mur23 | 1 | Relative permeability, yz component | Domain 23 |
| mfnc.murzz | model.input.mur33 | 1 | Relative permeability, zz component | Domain 23 |
| mfnc.Bx | mu0\_const\*mfnc.Ixx\*mfnc.Hx+mu0\_const\*mfnc.Ixy\*mfnc.Hy+mu0\_const\*mfnc.Ixz\*mfnc.Hz+mu0\_const\*mfnc.chimxx\*mfnc.Hx+mu0\_const\*mfnc.chimxy\*mfnc.Hy+mu0\_const\*mfnc.chimxz\*mfnc.Hz+mfnc.Brx | T | Magnetic flux density, x component | Domain 23 |
| mfnc.By | mu0\_const\*mfnc.Iyx\*mfnc.Hx+mu0\_const\*mfnc.Iyy\*mfnc.Hy+mu0\_const\*mfnc.Iyz\*mfnc.Hz+mu0\_const\*mfnc.chimyx\*mfnc.Hx+mu0\_const\*mfnc.chimyy\*mfnc.Hy+mu0\_const\*mfnc.chimyz\*mfnc.Hz+mfnc.Bry | T | Magnetic flux density, y component | Domain 23 |
| mfnc.Bz | mu0\_const\*mfnc.Izx\*mfnc.Hx+mu0\_const\*mfnc.Izy\*mfnc.Hy+mu0\_const\*mfnc.Izz\*mfnc.Hz+mu0\_const\*mfnc.chimzx\*mfnc.Hx+mu0\_const\*mfnc.chimzy\*mfnc.Hy+mu0\_const\*mfnc.chimzz\*mfnc.Hz+mfnc.Brz | T | Magnetic flux density, z component | Domain 23 |
| mfnc.normB | sqrt(realdot(mfnc.Bx,mfnc.Bx)+realdot(mfnc.By,mfnc.By)+realdot(mfnc.Bz,mfnc.Bz)) | T | Magnetic flux density norm | Domain 23 |
| mfnc.Mx | mfnc.Bx/mu0\_const-mfnc.Ixx\*mfnc.Hx-mfnc.Ixy\*mfnc.Hy-mfnc.Ixz\*mfnc.Hz | A/m | Magnetization, x component | Domain 23 |
| mfnc.My | mfnc.By/mu0\_const-mfnc.Iyx\*mfnc.Hx-mfnc.Iyy\*mfnc.Hy-mfnc.Iyz\*mfnc.Hz | A/m | Magnetization, y component | Domain 23 |
| mfnc.Mz | mfnc.Bz/mu0\_const-mfnc.Izx\*mfnc.Hx-mfnc.Izy\*mfnc.Hy-mfnc.Izz\*mfnc.Hz | A/m | Magnetization, z component | Domain 23 |
| mfnc.normM | sqrt(realdot(mfnc.Mx,mfnc.Mx)+realdot(mfnc.My,mfnc.My)+realdot(mfnc.Mz,mfnc.Mz)) | A/m | Magnetization norm | Domain 23 |
| mfnc.Ixx | 1 | 1 | Spatial identity matrix, xx component | Domain 23 |
| mfnc.Iyx | 0 | 1 | Spatial identity matrix, yx component | Domain 23 |
| mfnc.Izx | 0 | 1 | Spatial identity matrix, zx component | Domain 23 |
| mfnc.Ixy | 0 | 1 | Spatial identity matrix, xy component | Domain 23 |
| mfnc.Iyy | 1 | 1 | Spatial identity matrix, yy component | Domain 23 |
| mfnc.Izy | 0 | 1 | Spatial identity matrix, zy component | Domain 23 |
| mfnc.Ixz | 0 | 1 | Spatial identity matrix, xz component | Domain 23 |
| mfnc.Iyz | 0 | 1 | Spatial identity matrix, yz component | Domain 23 |
| mfnc.Izz | 1 | 1 | Spatial identity matrix, zz component | Domain 23 |
| mfnc.chimxx | -1+mfnc.murxx | 1 | Magnetic susceptibility, xx component | Domain 23 |
| mfnc.chimyx | mfnc.muryx | 1 | Magnetic susceptibility, yx component | Domain 23 |
| mfnc.chimzx | mfnc.murzx | 1 | Magnetic susceptibility, zx component | Domain 23 |
| mfnc.chimxy | mfnc.murxy | 1 | Magnetic susceptibility, xy component | Domain 23 |
| mfnc.chimyy | -1+mfnc.muryy | 1 | Magnetic susceptibility, yy component | Domain 23 |
| mfnc.chimzy | mfnc.murzy | 1 | Magnetic susceptibility, zy component | Domain 23 |
| mfnc.chimxz | mfnc.murxz | 1 | Magnetic susceptibility, xz component | Domain 23 |
| mfnc.chimyz | mfnc.muryz | 1 | Magnetic susceptibility, yz component | Domain 23 |
| mfnc.chimzz | -1+mfnc.murzz | 1 | Magnetic susceptibility, zz component | Domain 23 |
| mfnc.Brx | BB\*cos(pi\*(120+corr\_b)/180) | T | Remanent flux density, x component | Domain 23 |
| mfnc.Bry | BB\*sin(pi\*(120+corr\_b)/180) | T | Remanent flux density, y component | Domain 23 |
| mfnc.Brz | 0 | T | Remanent flux density, z component | Domain 23 |
| mfnc.normBr | sqrt(realdot(mfnc.Brx,mfnc.Brx)+realdot(mfnc.Bry,mfnc.Bry)+realdot(mfnc.Brz,mfnc.Brz)) | T | Remanent flux density norm | Domain 23 |
| mfnc.unTx | 0 | Pa | Maxwell upward surface stress tensor, x component | Boundaries 206–209, 211, 213, 215, 217, 219, 231 |
| mfnc.unTy | 0 | Pa | Maxwell upward surface stress tensor, y component | Boundaries 206–209, 211, 213, 215, 217, 219, 231 |
| mfnc.unTz | 0 | Pa | Maxwell upward surface stress tensor, z component | Boundaries 206–209, 211, 213, 215, 217, 219, 231 |
| mfnc.dnTx | mfnc.dnTmx | Pa | Maxwell downward surface stress tensor, x component | Boundaries 206–209, 211, 213, 215, 217, 219, 231 |
| mfnc.dnTy | mfnc.dnTmy | Pa | Maxwell downward surface stress tensor, y component | Boundaries 206–209, 211, 213, 215, 217, 219, 231 |
| mfnc.dnTz | mfnc.dnTmz | Pa | Maxwell downward surface stress tensor, z component | Boundaries 206–209, 211, 213, 215, 217, 219, 231 |
| mfnc.unx | unx |  | Normal vector up direction, x component | Boundaries 206–209, 211, 213, 215, 217, 219, 231 |
| mfnc.uny | uny |  | Normal vector up direction, y component | Boundaries 206–209, 211, 213, 215, 217, 219, 231 |
| mfnc.unz | unz |  | Normal vector up direction, z component | Boundaries 206–209, 211, 213, 215, 217, 219, 231 |
| mfnc.dnx | dnx |  | Normal vector down direction, x component | Boundaries 206–209, 211, 213, 215, 217, 219, 231 |
| mfnc.dny | dny |  | Normal vector down direction, y component | Boundaries 206–209, 211, 213, 215, 217, 219, 231 |
| mfnc.dnz | dnz |  | Normal vector down direction, z component | Boundaries 206–209, 211, 213, 215, 217, 219, 231 |
| mfnc.W | mfnc.Wm | J/m^3 | Energy density | Domain 23 |
| mfnc.dWm | mfnc.Wm | J/m^3 | Integrand for total magnetic energy | Domain 23 |
| mfnc.Wm | 0.5\*mu0\_const\*((mfnc.murxx\*mfnc.Hx+mfnc.murxy\*mfnc.Hy+mfnc.murxz\*mfnc.Hz)\*mfnc.Hx+(mfnc.muryx\*mfnc.Hx+mfnc.muryy\*mfnc.Hy+mfnc.muryz\*mfnc.Hz)\*mfnc.Hy+(mfnc.murzx\*mfnc.Hx+mfnc.murzy\*mfnc.Hy+mfnc.murzz\*mfnc.Hz)\*mfnc.Hz) | J/m^3 | Magnetic energy density | Domain 23 |

##### Shape Functions

| Name | Shape function | Unit | Description | Shape frame | Selection |
| --- | --- | --- | --- | --- | --- |
| Vm | Lagrange (Quadratic) | A | Magnetic scalar potential | Material | Domain 23 |

##### Weak Expressions

| Weak expression | Integration frame | Selection |
| --- | --- | --- |
| mfnc.d\*(-mfnc.Bx\*test(Vmx)-mfnc.By\*test(Vmy)-mfnc.Bz\*test(Vmz)) | Material | Domain 23 |

#### 2.4.7. ArrayBMagnet4

ArrayBMagnet4

Selection

| Geometric entity level | Domain |
| Selection | Domain 19 |

Equations

Settings

| Description | Value |
| Constitutive relation | Remanent flux density |
| Remanent flux density, x component | BB\*cos(2\*pi/360\*(3\*60 + corr\_b)) |
| Remanent flux density, y component | BB\*sin(2\*pi/360\*(3\*60 + corr\_b)) |
| Remanent flux density, z component | 0 |
| Relative permeability | From material |
| Relative permeability | {{1, 0, 0}, {0, 1, 0}, {0, 0, 1}} |

Properties from material

| Property | Material | Property group |
| Relative permeability | Soft Iron (without losses) | Basic |

##### Variables

| Name | Expression | Unit | Description | Selection |
| --- | --- | --- | --- | --- |
| mfnc.dnTmx | -0.5\*mfnc.unx\*(real(down(mfnc.Bx))\*real(down(mfnc.Hx))+real(down(mfnc.By))\*real(down(mfnc.Hy))+real(down(mfnc.Bz))\*real(down(mfnc.Hz)))+real(down(mfnc.Bx))\*(real(down(mfnc.Hx))\*mfnc.unx+real(down(mfnc.Hy))\*mfnc.uny+real(down(mfnc.Hz))\*mfnc.unz) | Pa | Maxwell downward magnetic surface stress tensor, x component | Boundaries 159–163, 166–167, 172–173, 175 |
| mfnc.dnTmy | -0.5\*mfnc.uny\*(real(down(mfnc.Bx))\*real(down(mfnc.Hx))+real(down(mfnc.By))\*real(down(mfnc.Hy))+real(down(mfnc.Bz))\*real(down(mfnc.Hz)))+real(down(mfnc.By))\*(real(down(mfnc.Hx))\*mfnc.unx+real(down(mfnc.Hy))\*mfnc.uny+real(down(mfnc.Hz))\*mfnc.unz) | Pa | Maxwell downward magnetic surface stress tensor, y component | Boundaries 159–163, 166–167, 172–173, 175 |
| mfnc.dnTmz | -0.5\*mfnc.unz\*(real(down(mfnc.Bx))\*real(down(mfnc.Hx))+real(down(mfnc.By))\*real(down(mfnc.Hy))+real(down(mfnc.Bz))\*real(down(mfnc.Hz)))+real(down(mfnc.Bz))\*(real(down(mfnc.Hx))\*mfnc.unx+real(down(mfnc.Hy))\*mfnc.uny+real(down(mfnc.Hz))\*mfnc.unz) | Pa | Maxwell downward magnetic surface stress tensor, z component | Boundaries 159–163, 166–167, 172–173, 175 |
| mfnc.Hx | -Vmx | A/m | Magnetic field, x component | Domain 19 |
| mfnc.Hy | -Vmy | A/m | Magnetic field, y component | Domain 19 |
| mfnc.Hz | -Vmz | A/m | Magnetic field, z component | Domain 19 |
| mfnc.tHx | -VmTx | A/m | Tangential magnetic field, x component | Boundaries 159–163, 166–167, 172–173, 175 |
| mfnc.tHy | -VmTy | A/m | Tangential magnetic field, y component | Boundaries 159–163, 166–167, 172–173, 175 |
| mfnc.tHz | -VmTz | A/m | Tangential magnetic field, z component | Boundaries 159–163, 166–167, 172–173, 175 |
| mfnc.normH | sqrt(realdot(mfnc.Hx,mfnc.Hx)+realdot(mfnc.Hy,mfnc.Hy)+realdot(mfnc.Hz,mfnc.Hz)) | A/m | Magnetic field norm | Domain 19 |
| mfnc.murxx | model.input.mur11 | 1 | Relative permeability, xx component | Domain 19 |
| mfnc.muryx | model.input.mur21 | 1 | Relative permeability, yx component | Domain 19 |
| mfnc.murzx | model.input.mur31 | 1 | Relative permeability, zx component | Domain 19 |
| mfnc.murxy | model.input.mur12 | 1 | Relative permeability, xy component | Domain 19 |
| mfnc.muryy | model.input.mur22 | 1 | Relative permeability, yy component | Domain 19 |
| mfnc.murzy | model.input.mur32 | 1 | Relative permeability, zy component | Domain 19 |
| mfnc.murxz | model.input.mur13 | 1 | Relative permeability, xz component | Domain 19 |
| mfnc.muryz | model.input.mur23 | 1 | Relative permeability, yz component | Domain 19 |
| mfnc.murzz | model.input.mur33 | 1 | Relative permeability, zz component | Domain 19 |
| mfnc.Bx | mu0\_const\*mfnc.Ixx\*mfnc.Hx+mu0\_const\*mfnc.Ixy\*mfnc.Hy+mu0\_const\*mfnc.Ixz\*mfnc.Hz+mu0\_const\*mfnc.chimxx\*mfnc.Hx+mu0\_const\*mfnc.chimxy\*mfnc.Hy+mu0\_const\*mfnc.chimxz\*mfnc.Hz+mfnc.Brx | T | Magnetic flux density, x component | Domain 19 |
| mfnc.By | mu0\_const\*mfnc.Iyx\*mfnc.Hx+mu0\_const\*mfnc.Iyy\*mfnc.Hy+mu0\_const\*mfnc.Iyz\*mfnc.Hz+mu0\_const\*mfnc.chimyx\*mfnc.Hx+mu0\_const\*mfnc.chimyy\*mfnc.Hy+mu0\_const\*mfnc.chimyz\*mfnc.Hz+mfnc.Bry | T | Magnetic flux density, y component | Domain 19 |
| mfnc.Bz | mu0\_const\*mfnc.Izx\*mfnc.Hx+mu0\_const\*mfnc.Izy\*mfnc.Hy+mu0\_const\*mfnc.Izz\*mfnc.Hz+mu0\_const\*mfnc.chimzx\*mfnc.Hx+mu0\_const\*mfnc.chimzy\*mfnc.Hy+mu0\_const\*mfnc.chimzz\*mfnc.Hz+mfnc.Brz | T | Magnetic flux density, z component | Domain 19 |
| mfnc.normB | sqrt(realdot(mfnc.Bx,mfnc.Bx)+realdot(mfnc.By,mfnc.By)+realdot(mfnc.Bz,mfnc.Bz)) | T | Magnetic flux density norm | Domain 19 |
| mfnc.Mx | mfnc.Bx/mu0\_const-mfnc.Ixx\*mfnc.Hx-mfnc.Ixy\*mfnc.Hy-mfnc.Ixz\*mfnc.Hz | A/m | Magnetization, x component | Domain 19 |
| mfnc.My | mfnc.By/mu0\_const-mfnc.Iyx\*mfnc.Hx-mfnc.Iyy\*mfnc.Hy-mfnc.Iyz\*mfnc.Hz | A/m | Magnetization, y component | Domain 19 |
| mfnc.Mz | mfnc.Bz/mu0\_const-mfnc.Izx\*mfnc.Hx-mfnc.Izy\*mfnc.Hy-mfnc.Izz\*mfnc.Hz | A/m | Magnetization, z component | Domain 19 |
| mfnc.normM | sqrt(realdot(mfnc.Mx,mfnc.Mx)+realdot(mfnc.My,mfnc.My)+realdot(mfnc.Mz,mfnc.Mz)) | A/m | Magnetization norm | Domain 19 |
| mfnc.Ixx | 1 | 1 | Spatial identity matrix, xx component | Domain 19 |
| mfnc.Iyx | 0 | 1 | Spatial identity matrix, yx component | Domain 19 |
| mfnc.Izx | 0 | 1 | Spatial identity matrix, zx component | Domain 19 |
| mfnc.Ixy | 0 | 1 | Spatial identity matrix, xy component | Domain 19 |
| mfnc.Iyy | 1 | 1 | Spatial identity matrix, yy component | Domain 19 |
| mfnc.Izy | 0 | 1 | Spatial identity matrix, zy component | Domain 19 |
| mfnc.Ixz | 0 | 1 | Spatial identity matrix, xz component | Domain 19 |
| mfnc.Iyz | 0 | 1 | Spatial identity matrix, yz component | Domain 19 |
| mfnc.Izz | 1 | 1 | Spatial identity matrix, zz component | Domain 19 |
| mfnc.chimxx | -1+mfnc.murxx | 1 | Magnetic susceptibility, xx component | Domain 19 |
| mfnc.chimyx | mfnc.muryx | 1 | Magnetic susceptibility, yx component | Domain 19 |
| mfnc.chimzx | mfnc.murzx | 1 | Magnetic susceptibility, zx component | Domain 19 |
| mfnc.chimxy | mfnc.murxy | 1 | Magnetic susceptibility, xy component | Domain 19 |
| mfnc.chimyy | -1+mfnc.muryy | 1 | Magnetic susceptibility, yy component | Domain 19 |
| mfnc.chimzy | mfnc.murzy | 1 | Magnetic susceptibility, zy component | Domain 19 |
| mfnc.chimxz | mfnc.murxz | 1 | Magnetic susceptibility, xz component | Domain 19 |
| mfnc.chimyz | mfnc.muryz | 1 | Magnetic susceptibility, yz component | Domain 19 |
| mfnc.chimzz | -1+mfnc.murzz | 1 | Magnetic susceptibility, zz component | Domain 19 |
| mfnc.Brx | BB\*cos(pi\*(180+corr\_b)/180) | T | Remanent flux density, x component | Domain 19 |
| mfnc.Bry | BB\*sin(pi\*(180+corr\_b)/180) | T | Remanent flux density, y component | Domain 19 |
| mfnc.Brz | 0 | T | Remanent flux density, z component | Domain 19 |
| mfnc.normBr | sqrt(realdot(mfnc.Brx,mfnc.Brx)+realdot(mfnc.Bry,mfnc.Bry)+realdot(mfnc.Brz,mfnc.Brz)) | T | Remanent flux density norm | Domain 19 |
| mfnc.unTx | 0 | Pa | Maxwell upward surface stress tensor, x component | Boundaries 159–163, 166–167, 172–173, 175 |
| mfnc.unTy | 0 | Pa | Maxwell upward surface stress tensor, y component | Boundaries 159–163, 166–167, 172–173, 175 |
| mfnc.unTz | 0 | Pa | Maxwell upward surface stress tensor, z component | Boundaries 159–163, 166–167, 172–173, 175 |
| mfnc.dnTx | mfnc.dnTmx | Pa | Maxwell downward surface stress tensor, x component | Boundaries 159–163, 166–167, 172–173, 175 |
| mfnc.dnTy | mfnc.dnTmy | Pa | Maxwell downward surface stress tensor, y component | Boundaries 159–163, 166–167, 172–173, 175 |
| mfnc.dnTz | mfnc.dnTmz | Pa | Maxwell downward surface stress tensor, z component | Boundaries 159–163, 166–167, 172–173, 175 |
| mfnc.unx | unx |  | Normal vector up direction, x component | Boundaries 159–163, 166–167, 172–173, 175 |
| mfnc.uny | uny |  | Normal vector up direction, y component | Boundaries 159–163, 166–167, 172–173, 175 |
| mfnc.unz | unz |  | Normal vector up direction, z component | Boundaries 159–163, 166–167, 172–173, 175 |
| mfnc.dnx | dnx |  | Normal vector down direction, x component | Boundaries 159–163, 166–167, 172–173, 175 |
| mfnc.dny | dny |  | Normal vector down direction, y component | Boundaries 159–163, 166–167, 172–173, 175 |
| mfnc.dnz | dnz |  | Normal vector down direction, z component | Boundaries 159–163, 166–167, 172–173, 175 |
| mfnc.W | mfnc.Wm | J/m^3 | Energy density | Domain 19 |
| mfnc.dWm | mfnc.Wm | J/m^3 | Integrand for total magnetic energy | Domain 19 |
| mfnc.Wm | 0.5\*mu0\_const\*((mfnc.murxx\*mfnc.Hx+mfnc.murxy\*mfnc.Hy+mfnc.murxz\*mfnc.Hz)\*mfnc.Hx+(mfnc.muryx\*mfnc.Hx+mfnc.muryy\*mfnc.Hy+mfnc.muryz\*mfnc.Hz)\*mfnc.Hy+(mfnc.murzx\*mfnc.Hx+mfnc.murzy\*mfnc.Hy+mfnc.murzz\*mfnc.Hz)\*mfnc.Hz) | J/m^3 | Magnetic energy density | Domain 19 |

##### Shape Functions

| Name | Shape function | Unit | Description | Shape frame | Selection |
| --- | --- | --- | --- | --- | --- |
| Vm | Lagrange (Quadratic) | A | Magnetic scalar potential | Material | Domain 19 |

##### Weak Expressions

| Weak expression | Integration frame | Selection |
| --- | --- | --- |
| mfnc.d\*(-mfnc.Bx\*test(Vmx)-mfnc.By\*test(Vmy)-mfnc.Bz\*test(Vmz)) | Material | Domain 19 |

#### 2.4.8. ArrayBMagnet5

ArrayBMagnet5

Selection

| Geometric entity level | Domain |
| Selection | Domain 12 |

Equations

Settings

| Description | Value |
| Constitutive relation | Remanent flux density |
| Remanent flux density, x component | BB\*cos(2\*pi/360\*(4\*60 + corr\_b)) |
| Remanent flux density, y component | BB\*sin(2\*pi/360\*(4\*60 + corr\_b)) |
| Remanent flux density, z component | 0 |
| Relative permeability | From material |
| Relative permeability | {{1, 0, 0}, {0, 1, 0}, {0, 0, 1}} |

Properties from material

| Property | Material | Property group |
| Relative permeability | Soft Iron (without losses) | Basic |

##### Variables

| Name | Expression | Unit | Description | Selection |
| --- | --- | --- | --- | --- |
| mfnc.dnTmx | -0.5\*mfnc.unx\*(real(down(mfnc.Bx))\*real(down(mfnc.Hx))+real(down(mfnc.By))\*real(down(mfnc.Hy))+real(down(mfnc.Bz))\*real(down(mfnc.Hz)))+real(down(mfnc.Bx))\*(real(down(mfnc.Hx))\*mfnc.unx+real(down(mfnc.Hy))\*mfnc.uny+real(down(mfnc.Hz))\*mfnc.unz) | Pa | Maxwell downward magnetic surface stress tensor, x component | Boundaries 98–101, 103, 107, 109, 111, 113, 115 |
| mfnc.dnTmy | -0.5\*mfnc.uny\*(real(down(mfnc.Bx))\*real(down(mfnc.Hx))+real(down(mfnc.By))\*real(down(mfnc.Hy))+real(down(mfnc.Bz))\*real(down(mfnc.Hz)))+real(down(mfnc.By))\*(real(down(mfnc.Hx))\*mfnc.unx+real(down(mfnc.Hy))\*mfnc.uny+real(down(mfnc.Hz))\*mfnc.unz) | Pa | Maxwell downward magnetic surface stress tensor, y component | Boundaries 98–101, 103, 107, 109, 111, 113, 115 |
| mfnc.dnTmz | -0.5\*mfnc.unz\*(real(down(mfnc.Bx))\*real(down(mfnc.Hx))+real(down(mfnc.By))\*real(down(mfnc.Hy))+real(down(mfnc.Bz))\*real(down(mfnc.Hz)))+real(down(mfnc.Bz))\*(real(down(mfnc.Hx))\*mfnc.unx+real(down(mfnc.Hy))\*mfnc.uny+real(down(mfnc.Hz))\*mfnc.unz) | Pa | Maxwell downward magnetic surface stress tensor, z component | Boundaries 98–101, 103, 107, 109, 111, 113, 115 |
| mfnc.Hx | -Vmx | A/m | Magnetic field, x component | Domain 12 |
| mfnc.Hy | -Vmy | A/m | Magnetic field, y component | Domain 12 |
| mfnc.Hz | -Vmz | A/m | Magnetic field, z component | Domain 12 |
| mfnc.tHx | -VmTx | A/m | Tangential magnetic field, x component | Boundaries 98–101, 103, 107, 109, 111, 113, 115 |
| mfnc.tHy | -VmTy | A/m | Tangential magnetic field, y component | Boundaries 98–101, 103, 107, 109, 111, 113, 115 |
| mfnc.tHz | -VmTz | A/m | Tangential magnetic field, z component | Boundaries 98–101, 103, 107, 109, 111, 113, 115 |
| mfnc.normH | sqrt(realdot(mfnc.Hx,mfnc.Hx)+realdot(mfnc.Hy,mfnc.Hy)+realdot(mfnc.Hz,mfnc.Hz)) | A/m | Magnetic field norm | Domain 12 |
| mfnc.murxx | model.input.mur11 | 1 | Relative permeability, xx component | Domain 12 |
| mfnc.muryx | model.input.mur21 | 1 | Relative permeability, yx component | Domain 12 |
| mfnc.murzx | model.input.mur31 | 1 | Relative permeability, zx component | Domain 12 |
| mfnc.murxy | model.input.mur12 | 1 | Relative permeability, xy component | Domain 12 |
| mfnc.muryy | model.input.mur22 | 1 | Relative permeability, yy component | Domain 12 |
| mfnc.murzy | model.input.mur32 | 1 | Relative permeability, zy component | Domain 12 |
| mfnc.murxz | model.input.mur13 | 1 | Relative permeability, xz component | Domain 12 |
| mfnc.muryz | model.input.mur23 | 1 | Relative permeability, yz component | Domain 12 |
| mfnc.murzz | model.input.mur33 | 1 | Relative permeability, zz component | Domain 12 |
| mfnc.Bx | mu0\_const\*mfnc.Ixx\*mfnc.Hx+mu0\_const\*mfnc.Ixy\*mfnc.Hy+mu0\_const\*mfnc.Ixz\*mfnc.Hz+mu0\_const\*mfnc.chimxx\*mfnc.Hx+mu0\_const\*mfnc.chimxy\*mfnc.Hy+mu0\_const\*mfnc.chimxz\*mfnc.Hz+mfnc.Brx | T | Magnetic flux density, x component | Domain 12 |
| mfnc.By | mu0\_const\*mfnc.Iyx\*mfnc.Hx+mu0\_const\*mfnc.Iyy\*mfnc.Hy+mu0\_const\*mfnc.Iyz\*mfnc.Hz+mu0\_const\*mfnc.chimyx\*mfnc.Hx+mu0\_const\*mfnc.chimyy\*mfnc.Hy+mu0\_const\*mfnc.chimyz\*mfnc.Hz+mfnc.Bry | T | Magnetic flux density, y component | Domain 12 |
| mfnc.Bz | mu0\_const\*mfnc.Izx\*mfnc.Hx+mu0\_const\*mfnc.Izy\*mfnc.Hy+mu0\_const\*mfnc.Izz\*mfnc.Hz+mu0\_const\*mfnc.chimzx\*mfnc.Hx+mu0\_const\*mfnc.chimzy\*mfnc.Hy+mu0\_const\*mfnc.chimzz\*mfnc.Hz+mfnc.Brz | T | Magnetic flux density, z component | Domain 12 |
| mfnc.normB | sqrt(realdot(mfnc.Bx,mfnc.Bx)+realdot(mfnc.By,mfnc.By)+realdot(mfnc.Bz,mfnc.Bz)) | T | Magnetic flux density norm | Domain 12 |
| mfnc.Mx | mfnc.Bx/mu0\_const-mfnc.Ixx\*mfnc.Hx-mfnc.Ixy\*mfnc.Hy-mfnc.Ixz\*mfnc.Hz | A/m | Magnetization, x component | Domain 12 |
| mfnc.My | mfnc.By/mu0\_const-mfnc.Iyx\*mfnc.Hx-mfnc.Iyy\*mfnc.Hy-mfnc.Iyz\*mfnc.Hz | A/m | Magnetization, y component | Domain 12 |
| mfnc.Mz | mfnc.Bz/mu0\_const-mfnc.Izx\*mfnc.Hx-mfnc.Izy\*mfnc.Hy-mfnc.Izz\*mfnc.Hz | A/m | Magnetization, z component | Domain 12 |
| mfnc.normM | sqrt(realdot(mfnc.Mx,mfnc.Mx)+realdot(mfnc.My,mfnc.My)+realdot(mfnc.Mz,mfnc.Mz)) | A/m | Magnetization norm | Domain 12 |
| mfnc.Ixx | 1 | 1 | Spatial identity matrix, xx component | Domain 12 |
| mfnc.Iyx | 0 | 1 | Spatial identity matrix, yx component | Domain 12 |
| mfnc.Izx | 0 | 1 | Spatial identity matrix, zx component | Domain 12 |
| mfnc.Ixy | 0 | 1 | Spatial identity matrix, xy component | Domain 12 |
| mfnc.Iyy | 1 | 1 | Spatial identity matrix, yy component | Domain 12 |
| mfnc.Izy | 0 | 1 | Spatial identity matrix, zy component | Domain 12 |
| mfnc.Ixz | 0 | 1 | Spatial identity matrix, xz component | Domain 12 |
| mfnc.Iyz | 0 | 1 | Spatial identity matrix, yz component | Domain 12 |
| mfnc.Izz | 1 | 1 | Spatial identity matrix, zz component | Domain 12 |
| mfnc.chimxx | -1+mfnc.murxx | 1 | Magnetic susceptibility, xx component | Domain 12 |
| mfnc.chimyx | mfnc.muryx | 1 | Magnetic susceptibility, yx component | Domain 12 |
| mfnc.chimzx | mfnc.murzx | 1 | Magnetic susceptibility, zx component | Domain 12 |
| mfnc.chimxy | mfnc.murxy | 1 | Magnetic susceptibility, xy component | Domain 12 |
| mfnc.chimyy | -1+mfnc.muryy | 1 | Magnetic susceptibility, yy component | Domain 12 |
| mfnc.chimzy | mfnc.murzy | 1 | Magnetic susceptibility, zy component | Domain 12 |
| mfnc.chimxz | mfnc.murxz | 1 | Magnetic susceptibility, xz component | Domain 12 |
| mfnc.chimyz | mfnc.muryz | 1 | Magnetic susceptibility, yz component | Domain 12 |
| mfnc.chimzz | -1+mfnc.murzz | 1 | Magnetic susceptibility, zz component | Domain 12 |
| mfnc.Brx | BB\*cos(pi\*(240+corr\_b)/180) | T | Remanent flux density, x component | Domain 12 |
| mfnc.Bry | BB\*sin(pi\*(240+corr\_b)/180) | T | Remanent flux density, y component | Domain 12 |
| mfnc.Brz | 0 | T | Remanent flux density, z component | Domain 12 |
| mfnc.normBr | sqrt(realdot(mfnc.Brx,mfnc.Brx)+realdot(mfnc.Bry,mfnc.Bry)+realdot(mfnc.Brz,mfnc.Brz)) | T | Remanent flux density norm | Domain 12 |
| mfnc.unTx | 0 | Pa | Maxwell upward surface stress tensor, x component | Boundaries 98–101, 103, 107, 109, 111, 113, 115 |
| mfnc.unTy | 0 | Pa | Maxwell upward surface stress tensor, y component | Boundaries 98–101, 103, 107, 109, 111, 113, 115 |
| mfnc.unTz | 0 | Pa | Maxwell upward surface stress tensor, z component | Boundaries 98–101, 103, 107, 109, 111, 113, 115 |
| mfnc.dnTx | mfnc.dnTmx | Pa | Maxwell downward surface stress tensor, x component | Boundaries 98–101, 103, 107, 109, 111, 113, 115 |
| mfnc.dnTy | mfnc.dnTmy | Pa | Maxwell downward surface stress tensor, y component | Boundaries 98–101, 103, 107, 109, 111, 113, 115 |
| mfnc.dnTz | mfnc.dnTmz | Pa | Maxwell downward surface stress tensor, z component | Boundaries 98–101, 103, 107, 109, 111, 113, 115 |
| mfnc.unx | unx |  | Normal vector up direction, x component | Boundaries 98–101, 103, 107, 109, 111, 113, 115 |
| mfnc.uny | uny |  | Normal vector up direction, y component | Boundaries 98–101, 103, 107, 109, 111, 113, 115 |
| mfnc.unz | unz |  | Normal vector up direction, z component | Boundaries 98–101, 103, 107, 109, 111, 113, 115 |
| mfnc.dnx | dnx |  | Normal vector down direction, x component | Boundaries 98–101, 103, 107, 109, 111, 113, 115 |
| mfnc.dny | dny |  | Normal vector down direction, y component | Boundaries 98–101, 103, 107, 109, 111, 113, 115 |
| mfnc.dnz | dnz |  | Normal vector down direction, z component | Boundaries 98–101, 103, 107, 109, 111, 113, 115 |
| mfnc.W | mfnc.Wm | J/m^3 | Energy density | Domain 12 |
| mfnc.dWm | mfnc.Wm | J/m^3 | Integrand for total magnetic energy | Domain 12 |
| mfnc.Wm | 0.5\*mu0\_const\*((mfnc.murxx\*mfnc.Hx+mfnc.murxy\*mfnc.Hy+mfnc.murxz\*mfnc.Hz)\*mfnc.Hx+(mfnc.muryx\*mfnc.Hx+mfnc.muryy\*mfnc.Hy+mfnc.muryz\*mfnc.Hz)\*mfnc.Hy+(mfnc.murzx\*mfnc.Hx+mfnc.murzy\*mfnc.Hy+mfnc.murzz\*mfnc.Hz)\*mfnc.Hz) | J/m^3 | Magnetic energy density | Domain 12 |

##### Shape Functions

| Name | Shape function | Unit | Description | Shape frame | Selection |
| --- | --- | --- | --- | --- | --- |
| Vm | Lagrange (Quadratic) | A | Magnetic scalar potential | Material | Domain 12 |

##### Weak Expressions

| Weak expression | Integration frame | Selection |
| --- | --- | --- |
| mfnc.d\*(-mfnc.Bx\*test(Vmx)-mfnc.By\*test(Vmy)-mfnc.Bz\*test(Vmz)) | Material | Domain 12 |

#### 2.4.9. ArrayBMagnet6

ArrayBMagnet6

Selection

| Geometric entity level | Domain |
| Selection | Domain 7 |

Equations

Settings

| Description | Value |
| Constitutive relation | Remanent flux density |
| Remanent flux density, x component | BB\*cos(2\*pi/360\*(5\*60 + corr\_b)) |
| Remanent flux density, y component | BB\*sin(2\*pi/360\*(5\*60 + corr\_b)) |
| Remanent flux density, z component | 0 |
| Relative permeability | From material |
| Relative permeability | {{1, 0, 0}, {0, 1, 0}, {0, 0, 1}} |

Properties from material

| Property | Material | Property group |
| Relative permeability | Soft Iron (without losses) | Basic |

##### Variables

| Name | Expression | Unit | Description | Selection |
| --- | --- | --- | --- | --- |
| mfnc.dnTmx | -0.5\*mfnc.unx\*(real(down(mfnc.Bx))\*real(down(mfnc.Hx))+real(down(mfnc.By))\*real(down(mfnc.Hy))+real(down(mfnc.Bz))\*real(down(mfnc.Hz)))+real(down(mfnc.Bx))\*(real(down(mfnc.Hx))\*mfnc.unx+real(down(mfnc.Hy))\*mfnc.uny+real(down(mfnc.Hz))\*mfnc.unz) | Pa | Maxwell downward magnetic surface stress tensor, x component | Boundaries 50–53, 55, 57, 59, 71, 73, 75 |
| mfnc.dnTmy | -0.5\*mfnc.uny\*(real(down(mfnc.Bx))\*real(down(mfnc.Hx))+real(down(mfnc.By))\*real(down(mfnc.Hy))+real(down(mfnc.Bz))\*real(down(mfnc.Hz)))+real(down(mfnc.By))\*(real(down(mfnc.Hx))\*mfnc.unx+real(down(mfnc.Hy))\*mfnc.uny+real(down(mfnc.Hz))\*mfnc.unz) | Pa | Maxwell downward magnetic surface stress tensor, y component | Boundaries 50–53, 55, 57, 59, 71, 73, 75 |
| mfnc.dnTmz | -0.5\*mfnc.unz\*(real(down(mfnc.Bx))\*real(down(mfnc.Hx))+real(down(mfnc.By))\*real(down(mfnc.Hy))+real(down(mfnc.Bz))\*real(down(mfnc.Hz)))+real(down(mfnc.Bz))\*(real(down(mfnc.Hx))\*mfnc.unx+real(down(mfnc.Hy))\*mfnc.uny+real(down(mfnc.Hz))\*mfnc.unz) | Pa | Maxwell downward magnetic surface stress tensor, z component | Boundaries 50–53, 55, 57, 59, 71, 73, 75 |
| mfnc.Hx | -Vmx | A/m | Magnetic field, x component | Domain 7 |
| mfnc.Hy | -Vmy | A/m | Magnetic field, y component | Domain 7 |
| mfnc.Hz | -Vmz | A/m | Magnetic field, z component | Domain 7 |
| mfnc.tHx | -VmTx | A/m | Tangential magnetic field, x component | Boundaries 50–53, 55, 57, 59, 71, 73, 75 |
| mfnc.tHy | -VmTy | A/m | Tangential magnetic field, y component | Boundaries 50–53, 55, 57, 59, 71, 73, 75 |
| mfnc.tHz | -VmTz | A/m | Tangential magnetic field, z component | Boundaries 50–53, 55, 57, 59, 71, 73, 75 |
| mfnc.normH | sqrt(realdot(mfnc.Hx,mfnc.Hx)+realdot(mfnc.Hy,mfnc.Hy)+realdot(mfnc.Hz,mfnc.Hz)) | A/m | Magnetic field norm | Domain 7 |
| mfnc.murxx | model.input.mur11 | 1 | Relative permeability, xx component | Domain 7 |
| mfnc.muryx | model.input.mur21 | 1 | Relative permeability, yx component | Domain 7 |
| mfnc.murzx | model.input.mur31 | 1 | Relative permeability, zx component | Domain 7 |
| mfnc.murxy | model.input.mur12 | 1 | Relative permeability, xy component | Domain 7 |
| mfnc.muryy | model.input.mur22 | 1 | Relative permeability, yy component | Domain 7 |
| mfnc.murzy | model.input.mur32 | 1 | Relative permeability, zy component | Domain 7 |
| mfnc.murxz | model.input.mur13 | 1 | Relative permeability, xz component | Domain 7 |
| mfnc.muryz | model.input.mur23 | 1 | Relative permeability, yz component | Domain 7 |
| mfnc.murzz | model.input.mur33 | 1 | Relative permeability, zz component | Domain 7 |
| mfnc.Bx | mu0\_const\*mfnc.Ixx\*mfnc.Hx+mu0\_const\*mfnc.Ixy\*mfnc.Hy+mu0\_const\*mfnc.Ixz\*mfnc.Hz+mu0\_const\*mfnc.chimxx\*mfnc.Hx+mu0\_const\*mfnc.chimxy\*mfnc.Hy+mu0\_const\*mfnc.chimxz\*mfnc.Hz+mfnc.Brx | T | Magnetic flux density, x component | Domain 7 |
| mfnc.By | mu0\_const\*mfnc.Iyx\*mfnc.Hx+mu0\_const\*mfnc.Iyy\*mfnc.Hy+mu0\_const\*mfnc.Iyz\*mfnc.Hz+mu0\_const\*mfnc.chimyx\*mfnc.Hx+mu0\_const\*mfnc.chimyy\*mfnc.Hy+mu0\_const\*mfnc.chimyz\*mfnc.Hz+mfnc.Bry | T | Magnetic flux density, y component | Domain 7 |
| mfnc.Bz | mu0\_const\*mfnc.Izx\*mfnc.Hx+mu0\_const\*mfnc.Izy\*mfnc.Hy+mu0\_const\*mfnc.Izz\*mfnc.Hz+mu0\_const\*mfnc.chimzx\*mfnc.Hx+mu0\_const\*mfnc.chimzy\*mfnc.Hy+mu0\_const\*mfnc.chimzz\*mfnc.Hz+mfnc.Brz | T | Magnetic flux density, z component | Domain 7 |
| mfnc.normB | sqrt(realdot(mfnc.Bx,mfnc.Bx)+realdot(mfnc.By,mfnc.By)+realdot(mfnc.Bz,mfnc.Bz)) | T | Magnetic flux density norm | Domain 7 |
| mfnc.Mx | mfnc.Bx/mu0\_const-mfnc.Ixx\*mfnc.Hx-mfnc.Ixy\*mfnc.Hy-mfnc.Ixz\*mfnc.Hz | A/m | Magnetization, x component | Domain 7 |
| mfnc.My | mfnc.By/mu0\_const-mfnc.Iyx\*mfnc.Hx-mfnc.Iyy\*mfnc.Hy-mfnc.Iyz\*mfnc.Hz | A/m | Magnetization, y component | Domain 7 |
| mfnc.Mz | mfnc.Bz/mu0\_const-mfnc.Izx\*mfnc.Hx-mfnc.Izy\*mfnc.Hy-mfnc.Izz\*mfnc.Hz | A/m | Magnetization, z component | Domain 7 |
| mfnc.normM | sqrt(realdot(mfnc.Mx,mfnc.Mx)+realdot(mfnc.My,mfnc.My)+realdot(mfnc.Mz,mfnc.Mz)) | A/m | Magnetization norm | Domain 7 |
| mfnc.Ixx | 1 | 1 | Spatial identity matrix, xx component | Domain 7 |
| mfnc.Iyx | 0 | 1 | Spatial identity matrix, yx component | Domain 7 |
| mfnc.Izx | 0 | 1 | Spatial identity matrix, zx component | Domain 7 |
| mfnc.Ixy | 0 | 1 | Spatial identity matrix, xy component | Domain 7 |
| mfnc.Iyy | 1 | 1 | Spatial identity matrix, yy component | Domain 7 |
| mfnc.Izy | 0 | 1 | Spatial identity matrix, zy component | Domain 7 |
| mfnc.Ixz | 0 | 1 | Spatial identity matrix, xz component | Domain 7 |
| mfnc.Iyz | 0 | 1 | Spatial identity matrix, yz component | Domain 7 |
| mfnc.Izz | 1 | 1 | Spatial identity matrix, zz component | Domain 7 |
| mfnc.chimxx | -1+mfnc.murxx | 1 | Magnetic susceptibility, xx component | Domain 7 |
| mfnc.chimyx | mfnc.muryx | 1 | Magnetic susceptibility, yx component | Domain 7 |
| mfnc.chimzx | mfnc.murzx | 1 | Magnetic susceptibility, zx component | Domain 7 |
| mfnc.chimxy | mfnc.murxy | 1 | Magnetic susceptibility, xy component | Domain 7 |
| mfnc.chimyy | -1+mfnc.muryy | 1 | Magnetic susceptibility, yy component | Domain 7 |
| mfnc.chimzy | mfnc.murzy | 1 | Magnetic susceptibility, zy component | Domain 7 |
| mfnc.chimxz | mfnc.murxz | 1 | Magnetic susceptibility, xz component | Domain 7 |
| mfnc.chimyz | mfnc.muryz | 1 | Magnetic susceptibility, yz component | Domain 7 |
| mfnc.chimzz | -1+mfnc.murzz | 1 | Magnetic susceptibility, zz component | Domain 7 |
| mfnc.Brx | BB\*cos(pi\*(300+corr\_b)/180) | T | Remanent flux density, x component | Domain 7 |
| mfnc.Bry | BB\*sin(pi\*(300+corr\_b)/180) | T | Remanent flux density, y component | Domain 7 |
| mfnc.Brz | 0 | T | Remanent flux density, z component | Domain 7 |
| mfnc.normBr | sqrt(realdot(mfnc.Brx,mfnc.Brx)+realdot(mfnc.Bry,mfnc.Bry)+realdot(mfnc.Brz,mfnc.Brz)) | T | Remanent flux density norm | Domain 7 |
| mfnc.unTx | 0 | Pa | Maxwell upward surface stress tensor, x component | Boundaries 50–53, 55, 57, 59, 71, 73, 75 |
| mfnc.unTy | 0 | Pa | Maxwell upward surface stress tensor, y component | Boundaries 50–53, 55, 57, 59, 71, 73, 75 |
| mfnc.unTz | 0 | Pa | Maxwell upward surface stress tensor, z component | Boundaries 50–53, 55, 57, 59, 71, 73, 75 |
| mfnc.dnTx | mfnc.dnTmx | Pa | Maxwell downward surface stress tensor, x component | Boundaries 50–53, 55, 57, 59, 71, 73, 75 |
| mfnc.dnTy | mfnc.dnTmy | Pa | Maxwell downward surface stress tensor, y component | Boundaries 50–53, 55, 57, 59, 71, 73, 75 |
| mfnc.dnTz | mfnc.dnTmz | Pa | Maxwell downward surface stress tensor, z component | Boundaries 50–53, 55, 57, 59, 71, 73, 75 |
| mfnc.unx | unx |  | Normal vector up direction, x component | Boundaries 50–53, 55, 57, 59, 71, 73, 75 |
| mfnc.uny | uny |  | Normal vector up direction, y component | Boundaries 50–53, 55, 57, 59, 71, 73, 75 |
| mfnc.unz | unz |  | Normal vector up direction, z component | Boundaries 50–53, 55, 57, 59, 71, 73, 75 |
| mfnc.dnx | dnx |  | Normal vector down direction, x component | Boundaries 50–53, 55, 57, 59, 71, 73, 75 |
| mfnc.dny | dny |  | Normal vector down direction, y component | Boundaries 50–53, 55, 57, 59, 71, 73, 75 |
| mfnc.dnz | dnz |  | Normal vector down direction, z component | Boundaries 50–53, 55, 57, 59, 71, 73, 75 |
| mfnc.W | mfnc.Wm | J/m^3 | Energy density | Domain 7 |
| mfnc.dWm | mfnc.Wm | J/m^3 | Integrand for total magnetic energy | Domain 7 |
| mfnc.Wm | 0.5\*mu0\_const\*((mfnc.murxx\*mfnc.Hx+mfnc.murxy\*mfnc.Hy+mfnc.murxz\*mfnc.Hz)\*mfnc.Hx+(mfnc.muryx\*mfnc.Hx+mfnc.muryy\*mfnc.Hy+mfnc.muryz\*mfnc.Hz)\*mfnc.Hy+(mfnc.murzx\*mfnc.Hx+mfnc.murzy\*mfnc.Hy+mfnc.murzz\*mfnc.Hz)\*mfnc.Hz) | J/m^3 | Magnetic energy density | Domain 7 |

##### Shape Functions

| Name | Shape function | Unit | Description | Shape frame | Selection |
| --- | --- | --- | --- | --- | --- |
| Vm | Lagrange (Quadratic) | A | Magnetic scalar potential | Material | Domain 7 |

##### Weak Expressions

| Weak expression | Integration frame | Selection |
| --- | --- | --- |
| mfnc.d\*(-mfnc.Bx\*test(Vmx)-mfnc.By\*test(Vmy)-mfnc.Bz\*test(Vmz)) | Material | Domain 7 |

#### 2.4.10. ArrayBMagnet7

ArrayBMagnet7

Selection

| Geometric entity level | Domain |
| Selection | Domain 5 |

Equations

Settings

| Description | Value |
| Constitutive relation | Remanent flux density |
| Remanent flux density, x component | BB\*cos(2\*pi/360\*(6\*60 + corr\_b)) |
| Remanent flux density, y component | BB\*sin(2\*pi/360\*(6\*60 + corr\_b)) |
| Remanent flux density, z component | 0 |
| Relative permeability | From material |
| Relative permeability | {{1, 0, 0}, {0, 1, 0}, {0, 0, 1}} |

Properties from material

| Property | Material | Property group |
| Relative permeability | Soft Iron (without losses) | Basic |

##### Variables

| Name | Expression | Unit | Description | Selection |
| --- | --- | --- | --- | --- |
| mfnc.dnTmx | -0.5\*mfnc.unx\*(real(down(mfnc.Bx))\*real(down(mfnc.Hx))+real(down(mfnc.By))\*real(down(mfnc.Hy))+real(down(mfnc.Bz))\*real(down(mfnc.Hz)))+real(down(mfnc.Bx))\*(real(down(mfnc.Hx))\*mfnc.unx+real(down(mfnc.Hy))\*mfnc.uny+real(down(mfnc.Hz))\*mfnc.unz) | Pa | Maxwell downward magnetic surface stress tensor, x component | Boundaries 36–45 |
| mfnc.dnTmy | -0.5\*mfnc.uny\*(real(down(mfnc.Bx))\*real(down(mfnc.Hx))+real(down(mfnc.By))\*real(down(mfnc.Hy))+real(down(mfnc.Bz))\*real(down(mfnc.Hz)))+real(down(mfnc.By))\*(real(down(mfnc.Hx))\*mfnc.unx+real(down(mfnc.Hy))\*mfnc.uny+real(down(mfnc.Hz))\*mfnc.unz) | Pa | Maxwell downward magnetic surface stress tensor, y component | Boundaries 36–45 |
| mfnc.dnTmz | -0.5\*mfnc.unz\*(real(down(mfnc.Bx))\*real(down(mfnc.Hx))+real(down(mfnc.By))\*real(down(mfnc.Hy))+real(down(mfnc.Bz))\*real(down(mfnc.Hz)))+real(down(mfnc.Bz))\*(real(down(mfnc.Hx))\*mfnc.unx+real(down(mfnc.Hy))\*mfnc.uny+real(down(mfnc.Hz))\*mfnc.unz) | Pa | Maxwell downward magnetic surface stress tensor, z component | Boundaries 36–45 |
| mfnc.Hx | -Vmx | A/m | Magnetic field, x component | Domain 5 |
| mfnc.Hy | -Vmy | A/m | Magnetic field, y component | Domain 5 |
| mfnc.Hz | -Vmz | A/m | Magnetic field, z component | Domain 5 |
| mfnc.tHx | -VmTx | A/m | Tangential magnetic field, x component | Boundaries 36–45 |
| mfnc.tHy | -VmTy | A/m | Tangential magnetic field, y component | Boundaries 36–45 |
| mfnc.tHz | -VmTz | A/m | Tangential magnetic field, z component | Boundaries 36–45 |
| mfnc.normH | sqrt(realdot(mfnc.Hx,mfnc.Hx)+realdot(mfnc.Hy,mfnc.Hy)+realdot(mfnc.Hz,mfnc.Hz)) | A/m | Magnetic field norm | Domain 5 |
| mfnc.murxx | model.input.mur11 | 1 | Relative permeability, xx component | Domain 5 |
| mfnc.muryx | model.input.mur21 | 1 | Relative permeability, yx component | Domain 5 |
| mfnc.murzx | model.input.mur31 | 1 | Relative permeability, zx component | Domain 5 |
| mfnc.murxy | model.input.mur12 | 1 | Relative permeability, xy component | Domain 5 |
| mfnc.muryy | model.input.mur22 | 1 | Relative permeability, yy component | Domain 5 |
| mfnc.murzy | model.input.mur32 | 1 | Relative permeability, zy component | Domain 5 |
| mfnc.murxz | model.input.mur13 | 1 | Relative permeability, xz component | Domain 5 |
| mfnc.muryz | model.input.mur23 | 1 | Relative permeability, yz component | Domain 5 |
| mfnc.murzz | model.input.mur33 | 1 | Relative permeability, zz component | Domain 5 |
| mfnc.Bx | mu0\_const\*mfnc.Ixx\*mfnc.Hx+mu0\_const\*mfnc.Ixy\*mfnc.Hy+mu0\_const\*mfnc.Ixz\*mfnc.Hz+mu0\_const\*mfnc.chimxx\*mfnc.Hx+mu0\_const\*mfnc.chimxy\*mfnc.Hy+mu0\_const\*mfnc.chimxz\*mfnc.Hz+mfnc.Brx | T | Magnetic flux density, x component | Domain 5 |
| mfnc.By | mu0\_const\*mfnc.Iyx\*mfnc.Hx+mu0\_const\*mfnc.Iyy\*mfnc.Hy+mu0\_const\*mfnc.Iyz\*mfnc.Hz+mu0\_const\*mfnc.chimyx\*mfnc.Hx+mu0\_const\*mfnc.chimyy\*mfnc.Hy+mu0\_const\*mfnc.chimyz\*mfnc.Hz+mfnc.Bry | T | Magnetic flux density, y component | Domain 5 |
| mfnc.Bz | mu0\_const\*mfnc.Izx\*mfnc.Hx+mu0\_const\*mfnc.Izy\*mfnc.Hy+mu0\_const\*mfnc.Izz\*mfnc.Hz+mu0\_const\*mfnc.chimzx\*mfnc.Hx+mu0\_const\*mfnc.chimzy\*mfnc.Hy+mu0\_const\*mfnc.chimzz\*mfnc.Hz+mfnc.Brz | T | Magnetic flux density, z component | Domain 5 |
| mfnc.normB | sqrt(realdot(mfnc.Bx,mfnc.Bx)+realdot(mfnc.By,mfnc.By)+realdot(mfnc.Bz,mfnc.Bz)) | T | Magnetic flux density norm | Domain 5 |
| mfnc.Mx | mfnc.Bx/mu0\_const-mfnc.Ixx\*mfnc.Hx-mfnc.Ixy\*mfnc.Hy-mfnc.Ixz\*mfnc.Hz | A/m | Magnetization, x component | Domain 5 |
| mfnc.My | mfnc.By/mu0\_const-mfnc.Iyx\*mfnc.Hx-mfnc.Iyy\*mfnc.Hy-mfnc.Iyz\*mfnc.Hz | A/m | Magnetization, y component | Domain 5 |
| mfnc.Mz | mfnc.Bz/mu0\_const-mfnc.Izx\*mfnc.Hx-mfnc.Izy\*mfnc.Hy-mfnc.Izz\*mfnc.Hz | A/m | Magnetization, z component | Domain 5 |
| mfnc.normM | sqrt(realdot(mfnc.Mx,mfnc.Mx)+realdot(mfnc.My,mfnc.My)+realdot(mfnc.Mz,mfnc.Mz)) | A/m | Magnetization norm | Domain 5 |
| mfnc.Ixx | 1 | 1 | Spatial identity matrix, xx component | Domain 5 |
| mfnc.Iyx | 0 | 1 | Spatial identity matrix, yx component | Domain 5 |
| mfnc.Izx | 0 | 1 | Spatial identity matrix, zx component | Domain 5 |
| mfnc.Ixy | 0 | 1 | Spatial identity matrix, xy component | Domain 5 |
| mfnc.Iyy | 1 | 1 | Spatial identity matrix, yy component | Domain 5 |
| mfnc.Izy | 0 | 1 | Spatial identity matrix, zy component | Domain 5 |
| mfnc.Ixz | 0 | 1 | Spatial identity matrix, xz component | Domain 5 |
| mfnc.Iyz | 0 | 1 | Spatial identity matrix, yz component | Domain 5 |
| mfnc.Izz | 1 | 1 | Spatial identity matrix, zz component | Domain 5 |
| mfnc.chimxx | -1+mfnc.murxx | 1 | Magnetic susceptibility, xx component | Domain 5 |
| mfnc.chimyx | mfnc.muryx | 1 | Magnetic susceptibility, yx component | Domain 5 |
| mfnc.chimzx | mfnc.murzx | 1 | Magnetic susceptibility, zx component | Domain 5 |
| mfnc.chimxy | mfnc.murxy | 1 | Magnetic susceptibility, xy component | Domain 5 |
| mfnc.chimyy | -1+mfnc.muryy | 1 | Magnetic susceptibility, yy component | Domain 5 |
| mfnc.chimzy | mfnc.murzy | 1 | Magnetic susceptibility, zy component | Domain 5 |
| mfnc.chimxz | mfnc.murxz | 1 | Magnetic susceptibility, xz component | Domain 5 |
| mfnc.chimyz | mfnc.muryz | 1 | Magnetic susceptibility, yz component | Domain 5 |
| mfnc.chimzz | -1+mfnc.murzz | 1 | Magnetic susceptibility, zz component | Domain 5 |
| mfnc.Brx | BB\*cos(pi\*(360+corr\_b)/180) | T | Remanent flux density, x component | Domain 5 |
| mfnc.Bry | BB\*sin(pi\*(360+corr\_b)/180) | T | Remanent flux density, y component | Domain 5 |
| mfnc.Brz | 0 | T | Remanent flux density, z component | Domain 5 |
| mfnc.normBr | sqrt(realdot(mfnc.Brx,mfnc.Brx)+realdot(mfnc.Bry,mfnc.Bry)+realdot(mfnc.Brz,mfnc.Brz)) | T | Remanent flux density norm | Domain 5 |
| mfnc.unTx | 0 | Pa | Maxwell upward surface stress tensor, x component | Boundaries 36–45 |
| mfnc.unTy | 0 | Pa | Maxwell upward surface stress tensor, y component | Boundaries 36–45 |
| mfnc.unTz | 0 | Pa | Maxwell upward surface stress tensor, z component | Boundaries 36–45 |
| mfnc.dnTx | mfnc.dnTmx | Pa | Maxwell downward surface stress tensor, x component | Boundaries 36–45 |
| mfnc.dnTy | mfnc.dnTmy | Pa | Maxwell downward surface stress tensor, y component | Boundaries 36–45 |
| mfnc.dnTz | mfnc.dnTmz | Pa | Maxwell downward surface stress tensor, z component | Boundaries 36–45 |
| mfnc.unx | unx |  | Normal vector up direction, x component | Boundaries 36–45 |
| mfnc.uny | uny |  | Normal vector up direction, y component | Boundaries 36–45 |
| mfnc.unz | unz |  | Normal vector up direction, z component | Boundaries 36–45 |
| mfnc.dnx | dnx |  | Normal vector down direction, x component | Boundaries 36–45 |
| mfnc.dny | dny |  | Normal vector down direction, y component | Boundaries 36–45 |
| mfnc.dnz | dnz |  | Normal vector down direction, z component | Boundaries 36–45 |
| mfnc.W | mfnc.Wm | J/m^3 | Energy density | Domain 5 |
| mfnc.dWm | mfnc.Wm | J/m^3 | Integrand for total magnetic energy | Domain 5 |
| mfnc.Wm | 0.5\*mu0\_const\*((mfnc.murxx\*mfnc.Hx+mfnc.murxy\*mfnc.Hy+mfnc.murxz\*mfnc.Hz)\*mfnc.Hx+(mfnc.muryx\*mfnc.Hx+mfnc.muryy\*mfnc.Hy+mfnc.muryz\*mfnc.Hz)\*mfnc.Hy+(mfnc.murzx\*mfnc.Hx+mfnc.murzy\*mfnc.Hy+mfnc.murzz\*mfnc.Hz)\*mfnc.Hz) | J/m^3 | Magnetic energy density | Domain 5 |

##### Shape Functions

| Name | Shape function | Unit | Description | Shape frame | Selection |
| --- | --- | --- | --- | --- | --- |
| Vm | Lagrange (Quadratic) | A | Magnetic scalar potential | Material | Domain 5 |

##### Weak Expressions

| Weak expression | Integration frame | Selection |
| --- | --- | --- |
| mfnc.d\*(-mfnc.Bx\*test(Vmx)-mfnc.By\*test(Vmy)-mfnc.Bz\*test(Vmz)) | Material | Domain 5 |

#### 2.4.11. ArrayBMagnet8

ArrayBMagnet8

Selection

| Geometric entity level | Domain |
| Selection | Domain 6 |

Equations

Settings

| Description | Value |
| Constitutive relation | Remanent flux density |
| Remanent flux density, x component | BB\*cos(2\*pi/360\*(7\*60 + corr\_b)) |
| Remanent flux density, y component | BB\*sin(2\*pi/360\*(7\*60 + corr\_b)) |
| Remanent flux density, z component | 0 |
| Relative permeability | From material |
| Relative permeability | {{1, 0, 0}, {0, 1, 0}, {0, 0, 1}} |

Properties from material

| Property | Material | Property group |
| Relative permeability | Soft Iron (without losses) | Basic |

##### Variables

| Name | Expression | Unit | Description | Selection |
| --- | --- | --- | --- | --- |
| mfnc.dnTmx | -0.5\*mfnc.unx\*(real(down(mfnc.Bx))\*real(down(mfnc.Hx))+real(down(mfnc.By))\*real(down(mfnc.Hy))+real(down(mfnc.Bz))\*real(down(mfnc.Hz)))+real(down(mfnc.Bx))\*(real(down(mfnc.Hx))\*mfnc.unx+real(down(mfnc.Hy))\*mfnc.uny+real(down(mfnc.Hz))\*mfnc.unz) | Pa | Maxwell downward magnetic surface stress tensor, x component | Boundaries 46–49, 54, 56, 58, 70, 72, 74 |
| mfnc.dnTmy | -0.5\*mfnc.uny\*(real(down(mfnc.Bx))\*real(down(mfnc.Hx))+real(down(mfnc.By))\*real(down(mfnc.Hy))+real(down(mfnc.Bz))\*real(down(mfnc.Hz)))+real(down(mfnc.By))\*(real(down(mfnc.Hx))\*mfnc.unx+real(down(mfnc.Hy))\*mfnc.uny+real(down(mfnc.Hz))\*mfnc.unz) | Pa | Maxwell downward magnetic surface stress tensor, y component | Boundaries 46–49, 54, 56, 58, 70, 72, 74 |
| mfnc.dnTmz | -0.5\*mfnc.unz\*(real(down(mfnc.Bx))\*real(down(mfnc.Hx))+real(down(mfnc.By))\*real(down(mfnc.Hy))+real(down(mfnc.Bz))\*real(down(mfnc.Hz)))+real(down(mfnc.Bz))\*(real(down(mfnc.Hx))\*mfnc.unx+real(down(mfnc.Hy))\*mfnc.uny+real(down(mfnc.Hz))\*mfnc.unz) | Pa | Maxwell downward magnetic surface stress tensor, z component | Boundaries 46–49, 54, 56, 58, 70, 72, 74 |
| mfnc.Hx | -Vmx | A/m | Magnetic field, x component | Domain 6 |
| mfnc.Hy | -Vmy | A/m | Magnetic field, y component | Domain 6 |
| mfnc.Hz | -Vmz | A/m | Magnetic field, z component | Domain 6 |
| mfnc.tHx | -VmTx | A/m | Tangential magnetic field, x component | Boundaries 46–49, 54, 56, 58, 70, 72, 74 |
| mfnc.tHy | -VmTy | A/m | Tangential magnetic field, y component | Boundaries 46–49, 54, 56, 58, 70, 72, 74 |
| mfnc.tHz | -VmTz | A/m | Tangential magnetic field, z component | Boundaries 46–49, 54, 56, 58, 70, 72, 74 |
| mfnc.normH | sqrt(realdot(mfnc.Hx,mfnc.Hx)+realdot(mfnc.Hy,mfnc.Hy)+realdot(mfnc.Hz,mfnc.Hz)) | A/m | Magnetic field norm | Domain 6 |
| mfnc.murxx | model.input.mur11 | 1 | Relative permeability, xx component | Domain 6 |
| mfnc.muryx | model.input.mur21 | 1 | Relative permeability, yx component | Domain 6 |
| mfnc.murzx | model.input.mur31 | 1 | Relative permeability, zx component | Domain 6 |
| mfnc.murxy | model.input.mur12 | 1 | Relative permeability, xy component | Domain 6 |
| mfnc.muryy | model.input.mur22 | 1 | Relative permeability, yy component | Domain 6 |
| mfnc.murzy | model.input.mur32 | 1 | Relative permeability, zy component | Domain 6 |
| mfnc.murxz | model.input.mur13 | 1 | Relative permeability, xz component | Domain 6 |
| mfnc.muryz | model.input.mur23 | 1 | Relative permeability, yz component | Domain 6 |
| mfnc.murzz | model.input.mur33 | 1 | Relative permeability, zz component | Domain 6 |
| mfnc.Bx | mu0\_const\*mfnc.Ixx\*mfnc.Hx+mu0\_const\*mfnc.Ixy\*mfnc.Hy+mu0\_const\*mfnc.Ixz\*mfnc.Hz+mu0\_const\*mfnc.chimxx\*mfnc.Hx+mu0\_const\*mfnc.chimxy\*mfnc.Hy+mu0\_const\*mfnc.chimxz\*mfnc.Hz+mfnc.Brx | T | Magnetic flux density, x component | Domain 6 |
| mfnc.By | mu0\_const\*mfnc.Iyx\*mfnc.Hx+mu0\_const\*mfnc.Iyy\*mfnc.Hy+mu0\_const\*mfnc.Iyz\*mfnc.Hz+mu0\_const\*mfnc.chimyx\*mfnc.Hx+mu0\_const\*mfnc.chimyy\*mfnc.Hy+mu0\_const\*mfnc.chimyz\*mfnc.Hz+mfnc.Bry | T | Magnetic flux density, y component | Domain 6 |
| mfnc.Bz | mu0\_const\*mfnc.Izx\*mfnc.Hx+mu0\_const\*mfnc.Izy\*mfnc.Hy+mu0\_const\*mfnc.Izz\*mfnc.Hz+mu0\_const\*mfnc.chimzx\*mfnc.Hx+mu0\_const\*mfnc.chimzy\*mfnc.Hy+mu0\_const\*mfnc.chimzz\*mfnc.Hz+mfnc.Brz | T | Magnetic flux density, z component | Domain 6 |
| mfnc.normB | sqrt(realdot(mfnc.Bx,mfnc.Bx)+realdot(mfnc.By,mfnc.By)+realdot(mfnc.Bz,mfnc.Bz)) | T | Magnetic flux density norm | Domain 6 |
| mfnc.Mx | mfnc.Bx/mu0\_const-mfnc.Ixx\*mfnc.Hx-mfnc.Ixy\*mfnc.Hy-mfnc.Ixz\*mfnc.Hz | A/m | Magnetization, x component | Domain 6 |
| mfnc.My | mfnc.By/mu0\_const-mfnc.Iyx\*mfnc.Hx-mfnc.Iyy\*mfnc.Hy-mfnc.Iyz\*mfnc.Hz | A/m | Magnetization, y component | Domain 6 |
| mfnc.Mz | mfnc.Bz/mu0\_const-mfnc.Izx\*mfnc.Hx-mfnc.Izy\*mfnc.Hy-mfnc.Izz\*mfnc.Hz | A/m | Magnetization, z component | Domain 6 |
| mfnc.normM | sqrt(realdot(mfnc.Mx,mfnc.Mx)+realdot(mfnc.My,mfnc.My)+realdot(mfnc.Mz,mfnc.Mz)) | A/m | Magnetization norm | Domain 6 |
| mfnc.Ixx | 1 | 1 | Spatial identity matrix, xx component | Domain 6 |
| mfnc.Iyx | 0 | 1 | Spatial identity matrix, yx component | Domain 6 |
| mfnc.Izx | 0 | 1 | Spatial identity matrix, zx component | Domain 6 |
| mfnc.Ixy | 0 | 1 | Spatial identity matrix, xy component | Domain 6 |
| mfnc.Iyy | 1 | 1 | Spatial identity matrix, yy component | Domain 6 |
| mfnc.Izy | 0 | 1 | Spatial identity matrix, zy component | Domain 6 |
| mfnc.Ixz | 0 | 1 | Spatial identity matrix, xz component | Domain 6 |
| mfnc.Iyz | 0 | 1 | Spatial identity matrix, yz component | Domain 6 |
| mfnc.Izz | 1 | 1 | Spatial identity matrix, zz component | Domain 6 |
| mfnc.chimxx | -1+mfnc.murxx | 1 | Magnetic susceptibility, xx component | Domain 6 |
| mfnc.chimyx | mfnc.muryx | 1 | Magnetic susceptibility, yx component | Domain 6 |
| mfnc.chimzx | mfnc.murzx | 1 | Magnetic susceptibility, zx component | Domain 6 |
| mfnc.chimxy | mfnc.murxy | 1 | Magnetic susceptibility, xy component | Domain 6 |
| mfnc.chimyy | -1+mfnc.muryy | 1 | Magnetic susceptibility, yy component | Domain 6 |
| mfnc.chimzy | mfnc.murzy | 1 | Magnetic susceptibility, zy component | Domain 6 |
| mfnc.chimxz | mfnc.murxz | 1 | Magnetic susceptibility, xz component | Domain 6 |
| mfnc.chimyz | mfnc.muryz | 1 | Magnetic susceptibility, yz component | Domain 6 |
| mfnc.chimzz | -1+mfnc.murzz | 1 | Magnetic susceptibility, zz component | Domain 6 |
| mfnc.Brx | BB\*cos(pi\*(420+corr\_b)/180) | T | Remanent flux density, x component | Domain 6 |
| mfnc.Bry | BB\*sin(pi\*(420+corr\_b)/180) | T | Remanent flux density, y component | Domain 6 |
| mfnc.Brz | 0 | T | Remanent flux density, z component | Domain 6 |
| mfnc.normBr | sqrt(realdot(mfnc.Brx,mfnc.Brx)+realdot(mfnc.Bry,mfnc.Bry)+realdot(mfnc.Brz,mfnc.Brz)) | T | Remanent flux density norm | Domain 6 |
| mfnc.unTx | 0 | Pa | Maxwell upward surface stress tensor, x component | Boundaries 46–49, 54, 56, 58, 70, 72, 74 |
| mfnc.unTy | 0 | Pa | Maxwell upward surface stress tensor, y component | Boundaries 46–49, 54, 56, 58, 70, 72, 74 |
| mfnc.unTz | 0 | Pa | Maxwell upward surface stress tensor, z component | Boundaries 46–49, 54, 56, 58, 70, 72, 74 |
| mfnc.dnTx | mfnc.dnTmx | Pa | Maxwell downward surface stress tensor, x component | Boundaries 46–49, 54, 56, 58, 70, 72, 74 |
| mfnc.dnTy | mfnc.dnTmy | Pa | Maxwell downward surface stress tensor, y component | Boundaries 46–49, 54, 56, 58, 70, 72, 74 |
| mfnc.dnTz | mfnc.dnTmz | Pa | Maxwell downward surface stress tensor, z component | Boundaries 46–49, 54, 56, 58, 70, 72, 74 |
| mfnc.unx | unx |  | Normal vector up direction, x component | Boundaries 46–49, 54, 56, 58, 70, 72, 74 |
| mfnc.uny | uny |  | Normal vector up direction, y component | Boundaries 46–49, 54, 56, 58, 70, 72, 74 |
| mfnc.unz | unz |  | Normal vector up direction, z component | Boundaries 46–49, 54, 56, 58, 70, 72, 74 |
| mfnc.dnx | dnx |  | Normal vector down direction, x component | Boundaries 46–49, 54, 56, 58, 70, 72, 74 |
| mfnc.dny | dny |  | Normal vector down direction, y component | Boundaries 46–49, 54, 56, 58, 70, 72, 74 |
| mfnc.dnz | dnz |  | Normal vector down direction, z component | Boundaries 46–49, 54, 56, 58, 70, 72, 74 |
| mfnc.W | mfnc.Wm | J/m^3 | Energy density | Domain 6 |
| mfnc.dWm | mfnc.Wm | J/m^3 | Integrand for total magnetic energy | Domain 6 |
| mfnc.Wm | 0.5\*mu0\_const\*((mfnc.murxx\*mfnc.Hx+mfnc.murxy\*mfnc.Hy+mfnc.murxz\*mfnc.Hz)\*mfnc.Hx+(mfnc.muryx\*mfnc.Hx+mfnc.muryy\*mfnc.Hy+mfnc.muryz\*mfnc.Hz)\*mfnc.Hy+(mfnc.murzx\*mfnc.Hx+mfnc.murzy\*mfnc.Hy+mfnc.murzz\*mfnc.Hz)\*mfnc.Hz) | J/m^3 | Magnetic energy density | Domain 6 |

##### Shape Functions

| Name | Shape function | Unit | Description | Shape frame | Selection |
| --- | --- | --- | --- | --- | --- |
| Vm | Lagrange (Quadratic) | A | Magnetic scalar potential | Material | Domain 6 |

##### Weak Expressions

| Weak expression | Integration frame | Selection |
| --- | --- | --- |
| mfnc.d\*(-mfnc.Bx\*test(Vmx)-mfnc.By\*test(Vmy)-mfnc.Bz\*test(Vmz)) | Material | Domain 6 |

#### 2.4.12. ArrayBMagnet9

ArrayBMagnet9

Selection

| Geometric entity level | Domain |
| Selection | Domain 11 |

Equations

Settings

| Description | Value |
| Constitutive relation | Remanent flux density |
| Remanent flux density, x component | BB\*cos(2\*pi/360\*(8\*60 + corr\_b)) |
| Remanent flux density, y component | BB\*sin(2\*pi/360\*(8\*60 + corr\_b)) |
| Remanent flux density, z component | 0 |
| Relative permeability | From material |
| Relative permeability | {{1, 0, 0}, {0, 1, 0}, {0, 0, 1}} |

Properties from material

| Property | Material | Property group |
| Relative permeability | Soft Iron (without losses) | Basic |

##### Variables

| Name | Expression | Unit | Description | Selection |
| --- | --- | --- | --- | --- |
| mfnc.dnTmx | -0.5\*mfnc.unx\*(real(down(mfnc.Bx))\*real(down(mfnc.Hx))+real(down(mfnc.By))\*real(down(mfnc.Hy))+real(down(mfnc.Bz))\*real(down(mfnc.Hz)))+real(down(mfnc.Bx))\*(real(down(mfnc.Hx))\*mfnc.unx+real(down(mfnc.Hy))\*mfnc.uny+real(down(mfnc.Hz))\*mfnc.unz) | Pa | Maxwell downward magnetic surface stress tensor, x component | Boundaries 94–97, 102, 106, 108, 110, 112, 114 |
| mfnc.dnTmy | -0.5\*mfnc.uny\*(real(down(mfnc.Bx))\*real(down(mfnc.Hx))+real(down(mfnc.By))\*real(down(mfnc.Hy))+real(down(mfnc.Bz))\*real(down(mfnc.Hz)))+real(down(mfnc.By))\*(real(down(mfnc.Hx))\*mfnc.unx+real(down(mfnc.Hy))\*mfnc.uny+real(down(mfnc.Hz))\*mfnc.unz) | Pa | Maxwell downward magnetic surface stress tensor, y component | Boundaries 94–97, 102, 106, 108, 110, 112, 114 |
| mfnc.dnTmz | -0.5\*mfnc.unz\*(real(down(mfnc.Bx))\*real(down(mfnc.Hx))+real(down(mfnc.By))\*real(down(mfnc.Hy))+real(down(mfnc.Bz))\*real(down(mfnc.Hz)))+real(down(mfnc.Bz))\*(real(down(mfnc.Hx))\*mfnc.unx+real(down(mfnc.Hy))\*mfnc.uny+real(down(mfnc.Hz))\*mfnc.unz) | Pa | Maxwell downward magnetic surface stress tensor, z component | Boundaries 94–97, 102, 106, 108, 110, 112, 114 |
| mfnc.Hx | -Vmx | A/m | Magnetic field, x component | Domain 11 |
| mfnc.Hy | -Vmy | A/m | Magnetic field, y component | Domain 11 |
| mfnc.Hz | -Vmz | A/m | Magnetic field, z component | Domain 11 |
| mfnc.tHx | -VmTx | A/m | Tangential magnetic field, x component | Boundaries 94–97, 102, 106, 108, 110, 112, 114 |
| mfnc.tHy | -VmTy | A/m | Tangential magnetic field, y component | Boundaries 94–97, 102, 106, 108, 110, 112, 114 |
| mfnc.tHz | -VmTz | A/m | Tangential magnetic field, z component | Boundaries 94–97, 102, 106, 108, 110, 112, 114 |
| mfnc.normH | sqrt(realdot(mfnc.Hx,mfnc.Hx)+realdot(mfnc.Hy,mfnc.Hy)+realdot(mfnc.Hz,mfnc.Hz)) | A/m | Magnetic field norm | Domain 11 |
| mfnc.murxx | model.input.mur11 | 1 | Relative permeability, xx component | Domain 11 |
| mfnc.muryx | model.input.mur21 | 1 | Relative permeability, yx component | Domain 11 |
| mfnc.murzx | model.input.mur31 | 1 | Relative permeability, zx component | Domain 11 |
| mfnc.murxy | model.input.mur12 | 1 | Relative permeability, xy component | Domain 11 |
| mfnc.muryy | model.input.mur22 | 1 | Relative permeability, yy component | Domain 11 |
| mfnc.murzy | model.input.mur32 | 1 | Relative permeability, zy component | Domain 11 |
| mfnc.murxz | model.input.mur13 | 1 | Relative permeability, xz component | Domain 11 |
| mfnc.muryz | model.input.mur23 | 1 | Relative permeability, yz component | Domain 11 |
| mfnc.murzz | model.input.mur33 | 1 | Relative permeability, zz component | Domain 11 |
| mfnc.Bx | mu0\_const\*mfnc.Ixx\*mfnc.Hx+mu0\_const\*mfnc.Ixy\*mfnc.Hy+mu0\_const\*mfnc.Ixz\*mfnc.Hz+mu0\_const\*mfnc.chimxx\*mfnc.Hx+mu0\_const\*mfnc.chimxy\*mfnc.Hy+mu0\_const\*mfnc.chimxz\*mfnc.Hz+mfnc.Brx | T | Magnetic flux density, x component | Domain 11 |
| mfnc.By | mu0\_const\*mfnc.Iyx\*mfnc.Hx+mu0\_const\*mfnc.Iyy\*mfnc.Hy+mu0\_const\*mfnc.Iyz\*mfnc.Hz+mu0\_const\*mfnc.chimyx\*mfnc.Hx+mu0\_const\*mfnc.chimyy\*mfnc.Hy+mu0\_const\*mfnc.chimyz\*mfnc.Hz+mfnc.Bry | T | Magnetic flux density, y component | Domain 11 |
| mfnc.Bz | mu0\_const\*mfnc.Izx\*mfnc.Hx+mu0\_const\*mfnc.Izy\*mfnc.Hy+mu0\_const\*mfnc.Izz\*mfnc.Hz+mu0\_const\*mfnc.chimzx\*mfnc.Hx+mu0\_const\*mfnc.chimzy\*mfnc.Hy+mu0\_const\*mfnc.chimzz\*mfnc.Hz+mfnc.Brz | T | Magnetic flux density, z component | Domain 11 |
| mfnc.normB | sqrt(realdot(mfnc.Bx,mfnc.Bx)+realdot(mfnc.By,mfnc.By)+realdot(mfnc.Bz,mfnc.Bz)) | T | Magnetic flux density norm | Domain 11 |
| mfnc.Mx | mfnc.Bx/mu0\_const-mfnc.Ixx\*mfnc.Hx-mfnc.Ixy\*mfnc.Hy-mfnc.Ixz\*mfnc.Hz | A/m | Magnetization, x component | Domain 11 |
| mfnc.My | mfnc.By/mu0\_const-mfnc.Iyx\*mfnc.Hx-mfnc.Iyy\*mfnc.Hy-mfnc.Iyz\*mfnc.Hz | A/m | Magnetization, y component | Domain 11 |
| mfnc.Mz | mfnc.Bz/mu0\_const-mfnc.Izx\*mfnc.Hx-mfnc.Izy\*mfnc.Hy-mfnc.Izz\*mfnc.Hz | A/m | Magnetization, z component | Domain 11 |
| mfnc.normM | sqrt(realdot(mfnc.Mx,mfnc.Mx)+realdot(mfnc.My,mfnc.My)+realdot(mfnc.Mz,mfnc.Mz)) | A/m | Magnetization norm | Domain 11 |
| mfnc.Ixx | 1 | 1 | Spatial identity matrix, xx component | Domain 11 |
| mfnc.Iyx | 0 | 1 | Spatial identity matrix, yx component | Domain 11 |
| mfnc.Izx | 0 | 1 | Spatial identity matrix, zx component | Domain 11 |
| mfnc.Ixy | 0 | 1 | Spatial identity matrix, xy component | Domain 11 |
| mfnc.Iyy | 1 | 1 | Spatial identity matrix, yy component | Domain 11 |
| mfnc.Izy | 0 | 1 | Spatial identity matrix, zy component | Domain 11 |
| mfnc.Ixz | 0 | 1 | Spatial identity matrix, xz component | Domain 11 |
| mfnc.Iyz | 0 | 1 | Spatial identity matrix, yz component | Domain 11 |
| mfnc.Izz | 1 | 1 | Spatial identity matrix, zz component | Domain 11 |
| mfnc.chimxx | -1+mfnc.murxx | 1 | Magnetic susceptibility, xx component | Domain 11 |
| mfnc.chimyx | mfnc.muryx | 1 | Magnetic susceptibility, yx component | Domain 11 |
| mfnc.chimzx | mfnc.murzx | 1 | Magnetic susceptibility, zx component | Domain 11 |
| mfnc.chimxy | mfnc.murxy | 1 | Magnetic susceptibility, xy component | Domain 11 |
| mfnc.chimyy | -1+mfnc.muryy | 1 | Magnetic susceptibility, yy component | Domain 11 |
| mfnc.chimzy | mfnc.murzy | 1 | Magnetic susceptibility, zy component | Domain 11 |
| mfnc.chimxz | mfnc.murxz | 1 | Magnetic susceptibility, xz component | Domain 11 |
| mfnc.chimyz | mfnc.muryz | 1 | Magnetic susceptibility, yz component | Domain 11 |
| mfnc.chimzz | -1+mfnc.murzz | 1 | Magnetic susceptibility, zz component | Domain 11 |
| mfnc.Brx | BB\*cos(pi\*(480+corr\_b)/180) | T | Remanent flux density, x component | Domain 11 |
| mfnc.Bry | BB\*sin(pi\*(480+corr\_b)/180) | T | Remanent flux density, y component | Domain 11 |
| mfnc.Brz | 0 | T | Remanent flux density, z component | Domain 11 |
| mfnc.normBr | sqrt(realdot(mfnc.Brx,mfnc.Brx)+realdot(mfnc.Bry,mfnc.Bry)+realdot(mfnc.Brz,mfnc.Brz)) | T | Remanent flux density norm | Domain 11 |
| mfnc.unTx | 0 | Pa | Maxwell upward surface stress tensor, x component | Boundaries 94–97, 102, 106, 108, 110, 112, 114 |
| mfnc.unTy | 0 | Pa | Maxwell upward surface stress tensor, y component | Boundaries 94–97, 102, 106, 108, 110, 112, 114 |
| mfnc.unTz | 0 | Pa | Maxwell upward surface stress tensor, z component | Boundaries 94–97, 102, 106, 108, 110, 112, 114 |
| mfnc.dnTx | mfnc.dnTmx | Pa | Maxwell downward surface stress tensor, x component | Boundaries 94–97, 102, 106, 108, 110, 112, 114 |
| mfnc.dnTy | mfnc.dnTmy | Pa | Maxwell downward surface stress tensor, y component | Boundaries 94–97, 102, 106, 108, 110, 112, 114 |
| mfnc.dnTz | mfnc.dnTmz | Pa | Maxwell downward surface stress tensor, z component | Boundaries 94–97, 102, 106, 108, 110, 112, 114 |
| mfnc.unx | unx |  | Normal vector up direction, x component | Boundaries 94–97, 102, 106, 108, 110, 112, 114 |
| mfnc.uny | uny |  | Normal vector up direction, y component | Boundaries 94–97, 102, 106, 108, 110, 112, 114 |
| mfnc.unz | unz |  | Normal vector up direction, z component | Boundaries 94–97, 102, 106, 108, 110, 112, 114 |
| mfnc.dnx | dnx |  | Normal vector down direction, x component | Boundaries 94–97, 102, 106, 108, 110, 112, 114 |
| mfnc.dny | dny |  | Normal vector down direction, y component | Boundaries 94–97, 102, 106, 108, 110, 112, 114 |
| mfnc.dnz | dnz |  | Normal vector down direction, z component | Boundaries 94–97, 102, 106, 108, 110, 112, 114 |
| mfnc.W | mfnc.Wm | J/m^3 | Energy density | Domain 11 |
| mfnc.dWm | mfnc.Wm | J/m^3 | Integrand for total magnetic energy | Domain 11 |
| mfnc.Wm | 0.5\*mu0\_const\*((mfnc.murxx\*mfnc.Hx+mfnc.murxy\*mfnc.Hy+mfnc.murxz\*mfnc.Hz)\*mfnc.Hx+(mfnc.muryx\*mfnc.Hx+mfnc.muryy\*mfnc.Hy+mfnc.muryz\*mfnc.Hz)\*mfnc.Hy+(mfnc.murzx\*mfnc.Hx+mfnc.murzy\*mfnc.Hy+mfnc.murzz\*mfnc.Hz)\*mfnc.Hz) | J/m^3 | Magnetic energy density | Domain 11 |

##### Shape Functions

| Name | Shape function | Unit | Description | Shape frame | Selection |
| --- | --- | --- | --- | --- | --- |
| Vm | Lagrange (Quadratic) | A | Magnetic scalar potential | Material | Domain 11 |

##### Weak Expressions

| Weak expression | Integration frame | Selection |
| --- | --- | --- |
| mfnc.d\*(-mfnc.Bx\*test(Vmx)-mfnc.By\*test(Vmy)-mfnc.Bz\*test(Vmz)) | Material | Domain 11 |

#### 2.4.13. ArrayBMagnet10

ArrayBMagnet10

Selection

| Geometric entity level | Domain |
| Selection | Domain 18 |

Equations

Settings

| Description | Value |
| Constitutive relation | Remanent flux density |
| Remanent flux density, x component | BB\*cos(2\*pi/360\*(9\*60 + corr\_b)) |
| Remanent flux density, y component | BB\*sin(2\*pi/360\*(9\*60 + corr\_b)) |
| Remanent flux density, z component | 0 |
| Relative permeability | From material |
| Relative permeability | {{1, 0, 0}, {0, 1, 0}, {0, 0, 1}} |

Properties from material

| Property | Material | Property group |
| Relative permeability | Soft Iron (without losses) | Basic |

##### Variables

| Name | Expression | Unit | Description | Selection |
| --- | --- | --- | --- | --- |
| mfnc.dnTmx | -0.5\*mfnc.unx\*(real(down(mfnc.Bx))\*real(down(mfnc.Hx))+real(down(mfnc.By))\*real(down(mfnc.Hy))+real(down(mfnc.Bz))\*real(down(mfnc.Hz)))+real(down(mfnc.Bx))\*(real(down(mfnc.Hx))\*mfnc.unx+real(down(mfnc.Hy))\*mfnc.uny+real(down(mfnc.Hz))\*mfnc.unz) | Pa | Maxwell downward magnetic surface stress tensor, x component | Boundaries 154–158, 164–165, 170–171, 174 |
| mfnc.dnTmy | -0.5\*mfnc.uny\*(real(down(mfnc.Bx))\*real(down(mfnc.Hx))+real(down(mfnc.By))\*real(down(mfnc.Hy))+real(down(mfnc.Bz))\*real(down(mfnc.Hz)))+real(down(mfnc.By))\*(real(down(mfnc.Hx))\*mfnc.unx+real(down(mfnc.Hy))\*mfnc.uny+real(down(mfnc.Hz))\*mfnc.unz) | Pa | Maxwell downward magnetic surface stress tensor, y component | Boundaries 154–158, 164–165, 170–171, 174 |
| mfnc.dnTmz | -0.5\*mfnc.unz\*(real(down(mfnc.Bx))\*real(down(mfnc.Hx))+real(down(mfnc.By))\*real(down(mfnc.Hy))+real(down(mfnc.Bz))\*real(down(mfnc.Hz)))+real(down(mfnc.Bz))\*(real(down(mfnc.Hx))\*mfnc.unx+real(down(mfnc.Hy))\*mfnc.uny+real(down(mfnc.Hz))\*mfnc.unz) | Pa | Maxwell downward magnetic surface stress tensor, z component | Boundaries 154–158, 164–165, 170–171, 174 |
| mfnc.Hx | -Vmx | A/m | Magnetic field, x component | Domain 18 |
| mfnc.Hy | -Vmy | A/m | Magnetic field, y component | Domain 18 |
| mfnc.Hz | -Vmz | A/m | Magnetic field, z component | Domain 18 |
| mfnc.tHx | -VmTx | A/m | Tangential magnetic field, x component | Boundaries 154–158, 164–165, 170–171, 174 |
| mfnc.tHy | -VmTy | A/m | Tangential magnetic field, y component | Boundaries 154–158, 164–165, 170–171, 174 |
| mfnc.tHz | -VmTz | A/m | Tangential magnetic field, z component | Boundaries 154–158, 164–165, 170–171, 174 |
| mfnc.normH | sqrt(realdot(mfnc.Hx,mfnc.Hx)+realdot(mfnc.Hy,mfnc.Hy)+realdot(mfnc.Hz,mfnc.Hz)) | A/m | Magnetic field norm | Domain 18 |
| mfnc.murxx | model.input.mur11 | 1 | Relative permeability, xx component | Domain 18 |
| mfnc.muryx | model.input.mur21 | 1 | Relative permeability, yx component | Domain 18 |
| mfnc.murzx | model.input.mur31 | 1 | Relative permeability, zx component | Domain 18 |
| mfnc.murxy | model.input.mur12 | 1 | Relative permeability, xy component | Domain 18 |
| mfnc.muryy | model.input.mur22 | 1 | Relative permeability, yy component | Domain 18 |
| mfnc.murzy | model.input.mur32 | 1 | Relative permeability, zy component | Domain 18 |
| mfnc.murxz | model.input.mur13 | 1 | Relative permeability, xz component | Domain 18 |
| mfnc.muryz | model.input.mur23 | 1 | Relative permeability, yz component | Domain 18 |
| mfnc.murzz | model.input.mur33 | 1 | Relative permeability, zz component | Domain 18 |
| mfnc.Bx | mu0\_const\*mfnc.Ixx\*mfnc.Hx+mu0\_const\*mfnc.Ixy\*mfnc.Hy+mu0\_const\*mfnc.Ixz\*mfnc.Hz+mu0\_const\*mfnc.chimxx\*mfnc.Hx+mu0\_const\*mfnc.chimxy\*mfnc.Hy+mu0\_const\*mfnc.chimxz\*mfnc.Hz+mfnc.Brx | T | Magnetic flux density, x component | Domain 18 |
| mfnc.By | mu0\_const\*mfnc.Iyx\*mfnc.Hx+mu0\_const\*mfnc.Iyy\*mfnc.Hy+mu0\_const\*mfnc.Iyz\*mfnc.Hz+mu0\_const\*mfnc.chimyx\*mfnc.Hx+mu0\_const\*mfnc.chimyy\*mfnc.Hy+mu0\_const\*mfnc.chimyz\*mfnc.Hz+mfnc.Bry | T | Magnetic flux density, y component | Domain 18 |
| mfnc.Bz | mu0\_const\*mfnc.Izx\*mfnc.Hx+mu0\_const\*mfnc.Izy\*mfnc.Hy+mu0\_const\*mfnc.Izz\*mfnc.Hz+mu0\_const\*mfnc.chimzx\*mfnc.Hx+mu0\_const\*mfnc.chimzy\*mfnc.Hy+mu0\_const\*mfnc.chimzz\*mfnc.Hz+mfnc.Brz | T | Magnetic flux density, z component | Domain 18 |
| mfnc.normB | sqrt(realdot(mfnc.Bx,mfnc.Bx)+realdot(mfnc.By,mfnc.By)+realdot(mfnc.Bz,mfnc.Bz)) | T | Magnetic flux density norm | Domain 18 |
| mfnc.Mx | mfnc.Bx/mu0\_const-mfnc.Ixx\*mfnc.Hx-mfnc.Ixy\*mfnc.Hy-mfnc.Ixz\*mfnc.Hz | A/m | Magnetization, x component | Domain 18 |
| mfnc.My | mfnc.By/mu0\_const-mfnc.Iyx\*mfnc.Hx-mfnc.Iyy\*mfnc.Hy-mfnc.Iyz\*mfnc.Hz | A/m | Magnetization, y component | Domain 18 |
| mfnc.Mz | mfnc.Bz/mu0\_const-mfnc.Izx\*mfnc.Hx-mfnc.Izy\*mfnc.Hy-mfnc.Izz\*mfnc.Hz | A/m | Magnetization, z component | Domain 18 |
| mfnc.normM | sqrt(realdot(mfnc.Mx,mfnc.Mx)+realdot(mfnc.My,mfnc.My)+realdot(mfnc.Mz,mfnc.Mz)) | A/m | Magnetization norm | Domain 18 |
| mfnc.Ixx | 1 | 1 | Spatial identity matrix, xx component | Domain 18 |
| mfnc.Iyx | 0 | 1 | Spatial identity matrix, yx component | Domain 18 |
| mfnc.Izx | 0 | 1 | Spatial identity matrix, zx component | Domain 18 |
| mfnc.Ixy | 0 | 1 | Spatial identity matrix, xy component | Domain 18 |
| mfnc.Iyy | 1 | 1 | Spatial identity matrix, yy component | Domain 18 |
| mfnc.Izy | 0 | 1 | Spatial identity matrix, zy component | Domain 18 |
| mfnc.Ixz | 0 | 1 | Spatial identity matrix, xz component | Domain 18 |
| mfnc.Iyz | 0 | 1 | Spatial identity matrix, yz component | Domain 18 |
| mfnc.Izz | 1 | 1 | Spatial identity matrix, zz component | Domain 18 |
| mfnc.chimxx | -1+mfnc.murxx | 1 | Magnetic susceptibility, xx component | Domain 18 |
| mfnc.chimyx | mfnc.muryx | 1 | Magnetic susceptibility, yx component | Domain 18 |
| mfnc.chimzx | mfnc.murzx | 1 | Magnetic susceptibility, zx component | Domain 18 |
| mfnc.chimxy | mfnc.murxy | 1 | Magnetic susceptibility, xy component | Domain 18 |
| mfnc.chimyy | -1+mfnc.muryy | 1 | Magnetic susceptibility, yy component | Domain 18 |
| mfnc.chimzy | mfnc.murzy | 1 | Magnetic susceptibility, zy component | Domain 18 |
| mfnc.chimxz | mfnc.murxz | 1 | Magnetic susceptibility, xz component | Domain 18 |
| mfnc.chimyz | mfnc.muryz | 1 | Magnetic susceptibility, yz component | Domain 18 |
| mfnc.chimzz | -1+mfnc.murzz | 1 | Magnetic susceptibility, zz component | Domain 18 |
| mfnc.Brx | BB\*cos(pi\*(540+corr\_b)/180) | T | Remanent flux density, x component | Domain 18 |
| mfnc.Bry | BB\*sin(pi\*(540+corr\_b)/180) | T | Remanent flux density, y component | Domain 18 |
| mfnc.Brz | 0 | T | Remanent flux density, z component | Domain 18 |
| mfnc.normBr | sqrt(realdot(mfnc.Brx,mfnc.Brx)+realdot(mfnc.Bry,mfnc.Bry)+realdot(mfnc.Brz,mfnc.Brz)) | T | Remanent flux density norm | Domain 18 |
| mfnc.unTx | 0 | Pa | Maxwell upward surface stress tensor, x component | Boundaries 154–158, 164–165, 170–171, 174 |
| mfnc.unTy | 0 | Pa | Maxwell upward surface stress tensor, y component | Boundaries 154–158, 164–165, 170–171, 174 |
| mfnc.unTz | 0 | Pa | Maxwell upward surface stress tensor, z component | Boundaries 154–158, 164–165, 170–171, 174 |
| mfnc.dnTx | mfnc.dnTmx | Pa | Maxwell downward surface stress tensor, x component | Boundaries 154–158, 164–165, 170–171, 174 |
| mfnc.dnTy | mfnc.dnTmy | Pa | Maxwell downward surface stress tensor, y component | Boundaries 154–158, 164–165, 170–171, 174 |
| mfnc.dnTz | mfnc.dnTmz | Pa | Maxwell downward surface stress tensor, z component | Boundaries 154–158, 164–165, 170–171, 174 |
| mfnc.unx | unx |  | Normal vector up direction, x component | Boundaries 154–158, 164–165, 170–171, 174 |
| mfnc.uny | uny |  | Normal vector up direction, y component | Boundaries 154–158, 164–165, 170–171, 174 |
| mfnc.unz | unz |  | Normal vector up direction, z component | Boundaries 154–158, 164–165, 170–171, 174 |
| mfnc.dnx | dnx |  | Normal vector down direction, x component | Boundaries 154–158, 164–165, 170–171, 174 |
| mfnc.dny | dny |  | Normal vector down direction, y component | Boundaries 154–158, 164–165, 170–171, 174 |
| mfnc.dnz | dnz |  | Normal vector down direction, z component | Boundaries 154–158, 164–165, 170–171, 174 |
| mfnc.W | mfnc.Wm | J/m^3 | Energy density | Domain 18 |
| mfnc.dWm | mfnc.Wm | J/m^3 | Integrand for total magnetic energy | Domain 18 |
| mfnc.Wm | 0.5\*mu0\_const\*((mfnc.murxx\*mfnc.Hx+mfnc.murxy\*mfnc.Hy+mfnc.murxz\*mfnc.Hz)\*mfnc.Hx+(mfnc.muryx\*mfnc.Hx+mfnc.muryy\*mfnc.Hy+mfnc.muryz\*mfnc.Hz)\*mfnc.Hy+(mfnc.murzx\*mfnc.Hx+mfnc.murzy\*mfnc.Hy+mfnc.murzz\*mfnc.Hz)\*mfnc.Hz) | J/m^3 | Magnetic energy density | Domain 18 |

##### Shape Functions

| Name | Shape function | Unit | Description | Shape frame | Selection |
| --- | --- | --- | --- | --- | --- |
| Vm | Lagrange (Quadratic) | A | Magnetic scalar potential | Material | Domain 18 |

##### Weak Expressions

| Weak expression | Integration frame | Selection |
| --- | --- | --- |
| mfnc.d\*(-mfnc.Bx\*test(Vmx)-mfnc.By\*test(Vmy)-mfnc.Bz\*test(Vmz)) | Material | Domain 18 |

#### 2.4.14. ArrayBMagnet11

ArrayBMagnet11

Selection

| Geometric entity level | Domain |
| Selection | Domain 22 |

Equations

Settings

| Description | Value |
| Constitutive relation | Remanent flux density |
| Remanent flux density, x component | BB\*cos(2\*pi/360\*(10\*60 + corr\_b)) |
| Remanent flux density, y component | BB\*sin(2\*pi/360\*(10\*60 + corr\_b)) |
| Remanent flux density, z component | 0 |
| Relative permeability | From material |
| Relative permeability | {{1, 0, 0}, {0, 1, 0}, {0, 0, 1}} |

Properties from material

| Property | Material | Property group |
| Relative permeability | Soft Iron (without losses) | Basic |

##### Variables

| Name | Expression | Unit | Description | Selection |
| --- | --- | --- | --- | --- |
| mfnc.dnTmx | -0.5\*mfnc.unx\*(real(down(mfnc.Bx))\*real(down(mfnc.Hx))+real(down(mfnc.By))\*real(down(mfnc.Hy))+real(down(mfnc.Bz))\*real(down(mfnc.Hz)))+real(down(mfnc.Bx))\*(real(down(mfnc.Hx))\*mfnc.unx+real(down(mfnc.Hy))\*mfnc.uny+real(down(mfnc.Hz))\*mfnc.unz) | Pa | Maxwell downward magnetic surface stress tensor, x component | Boundaries 202–205, 210, 212, 214, 216, 218, 230 |
| mfnc.dnTmy | -0.5\*mfnc.uny\*(real(down(mfnc.Bx))\*real(down(mfnc.Hx))+real(down(mfnc.By))\*real(down(mfnc.Hy))+real(down(mfnc.Bz))\*real(down(mfnc.Hz)))+real(down(mfnc.By))\*(real(down(mfnc.Hx))\*mfnc.unx+real(down(mfnc.Hy))\*mfnc.uny+real(down(mfnc.Hz))\*mfnc.unz) | Pa | Maxwell downward magnetic surface stress tensor, y component | Boundaries 202–205, 210, 212, 214, 216, 218, 230 |
| mfnc.dnTmz | -0.5\*mfnc.unz\*(real(down(mfnc.Bx))\*real(down(mfnc.Hx))+real(down(mfnc.By))\*real(down(mfnc.Hy))+real(down(mfnc.Bz))\*real(down(mfnc.Hz)))+real(down(mfnc.Bz))\*(real(down(mfnc.Hx))\*mfnc.unx+real(down(mfnc.Hy))\*mfnc.uny+real(down(mfnc.Hz))\*mfnc.unz) | Pa | Maxwell downward magnetic surface stress tensor, z component | Boundaries 202–205, 210, 212, 214, 216, 218, 230 |
| mfnc.Hx | -Vmx | A/m | Magnetic field, x component | Domain 22 |
| mfnc.Hy | -Vmy | A/m | Magnetic field, y component | Domain 22 |
| mfnc.Hz | -Vmz | A/m | Magnetic field, z component | Domain 22 |
| mfnc.tHx | -VmTx | A/m | Tangential magnetic field, x component | Boundaries 202–205, 210, 212, 214, 216, 218, 230 |
| mfnc.tHy | -VmTy | A/m | Tangential magnetic field, y component | Boundaries 202–205, 210, 212, 214, 216, 218, 230 |
| mfnc.tHz | -VmTz | A/m | Tangential magnetic field, z component | Boundaries 202–205, 210, 212, 214, 216, 218, 230 |
| mfnc.normH | sqrt(realdot(mfnc.Hx,mfnc.Hx)+realdot(mfnc.Hy,mfnc.Hy)+realdot(mfnc.Hz,mfnc.Hz)) | A/m | Magnetic field norm | Domain 22 |
| mfnc.murxx | model.input.mur11 | 1 | Relative permeability, xx component | Domain 22 |
| mfnc.muryx | model.input.mur21 | 1 | Relative permeability, yx component | Domain 22 |
| mfnc.murzx | model.input.mur31 | 1 | Relative permeability, zx component | Domain 22 |
| mfnc.murxy | model.input.mur12 | 1 | Relative permeability, xy component | Domain 22 |
| mfnc.muryy | model.input.mur22 | 1 | Relative permeability, yy component | Domain 22 |
| mfnc.murzy | model.input.mur32 | 1 | Relative permeability, zy component | Domain 22 |
| mfnc.murxz | model.input.mur13 | 1 | Relative permeability, xz component | Domain 22 |
| mfnc.muryz | model.input.mur23 | 1 | Relative permeability, yz component | Domain 22 |
| mfnc.murzz | model.input.mur33 | 1 | Relative permeability, zz component | Domain 22 |
| mfnc.Bx | mu0\_const\*mfnc.Ixx\*mfnc.Hx+mu0\_const\*mfnc.Ixy\*mfnc.Hy+mu0\_const\*mfnc.Ixz\*mfnc.Hz+mu0\_const\*mfnc.chimxx\*mfnc.Hx+mu0\_const\*mfnc.chimxy\*mfnc.Hy+mu0\_const\*mfnc.chimxz\*mfnc.Hz+mfnc.Brx | T | Magnetic flux density, x component | Domain 22 |
| mfnc.By | mu0\_const\*mfnc.Iyx\*mfnc.Hx+mu0\_const\*mfnc.Iyy\*mfnc.Hy+mu0\_const\*mfnc.Iyz\*mfnc.Hz+mu0\_const\*mfnc.chimyx\*mfnc.Hx+mu0\_const\*mfnc.chimyy\*mfnc.Hy+mu0\_const\*mfnc.chimyz\*mfnc.Hz+mfnc.Bry | T | Magnetic flux density, y component | Domain 22 |
| mfnc.Bz | mu0\_const\*mfnc.Izx\*mfnc.Hx+mu0\_const\*mfnc.Izy\*mfnc.Hy+mu0\_const\*mfnc.Izz\*mfnc.Hz+mu0\_const\*mfnc.chimzx\*mfnc.Hx+mu0\_const\*mfnc.chimzy\*mfnc.Hy+mu0\_const\*mfnc.chimzz\*mfnc.Hz+mfnc.Brz | T | Magnetic flux density, z component | Domain 22 |
| mfnc.normB | sqrt(realdot(mfnc.Bx,mfnc.Bx)+realdot(mfnc.By,mfnc.By)+realdot(mfnc.Bz,mfnc.Bz)) | T | Magnetic flux density norm | Domain 22 |
| mfnc.Mx | mfnc.Bx/mu0\_const-mfnc.Ixx\*mfnc.Hx-mfnc.Ixy\*mfnc.Hy-mfnc.Ixz\*mfnc.Hz | A/m | Magnetization, x component | Domain 22 |
| mfnc.My | mfnc.By/mu0\_const-mfnc.Iyx\*mfnc.Hx-mfnc.Iyy\*mfnc.Hy-mfnc.Iyz\*mfnc.Hz | A/m | Magnetization, y component | Domain 22 |
| mfnc.Mz | mfnc.Bz/mu0\_const-mfnc.Izx\*mfnc.Hx-mfnc.Izy\*mfnc.Hy-mfnc.Izz\*mfnc.Hz | A/m | Magnetization, z component | Domain 22 |
| mfnc.normM | sqrt(realdot(mfnc.Mx,mfnc.Mx)+realdot(mfnc.My,mfnc.My)+realdot(mfnc.Mz,mfnc.Mz)) | A/m | Magnetization norm | Domain 22 |
| mfnc.Ixx | 1 | 1 | Spatial identity matrix, xx component | Domain 22 |
| mfnc.Iyx | 0 | 1 | Spatial identity matrix, yx component | Domain 22 |
| mfnc.Izx | 0 | 1 | Spatial identity matrix, zx component | Domain 22 |
| mfnc.Ixy | 0 | 1 | Spatial identity matrix, xy component | Domain 22 |
| mfnc.Iyy | 1 | 1 | Spatial identity matrix, yy component | Domain 22 |
| mfnc.Izy | 0 | 1 | Spatial identity matrix, zy component | Domain 22 |
| mfnc.Ixz | 0 | 1 | Spatial identity matrix, xz component | Domain 22 |
| mfnc.Iyz | 0 | 1 | Spatial identity matrix, yz component | Domain 22 |
| mfnc.Izz | 1 | 1 | Spatial identity matrix, zz component | Domain 22 |
| mfnc.chimxx | -1+mfnc.murxx | 1 | Magnetic susceptibility, xx component | Domain 22 |
| mfnc.chimyx | mfnc.muryx | 1 | Magnetic susceptibility, yx component | Domain 22 |
| mfnc.chimzx | mfnc.murzx | 1 | Magnetic susceptibility, zx component | Domain 22 |
| mfnc.chimxy | mfnc.murxy | 1 | Magnetic susceptibility, xy component | Domain 22 |
| mfnc.chimyy | -1+mfnc.muryy | 1 | Magnetic susceptibility, yy component | Domain 22 |
| mfnc.chimzy | mfnc.murzy | 1 | Magnetic susceptibility, zy component | Domain 22 |
| mfnc.chimxz | mfnc.murxz | 1 | Magnetic susceptibility, xz component | Domain 22 |
| mfnc.chimyz | mfnc.muryz | 1 | Magnetic susceptibility, yz component | Domain 22 |
| mfnc.chimzz | -1+mfnc.murzz | 1 | Magnetic susceptibility, zz component | Domain 22 |
| mfnc.Brx | BB\*cos(pi\*(600+corr\_b)/180) | T | Remanent flux density, x component | Domain 22 |
| mfnc.Bry | BB\*sin(pi\*(600+corr\_b)/180) | T | Remanent flux density, y component | Domain 22 |
| mfnc.Brz | 0 | T | Remanent flux density, z component | Domain 22 |
| mfnc.normBr | sqrt(realdot(mfnc.Brx,mfnc.Brx)+realdot(mfnc.Bry,mfnc.Bry)+realdot(mfnc.Brz,mfnc.Brz)) | T | Remanent flux density norm | Domain 22 |
| mfnc.unTx | 0 | Pa | Maxwell upward surface stress tensor, x component | Boundaries 202–205, 210, 212, 214, 216, 218, 230 |
| mfnc.unTy | 0 | Pa | Maxwell upward surface stress tensor, y component | Boundaries 202–205, 210, 212, 214, 216, 218, 230 |
| mfnc.unTz | 0 | Pa | Maxwell upward surface stress tensor, z component | Boundaries 202–205, 210, 212, 214, 216, 218, 230 |
| mfnc.dnTx | mfnc.dnTmx | Pa | Maxwell downward surface stress tensor, x component | Boundaries 202–205, 210, 212, 214, 216, 218, 230 |
| mfnc.dnTy | mfnc.dnTmy | Pa | Maxwell downward surface stress tensor, y component | Boundaries 202–205, 210, 212, 214, 216, 218, 230 |
| mfnc.dnTz | mfnc.dnTmz | Pa | Maxwell downward surface stress tensor, z component | Boundaries 202–205, 210, 212, 214, 216, 218, 230 |
| mfnc.unx | unx |  | Normal vector up direction, x component | Boundaries 202–205, 210, 212, 214, 216, 218, 230 |
| mfnc.uny | uny |  | Normal vector up direction, y component | Boundaries 202–205, 210, 212, 214, 216, 218, 230 |
| mfnc.unz | unz |  | Normal vector up direction, z component | Boundaries 202–205, 210, 212, 214, 216, 218, 230 |
| mfnc.dnx | dnx |  | Normal vector down direction, x component | Boundaries 202–205, 210, 212, 214, 216, 218, 230 |
| mfnc.dny | dny |  | Normal vector down direction, y component | Boundaries 202–205, 210, 212, 214, 216, 218, 230 |
| mfnc.dnz | dnz |  | Normal vector down direction, z component | Boundaries 202–205, 210, 212, 214, 216, 218, 230 |
| mfnc.W | mfnc.Wm | J/m^3 | Energy density | Domain 22 |
| mfnc.dWm | mfnc.Wm | J/m^3 | Integrand for total magnetic energy | Domain 22 |
| mfnc.Wm | 0.5\*mu0\_const\*((mfnc.murxx\*mfnc.Hx+mfnc.murxy\*mfnc.Hy+mfnc.murxz\*mfnc.Hz)\*mfnc.Hx+(mfnc.muryx\*mfnc.Hx+mfnc.muryy\*mfnc.Hy+mfnc.muryz\*mfnc.Hz)\*mfnc.Hy+(mfnc.murzx\*mfnc.Hx+mfnc.murzy\*mfnc.Hy+mfnc.murzz\*mfnc.Hz)\*mfnc.Hz) | J/m^3 | Magnetic energy density | Domain 22 |

##### Shape Functions

| Name | Shape function | Unit | Description | Shape frame | Selection |
| --- | --- | --- | --- | --- | --- |
| Vm | Lagrange (Quadratic) | A | Magnetic scalar potential | Material | Domain 22 |

##### Weak Expressions

| Weak expression | Integration frame | Selection |
| --- | --- | --- |
| mfnc.d\*(-mfnc.Bx\*test(Vmx)-mfnc.By\*test(Vmy)-mfnc.Bz\*test(Vmz)) | Material | Domain 22 |

#### 2.4.15. ArrayBMagnet12

ArrayBMagnet12

Selection

| Geometric entity level | Domain |
| Selection | Domain 27 |

Equations

Settings

| Description | Value |
| Constitutive relation | Remanent flux density |
| Remanent flux density, x component | BB\*cos(2\*pi/360\*(11\*60 + corr\_b)) |
| Remanent flux density, y component | BB\*sin(2\*pi/360\*(11\*60 + corr\_b)) |
| Remanent flux density, z component | 0 |
| Relative permeability | From material |
| Relative permeability | {{1, 0, 0}, {0, 1, 0}, {0, 0, 1}} |

Properties from material

| Property | Material | Property group |
| Relative permeability | Soft Iron (without losses) | Basic |

##### Variables

| Name | Expression | Unit | Description | Selection |
| --- | --- | --- | --- | --- |
| mfnc.dnTmx | -0.5\*mfnc.unx\*(real(down(mfnc.Bx))\*real(down(mfnc.Hx))+real(down(mfnc.By))\*real(down(mfnc.Hy))+real(down(mfnc.Bz))\*real(down(mfnc.Hz)))+real(down(mfnc.Bx))\*(real(down(mfnc.Hx))\*mfnc.unx+real(down(mfnc.Hy))\*mfnc.uny+real(down(mfnc.Hz))\*mfnc.unz) | Pa | Maxwell downward magnetic surface stress tensor, x component | Boundaries 250–253, 258, 260, 262, 266, 268, 270 |
| mfnc.dnTmy | -0.5\*mfnc.uny\*(real(down(mfnc.Bx))\*real(down(mfnc.Hx))+real(down(mfnc.By))\*real(down(mfnc.Hy))+real(down(mfnc.Bz))\*real(down(mfnc.Hz)))+real(down(mfnc.By))\*(real(down(mfnc.Hx))\*mfnc.unx+real(down(mfnc.Hy))\*mfnc.uny+real(down(mfnc.Hz))\*mfnc.unz) | Pa | Maxwell downward magnetic surface stress tensor, y component | Boundaries 250–253, 258, 260, 262, 266, 268, 270 |
| mfnc.dnTmz | -0.5\*mfnc.unz\*(real(down(mfnc.Bx))\*real(down(mfnc.Hx))+real(down(mfnc.By))\*real(down(mfnc.Hy))+real(down(mfnc.Bz))\*real(down(mfnc.Hz)))+real(down(mfnc.Bz))\*(real(down(mfnc.Hx))\*mfnc.unx+real(down(mfnc.Hy))\*mfnc.uny+real(down(mfnc.Hz))\*mfnc.unz) | Pa | Maxwell downward magnetic surface stress tensor, z component | Boundaries 250–253, 258, 260, 262, 266, 268, 270 |
| mfnc.Hx | -Vmx | A/m | Magnetic field, x component | Domain 27 |
| mfnc.Hy | -Vmy | A/m | Magnetic field, y component | Domain 27 |
| mfnc.Hz | -Vmz | A/m | Magnetic field, z component | Domain 27 |
| mfnc.tHx | -VmTx | A/m | Tangential magnetic field, x component | Boundaries 250–253, 258, 260, 262, 266, 268, 270 |
| mfnc.tHy | -VmTy | A/m | Tangential magnetic field, y component | Boundaries 250–253, 258, 260, 262, 266, 268, 270 |
| mfnc.tHz | -VmTz | A/m | Tangential magnetic field, z component | Boundaries 250–253, 258, 260, 262, 266, 268, 270 |
| mfnc.normH | sqrt(realdot(mfnc.Hx,mfnc.Hx)+realdot(mfnc.Hy,mfnc.Hy)+realdot(mfnc.Hz,mfnc.Hz)) | A/m | Magnetic field norm | Domain 27 |
| mfnc.murxx | model.input.mur11 | 1 | Relative permeability, xx component | Domain 27 |
| mfnc.muryx | model.input.mur21 | 1 | Relative permeability, yx component | Domain 27 |
| mfnc.murzx | model.input.mur31 | 1 | Relative permeability, zx component | Domain 27 |
| mfnc.murxy | model.input.mur12 | 1 | Relative permeability, xy component | Domain 27 |
| mfnc.muryy | model.input.mur22 | 1 | Relative permeability, yy component | Domain 27 |
| mfnc.murzy | model.input.mur32 | 1 | Relative permeability, zy component | Domain 27 |
| mfnc.murxz | model.input.mur13 | 1 | Relative permeability, xz component | Domain 27 |
| mfnc.muryz | model.input.mur23 | 1 | Relative permeability, yz component | Domain 27 |
| mfnc.murzz | model.input.mur33 | 1 | Relative permeability, zz component | Domain 27 |
| mfnc.Bx | mu0\_const\*mfnc.Ixx\*mfnc.Hx+mu0\_const\*mfnc.Ixy\*mfnc.Hy+mu0\_const\*mfnc.Ixz\*mfnc.Hz+mu0\_const\*mfnc.chimxx\*mfnc.Hx+mu0\_const\*mfnc.chimxy\*mfnc.Hy+mu0\_const\*mfnc.chimxz\*mfnc.Hz+mfnc.Brx | T | Magnetic flux density, x component | Domain 27 |
| mfnc.By | mu0\_const\*mfnc.Iyx\*mfnc.Hx+mu0\_const\*mfnc.Iyy\*mfnc.Hy+mu0\_const\*mfnc.Iyz\*mfnc.Hz+mu0\_const\*mfnc.chimyx\*mfnc.Hx+mu0\_const\*mfnc.chimyy\*mfnc.Hy+mu0\_const\*mfnc.chimyz\*mfnc.Hz+mfnc.Bry | T | Magnetic flux density, y component | Domain 27 |
| mfnc.Bz | mu0\_const\*mfnc.Izx\*mfnc.Hx+mu0\_const\*mfnc.Izy\*mfnc.Hy+mu0\_const\*mfnc.Izz\*mfnc.Hz+mu0\_const\*mfnc.chimzx\*mfnc.Hx+mu0\_const\*mfnc.chimzy\*mfnc.Hy+mu0\_const\*mfnc.chimzz\*mfnc.Hz+mfnc.Brz | T | Magnetic flux density, z component | Domain 27 |
| mfnc.normB | sqrt(realdot(mfnc.Bx,mfnc.Bx)+realdot(mfnc.By,mfnc.By)+realdot(mfnc.Bz,mfnc.Bz)) | T | Magnetic flux density norm | Domain 27 |
| mfnc.Mx | mfnc.Bx/mu0\_const-mfnc.Ixx\*mfnc.Hx-mfnc.Ixy\*mfnc.Hy-mfnc.Ixz\*mfnc.Hz | A/m | Magnetization, x component | Domain 27 |
| mfnc.My | mfnc.By/mu0\_const-mfnc.Iyx\*mfnc.Hx-mfnc.Iyy\*mfnc.Hy-mfnc.Iyz\*mfnc.Hz | A/m | Magnetization, y component | Domain 27 |
| mfnc.Mz | mfnc.Bz/mu0\_const-mfnc.Izx\*mfnc.Hx-mfnc.Izy\*mfnc.Hy-mfnc.Izz\*mfnc.Hz | A/m | Magnetization, z component | Domain 27 |
| mfnc.normM | sqrt(realdot(mfnc.Mx,mfnc.Mx)+realdot(mfnc.My,mfnc.My)+realdot(mfnc.Mz,mfnc.Mz)) | A/m | Magnetization norm | Domain 27 |
| mfnc.Ixx | 1 | 1 | Spatial identity matrix, xx component | Domain 27 |
| mfnc.Iyx | 0 | 1 | Spatial identity matrix, yx component | Domain 27 |
| mfnc.Izx | 0 | 1 | Spatial identity matrix, zx component | Domain 27 |
| mfnc.Ixy | 0 | 1 | Spatial identity matrix, xy component | Domain 27 |
| mfnc.Iyy | 1 | 1 | Spatial identity matrix, yy component | Domain 27 |
| mfnc.Izy | 0 | 1 | Spatial identity matrix, zy component | Domain 27 |
| mfnc.Ixz | 0 | 1 | Spatial identity matrix, xz component | Domain 27 |
| mfnc.Iyz | 0 | 1 | Spatial identity matrix, yz component | Domain 27 |
| mfnc.Izz | 1 | 1 | Spatial identity matrix, zz component | Domain 27 |
| mfnc.chimxx | -1+mfnc.murxx | 1 | Magnetic susceptibility, xx component | Domain 27 |
| mfnc.chimyx | mfnc.muryx | 1 | Magnetic susceptibility, yx component | Domain 27 |
| mfnc.chimzx | mfnc.murzx | 1 | Magnetic susceptibility, zx component | Domain 27 |
| mfnc.chimxy | mfnc.murxy | 1 | Magnetic susceptibility, xy component | Domain 27 |
| mfnc.chimyy | -1+mfnc.muryy | 1 | Magnetic susceptibility, yy component | Domain 27 |
| mfnc.chimzy | mfnc.murzy | 1 | Magnetic susceptibility, zy component | Domain 27 |
| mfnc.chimxz | mfnc.murxz | 1 | Magnetic susceptibility, xz component | Domain 27 |
| mfnc.chimyz | mfnc.muryz | 1 | Magnetic susceptibility, yz component | Domain 27 |
| mfnc.chimzz | -1+mfnc.murzz | 1 | Magnetic susceptibility, zz component | Domain 27 |
| mfnc.Brx | BB\*cos(pi\*(660+corr\_b)/180) | T | Remanent flux density, x component | Domain 27 |
| mfnc.Bry | BB\*sin(pi\*(660+corr\_b)/180) | T | Remanent flux density, y component | Domain 27 |
| mfnc.Brz | 0 | T | Remanent flux density, z component | Domain 27 |
| mfnc.normBr | sqrt(realdot(mfnc.Brx,mfnc.Brx)+realdot(mfnc.Bry,mfnc.Bry)+realdot(mfnc.Brz,mfnc.Brz)) | T | Remanent flux density norm | Domain 27 |
| mfnc.unTx | 0 | Pa | Maxwell upward surface stress tensor, x component | Boundaries 250–253, 258, 260, 262, 266, 268, 270 |
| mfnc.unTy | 0 | Pa | Maxwell upward surface stress tensor, y component | Boundaries 250–253, 258, 260, 262, 266, 268, 270 |
| mfnc.unTz | 0 | Pa | Maxwell upward surface stress tensor, z component | Boundaries 250–253, 258, 260, 262, 266, 268, 270 |
| mfnc.dnTx | mfnc.dnTmx | Pa | Maxwell downward surface stress tensor, x component | Boundaries 250–253, 258, 260, 262, 266, 268, 270 |
| mfnc.dnTy | mfnc.dnTmy | Pa | Maxwell downward surface stress tensor, y component | Boundaries 250–253, 258, 260, 262, 266, 268, 270 |
| mfnc.dnTz | mfnc.dnTmz | Pa | Maxwell downward surface stress tensor, z component | Boundaries 250–253, 258, 260, 262, 266, 268, 270 |
| mfnc.unx | unx |  | Normal vector up direction, x component | Boundaries 250–253, 258, 260, 262, 266, 268, 270 |
| mfnc.uny | uny |  | Normal vector up direction, y component | Boundaries 250–253, 258, 260, 262, 266, 268, 270 |
| mfnc.unz | unz |  | Normal vector up direction, z component | Boundaries 250–253, 258, 260, 262, 266, 268, 270 |
| mfnc.dnx | dnx |  | Normal vector down direction, x component | Boundaries 250–253, 258, 260, 262, 266, 268, 270 |
| mfnc.dny | dny |  | Normal vector down direction, y component | Boundaries 250–253, 258, 260, 262, 266, 268, 270 |
| mfnc.dnz | dnz |  | Normal vector down direction, z component | Boundaries 250–253, 258, 260, 262, 266, 268, 270 |
| mfnc.W | mfnc.Wm | J/m^3 | Energy density | Domain 27 |
| mfnc.dWm | mfnc.Wm | J/m^3 | Integrand for total magnetic energy | Domain 27 |
| mfnc.Wm | 0.5\*mu0\_const\*((mfnc.murxx\*mfnc.Hx+mfnc.murxy\*mfnc.Hy+mfnc.murxz\*mfnc.Hz)\*mfnc.Hx+(mfnc.muryx\*mfnc.Hx+mfnc.muryy\*mfnc.Hy+mfnc.muryz\*mfnc.Hz)\*mfnc.Hy+(mfnc.murzx\*mfnc.Hx+mfnc.murzy\*mfnc.Hy+mfnc.murzz\*mfnc.Hz)\*mfnc.Hz) | J/m^3 | Magnetic energy density | Domain 27 |

##### Shape Functions

| Name | Shape function | Unit | Description | Shape frame | Selection |
| --- | --- | --- | --- | --- | --- |
| Vm | Lagrange (Quadratic) | A | Magnetic scalar potential | Material | Domain 27 |

##### Weak Expressions

| Weak expression | Integration frame | Selection |
| --- | --- | --- |
| mfnc.d\*(-mfnc.Bx\*test(Vmx)-mfnc.By\*test(Vmy)-mfnc.Bz\*test(Vmz)) | Material | Domain 27 |

#### 2.4.16. ArrayCMagnet1

ArrayCMagnet1

Selection

| Geometric entity level | Domain |
| Selection | Domain 26 |

Equations

Settings

| Description | Value |
| Constitutive relation | Remanent flux density |
| Remanent flux density, x component | BC\*cos(2\*pi/360\*(0\*60 + corr\_c)) |
| Remanent flux density, y component | BC\*sin(2\*pi/360\*(0\*60 + corr\_c)) |
| Remanent flux density, z component | 0 |
| Relative permeability | From material |
| Relative permeability | {{1, 0, 0}, {0, 1, 0}, {0, 0, 1}} |

Properties from material

| Property | Material | Property group |
| Relative permeability | Soft Iron (without losses) | Basic |

##### Variables

| Name | Expression | Unit | Description | Selection |
| --- | --- | --- | --- | --- |
| mfnc.dnTmx | -0.5\*mfnc.unx\*(real(down(mfnc.Bx))\*real(down(mfnc.Hx))+real(down(mfnc.By))\*real(down(mfnc.Hy))+real(down(mfnc.Bz))\*real(down(mfnc.Hz)))+real(down(mfnc.Bx))\*(real(down(mfnc.Hx))\*mfnc.unx+real(down(mfnc.Hy))\*mfnc.uny+real(down(mfnc.Hz))\*mfnc.unz) | Pa | Maxwell downward magnetic surface stress tensor, x component | Boundaries 236–245 |
| mfnc.dnTmy | -0.5\*mfnc.uny\*(real(down(mfnc.Bx))\*real(down(mfnc.Hx))+real(down(mfnc.By))\*real(down(mfnc.Hy))+real(down(mfnc.Bz))\*real(down(mfnc.Hz)))+real(down(mfnc.By))\*(real(down(mfnc.Hx))\*mfnc.unx+real(down(mfnc.Hy))\*mfnc.uny+real(down(mfnc.Hz))\*mfnc.unz) | Pa | Maxwell downward magnetic surface stress tensor, y component | Boundaries 236–245 |
| mfnc.dnTmz | -0.5\*mfnc.unz\*(real(down(mfnc.Bx))\*real(down(mfnc.Hx))+real(down(mfnc.By))\*real(down(mfnc.Hy))+real(down(mfnc.Bz))\*real(down(mfnc.Hz)))+real(down(mfnc.Bz))\*(real(down(mfnc.Hx))\*mfnc.unx+real(down(mfnc.Hy))\*mfnc.uny+real(down(mfnc.Hz))\*mfnc.unz) | Pa | Maxwell downward magnetic surface stress tensor, z component | Boundaries 236–245 |
| mfnc.Hx | -Vmx | A/m | Magnetic field, x component | Domain 26 |
| mfnc.Hy | -Vmy | A/m | Magnetic field, y component | Domain 26 |
| mfnc.Hz | -Vmz | A/m | Magnetic field, z component | Domain 26 |
| mfnc.tHx | -VmTx | A/m | Tangential magnetic field, x component | Boundaries 236–245 |
| mfnc.tHy | -VmTy | A/m | Tangential magnetic field, y component | Boundaries 236–245 |
| mfnc.tHz | -VmTz | A/m | Tangential magnetic field, z component | Boundaries 236–245 |
| mfnc.normH | sqrt(realdot(mfnc.Hx,mfnc.Hx)+realdot(mfnc.Hy,mfnc.Hy)+realdot(mfnc.Hz,mfnc.Hz)) | A/m | Magnetic field norm | Domain 26 |
| mfnc.murxx | model.input.mur11 | 1 | Relative permeability, xx component | Domain 26 |
| mfnc.muryx | model.input.mur21 | 1 | Relative permeability, yx component | Domain 26 |
| mfnc.murzx | model.input.mur31 | 1 | Relative permeability, zx component | Domain 26 |
| mfnc.murxy | model.input.mur12 | 1 | Relative permeability, xy component | Domain 26 |
| mfnc.muryy | model.input.mur22 | 1 | Relative permeability, yy component | Domain 26 |
| mfnc.murzy | model.input.mur32 | 1 | Relative permeability, zy component | Domain 26 |
| mfnc.murxz | model.input.mur13 | 1 | Relative permeability, xz component | Domain 26 |
| mfnc.muryz | model.input.mur23 | 1 | Relative permeability, yz component | Domain 26 |
| mfnc.murzz | model.input.mur33 | 1 | Relative permeability, zz component | Domain 26 |
| mfnc.Bx | mu0\_const\*mfnc.Ixx\*mfnc.Hx+mu0\_const\*mfnc.Ixy\*mfnc.Hy+mu0\_const\*mfnc.Ixz\*mfnc.Hz+mu0\_const\*mfnc.chimxx\*mfnc.Hx+mu0\_const\*mfnc.chimxy\*mfnc.Hy+mu0\_const\*mfnc.chimxz\*mfnc.Hz+mfnc.Brx | T | Magnetic flux density, x component | Domain 26 |
| mfnc.By | mu0\_const\*mfnc.Iyx\*mfnc.Hx+mu0\_const\*mfnc.Iyy\*mfnc.Hy+mu0\_const\*mfnc.Iyz\*mfnc.Hz+mu0\_const\*mfnc.chimyx\*mfnc.Hx+mu0\_const\*mfnc.chimyy\*mfnc.Hy+mu0\_const\*mfnc.chimyz\*mfnc.Hz+mfnc.Bry | T | Magnetic flux density, y component | Domain 26 |
| mfnc.Bz | mu0\_const\*mfnc.Izx\*mfnc.Hx+mu0\_const\*mfnc.Izy\*mfnc.Hy+mu0\_const\*mfnc.Izz\*mfnc.Hz+mu0\_const\*mfnc.chimzx\*mfnc.Hx+mu0\_const\*mfnc.chimzy\*mfnc.Hy+mu0\_const\*mfnc.chimzz\*mfnc.Hz+mfnc.Brz | T | Magnetic flux density, z component | Domain 26 |
| mfnc.normB | sqrt(realdot(mfnc.Bx,mfnc.Bx)+realdot(mfnc.By,mfnc.By)+realdot(mfnc.Bz,mfnc.Bz)) | T | Magnetic flux density norm | Domain 26 |
| mfnc.Mx | mfnc.Bx/mu0\_const-mfnc.Ixx\*mfnc.Hx-mfnc.Ixy\*mfnc.Hy-mfnc.Ixz\*mfnc.Hz | A/m | Magnetization, x component | Domain 26 |
| mfnc.My | mfnc.By/mu0\_const-mfnc.Iyx\*mfnc.Hx-mfnc.Iyy\*mfnc.Hy-mfnc.Iyz\*mfnc.Hz | A/m | Magnetization, y component | Domain 26 |
| mfnc.Mz | mfnc.Bz/mu0\_const-mfnc.Izx\*mfnc.Hx-mfnc.Izy\*mfnc.Hy-mfnc.Izz\*mfnc.Hz | A/m | Magnetization, z component | Domain 26 |
| mfnc.normM | sqrt(realdot(mfnc.Mx,mfnc.Mx)+realdot(mfnc.My,mfnc.My)+realdot(mfnc.Mz,mfnc.Mz)) | A/m | Magnetization norm | Domain 26 |
| mfnc.Ixx | 1 | 1 | Spatial identity matrix, xx component | Domain 26 |
| mfnc.Iyx | 0 | 1 | Spatial identity matrix, yx component | Domain 26 |
| mfnc.Izx | 0 | 1 | Spatial identity matrix, zx component | Domain 26 |
| mfnc.Ixy | 0 | 1 | Spatial identity matrix, xy component | Domain 26 |
| mfnc.Iyy | 1 | 1 | Spatial identity matrix, yy component | Domain 26 |
| mfnc.Izy | 0 | 1 | Spatial identity matrix, zy component | Domain 26 |
| mfnc.Ixz | 0 | 1 | Spatial identity matrix, xz component | Domain 26 |
| mfnc.Iyz | 0 | 1 | Spatial identity matrix, yz component | Domain 26 |
| mfnc.Izz | 1 | 1 | Spatial identity matrix, zz component | Domain 26 |
| mfnc.chimxx | -1+mfnc.murxx | 1 | Magnetic susceptibility, xx component | Domain 26 |
| mfnc.chimyx | mfnc.muryx | 1 | Magnetic susceptibility, yx component | Domain 26 |
| mfnc.chimzx | mfnc.murzx | 1 | Magnetic susceptibility, zx component | Domain 26 |
| mfnc.chimxy | mfnc.murxy | 1 | Magnetic susceptibility, xy component | Domain 26 |
| mfnc.chimyy | -1+mfnc.muryy | 1 | Magnetic susceptibility, yy component | Domain 26 |
| mfnc.chimzy | mfnc.murzy | 1 | Magnetic susceptibility, zy component | Domain 26 |
| mfnc.chimxz | mfnc.murxz | 1 | Magnetic susceptibility, xz component | Domain 26 |
| mfnc.chimyz | mfnc.muryz | 1 | Magnetic susceptibility, yz component | Domain 26 |
| mfnc.chimzz | -1+mfnc.murzz | 1 | Magnetic susceptibility, zz component | Domain 26 |
| mfnc.Brx | BC\*cos(pi\*corr\_c/180) | T | Remanent flux density, x component | Domain 26 |
| mfnc.Bry | BC\*sin(pi\*corr\_c/180) | T | Remanent flux density, y component | Domain 26 |
| mfnc.Brz | 0 | T | Remanent flux density, z component | Domain 26 |
| mfnc.normBr | sqrt(realdot(mfnc.Brx,mfnc.Brx)+realdot(mfnc.Bry,mfnc.Bry)+realdot(mfnc.Brz,mfnc.Brz)) | T | Remanent flux density norm | Domain 26 |
| mfnc.unTx | 0 | Pa | Maxwell upward surface stress tensor, x component | Boundaries 236–245 |
| mfnc.unTy | 0 | Pa | Maxwell upward surface stress tensor, y component | Boundaries 236–245 |
| mfnc.unTz | 0 | Pa | Maxwell upward surface stress tensor, z component | Boundaries 236–245 |
| mfnc.dnTx | mfnc.dnTmx | Pa | Maxwell downward surface stress tensor, x component | Boundaries 236–245 |
| mfnc.dnTy | mfnc.dnTmy | Pa | Maxwell downward surface stress tensor, y component | Boundaries 236–245 |
| mfnc.dnTz | mfnc.dnTmz | Pa | Maxwell downward surface stress tensor, z component | Boundaries 236–245 |
| mfnc.unx | unx |  | Normal vector up direction, x component | Boundaries 236–245 |
| mfnc.uny | uny |  | Normal vector up direction, y component | Boundaries 236–245 |
| mfnc.unz | unz |  | Normal vector up direction, z component | Boundaries 236–245 |
| mfnc.dnx | dnx |  | Normal vector down direction, x component | Boundaries 236–245 |
| mfnc.dny | dny |  | Normal vector down direction, y component | Boundaries 236–245 |
| mfnc.dnz | dnz |  | Normal vector down direction, z component | Boundaries 236–245 |
| mfnc.W | mfnc.Wm | J/m^3 | Energy density | Domain 26 |
| mfnc.dWm | mfnc.Wm | J/m^3 | Integrand for total magnetic energy | Domain 26 |
| mfnc.Wm | 0.5\*mu0\_const\*((mfnc.murxx\*mfnc.Hx+mfnc.murxy\*mfnc.Hy+mfnc.murxz\*mfnc.Hz)\*mfnc.Hx+(mfnc.muryx\*mfnc.Hx+mfnc.muryy\*mfnc.Hy+mfnc.muryz\*mfnc.Hz)\*mfnc.Hy+(mfnc.murzx\*mfnc.Hx+mfnc.murzy\*mfnc.Hy+mfnc.murzz\*mfnc.Hz)\*mfnc.Hz) | J/m^3 | Magnetic energy density | Domain 26 |

##### Shape Functions

| Name | Shape function | Unit | Description | Shape frame | Selection |
| --- | --- | --- | --- | --- | --- |
| Vm | Lagrange (Quadratic) | A | Magnetic scalar potential | Material | Domain 26 |

##### Weak Expressions

| Weak expression | Integration frame | Selection |
| --- | --- | --- |
| mfnc.d\*(-mfnc.Bx\*test(Vmx)-mfnc.By\*test(Vmy)-mfnc.Bz\*test(Vmz)) | Material | Domain 26 |

#### 2.4.17. ArrayCMagnet3

ArrayCMagnet3

Selection

| Geometric entity level | Domain |
| Selection | Domain 21 |

Equations

Settings

| Description | Value |
| Constitutive relation | Remanent flux density |
| Remanent flux density, x component | BC\*cos(2\*pi/360\*(2\*60 + corr\_c)) |
| Remanent flux density, y component | BC\*sin(2\*pi/360\*(2\*60 + corr\_c)) |
| Remanent flux density, z component | 0 |
| Relative permeability | From material |
| Relative permeability | {{1, 0, 0}, {0, 1, 0}, {0, 0, 1}} |

Properties from material

| Property | Material | Property group |
| Relative permeability | Soft Iron (without losses) | Basic |

##### Variables

| Name | Expression | Unit | Description | Selection |
| --- | --- | --- | --- | --- |
| mfnc.dnTmx | -0.5\*mfnc.unx\*(real(down(mfnc.Bx))\*real(down(mfnc.Hx))+real(down(mfnc.By))\*real(down(mfnc.Hy))+real(down(mfnc.Bz))\*real(down(mfnc.Hz)))+real(down(mfnc.Bx))\*(real(down(mfnc.Hx))\*mfnc.unx+real(down(mfnc.Hy))\*mfnc.uny+real(down(mfnc.Hz))\*mfnc.unz) | Pa | Maxwell downward magnetic surface stress tensor, x component | Boundaries 186–189, 191, 193, 195, 197, 199, 201 |
| mfnc.dnTmy | -0.5\*mfnc.uny\*(real(down(mfnc.Bx))\*real(down(mfnc.Hx))+real(down(mfnc.By))\*real(down(mfnc.Hy))+real(down(mfnc.Bz))\*real(down(mfnc.Hz)))+real(down(mfnc.By))\*(real(down(mfnc.Hx))\*mfnc.unx+real(down(mfnc.Hy))\*mfnc.uny+real(down(mfnc.Hz))\*mfnc.unz) | Pa | Maxwell downward magnetic surface stress tensor, y component | Boundaries 186–189, 191, 193, 195, 197, 199, 201 |
| mfnc.dnTmz | -0.5\*mfnc.unz\*(real(down(mfnc.Bx))\*real(down(mfnc.Hx))+real(down(mfnc.By))\*real(down(mfnc.Hy))+real(down(mfnc.Bz))\*real(down(mfnc.Hz)))+real(down(mfnc.Bz))\*(real(down(mfnc.Hx))\*mfnc.unx+real(down(mfnc.Hy))\*mfnc.uny+real(down(mfnc.Hz))\*mfnc.unz) | Pa | Maxwell downward magnetic surface stress tensor, z component | Boundaries 186–189, 191, 193, 195, 197, 199, 201 |
| mfnc.Hx | -Vmx | A/m | Magnetic field, x component | Domain 21 |
| mfnc.Hy | -Vmy | A/m | Magnetic field, y component | Domain 21 |
| mfnc.Hz | -Vmz | A/m | Magnetic field, z component | Domain 21 |
| mfnc.tHx | -VmTx | A/m | Tangential magnetic field, x component | Boundaries 186–189, 191, 193, 195, 197, 199, 201 |
| mfnc.tHy | -VmTy | A/m | Tangential magnetic field, y component | Boundaries 186–189, 191, 193, 195, 197, 199, 201 |
| mfnc.tHz | -VmTz | A/m | Tangential magnetic field, z component | Boundaries 186–189, 191, 193, 195, 197, 199, 201 |
| mfnc.normH | sqrt(realdot(mfnc.Hx,mfnc.Hx)+realdot(mfnc.Hy,mfnc.Hy)+realdot(mfnc.Hz,mfnc.Hz)) | A/m | Magnetic field norm | Domain 21 |
| mfnc.murxx | model.input.mur11 | 1 | Relative permeability, xx component | Domain 21 |
| mfnc.muryx | model.input.mur21 | 1 | Relative permeability, yx component | Domain 21 |
| mfnc.murzx | model.input.mur31 | 1 | Relative permeability, zx component | Domain 21 |
| mfnc.murxy | model.input.mur12 | 1 | Relative permeability, xy component | Domain 21 |
| mfnc.muryy | model.input.mur22 | 1 | Relative permeability, yy component | Domain 21 |
| mfnc.murzy | model.input.mur32 | 1 | Relative permeability, zy component | Domain 21 |
| mfnc.murxz | model.input.mur13 | 1 | Relative permeability, xz component | Domain 21 |
| mfnc.muryz | model.input.mur23 | 1 | Relative permeability, yz component | Domain 21 |
| mfnc.murzz | model.input.mur33 | 1 | Relative permeability, zz component | Domain 21 |
| mfnc.Bx | mu0\_const\*mfnc.Ixx\*mfnc.Hx+mu0\_const\*mfnc.Ixy\*mfnc.Hy+mu0\_const\*mfnc.Ixz\*mfnc.Hz+mu0\_const\*mfnc.chimxx\*mfnc.Hx+mu0\_const\*mfnc.chimxy\*mfnc.Hy+mu0\_const\*mfnc.chimxz\*mfnc.Hz+mfnc.Brx | T | Magnetic flux density, x component | Domain 21 |
| mfnc.By | mu0\_const\*mfnc.Iyx\*mfnc.Hx+mu0\_const\*mfnc.Iyy\*mfnc.Hy+mu0\_const\*mfnc.Iyz\*mfnc.Hz+mu0\_const\*mfnc.chimyx\*mfnc.Hx+mu0\_const\*mfnc.chimyy\*mfnc.Hy+mu0\_const\*mfnc.chimyz\*mfnc.Hz+mfnc.Bry | T | Magnetic flux density, y component | Domain 21 |
| mfnc.Bz | mu0\_const\*mfnc.Izx\*mfnc.Hx+mu0\_const\*mfnc.Izy\*mfnc.Hy+mu0\_const\*mfnc.Izz\*mfnc.Hz+mu0\_const\*mfnc.chimzx\*mfnc.Hx+mu0\_const\*mfnc.chimzy\*mfnc.Hy+mu0\_const\*mfnc.chimzz\*mfnc.Hz+mfnc.Brz | T | Magnetic flux density, z component | Domain 21 |
| mfnc.normB | sqrt(realdot(mfnc.Bx,mfnc.Bx)+realdot(mfnc.By,mfnc.By)+realdot(mfnc.Bz,mfnc.Bz)) | T | Magnetic flux density norm | Domain 21 |
| mfnc.Mx | mfnc.Bx/mu0\_const-mfnc.Ixx\*mfnc.Hx-mfnc.Ixy\*mfnc.Hy-mfnc.Ixz\*mfnc.Hz | A/m | Magnetization, x component | Domain 21 |
| mfnc.My | mfnc.By/mu0\_const-mfnc.Iyx\*mfnc.Hx-mfnc.Iyy\*mfnc.Hy-mfnc.Iyz\*mfnc.Hz | A/m | Magnetization, y component | Domain 21 |
| mfnc.Mz | mfnc.Bz/mu0\_const-mfnc.Izx\*mfnc.Hx-mfnc.Izy\*mfnc.Hy-mfnc.Izz\*mfnc.Hz | A/m | Magnetization, z component | Domain 21 |
| mfnc.normM | sqrt(realdot(mfnc.Mx,mfnc.Mx)+realdot(mfnc.My,mfnc.My)+realdot(mfnc.Mz,mfnc.Mz)) | A/m | Magnetization norm | Domain 21 |
| mfnc.Ixx | 1 | 1 | Spatial identity matrix, xx component | Domain 21 |
| mfnc.Iyx | 0 | 1 | Spatial identity matrix, yx component | Domain 21 |
| mfnc.Izx | 0 | 1 | Spatial identity matrix, zx component | Domain 21 |
| mfnc.Ixy | 0 | 1 | Spatial identity matrix, xy component | Domain 21 |
| mfnc.Iyy | 1 | 1 | Spatial identity matrix, yy component | Domain 21 |
| mfnc.Izy | 0 | 1 | Spatial identity matrix, zy component | Domain 21 |
| mfnc.Ixz | 0 | 1 | Spatial identity matrix, xz component | Domain 21 |
| mfnc.Iyz | 0 | 1 | Spatial identity matrix, yz component | Domain 21 |
| mfnc.Izz | 1 | 1 | Spatial identity matrix, zz component | Domain 21 |
| mfnc.chimxx | -1+mfnc.murxx | 1 | Magnetic susceptibility, xx component | Domain 21 |
| mfnc.chimyx | mfnc.muryx | 1 | Magnetic susceptibility, yx component | Domain 21 |
| mfnc.chimzx | mfnc.murzx | 1 | Magnetic susceptibility, zx component | Domain 21 |
| mfnc.chimxy | mfnc.murxy | 1 | Magnetic susceptibility, xy component | Domain 21 |
| mfnc.chimyy | -1+mfnc.muryy | 1 | Magnetic susceptibility, yy component | Domain 21 |
| mfnc.chimzy | mfnc.murzy | 1 | Magnetic susceptibility, zy component | Domain 21 |
| mfnc.chimxz | mfnc.murxz | 1 | Magnetic susceptibility, xz component | Domain 21 |
| mfnc.chimyz | mfnc.muryz | 1 | Magnetic susceptibility, yz component | Domain 21 |
| mfnc.chimzz | -1+mfnc.murzz | 1 | Magnetic susceptibility, zz component | Domain 21 |
| mfnc.Brx | BC\*cos(pi\*(120+corr\_c)/180) | T | Remanent flux density, x component | Domain 21 |
| mfnc.Bry | BC\*sin(pi\*(120+corr\_c)/180) | T | Remanent flux density, y component | Domain 21 |
| mfnc.Brz | 0 | T | Remanent flux density, z component | Domain 21 |
| mfnc.normBr | sqrt(realdot(mfnc.Brx,mfnc.Brx)+realdot(mfnc.Bry,mfnc.Bry)+realdot(mfnc.Brz,mfnc.Brz)) | T | Remanent flux density norm | Domain 21 |
| mfnc.unTx | 0 | Pa | Maxwell upward surface stress tensor, x component | Boundaries 186–189, 191, 193, 195, 197, 199, 201 |
| mfnc.unTy | 0 | Pa | Maxwell upward surface stress tensor, y component | Boundaries 186–189, 191, 193, 195, 197, 199, 201 |
| mfnc.unTz | 0 | Pa | Maxwell upward surface stress tensor, z component | Boundaries 186–189, 191, 193, 195, 197, 199, 201 |
| mfnc.dnTx | mfnc.dnTmx | Pa | Maxwell downward surface stress tensor, x component | Boundaries 186–189, 191, 193, 195, 197, 199, 201 |
| mfnc.dnTy | mfnc.dnTmy | Pa | Maxwell downward surface stress tensor, y component | Boundaries 186–189, 191, 193, 195, 197, 199, 201 |
| mfnc.dnTz | mfnc.dnTmz | Pa | Maxwell downward surface stress tensor, z component | Boundaries 186–189, 191, 193, 195, 197, 199, 201 |
| mfnc.unx | unx |  | Normal vector up direction, x component | Boundaries 186–189, 191, 193, 195, 197, 199, 201 |
| mfnc.uny | uny |  | Normal vector up direction, y component | Boundaries 186–189, 191, 193, 195, 197, 199, 201 |
| mfnc.unz | unz |  | Normal vector up direction, z component | Boundaries 186–189, 191, 193, 195, 197, 199, 201 |
| mfnc.dnx | dnx |  | Normal vector down direction, x component | Boundaries 186–189, 191, 193, 195, 197, 199, 201 |
| mfnc.dny | dny |  | Normal vector down direction, y component | Boundaries 186–189, 191, 193, 195, 197, 199, 201 |
| mfnc.dnz | dnz |  | Normal vector down direction, z component | Boundaries 186–189, 191, 193, 195, 197, 199, 201 |
| mfnc.W | mfnc.Wm | J/m^3 | Energy density | Domain 21 |
| mfnc.dWm | mfnc.Wm | J/m^3 | Integrand for total magnetic energy | Domain 21 |
| mfnc.Wm | 0.5\*mu0\_const\*((mfnc.murxx\*mfnc.Hx+mfnc.murxy\*mfnc.Hy+mfnc.murxz\*mfnc.Hz)\*mfnc.Hx+(mfnc.muryx\*mfnc.Hx+mfnc.muryy\*mfnc.Hy+mfnc.muryz\*mfnc.Hz)\*mfnc.Hy+(mfnc.murzx\*mfnc.Hx+mfnc.murzy\*mfnc.Hy+mfnc.murzz\*mfnc.Hz)\*mfnc.Hz) | J/m^3 | Magnetic energy density | Domain 21 |

##### Shape Functions

| Name | Shape function | Unit | Description | Shape frame | Selection |
| --- | --- | --- | --- | --- | --- |
| Vm | Lagrange (Quadratic) | A | Magnetic scalar potential | Material | Domain 21 |

##### Weak Expressions

| Weak expression | Integration frame | Selection |
| --- | --- | --- |
| mfnc.d\*(-mfnc.Bx\*test(Vmx)-mfnc.By\*test(Vmy)-mfnc.Bz\*test(Vmz)) | Material | Domain 21 |

#### 2.4.18. ArrayCMagnet5

ArrayCMagnet5

Selection

| Geometric entity level | Domain |
| Selection | Domain 14 |

Equations

Settings

| Description | Value |
| Constitutive relation | Remanent flux density |
| Remanent flux density, x component | BC\*cos(2\*pi/360\*(4\*60 + corr\_c)) |
| Remanent flux density, y component | BC\*sin(2\*pi/360\*(4\*60 + corr\_c)) |
| Remanent flux density, z component | 0 |
| Relative permeability | From material |
| Relative permeability | {{1, 0, 0}, {0, 1, 0}, {0, 0, 1}} |

Properties from material

| Property | Material | Property group |
| Relative permeability | Soft Iron (without losses) | Basic |

##### Variables

| Name | Expression | Unit | Description | Selection |
| --- | --- | --- | --- | --- |
| mfnc.dnTmx | -0.5\*mfnc.unx\*(real(down(mfnc.Bx))\*real(down(mfnc.Hx))+real(down(mfnc.By))\*real(down(mfnc.Hy))+real(down(mfnc.Bz))\*real(down(mfnc.Hz)))+real(down(mfnc.Bx))\*(real(down(mfnc.Hx))\*mfnc.unx+real(down(mfnc.Hy))\*mfnc.uny+real(down(mfnc.Hz))\*mfnc.unz) | Pa | Maxwell downward magnetic surface stress tensor, x component | Boundaries 120–123, 125, 127, 129, 131, 133, 135 |
| mfnc.dnTmy | -0.5\*mfnc.uny\*(real(down(mfnc.Bx))\*real(down(mfnc.Hx))+real(down(mfnc.By))\*real(down(mfnc.Hy))+real(down(mfnc.Bz))\*real(down(mfnc.Hz)))+real(down(mfnc.By))\*(real(down(mfnc.Hx))\*mfnc.unx+real(down(mfnc.Hy))\*mfnc.uny+real(down(mfnc.Hz))\*mfnc.unz) | Pa | Maxwell downward magnetic surface stress tensor, y component | Boundaries 120–123, 125, 127, 129, 131, 133, 135 |
| mfnc.dnTmz | -0.5\*mfnc.unz\*(real(down(mfnc.Bx))\*real(down(mfnc.Hx))+real(down(mfnc.By))\*real(down(mfnc.Hy))+real(down(mfnc.Bz))\*real(down(mfnc.Hz)))+real(down(mfnc.Bz))\*(real(down(mfnc.Hx))\*mfnc.unx+real(down(mfnc.Hy))\*mfnc.uny+real(down(mfnc.Hz))\*mfnc.unz) | Pa | Maxwell downward magnetic surface stress tensor, z component | Boundaries 120–123, 125, 127, 129, 131, 133, 135 |
| mfnc.Hx | -Vmx | A/m | Magnetic field, x component | Domain 14 |
| mfnc.Hy | -Vmy | A/m | Magnetic field, y component | Domain 14 |
| mfnc.Hz | -Vmz | A/m | Magnetic field, z component | Domain 14 |
| mfnc.tHx | -VmTx | A/m | Tangential magnetic field, x component | Boundaries 120–123, 125, 127, 129, 131, 133, 135 |
| mfnc.tHy | -VmTy | A/m | Tangential magnetic field, y component | Boundaries 120–123, 125, 127, 129, 131, 133, 135 |
| mfnc.tHz | -VmTz | A/m | Tangential magnetic field, z component | Boundaries 120–123, 125, 127, 129, 131, 133, 135 |
| mfnc.normH | sqrt(realdot(mfnc.Hx,mfnc.Hx)+realdot(mfnc.Hy,mfnc.Hy)+realdot(mfnc.Hz,mfnc.Hz)) | A/m | Magnetic field norm | Domain 14 |
| mfnc.murxx | model.input.mur11 | 1 | Relative permeability, xx component | Domain 14 |
| mfnc.muryx | model.input.mur21 | 1 | Relative permeability, yx component | Domain 14 |
| mfnc.murzx | model.input.mur31 | 1 | Relative permeability, zx component | Domain 14 |
| mfnc.murxy | model.input.mur12 | 1 | Relative permeability, xy component | Domain 14 |
| mfnc.muryy | model.input.mur22 | 1 | Relative permeability, yy component | Domain 14 |
| mfnc.murzy | model.input.mur32 | 1 | Relative permeability, zy component | Domain 14 |
| mfnc.murxz | model.input.mur13 | 1 | Relative permeability, xz component | Domain 14 |
| mfnc.muryz | model.input.mur23 | 1 | Relative permeability, yz component | Domain 14 |
| mfnc.murzz | model.input.mur33 | 1 | Relative permeability, zz component | Domain 14 |
| mfnc.Bx | mu0\_const\*mfnc.Ixx\*mfnc.Hx+mu0\_const\*mfnc.Ixy\*mfnc.Hy+mu0\_const\*mfnc.Ixz\*mfnc.Hz+mu0\_const\*mfnc.chimxx\*mfnc.Hx+mu0\_const\*mfnc.chimxy\*mfnc.Hy+mu0\_const\*mfnc.chimxz\*mfnc.Hz+mfnc.Brx | T | Magnetic flux density, x component | Domain 14 |
| mfnc.By | mu0\_const\*mfnc.Iyx\*mfnc.Hx+mu0\_const\*mfnc.Iyy\*mfnc.Hy+mu0\_const\*mfnc.Iyz\*mfnc.Hz+mu0\_const\*mfnc.chimyx\*mfnc.Hx+mu0\_const\*mfnc.chimyy\*mfnc.Hy+mu0\_const\*mfnc.chimyz\*mfnc.Hz+mfnc.Bry | T | Magnetic flux density, y component | Domain 14 |
| mfnc.Bz | mu0\_const\*mfnc.Izx\*mfnc.Hx+mu0\_const\*mfnc.Izy\*mfnc.Hy+mu0\_const\*mfnc.Izz\*mfnc.Hz+mu0\_const\*mfnc.chimzx\*mfnc.Hx+mu0\_const\*mfnc.chimzy\*mfnc.Hy+mu0\_const\*mfnc.chimzz\*mfnc.Hz+mfnc.Brz | T | Magnetic flux density, z component | Domain 14 |
| mfnc.normB | sqrt(realdot(mfnc.Bx,mfnc.Bx)+realdot(mfnc.By,mfnc.By)+realdot(mfnc.Bz,mfnc.Bz)) | T | Magnetic flux density norm | Domain 14 |
| mfnc.Mx | mfnc.Bx/mu0\_const-mfnc.Ixx\*mfnc.Hx-mfnc.Ixy\*mfnc.Hy-mfnc.Ixz\*mfnc.Hz | A/m | Magnetization, x component | Domain 14 |
| mfnc.My | mfnc.By/mu0\_const-mfnc.Iyx\*mfnc.Hx-mfnc.Iyy\*mfnc.Hy-mfnc.Iyz\*mfnc.Hz | A/m | Magnetization, y component | Domain 14 |
| mfnc.Mz | mfnc.Bz/mu0\_const-mfnc.Izx\*mfnc.Hx-mfnc.Izy\*mfnc.Hy-mfnc.Izz\*mfnc.Hz | A/m | Magnetization, z component | Domain 14 |
| mfnc.normM | sqrt(realdot(mfnc.Mx,mfnc.Mx)+realdot(mfnc.My,mfnc.My)+realdot(mfnc.Mz,mfnc.Mz)) | A/m | Magnetization norm | Domain 14 |
| mfnc.Ixx | 1 | 1 | Spatial identity matrix, xx component | Domain 14 |
| mfnc.Iyx | 0 | 1 | Spatial identity matrix, yx component | Domain 14 |
| mfnc.Izx | 0 | 1 | Spatial identity matrix, zx component | Domain 14 |
| mfnc.Ixy | 0 | 1 | Spatial identity matrix, xy component | Domain 14 |
| mfnc.Iyy | 1 | 1 | Spatial identity matrix, yy component | Domain 14 |
| mfnc.Izy | 0 | 1 | Spatial identity matrix, zy component | Domain 14 |
| mfnc.Ixz | 0 | 1 | Spatial identity matrix, xz component | Domain 14 |
| mfnc.Iyz | 0 | 1 | Spatial identity matrix, yz component | Domain 14 |
| mfnc.Izz | 1 | 1 | Spatial identity matrix, zz component | Domain 14 |
| mfnc.chimxx | -1+mfnc.murxx | 1 | Magnetic susceptibility, xx component | Domain 14 |
| mfnc.chimyx | mfnc.muryx | 1 | Magnetic susceptibility, yx component | Domain 14 |
| mfnc.chimzx | mfnc.murzx | 1 | Magnetic susceptibility, zx component | Domain 14 |
| mfnc.chimxy | mfnc.murxy | 1 | Magnetic susceptibility, xy component | Domain 14 |
| mfnc.chimyy | -1+mfnc.muryy | 1 | Magnetic susceptibility, yy component | Domain 14 |
| mfnc.chimzy | mfnc.murzy | 1 | Magnetic susceptibility, zy component | Domain 14 |
| mfnc.chimxz | mfnc.murxz | 1 | Magnetic susceptibility, xz component | Domain 14 |
| mfnc.chimyz | mfnc.muryz | 1 | Magnetic susceptibility, yz component | Domain 14 |
| mfnc.chimzz | -1+mfnc.murzz | 1 | Magnetic susceptibility, zz component | Domain 14 |
| mfnc.Brx | BC\*cos(pi\*(240+corr\_c)/180) | T | Remanent flux density, x component | Domain 14 |
| mfnc.Bry | BC\*sin(pi\*(240+corr\_c)/180) | T | Remanent flux density, y component | Domain 14 |
| mfnc.Brz | 0 | T | Remanent flux density, z component | Domain 14 |
| mfnc.normBr | sqrt(realdot(mfnc.Brx,mfnc.Brx)+realdot(mfnc.Bry,mfnc.Bry)+realdot(mfnc.Brz,mfnc.Brz)) | T | Remanent flux density norm | Domain 14 |
| mfnc.unTx | 0 | Pa | Maxwell upward surface stress tensor, x component | Boundaries 120–123, 125, 127, 129, 131, 133, 135 |
| mfnc.unTy | 0 | Pa | Maxwell upward surface stress tensor, y component | Boundaries 120–123, 125, 127, 129, 131, 133, 135 |
| mfnc.unTz | 0 | Pa | Maxwell upward surface stress tensor, z component | Boundaries 120–123, 125, 127, 129, 131, 133, 135 |
| mfnc.dnTx | mfnc.dnTmx | Pa | Maxwell downward surface stress tensor, x component | Boundaries 120–123, 125, 127, 129, 131, 133, 135 |
| mfnc.dnTy | mfnc.dnTmy | Pa | Maxwell downward surface stress tensor, y component | Boundaries 120–123, 125, 127, 129, 131, 133, 135 |
| mfnc.dnTz | mfnc.dnTmz | Pa | Maxwell downward surface stress tensor, z component | Boundaries 120–123, 125, 127, 129, 131, 133, 135 |
| mfnc.unx | unx |  | Normal vector up direction, x component | Boundaries 120–123, 125, 127, 129, 131, 133, 135 |
| mfnc.uny | uny |  | Normal vector up direction, y component | Boundaries 120–123, 125, 127, 129, 131, 133, 135 |
| mfnc.unz | unz |  | Normal vector up direction, z component | Boundaries 120–123, 125, 127, 129, 131, 133, 135 |
| mfnc.dnx | dnx |  | Normal vector down direction, x component | Boundaries 120–123, 125, 127, 129, 131, 133, 135 |
| mfnc.dny | dny |  | Normal vector down direction, y component | Boundaries 120–123, 125, 127, 129, 131, 133, 135 |
| mfnc.dnz | dnz |  | Normal vector down direction, z component | Boundaries 120–123, 125, 127, 129, 131, 133, 135 |
| mfnc.W | mfnc.Wm | J/m^3 | Energy density | Domain 14 |
| mfnc.dWm | mfnc.Wm | J/m^3 | Integrand for total magnetic energy | Domain 14 |
| mfnc.Wm | 0.5\*mu0\_const\*((mfnc.murxx\*mfnc.Hx+mfnc.murxy\*mfnc.Hy+mfnc.murxz\*mfnc.Hz)\*mfnc.Hx+(mfnc.muryx\*mfnc.Hx+mfnc.muryy\*mfnc.Hy+mfnc.muryz\*mfnc.Hz)\*mfnc.Hy+(mfnc.murzx\*mfnc.Hx+mfnc.murzy\*mfnc.Hy+mfnc.murzz\*mfnc.Hz)\*mfnc.Hz) | J/m^3 | Magnetic energy density | Domain 14 |

##### Shape Functions

| Name | Shape function | Unit | Description | Shape frame | Selection |
| --- | --- | --- | --- | --- | --- |
| Vm | Lagrange (Quadratic) | A | Magnetic scalar potential | Material | Domain 14 |

##### Weak Expressions

| Weak expression | Integration frame | Selection |
| --- | --- | --- |
| mfnc.d\*(-mfnc.Bx\*test(Vmx)-mfnc.By\*test(Vmy)-mfnc.Bz\*test(Vmz)) | Material | Domain 14 |

#### 2.4.19. ArrayCMagnet7

ArrayCMagnet7

Selection

| Geometric entity level | Domain |
| Selection | Domain 10 |

Equations

Settings

| Description | Value |
| Constitutive relation | Remanent flux density |
| Remanent flux density, x component | BC\*cos(2\*pi/360\*(6\*60 + corr\_c)) |
| Remanent flux density, y component | BC\*sin(2\*pi/360\*(6\*60 + corr\_c)) |
| Remanent flux density, z component | 0 |
| Relative permeability | From material |
| Relative permeability | {{1, 0, 0}, {0, 1, 0}, {0, 0, 1}} |

Properties from material

| Property | Material | Property group |
| Relative permeability | Soft Iron (without losses) | Basic |

##### Variables

| Name | Expression | Unit | Description | Selection |
| --- | --- | --- | --- | --- |
| mfnc.dnTmx | -0.5\*mfnc.unx\*(real(down(mfnc.Bx))\*real(down(mfnc.Hx))+real(down(mfnc.By))\*real(down(mfnc.Hy))+real(down(mfnc.Bz))\*real(down(mfnc.Hz)))+real(down(mfnc.Bx))\*(real(down(mfnc.Hx))\*mfnc.unx+real(down(mfnc.Hy))\*mfnc.uny+real(down(mfnc.Hz))\*mfnc.unz) | Pa | Maxwell downward magnetic surface stress tensor, x component | Boundaries 80–89 |
| mfnc.dnTmy | -0.5\*mfnc.uny\*(real(down(mfnc.Bx))\*real(down(mfnc.Hx))+real(down(mfnc.By))\*real(down(mfnc.Hy))+real(down(mfnc.Bz))\*real(down(mfnc.Hz)))+real(down(mfnc.By))\*(real(down(mfnc.Hx))\*mfnc.unx+real(down(mfnc.Hy))\*mfnc.uny+real(down(mfnc.Hz))\*mfnc.unz) | Pa | Maxwell downward magnetic surface stress tensor, y component | Boundaries 80–89 |
| mfnc.dnTmz | -0.5\*mfnc.unz\*(real(down(mfnc.Bx))\*real(down(mfnc.Hx))+real(down(mfnc.By))\*real(down(mfnc.Hy))+real(down(mfnc.Bz))\*real(down(mfnc.Hz)))+real(down(mfnc.Bz))\*(real(down(mfnc.Hx))\*mfnc.unx+real(down(mfnc.Hy))\*mfnc.uny+real(down(mfnc.Hz))\*mfnc.unz) | Pa | Maxwell downward magnetic surface stress tensor, z component | Boundaries 80–89 |
| mfnc.Hx | -Vmx | A/m | Magnetic field, x component | Domain 10 |
| mfnc.Hy | -Vmy | A/m | Magnetic field, y component | Domain 10 |
| mfnc.Hz | -Vmz | A/m | Magnetic field, z component | Domain 10 |
| mfnc.tHx | -VmTx | A/m | Tangential magnetic field, x component | Boundaries 80–89 |
| mfnc.tHy | -VmTy | A/m | Tangential magnetic field, y component | Boundaries 80–89 |
| mfnc.tHz | -VmTz | A/m | Tangential magnetic field, z component | Boundaries 80–89 |
| mfnc.normH | sqrt(realdot(mfnc.Hx,mfnc.Hx)+realdot(mfnc.Hy,mfnc.Hy)+realdot(mfnc.Hz,mfnc.Hz)) | A/m | Magnetic field norm | Domain 10 |
| mfnc.murxx | model.input.mur11 | 1 | Relative permeability, xx component | Domain 10 |
| mfnc.muryx | model.input.mur21 | 1 | Relative permeability, yx component | Domain 10 |
| mfnc.murzx | model.input.mur31 | 1 | Relative permeability, zx component | Domain 10 |
| mfnc.murxy | model.input.mur12 | 1 | Relative permeability, xy component | Domain 10 |
| mfnc.muryy | model.input.mur22 | 1 | Relative permeability, yy component | Domain 10 |
| mfnc.murzy | model.input.mur32 | 1 | Relative permeability, zy component | Domain 10 |
| mfnc.murxz | model.input.mur13 | 1 | Relative permeability, xz component | Domain 10 |
| mfnc.muryz | model.input.mur23 | 1 | Relative permeability, yz component | Domain 10 |
| mfnc.murzz | model.input.mur33 | 1 | Relative permeability, zz component | Domain 10 |
| mfnc.Bx | mu0\_const\*mfnc.Ixx\*mfnc.Hx+mu0\_const\*mfnc.Ixy\*mfnc.Hy+mu0\_const\*mfnc.Ixz\*mfnc.Hz+mu0\_const\*mfnc.chimxx\*mfnc.Hx+mu0\_const\*mfnc.chimxy\*mfnc.Hy+mu0\_const\*mfnc.chimxz\*mfnc.Hz+mfnc.Brx | T | Magnetic flux density, x component | Domain 10 |
| mfnc.By | mu0\_const\*mfnc.Iyx\*mfnc.Hx+mu0\_const\*mfnc.Iyy\*mfnc.Hy+mu0\_const\*mfnc.Iyz\*mfnc.Hz+mu0\_const\*mfnc.chimyx\*mfnc.Hx+mu0\_const\*mfnc.chimyy\*mfnc.Hy+mu0\_const\*mfnc.chimyz\*mfnc.Hz+mfnc.Bry | T | Magnetic flux density, y component | Domain 10 |
| mfnc.Bz | mu0\_const\*mfnc.Izx\*mfnc.Hx+mu0\_const\*mfnc.Izy\*mfnc.Hy+mu0\_const\*mfnc.Izz\*mfnc.Hz+mu0\_const\*mfnc.chimzx\*mfnc.Hx+mu0\_const\*mfnc.chimzy\*mfnc.Hy+mu0\_const\*mfnc.chimzz\*mfnc.Hz+mfnc.Brz | T | Magnetic flux density, z component | Domain 10 |
| mfnc.normB | sqrt(realdot(mfnc.Bx,mfnc.Bx)+realdot(mfnc.By,mfnc.By)+realdot(mfnc.Bz,mfnc.Bz)) | T | Magnetic flux density norm | Domain 10 |
| mfnc.Mx | mfnc.Bx/mu0\_const-mfnc.Ixx\*mfnc.Hx-mfnc.Ixy\*mfnc.Hy-mfnc.Ixz\*mfnc.Hz | A/m | Magnetization, x component | Domain 10 |
| mfnc.My | mfnc.By/mu0\_const-mfnc.Iyx\*mfnc.Hx-mfnc.Iyy\*mfnc.Hy-mfnc.Iyz\*mfnc.Hz | A/m | Magnetization, y component | Domain 10 |
| mfnc.Mz | mfnc.Bz/mu0\_const-mfnc.Izx\*mfnc.Hx-mfnc.Izy\*mfnc.Hy-mfnc.Izz\*mfnc.Hz | A/m | Magnetization, z component | Domain 10 |
| mfnc.normM | sqrt(realdot(mfnc.Mx,mfnc.Mx)+realdot(mfnc.My,mfnc.My)+realdot(mfnc.Mz,mfnc.Mz)) | A/m | Magnetization norm | Domain 10 |
| mfnc.Ixx | 1 | 1 | Spatial identity matrix, xx component | Domain 10 |
| mfnc.Iyx | 0 | 1 | Spatial identity matrix, yx component | Domain 10 |
| mfnc.Izx | 0 | 1 | Spatial identity matrix, zx component | Domain 10 |
| mfnc.Ixy | 0 | 1 | Spatial identity matrix, xy component | Domain 10 |
| mfnc.Iyy | 1 | 1 | Spatial identity matrix, yy component | Domain 10 |
| mfnc.Izy | 0 | 1 | Spatial identity matrix, zy component | Domain 10 |
| mfnc.Ixz | 0 | 1 | Spatial identity matrix, xz component | Domain 10 |
| mfnc.Iyz | 0 | 1 | Spatial identity matrix, yz component | Domain 10 |
| mfnc.Izz | 1 | 1 | Spatial identity matrix, zz component | Domain 10 |
| mfnc.chimxx | -1+mfnc.murxx | 1 | Magnetic susceptibility, xx component | Domain 10 |
| mfnc.chimyx | mfnc.muryx | 1 | Magnetic susceptibility, yx component | Domain 10 |
| mfnc.chimzx | mfnc.murzx | 1 | Magnetic susceptibility, zx component | Domain 10 |
| mfnc.chimxy | mfnc.murxy | 1 | Magnetic susceptibility, xy component | Domain 10 |
| mfnc.chimyy | -1+mfnc.muryy | 1 | Magnetic susceptibility, yy component | Domain 10 |
| mfnc.chimzy | mfnc.murzy | 1 | Magnetic susceptibility, zy component | Domain 10 |
| mfnc.chimxz | mfnc.murxz | 1 | Magnetic susceptibility, xz component | Domain 10 |
| mfnc.chimyz | mfnc.muryz | 1 | Magnetic susceptibility, yz component | Domain 10 |
| mfnc.chimzz | -1+mfnc.murzz | 1 | Magnetic susceptibility, zz component | Domain 10 |
| mfnc.Brx | BC\*cos(pi\*(360+corr\_c)/180) | T | Remanent flux density, x component | Domain 10 |
| mfnc.Bry | BC\*sin(pi\*(360+corr\_c)/180) | T | Remanent flux density, y component | Domain 10 |
| mfnc.Brz | 0 | T | Remanent flux density, z component | Domain 10 |
| mfnc.normBr | sqrt(realdot(mfnc.Brx,mfnc.Brx)+realdot(mfnc.Bry,mfnc.Bry)+realdot(mfnc.Brz,mfnc.Brz)) | T | Remanent flux density norm | Domain 10 |
| mfnc.unTx | 0 | Pa | Maxwell upward surface stress tensor, x component | Boundaries 80–89 |
| mfnc.unTy | 0 | Pa | Maxwell upward surface stress tensor, y component | Boundaries 80–89 |
| mfnc.unTz | 0 | Pa | Maxwell upward surface stress tensor, z component | Boundaries 80–89 |
| mfnc.dnTx | mfnc.dnTmx | Pa | Maxwell downward surface stress tensor, x component | Boundaries 80–89 |
| mfnc.dnTy | mfnc.dnTmy | Pa | Maxwell downward surface stress tensor, y component | Boundaries 80–89 |
| mfnc.dnTz | mfnc.dnTmz | Pa | Maxwell downward surface stress tensor, z component | Boundaries 80–89 |
| mfnc.unx | unx |  | Normal vector up direction, x component | Boundaries 80–89 |
| mfnc.uny | uny |  | Normal vector up direction, y component | Boundaries 80–89 |
| mfnc.unz | unz |  | Normal vector up direction, z component | Boundaries 80–89 |
| mfnc.dnx | dnx |  | Normal vector down direction, x component | Boundaries 80–89 |
| mfnc.dny | dny |  | Normal vector down direction, y component | Boundaries 80–89 |
| mfnc.dnz | dnz |  | Normal vector down direction, z component | Boundaries 80–89 |
| mfnc.W | mfnc.Wm | J/m^3 | Energy density | Domain 10 |
| mfnc.dWm | mfnc.Wm | J/m^3 | Integrand for total magnetic energy | Domain 10 |
| mfnc.Wm | 0.5\*mu0\_const\*((mfnc.murxx\*mfnc.Hx+mfnc.murxy\*mfnc.Hy+mfnc.murxz\*mfnc.Hz)\*mfnc.Hx+(mfnc.muryx\*mfnc.Hx+mfnc.muryy\*mfnc.Hy+mfnc.muryz\*mfnc.Hz)\*mfnc.Hy+(mfnc.murzx\*mfnc.Hx+mfnc.murzy\*mfnc.Hy+mfnc.murzz\*mfnc.Hz)\*mfnc.Hz) | J/m^3 | Magnetic energy density | Domain 10 |

##### Shape Functions

| Name | Shape function | Unit | Description | Shape frame | Selection |
| --- | --- | --- | --- | --- | --- |
| Vm | Lagrange (Quadratic) | A | Magnetic scalar potential | Material | Domain 10 |

##### Weak Expressions

| Weak expression | Integration frame | Selection |
| --- | --- | --- |
| mfnc.d\*(-mfnc.Bx\*test(Vmx)-mfnc.By\*test(Vmy)-mfnc.Bz\*test(Vmz)) | Material | Domain 10 |

#### 2.4.20. ArrayCMagnet9

ArrayCMagnet9

Selection

| Geometric entity level | Domain |
| Selection | Domain 13 |

Equations

Settings

| Description | Value |
| Constitutive relation | Remanent flux density |
| Remanent flux density, x component | BC\*cos(2\*pi/360\*(8\*60 + corr\_c)) |
| Remanent flux density, y component | BC\*sin(2\*pi/360\*(8\*60 + corr\_c)) |
| Remanent flux density, z component | 0 |
| Relative permeability | From material |
| Relative permeability | {{1, 0, 0}, {0, 1, 0}, {0, 0, 1}} |

Properties from material

| Property | Material | Property group |
| Relative permeability | Soft Iron (without losses) | Basic |

##### Variables

| Name | Expression | Unit | Description | Selection |
| --- | --- | --- | --- | --- |
| mfnc.dnTmx | -0.5\*mfnc.unx\*(real(down(mfnc.Bx))\*real(down(mfnc.Hx))+real(down(mfnc.By))\*real(down(mfnc.Hy))+real(down(mfnc.Bz))\*real(down(mfnc.Hz)))+real(down(mfnc.Bx))\*(real(down(mfnc.Hx))\*mfnc.unx+real(down(mfnc.Hy))\*mfnc.uny+real(down(mfnc.Hz))\*mfnc.unz) | Pa | Maxwell downward magnetic surface stress tensor, x component | Boundaries 116–119, 124, 126, 128, 130, 132, 134 |
| mfnc.dnTmy | -0.5\*mfnc.uny\*(real(down(mfnc.Bx))\*real(down(mfnc.Hx))+real(down(mfnc.By))\*real(down(mfnc.Hy))+real(down(mfnc.Bz))\*real(down(mfnc.Hz)))+real(down(mfnc.By))\*(real(down(mfnc.Hx))\*mfnc.unx+real(down(mfnc.Hy))\*mfnc.uny+real(down(mfnc.Hz))\*mfnc.unz) | Pa | Maxwell downward magnetic surface stress tensor, y component | Boundaries 116–119, 124, 126, 128, 130, 132, 134 |
| mfnc.dnTmz | -0.5\*mfnc.unz\*(real(down(mfnc.Bx))\*real(down(mfnc.Hx))+real(down(mfnc.By))\*real(down(mfnc.Hy))+real(down(mfnc.Bz))\*real(down(mfnc.Hz)))+real(down(mfnc.Bz))\*(real(down(mfnc.Hx))\*mfnc.unx+real(down(mfnc.Hy))\*mfnc.uny+real(down(mfnc.Hz))\*mfnc.unz) | Pa | Maxwell downward magnetic surface stress tensor, z component | Boundaries 116–119, 124, 126, 128, 130, 132, 134 |
| mfnc.Hx | -Vmx | A/m | Magnetic field, x component | Domain 13 |
| mfnc.Hy | -Vmy | A/m | Magnetic field, y component | Domain 13 |
| mfnc.Hz | -Vmz | A/m | Magnetic field, z component | Domain 13 |
| mfnc.tHx | -VmTx | A/m | Tangential magnetic field, x component | Boundaries 116–119, 124, 126, 128, 130, 132, 134 |
| mfnc.tHy | -VmTy | A/m | Tangential magnetic field, y component | Boundaries 116–119, 124, 126, 128, 130, 132, 134 |
| mfnc.tHz | -VmTz | A/m | Tangential magnetic field, z component | Boundaries 116–119, 124, 126, 128, 130, 132, 134 |
| mfnc.normH | sqrt(realdot(mfnc.Hx,mfnc.Hx)+realdot(mfnc.Hy,mfnc.Hy)+realdot(mfnc.Hz,mfnc.Hz)) | A/m | Magnetic field norm | Domain 13 |
| mfnc.murxx | model.input.mur11 | 1 | Relative permeability, xx component | Domain 13 |
| mfnc.muryx | model.input.mur21 | 1 | Relative permeability, yx component | Domain 13 |
| mfnc.murzx | model.input.mur31 | 1 | Relative permeability, zx component | Domain 13 |
| mfnc.murxy | model.input.mur12 | 1 | Relative permeability, xy component | Domain 13 |
| mfnc.muryy | model.input.mur22 | 1 | Relative permeability, yy component | Domain 13 |
| mfnc.murzy | model.input.mur32 | 1 | Relative permeability, zy component | Domain 13 |
| mfnc.murxz | model.input.mur13 | 1 | Relative permeability, xz component | Domain 13 |
| mfnc.muryz | model.input.mur23 | 1 | Relative permeability, yz component | Domain 13 |
| mfnc.murzz | model.input.mur33 | 1 | Relative permeability, zz component | Domain 13 |
| mfnc.Bx | mu0\_const\*mfnc.Ixx\*mfnc.Hx+mu0\_const\*mfnc.Ixy\*mfnc.Hy+mu0\_const\*mfnc.Ixz\*mfnc.Hz+mu0\_const\*mfnc.chimxx\*mfnc.Hx+mu0\_const\*mfnc.chimxy\*mfnc.Hy+mu0\_const\*mfnc.chimxz\*mfnc.Hz+mfnc.Brx | T | Magnetic flux density, x component | Domain 13 |
| mfnc.By | mu0\_const\*mfnc.Iyx\*mfnc.Hx+mu0\_const\*mfnc.Iyy\*mfnc.Hy+mu0\_const\*mfnc.Iyz\*mfnc.Hz+mu0\_const\*mfnc.chimyx\*mfnc.Hx+mu0\_const\*mfnc.chimyy\*mfnc.Hy+mu0\_const\*mfnc.chimyz\*mfnc.Hz+mfnc.Bry | T | Magnetic flux density, y component | Domain 13 |
| mfnc.Bz | mu0\_const\*mfnc.Izx\*mfnc.Hx+mu0\_const\*mfnc.Izy\*mfnc.Hy+mu0\_const\*mfnc.Izz\*mfnc.Hz+mu0\_const\*mfnc.chimzx\*mfnc.Hx+mu0\_const\*mfnc.chimzy\*mfnc.Hy+mu0\_const\*mfnc.chimzz\*mfnc.Hz+mfnc.Brz | T | Magnetic flux density, z component | Domain 13 |
| mfnc.normB | sqrt(realdot(mfnc.Bx,mfnc.Bx)+realdot(mfnc.By,mfnc.By)+realdot(mfnc.Bz,mfnc.Bz)) | T | Magnetic flux density norm | Domain 13 |
| mfnc.Mx | mfnc.Bx/mu0\_const-mfnc.Ixx\*mfnc.Hx-mfnc.Ixy\*mfnc.Hy-mfnc.Ixz\*mfnc.Hz | A/m | Magnetization, x component | Domain 13 |
| mfnc.My | mfnc.By/mu0\_const-mfnc.Iyx\*mfnc.Hx-mfnc.Iyy\*mfnc.Hy-mfnc.Iyz\*mfnc.Hz | A/m | Magnetization, y component | Domain 13 |
| mfnc.Mz | mfnc.Bz/mu0\_const-mfnc.Izx\*mfnc.Hx-mfnc.Izy\*mfnc.Hy-mfnc.Izz\*mfnc.Hz | A/m | Magnetization, z component | Domain 13 |
| mfnc.normM | sqrt(realdot(mfnc.Mx,mfnc.Mx)+realdot(mfnc.My,mfnc.My)+realdot(mfnc.Mz,mfnc.Mz)) | A/m | Magnetization norm | Domain 13 |
| mfnc.Ixx | 1 | 1 | Spatial identity matrix, xx component | Domain 13 |
| mfnc.Iyx | 0 | 1 | Spatial identity matrix, yx component | Domain 13 |
| mfnc.Izx | 0 | 1 | Spatial identity matrix, zx component | Domain 13 |
| mfnc.Ixy | 0 | 1 | Spatial identity matrix, xy component | Domain 13 |
| mfnc.Iyy | 1 | 1 | Spatial identity matrix, yy component | Domain 13 |
| mfnc.Izy | 0 | 1 | Spatial identity matrix, zy component | Domain 13 |
| mfnc.Ixz | 0 | 1 | Spatial identity matrix, xz component | Domain 13 |
| mfnc.Iyz | 0 | 1 | Spatial identity matrix, yz component | Domain 13 |
| mfnc.Izz | 1 | 1 | Spatial identity matrix, zz component | Domain 13 |
| mfnc.chimxx | -1+mfnc.murxx | 1 | Magnetic susceptibility, xx component | Domain 13 |
| mfnc.chimyx | mfnc.muryx | 1 | Magnetic susceptibility, yx component | Domain 13 |
| mfnc.chimzx | mfnc.murzx | 1 | Magnetic susceptibility, zx component | Domain 13 |
| mfnc.chimxy | mfnc.murxy | 1 | Magnetic susceptibility, xy component | Domain 13 |
| mfnc.chimyy | -1+mfnc.muryy | 1 | Magnetic susceptibility, yy component | Domain 13 |
| mfnc.chimzy | mfnc.murzy | 1 | Magnetic susceptibility, zy component | Domain 13 |
| mfnc.chimxz | mfnc.murxz | 1 | Magnetic susceptibility, xz component | Domain 13 |
| mfnc.chimyz | mfnc.muryz | 1 | Magnetic susceptibility, yz component | Domain 13 |
| mfnc.chimzz | -1+mfnc.murzz | 1 | Magnetic susceptibility, zz component | Domain 13 |
| mfnc.Brx | BC\*cos(pi\*(480+corr\_c)/180) | T | Remanent flux density, x component | Domain 13 |
| mfnc.Bry | BC\*sin(pi\*(480+corr\_c)/180) | T | Remanent flux density, y component | Domain 13 |
| mfnc.Brz | 0 | T | Remanent flux density, z component | Domain 13 |
| mfnc.normBr | sqrt(realdot(mfnc.Brx,mfnc.Brx)+realdot(mfnc.Bry,mfnc.Bry)+realdot(mfnc.Brz,mfnc.Brz)) | T | Remanent flux density norm | Domain 13 |
| mfnc.unTx | 0 | Pa | Maxwell upward surface stress tensor, x component | Boundaries 116–119, 124, 126, 128, 130, 132, 134 |
| mfnc.unTy | 0 | Pa | Maxwell upward surface stress tensor, y component | Boundaries 116–119, 124, 126, 128, 130, 132, 134 |
| mfnc.unTz | 0 | Pa | Maxwell upward surface stress tensor, z component | Boundaries 116–119, 124, 126, 128, 130, 132, 134 |
| mfnc.dnTx | mfnc.dnTmx | Pa | Maxwell downward surface stress tensor, x component | Boundaries 116–119, 124, 126, 128, 130, 132, 134 |
| mfnc.dnTy | mfnc.dnTmy | Pa | Maxwell downward surface stress tensor, y component | Boundaries 116–119, 124, 126, 128, 130, 132, 134 |
| mfnc.dnTz | mfnc.dnTmz | Pa | Maxwell downward surface stress tensor, z component | Boundaries 116–119, 124, 126, 128, 130, 132, 134 |
| mfnc.unx | unx |  | Normal vector up direction, x component | Boundaries 116–119, 124, 126, 128, 130, 132, 134 |
| mfnc.uny | uny |  | Normal vector up direction, y component | Boundaries 116–119, 124, 126, 128, 130, 132, 134 |
| mfnc.unz | unz |  | Normal vector up direction, z component | Boundaries 116–119, 124, 126, 128, 130, 132, 134 |
| mfnc.dnx | dnx |  | Normal vector down direction, x component | Boundaries 116–119, 124, 126, 128, 130, 132, 134 |
| mfnc.dny | dny |  | Normal vector down direction, y component | Boundaries 116–119, 124, 126, 128, 130, 132, 134 |
| mfnc.dnz | dnz |  | Normal vector down direction, z component | Boundaries 116–119, 124, 126, 128, 130, 132, 134 |
| mfnc.W | mfnc.Wm | J/m^3 | Energy density | Domain 13 |
| mfnc.dWm | mfnc.Wm | J/m^3 | Integrand for total magnetic energy | Domain 13 |
| mfnc.Wm | 0.5\*mu0\_const\*((mfnc.murxx\*mfnc.Hx+mfnc.murxy\*mfnc.Hy+mfnc.murxz\*mfnc.Hz)\*mfnc.Hx+(mfnc.muryx\*mfnc.Hx+mfnc.muryy\*mfnc.Hy+mfnc.muryz\*mfnc.Hz)\*mfnc.Hy+(mfnc.murzx\*mfnc.Hx+mfnc.murzy\*mfnc.Hy+mfnc.murzz\*mfnc.Hz)\*mfnc.Hz) | J/m^3 | Magnetic energy density | Domain 13 |

##### Shape Functions

| Name | Shape function | Unit | Description | Shape frame | Selection |
| --- | --- | --- | --- | --- | --- |
| Vm | Lagrange (Quadratic) | A | Magnetic scalar potential | Material | Domain 13 |

##### Weak Expressions

| Weak expression | Integration frame | Selection |
| --- | --- | --- |
| mfnc.d\*(-mfnc.Bx\*test(Vmx)-mfnc.By\*test(Vmy)-mfnc.Bz\*test(Vmz)) | Material | Domain 13 |

#### 2.4.21. ArrayCMagnet11

ArrayCMagnet11

Selection

| Geometric entity level | Domain |
| Selection | Domain 20 |

Equations

Settings

| Description | Value |
| Constitutive relation | Remanent flux density |
| Remanent flux density, x component | BC\*cos(2\*pi/360\*(10\*60 + corr\_c)) |
| Remanent flux density, y component | BC\*sin(2\*pi/360\*(10\*60 + corr\_c)) |
| Remanent flux density, z component | 0 |
| Relative permeability | From material |
| Relative permeability | {{1, 0, 0}, {0, 1, 0}, {0, 0, 1}} |

Properties from material

| Property | Material | Property group |
| Relative permeability | Soft Iron (without losses) | Basic |

##### Variables

| Name | Expression | Unit | Description | Selection |
| --- | --- | --- | --- | --- |
| mfnc.dnTmx | -0.5\*mfnc.unx\*(real(down(mfnc.Bx))\*real(down(mfnc.Hx))+real(down(mfnc.By))\*real(down(mfnc.Hy))+real(down(mfnc.Bz))\*real(down(mfnc.Hz)))+real(down(mfnc.Bx))\*(real(down(mfnc.Hx))\*mfnc.unx+real(down(mfnc.Hy))\*mfnc.uny+real(down(mfnc.Hz))\*mfnc.unz) | Pa | Maxwell downward magnetic surface stress tensor, x component | Boundaries 182–185, 190, 192, 194, 196, 198, 200 |
| mfnc.dnTmy | -0.5\*mfnc.uny\*(real(down(mfnc.Bx))\*real(down(mfnc.Hx))+real(down(mfnc.By))\*real(down(mfnc.Hy))+real(down(mfnc.Bz))\*real(down(mfnc.Hz)))+real(down(mfnc.By))\*(real(down(mfnc.Hx))\*mfnc.unx+real(down(mfnc.Hy))\*mfnc.uny+real(down(mfnc.Hz))\*mfnc.unz) | Pa | Maxwell downward magnetic surface stress tensor, y component | Boundaries 182–185, 190, 192, 194, 196, 198, 200 |
| mfnc.dnTmz | -0.5\*mfnc.unz\*(real(down(mfnc.Bx))\*real(down(mfnc.Hx))+real(down(mfnc.By))\*real(down(mfnc.Hy))+real(down(mfnc.Bz))\*real(down(mfnc.Hz)))+real(down(mfnc.Bz))\*(real(down(mfnc.Hx))\*mfnc.unx+real(down(mfnc.Hy))\*mfnc.uny+real(down(mfnc.Hz))\*mfnc.unz) | Pa | Maxwell downward magnetic surface stress tensor, z component | Boundaries 182–185, 190, 192, 194, 196, 198, 200 |
| mfnc.Hx | -Vmx | A/m | Magnetic field, x component | Domain 20 |
| mfnc.Hy | -Vmy | A/m | Magnetic field, y component | Domain 20 |
| mfnc.Hz | -Vmz | A/m | Magnetic field, z component | Domain 20 |
| mfnc.tHx | -VmTx | A/m | Tangential magnetic field, x component | Boundaries 182–185, 190, 192, 194, 196, 198, 200 |
| mfnc.tHy | -VmTy | A/m | Tangential magnetic field, y component | Boundaries 182–185, 190, 192, 194, 196, 198, 200 |
| mfnc.tHz | -VmTz | A/m | Tangential magnetic field, z component | Boundaries 182–185, 190, 192, 194, 196, 198, 200 |
| mfnc.normH | sqrt(realdot(mfnc.Hx,mfnc.Hx)+realdot(mfnc.Hy,mfnc.Hy)+realdot(mfnc.Hz,mfnc.Hz)) | A/m | Magnetic field norm | Domain 20 |
| mfnc.murxx | model.input.mur11 | 1 | Relative permeability, xx component | Domain 20 |
| mfnc.muryx | model.input.mur21 | 1 | Relative permeability, yx component | Domain 20 |
| mfnc.murzx | model.input.mur31 | 1 | Relative permeability, zx component | Domain 20 |
| mfnc.murxy | model.input.mur12 | 1 | Relative permeability, xy component | Domain 20 |
| mfnc.muryy | model.input.mur22 | 1 | Relative permeability, yy component | Domain 20 |
| mfnc.murzy | model.input.mur32 | 1 | Relative permeability, zy component | Domain 20 |
| mfnc.murxz | model.input.mur13 | 1 | Relative permeability, xz component | Domain 20 |
| mfnc.muryz | model.input.mur23 | 1 | Relative permeability, yz component | Domain 20 |
| mfnc.murzz | model.input.mur33 | 1 | Relative permeability, zz component | Domain 20 |
| mfnc.Bx | mu0\_const\*mfnc.Ixx\*mfnc.Hx+mu0\_const\*mfnc.Ixy\*mfnc.Hy+mu0\_const\*mfnc.Ixz\*mfnc.Hz+mu0\_const\*mfnc.chimxx\*mfnc.Hx+mu0\_const\*mfnc.chimxy\*mfnc.Hy+mu0\_const\*mfnc.chimxz\*mfnc.Hz+mfnc.Brx | T | Magnetic flux density, x component | Domain 20 |
| mfnc.By | mu0\_const\*mfnc.Iyx\*mfnc.Hx+mu0\_const\*mfnc.Iyy\*mfnc.Hy+mu0\_const\*mfnc.Iyz\*mfnc.Hz+mu0\_const\*mfnc.chimyx\*mfnc.Hx+mu0\_const\*mfnc.chimyy\*mfnc.Hy+mu0\_const\*mfnc.chimyz\*mfnc.Hz+mfnc.Bry | T | Magnetic flux density, y component | Domain 20 |
| mfnc.Bz | mu0\_const\*mfnc.Izx\*mfnc.Hx+mu0\_const\*mfnc.Izy\*mfnc.Hy+mu0\_const\*mfnc.Izz\*mfnc.Hz+mu0\_const\*mfnc.chimzx\*mfnc.Hx+mu0\_const\*mfnc.chimzy\*mfnc.Hy+mu0\_const\*mfnc.chimzz\*mfnc.Hz+mfnc.Brz | T | Magnetic flux density, z component | Domain 20 |
| mfnc.normB | sqrt(realdot(mfnc.Bx,mfnc.Bx)+realdot(mfnc.By,mfnc.By)+realdot(mfnc.Bz,mfnc.Bz)) | T | Magnetic flux density norm | Domain 20 |
| mfnc.Mx | mfnc.Bx/mu0\_const-mfnc.Ixx\*mfnc.Hx-mfnc.Ixy\*mfnc.Hy-mfnc.Ixz\*mfnc.Hz | A/m | Magnetization, x component | Domain 20 |
| mfnc.My | mfnc.By/mu0\_const-mfnc.Iyx\*mfnc.Hx-mfnc.Iyy\*mfnc.Hy-mfnc.Iyz\*mfnc.Hz | A/m | Magnetization, y component | Domain 20 |
| mfnc.Mz | mfnc.Bz/mu0\_const-mfnc.Izx\*mfnc.Hx-mfnc.Izy\*mfnc.Hy-mfnc.Izz\*mfnc.Hz | A/m | Magnetization, z component | Domain 20 |
| mfnc.normM | sqrt(realdot(mfnc.Mx,mfnc.Mx)+realdot(mfnc.My,mfnc.My)+realdot(mfnc.Mz,mfnc.Mz)) | A/m | Magnetization norm | Domain 20 |
| mfnc.Ixx | 1 | 1 | Spatial identity matrix, xx component | Domain 20 |
| mfnc.Iyx | 0 | 1 | Spatial identity matrix, yx component | Domain 20 |
| mfnc.Izx | 0 | 1 | Spatial identity matrix, zx component | Domain 20 |
| mfnc.Ixy | 0 | 1 | Spatial identity matrix, xy component | Domain 20 |
| mfnc.Iyy | 1 | 1 | Spatial identity matrix, yy component | Domain 20 |
| mfnc.Izy | 0 | 1 | Spatial identity matrix, zy component | Domain 20 |
| mfnc.Ixz | 0 | 1 | Spatial identity matrix, xz component | Domain 20 |
| mfnc.Iyz | 0 | 1 | Spatial identity matrix, yz component | Domain 20 |
| mfnc.Izz | 1 | 1 | Spatial identity matrix, zz component | Domain 20 |
| mfnc.chimxx | -1+mfnc.murxx | 1 | Magnetic susceptibility, xx component | Domain 20 |
| mfnc.chimyx | mfnc.muryx | 1 | Magnetic susceptibility, yx component | Domain 20 |
| mfnc.chimzx | mfnc.murzx | 1 | Magnetic susceptibility, zx component | Domain 20 |
| mfnc.chimxy | mfnc.murxy | 1 | Magnetic susceptibility, xy component | Domain 20 |
| mfnc.chimyy | -1+mfnc.muryy | 1 | Magnetic susceptibility, yy component | Domain 20 |
| mfnc.chimzy | mfnc.murzy | 1 | Magnetic susceptibility, zy component | Domain 20 |
| mfnc.chimxz | mfnc.murxz | 1 | Magnetic susceptibility, xz component | Domain 20 |
| mfnc.chimyz | mfnc.muryz | 1 | Magnetic susceptibility, yz component | Domain 20 |
| mfnc.chimzz | -1+mfnc.murzz | 1 | Magnetic susceptibility, zz component | Domain 20 |
| mfnc.Brx | BC\*cos(pi\*(600+corr\_c)/180) | T | Remanent flux density, x component | Domain 20 |
| mfnc.Bry | BC\*sin(pi\*(600+corr\_c)/180) | T | Remanent flux density, y component | Domain 20 |
| mfnc.Brz | 0 | T | Remanent flux density, z component | Domain 20 |
| mfnc.normBr | sqrt(realdot(mfnc.Brx,mfnc.Brx)+realdot(mfnc.Bry,mfnc.Bry)+realdot(mfnc.Brz,mfnc.Brz)) | T | Remanent flux density norm | Domain 20 |
| mfnc.unTx | 0 | Pa | Maxwell upward surface stress tensor, x component | Boundaries 182–185, 190, 192, 194, 196, 198, 200 |
| mfnc.unTy | 0 | Pa | Maxwell upward surface stress tensor, y component | Boundaries 182–185, 190, 192, 194, 196, 198, 200 |
| mfnc.unTz | 0 | Pa | Maxwell upward surface stress tensor, z component | Boundaries 182–185, 190, 192, 194, 196, 198, 200 |
| mfnc.dnTx | mfnc.dnTmx | Pa | Maxwell downward surface stress tensor, x component | Boundaries 182–185, 190, 192, 194, 196, 198, 200 |
| mfnc.dnTy | mfnc.dnTmy | Pa | Maxwell downward surface stress tensor, y component | Boundaries 182–185, 190, 192, 194, 196, 198, 200 |
| mfnc.dnTz | mfnc.dnTmz | Pa | Maxwell downward surface stress tensor, z component | Boundaries 182–185, 190, 192, 194, 196, 198, 200 |
| mfnc.unx | unx |  | Normal vector up direction, x component | Boundaries 182–185, 190, 192, 194, 196, 198, 200 |
| mfnc.uny | uny |  | Normal vector up direction, y component | Boundaries 182–185, 190, 192, 194, 196, 198, 200 |
| mfnc.unz | unz |  | Normal vector up direction, z component | Boundaries 182–185, 190, 192, 194, 196, 198, 200 |
| mfnc.dnx | dnx |  | Normal vector down direction, x component | Boundaries 182–185, 190, 192, 194, 196, 198, 200 |
| mfnc.dny | dny |  | Normal vector down direction, y component | Boundaries 182–185, 190, 192, 194, 196, 198, 200 |
| mfnc.dnz | dnz |  | Normal vector down direction, z component | Boundaries 182–185, 190, 192, 194, 196, 198, 200 |
| mfnc.W | mfnc.Wm | J/m^3 | Energy density | Domain 20 |
| mfnc.dWm | mfnc.Wm | J/m^3 | Integrand for total magnetic energy | Domain 20 |
| mfnc.Wm | 0.5\*mu0\_const\*((mfnc.murxx\*mfnc.Hx+mfnc.murxy\*mfnc.Hy+mfnc.murxz\*mfnc.Hz)\*mfnc.Hx+(mfnc.muryx\*mfnc.Hx+mfnc.muryy\*mfnc.Hy+mfnc.muryz\*mfnc.Hz)\*mfnc.Hy+(mfnc.murzx\*mfnc.Hx+mfnc.murzy\*mfnc.Hy+mfnc.murzz\*mfnc.Hz)\*mfnc.Hz) | J/m^3 | Magnetic energy density | Domain 20 |

##### Shape Functions

| Name | Shape function | Unit | Description | Shape frame | Selection |
| --- | --- | --- | --- | --- | --- |
| Vm | Lagrange (Quadratic) | A | Magnetic scalar potential | Material | Domain 20 |

##### Weak Expressions

| Weak expression | Integration frame | Selection |
| --- | --- | --- |
| mfnc.d\*(-mfnc.Bx\*test(Vmx)-mfnc.By\*test(Vmy)-mfnc.Bz\*test(Vmz)) | Material | Domain 20 |

#### 2.4.22. ArrayAMagnet1

ArrayAMagnet1

Selection

| Geometric entity level | Domain |
| Selection | Domain 32 |

Equations

Settings

| Description | Value |
| Constitutive relation | Remanent flux density |
| Remanent flux density, x component | BA\*cos(2\*pi\*(0\*60)/360) |
| Remanent flux density, y component | BA\*sin(2\*pi\*(0\*60)/360) |
| Remanent flux density, z component | 0 |
| Relative permeability | From material |
| Relative permeability | {{1, 0, 0}, {0, 1, 0}, {0, 0, 1}} |

Properties from material

| Property | Material | Property group |
| Relative permeability | Soft Iron (without losses) | Basic |

##### Variables

| Name | Expression | Unit | Description | Selection |
| --- | --- | --- | --- | --- |
| mfnc.dnTmx | -0.5\*mfnc.unx\*(real(down(mfnc.Bx))\*real(down(mfnc.Hx))+real(down(mfnc.By))\*real(down(mfnc.Hy))+real(down(mfnc.Bz))\*real(down(mfnc.Hz)))+real(down(mfnc.Bx))\*(real(down(mfnc.Hx))\*mfnc.unx+real(down(mfnc.Hy))\*mfnc.uny+real(down(mfnc.Hz))\*mfnc.unz) | Pa | Maxwell downward magnetic surface stress tensor, x component | Boundaries 300–306, 309–311 |
| mfnc.dnTmy | -0.5\*mfnc.uny\*(real(down(mfnc.Bx))\*real(down(mfnc.Hx))+real(down(mfnc.By))\*real(down(mfnc.Hy))+real(down(mfnc.Bz))\*real(down(mfnc.Hz)))+real(down(mfnc.By))\*(real(down(mfnc.Hx))\*mfnc.unx+real(down(mfnc.Hy))\*mfnc.uny+real(down(mfnc.Hz))\*mfnc.unz) | Pa | Maxwell downward magnetic surface stress tensor, y component | Boundaries 300–306, 309–311 |
| mfnc.dnTmz | -0.5\*mfnc.unz\*(real(down(mfnc.Bx))\*real(down(mfnc.Hx))+real(down(mfnc.By))\*real(down(mfnc.Hy))+real(down(mfnc.Bz))\*real(down(mfnc.Hz)))+real(down(mfnc.Bz))\*(real(down(mfnc.Hx))\*mfnc.unx+real(down(mfnc.Hy))\*mfnc.uny+real(down(mfnc.Hz))\*mfnc.unz) | Pa | Maxwell downward magnetic surface stress tensor, z component | Boundaries 300–306, 309–311 |
| mfnc.Hx | -Vmx | A/m | Magnetic field, x component | Domain 32 |
| mfnc.Hy | -Vmy | A/m | Magnetic field, y component | Domain 32 |
| mfnc.Hz | -Vmz | A/m | Magnetic field, z component | Domain 32 |
| mfnc.tHx | -VmTx | A/m | Tangential magnetic field, x component | Boundaries 300–306, 309–311 |
| mfnc.tHy | -VmTy | A/m | Tangential magnetic field, y component | Boundaries 300–306, 309–311 |
| mfnc.tHz | -VmTz | A/m | Tangential magnetic field, z component | Boundaries 300–306, 309–311 |
| mfnc.normH | sqrt(realdot(mfnc.Hx,mfnc.Hx)+realdot(mfnc.Hy,mfnc.Hy)+realdot(mfnc.Hz,mfnc.Hz)) | A/m | Magnetic field norm | Domain 32 |
| mfnc.murxx | model.input.mur11 | 1 | Relative permeability, xx component | Domain 32 |
| mfnc.muryx | model.input.mur21 | 1 | Relative permeability, yx component | Domain 32 |
| mfnc.murzx | model.input.mur31 | 1 | Relative permeability, zx component | Domain 32 |
| mfnc.murxy | model.input.mur12 | 1 | Relative permeability, xy component | Domain 32 |
| mfnc.muryy | model.input.mur22 | 1 | Relative permeability, yy component | Domain 32 |
| mfnc.murzy | model.input.mur32 | 1 | Relative permeability, zy component | Domain 32 |
| mfnc.murxz | model.input.mur13 | 1 | Relative permeability, xz component | Domain 32 |
| mfnc.muryz | model.input.mur23 | 1 | Relative permeability, yz component | Domain 32 |
| mfnc.murzz | model.input.mur33 | 1 | Relative permeability, zz component | Domain 32 |
| mfnc.Bx | mu0\_const\*mfnc.Ixx\*mfnc.Hx+mu0\_const\*mfnc.Ixy\*mfnc.Hy+mu0\_const\*mfnc.Ixz\*mfnc.Hz+mu0\_const\*mfnc.chimxx\*mfnc.Hx+mu0\_const\*mfnc.chimxy\*mfnc.Hy+mu0\_const\*mfnc.chimxz\*mfnc.Hz+mfnc.Brx | T | Magnetic flux density, x component | Domain 32 |
| mfnc.By | mu0\_const\*mfnc.Iyx\*mfnc.Hx+mu0\_const\*mfnc.Iyy\*mfnc.Hy+mu0\_const\*mfnc.Iyz\*mfnc.Hz+mu0\_const\*mfnc.chimyx\*mfnc.Hx+mu0\_const\*mfnc.chimyy\*mfnc.Hy+mu0\_const\*mfnc.chimyz\*mfnc.Hz+mfnc.Bry | T | Magnetic flux density, y component | Domain 32 |
| mfnc.Bz | mu0\_const\*mfnc.Izx\*mfnc.Hx+mu0\_const\*mfnc.Izy\*mfnc.Hy+mu0\_const\*mfnc.Izz\*mfnc.Hz+mu0\_const\*mfnc.chimzx\*mfnc.Hx+mu0\_const\*mfnc.chimzy\*mfnc.Hy+mu0\_const\*mfnc.chimzz\*mfnc.Hz+mfnc.Brz | T | Magnetic flux density, z component | Domain 32 |
| mfnc.normB | sqrt(realdot(mfnc.Bx,mfnc.Bx)+realdot(mfnc.By,mfnc.By)+realdot(mfnc.Bz,mfnc.Bz)) | T | Magnetic flux density norm | Domain 32 |
| mfnc.Mx | mfnc.Bx/mu0\_const-mfnc.Ixx\*mfnc.Hx-mfnc.Ixy\*mfnc.Hy-mfnc.Ixz\*mfnc.Hz | A/m | Magnetization, x component | Domain 32 |
| mfnc.My | mfnc.By/mu0\_const-mfnc.Iyx\*mfnc.Hx-mfnc.Iyy\*mfnc.Hy-mfnc.Iyz\*mfnc.Hz | A/m | Magnetization, y component | Domain 32 |
| mfnc.Mz | mfnc.Bz/mu0\_const-mfnc.Izx\*mfnc.Hx-mfnc.Izy\*mfnc.Hy-mfnc.Izz\*mfnc.Hz | A/m | Magnetization, z component | Domain 32 |
| mfnc.normM | sqrt(realdot(mfnc.Mx,mfnc.Mx)+realdot(mfnc.My,mfnc.My)+realdot(mfnc.Mz,mfnc.Mz)) | A/m | Magnetization norm | Domain 32 |
| mfnc.Ixx | 1 | 1 | Spatial identity matrix, xx component | Domain 32 |
| mfnc.Iyx | 0 | 1 | Spatial identity matrix, yx component | Domain 32 |
| mfnc.Izx | 0 | 1 | Spatial identity matrix, zx component | Domain 32 |
| mfnc.Ixy | 0 | 1 | Spatial identity matrix, xy component | Domain 32 |
| mfnc.Iyy | 1 | 1 | Spatial identity matrix, yy component | Domain 32 |
| mfnc.Izy | 0 | 1 | Spatial identity matrix, zy component | Domain 32 |
| mfnc.Ixz | 0 | 1 | Spatial identity matrix, xz component | Domain 32 |
| mfnc.Iyz | 0 | 1 | Spatial identity matrix, yz component | Domain 32 |
| mfnc.Izz | 1 | 1 | Spatial identity matrix, zz component | Domain 32 |
| mfnc.chimxx | -1+mfnc.murxx | 1 | Magnetic susceptibility, xx component | Domain 32 |
| mfnc.chimyx | mfnc.muryx | 1 | Magnetic susceptibility, yx component | Domain 32 |
| mfnc.chimzx | mfnc.murzx | 1 | Magnetic susceptibility, zx component | Domain 32 |
| mfnc.chimxy | mfnc.murxy | 1 | Magnetic susceptibility, xy component | Domain 32 |
| mfnc.chimyy | -1+mfnc.muryy | 1 | Magnetic susceptibility, yy component | Domain 32 |
| mfnc.chimzy | mfnc.murzy | 1 | Magnetic susceptibility, zy component | Domain 32 |
| mfnc.chimxz | mfnc.murxz | 1 | Magnetic susceptibility, xz component | Domain 32 |
| mfnc.chimyz | mfnc.muryz | 1 | Magnetic susceptibility, yz component | Domain 32 |
| mfnc.chimzz | -1+mfnc.murzz | 1 | Magnetic susceptibility, zz component | Domain 32 |
| mfnc.Brx | BA | T | Remanent flux density, x component | Domain 32 |
| mfnc.Bry | 0 | T | Remanent flux density, y component | Domain 32 |
| mfnc.Brz | 0 | T | Remanent flux density, z component | Domain 32 |
| mfnc.normBr | sqrt(realdot(mfnc.Brx,mfnc.Brx)+realdot(mfnc.Bry,mfnc.Bry)+realdot(mfnc.Brz,mfnc.Brz)) | T | Remanent flux density norm | Domain 32 |
| mfnc.unTx | 0 | Pa | Maxwell upward surface stress tensor, x component | Boundaries 300–306, 309–311 |
| mfnc.unTy | 0 | Pa | Maxwell upward surface stress tensor, y component | Boundaries 300–306, 309–311 |
| mfnc.unTz | 0 | Pa | Maxwell upward surface stress tensor, z component | Boundaries 300–306, 309–311 |
| mfnc.dnTx | mfnc.dnTmx | Pa | Maxwell downward surface stress tensor, x component | Boundaries 300–306, 309–311 |
| mfnc.dnTy | mfnc.dnTmy | Pa | Maxwell downward surface stress tensor, y component | Boundaries 300–306, 309–311 |
| mfnc.dnTz | mfnc.dnTmz | Pa | Maxwell downward surface stress tensor, z component | Boundaries 300–306, 309–311 |
| mfnc.unx | unx |  | Normal vector up direction, x component | Boundaries 300–306, 309–311 |
| mfnc.uny | uny |  | Normal vector up direction, y component | Boundaries 300–306, 309–311 |
| mfnc.unz | unz |  | Normal vector up direction, z component | Boundaries 300–306, 309–311 |
| mfnc.dnx | dnx |  | Normal vector down direction, x component | Boundaries 300–306, 309–311 |
| mfnc.dny | dny |  | Normal vector down direction, y component | Boundaries 300–306, 309–311 |
| mfnc.dnz | dnz |  | Normal vector down direction, z component | Boundaries 300–306, 309–311 |
| mfnc.W | mfnc.Wm | J/m^3 | Energy density | Domain 32 |
| mfnc.dWm | mfnc.Wm | J/m^3 | Integrand for total magnetic energy | Domain 32 |
| mfnc.Wm | 0.5\*mu0\_const\*((mfnc.murxx\*mfnc.Hx+mfnc.murxy\*mfnc.Hy+mfnc.murxz\*mfnc.Hz)\*mfnc.Hx+(mfnc.muryx\*mfnc.Hx+mfnc.muryy\*mfnc.Hy+mfnc.muryz\*mfnc.Hz)\*mfnc.Hy+(mfnc.murzx\*mfnc.Hx+mfnc.murzy\*mfnc.Hy+mfnc.murzz\*mfnc.Hz)\*mfnc.Hz) | J/m^3 | Magnetic energy density | Domain 32 |

##### Shape Functions

| Name | Shape function | Unit | Description | Shape frame | Selection |
| --- | --- | --- | --- | --- | --- |
| Vm | Lagrange (Quadratic) | A | Magnetic scalar potential | Material | Domain 32 |

##### Weak Expressions

| Weak expression | Integration frame | Selection |
| --- | --- | --- |
| mfnc.d\*(-mfnc.Bx\*test(Vmx)-mfnc.By\*test(Vmy)-mfnc.Bz\*test(Vmz)) | Material | Domain 32 |

#### 2.4.23. ArrayAMagnet2

ArrayAMagnet2

Selection

| Geometric entity level | Domain |
| Selection | Domain 31 |

Equations

Settings

| Description | Value |
| Constitutive relation | Remanent flux density |
| Remanent flux density, x component | BA\*cos(2\*pi\*(1\*60)/360) |
| Remanent flux density, y component | BA\*sin(2\*pi\*(1\*60)/360) |
| Remanent flux density, z component | 0 |
| Relative permeability | From material |
| Relative permeability | {{1, 0, 0}, {0, 1, 0}, {0, 0, 1}} |

Properties from material

| Property | Material | Property group |
| Relative permeability | Soft Iron (without losses) | Basic |

##### Variables

| Name | Expression | Unit | Description | Selection |
| --- | --- | --- | --- | --- |
| mfnc.dnTmx | -0.5\*mfnc.unx\*(real(down(mfnc.Bx))\*real(down(mfnc.Hx))+real(down(mfnc.By))\*real(down(mfnc.Hy))+real(down(mfnc.Bz))\*real(down(mfnc.Hz)))+real(down(mfnc.Bx))\*(real(down(mfnc.Hx))\*mfnc.unx+real(down(mfnc.Hy))\*mfnc.uny+real(down(mfnc.Hz))\*mfnc.unz) | Pa | Maxwell downward magnetic surface stress tensor, x component | Boundaries 286–289, 291, 293, 295, 297, 299, 308 |
| mfnc.dnTmy | -0.5\*mfnc.uny\*(real(down(mfnc.Bx))\*real(down(mfnc.Hx))+real(down(mfnc.By))\*real(down(mfnc.Hy))+real(down(mfnc.Bz))\*real(down(mfnc.Hz)))+real(down(mfnc.By))\*(real(down(mfnc.Hx))\*mfnc.unx+real(down(mfnc.Hy))\*mfnc.uny+real(down(mfnc.Hz))\*mfnc.unz) | Pa | Maxwell downward magnetic surface stress tensor, y component | Boundaries 286–289, 291, 293, 295, 297, 299, 308 |
| mfnc.dnTmz | -0.5\*mfnc.unz\*(real(down(mfnc.Bx))\*real(down(mfnc.Hx))+real(down(mfnc.By))\*real(down(mfnc.Hy))+real(down(mfnc.Bz))\*real(down(mfnc.Hz)))+real(down(mfnc.Bz))\*(real(down(mfnc.Hx))\*mfnc.unx+real(down(mfnc.Hy))\*mfnc.uny+real(down(mfnc.Hz))\*mfnc.unz) | Pa | Maxwell downward magnetic surface stress tensor, z component | Boundaries 286–289, 291, 293, 295, 297, 299, 308 |
| mfnc.Hx | -Vmx | A/m | Magnetic field, x component | Domain 31 |
| mfnc.Hy | -Vmy | A/m | Magnetic field, y component | Domain 31 |
| mfnc.Hz | -Vmz | A/m | Magnetic field, z component | Domain 31 |
| mfnc.tHx | -VmTx | A/m | Tangential magnetic field, x component | Boundaries 286–289, 291, 293, 295, 297, 299, 308 |
| mfnc.tHy | -VmTy | A/m | Tangential magnetic field, y component | Boundaries 286–289, 291, 293, 295, 297, 299, 308 |
| mfnc.tHz | -VmTz | A/m | Tangential magnetic field, z component | Boundaries 286–289, 291, 293, 295, 297, 299, 308 |
| mfnc.normH | sqrt(realdot(mfnc.Hx,mfnc.Hx)+realdot(mfnc.Hy,mfnc.Hy)+realdot(mfnc.Hz,mfnc.Hz)) | A/m | Magnetic field norm | Domain 31 |
| mfnc.murxx | model.input.mur11 | 1 | Relative permeability, xx component | Domain 31 |
| mfnc.muryx | model.input.mur21 | 1 | Relative permeability, yx component | Domain 31 |
| mfnc.murzx | model.input.mur31 | 1 | Relative permeability, zx component | Domain 31 |
| mfnc.murxy | model.input.mur12 | 1 | Relative permeability, xy component | Domain 31 |
| mfnc.muryy | model.input.mur22 | 1 | Relative permeability, yy component | Domain 31 |
| mfnc.murzy | model.input.mur32 | 1 | Relative permeability, zy component | Domain 31 |
| mfnc.murxz | model.input.mur13 | 1 | Relative permeability, xz component | Domain 31 |
| mfnc.muryz | model.input.mur23 | 1 | Relative permeability, yz component | Domain 31 |
| mfnc.murzz | model.input.mur33 | 1 | Relative permeability, zz component | Domain 31 |
| mfnc.Bx | mu0\_const\*mfnc.Ixx\*mfnc.Hx+mu0\_const\*mfnc.Ixy\*mfnc.Hy+mu0\_const\*mfnc.Ixz\*mfnc.Hz+mu0\_const\*mfnc.chimxx\*mfnc.Hx+mu0\_const\*mfnc.chimxy\*mfnc.Hy+mu0\_const\*mfnc.chimxz\*mfnc.Hz+mfnc.Brx | T | Magnetic flux density, x component | Domain 31 |
| mfnc.By | mu0\_const\*mfnc.Iyx\*mfnc.Hx+mu0\_const\*mfnc.Iyy\*mfnc.Hy+mu0\_const\*mfnc.Iyz\*mfnc.Hz+mu0\_const\*mfnc.chimyx\*mfnc.Hx+mu0\_const\*mfnc.chimyy\*mfnc.Hy+mu0\_const\*mfnc.chimyz\*mfnc.Hz+mfnc.Bry | T | Magnetic flux density, y component | Domain 31 |
| mfnc.Bz | mu0\_const\*mfnc.Izx\*mfnc.Hx+mu0\_const\*mfnc.Izy\*mfnc.Hy+mu0\_const\*mfnc.Izz\*mfnc.Hz+mu0\_const\*mfnc.chimzx\*mfnc.Hx+mu0\_const\*mfnc.chimzy\*mfnc.Hy+mu0\_const\*mfnc.chimzz\*mfnc.Hz+mfnc.Brz | T | Magnetic flux density, z component | Domain 31 |
| mfnc.normB | sqrt(realdot(mfnc.Bx,mfnc.Bx)+realdot(mfnc.By,mfnc.By)+realdot(mfnc.Bz,mfnc.Bz)) | T | Magnetic flux density norm | Domain 31 |
| mfnc.Mx | mfnc.Bx/mu0\_const-mfnc.Ixx\*mfnc.Hx-mfnc.Ixy\*mfnc.Hy-mfnc.Ixz\*mfnc.Hz | A/m | Magnetization, x component | Domain 31 |
| mfnc.My | mfnc.By/mu0\_const-mfnc.Iyx\*mfnc.Hx-mfnc.Iyy\*mfnc.Hy-mfnc.Iyz\*mfnc.Hz | A/m | Magnetization, y component | Domain 31 |
| mfnc.Mz | mfnc.Bz/mu0\_const-mfnc.Izx\*mfnc.Hx-mfnc.Izy\*mfnc.Hy-mfnc.Izz\*mfnc.Hz | A/m | Magnetization, z component | Domain 31 |
| mfnc.normM | sqrt(realdot(mfnc.Mx,mfnc.Mx)+realdot(mfnc.My,mfnc.My)+realdot(mfnc.Mz,mfnc.Mz)) | A/m | Magnetization norm | Domain 31 |
| mfnc.Ixx | 1 | 1 | Spatial identity matrix, xx component | Domain 31 |
| mfnc.Iyx | 0 | 1 | Spatial identity matrix, yx component | Domain 31 |
| mfnc.Izx | 0 | 1 | Spatial identity matrix, zx component | Domain 31 |
| mfnc.Ixy | 0 | 1 | Spatial identity matrix, xy component | Domain 31 |
| mfnc.Iyy | 1 | 1 | Spatial identity matrix, yy component | Domain 31 |
| mfnc.Izy | 0 | 1 | Spatial identity matrix, zy component | Domain 31 |
| mfnc.Ixz | 0 | 1 | Spatial identity matrix, xz component | Domain 31 |
| mfnc.Iyz | 0 | 1 | Spatial identity matrix, yz component | Domain 31 |
| mfnc.Izz | 1 | 1 | Spatial identity matrix, zz component | Domain 31 |
| mfnc.chimxx | -1+mfnc.murxx | 1 | Magnetic susceptibility, xx component | Domain 31 |
| mfnc.chimyx | mfnc.muryx | 1 | Magnetic susceptibility, yx component | Domain 31 |
| mfnc.chimzx | mfnc.murzx | 1 | Magnetic susceptibility, zx component | Domain 31 |
| mfnc.chimxy | mfnc.murxy | 1 | Magnetic susceptibility, xy component | Domain 31 |
| mfnc.chimyy | -1+mfnc.muryy | 1 | Magnetic susceptibility, yy component | Domain 31 |
| mfnc.chimzy | mfnc.murzy | 1 | Magnetic susceptibility, zy component | Domain 31 |
| mfnc.chimxz | mfnc.murxz | 1 | Magnetic susceptibility, xz component | Domain 31 |
| mfnc.chimyz | mfnc.muryz | 1 | Magnetic susceptibility, yz component | Domain 31 |
| mfnc.chimzz | -1+mfnc.murzz | 1 | Magnetic susceptibility, zz component | Domain 31 |
| mfnc.Brx | BA\*cos(pi/3) | T | Remanent flux density, x component | Domain 31 |
| mfnc.Bry | BA\*sin(pi/3) | T | Remanent flux density, y component | Domain 31 |
| mfnc.Brz | 0 | T | Remanent flux density, z component | Domain 31 |
| mfnc.normBr | sqrt(realdot(mfnc.Brx,mfnc.Brx)+realdot(mfnc.Bry,mfnc.Bry)+realdot(mfnc.Brz,mfnc.Brz)) | T | Remanent flux density norm | Domain 31 |
| mfnc.unTx | 0 | Pa | Maxwell upward surface stress tensor, x component | Boundaries 286–289, 291, 293, 295, 297, 299, 308 |
| mfnc.unTy | 0 | Pa | Maxwell upward surface stress tensor, y component | Boundaries 286–289, 291, 293, 295, 297, 299, 308 |
| mfnc.unTz | 0 | Pa | Maxwell upward surface stress tensor, z component | Boundaries 286–289, 291, 293, 295, 297, 299, 308 |
| mfnc.dnTx | mfnc.dnTmx | Pa | Maxwell downward surface stress tensor, x component | Boundaries 286–289, 291, 293, 295, 297, 299, 308 |
| mfnc.dnTy | mfnc.dnTmy | Pa | Maxwell downward surface stress tensor, y component | Boundaries 286–289, 291, 293, 295, 297, 299, 308 |
| mfnc.dnTz | mfnc.dnTmz | Pa | Maxwell downward surface stress tensor, z component | Boundaries 286–289, 291, 293, 295, 297, 299, 308 |
| mfnc.unx | unx |  | Normal vector up direction, x component | Boundaries 286–289, 291, 293, 295, 297, 299, 308 |
| mfnc.uny | uny |  | Normal vector up direction, y component | Boundaries 286–289, 291, 293, 295, 297, 299, 308 |
| mfnc.unz | unz |  | Normal vector up direction, z component | Boundaries 286–289, 291, 293, 295, 297, 299, 308 |
| mfnc.dnx | dnx |  | Normal vector down direction, x component | Boundaries 286–289, 291, 293, 295, 297, 299, 308 |
| mfnc.dny | dny |  | Normal vector down direction, y component | Boundaries 286–289, 291, 293, 295, 297, 299, 308 |
| mfnc.dnz | dnz |  | Normal vector down direction, z component | Boundaries 286–289, 291, 293, 295, 297, 299, 308 |
| mfnc.W | mfnc.Wm | J/m^3 | Energy density | Domain 31 |
| mfnc.dWm | mfnc.Wm | J/m^3 | Integrand for total magnetic energy | Domain 31 |
| mfnc.Wm | 0.5\*mu0\_const\*((mfnc.murxx\*mfnc.Hx+mfnc.murxy\*mfnc.Hy+mfnc.murxz\*mfnc.Hz)\*mfnc.Hx+(mfnc.muryx\*mfnc.Hx+mfnc.muryy\*mfnc.Hy+mfnc.muryz\*mfnc.Hz)\*mfnc.Hy+(mfnc.murzx\*mfnc.Hx+mfnc.murzy\*mfnc.Hy+mfnc.murzz\*mfnc.Hz)\*mfnc.Hz) | J/m^3 | Magnetic energy density | Domain 31 |

##### Shape Functions

| Name | Shape function | Unit | Description | Shape frame | Selection |
| --- | --- | --- | --- | --- | --- |
| Vm | Lagrange (Quadratic) | A | Magnetic scalar potential | Material | Domain 31 |

##### Weak Expressions

| Weak expression | Integration frame | Selection |
| --- | --- | --- |
| mfnc.d\*(-mfnc.Bx\*test(Vmx)-mfnc.By\*test(Vmy)-mfnc.Bz\*test(Vmz)) | Material | Domain 31 |

#### 2.4.24. ArrayAMagnet3

ArrayAMagnet3

Selection

| Geometric entity level | Domain |
| Selection | Domain 25 |

Equations

Settings

| Description | Value |
| Constitutive relation | Remanent flux density |
| Remanent flux density, x component | BA\*cos(2\*pi\*(2\*60)/360) |
| Remanent flux density, y component | BA\*sin(2\*pi\*(2\*60)/360) |
| Remanent flux density, z component | 0 |
| Relative permeability | From material |
| Relative permeability | {{1, 0, 0}, {0, 1, 0}, {0, 0, 1}} |

Properties from material

| Property | Material | Property group |
| Relative permeability | Soft Iron (without losses) | Basic |

##### Variables

| Name | Expression | Unit | Description | Selection |
| --- | --- | --- | --- | --- |
| mfnc.dnTmx | -0.5\*mfnc.unx\*(real(down(mfnc.Bx))\*real(down(mfnc.Hx))+real(down(mfnc.By))\*real(down(mfnc.Hy))+real(down(mfnc.Bz))\*real(down(mfnc.Hz)))+real(down(mfnc.Bx))\*(real(down(mfnc.Hx))\*mfnc.unx+real(down(mfnc.Hy))\*mfnc.uny+real(down(mfnc.Hz))\*mfnc.unz) | Pa | Maxwell downward magnetic surface stress tensor, x component | Boundaries 224–227, 229, 233, 235, 247, 249, 265 |
| mfnc.dnTmy | -0.5\*mfnc.uny\*(real(down(mfnc.Bx))\*real(down(mfnc.Hx))+real(down(mfnc.By))\*real(down(mfnc.Hy))+real(down(mfnc.Bz))\*real(down(mfnc.Hz)))+real(down(mfnc.By))\*(real(down(mfnc.Hx))\*mfnc.unx+real(down(mfnc.Hy))\*mfnc.uny+real(down(mfnc.Hz))\*mfnc.unz) | Pa | Maxwell downward magnetic surface stress tensor, y component | Boundaries 224–227, 229, 233, 235, 247, 249, 265 |
| mfnc.dnTmz | -0.5\*mfnc.unz\*(real(down(mfnc.Bx))\*real(down(mfnc.Hx))+real(down(mfnc.By))\*real(down(mfnc.Hy))+real(down(mfnc.Bz))\*real(down(mfnc.Hz)))+real(down(mfnc.Bz))\*(real(down(mfnc.Hx))\*mfnc.unx+real(down(mfnc.Hy))\*mfnc.uny+real(down(mfnc.Hz))\*mfnc.unz) | Pa | Maxwell downward magnetic surface stress tensor, z component | Boundaries 224–227, 229, 233, 235, 247, 249, 265 |
| mfnc.Hx | -Vmx | A/m | Magnetic field, x component | Domain 25 |
| mfnc.Hy | -Vmy | A/m | Magnetic field, y component | Domain 25 |
| mfnc.Hz | -Vmz | A/m | Magnetic field, z component | Domain 25 |
| mfnc.tHx | -VmTx | A/m | Tangential magnetic field, x component | Boundaries 224–227, 229, 233, 235, 247, 249, 265 |
| mfnc.tHy | -VmTy | A/m | Tangential magnetic field, y component | Boundaries 224–227, 229, 233, 235, 247, 249, 265 |
| mfnc.tHz | -VmTz | A/m | Tangential magnetic field, z component | Boundaries 224–227, 229, 233, 235, 247, 249, 265 |
| mfnc.normH | sqrt(realdot(mfnc.Hx,mfnc.Hx)+realdot(mfnc.Hy,mfnc.Hy)+realdot(mfnc.Hz,mfnc.Hz)) | A/m | Magnetic field norm | Domain 25 |
| mfnc.murxx | model.input.mur11 | 1 | Relative permeability, xx component | Domain 25 |
| mfnc.muryx | model.input.mur21 | 1 | Relative permeability, yx component | Domain 25 |
| mfnc.murzx | model.input.mur31 | 1 | Relative permeability, zx component | Domain 25 |
| mfnc.murxy | model.input.mur12 | 1 | Relative permeability, xy component | Domain 25 |
| mfnc.muryy | model.input.mur22 | 1 | Relative permeability, yy component | Domain 25 |
| mfnc.murzy | model.input.mur32 | 1 | Relative permeability, zy component | Domain 25 |
| mfnc.murxz | model.input.mur13 | 1 | Relative permeability, xz component | Domain 25 |
| mfnc.muryz | model.input.mur23 | 1 | Relative permeability, yz component | Domain 25 |
| mfnc.murzz | model.input.mur33 | 1 | Relative permeability, zz component | Domain 25 |
| mfnc.Bx | mu0\_const\*mfnc.Ixx\*mfnc.Hx+mu0\_const\*mfnc.Ixy\*mfnc.Hy+mu0\_const\*mfnc.Ixz\*mfnc.Hz+mu0\_const\*mfnc.chimxx\*mfnc.Hx+mu0\_const\*mfnc.chimxy\*mfnc.Hy+mu0\_const\*mfnc.chimxz\*mfnc.Hz+mfnc.Brx | T | Magnetic flux density, x component | Domain 25 |
| mfnc.By | mu0\_const\*mfnc.Iyx\*mfnc.Hx+mu0\_const\*mfnc.Iyy\*mfnc.Hy+mu0\_const\*mfnc.Iyz\*mfnc.Hz+mu0\_const\*mfnc.chimyx\*mfnc.Hx+mu0\_const\*mfnc.chimyy\*mfnc.Hy+mu0\_const\*mfnc.chimyz\*mfnc.Hz+mfnc.Bry | T | Magnetic flux density, y component | Domain 25 |
| mfnc.Bz | mu0\_const\*mfnc.Izx\*mfnc.Hx+mu0\_const\*mfnc.Izy\*mfnc.Hy+mu0\_const\*mfnc.Izz\*mfnc.Hz+mu0\_const\*mfnc.chimzx\*mfnc.Hx+mu0\_const\*mfnc.chimzy\*mfnc.Hy+mu0\_const\*mfnc.chimzz\*mfnc.Hz+mfnc.Brz | T | Magnetic flux density, z component | Domain 25 |
| mfnc.normB | sqrt(realdot(mfnc.Bx,mfnc.Bx)+realdot(mfnc.By,mfnc.By)+realdot(mfnc.Bz,mfnc.Bz)) | T | Magnetic flux density norm | Domain 25 |
| mfnc.Mx | mfnc.Bx/mu0\_const-mfnc.Ixx\*mfnc.Hx-mfnc.Ixy\*mfnc.Hy-mfnc.Ixz\*mfnc.Hz | A/m | Magnetization, x component | Domain 25 |
| mfnc.My | mfnc.By/mu0\_const-mfnc.Iyx\*mfnc.Hx-mfnc.Iyy\*mfnc.Hy-mfnc.Iyz\*mfnc.Hz | A/m | Magnetization, y component | Domain 25 |
| mfnc.Mz | mfnc.Bz/mu0\_const-mfnc.Izx\*mfnc.Hx-mfnc.Izy\*mfnc.Hy-mfnc.Izz\*mfnc.Hz | A/m | Magnetization, z component | Domain 25 |
| mfnc.normM | sqrt(realdot(mfnc.Mx,mfnc.Mx)+realdot(mfnc.My,mfnc.My)+realdot(mfnc.Mz,mfnc.Mz)) | A/m | Magnetization norm | Domain 25 |
| mfnc.Ixx | 1 | 1 | Spatial identity matrix, xx component | Domain 25 |
| mfnc.Iyx | 0 | 1 | Spatial identity matrix, yx component | Domain 25 |
| mfnc.Izx | 0 | 1 | Spatial identity matrix, zx component | Domain 25 |
| mfnc.Ixy | 0 | 1 | Spatial identity matrix, xy component | Domain 25 |
| mfnc.Iyy | 1 | 1 | Spatial identity matrix, yy component | Domain 25 |
| mfnc.Izy | 0 | 1 | Spatial identity matrix, zy component | Domain 25 |
| mfnc.Ixz | 0 | 1 | Spatial identity matrix, xz component | Domain 25 |
| mfnc.Iyz | 0 | 1 | Spatial identity matrix, yz component | Domain 25 |
| mfnc.Izz | 1 | 1 | Spatial identity matrix, zz component | Domain 25 |
| mfnc.chimxx | -1+mfnc.murxx | 1 | Magnetic susceptibility, xx component | Domain 25 |
| mfnc.chimyx | mfnc.muryx | 1 | Magnetic susceptibility, yx component | Domain 25 |
| mfnc.chimzx | mfnc.murzx | 1 | Magnetic susceptibility, zx component | Domain 25 |
| mfnc.chimxy | mfnc.murxy | 1 | Magnetic susceptibility, xy component | Domain 25 |
| mfnc.chimyy | -1+mfnc.muryy | 1 | Magnetic susceptibility, yy component | Domain 25 |
| mfnc.chimzy | mfnc.murzy | 1 | Magnetic susceptibility, zy component | Domain 25 |
| mfnc.chimxz | mfnc.murxz | 1 | Magnetic susceptibility, xz component | Domain 25 |
| mfnc.chimyz | mfnc.muryz | 1 | Magnetic susceptibility, yz component | Domain 25 |
| mfnc.chimzz | -1+mfnc.murzz | 1 | Magnetic susceptibility, zz component | Domain 25 |
| mfnc.Brx | BA\*cos(2\*pi/3) | T | Remanent flux density, x component | Domain 25 |
| mfnc.Bry | BA\*sin(2\*pi/3) | T | Remanent flux density, y component | Domain 25 |
| mfnc.Brz | 0 | T | Remanent flux density, z component | Domain 25 |
| mfnc.normBr | sqrt(realdot(mfnc.Brx,mfnc.Brx)+realdot(mfnc.Bry,mfnc.Bry)+realdot(mfnc.Brz,mfnc.Brz)) | T | Remanent flux density norm | Domain 25 |
| mfnc.unTx | 0 | Pa | Maxwell upward surface stress tensor, x component | Boundaries 224–227, 229, 233, 235, 247, 249, 265 |
| mfnc.unTy | 0 | Pa | Maxwell upward surface stress tensor, y component | Boundaries 224–227, 229, 233, 235, 247, 249, 265 |
| mfnc.unTz | 0 | Pa | Maxwell upward surface stress tensor, z component | Boundaries 224–227, 229, 233, 235, 247, 249, 265 |
| mfnc.dnTx | mfnc.dnTmx | Pa | Maxwell downward surface stress tensor, x component | Boundaries 224–227, 229, 233, 235, 247, 249, 265 |
| mfnc.dnTy | mfnc.dnTmy | Pa | Maxwell downward surface stress tensor, y component | Boundaries 224–227, 229, 233, 235, 247, 249, 265 |
| mfnc.dnTz | mfnc.dnTmz | Pa | Maxwell downward surface stress tensor, z component | Boundaries 224–227, 229, 233, 235, 247, 249, 265 |
| mfnc.unx | unx |  | Normal vector up direction, x component | Boundaries 224–227, 229, 233, 235, 247, 249, 265 |
| mfnc.uny | uny |  | Normal vector up direction, y component | Boundaries 224–227, 229, 233, 235, 247, 249, 265 |
| mfnc.unz | unz |  | Normal vector up direction, z component | Boundaries 224–227, 229, 233, 235, 247, 249, 265 |
| mfnc.dnx | dnx |  | Normal vector down direction, x component | Boundaries 224–227, 229, 233, 235, 247, 249, 265 |
| mfnc.dny | dny |  | Normal vector down direction, y component | Boundaries 224–227, 229, 233, 235, 247, 249, 265 |
| mfnc.dnz | dnz |  | Normal vector down direction, z component | Boundaries 224–227, 229, 233, 235, 247, 249, 265 |
| mfnc.W | mfnc.Wm | J/m^3 | Energy density | Domain 25 |
| mfnc.dWm | mfnc.Wm | J/m^3 | Integrand for total magnetic energy | Domain 25 |
| mfnc.Wm | 0.5\*mu0\_const\*((mfnc.murxx\*mfnc.Hx+mfnc.murxy\*mfnc.Hy+mfnc.murxz\*mfnc.Hz)\*mfnc.Hx+(mfnc.muryx\*mfnc.Hx+mfnc.muryy\*mfnc.Hy+mfnc.muryz\*mfnc.Hz)\*mfnc.Hy+(mfnc.murzx\*mfnc.Hx+mfnc.murzy\*mfnc.Hy+mfnc.murzz\*mfnc.Hz)\*mfnc.Hz) | J/m^3 | Magnetic energy density | Domain 25 |

##### Shape Functions

| Name | Shape function | Unit | Description | Shape frame | Selection |
| --- | --- | --- | --- | --- | --- |
| Vm | Lagrange (Quadratic) | A | Magnetic scalar potential | Material | Domain 25 |

##### Weak Expressions

| Weak expression | Integration frame | Selection |
| --- | --- | --- |
| mfnc.d\*(-mfnc.Bx\*test(Vmx)-mfnc.By\*test(Vmy)-mfnc.Bz\*test(Vmz)) | Material | Domain 25 |

#### 2.4.25. ArrayAMagnet4

ArrayAMagnet4

Selection

| Geometric entity level | Domain |
| Selection | Domain 17 |

Equations

Settings

| Description | Value |
| Constitutive relation | Remanent flux density |
| Remanent flux density, x component | BA\*cos(2\*pi\*(3\*60)/360) |
| Remanent flux density, y component | BA\*sin(2\*pi\*(3\*60)/360) |
| Remanent flux density, z component | 0 |
| Relative permeability | From material |
| Relative permeability | {{1, 0, 0}, {0, 1, 0}, {0, 0, 1}} |

Properties from material

| Property | Material | Property group |
| Relative permeability | Soft Iron (without losses) | Basic |

##### Variables

| Name | Expression | Unit | Description | Selection |
| --- | --- | --- | --- | --- |
| mfnc.dnTmx | -0.5\*mfnc.unx\*(real(down(mfnc.Bx))\*real(down(mfnc.Hx))+real(down(mfnc.By))\*real(down(mfnc.Hy))+real(down(mfnc.Bz))\*real(down(mfnc.Hz)))+real(down(mfnc.Bx))\*(real(down(mfnc.Hx))\*mfnc.unx+real(down(mfnc.Hy))\*mfnc.uny+real(down(mfnc.Hz))\*mfnc.unz) | Pa | Maxwell downward magnetic surface stress tensor, x component | Boundaries 145–151, 178–180 |
| mfnc.dnTmy | -0.5\*mfnc.uny\*(real(down(mfnc.Bx))\*real(down(mfnc.Hx))+real(down(mfnc.By))\*real(down(mfnc.Hy))+real(down(mfnc.Bz))\*real(down(mfnc.Hz)))+real(down(mfnc.By))\*(real(down(mfnc.Hx))\*mfnc.unx+real(down(mfnc.Hy))\*mfnc.uny+real(down(mfnc.Hz))\*mfnc.unz) | Pa | Maxwell downward magnetic surface stress tensor, y component | Boundaries 145–151, 178–180 |
| mfnc.dnTmz | -0.5\*mfnc.unz\*(real(down(mfnc.Bx))\*real(down(mfnc.Hx))+real(down(mfnc.By))\*real(down(mfnc.Hy))+real(down(mfnc.Bz))\*real(down(mfnc.Hz)))+real(down(mfnc.Bz))\*(real(down(mfnc.Hx))\*mfnc.unx+real(down(mfnc.Hy))\*mfnc.uny+real(down(mfnc.Hz))\*mfnc.unz) | Pa | Maxwell downward magnetic surface stress tensor, z component | Boundaries 145–151, 178–180 |
| mfnc.Hx | -Vmx | A/m | Magnetic field, x component | Domain 17 |
| mfnc.Hy | -Vmy | A/m | Magnetic field, y component | Domain 17 |
| mfnc.Hz | -Vmz | A/m | Magnetic field, z component | Domain 17 |
| mfnc.tHx | -VmTx | A/m | Tangential magnetic field, x component | Boundaries 145–151, 178–180 |
| mfnc.tHy | -VmTy | A/m | Tangential magnetic field, y component | Boundaries 145–151, 178–180 |
| mfnc.tHz | -VmTz | A/m | Tangential magnetic field, z component | Boundaries 145–151, 178–180 |
| mfnc.normH | sqrt(realdot(mfnc.Hx,mfnc.Hx)+realdot(mfnc.Hy,mfnc.Hy)+realdot(mfnc.Hz,mfnc.Hz)) | A/m | Magnetic field norm | Domain 17 |
| mfnc.murxx | model.input.mur11 | 1 | Relative permeability, xx component | Domain 17 |
| mfnc.muryx | model.input.mur21 | 1 | Relative permeability, yx component | Domain 17 |
| mfnc.murzx | model.input.mur31 | 1 | Relative permeability, zx component | Domain 17 |
| mfnc.murxy | model.input.mur12 | 1 | Relative permeability, xy component | Domain 17 |
| mfnc.muryy | model.input.mur22 | 1 | Relative permeability, yy component | Domain 17 |
| mfnc.murzy | model.input.mur32 | 1 | Relative permeability, zy component | Domain 17 |
| mfnc.murxz | model.input.mur13 | 1 | Relative permeability, xz component | Domain 17 |
| mfnc.muryz | model.input.mur23 | 1 | Relative permeability, yz component | Domain 17 |
| mfnc.murzz | model.input.mur33 | 1 | Relative permeability, zz component | Domain 17 |
| mfnc.Bx | mu0\_const\*mfnc.Ixx\*mfnc.Hx+mu0\_const\*mfnc.Ixy\*mfnc.Hy+mu0\_const\*mfnc.Ixz\*mfnc.Hz+mu0\_const\*mfnc.chimxx\*mfnc.Hx+mu0\_const\*mfnc.chimxy\*mfnc.Hy+mu0\_const\*mfnc.chimxz\*mfnc.Hz+mfnc.Brx | T | Magnetic flux density, x component | Domain 17 |
| mfnc.By | mu0\_const\*mfnc.Iyx\*mfnc.Hx+mu0\_const\*mfnc.Iyy\*mfnc.Hy+mu0\_const\*mfnc.Iyz\*mfnc.Hz+mu0\_const\*mfnc.chimyx\*mfnc.Hx+mu0\_const\*mfnc.chimyy\*mfnc.Hy+mu0\_const\*mfnc.chimyz\*mfnc.Hz+mfnc.Bry | T | Magnetic flux density, y component | Domain 17 |
| mfnc.Bz | mu0\_const\*mfnc.Izx\*mfnc.Hx+mu0\_const\*mfnc.Izy\*mfnc.Hy+mu0\_const\*mfnc.Izz\*mfnc.Hz+mu0\_const\*mfnc.chimzx\*mfnc.Hx+mu0\_const\*mfnc.chimzy\*mfnc.Hy+mu0\_const\*mfnc.chimzz\*mfnc.Hz+mfnc.Brz | T | Magnetic flux density, z component | Domain 17 |
| mfnc.normB | sqrt(realdot(mfnc.Bx,mfnc.Bx)+realdot(mfnc.By,mfnc.By)+realdot(mfnc.Bz,mfnc.Bz)) | T | Magnetic flux density norm | Domain 17 |
| mfnc.Mx | mfnc.Bx/mu0\_const-mfnc.Ixx\*mfnc.Hx-mfnc.Ixy\*mfnc.Hy-mfnc.Ixz\*mfnc.Hz | A/m | Magnetization, x component | Domain 17 |
| mfnc.My | mfnc.By/mu0\_const-mfnc.Iyx\*mfnc.Hx-mfnc.Iyy\*mfnc.Hy-mfnc.Iyz\*mfnc.Hz | A/m | Magnetization, y component | Domain 17 |
| mfnc.Mz | mfnc.Bz/mu0\_const-mfnc.Izx\*mfnc.Hx-mfnc.Izy\*mfnc.Hy-mfnc.Izz\*mfnc.Hz | A/m | Magnetization, z component | Domain 17 |
| mfnc.normM | sqrt(realdot(mfnc.Mx,mfnc.Mx)+realdot(mfnc.My,mfnc.My)+realdot(mfnc.Mz,mfnc.Mz)) | A/m | Magnetization norm | Domain 17 |
| mfnc.Ixx | 1 | 1 | Spatial identity matrix, xx component | Domain 17 |
| mfnc.Iyx | 0 | 1 | Spatial identity matrix, yx component | Domain 17 |
| mfnc.Izx | 0 | 1 | Spatial identity matrix, zx component | Domain 17 |
| mfnc.Ixy | 0 | 1 | Spatial identity matrix, xy component | Domain 17 |
| mfnc.Iyy | 1 | 1 | Spatial identity matrix, yy component | Domain 17 |
| mfnc.Izy | 0 | 1 | Spatial identity matrix, zy component | Domain 17 |
| mfnc.Ixz | 0 | 1 | Spatial identity matrix, xz component | Domain 17 |
| mfnc.Iyz | 0 | 1 | Spatial identity matrix, yz component | Domain 17 |
| mfnc.Izz | 1 | 1 | Spatial identity matrix, zz component | Domain 17 |
| mfnc.chimxx | -1+mfnc.murxx | 1 | Magnetic susceptibility, xx component | Domain 17 |
| mfnc.chimyx | mfnc.muryx | 1 | Magnetic susceptibility, yx component | Domain 17 |
| mfnc.chimzx | mfnc.murzx | 1 | Magnetic susceptibility, zx component | Domain 17 |
| mfnc.chimxy | mfnc.murxy | 1 | Magnetic susceptibility, xy component | Domain 17 |
| mfnc.chimyy | -1+mfnc.muryy | 1 | Magnetic susceptibility, yy component | Domain 17 |
| mfnc.chimzy | mfnc.murzy | 1 | Magnetic susceptibility, zy component | Domain 17 |
| mfnc.chimxz | mfnc.murxz | 1 | Magnetic susceptibility, xz component | Domain 17 |
| mfnc.chimyz | mfnc.muryz | 1 | Magnetic susceptibility, yz component | Domain 17 |
| mfnc.chimzz | -1+mfnc.murzz | 1 | Magnetic susceptibility, zz component | Domain 17 |
| mfnc.Brx | BA\*cos(pi) | T | Remanent flux density, x component | Domain 17 |
| mfnc.Bry | BA\*sin(pi) | T | Remanent flux density, y component | Domain 17 |
| mfnc.Brz | 0 | T | Remanent flux density, z component | Domain 17 |
| mfnc.normBr | sqrt(realdot(mfnc.Brx,mfnc.Brx)+realdot(mfnc.Bry,mfnc.Bry)+realdot(mfnc.Brz,mfnc.Brz)) | T | Remanent flux density norm | Domain 17 |
| mfnc.unTx | 0 | Pa | Maxwell upward surface stress tensor, x component | Boundaries 145–151, 178–180 |
| mfnc.unTy | 0 | Pa | Maxwell upward surface stress tensor, y component | Boundaries 145–151, 178–180 |
| mfnc.unTz | 0 | Pa | Maxwell upward surface stress tensor, z component | Boundaries 145–151, 178–180 |
| mfnc.dnTx | mfnc.dnTmx | Pa | Maxwell downward surface stress tensor, x component | Boundaries 145–151, 178–180 |
| mfnc.dnTy | mfnc.dnTmy | Pa | Maxwell downward surface stress tensor, y component | Boundaries 145–151, 178–180 |
| mfnc.dnTz | mfnc.dnTmz | Pa | Maxwell downward surface stress tensor, z component | Boundaries 145–151, 178–180 |
| mfnc.unx | unx |  | Normal vector up direction, x component | Boundaries 145–151, 178–180 |
| mfnc.uny | uny |  | Normal vector up direction, y component | Boundaries 145–151, 178–180 |
| mfnc.unz | unz |  | Normal vector up direction, z component | Boundaries 145–151, 178–180 |
| mfnc.dnx | dnx |  | Normal vector down direction, x component | Boundaries 145–151, 178–180 |
| mfnc.dny | dny |  | Normal vector down direction, y component | Boundaries 145–151, 178–180 |
| mfnc.dnz | dnz |  | Normal vector down direction, z component | Boundaries 145–151, 178–180 |
| mfnc.W | mfnc.Wm | J/m^3 | Energy density | Domain 17 |
| mfnc.dWm | mfnc.Wm | J/m^3 | Integrand for total magnetic energy | Domain 17 |
| mfnc.Wm | 0.5\*mu0\_const\*((mfnc.murxx\*mfnc.Hx+mfnc.murxy\*mfnc.Hy+mfnc.murxz\*mfnc.Hz)\*mfnc.Hx+(mfnc.muryx\*mfnc.Hx+mfnc.muryy\*mfnc.Hy+mfnc.muryz\*mfnc.Hz)\*mfnc.Hy+(mfnc.murzx\*mfnc.Hx+mfnc.murzy\*mfnc.Hy+mfnc.murzz\*mfnc.Hz)\*mfnc.Hz) | J/m^3 | Magnetic energy density | Domain 17 |

##### Shape Functions

| Name | Shape function | Unit | Description | Shape frame | Selection |
| --- | --- | --- | --- | --- | --- |
| Vm | Lagrange (Quadratic) | A | Magnetic scalar potential | Material | Domain 17 |

##### Weak Expressions

| Weak expression | Integration frame | Selection |
| --- | --- | --- |
| mfnc.d\*(-mfnc.Bx\*test(Vmx)-mfnc.By\*test(Vmy)-mfnc.Bz\*test(Vmz)) | Material | Domain 17 |

#### 2.4.26. ArrayAMagnet5

ArrayAMagnet5

Selection

| Geometric entity level | Domain |
| Selection | Domain 9 |

Equations

Settings

| Description | Value |
| Constitutive relation | Remanent flux density |
| Remanent flux density, x component | BA\*cos(2\*pi\*(4\*60)/360) |
| Remanent flux density, y component | BA\*sin(2\*pi\*(4\*60)/360) |
| Remanent flux density, z component | 0 |
| Relative permeability | From material |
| Relative permeability | {{1, 0, 0}, {0, 1, 0}, {0, 0, 1}} |

Properties from material

| Property | Material | Property group |
| Relative permeability | Soft Iron (without losses) | Basic |

##### Variables

| Name | Expression | Unit | Description | Selection |
| --- | --- | --- | --- | --- |
| mfnc.dnTmx | -0.5\*mfnc.unx\*(real(down(mfnc.Bx))\*real(down(mfnc.Hx))+real(down(mfnc.By))\*real(down(mfnc.Hy))+real(down(mfnc.Bz))\*real(down(mfnc.Hz)))+real(down(mfnc.Bx))\*(real(down(mfnc.Hx))\*mfnc.unx+real(down(mfnc.Hy))\*mfnc.uny+real(down(mfnc.Hz))\*mfnc.unz) | Pa | Maxwell downward magnetic surface stress tensor, x component | Boundaries 64–67, 69, 77, 79, 91, 93, 105 |
| mfnc.dnTmy | -0.5\*mfnc.uny\*(real(down(mfnc.Bx))\*real(down(mfnc.Hx))+real(down(mfnc.By))\*real(down(mfnc.Hy))+real(down(mfnc.Bz))\*real(down(mfnc.Hz)))+real(down(mfnc.By))\*(real(down(mfnc.Hx))\*mfnc.unx+real(down(mfnc.Hy))\*mfnc.uny+real(down(mfnc.Hz))\*mfnc.unz) | Pa | Maxwell downward magnetic surface stress tensor, y component | Boundaries 64–67, 69, 77, 79, 91, 93, 105 |
| mfnc.dnTmz | -0.5\*mfnc.unz\*(real(down(mfnc.Bx))\*real(down(mfnc.Hx))+real(down(mfnc.By))\*real(down(mfnc.Hy))+real(down(mfnc.Bz))\*real(down(mfnc.Hz)))+real(down(mfnc.Bz))\*(real(down(mfnc.Hx))\*mfnc.unx+real(down(mfnc.Hy))\*mfnc.uny+real(down(mfnc.Hz))\*mfnc.unz) | Pa | Maxwell downward magnetic surface stress tensor, z component | Boundaries 64–67, 69, 77, 79, 91, 93, 105 |
| mfnc.Hx | -Vmx | A/m | Magnetic field, x component | Domain 9 |
| mfnc.Hy | -Vmy | A/m | Magnetic field, y component | Domain 9 |
| mfnc.Hz | -Vmz | A/m | Magnetic field, z component | Domain 9 |
| mfnc.tHx | -VmTx | A/m | Tangential magnetic field, x component | Boundaries 64–67, 69, 77, 79, 91, 93, 105 |
| mfnc.tHy | -VmTy | A/m | Tangential magnetic field, y component | Boundaries 64–67, 69, 77, 79, 91, 93, 105 |
| mfnc.tHz | -VmTz | A/m | Tangential magnetic field, z component | Boundaries 64–67, 69, 77, 79, 91, 93, 105 |
| mfnc.normH | sqrt(realdot(mfnc.Hx,mfnc.Hx)+realdot(mfnc.Hy,mfnc.Hy)+realdot(mfnc.Hz,mfnc.Hz)) | A/m | Magnetic field norm | Domain 9 |
| mfnc.murxx | model.input.mur11 | 1 | Relative permeability, xx component | Domain 9 |
| mfnc.muryx | model.input.mur21 | 1 | Relative permeability, yx component | Domain 9 |
| mfnc.murzx | model.input.mur31 | 1 | Relative permeability, zx component | Domain 9 |
| mfnc.murxy | model.input.mur12 | 1 | Relative permeability, xy component | Domain 9 |
| mfnc.muryy | model.input.mur22 | 1 | Relative permeability, yy component | Domain 9 |
| mfnc.murzy | model.input.mur32 | 1 | Relative permeability, zy component | Domain 9 |
| mfnc.murxz | model.input.mur13 | 1 | Relative permeability, xz component | Domain 9 |
| mfnc.muryz | model.input.mur23 | 1 | Relative permeability, yz component | Domain 9 |
| mfnc.murzz | model.input.mur33 | 1 | Relative permeability, zz component | Domain 9 |
| mfnc.Bx | mu0\_const\*mfnc.Ixx\*mfnc.Hx+mu0\_const\*mfnc.Ixy\*mfnc.Hy+mu0\_const\*mfnc.Ixz\*mfnc.Hz+mu0\_const\*mfnc.chimxx\*mfnc.Hx+mu0\_const\*mfnc.chimxy\*mfnc.Hy+mu0\_const\*mfnc.chimxz\*mfnc.Hz+mfnc.Brx | T | Magnetic flux density, x component | Domain 9 |
| mfnc.By | mu0\_const\*mfnc.Iyx\*mfnc.Hx+mu0\_const\*mfnc.Iyy\*mfnc.Hy+mu0\_const\*mfnc.Iyz\*mfnc.Hz+mu0\_const\*mfnc.chimyx\*mfnc.Hx+mu0\_const\*mfnc.chimyy\*mfnc.Hy+mu0\_const\*mfnc.chimyz\*mfnc.Hz+mfnc.Bry | T | Magnetic flux density, y component | Domain 9 |
| mfnc.Bz | mu0\_const\*mfnc.Izx\*mfnc.Hx+mu0\_const\*mfnc.Izy\*mfnc.Hy+mu0\_const\*mfnc.Izz\*mfnc.Hz+mu0\_const\*mfnc.chimzx\*mfnc.Hx+mu0\_const\*mfnc.chimzy\*mfnc.Hy+mu0\_const\*mfnc.chimzz\*mfnc.Hz+mfnc.Brz | T | Magnetic flux density, z component | Domain 9 |
| mfnc.normB | sqrt(realdot(mfnc.Bx,mfnc.Bx)+realdot(mfnc.By,mfnc.By)+realdot(mfnc.Bz,mfnc.Bz)) | T | Magnetic flux density norm | Domain 9 |
| mfnc.Mx | mfnc.Bx/mu0\_const-mfnc.Ixx\*mfnc.Hx-mfnc.Ixy\*mfnc.Hy-mfnc.Ixz\*mfnc.Hz | A/m | Magnetization, x component | Domain 9 |
| mfnc.My | mfnc.By/mu0\_const-mfnc.Iyx\*mfnc.Hx-mfnc.Iyy\*mfnc.Hy-mfnc.Iyz\*mfnc.Hz | A/m | Magnetization, y component | Domain 9 |
| mfnc.Mz | mfnc.Bz/mu0\_const-mfnc.Izx\*mfnc.Hx-mfnc.Izy\*mfnc.Hy-mfnc.Izz\*mfnc.Hz | A/m | Magnetization, z component | Domain 9 |
| mfnc.normM | sqrt(realdot(mfnc.Mx,mfnc.Mx)+realdot(mfnc.My,mfnc.My)+realdot(mfnc.Mz,mfnc.Mz)) | A/m | Magnetization norm | Domain 9 |
| mfnc.Ixx | 1 | 1 | Spatial identity matrix, xx component | Domain 9 |
| mfnc.Iyx | 0 | 1 | Spatial identity matrix, yx component | Domain 9 |
| mfnc.Izx | 0 | 1 | Spatial identity matrix, zx component | Domain 9 |
| mfnc.Ixy | 0 | 1 | Spatial identity matrix, xy component | Domain 9 |
| mfnc.Iyy | 1 | 1 | Spatial identity matrix, yy component | Domain 9 |
| mfnc.Izy | 0 | 1 | Spatial identity matrix, zy component | Domain 9 |
| mfnc.Ixz | 0 | 1 | Spatial identity matrix, xz component | Domain 9 |
| mfnc.Iyz | 0 | 1 | Spatial identity matrix, yz component | Domain 9 |
| mfnc.Izz | 1 | 1 | Spatial identity matrix, zz component | Domain 9 |
| mfnc.chimxx | -1+mfnc.murxx | 1 | Magnetic susceptibility, xx component | Domain 9 |
| mfnc.chimyx | mfnc.muryx | 1 | Magnetic susceptibility, yx component | Domain 9 |
| mfnc.chimzx | mfnc.murzx | 1 | Magnetic susceptibility, zx component | Domain 9 |
| mfnc.chimxy | mfnc.murxy | 1 | Magnetic susceptibility, xy component | Domain 9 |
| mfnc.chimyy | -1+mfnc.muryy | 1 | Magnetic susceptibility, yy component | Domain 9 |
| mfnc.chimzy | mfnc.murzy | 1 | Magnetic susceptibility, zy component | Domain 9 |
| mfnc.chimxz | mfnc.murxz | 1 | Magnetic susceptibility, xz component | Domain 9 |
| mfnc.chimyz | mfnc.muryz | 1 | Magnetic susceptibility, yz component | Domain 9 |
| mfnc.chimzz | -1+mfnc.murzz | 1 | Magnetic susceptibility, zz component | Domain 9 |
| mfnc.Brx | BA\*cos(4\*pi/3) | T | Remanent flux density, x component | Domain 9 |
| mfnc.Bry | BA\*sin(4\*pi/3) | T | Remanent flux density, y component | Domain 9 |
| mfnc.Brz | 0 | T | Remanent flux density, z component | Domain 9 |
| mfnc.normBr | sqrt(realdot(mfnc.Brx,mfnc.Brx)+realdot(mfnc.Bry,mfnc.Bry)+realdot(mfnc.Brz,mfnc.Brz)) | T | Remanent flux density norm | Domain 9 |
| mfnc.unTx | 0 | Pa | Maxwell upward surface stress tensor, x component | Boundaries 64–67, 69, 77, 79, 91, 93, 105 |
| mfnc.unTy | 0 | Pa | Maxwell upward surface stress tensor, y component | Boundaries 64–67, 69, 77, 79, 91, 93, 105 |
| mfnc.unTz | 0 | Pa | Maxwell upward surface stress tensor, z component | Boundaries 64–67, 69, 77, 79, 91, 93, 105 |
| mfnc.dnTx | mfnc.dnTmx | Pa | Maxwell downward surface stress tensor, x component | Boundaries 64–67, 69, 77, 79, 91, 93, 105 |
| mfnc.dnTy | mfnc.dnTmy | Pa | Maxwell downward surface stress tensor, y component | Boundaries 64–67, 69, 77, 79, 91, 93, 105 |
| mfnc.dnTz | mfnc.dnTmz | Pa | Maxwell downward surface stress tensor, z component | Boundaries 64–67, 69, 77, 79, 91, 93, 105 |
| mfnc.unx | unx |  | Normal vector up direction, x component | Boundaries 64–67, 69, 77, 79, 91, 93, 105 |
| mfnc.uny | uny |  | Normal vector up direction, y component | Boundaries 64–67, 69, 77, 79, 91, 93, 105 |
| mfnc.unz | unz |  | Normal vector up direction, z component | Boundaries 64–67, 69, 77, 79, 91, 93, 105 |
| mfnc.dnx | dnx |  | Normal vector down direction, x component | Boundaries 64–67, 69, 77, 79, 91, 93, 105 |
| mfnc.dny | dny |  | Normal vector down direction, y component | Boundaries 64–67, 69, 77, 79, 91, 93, 105 |
| mfnc.dnz | dnz |  | Normal vector down direction, z component | Boundaries 64–67, 69, 77, 79, 91, 93, 105 |
| mfnc.W | mfnc.Wm | J/m^3 | Energy density | Domain 9 |
| mfnc.dWm | mfnc.Wm | J/m^3 | Integrand for total magnetic energy | Domain 9 |
| mfnc.Wm | 0.5\*mu0\_const\*((mfnc.murxx\*mfnc.Hx+mfnc.murxy\*mfnc.Hy+mfnc.murxz\*mfnc.Hz)\*mfnc.Hx+(mfnc.muryx\*mfnc.Hx+mfnc.muryy\*mfnc.Hy+mfnc.muryz\*mfnc.Hz)\*mfnc.Hy+(mfnc.murzx\*mfnc.Hx+mfnc.murzy\*mfnc.Hy+mfnc.murzz\*mfnc.Hz)\*mfnc.Hz) | J/m^3 | Magnetic energy density | Domain 9 |

##### Shape Functions

| Name | Shape function | Unit | Description | Shape frame | Selection |
| --- | --- | --- | --- | --- | --- |
| Vm | Lagrange (Quadratic) | A | Magnetic scalar potential | Material | Domain 9 |

##### Weak Expressions

| Weak expression | Integration frame | Selection |
| --- | --- | --- |
| mfnc.d\*(-mfnc.Bx\*test(Vmx)-mfnc.By\*test(Vmy)-mfnc.Bz\*test(Vmz)) | Material | Domain 9 |

#### 2.4.27. ArrayAMagnet6

ArrayAMagnet6

Selection

| Geometric entity level | Domain |
| Selection | Domain 4 |

Equations

Settings

| Description | Value |
| Constitutive relation | Remanent flux density |
| Remanent flux density, x component | BA\*cos(2\*pi\*(5\*60)/360) |
| Remanent flux density, y component | BA\*sin(2\*pi\*(5\*60)/360) |
| Remanent flux density, z component | 0 |
| Relative permeability | From material |
| Relative permeability | {{1, 0, 0}, {0, 1, 0}, {0, 0, 1}} |

Properties from material

| Property | Material | Property group |
| Relative permeability | Soft Iron (without losses) | Basic |

##### Variables

| Name | Expression | Unit | Description | Selection |
| --- | --- | --- | --- | --- |
| mfnc.dnTmx | -0.5\*mfnc.unx\*(real(down(mfnc.Bx))\*real(down(mfnc.Hx))+real(down(mfnc.By))\*real(down(mfnc.Hy))+real(down(mfnc.Bz))\*real(down(mfnc.Hz)))+real(down(mfnc.Bx))\*(real(down(mfnc.Hx))\*mfnc.unx+real(down(mfnc.Hy))\*mfnc.uny+real(down(mfnc.Hz))\*mfnc.unz) | Pa | Maxwell downward magnetic surface stress tensor, x component | Boundaries 17–20, 22, 27, 29, 31, 33, 35 |
| mfnc.dnTmy | -0.5\*mfnc.uny\*(real(down(mfnc.Bx))\*real(down(mfnc.Hx))+real(down(mfnc.By))\*real(down(mfnc.Hy))+real(down(mfnc.Bz))\*real(down(mfnc.Hz)))+real(down(mfnc.By))\*(real(down(mfnc.Hx))\*mfnc.unx+real(down(mfnc.Hy))\*mfnc.uny+real(down(mfnc.Hz))\*mfnc.unz) | Pa | Maxwell downward magnetic surface stress tensor, y component | Boundaries 17–20, 22, 27, 29, 31, 33, 35 |
| mfnc.dnTmz | -0.5\*mfnc.unz\*(real(down(mfnc.Bx))\*real(down(mfnc.Hx))+real(down(mfnc.By))\*real(down(mfnc.Hy))+real(down(mfnc.Bz))\*real(down(mfnc.Hz)))+real(down(mfnc.Bz))\*(real(down(mfnc.Hx))\*mfnc.unx+real(down(mfnc.Hy))\*mfnc.uny+real(down(mfnc.Hz))\*mfnc.unz) | Pa | Maxwell downward magnetic surface stress tensor, z component | Boundaries 17–20, 22, 27, 29, 31, 33, 35 |
| mfnc.Hx | -Vmx | A/m | Magnetic field, x component | Domain 4 |
| mfnc.Hy | -Vmy | A/m | Magnetic field, y component | Domain 4 |
| mfnc.Hz | -Vmz | A/m | Magnetic field, z component | Domain 4 |
| mfnc.tHx | -VmTx | A/m | Tangential magnetic field, x component | Boundaries 17–20, 22, 27, 29, 31, 33, 35 |
| mfnc.tHy | -VmTy | A/m | Tangential magnetic field, y component | Boundaries 17–20, 22, 27, 29, 31, 33, 35 |
| mfnc.tHz | -VmTz | A/m | Tangential magnetic field, z component | Boundaries 17–20, 22, 27, 29, 31, 33, 35 |
| mfnc.normH | sqrt(realdot(mfnc.Hx,mfnc.Hx)+realdot(mfnc.Hy,mfnc.Hy)+realdot(mfnc.Hz,mfnc.Hz)) | A/m | Magnetic field norm | Domain 4 |
| mfnc.murxx | model.input.mur11 | 1 | Relative permeability, xx component | Domain 4 |
| mfnc.muryx | model.input.mur21 | 1 | Relative permeability, yx component | Domain 4 |
| mfnc.murzx | model.input.mur31 | 1 | Relative permeability, zx component | Domain 4 |
| mfnc.murxy | model.input.mur12 | 1 | Relative permeability, xy component | Domain 4 |
| mfnc.muryy | model.input.mur22 | 1 | Relative permeability, yy component | Domain 4 |
| mfnc.murzy | model.input.mur32 | 1 | Relative permeability, zy component | Domain 4 |
| mfnc.murxz | model.input.mur13 | 1 | Relative permeability, xz component | Domain 4 |
| mfnc.muryz | model.input.mur23 | 1 | Relative permeability, yz component | Domain 4 |
| mfnc.murzz | model.input.mur33 | 1 | Relative permeability, zz component | Domain 4 |
| mfnc.Bx | mu0\_const\*mfnc.Ixx\*mfnc.Hx+mu0\_const\*mfnc.Ixy\*mfnc.Hy+mu0\_const\*mfnc.Ixz\*mfnc.Hz+mu0\_const\*mfnc.chimxx\*mfnc.Hx+mu0\_const\*mfnc.chimxy\*mfnc.Hy+mu0\_const\*mfnc.chimxz\*mfnc.Hz+mfnc.Brx | T | Magnetic flux density, x component | Domain 4 |
| mfnc.By | mu0\_const\*mfnc.Iyx\*mfnc.Hx+mu0\_const\*mfnc.Iyy\*mfnc.Hy+mu0\_const\*mfnc.Iyz\*mfnc.Hz+mu0\_const\*mfnc.chimyx\*mfnc.Hx+mu0\_const\*mfnc.chimyy\*mfnc.Hy+mu0\_const\*mfnc.chimyz\*mfnc.Hz+mfnc.Bry | T | Magnetic flux density, y component | Domain 4 |
| mfnc.Bz | mu0\_const\*mfnc.Izx\*mfnc.Hx+mu0\_const\*mfnc.Izy\*mfnc.Hy+mu0\_const\*mfnc.Izz\*mfnc.Hz+mu0\_const\*mfnc.chimzx\*mfnc.Hx+mu0\_const\*mfnc.chimzy\*mfnc.Hy+mu0\_const\*mfnc.chimzz\*mfnc.Hz+mfnc.Brz | T | Magnetic flux density, z component | Domain 4 |
| mfnc.normB | sqrt(realdot(mfnc.Bx,mfnc.Bx)+realdot(mfnc.By,mfnc.By)+realdot(mfnc.Bz,mfnc.Bz)) | T | Magnetic flux density norm | Domain 4 |
| mfnc.Mx | mfnc.Bx/mu0\_const-mfnc.Ixx\*mfnc.Hx-mfnc.Ixy\*mfnc.Hy-mfnc.Ixz\*mfnc.Hz | A/m | Magnetization, x component | Domain 4 |
| mfnc.My | mfnc.By/mu0\_const-mfnc.Iyx\*mfnc.Hx-mfnc.Iyy\*mfnc.Hy-mfnc.Iyz\*mfnc.Hz | A/m | Magnetization, y component | Domain 4 |
| mfnc.Mz | mfnc.Bz/mu0\_const-mfnc.Izx\*mfnc.Hx-mfnc.Izy\*mfnc.Hy-mfnc.Izz\*mfnc.Hz | A/m | Magnetization, z component | Domain 4 |
| mfnc.normM | sqrt(realdot(mfnc.Mx,mfnc.Mx)+realdot(mfnc.My,mfnc.My)+realdot(mfnc.Mz,mfnc.Mz)) | A/m | Magnetization norm | Domain 4 |
| mfnc.Ixx | 1 | 1 | Spatial identity matrix, xx component | Domain 4 |
| mfnc.Iyx | 0 | 1 | Spatial identity matrix, yx component | Domain 4 |
| mfnc.Izx | 0 | 1 | Spatial identity matrix, zx component | Domain 4 |
| mfnc.Ixy | 0 | 1 | Spatial identity matrix, xy component | Domain 4 |
| mfnc.Iyy | 1 | 1 | Spatial identity matrix, yy component | Domain 4 |
| mfnc.Izy | 0 | 1 | Spatial identity matrix, zy component | Domain 4 |
| mfnc.Ixz | 0 | 1 | Spatial identity matrix, xz component | Domain 4 |
| mfnc.Iyz | 0 | 1 | Spatial identity matrix, yz component | Domain 4 |
| mfnc.Izz | 1 | 1 | Spatial identity matrix, zz component | Domain 4 |
| mfnc.chimxx | -1+mfnc.murxx | 1 | Magnetic susceptibility, xx component | Domain 4 |
| mfnc.chimyx | mfnc.muryx | 1 | Magnetic susceptibility, yx component | Domain 4 |
| mfnc.chimzx | mfnc.murzx | 1 | Magnetic susceptibility, zx component | Domain 4 |
| mfnc.chimxy | mfnc.murxy | 1 | Magnetic susceptibility, xy component | Domain 4 |
| mfnc.chimyy | -1+mfnc.muryy | 1 | Magnetic susceptibility, yy component | Domain 4 |
| mfnc.chimzy | mfnc.murzy | 1 | Magnetic susceptibility, zy component | Domain 4 |
| mfnc.chimxz | mfnc.murxz | 1 | Magnetic susceptibility, xz component | Domain 4 |
| mfnc.chimyz | mfnc.muryz | 1 | Magnetic susceptibility, yz component | Domain 4 |
| mfnc.chimzz | -1+mfnc.murzz | 1 | Magnetic susceptibility, zz component | Domain 4 |
| mfnc.Brx | BA\*cos(5\*pi/3) | T | Remanent flux density, x component | Domain 4 |
| mfnc.Bry | BA\*sin(5\*pi/3) | T | Remanent flux density, y component | Domain 4 |
| mfnc.Brz | 0 | T | Remanent flux density, z component | Domain 4 |
| mfnc.normBr | sqrt(realdot(mfnc.Brx,mfnc.Brx)+realdot(mfnc.Bry,mfnc.Bry)+realdot(mfnc.Brz,mfnc.Brz)) | T | Remanent flux density norm | Domain 4 |
| mfnc.unTx | 0 | Pa | Maxwell upward surface stress tensor, x component | Boundaries 17–20, 22, 27, 29, 31, 33, 35 |
| mfnc.unTy | 0 | Pa | Maxwell upward surface stress tensor, y component | Boundaries 17–20, 22, 27, 29, 31, 33, 35 |
| mfnc.unTz | 0 | Pa | Maxwell upward surface stress tensor, z component | Boundaries 17–20, 22, 27, 29, 31, 33, 35 |
| mfnc.dnTx | mfnc.dnTmx | Pa | Maxwell downward surface stress tensor, x component | Boundaries 17–20, 22, 27, 29, 31, 33, 35 |
| mfnc.dnTy | mfnc.dnTmy | Pa | Maxwell downward surface stress tensor, y component | Boundaries 17–20, 22, 27, 29, 31, 33, 35 |
| mfnc.dnTz | mfnc.dnTmz | Pa | Maxwell downward surface stress tensor, z component | Boundaries 17–20, 22, 27, 29, 31, 33, 35 |
| mfnc.unx | unx |  | Normal vector up direction, x component | Boundaries 17–20, 22, 27, 29, 31, 33, 35 |
| mfnc.uny | uny |  | Normal vector up direction, y component | Boundaries 17–20, 22, 27, 29, 31, 33, 35 |
| mfnc.unz | unz |  | Normal vector up direction, z component | Boundaries 17–20, 22, 27, 29, 31, 33, 35 |
| mfnc.dnx | dnx |  | Normal vector down direction, x component | Boundaries 17–20, 22, 27, 29, 31, 33, 35 |
| mfnc.dny | dny |  | Normal vector down direction, y component | Boundaries 17–20, 22, 27, 29, 31, 33, 35 |
| mfnc.dnz | dnz |  | Normal vector down direction, z component | Boundaries 17–20, 22, 27, 29, 31, 33, 35 |
| mfnc.W | mfnc.Wm | J/m^3 | Energy density | Domain 4 |
| mfnc.dWm | mfnc.Wm | J/m^3 | Integrand for total magnetic energy | Domain 4 |
| mfnc.Wm | 0.5\*mu0\_const\*((mfnc.murxx\*mfnc.Hx+mfnc.murxy\*mfnc.Hy+mfnc.murxz\*mfnc.Hz)\*mfnc.Hx+(mfnc.muryx\*mfnc.Hx+mfnc.muryy\*mfnc.Hy+mfnc.muryz\*mfnc.Hz)\*mfnc.Hy+(mfnc.murzx\*mfnc.Hx+mfnc.murzy\*mfnc.Hy+mfnc.murzz\*mfnc.Hz)\*mfnc.Hz) | J/m^3 | Magnetic energy density | Domain 4 |

##### Shape Functions

| Name | Shape function | Unit | Description | Shape frame | Selection |
| --- | --- | --- | --- | --- | --- |
| Vm | Lagrange (Quadratic) | A | Magnetic scalar potential | Material | Domain 4 |

##### Weak Expressions

| Weak expression | Integration frame | Selection |
| --- | --- | --- |
| mfnc.d\*(-mfnc.Bx\*test(Vmx)-mfnc.By\*test(Vmy)-mfnc.Bz\*test(Vmz)) | Material | Domain 4 |

#### 2.4.28. ArrayAMagnet7

ArrayAMagnet7

Selection

| Geometric entity level | Domain |
| Selection | Domain 2 |

Equations

Settings

| Description | Value |
| Constitutive relation | Remanent flux density |
| Remanent flux density, x component | BA\*cos(2\*pi\*(6\*60)/360) |
| Remanent flux density, y component | BA\*sin(2\*pi\*(6\*60)/360) |
| Remanent flux density, z component | 0 |
| Relative permeability | From material |
| Relative permeability | {{1, 0, 0}, {0, 1, 0}, {0, 0, 1}} |

Properties from material

| Property | Material | Property group |
| Relative permeability | Soft Iron (without losses) | Basic |

##### Variables

| Name | Expression | Unit | Description | Selection |
| --- | --- | --- | --- | --- |
| mfnc.dnTmx | -0.5\*mfnc.unx\*(real(down(mfnc.Bx))\*real(down(mfnc.Hx))+real(down(mfnc.By))\*real(down(mfnc.Hy))+real(down(mfnc.Bz))\*real(down(mfnc.Hz)))+real(down(mfnc.Bx))\*(real(down(mfnc.Hx))\*mfnc.unx+real(down(mfnc.Hy))\*mfnc.uny+real(down(mfnc.Hz))\*mfnc.unz) | Pa | Maxwell downward magnetic surface stress tensor, x component | Boundaries 6–12, 23–25 |
| mfnc.dnTmy | -0.5\*mfnc.uny\*(real(down(mfnc.Bx))\*real(down(mfnc.Hx))+real(down(mfnc.By))\*real(down(mfnc.Hy))+real(down(mfnc.Bz))\*real(down(mfnc.Hz)))+real(down(mfnc.By))\*(real(down(mfnc.Hx))\*mfnc.unx+real(down(mfnc.Hy))\*mfnc.uny+real(down(mfnc.Hz))\*mfnc.unz) | Pa | Maxwell downward magnetic surface stress tensor, y component | Boundaries 6–12, 23–25 |
| mfnc.dnTmz | -0.5\*mfnc.unz\*(real(down(mfnc.Bx))\*real(down(mfnc.Hx))+real(down(mfnc.By))\*real(down(mfnc.Hy))+real(down(mfnc.Bz))\*real(down(mfnc.Hz)))+real(down(mfnc.Bz))\*(real(down(mfnc.Hx))\*mfnc.unx+real(down(mfnc.Hy))\*mfnc.uny+real(down(mfnc.Hz))\*mfnc.unz) | Pa | Maxwell downward magnetic surface stress tensor, z component | Boundaries 6–12, 23–25 |
| mfnc.Hx | -Vmx | A/m | Magnetic field, x component | Domain 2 |
| mfnc.Hy | -Vmy | A/m | Magnetic field, y component | Domain 2 |
| mfnc.Hz | -Vmz | A/m | Magnetic field, z component | Domain 2 |
| mfnc.tHx | -VmTx | A/m | Tangential magnetic field, x component | Boundaries 6–12, 23–25 |
| mfnc.tHy | -VmTy | A/m | Tangential magnetic field, y component | Boundaries 6–12, 23–25 |
| mfnc.tHz | -VmTz | A/m | Tangential magnetic field, z component | Boundaries 6–12, 23–25 |
| mfnc.normH | sqrt(realdot(mfnc.Hx,mfnc.Hx)+realdot(mfnc.Hy,mfnc.Hy)+realdot(mfnc.Hz,mfnc.Hz)) | A/m | Magnetic field norm | Domain 2 |
| mfnc.murxx | model.input.mur11 | 1 | Relative permeability, xx component | Domain 2 |
| mfnc.muryx | model.input.mur21 | 1 | Relative permeability, yx component | Domain 2 |
| mfnc.murzx | model.input.mur31 | 1 | Relative permeability, zx component | Domain 2 |
| mfnc.murxy | model.input.mur12 | 1 | Relative permeability, xy component | Domain 2 |
| mfnc.muryy | model.input.mur22 | 1 | Relative permeability, yy component | Domain 2 |
| mfnc.murzy | model.input.mur32 | 1 | Relative permeability, zy component | Domain 2 |
| mfnc.murxz | model.input.mur13 | 1 | Relative permeability, xz component | Domain 2 |
| mfnc.muryz | model.input.mur23 | 1 | Relative permeability, yz component | Domain 2 |
| mfnc.murzz | model.input.mur33 | 1 | Relative permeability, zz component | Domain 2 |
| mfnc.Bx | mu0\_const\*mfnc.Ixx\*mfnc.Hx+mu0\_const\*mfnc.Ixy\*mfnc.Hy+mu0\_const\*mfnc.Ixz\*mfnc.Hz+mu0\_const\*mfnc.chimxx\*mfnc.Hx+mu0\_const\*mfnc.chimxy\*mfnc.Hy+mu0\_const\*mfnc.chimxz\*mfnc.Hz+mfnc.Brx | T | Magnetic flux density, x component | Domain 2 |
| mfnc.By | mu0\_const\*mfnc.Iyx\*mfnc.Hx+mu0\_const\*mfnc.Iyy\*mfnc.Hy+mu0\_const\*mfnc.Iyz\*mfnc.Hz+mu0\_const\*mfnc.chimyx\*mfnc.Hx+mu0\_const\*mfnc.chimyy\*mfnc.Hy+mu0\_const\*mfnc.chimyz\*mfnc.Hz+mfnc.Bry | T | Magnetic flux density, y component | Domain 2 |
| mfnc.Bz | mu0\_const\*mfnc.Izx\*mfnc.Hx+mu0\_const\*mfnc.Izy\*mfnc.Hy+mu0\_const\*mfnc.Izz\*mfnc.Hz+mu0\_const\*mfnc.chimzx\*mfnc.Hx+mu0\_const\*mfnc.chimzy\*mfnc.Hy+mu0\_const\*mfnc.chimzz\*mfnc.Hz+mfnc.Brz | T | Magnetic flux density, z component | Domain 2 |
| mfnc.normB | sqrt(realdot(mfnc.Bx,mfnc.Bx)+realdot(mfnc.By,mfnc.By)+realdot(mfnc.Bz,mfnc.Bz)) | T | Magnetic flux density norm | Domain 2 |
| mfnc.Mx | mfnc.Bx/mu0\_const-mfnc.Ixx\*mfnc.Hx-mfnc.Ixy\*mfnc.Hy-mfnc.Ixz\*mfnc.Hz | A/m | Magnetization, x component | Domain 2 |
| mfnc.My | mfnc.By/mu0\_const-mfnc.Iyx\*mfnc.Hx-mfnc.Iyy\*mfnc.Hy-mfnc.Iyz\*mfnc.Hz | A/m | Magnetization, y component | Domain 2 |
| mfnc.Mz | mfnc.Bz/mu0\_const-mfnc.Izx\*mfnc.Hx-mfnc.Izy\*mfnc.Hy-mfnc.Izz\*mfnc.Hz | A/m | Magnetization, z component | Domain 2 |
| mfnc.normM | sqrt(realdot(mfnc.Mx,mfnc.Mx)+realdot(mfnc.My,mfnc.My)+realdot(mfnc.Mz,mfnc.Mz)) | A/m | Magnetization norm | Domain 2 |
| mfnc.Ixx | 1 | 1 | Spatial identity matrix, xx component | Domain 2 |
| mfnc.Iyx | 0 | 1 | Spatial identity matrix, yx component | Domain 2 |
| mfnc.Izx | 0 | 1 | Spatial identity matrix, zx component | Domain 2 |
| mfnc.Ixy | 0 | 1 | Spatial identity matrix, xy component | Domain 2 |
| mfnc.Iyy | 1 | 1 | Spatial identity matrix, yy component | Domain 2 |
| mfnc.Izy | 0 | 1 | Spatial identity matrix, zy component | Domain 2 |
| mfnc.Ixz | 0 | 1 | Spatial identity matrix, xz component | Domain 2 |
| mfnc.Iyz | 0 | 1 | Spatial identity matrix, yz component | Domain 2 |
| mfnc.Izz | 1 | 1 | Spatial identity matrix, zz component | Domain 2 |
| mfnc.chimxx | -1+mfnc.murxx | 1 | Magnetic susceptibility, xx component | Domain 2 |
| mfnc.chimyx | mfnc.muryx | 1 | Magnetic susceptibility, yx component | Domain 2 |
| mfnc.chimzx | mfnc.murzx | 1 | Magnetic susceptibility, zx component | Domain 2 |
| mfnc.chimxy | mfnc.murxy | 1 | Magnetic susceptibility, xy component | Domain 2 |
| mfnc.chimyy | -1+mfnc.muryy | 1 | Magnetic susceptibility, yy component | Domain 2 |
| mfnc.chimzy | mfnc.murzy | 1 | Magnetic susceptibility, zy component | Domain 2 |
| mfnc.chimxz | mfnc.murxz | 1 | Magnetic susceptibility, xz component | Domain 2 |
| mfnc.chimyz | mfnc.muryz | 1 | Magnetic susceptibility, yz component | Domain 2 |
| mfnc.chimzz | -1+mfnc.murzz | 1 | Magnetic susceptibility, zz component | Domain 2 |
| mfnc.Brx | BA\*cos(2\*pi) | T | Remanent flux density, x component | Domain 2 |
| mfnc.Bry | BA\*sin(2\*pi) | T | Remanent flux density, y component | Domain 2 |
| mfnc.Brz | 0 | T | Remanent flux density, z component | Domain 2 |
| mfnc.normBr | sqrt(realdot(mfnc.Brx,mfnc.Brx)+realdot(mfnc.Bry,mfnc.Bry)+realdot(mfnc.Brz,mfnc.Brz)) | T | Remanent flux density norm | Domain 2 |
| mfnc.unTx | 0 | Pa | Maxwell upward surface stress tensor, x component | Boundaries 6–12, 23–25 |
| mfnc.unTy | 0 | Pa | Maxwell upward surface stress tensor, y component | Boundaries 6–12, 23–25 |
| mfnc.unTz | 0 | Pa | Maxwell upward surface stress tensor, z component | Boundaries 6–12, 23–25 |
| mfnc.dnTx | mfnc.dnTmx | Pa | Maxwell downward surface stress tensor, x component | Boundaries 6–12, 23–25 |
| mfnc.dnTy | mfnc.dnTmy | Pa | Maxwell downward surface stress tensor, y component | Boundaries 6–12, 23–25 |
| mfnc.dnTz | mfnc.dnTmz | Pa | Maxwell downward surface stress tensor, z component | Boundaries 6–12, 23–25 |
| mfnc.unx | unx |  | Normal vector up direction, x component | Boundaries 6–12, 23–25 |
| mfnc.uny | uny |  | Normal vector up direction, y component | Boundaries 6–12, 23–25 |
| mfnc.unz | unz |  | Normal vector up direction, z component | Boundaries 6–12, 23–25 |
| mfnc.dnx | dnx |  | Normal vector down direction, x component | Boundaries 6–12, 23–25 |
| mfnc.dny | dny |  | Normal vector down direction, y component | Boundaries 6–12, 23–25 |
| mfnc.dnz | dnz |  | Normal vector down direction, z component | Boundaries 6–12, 23–25 |
| mfnc.W | mfnc.Wm | J/m^3 | Energy density | Domain 2 |
| mfnc.dWm | mfnc.Wm | J/m^3 | Integrand for total magnetic energy | Domain 2 |
| mfnc.Wm | 0.5\*mu0\_const\*((mfnc.murxx\*mfnc.Hx+mfnc.murxy\*mfnc.Hy+mfnc.murxz\*mfnc.Hz)\*mfnc.Hx+(mfnc.muryx\*mfnc.Hx+mfnc.muryy\*mfnc.Hy+mfnc.muryz\*mfnc.Hz)\*mfnc.Hy+(mfnc.murzx\*mfnc.Hx+mfnc.murzy\*mfnc.Hy+mfnc.murzz\*mfnc.Hz)\*mfnc.Hz) | J/m^3 | Magnetic energy density | Domain 2 |

##### Shape Functions

| Name | Shape function | Unit | Description | Shape frame | Selection |
| --- | --- | --- | --- | --- | --- |
| Vm | Lagrange (Quadratic) | A | Magnetic scalar potential | Material | Domain 2 |

##### Weak Expressions

| Weak expression | Integration frame | Selection |
| --- | --- | --- |
| mfnc.d\*(-mfnc.Bx\*test(Vmx)-mfnc.By\*test(Vmy)-mfnc.Bz\*test(Vmz)) | Material | Domain 2 |

#### 2.4.29. ArrayAMagnet8

ArrayAMagnet8

Selection

| Geometric entity level | Domain |
| Selection | Domain 3 |

Equations

Settings

| Description | Value |
| Constitutive relation | Remanent flux density |
| Remanent flux density, x component | BA\*cos(2\*pi\*(7\*60)/360) |
| Remanent flux density, y component | BA\*sin(2\*pi\*(7\*60)/360) |
| Remanent flux density, z component | 0 |
| Relative permeability | From material |
| Relative permeability | {{1, 0, 0}, {0, 1, 0}, {0, 0, 1}} |

Properties from material

| Property | Material | Property group |
| Relative permeability | Soft Iron (without losses) | Basic |

##### Variables

| Name | Expression | Unit | Description | Selection |
| --- | --- | --- | --- | --- |
| mfnc.dnTmx | -0.5\*mfnc.unx\*(real(down(mfnc.Bx))\*real(down(mfnc.Hx))+real(down(mfnc.By))\*real(down(mfnc.Hy))+real(down(mfnc.Bz))\*real(down(mfnc.Hz)))+real(down(mfnc.Bx))\*(real(down(mfnc.Hx))\*mfnc.unx+real(down(mfnc.Hy))\*mfnc.uny+real(down(mfnc.Hz))\*mfnc.unz) | Pa | Maxwell downward magnetic surface stress tensor, x component | Boundaries 13–16, 21, 26, 28, 30, 32, 34 |
| mfnc.dnTmy | -0.5\*mfnc.uny\*(real(down(mfnc.Bx))\*real(down(mfnc.Hx))+real(down(mfnc.By))\*real(down(mfnc.Hy))+real(down(mfnc.Bz))\*real(down(mfnc.Hz)))+real(down(mfnc.By))\*(real(down(mfnc.Hx))\*mfnc.unx+real(down(mfnc.Hy))\*mfnc.uny+real(down(mfnc.Hz))\*mfnc.unz) | Pa | Maxwell downward magnetic surface stress tensor, y component | Boundaries 13–16, 21, 26, 28, 30, 32, 34 |
| mfnc.dnTmz | -0.5\*mfnc.unz\*(real(down(mfnc.Bx))\*real(down(mfnc.Hx))+real(down(mfnc.By))\*real(down(mfnc.Hy))+real(down(mfnc.Bz))\*real(down(mfnc.Hz)))+real(down(mfnc.Bz))\*(real(down(mfnc.Hx))\*mfnc.unx+real(down(mfnc.Hy))\*mfnc.uny+real(down(mfnc.Hz))\*mfnc.unz) | Pa | Maxwell downward magnetic surface stress tensor, z component | Boundaries 13–16, 21, 26, 28, 30, 32, 34 |
| mfnc.Hx | -Vmx | A/m | Magnetic field, x component | Domain 3 |
| mfnc.Hy | -Vmy | A/m | Magnetic field, y component | Domain 3 |
| mfnc.Hz | -Vmz | A/m | Magnetic field, z component | Domain 3 |
| mfnc.tHx | -VmTx | A/m | Tangential magnetic field, x component | Boundaries 13–16, 21, 26, 28, 30, 32, 34 |
| mfnc.tHy | -VmTy | A/m | Tangential magnetic field, y component | Boundaries 13–16, 21, 26, 28, 30, 32, 34 |
| mfnc.tHz | -VmTz | A/m | Tangential magnetic field, z component | Boundaries 13–16, 21, 26, 28, 30, 32, 34 |
| mfnc.normH | sqrt(realdot(mfnc.Hx,mfnc.Hx)+realdot(mfnc.Hy,mfnc.Hy)+realdot(mfnc.Hz,mfnc.Hz)) | A/m | Magnetic field norm | Domain 3 |
| mfnc.murxx | model.input.mur11 | 1 | Relative permeability, xx component | Domain 3 |
| mfnc.muryx | model.input.mur21 | 1 | Relative permeability, yx component | Domain 3 |
| mfnc.murzx | model.input.mur31 | 1 | Relative permeability, zx component | Domain 3 |
| mfnc.murxy | model.input.mur12 | 1 | Relative permeability, xy component | Domain 3 |
| mfnc.muryy | model.input.mur22 | 1 | Relative permeability, yy component | Domain 3 |
| mfnc.murzy | model.input.mur32 | 1 | Relative permeability, zy component | Domain 3 |
| mfnc.murxz | model.input.mur13 | 1 | Relative permeability, xz component | Domain 3 |
| mfnc.muryz | model.input.mur23 | 1 | Relative permeability, yz component | Domain 3 |
| mfnc.murzz | model.input.mur33 | 1 | Relative permeability, zz component | Domain 3 |
| mfnc.Bx | mu0\_const\*mfnc.Ixx\*mfnc.Hx+mu0\_const\*mfnc.Ixy\*mfnc.Hy+mu0\_const\*mfnc.Ixz\*mfnc.Hz+mu0\_const\*mfnc.chimxx\*mfnc.Hx+mu0\_const\*mfnc.chimxy\*mfnc.Hy+mu0\_const\*mfnc.chimxz\*mfnc.Hz+mfnc.Brx | T | Magnetic flux density, x component | Domain 3 |
| mfnc.By | mu0\_const\*mfnc.Iyx\*mfnc.Hx+mu0\_const\*mfnc.Iyy\*mfnc.Hy+mu0\_const\*mfnc.Iyz\*mfnc.Hz+mu0\_const\*mfnc.chimyx\*mfnc.Hx+mu0\_const\*mfnc.chimyy\*mfnc.Hy+mu0\_const\*mfnc.chimyz\*mfnc.Hz+mfnc.Bry | T | Magnetic flux density, y component | Domain 3 |
| mfnc.Bz | mu0\_const\*mfnc.Izx\*mfnc.Hx+mu0\_const\*mfnc.Izy\*mfnc.Hy+mu0\_const\*mfnc.Izz\*mfnc.Hz+mu0\_const\*mfnc.chimzx\*mfnc.Hx+mu0\_const\*mfnc.chimzy\*mfnc.Hy+mu0\_const\*mfnc.chimzz\*mfnc.Hz+mfnc.Brz | T | Magnetic flux density, z component | Domain 3 |
| mfnc.normB | sqrt(realdot(mfnc.Bx,mfnc.Bx)+realdot(mfnc.By,mfnc.By)+realdot(mfnc.Bz,mfnc.Bz)) | T | Magnetic flux density norm | Domain 3 |
| mfnc.Mx | mfnc.Bx/mu0\_const-mfnc.Ixx\*mfnc.Hx-mfnc.Ixy\*mfnc.Hy-mfnc.Ixz\*mfnc.Hz | A/m | Magnetization, x component | Domain 3 |
| mfnc.My | mfnc.By/mu0\_const-mfnc.Iyx\*mfnc.Hx-mfnc.Iyy\*mfnc.Hy-mfnc.Iyz\*mfnc.Hz | A/m | Magnetization, y component | Domain 3 |
| mfnc.Mz | mfnc.Bz/mu0\_const-mfnc.Izx\*mfnc.Hx-mfnc.Izy\*mfnc.Hy-mfnc.Izz\*mfnc.Hz | A/m | Magnetization, z component | Domain 3 |
| mfnc.normM | sqrt(realdot(mfnc.Mx,mfnc.Mx)+realdot(mfnc.My,mfnc.My)+realdot(mfnc.Mz,mfnc.Mz)) | A/m | Magnetization norm | Domain 3 |
| mfnc.Ixx | 1 | 1 | Spatial identity matrix, xx component | Domain 3 |
| mfnc.Iyx | 0 | 1 | Spatial identity matrix, yx component | Domain 3 |
| mfnc.Izx | 0 | 1 | Spatial identity matrix, zx component | Domain 3 |
| mfnc.Ixy | 0 | 1 | Spatial identity matrix, xy component | Domain 3 |
| mfnc.Iyy | 1 | 1 | Spatial identity matrix, yy component | Domain 3 |
| mfnc.Izy | 0 | 1 | Spatial identity matrix, zy component | Domain 3 |
| mfnc.Ixz | 0 | 1 | Spatial identity matrix, xz component | Domain 3 |
| mfnc.Iyz | 0 | 1 | Spatial identity matrix, yz component | Domain 3 |
| mfnc.Izz | 1 | 1 | Spatial identity matrix, zz component | Domain 3 |
| mfnc.chimxx | -1+mfnc.murxx | 1 | Magnetic susceptibility, xx component | Domain 3 |
| mfnc.chimyx | mfnc.muryx | 1 | Magnetic susceptibility, yx component | Domain 3 |
| mfnc.chimzx | mfnc.murzx | 1 | Magnetic susceptibility, zx component | Domain 3 |
| mfnc.chimxy | mfnc.murxy | 1 | Magnetic susceptibility, xy component | Domain 3 |
| mfnc.chimyy | -1+mfnc.muryy | 1 | Magnetic susceptibility, yy component | Domain 3 |
| mfnc.chimzy | mfnc.murzy | 1 | Magnetic susceptibility, zy component | Domain 3 |
| mfnc.chimxz | mfnc.murxz | 1 | Magnetic susceptibility, xz component | Domain 3 |
| mfnc.chimyz | mfnc.muryz | 1 | Magnetic susceptibility, yz component | Domain 3 |
| mfnc.chimzz | -1+mfnc.murzz | 1 | Magnetic susceptibility, zz component | Domain 3 |
| mfnc.Brx | BA\*cos(7\*pi/3) | T | Remanent flux density, x component | Domain 3 |
| mfnc.Bry | BA\*sin(7\*pi/3) | T | Remanent flux density, y component | Domain 3 |
| mfnc.Brz | 0 | T | Remanent flux density, z component | Domain 3 |
| mfnc.normBr | sqrt(realdot(mfnc.Brx,mfnc.Brx)+realdot(mfnc.Bry,mfnc.Bry)+realdot(mfnc.Brz,mfnc.Brz)) | T | Remanent flux density norm | Domain 3 |
| mfnc.unTx | 0 | Pa | Maxwell upward surface stress tensor, x component | Boundaries 13–16, 21, 26, 28, 30, 32, 34 |
| mfnc.unTy | 0 | Pa | Maxwell upward surface stress tensor, y component | Boundaries 13–16, 21, 26, 28, 30, 32, 34 |
| mfnc.unTz | 0 | Pa | Maxwell upward surface stress tensor, z component | Boundaries 13–16, 21, 26, 28, 30, 32, 34 |
| mfnc.dnTx | mfnc.dnTmx | Pa | Maxwell downward surface stress tensor, x component | Boundaries 13–16, 21, 26, 28, 30, 32, 34 |
| mfnc.dnTy | mfnc.dnTmy | Pa | Maxwell downward surface stress tensor, y component | Boundaries 13–16, 21, 26, 28, 30, 32, 34 |
| mfnc.dnTz | mfnc.dnTmz | Pa | Maxwell downward surface stress tensor, z component | Boundaries 13–16, 21, 26, 28, 30, 32, 34 |
| mfnc.unx | unx |  | Normal vector up direction, x component | Boundaries 13–16, 21, 26, 28, 30, 32, 34 |
| mfnc.uny | uny |  | Normal vector up direction, y component | Boundaries 13–16, 21, 26, 28, 30, 32, 34 |
| mfnc.unz | unz |  | Normal vector up direction, z component | Boundaries 13–16, 21, 26, 28, 30, 32, 34 |
| mfnc.dnx | dnx |  | Normal vector down direction, x component | Boundaries 13–16, 21, 26, 28, 30, 32, 34 |
| mfnc.dny | dny |  | Normal vector down direction, y component | Boundaries 13–16, 21, 26, 28, 30, 32, 34 |
| mfnc.dnz | dnz |  | Normal vector down direction, z component | Boundaries 13–16, 21, 26, 28, 30, 32, 34 |
| mfnc.W | mfnc.Wm | J/m^3 | Energy density | Domain 3 |
| mfnc.dWm | mfnc.Wm | J/m^3 | Integrand for total magnetic energy | Domain 3 |
| mfnc.Wm | 0.5\*mu0\_const\*((mfnc.murxx\*mfnc.Hx+mfnc.murxy\*mfnc.Hy+mfnc.murxz\*mfnc.Hz)\*mfnc.Hx+(mfnc.muryx\*mfnc.Hx+mfnc.muryy\*mfnc.Hy+mfnc.muryz\*mfnc.Hz)\*mfnc.Hy+(mfnc.murzx\*mfnc.Hx+mfnc.murzy\*mfnc.Hy+mfnc.murzz\*mfnc.Hz)\*mfnc.Hz) | J/m^3 | Magnetic energy density | Domain 3 |

##### Shape Functions

| Name | Shape function | Unit | Description | Shape frame | Selection |
| --- | --- | --- | --- | --- | --- |
| Vm | Lagrange (Quadratic) | A | Magnetic scalar potential | Material | Domain 3 |

##### Weak Expressions

| Weak expression | Integration frame | Selection |
| --- | --- | --- |
| mfnc.d\*(-mfnc.Bx\*test(Vmx)-mfnc.By\*test(Vmy)-mfnc.Bz\*test(Vmz)) | Material | Domain 3 |

#### 2.4.30. ArrayAMagnet9

ArrayAMagnet9

Selection

| Geometric entity level | Domain |
| Selection | Domain 8 |

Equations

Settings

| Description | Value |
| Constitutive relation | Remanent flux density |
| Remanent flux density, x component | BA\*cos(2\*pi\*(8\*60)/360) |
| Remanent flux density, y component | BA\*sin(2\*pi\*(8\*60)/360) |
| Remanent flux density, z component | 0 |
| Relative permeability | From material |
| Relative permeability | {{1, 0, 0}, {0, 1, 0}, {0, 0, 1}} |

Properties from material

| Property | Material | Property group |
| Relative permeability | Soft Iron (without losses) | Basic |

##### Variables

| Name | Expression | Unit | Description | Selection |
| --- | --- | --- | --- | --- |
| mfnc.dnTmx | -0.5\*mfnc.unx\*(real(down(mfnc.Bx))\*real(down(mfnc.Hx))+real(down(mfnc.By))\*real(down(mfnc.Hy))+real(down(mfnc.Bz))\*real(down(mfnc.Hz)))+real(down(mfnc.Bx))\*(real(down(mfnc.Hx))\*mfnc.unx+real(down(mfnc.Hy))\*mfnc.uny+real(down(mfnc.Hz))\*mfnc.unz) | Pa | Maxwell downward magnetic surface stress tensor, x component | Boundaries 60–63, 68, 76, 78, 90, 92, 104 |
| mfnc.dnTmy | -0.5\*mfnc.uny\*(real(down(mfnc.Bx))\*real(down(mfnc.Hx))+real(down(mfnc.By))\*real(down(mfnc.Hy))+real(down(mfnc.Bz))\*real(down(mfnc.Hz)))+real(down(mfnc.By))\*(real(down(mfnc.Hx))\*mfnc.unx+real(down(mfnc.Hy))\*mfnc.uny+real(down(mfnc.Hz))\*mfnc.unz) | Pa | Maxwell downward magnetic surface stress tensor, y component | Boundaries 60–63, 68, 76, 78, 90, 92, 104 |
| mfnc.dnTmz | -0.5\*mfnc.unz\*(real(down(mfnc.Bx))\*real(down(mfnc.Hx))+real(down(mfnc.By))\*real(down(mfnc.Hy))+real(down(mfnc.Bz))\*real(down(mfnc.Hz)))+real(down(mfnc.Bz))\*(real(down(mfnc.Hx))\*mfnc.unx+real(down(mfnc.Hy))\*mfnc.uny+real(down(mfnc.Hz))\*mfnc.unz) | Pa | Maxwell downward magnetic surface stress tensor, z component | Boundaries 60–63, 68, 76, 78, 90, 92, 104 |
| mfnc.Hx | -Vmx | A/m | Magnetic field, x component | Domain 8 |
| mfnc.Hy | -Vmy | A/m | Magnetic field, y component | Domain 8 |
| mfnc.Hz | -Vmz | A/m | Magnetic field, z component | Domain 8 |
| mfnc.tHx | -VmTx | A/m | Tangential magnetic field, x component | Boundaries 60–63, 68, 76, 78, 90, 92, 104 |
| mfnc.tHy | -VmTy | A/m | Tangential magnetic field, y component | Boundaries 60–63, 68, 76, 78, 90, 92, 104 |
| mfnc.tHz | -VmTz | A/m | Tangential magnetic field, z component | Boundaries 60–63, 68, 76, 78, 90, 92, 104 |
| mfnc.normH | sqrt(realdot(mfnc.Hx,mfnc.Hx)+realdot(mfnc.Hy,mfnc.Hy)+realdot(mfnc.Hz,mfnc.Hz)) | A/m | Magnetic field norm | Domain 8 |
| mfnc.murxx | model.input.mur11 | 1 | Relative permeability, xx component | Domain 8 |
| mfnc.muryx | model.input.mur21 | 1 | Relative permeability, yx component | Domain 8 |
| mfnc.murzx | model.input.mur31 | 1 | Relative permeability, zx component | Domain 8 |
| mfnc.murxy | model.input.mur12 | 1 | Relative permeability, xy component | Domain 8 |
| mfnc.muryy | model.input.mur22 | 1 | Relative permeability, yy component | Domain 8 |
| mfnc.murzy | model.input.mur32 | 1 | Relative permeability, zy component | Domain 8 |
| mfnc.murxz | model.input.mur13 | 1 | Relative permeability, xz component | Domain 8 |
| mfnc.muryz | model.input.mur23 | 1 | Relative permeability, yz component | Domain 8 |
| mfnc.murzz | model.input.mur33 | 1 | Relative permeability, zz component | Domain 8 |
| mfnc.Bx | mu0\_const\*mfnc.Ixx\*mfnc.Hx+mu0\_const\*mfnc.Ixy\*mfnc.Hy+mu0\_const\*mfnc.Ixz\*mfnc.Hz+mu0\_const\*mfnc.chimxx\*mfnc.Hx+mu0\_const\*mfnc.chimxy\*mfnc.Hy+mu0\_const\*mfnc.chimxz\*mfnc.Hz+mfnc.Brx | T | Magnetic flux density, x component | Domain 8 |
| mfnc.By | mu0\_const\*mfnc.Iyx\*mfnc.Hx+mu0\_const\*mfnc.Iyy\*mfnc.Hy+mu0\_const\*mfnc.Iyz\*mfnc.Hz+mu0\_const\*mfnc.chimyx\*mfnc.Hx+mu0\_const\*mfnc.chimyy\*mfnc.Hy+mu0\_const\*mfnc.chimyz\*mfnc.Hz+mfnc.Bry | T | Magnetic flux density, y component | Domain 8 |
| mfnc.Bz | mu0\_const\*mfnc.Izx\*mfnc.Hx+mu0\_const\*mfnc.Izy\*mfnc.Hy+mu0\_const\*mfnc.Izz\*mfnc.Hz+mu0\_const\*mfnc.chimzx\*mfnc.Hx+mu0\_const\*mfnc.chimzy\*mfnc.Hy+mu0\_const\*mfnc.chimzz\*mfnc.Hz+mfnc.Brz | T | Magnetic flux density, z component | Domain 8 |
| mfnc.normB | sqrt(realdot(mfnc.Bx,mfnc.Bx)+realdot(mfnc.By,mfnc.By)+realdot(mfnc.Bz,mfnc.Bz)) | T | Magnetic flux density norm | Domain 8 |
| mfnc.Mx | mfnc.Bx/mu0\_const-mfnc.Ixx\*mfnc.Hx-mfnc.Ixy\*mfnc.Hy-mfnc.Ixz\*mfnc.Hz | A/m | Magnetization, x component | Domain 8 |
| mfnc.My | mfnc.By/mu0\_const-mfnc.Iyx\*mfnc.Hx-mfnc.Iyy\*mfnc.Hy-mfnc.Iyz\*mfnc.Hz | A/m | Magnetization, y component | Domain 8 |
| mfnc.Mz | mfnc.Bz/mu0\_const-mfnc.Izx\*mfnc.Hx-mfnc.Izy\*mfnc.Hy-mfnc.Izz\*mfnc.Hz | A/m | Magnetization, z component | Domain 8 |
| mfnc.normM | sqrt(realdot(mfnc.Mx,mfnc.Mx)+realdot(mfnc.My,mfnc.My)+realdot(mfnc.Mz,mfnc.Mz)) | A/m | Magnetization norm | Domain 8 |
| mfnc.Ixx | 1 | 1 | Spatial identity matrix, xx component | Domain 8 |
| mfnc.Iyx | 0 | 1 | Spatial identity matrix, yx component | Domain 8 |
| mfnc.Izx | 0 | 1 | Spatial identity matrix, zx component | Domain 8 |
| mfnc.Ixy | 0 | 1 | Spatial identity matrix, xy component | Domain 8 |
| mfnc.Iyy | 1 | 1 | Spatial identity matrix, yy component | Domain 8 |
| mfnc.Izy | 0 | 1 | Spatial identity matrix, zy component | Domain 8 |
| mfnc.Ixz | 0 | 1 | Spatial identity matrix, xz component | Domain 8 |
| mfnc.Iyz | 0 | 1 | Spatial identity matrix, yz component | Domain 8 |
| mfnc.Izz | 1 | 1 | Spatial identity matrix, zz component | Domain 8 |
| mfnc.chimxx | -1+mfnc.murxx | 1 | Magnetic susceptibility, xx component | Domain 8 |
| mfnc.chimyx | mfnc.muryx | 1 | Magnetic susceptibility, yx component | Domain 8 |
| mfnc.chimzx | mfnc.murzx | 1 | Magnetic susceptibility, zx component | Domain 8 |
| mfnc.chimxy | mfnc.murxy | 1 | Magnetic susceptibility, xy component | Domain 8 |
| mfnc.chimyy | -1+mfnc.muryy | 1 | Magnetic susceptibility, yy component | Domain 8 |
| mfnc.chimzy | mfnc.murzy | 1 | Magnetic susceptibility, zy component | Domain 8 |
| mfnc.chimxz | mfnc.murxz | 1 | Magnetic susceptibility, xz component | Domain 8 |
| mfnc.chimyz | mfnc.muryz | 1 | Magnetic susceptibility, yz component | Domain 8 |
| mfnc.chimzz | -1+mfnc.murzz | 1 | Magnetic susceptibility, zz component | Domain 8 |
| mfnc.Brx | BA\*cos(8\*pi/3) | T | Remanent flux density, x component | Domain 8 |
| mfnc.Bry | BA\*sin(8\*pi/3) | T | Remanent flux density, y component | Domain 8 |
| mfnc.Brz | 0 | T | Remanent flux density, z component | Domain 8 |
| mfnc.normBr | sqrt(realdot(mfnc.Brx,mfnc.Brx)+realdot(mfnc.Bry,mfnc.Bry)+realdot(mfnc.Brz,mfnc.Brz)) | T | Remanent flux density norm | Domain 8 |
| mfnc.unTx | 0 | Pa | Maxwell upward surface stress tensor, x component | Boundaries 60–63, 68, 76, 78, 90, 92, 104 |
| mfnc.unTy | 0 | Pa | Maxwell upward surface stress tensor, y component | Boundaries 60–63, 68, 76, 78, 90, 92, 104 |
| mfnc.unTz | 0 | Pa | Maxwell upward surface stress tensor, z component | Boundaries 60–63, 68, 76, 78, 90, 92, 104 |
| mfnc.dnTx | mfnc.dnTmx | Pa | Maxwell downward surface stress tensor, x component | Boundaries 60–63, 68, 76, 78, 90, 92, 104 |
| mfnc.dnTy | mfnc.dnTmy | Pa | Maxwell downward surface stress tensor, y component | Boundaries 60–63, 68, 76, 78, 90, 92, 104 |
| mfnc.dnTz | mfnc.dnTmz | Pa | Maxwell downward surface stress tensor, z component | Boundaries 60–63, 68, 76, 78, 90, 92, 104 |
| mfnc.unx | unx |  | Normal vector up direction, x component | Boundaries 60–63, 68, 76, 78, 90, 92, 104 |
| mfnc.uny | uny |  | Normal vector up direction, y component | Boundaries 60–63, 68, 76, 78, 90, 92, 104 |
| mfnc.unz | unz |  | Normal vector up direction, z component | Boundaries 60–63, 68, 76, 78, 90, 92, 104 |
| mfnc.dnx | dnx |  | Normal vector down direction, x component | Boundaries 60–63, 68, 76, 78, 90, 92, 104 |
| mfnc.dny | dny |  | Normal vector down direction, y component | Boundaries 60–63, 68, 76, 78, 90, 92, 104 |
| mfnc.dnz | dnz |  | Normal vector down direction, z component | Boundaries 60–63, 68, 76, 78, 90, 92, 104 |
| mfnc.W | mfnc.Wm | J/m^3 | Energy density | Domain 8 |
| mfnc.dWm | mfnc.Wm | J/m^3 | Integrand for total magnetic energy | Domain 8 |
| mfnc.Wm | 0.5\*mu0\_const\*((mfnc.murxx\*mfnc.Hx+mfnc.murxy\*mfnc.Hy+mfnc.murxz\*mfnc.Hz)\*mfnc.Hx+(mfnc.muryx\*mfnc.Hx+mfnc.muryy\*mfnc.Hy+mfnc.muryz\*mfnc.Hz)\*mfnc.Hy+(mfnc.murzx\*mfnc.Hx+mfnc.murzy\*mfnc.Hy+mfnc.murzz\*mfnc.Hz)\*mfnc.Hz) | J/m^3 | Magnetic energy density | Domain 8 |

##### Shape Functions

| Name | Shape function | Unit | Description | Shape frame | Selection |
| --- | --- | --- | --- | --- | --- |
| Vm | Lagrange (Quadratic) | A | Magnetic scalar potential | Material | Domain 8 |

##### Weak Expressions

| Weak expression | Integration frame | Selection |
| --- | --- | --- |
| mfnc.d\*(-mfnc.Bx\*test(Vmx)-mfnc.By\*test(Vmy)-mfnc.Bz\*test(Vmz)) | Material | Domain 8 |

#### 2.4.31. ArrayAMagnet10

ArrayAMagnet10

Selection

| Geometric entity level | Domain |
| Selection | Domain 16 |

Equations

Settings

| Description | Value |
| Constitutive relation | Remanent flux density |
| Remanent flux density, x component | BA\*cos(2\*pi\*(9\*60)/360) |
| Remanent flux density, y component | BA\*sin(2\*pi\*(9\*60)/360) |
| Remanent flux density, z component | 0 |
| Relative permeability | From material |
| Relative permeability | {{1, 0, 0}, {0, 1, 0}, {0, 0, 1}} |

Properties from material

| Property | Material | Property group |
| Relative permeability | Soft Iron (without losses) | Basic |

##### Variables

| Name | Expression | Unit | Description | Selection |
| --- | --- | --- | --- | --- |
| mfnc.dnTmx | -0.5\*mfnc.unx\*(real(down(mfnc.Bx))\*real(down(mfnc.Hx))+real(down(mfnc.By))\*real(down(mfnc.Hy))+real(down(mfnc.Bz))\*real(down(mfnc.Hz)))+real(down(mfnc.Bx))\*(real(down(mfnc.Hx))\*mfnc.unx+real(down(mfnc.Hy))\*mfnc.uny+real(down(mfnc.Hz))\*mfnc.unz) | Pa | Maxwell downward magnetic surface stress tensor, x component | Boundaries 140–144, 152–153, 176–177, 181 |
| mfnc.dnTmy | -0.5\*mfnc.uny\*(real(down(mfnc.Bx))\*real(down(mfnc.Hx))+real(down(mfnc.By))\*real(down(mfnc.Hy))+real(down(mfnc.Bz))\*real(down(mfnc.Hz)))+real(down(mfnc.By))\*(real(down(mfnc.Hx))\*mfnc.unx+real(down(mfnc.Hy))\*mfnc.uny+real(down(mfnc.Hz))\*mfnc.unz) | Pa | Maxwell downward magnetic surface stress tensor, y component | Boundaries 140–144, 152–153, 176–177, 181 |
| mfnc.dnTmz | -0.5\*mfnc.unz\*(real(down(mfnc.Bx))\*real(down(mfnc.Hx))+real(down(mfnc.By))\*real(down(mfnc.Hy))+real(down(mfnc.Bz))\*real(down(mfnc.Hz)))+real(down(mfnc.Bz))\*(real(down(mfnc.Hx))\*mfnc.unx+real(down(mfnc.Hy))\*mfnc.uny+real(down(mfnc.Hz))\*mfnc.unz) | Pa | Maxwell downward magnetic surface stress tensor, z component | Boundaries 140–144, 152–153, 176–177, 181 |
| mfnc.Hx | -Vmx | A/m | Magnetic field, x component | Domain 16 |
| mfnc.Hy | -Vmy | A/m | Magnetic field, y component | Domain 16 |
| mfnc.Hz | -Vmz | A/m | Magnetic field, z component | Domain 16 |
| mfnc.tHx | -VmTx | A/m | Tangential magnetic field, x component | Boundaries 140–144, 152–153, 176–177, 181 |
| mfnc.tHy | -VmTy | A/m | Tangential magnetic field, y component | Boundaries 140–144, 152–153, 176–177, 181 |
| mfnc.tHz | -VmTz | A/m | Tangential magnetic field, z component | Boundaries 140–144, 152–153, 176–177, 181 |
| mfnc.normH | sqrt(realdot(mfnc.Hx,mfnc.Hx)+realdot(mfnc.Hy,mfnc.Hy)+realdot(mfnc.Hz,mfnc.Hz)) | A/m | Magnetic field norm | Domain 16 |
| mfnc.murxx | model.input.mur11 | 1 | Relative permeability, xx component | Domain 16 |
| mfnc.muryx | model.input.mur21 | 1 | Relative permeability, yx component | Domain 16 |
| mfnc.murzx | model.input.mur31 | 1 | Relative permeability, zx component | Domain 16 |
| mfnc.murxy | model.input.mur12 | 1 | Relative permeability, xy component | Domain 16 |
| mfnc.muryy | model.input.mur22 | 1 | Relative permeability, yy component | Domain 16 |
| mfnc.murzy | model.input.mur32 | 1 | Relative permeability, zy component | Domain 16 |
| mfnc.murxz | model.input.mur13 | 1 | Relative permeability, xz component | Domain 16 |
| mfnc.muryz | model.input.mur23 | 1 | Relative permeability, yz component | Domain 16 |
| mfnc.murzz | model.input.mur33 | 1 | Relative permeability, zz component | Domain 16 |
| mfnc.Bx | mu0\_const\*mfnc.Ixx\*mfnc.Hx+mu0\_const\*mfnc.Ixy\*mfnc.Hy+mu0\_const\*mfnc.Ixz\*mfnc.Hz+mu0\_const\*mfnc.chimxx\*mfnc.Hx+mu0\_const\*mfnc.chimxy\*mfnc.Hy+mu0\_const\*mfnc.chimxz\*mfnc.Hz+mfnc.Brx | T | Magnetic flux density, x component | Domain 16 |
| mfnc.By | mu0\_const\*mfnc.Iyx\*mfnc.Hx+mu0\_const\*mfnc.Iyy\*mfnc.Hy+mu0\_const\*mfnc.Iyz\*mfnc.Hz+mu0\_const\*mfnc.chimyx\*mfnc.Hx+mu0\_const\*mfnc.chimyy\*mfnc.Hy+mu0\_const\*mfnc.chimyz\*mfnc.Hz+mfnc.Bry | T | Magnetic flux density, y component | Domain 16 |
| mfnc.Bz | mu0\_const\*mfnc.Izx\*mfnc.Hx+mu0\_const\*mfnc.Izy\*mfnc.Hy+mu0\_const\*mfnc.Izz\*mfnc.Hz+mu0\_const\*mfnc.chimzx\*mfnc.Hx+mu0\_const\*mfnc.chimzy\*mfnc.Hy+mu0\_const\*mfnc.chimzz\*mfnc.Hz+mfnc.Brz | T | Magnetic flux density, z component | Domain 16 |
| mfnc.normB | sqrt(realdot(mfnc.Bx,mfnc.Bx)+realdot(mfnc.By,mfnc.By)+realdot(mfnc.Bz,mfnc.Bz)) | T | Magnetic flux density norm | Domain 16 |
| mfnc.Mx | mfnc.Bx/mu0\_const-mfnc.Ixx\*mfnc.Hx-mfnc.Ixy\*mfnc.Hy-mfnc.Ixz\*mfnc.Hz | A/m | Magnetization, x component | Domain 16 |
| mfnc.My | mfnc.By/mu0\_const-mfnc.Iyx\*mfnc.Hx-mfnc.Iyy\*mfnc.Hy-mfnc.Iyz\*mfnc.Hz | A/m | Magnetization, y component | Domain 16 |
| mfnc.Mz | mfnc.Bz/mu0\_const-mfnc.Izx\*mfnc.Hx-mfnc.Izy\*mfnc.Hy-mfnc.Izz\*mfnc.Hz | A/m | Magnetization, z component | Domain 16 |
| mfnc.normM | sqrt(realdot(mfnc.Mx,mfnc.Mx)+realdot(mfnc.My,mfnc.My)+realdot(mfnc.Mz,mfnc.Mz)) | A/m | Magnetization norm | Domain 16 |
| mfnc.Ixx | 1 | 1 | Spatial identity matrix, xx component | Domain 16 |
| mfnc.Iyx | 0 | 1 | Spatial identity matrix, yx component | Domain 16 |
| mfnc.Izx | 0 | 1 | Spatial identity matrix, zx component | Domain 16 |
| mfnc.Ixy | 0 | 1 | Spatial identity matrix, xy component | Domain 16 |
| mfnc.Iyy | 1 | 1 | Spatial identity matrix, yy component | Domain 16 |
| mfnc.Izy | 0 | 1 | Spatial identity matrix, zy component | Domain 16 |
| mfnc.Ixz | 0 | 1 | Spatial identity matrix, xz component | Domain 16 |
| mfnc.Iyz | 0 | 1 | Spatial identity matrix, yz component | Domain 16 |
| mfnc.Izz | 1 | 1 | Spatial identity matrix, zz component | Domain 16 |
| mfnc.chimxx | -1+mfnc.murxx | 1 | Magnetic susceptibility, xx component | Domain 16 |
| mfnc.chimyx | mfnc.muryx | 1 | Magnetic susceptibility, yx component | Domain 16 |
| mfnc.chimzx | mfnc.murzx | 1 | Magnetic susceptibility, zx component | Domain 16 |
| mfnc.chimxy | mfnc.murxy | 1 | Magnetic susceptibility, xy component | Domain 16 |
| mfnc.chimyy | -1+mfnc.muryy | 1 | Magnetic susceptibility, yy component | Domain 16 |
| mfnc.chimzy | mfnc.murzy | 1 | Magnetic susceptibility, zy component | Domain 16 |
| mfnc.chimxz | mfnc.murxz | 1 | Magnetic susceptibility, xz component | Domain 16 |
| mfnc.chimyz | mfnc.muryz | 1 | Magnetic susceptibility, yz component | Domain 16 |
| mfnc.chimzz | -1+mfnc.murzz | 1 | Magnetic susceptibility, zz component | Domain 16 |
| mfnc.Brx | BA\*cos(3\*pi) | T | Remanent flux density, x component | Domain 16 |
| mfnc.Bry | BA\*sin(3\*pi) | T | Remanent flux density, y component | Domain 16 |
| mfnc.Brz | 0 | T | Remanent flux density, z component | Domain 16 |
| mfnc.normBr | sqrt(realdot(mfnc.Brx,mfnc.Brx)+realdot(mfnc.Bry,mfnc.Bry)+realdot(mfnc.Brz,mfnc.Brz)) | T | Remanent flux density norm | Domain 16 |
| mfnc.unTx | 0 | Pa | Maxwell upward surface stress tensor, x component | Boundaries 140–144, 152–153, 176–177, 181 |
| mfnc.unTy | 0 | Pa | Maxwell upward surface stress tensor, y component | Boundaries 140–144, 152–153, 176–177, 181 |
| mfnc.unTz | 0 | Pa | Maxwell upward surface stress tensor, z component | Boundaries 140–144, 152–153, 176–177, 181 |
| mfnc.dnTx | mfnc.dnTmx | Pa | Maxwell downward surface stress tensor, x component | Boundaries 140–144, 152–153, 176–177, 181 |
| mfnc.dnTy | mfnc.dnTmy | Pa | Maxwell downward surface stress tensor, y component | Boundaries 140–144, 152–153, 176–177, 181 |
| mfnc.dnTz | mfnc.dnTmz | Pa | Maxwell downward surface stress tensor, z component | Boundaries 140–144, 152–153, 176–177, 181 |
| mfnc.unx | unx |  | Normal vector up direction, x component | Boundaries 140–144, 152–153, 176–177, 181 |
| mfnc.uny | uny |  | Normal vector up direction, y component | Boundaries 140–144, 152–153, 176–177, 181 |
| mfnc.unz | unz |  | Normal vector up direction, z component | Boundaries 140–144, 152–153, 176–177, 181 |
| mfnc.dnx | dnx |  | Normal vector down direction, x component | Boundaries 140–144, 152–153, 176–177, 181 |
| mfnc.dny | dny |  | Normal vector down direction, y component | Boundaries 140–144, 152–153, 176–177, 181 |
| mfnc.dnz | dnz |  | Normal vector down direction, z component | Boundaries 140–144, 152–153, 176–177, 181 |
| mfnc.W | mfnc.Wm | J/m^3 | Energy density | Domain 16 |
| mfnc.dWm | mfnc.Wm | J/m^3 | Integrand for total magnetic energy | Domain 16 |
| mfnc.Wm | 0.5\*mu0\_const\*((mfnc.murxx\*mfnc.Hx+mfnc.murxy\*mfnc.Hy+mfnc.murxz\*mfnc.Hz)\*mfnc.Hx+(mfnc.muryx\*mfnc.Hx+mfnc.muryy\*mfnc.Hy+mfnc.muryz\*mfnc.Hz)\*mfnc.Hy+(mfnc.murzx\*mfnc.Hx+mfnc.murzy\*mfnc.Hy+mfnc.murzz\*mfnc.Hz)\*mfnc.Hz) | J/m^3 | Magnetic energy density | Domain 16 |

##### Shape Functions

| Name | Shape function | Unit | Description | Shape frame | Selection |
| --- | --- | --- | --- | --- | --- |
| Vm | Lagrange (Quadratic) | A | Magnetic scalar potential | Material | Domain 16 |

##### Weak Expressions

| Weak expression | Integration frame | Selection |
| --- | --- | --- |
| mfnc.d\*(-mfnc.Bx\*test(Vmx)-mfnc.By\*test(Vmy)-mfnc.Bz\*test(Vmz)) | Material | Domain 16 |

#### 2.4.32. ArrayAMagnet11

ArrayAMagnet11

Selection

| Geometric entity level | Domain |
| Selection | Domain 24 |

Equations

Settings

| Description | Value |
| Constitutive relation | Remanent flux density |
| Remanent flux density, x component | BA\*cos(2\*pi\*(10\*60)/360) |
| Remanent flux density, y component | BA\*sin(2\*pi\*(10\*60)/360) |
| Remanent flux density, z component | 0 |
| Relative permeability | From material |
| Relative permeability | {{1, 0, 0}, {0, 1, 0}, {0, 0, 1}} |

Properties from material

| Property | Material | Property group |
| Relative permeability | Soft Iron (without losses) | Basic |

##### Variables

| Name | Expression | Unit | Description | Selection |
| --- | --- | --- | --- | --- |
| mfnc.dnTmx | -0.5\*mfnc.unx\*(real(down(mfnc.Bx))\*real(down(mfnc.Hx))+real(down(mfnc.By))\*real(down(mfnc.Hy))+real(down(mfnc.Bz))\*real(down(mfnc.Hz)))+real(down(mfnc.Bx))\*(real(down(mfnc.Hx))\*mfnc.unx+real(down(mfnc.Hy))\*mfnc.uny+real(down(mfnc.Hz))\*mfnc.unz) | Pa | Maxwell downward magnetic surface stress tensor, x component | Boundaries 220–223, 228, 232, 234, 246, 248, 264 |
| mfnc.dnTmy | -0.5\*mfnc.uny\*(real(down(mfnc.Bx))\*real(down(mfnc.Hx))+real(down(mfnc.By))\*real(down(mfnc.Hy))+real(down(mfnc.Bz))\*real(down(mfnc.Hz)))+real(down(mfnc.By))\*(real(down(mfnc.Hx))\*mfnc.unx+real(down(mfnc.Hy))\*mfnc.uny+real(down(mfnc.Hz))\*mfnc.unz) | Pa | Maxwell downward magnetic surface stress tensor, y component | Boundaries 220–223, 228, 232, 234, 246, 248, 264 |
| mfnc.dnTmz | -0.5\*mfnc.unz\*(real(down(mfnc.Bx))\*real(down(mfnc.Hx))+real(down(mfnc.By))\*real(down(mfnc.Hy))+real(down(mfnc.Bz))\*real(down(mfnc.Hz)))+real(down(mfnc.Bz))\*(real(down(mfnc.Hx))\*mfnc.unx+real(down(mfnc.Hy))\*mfnc.uny+real(down(mfnc.Hz))\*mfnc.unz) | Pa | Maxwell downward magnetic surface stress tensor, z component | Boundaries 220–223, 228, 232, 234, 246, 248, 264 |
| mfnc.Hx | -Vmx | A/m | Magnetic field, x component | Domain 24 |
| mfnc.Hy | -Vmy | A/m | Magnetic field, y component | Domain 24 |
| mfnc.Hz | -Vmz | A/m | Magnetic field, z component | Domain 24 |
| mfnc.tHx | -VmTx | A/m | Tangential magnetic field, x component | Boundaries 220–223, 228, 232, 234, 246, 248, 264 |
| mfnc.tHy | -VmTy | A/m | Tangential magnetic field, y component | Boundaries 220–223, 228, 232, 234, 246, 248, 264 |
| mfnc.tHz | -VmTz | A/m | Tangential magnetic field, z component | Boundaries 220–223, 228, 232, 234, 246, 248, 264 |
| mfnc.normH | sqrt(realdot(mfnc.Hx,mfnc.Hx)+realdot(mfnc.Hy,mfnc.Hy)+realdot(mfnc.Hz,mfnc.Hz)) | A/m | Magnetic field norm | Domain 24 |
| mfnc.murxx | model.input.mur11 | 1 | Relative permeability, xx component | Domain 24 |
| mfnc.muryx | model.input.mur21 | 1 | Relative permeability, yx component | Domain 24 |
| mfnc.murzx | model.input.mur31 | 1 | Relative permeability, zx component | Domain 24 |
| mfnc.murxy | model.input.mur12 | 1 | Relative permeability, xy component | Domain 24 |
| mfnc.muryy | model.input.mur22 | 1 | Relative permeability, yy component | Domain 24 |
| mfnc.murzy | model.input.mur32 | 1 | Relative permeability, zy component | Domain 24 |
| mfnc.murxz | model.input.mur13 | 1 | Relative permeability, xz component | Domain 24 |
| mfnc.muryz | model.input.mur23 | 1 | Relative permeability, yz component | Domain 24 |
| mfnc.murzz | model.input.mur33 | 1 | Relative permeability, zz component | Domain 24 |
| mfnc.Bx | mu0\_const\*mfnc.Ixx\*mfnc.Hx+mu0\_const\*mfnc.Ixy\*mfnc.Hy+mu0\_const\*mfnc.Ixz\*mfnc.Hz+mu0\_const\*mfnc.chimxx\*mfnc.Hx+mu0\_const\*mfnc.chimxy\*mfnc.Hy+mu0\_const\*mfnc.chimxz\*mfnc.Hz+mfnc.Brx | T | Magnetic flux density, x component | Domain 24 |
| mfnc.By | mu0\_const\*mfnc.Iyx\*mfnc.Hx+mu0\_const\*mfnc.Iyy\*mfnc.Hy+mu0\_const\*mfnc.Iyz\*mfnc.Hz+mu0\_const\*mfnc.chimyx\*mfnc.Hx+mu0\_const\*mfnc.chimyy\*mfnc.Hy+mu0\_const\*mfnc.chimyz\*mfnc.Hz+mfnc.Bry | T | Magnetic flux density, y component | Domain 24 |
| mfnc.Bz | mu0\_const\*mfnc.Izx\*mfnc.Hx+mu0\_const\*mfnc.Izy\*mfnc.Hy+mu0\_const\*mfnc.Izz\*mfnc.Hz+mu0\_const\*mfnc.chimzx\*mfnc.Hx+mu0\_const\*mfnc.chimzy\*mfnc.Hy+mu0\_const\*mfnc.chimzz\*mfnc.Hz+mfnc.Brz | T | Magnetic flux density, z component | Domain 24 |
| mfnc.normB | sqrt(realdot(mfnc.Bx,mfnc.Bx)+realdot(mfnc.By,mfnc.By)+realdot(mfnc.Bz,mfnc.Bz)) | T | Magnetic flux density norm | Domain 24 |
| mfnc.Mx | mfnc.Bx/mu0\_const-mfnc.Ixx\*mfnc.Hx-mfnc.Ixy\*mfnc.Hy-mfnc.Ixz\*mfnc.Hz | A/m | Magnetization, x component | Domain 24 |
| mfnc.My | mfnc.By/mu0\_const-mfnc.Iyx\*mfnc.Hx-mfnc.Iyy\*mfnc.Hy-mfnc.Iyz\*mfnc.Hz | A/m | Magnetization, y component | Domain 24 |
| mfnc.Mz | mfnc.Bz/mu0\_const-mfnc.Izx\*mfnc.Hx-mfnc.Izy\*mfnc.Hy-mfnc.Izz\*mfnc.Hz | A/m | Magnetization, z component | Domain 24 |
| mfnc.normM | sqrt(realdot(mfnc.Mx,mfnc.Mx)+realdot(mfnc.My,mfnc.My)+realdot(mfnc.Mz,mfnc.Mz)) | A/m | Magnetization norm | Domain 24 |
| mfnc.Ixx | 1 | 1 | Spatial identity matrix, xx component | Domain 24 |
| mfnc.Iyx | 0 | 1 | Spatial identity matrix, yx component | Domain 24 |
| mfnc.Izx | 0 | 1 | Spatial identity matrix, zx component | Domain 24 |
| mfnc.Ixy | 0 | 1 | Spatial identity matrix, xy component | Domain 24 |
| mfnc.Iyy | 1 | 1 | Spatial identity matrix, yy component | Domain 24 |
| mfnc.Izy | 0 | 1 | Spatial identity matrix, zy component | Domain 24 |
| mfnc.Ixz | 0 | 1 | Spatial identity matrix, xz component | Domain 24 |
| mfnc.Iyz | 0 | 1 | Spatial identity matrix, yz component | Domain 24 |
| mfnc.Izz | 1 | 1 | Spatial identity matrix, zz component | Domain 24 |
| mfnc.chimxx | -1+mfnc.murxx | 1 | Magnetic susceptibility, xx component | Domain 24 |
| mfnc.chimyx | mfnc.muryx | 1 | Magnetic susceptibility, yx component | Domain 24 |
| mfnc.chimzx | mfnc.murzx | 1 | Magnetic susceptibility, zx component | Domain 24 |
| mfnc.chimxy | mfnc.murxy | 1 | Magnetic susceptibility, xy component | Domain 24 |
| mfnc.chimyy | -1+mfnc.muryy | 1 | Magnetic susceptibility, yy component | Domain 24 |
| mfnc.chimzy | mfnc.murzy | 1 | Magnetic susceptibility, zy component | Domain 24 |
| mfnc.chimxz | mfnc.murxz | 1 | Magnetic susceptibility, xz component | Domain 24 |
| mfnc.chimyz | mfnc.muryz | 1 | Magnetic susceptibility, yz component | Domain 24 |
| mfnc.chimzz | -1+mfnc.murzz | 1 | Magnetic susceptibility, zz component | Domain 24 |
| mfnc.Brx | BA\*cos(10\*pi/3) | T | Remanent flux density, x component | Domain 24 |
| mfnc.Bry | BA\*sin(10\*pi/3) | T | Remanent flux density, y component | Domain 24 |
| mfnc.Brz | 0 | T | Remanent flux density, z component | Domain 24 |
| mfnc.normBr | sqrt(realdot(mfnc.Brx,mfnc.Brx)+realdot(mfnc.Bry,mfnc.Bry)+realdot(mfnc.Brz,mfnc.Brz)) | T | Remanent flux density norm | Domain 24 |
| mfnc.unTx | 0 | Pa | Maxwell upward surface stress tensor, x component | Boundaries 220–223, 228, 232, 234, 246, 248, 264 |
| mfnc.unTy | 0 | Pa | Maxwell upward surface stress tensor, y component | Boundaries 220–223, 228, 232, 234, 246, 248, 264 |
| mfnc.unTz | 0 | Pa | Maxwell upward surface stress tensor, z component | Boundaries 220–223, 228, 232, 234, 246, 248, 264 |
| mfnc.dnTx | mfnc.dnTmx | Pa | Maxwell downward surface stress tensor, x component | Boundaries 220–223, 228, 232, 234, 246, 248, 264 |
| mfnc.dnTy | mfnc.dnTmy | Pa | Maxwell downward surface stress tensor, y component | Boundaries 220–223, 228, 232, 234, 246, 248, 264 |
| mfnc.dnTz | mfnc.dnTmz | Pa | Maxwell downward surface stress tensor, z component | Boundaries 220–223, 228, 232, 234, 246, 248, 264 |
| mfnc.unx | unx |  | Normal vector up direction, x component | Boundaries 220–223, 228, 232, 234, 246, 248, 264 |
| mfnc.uny | uny |  | Normal vector up direction, y component | Boundaries 220–223, 228, 232, 234, 246, 248, 264 |
| mfnc.unz | unz |  | Normal vector up direction, z component | Boundaries 220–223, 228, 232, 234, 246, 248, 264 |
| mfnc.dnx | dnx |  | Normal vector down direction, x component | Boundaries 220–223, 228, 232, 234, 246, 248, 264 |
| mfnc.dny | dny |  | Normal vector down direction, y component | Boundaries 220–223, 228, 232, 234, 246, 248, 264 |
| mfnc.dnz | dnz |  | Normal vector down direction, z component | Boundaries 220–223, 228, 232, 234, 246, 248, 264 |
| mfnc.W | mfnc.Wm | J/m^3 | Energy density | Domain 24 |
| mfnc.dWm | mfnc.Wm | J/m^3 | Integrand for total magnetic energy | Domain 24 |
| mfnc.Wm | 0.5\*mu0\_const\*((mfnc.murxx\*mfnc.Hx+mfnc.murxy\*mfnc.Hy+mfnc.murxz\*mfnc.Hz)\*mfnc.Hx+(mfnc.muryx\*mfnc.Hx+mfnc.muryy\*mfnc.Hy+mfnc.muryz\*mfnc.Hz)\*mfnc.Hy+(mfnc.murzx\*mfnc.Hx+mfnc.murzy\*mfnc.Hy+mfnc.murzz\*mfnc.Hz)\*mfnc.Hz) | J/m^3 | Magnetic energy density | Domain 24 |

##### Shape Functions

| Name | Shape function | Unit | Description | Shape frame | Selection |
| --- | --- | --- | --- | --- | --- |
| Vm | Lagrange (Quadratic) | A | Magnetic scalar potential | Material | Domain 24 |

##### Weak Expressions

| Weak expression | Integration frame | Selection |
| --- | --- | --- |
| mfnc.d\*(-mfnc.Bx\*test(Vmx)-mfnc.By\*test(Vmy)-mfnc.Bz\*test(Vmz)) | Material | Domain 24 |

#### 2.4.33. ArrayAMagnet12

ArrayAMagnet12

Selection

| Geometric entity level | Domain |
| Selection | Domain 30 |

Equations

Settings

| Description | Value |
| Constitutive relation | Remanent flux density |
| Remanent flux density, x component | BA\*cos(2\*pi\*(11\*60)/360) |
| Remanent flux density, y component | BA\*sin(2\*pi\*(11\*60)/360) |
| Remanent flux density, z component | 0 |
| Relative permeability | From material |
| Relative permeability | {{1, 0, 0}, {0, 1, 0}, {0, 0, 1}} |

Properties from material

| Property | Material | Property group |
| Relative permeability | Soft Iron (without losses) | Basic |

##### Variables

| Name | Expression | Unit | Description | Selection |
| --- | --- | --- | --- | --- |
| mfnc.dnTmx | -0.5\*mfnc.unx\*(real(down(mfnc.Bx))\*real(down(mfnc.Hx))+real(down(mfnc.By))\*real(down(mfnc.Hy))+real(down(mfnc.Bz))\*real(down(mfnc.Hz)))+real(down(mfnc.Bx))\*(real(down(mfnc.Hx))\*mfnc.unx+real(down(mfnc.Hy))\*mfnc.uny+real(down(mfnc.Hz))\*mfnc.unz) | Pa | Maxwell downward magnetic surface stress tensor, x component | Boundaries 282–285, 290, 292, 294, 296, 298, 307 |
| mfnc.dnTmy | -0.5\*mfnc.uny\*(real(down(mfnc.Bx))\*real(down(mfnc.Hx))+real(down(mfnc.By))\*real(down(mfnc.Hy))+real(down(mfnc.Bz))\*real(down(mfnc.Hz)))+real(down(mfnc.By))\*(real(down(mfnc.Hx))\*mfnc.unx+real(down(mfnc.Hy))\*mfnc.uny+real(down(mfnc.Hz))\*mfnc.unz) | Pa | Maxwell downward magnetic surface stress tensor, y component | Boundaries 282–285, 290, 292, 294, 296, 298, 307 |
| mfnc.dnTmz | -0.5\*mfnc.unz\*(real(down(mfnc.Bx))\*real(down(mfnc.Hx))+real(down(mfnc.By))\*real(down(mfnc.Hy))+real(down(mfnc.Bz))\*real(down(mfnc.Hz)))+real(down(mfnc.Bz))\*(real(down(mfnc.Hx))\*mfnc.unx+real(down(mfnc.Hy))\*mfnc.uny+real(down(mfnc.Hz))\*mfnc.unz) | Pa | Maxwell downward magnetic surface stress tensor, z component | Boundaries 282–285, 290, 292, 294, 296, 298, 307 |
| mfnc.Hx | -Vmx | A/m | Magnetic field, x component | Domain 30 |
| mfnc.Hy | -Vmy | A/m | Magnetic field, y component | Domain 30 |
| mfnc.Hz | -Vmz | A/m | Magnetic field, z component | Domain 30 |
| mfnc.tHx | -VmTx | A/m | Tangential magnetic field, x component | Boundaries 282–285, 290, 292, 294, 296, 298, 307 |
| mfnc.tHy | -VmTy | A/m | Tangential magnetic field, y component | Boundaries 282–285, 290, 292, 294, 296, 298, 307 |
| mfnc.tHz | -VmTz | A/m | Tangential magnetic field, z component | Boundaries 282–285, 290, 292, 294, 296, 298, 307 |
| mfnc.normH | sqrt(realdot(mfnc.Hx,mfnc.Hx)+realdot(mfnc.Hy,mfnc.Hy)+realdot(mfnc.Hz,mfnc.Hz)) | A/m | Magnetic field norm | Domain 30 |
| mfnc.murxx | model.input.mur11 | 1 | Relative permeability, xx component | Domain 30 |
| mfnc.muryx | model.input.mur21 | 1 | Relative permeability, yx component | Domain 30 |
| mfnc.murzx | model.input.mur31 | 1 | Relative permeability, zx component | Domain 30 |
| mfnc.murxy | model.input.mur12 | 1 | Relative permeability, xy component | Domain 30 |
| mfnc.muryy | model.input.mur22 | 1 | Relative permeability, yy component | Domain 30 |
| mfnc.murzy | model.input.mur32 | 1 | Relative permeability, zy component | Domain 30 |
| mfnc.murxz | model.input.mur13 | 1 | Relative permeability, xz component | Domain 30 |
| mfnc.muryz | model.input.mur23 | 1 | Relative permeability, yz component | Domain 30 |
| mfnc.murzz | model.input.mur33 | 1 | Relative permeability, zz component | Domain 30 |
| mfnc.Bx | mu0\_const\*mfnc.Ixx\*mfnc.Hx+mu0\_const\*mfnc.Ixy\*mfnc.Hy+mu0\_const\*mfnc.Ixz\*mfnc.Hz+mu0\_const\*mfnc.chimxx\*mfnc.Hx+mu0\_const\*mfnc.chimxy\*mfnc.Hy+mu0\_const\*mfnc.chimxz\*mfnc.Hz+mfnc.Brx | T | Magnetic flux density, x component | Domain 30 |
| mfnc.By | mu0\_const\*mfnc.Iyx\*mfnc.Hx+mu0\_const\*mfnc.Iyy\*mfnc.Hy+mu0\_const\*mfnc.Iyz\*mfnc.Hz+mu0\_const\*mfnc.chimyx\*mfnc.Hx+mu0\_const\*mfnc.chimyy\*mfnc.Hy+mu0\_const\*mfnc.chimyz\*mfnc.Hz+mfnc.Bry | T | Magnetic flux density, y component | Domain 30 |
| mfnc.Bz | mu0\_const\*mfnc.Izx\*mfnc.Hx+mu0\_const\*mfnc.Izy\*mfnc.Hy+mu0\_const\*mfnc.Izz\*mfnc.Hz+mu0\_const\*mfnc.chimzx\*mfnc.Hx+mu0\_const\*mfnc.chimzy\*mfnc.Hy+mu0\_const\*mfnc.chimzz\*mfnc.Hz+mfnc.Brz | T | Magnetic flux density, z component | Domain 30 |
| mfnc.normB | sqrt(realdot(mfnc.Bx,mfnc.Bx)+realdot(mfnc.By,mfnc.By)+realdot(mfnc.Bz,mfnc.Bz)) | T | Magnetic flux density norm | Domain 30 |
| mfnc.Mx | mfnc.Bx/mu0\_const-mfnc.Ixx\*mfnc.Hx-mfnc.Ixy\*mfnc.Hy-mfnc.Ixz\*mfnc.Hz | A/m | Magnetization, x component | Domain 30 |
| mfnc.My | mfnc.By/mu0\_const-mfnc.Iyx\*mfnc.Hx-mfnc.Iyy\*mfnc.Hy-mfnc.Iyz\*mfnc.Hz | A/m | Magnetization, y component | Domain 30 |
| mfnc.Mz | mfnc.Bz/mu0\_const-mfnc.Izx\*mfnc.Hx-mfnc.Izy\*mfnc.Hy-mfnc.Izz\*mfnc.Hz | A/m | Magnetization, z component | Domain 30 |
| mfnc.normM | sqrt(realdot(mfnc.Mx,mfnc.Mx)+realdot(mfnc.My,mfnc.My)+realdot(mfnc.Mz,mfnc.Mz)) | A/m | Magnetization norm | Domain 30 |
| mfnc.Ixx | 1 | 1 | Spatial identity matrix, xx component | Domain 30 |
| mfnc.Iyx | 0 | 1 | Spatial identity matrix, yx component | Domain 30 |
| mfnc.Izx | 0 | 1 | Spatial identity matrix, zx component | Domain 30 |
| mfnc.Ixy | 0 | 1 | Spatial identity matrix, xy component | Domain 30 |
| mfnc.Iyy | 1 | 1 | Spatial identity matrix, yy component | Domain 30 |
| mfnc.Izy | 0 | 1 | Spatial identity matrix, zy component | Domain 30 |
| mfnc.Ixz | 0 | 1 | Spatial identity matrix, xz component | Domain 30 |
| mfnc.Iyz | 0 | 1 | Spatial identity matrix, yz component | Domain 30 |
| mfnc.Izz | 1 | 1 | Spatial identity matrix, zz component | Domain 30 |
| mfnc.chimxx | -1+mfnc.murxx | 1 | Magnetic susceptibility, xx component | Domain 30 |
| mfnc.chimyx | mfnc.muryx | 1 | Magnetic susceptibility, yx component | Domain 30 |
| mfnc.chimzx | mfnc.murzx | 1 | Magnetic susceptibility, zx component | Domain 30 |
| mfnc.chimxy | mfnc.murxy | 1 | Magnetic susceptibility, xy component | Domain 30 |
| mfnc.chimyy | -1+mfnc.muryy | 1 | Magnetic susceptibility, yy component | Domain 30 |
| mfnc.chimzy | mfnc.murzy | 1 | Magnetic susceptibility, zy component | Domain 30 |
| mfnc.chimxz | mfnc.murxz | 1 | Magnetic susceptibility, xz component | Domain 30 |
| mfnc.chimyz | mfnc.muryz | 1 | Magnetic susceptibility, yz component | Domain 30 |
| mfnc.chimzz | -1+mfnc.murzz | 1 | Magnetic susceptibility, zz component | Domain 30 |
| mfnc.Brx | BA\*cos(11\*pi/3) | T | Remanent flux density, x component | Domain 30 |
| mfnc.Bry | BA\*sin(11\*pi/3) | T | Remanent flux density, y component | Domain 30 |
| mfnc.Brz | 0 | T | Remanent flux density, z component | Domain 30 |
| mfnc.normBr | sqrt(realdot(mfnc.Brx,mfnc.Brx)+realdot(mfnc.Bry,mfnc.Bry)+realdot(mfnc.Brz,mfnc.Brz)) | T | Remanent flux density norm | Domain 30 |
| mfnc.unTx | 0 | Pa | Maxwell upward surface stress tensor, x component | Boundaries 282–285, 290, 292, 294, 296, 298, 307 |
| mfnc.unTy | 0 | Pa | Maxwell upward surface stress tensor, y component | Boundaries 282–285, 290, 292, 294, 296, 298, 307 |
| mfnc.unTz | 0 | Pa | Maxwell upward surface stress tensor, z component | Boundaries 282–285, 290, 292, 294, 296, 298, 307 |
| mfnc.dnTx | mfnc.dnTmx | Pa | Maxwell downward surface stress tensor, x component | Boundaries 282–285, 290, 292, 294, 296, 298, 307 |
| mfnc.dnTy | mfnc.dnTmy | Pa | Maxwell downward surface stress tensor, y component | Boundaries 282–285, 290, 292, 294, 296, 298, 307 |
| mfnc.dnTz | mfnc.dnTmz | Pa | Maxwell downward surface stress tensor, z component | Boundaries 282–285, 290, 292, 294, 296, 298, 307 |
| mfnc.unx | unx |  | Normal vector up direction, x component | Boundaries 282–285, 290, 292, 294, 296, 298, 307 |
| mfnc.uny | uny |  | Normal vector up direction, y component | Boundaries 282–285, 290, 292, 294, 296, 298, 307 |
| mfnc.unz | unz |  | Normal vector up direction, z component | Boundaries 282–285, 290, 292, 294, 296, 298, 307 |
| mfnc.dnx | dnx |  | Normal vector down direction, x component | Boundaries 282–285, 290, 292, 294, 296, 298, 307 |
| mfnc.dny | dny |  | Normal vector down direction, y component | Boundaries 282–285, 290, 292, 294, 296, 298, 307 |
| mfnc.dnz | dnz |  | Normal vector down direction, z component | Boundaries 282–285, 290, 292, 294, 296, 298, 307 |
| mfnc.W | mfnc.Wm | J/m^3 | Energy density | Domain 30 |
| mfnc.dWm | mfnc.Wm | J/m^3 | Integrand for total magnetic energy | Domain 30 |
| mfnc.Wm | 0.5\*mu0\_const\*((mfnc.murxx\*mfnc.Hx+mfnc.murxy\*mfnc.Hy+mfnc.murxz\*mfnc.Hz)\*mfnc.Hx+(mfnc.muryx\*mfnc.Hx+mfnc.muryy\*mfnc.Hy+mfnc.muryz\*mfnc.Hz)\*mfnc.Hy+(mfnc.murzx\*mfnc.Hx+mfnc.murzy\*mfnc.Hy+mfnc.murzz\*mfnc.Hz)\*mfnc.Hz) | J/m^3 | Magnetic energy density | Domain 30 |

##### Shape Functions

| Name | Shape function | Unit | Description | Shape frame | Selection |
| --- | --- | --- | --- | --- | --- |
| Vm | Lagrange (Quadratic) | A | Magnetic scalar potential | Material | Domain 30 |

##### Weak Expressions

| Weak expression | Integration frame | Selection |
| --- | --- | --- |
| mfnc.d\*(-mfnc.Bx\*test(Vmx)-mfnc.By\*test(Vmy)-mfnc.Bz\*test(Vmz)) | Material | Domain 30 |

### 2.5. Mesh 1

Mesh statistics

| Property | Value |
| Minimum element quality | 0.008909 |
| Average element quality | 0.7156 |
| Tetrahedral elements | 6739177 |
| Triangular elements | 335544 |
| Edge elements | 44176 |
| Vertex elements | 496 |

Mesh 1

#### 2.5.1. Size (Size)

Settings

| Name | Value |
| Maximum element size | 0.021 |
| Minimum element size | 9.0E-4 |
| Curvature factor | 0.3 |
| Resolution of narrow regions | 0.85 |
| Maximum element growth rate | 1.35 |
| Predefined size | Extra fine |

#### 2.5.2. Free Tetrahedral 2 (Ftet2)

Selection

| Geometric entity level | Domain |
| Selection | No domains |

Free Tetrahedral 2

##### Size 1 (Size1)

Selection

| Geometric entity level | Domain |
| Selection | Domain 15 |

Size 1

Settings

| Name | Value |
| Maximum element size | 0.0005 |
| Minimum element size | 1.0E-4 |
| Curvature factor | 0.3 |
| Curvature factor | Off |
| Resolution of narrow regions | 0.85 |
| Resolution of narrow regions | Off |
| Maximum element growth rate | 1.35 |
| Maximum element growth rate | Off |
| Predefined size | Extra fine |
| Custom element size | Custom |

#### 2.5.3. Free Tetrahedral 3 (Ftet3)

Selection

| Geometric entity level | Remaining |

##### Size 1 (Size1)

Selection

| Geometric entity level | Domain |
| Selection | Domains 2–14, 16–32 |

Size 1

Settings

| Name | Value |
| Maximum element size | 0.05 |
| Minimum element size | 0.006 |
| Curvature factor | 0.5 |
| Curvature factor | Off |
| Resolution of narrow regions | 0.6 |
| Resolution of narrow regions | Off |
| Maximum element growth rate | 1.45 |
| Maximum element growth rate | Off |
| Predefined size | Fine |
| Custom element size | Custom |

#### 2.5.4. Free Tetrahedral 4 (Ftet4)

Selection

| Geometric entity level | Remaining |

##### Size 1 (Size1)

Selection

| Geometric entity level | Domain |
| Selection | Geometry geom1 |

Size 1

Settings

| Name | Value |
| Maximum element size | 0.06 |
| Minimum element size | 0.0108 |
| Curvature factor | 0.6 |
| Resolution of narrow regions | 0.5 |
| Maximum element growth rate | 1.5 |

## 3. Study 1

### 3.1. Stationary

Study settings

| Property | Value |
| Include geometric nonlinearity | Off |

Physics and variables selection

| Physics interface | Discretization |
| Magnetic Fields, No Currents (mfnc) | physics |

Mesh selection

| Geometry | Mesh |
| Geometry 1 (geom1) | mesh1 |

### 3.2. Solver Configurations

#### 3.2.1. Solution 1

##### Compile Equations: Stationary (St1)

Study and step

| Name | Value |
| Use study | Study 1 |
| Use study step | Stationary |

##### Dependent Variables 1 (V1)

General

| Name | Value |
| Defined by study step | Stationary |

Initial values of variables solved for

| Name | Value |
| Solution | Zero |

Values of variables not solved for

| Name | Value |
| Solution | Zero |

###### Magnetic Scalar Potential (Comp1.Vm) (Comp1\_Vm)

General

| Name | Value |
| Field components | comp1.Vm |

##### Stationary Solver 1 (S1)

General

| Name | Value |
| Defined by study step | Stationary |

Log

Stationary Solver 1 in Solution 1 started at 2-Mar-2016 11:33:03.

Linear solver

Number of degrees of freedom solved for: 8989380.

Symmetric matrices found.

Scales for dependent variables:

Magnetic scalar potential (comp1.Vm): 1

Orthonormal null-space function used.

Iter      SolEst     Damping    Stepsize #Res #Jac #Sol LinIt   LinErr   LinRes

1        0.96   1.0000000        0.96    1    1    1    28  0.00064 1.8e-006

Stationary Solver 1 in Solution 1: Solution time: 260 s (4 minutes, 20 seconds)

Physical memory: 13.79 GB

Virtual memory: 14.29 GB

###### Fully Coupled 1 (Fc1)

General

| Name | Value |
| Linear solver | Iterative 1 |

###### Iterative 1 (I1)

General

| Name | Value |
| Solver | Conjugate gradients |

###### Multigrid 1 (Mg1)

General

| Name | Value |
| Solver | Algebraic multigrid |

## 4. Results

### 4.1. Data Sets

#### 4.1.1. Study 1/Solution 1

Solution

| Name | Value |
| Solution | Solution 1 |
| Component | Save Point Geometry 1 |

Data set: Study 1/Solution 1

#### 4.1.2. Cut Plane XY

Data

| Name | Value |
| Data set | Study 1/Solution 1 |

Plane data

| Name | Value |
| Plane type | Quick |
| Plane | xy - planes |
| z-coordinate | 0.075 |

Advanced

| Name | Value |
| Space variables | {cpl1x, cpl1y} |

Data set: Cut Plane XY

#### 4.1.3. AlongXAxis

Data

| Name | Value |
| Data set | Cut Plane XY |

Line data

| Name | Value |
| Line entry method | Two points |
| Points | {{-0.15, 0}, {0.15, 0}} |

Advanced

| Name | Value |
| Space variable | cln1x |

Data set: AlongXAxis

#### 4.1.4. AlongYAxis

Data

| Name | Value |
| Data set | Cut Plane XY |

Line data

| Name | Value |
| Line entry method | Two points |
| Points | {{0, -0.15}, {0, 0.15}} |

Advanced

| Name | Value |
| Space variable | cln2x |

Data set: AlongYAxis

#### 4.1.5. Cut Plane YZ

Data

| Name | Value |
| Data set | Study 1/Solution 1 |

Plane data

| Name | Value |
| Plane type | Quick |
| x-coordinate | 0 |

Advanced

| Name | Value |
| Space variables | {cpl2x, cpl2y} |

Data set: Cut Plane YZ

#### 4.1.6. AlongZAxis

Data

| Name | Value |
| Data set | Study 1/Solution 1 |

Line data

| Name | Value |
| Line entry method | Two points |
| Points | {{0, 0, -0.15}, {0, 0, 0.15}} |

Advanced

| Name | Value |
| Space variable | cln3x |

Data set: AlongZAxis

#### 4.1.7. Cut Point 2D 1

Data

| Name | Value |
| Data set | Cut Plane XY |

Point data

| Name | Value |
| Entry method | Grid |

Settings

| Name | Value |
| x | {-0.02, -0.01, 0, 0.009999999999999998, 0.02} |
| y | {-0.02, -0.01, 0, 0.009999999999999998, 0.02} |

Data set: Cut Point 2D 1

### 4.2. Tables

#### 4.2.1. Table 1

Point Evaluation 1 (mfnc.normB)

Table 1

| Magnetic flux density norm (T), Point: (-0.02, -0.02) | Magnetic flux density norm (T), Point: (-0.01, -0.01) | Magnetic flux density norm (T), Point: (0, 0) | Magnetic flux density norm (T), Point: (0.01, 0.01) | Magnetic flux density norm (T), Point: (0.02, 0.02) | Magnetic flux density norm (T), Point: (-0.02, -0.02) | Magnetic flux density norm (T), Point: (-0.01, -0.02) | Magnetic flux density norm (T), Point: (0, -0.02) | Magnetic flux density norm (T), Point: (0.01, -0.02) | Magnetic flux density norm (T), Point: (0.02, -0.02) | Magnetic flux density norm (T), Point: (-0.02, -0.01) | Magnetic flux density norm (T), Point: (-0.01, -0.01) | Magnetic flux density norm (T), Point: (0, -0.01) | Magnetic flux density norm (T), Point: (0.01, -0.01) | Magnetic flux density norm (T), Point: (0.02, -0.01) | Magnetic flux density norm (T), Point: (-0.02, 0) | Magnetic flux density norm (T), Point: (-0.01, 0) | Magnetic flux density norm (T), Point: (0, 0) | Magnetic flux density norm (T), Point: (0.01, 0) | Magnetic flux density norm (T), Point: (0.02, 0) | Magnetic flux density norm (T), Point: (-0.02, 0.01) | Magnetic flux density norm (T), Point: (-0.01, 0.01) | Magnetic flux density norm (T), Point: (0, 0.01) | Magnetic flux density norm (T), Point: (0.01, 0.01) | Magnetic flux density norm (T), Point: (0.02, 0.01) | Magnetic flux density norm (T), Point: (-0.02, 0.02) | Magnetic flux density norm (T), Point: (-0.01, 0.02) | Magnetic flux density norm (T), Point: (0, 0.02) | Magnetic flux density norm (T), Point: (0.01, 0.02) | Magnetic flux density norm (T), Point: (0.02, 0.02) | Magnetic flux density norm (T), Point: (-0.02, -0.02) | Magnetic flux density norm (T), Point: (-0.01, -0.02) | Magnetic flux density norm (T), Point: (0, -0.02) | Magnetic flux density norm (T), Point: (0.01, -0.02) | Magnetic flux density norm (T), Point: (0.02, -0.02) | Magnetic flux density norm (T), Point: (-0.02, -0.01) | Magnetic flux density norm (T), Point: (-0.01, -0.01) | Magnetic flux density norm (T), Point: (0, -0.01) | Magnetic flux density norm (T), Point: (0.01, -0.01) | Magnetic flux density norm (T), Point: (0.02, -0.01) | Magnetic flux density norm (T), Point: (-0.02, 0) | Magnetic flux density norm (T), Point: (-0.01, 0) | Magnetic flux density norm (T), Point: (0, 0) | Magnetic flux density norm (T), Point: (0.01, 0) | Magnetic flux density norm (T), Point: (0.02, 0) | Magnetic flux density norm (T), Point: (-0.02, 0.01) | Magnetic flux density norm (T), Point: (-0.01, 0.01) | Magnetic flux density norm (T), Point: (0, 0.01) | Magnetic flux density norm (T), Point: (0.01, 0.01) | Magnetic flux density norm (T), Point: (0.02, 0.01) | Magnetic flux density norm (T), Point: (-0.02, 0.02) | Magnetic flux density norm (T), Point: (-0.01, 0.02) | Magnetic flux density norm (T), Point: (0, 0.02) | Magnetic flux density norm (T), Point: (0.01, 0.02) | Magnetic flux density norm (T), Point: (0.02, 0.02) | Magnetic flux density norm (T), Point: (-0.02, -0.02) | Magnetic flux density norm (T), Point: (-0.01, -0.02) | Magnetic flux density norm (T), Point: (0, -0.02) | Magnetic flux density norm (T), Point: (0.01, -0.02) | Magnetic flux density norm (T), Point: (0.02, -0.02) | Magnetic flux density norm (T), Point: (-0.02, -0.01) | Magnetic flux density norm (T), Point: (-0.01, -0.01) | Magnetic flux density norm (T), Point: (0, -0.01) | Magnetic flux density norm (T), Point: (0.01, -0.01) | Magnetic flux density norm (T), Point: (0.02, -0.01) | Magnetic flux density norm (T), Point: (-0.02, 0) | Magnetic flux density norm (T), Point: (-0.01, 0) | Magnetic flux density norm (T), Point: (0, 0) | Magnetic flux density norm (T), Point: (0.01, 0) | Magnetic flux density norm (T), Point: (0.02, 0) | Magnetic flux density norm (T), Point: (-0.02, 0.01) | Magnetic flux density norm (T), Point: (-0.01, 0.01) | Magnetic flux density norm (T), Point: (0, 0.01) | Magnetic flux density norm (T), Point: (0.01, 0.01) | Magnetic flux density norm (T), Point: (0.02, 0.01) | Magnetic flux density norm (T), Point: (-0.02, 0.02) | Magnetic flux density norm (T), Point: (-0.01, 0.02) | Magnetic flux density norm (T), Point: (0, 0.02) | Magnetic flux density norm (T), Point: (0.01, 0.02) | Magnetic flux density norm (T), Point: (0.02, 0.02) |
| 0.012518 | 0.012282 | 0.012188 | 0.012279 | 0.012506 | 0.012518 | 0.012304 | 0.012282 | 0.012306 | 0.012475 | 0.012467 | 0.012282 | 0.012221 | 0.012273 | 0.012477 | 0.012421 | 0.012265 | 0.012188 | 0.012270 | 0.012413 | 0.012488 | 0.012277 | 0.012186 | 0.012279 | 0.012483 | 0.012515 | 0.012306 | 0.012275 | 0.012319 | 0.012506 | 0.013655 | 0.013496 | 0.013430 | 0.013518 | 0.013639 | 0.013536 | 0.013425 | 0.013368 | 0.013419 | 0.013543 | 0.013554 | 0.013406 | 0.013334 | 0.013410 | 0.013574 | 0.013558 | 0.013417 | 0.013342 | 0.013422 | 0.013575 | 0.013647 | 0.013488 | 0.013428 | 0.013499 | 0.013644 | 0.013655 | 0.013496 | 0.013430 | 0.013518 | 0.013639 | 0.013536 | 0.013425 | 0.013368 | 0.013419 | 0.013543 | 0.013554 | 0.013406 | 0.013334 | 0.013410 | 0.013574 | 0.013558 | 0.013417 | 0.013342 | 0.013422 | 0.013575 | 0.013647 | 0.013488 | 0.013428 | 0.013499 | 0.013644 |

### 4.3. Plot Groups

#### 4.3.1. 1D Plot Group 1

Line Graph: Magnetic flux density, x component (T)

#### 4.3.2. 2D Plot Group 2

Arrow Surface: Magnetic flux density
