# Supplementary figures and images for: Rotatable Small Permanent Magnet Array for Ultra-Low Field Nuclear Magnetic Resonance Instrumentation: A Concept Study (part 2 of 2)
Source: PLoS One. 2016 Jun 6;11(6):e0157040. doi: 10.1371/journal.pone.0157040 (PMC4894570; doi:10.1371/journal.pone.0157040)

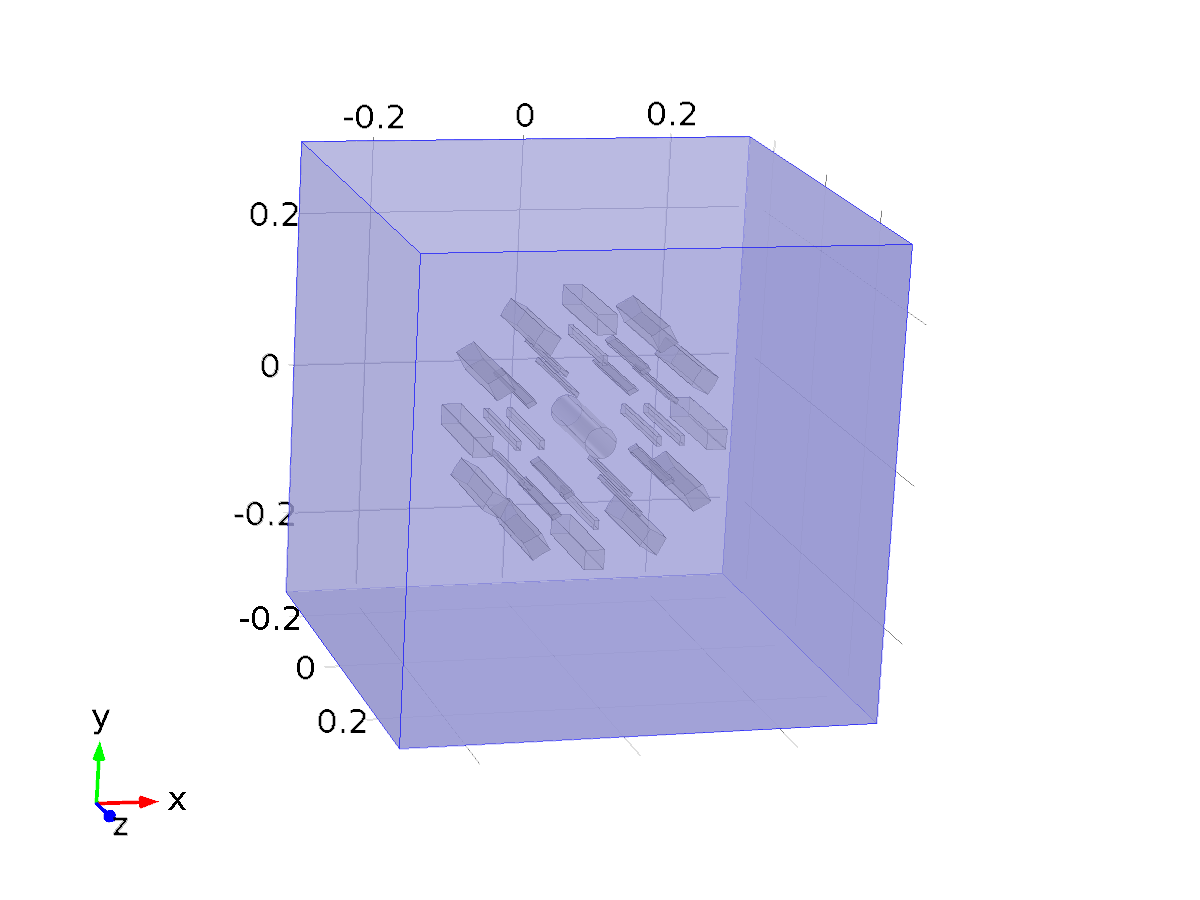

Supplement: S2 File — Model documentation generated by COMSOL with implemented parameters for the manual prototype. (ZIP) [file pone.0157040.s002.zip › SPMA_Rectangle_files/physics_mfnc_mi1.png]

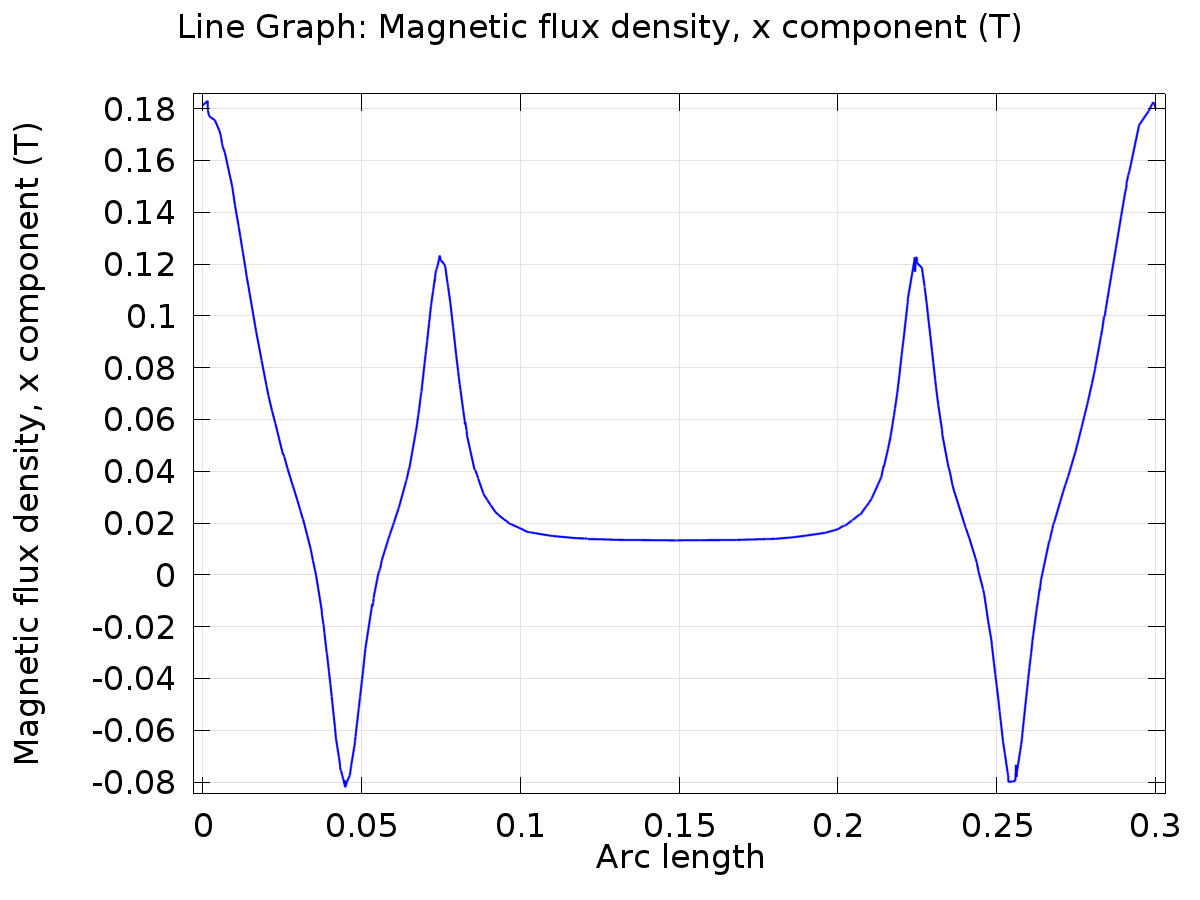

Supplement: S2 File — Model documentation generated by COMSOL with implemented parameters for the manual prototype. (ZIP) [file pone.0157040.s002.zip › SPMA_Rectangle_files/plotgroup_pg1.png]

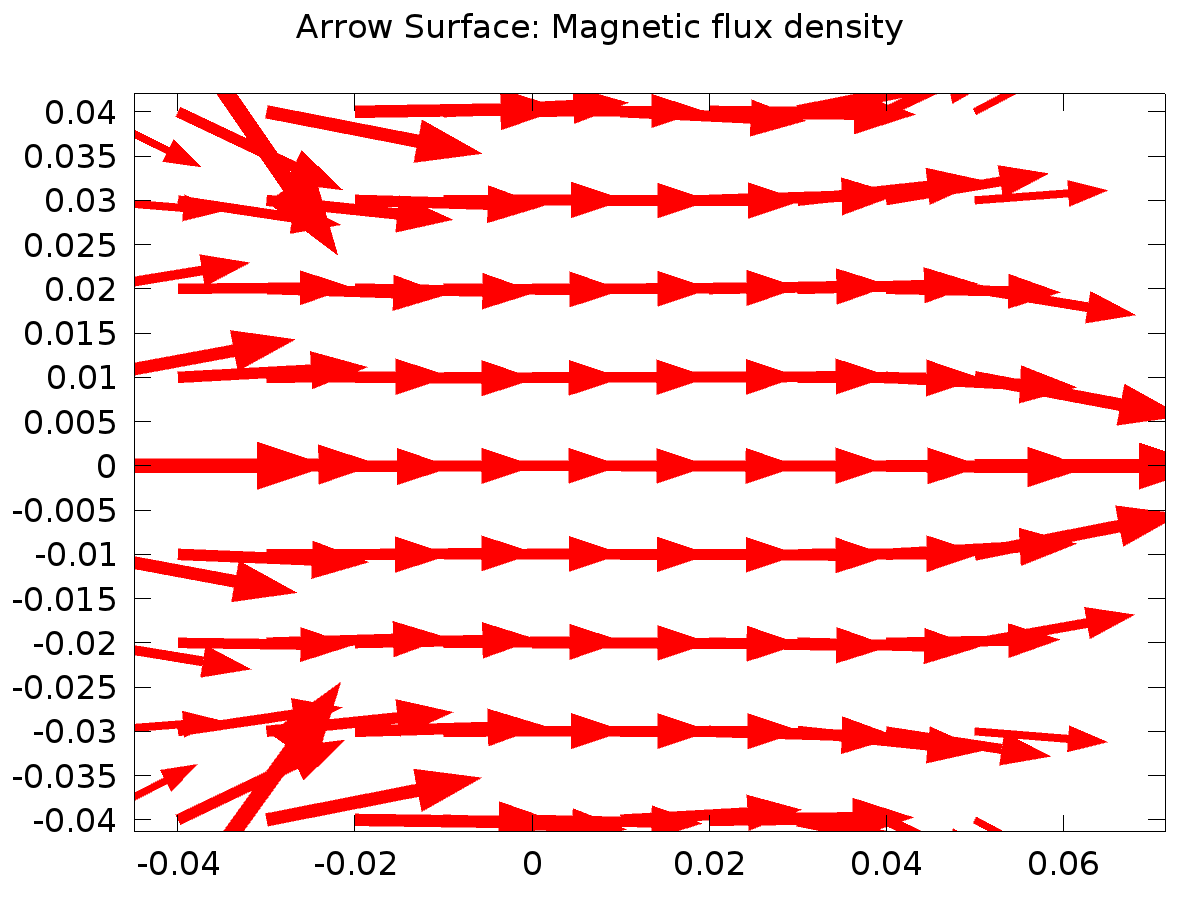

Supplement: S2 File — Model documentation generated by COMSOL with implemented parameters for the manual prototype. (ZIP) [file pone.0157040.s002.zip › SPMA_Rectangle_files/plotgroup_pg2.png]
